# Azirinyl-Substituted Nitrile Oxides: Generation and Use in the Synthesis of Isoxazole Containing Heterocyclic Hybrids

**DOI:** 10.3390/molecules30132834

**Published:** 2025-07-02

**Authors:** Alexander S. Dudik, Timur O. Zanakhov, Ekaterina E. Galenko, Mikhail S. Novikov, Alexander F. Khlebnikov

**Affiliations:** Institute of Chemistry, St. Petersburg State University, 7/9 Universitetskaya Naberezhnaya, St. Petersburg 199034, Russia; alexdudik28@gmail.com (A.S.D.); st078891@student.spbu.ru (T.O.Z.); ekaterina.e.galenko@gmail.com (E.E.G.); m.novikov@spbu.ru (M.S.N.)

**Keywords:** azirines, nitrile oxides, diazo compounds, isoxazoles, pyrroles, oxazoles, heterocyclic hybrids

## Abstract

The procedure for the generation of azirinyl-substituted nitrile oxides by the reaction of 2-(diazoacetyl)-2*H*-azirines with *tert*-butyl nitrite while preserving the azirine ring has been developed. The [3+2] cycloaddition of azirinyl-substituted nitrile oxides to terminal acetylenes produced azirinyl(isoxazolyl)ketones with various substituents in position 3 of azirine and position 5 of isoxazole fragments in a 51–91% yield at room temperature in DCM. DFT calculations and experimental data are consistent with the assumption that the formation of azirinyl-substituted nitrile oxides is accelerated by the acid catalyst. Cycloadducts of nitrile oxides with aryl/hetarylacetylenes and DMAD can be obtained by catalysis with boron trifluoride etherate, which significantly expands the scope of application of the reaction. Expansion of the azirine ring of the prepared cycloadducts allows obtaining a wide range of structurally diverse functionalized isoxazole-containing heterocyclic hybrids. LED light induces isomerization of the azirinecarbonyl moiety of the azirinyl(isoxazolyl)ketones, resulting in the formation of a set of 3,5’-biisoxazoles in a 40–71% yield, while the catalytic reaction of the azirine moiety with 1,3-diketones opens the way to pyrrole- and isoxazole-containing hybrids. 2-(Isoxazole-3-ylcarbonyl)-3-arylazirines were also easily isomerized to 3-(oxazol-5-yl)isoxazoles in methanol in the presence of excess potassium carbonate at room temperature.

## 1. Introduction

**Scheme 1:**
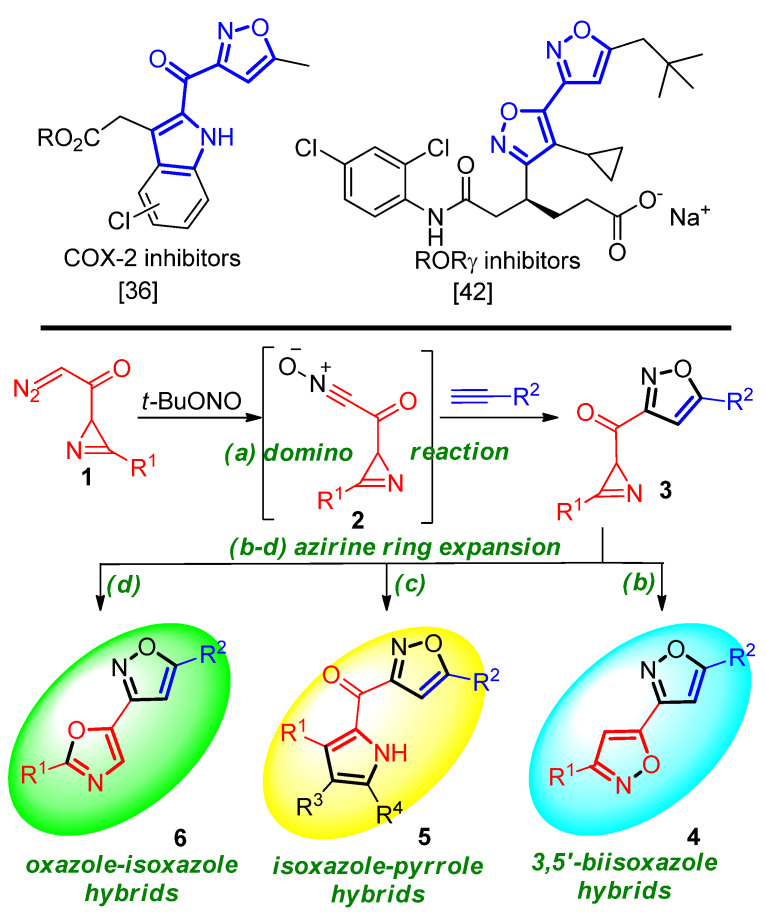
Bioactive isoxazole-containing hybrids and azirinyl-substituted nitrile oxide approach to such hybrids.

Isoxazoles are an important class of heterocyclic compounds, and their structural units are found in a wide range of natural and synthetic products and bioactive molecules. This is evidenced by the fact that many reviews have been published recently devoted to various aspects of the biological activity of isoxazole-containing compounds [1,2,3,4,5,6,7,8,9]. Due to their broad spectrum of pharmacological activity, they have been used to develop several commercially available drugs [1,2,3,4,5,6,7,8,9]. It should be emphasized that many of the drugs used, as well as the bioactive compounds studied, are isoxazole-containing heterocyclic hybrids. This is due to the fact that hybrid molecules, which are a combination of pharmacologically significant heterocycles acting on different biological targets, are currently an important strategy in drug development aimed at maximizing efficacy, minimizing side effects, and combating drug resistance [10,11,12,13,14,15]. Thus, in addition to the isoxazole fragment, such drugs as Paliperidone and Risperidone contain piperidine and pyrimidine fragments; Tivozanib contains a quinoline fragment; Sitaxentan contains a thiophene fragment; Pleconaril contains a 1,2,4-oxadiazole fragment; and Flucloxacillin, Dicloxacillin, Oxacillin, and Cloxacillin contain a 4-thia-1-azabicyclo[3.2.0]heptane fragment [1]. In addition, in order to find new therapeutic agents, a number of molecular hybrids containing an isoxazole moiety, such as isoxazole-piperazine [16], isoxazole-tacrine [17] and isoxazole-chromenone [18], and isoxazole-benzylpyridinium [19], have been synthesized, demonstrating selective inhibition of acetylcholinesterase, useful for the treatment of Alzheimer’s disease [16,17,18,19,20]. Isoxazole-indole [21], isoxazole-tetrahydroquinoline [22], isoxazole-acridine [23], isoxazole-oxazole [24], and isoxazole-oxazole-1,3,4-oxadiazole [25] hybrids exhibit antitumor properties. Isoxazole-1,2,4-oxadiazole hybrids have anti-HIV activity [26], and isoxazole–mercaptobenzimidazole hybrids are analgesic and anti-inflammatory agents [27]. All this suggests the need to develop new methods for the synthesis of isoxazoles that provide their diverse functionalization and preparation of new heterocyclic hybrids [4,7,28,29,30,31,32]. Although a large number of heterocyclic hybrids containing isoxazole have already been synthesized, only a few compounds have been obtained among isoxazole–pyrrole hybrids, where the heterocycles are linked by a carbonyl bridge [33,34,35]. Among such hybrids, derivatives of (isoxazol-3-yl)(1*H*-pyrrol-2-yl)ketone have not yet been synthesized, although their benzo analogues, (1*H*-indol-2-yl)(isoxazol-3-yl) ketones, are patented as COX-2 inhibitors (Scheme 1) [36]. Of the bisoxazoles in which the two isoxazole rings are directly linked, only a few examples have been published for 3,3’ [37], 3,4’ [38], 4,4′ [39], and 5,5′-biisoxazoles [40,41]. The 3,5’-biisoxazole fragment is part of the molecule of potent and metabolically stable RORγ inhibitors (Scheme 1) [42]. Of the oxazolyl–isoxazole hybrids, only a few examples have been published for 5-(oxazol-4-yl)isoxazole derivatives with anti-tubercular activity [43] and Salmonella thyphimurium serine acetyltransferase inhibitors [44], and for a 4-(oxazol-2-yl)isoxazole derivative with antidepressant and sedative activity [45]. Meanwhile, the synthesis of derivatives of 3-(oxazol-5-yl)isoxazole has not been published in the scientific literature.

We hypothesized that if the recently developed method for converting diazoketones to nitrile oxides by the action of *tert*-butyl nitrite (TBN) [46] could be applied to 2-(diazoacetyl)-2*H*-azirines, the azirinyl-substituted nitrile oxides thus generated could serve as a basis for the development of methods for synthesizing various isoxazole-containing hybrids (Scheme 1). This approach can be implemented due to the fact that (diazoacetyl)azirines **1** can react in orthogonal and domino modes, as demonstrated previously [47,48,49,50,51]. We report herein the development of a process for the generation of azirinyl-substituted nitrile oxides **2** with retention of the azirine ring; their [3+2] cycloaddition to acetylenes to form azirinyl-substituted isoxazoles **3**; and expansion of the azirine ring of **3** to isoxazoles **4**, pyrroles **5**, and oxazoles **6**. The developed methods open up ways to the synthesis of a wide range of structurally diverse functionalized heterocyclic hybrids (Scheme 1).

## 2. Results and Discussion

We began this study by reacting 2-(diazoacetyl)-2*H*-azirine **1a** with TBN in the presence of the active 2-π component, methyl propiolate (**7a**), to trap nitrile oxide **2a**. Carrying out the reaction in chloroform, as in the original paper [46], allowed isoxazole **3a** to be obtained in a 77% yield in 1 d (Table 1). As a result of minor optimization, it was found that the use of methylene chloride as a solvent allows increasing the yield of isoxazole **3a** to 91%. It should also be noted that the formation of furoxan dimer **8a** was not observed in any of the experiments, including the reaction of **1a** with TBN in the absence of a 2-π component (cf. [46]).

**Scheme 2:**
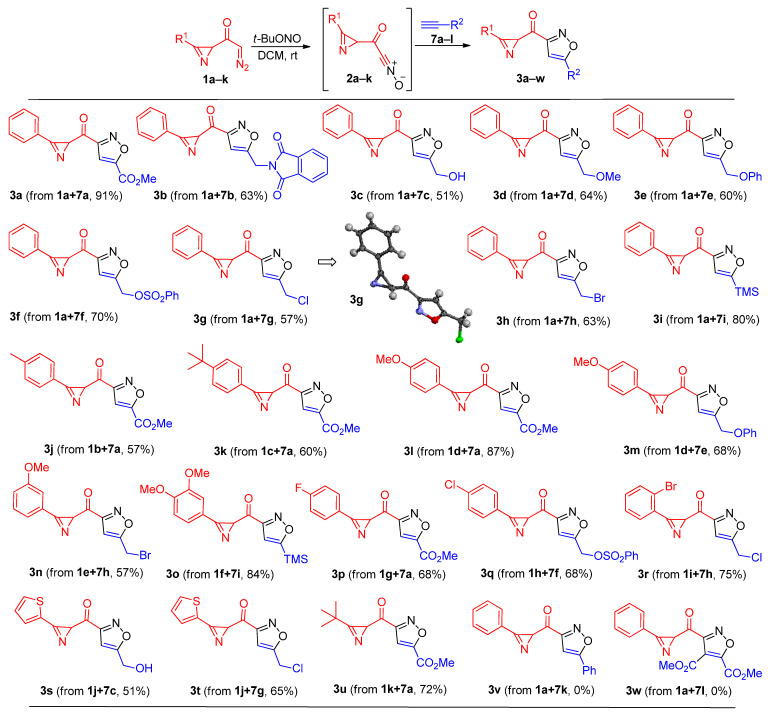
Synthesis of 3-(azirinylcarbonyl)isoxazoles **3**.

Using the found optimal conditions, diazoacetyl azirines **1a**–**i**, containing various *o*-, *m*-, and *p*-substituents in the 3-phenyl group, as well as 3-(thiophene-2-yl)- (**1j**) and 3-*tert*-butyl- (**1k**) 2-(diazoacetyl)-2*H*-azirines, were reacted with TBN in the presence of terminal acetylenes **7a**–**l** to form isoxazoles **3a**–**u** in 51–91% yields (Scheme 2). The reaction at room temperature requires 1 to 3 days for complete conversion of the starting azirine **1**. It was also found that the reaction with phenylacetylene (**7k**) at room temperature did not proceed, and increasing the temperature resulted in resinification of the reaction mixture. We were also unable to trap nitrile oxide **2a** using internal acetylene such as DMAD (**7l**).

Compounds **3** were characterized by ^1^H and ^13^C NMR and HRMS methods. ^1^H NMR spectra of compounds **3** contain characteristic signals of the azirine proton at 2-C of azirine in the region of 4.0–5.0 ppm (singlet) and the isoxazole proton at 4-C in the region of 6.5–7.5 ppm (singlet). ^13^C NMR spectra contain characteristic signals of the 2-C azirine carbon nucleus in the region of 34.0–40.0 ppm and the 4-C isoxazole carbon nucleus in the region of 100–115 ppm. The IR spectrum of compound **3a** contains characteristic absorption bands of C=O stretching of carbonyl and ester groups near 1680 and 1740 cm^−1^, respectively. The structure of compound **3g** was confirmed by X-ray structural analysis (CCDC 2455839). Substances **3a**–**u** are light-brown oils or yellowish crystals, readily soluble in common organic solvents and stable when stored in air at room temperature.

In order to understand the reasons for the observed regioselectivity of the reaction leading to the formation of only one isomeric adduct **3**, as well as the unexpected absence of a cycloaddition product to phenylacetylene, we performed DFT calculations of the energy profile of the cycloaddition reaction of nitrile oxide **2a** to alkynes **7a,g,i**–**l** at the B3LYP-D3/6-311+G(d,p) level of theory with the SMD solvent model for DCM (for details of the calculations, see the Supplementary Materials) (Table 2).

**Scheme 3:**
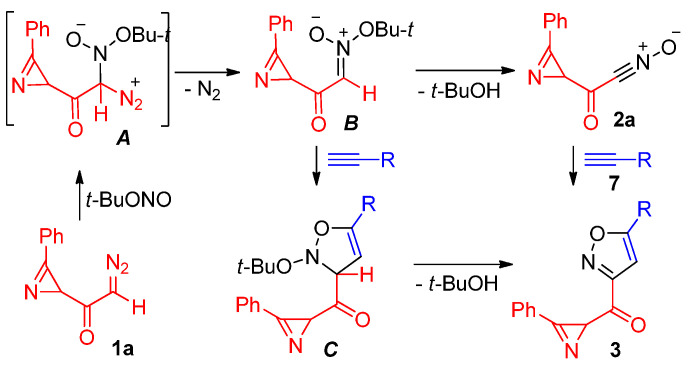
Possible mechanism for the formation of nitrile oxide.

From the calculation results, it follows that the transition state (TS) energy for the reaction leading to adduct **3** is at least 3.3 kcal/mol lower than the transition state energy for the reaction leading to regioisomeric adduct **3’**, which ensures the observed high regioselectivity of the reaction. The calculation results show that nitrile oxide **2a** should easily react at room temperature with all acetylenes **7a,g,i**–**l**, and with phenylacetylene **7k** most easily, which contradicts the experiment. This prompted us to turn to the mechanism of the formation of nitrile oxide and to conduct additional calculations and experiments. According to M. P. Doyle et al. [46], (2-diazoacetyl)derivatives N_2_CHC(O)Z (Z = OEt, NEt_2_, Ph) undergo nitrosyl addition with the electrophilic TBN to generate the diazonium ion intermediate of type ***A***, which loses dinitrogen and *t*-BuOH in a stepwise or concerted fashion to form nitrile oxide of type **2a** (Scheme 3).

According to DFT calculations (for details, see the Supplementary Materials), the attack of TBN on diazoacetylazirine **1a** in DCM is accompanied by the release of a nitrogen molecule and, through an energy barrier of 38.9 kcal/mol, leads to the formation of a relatively stable intermediate ***B***. In turn, the non-catalytic elimination of *t*-BuOH from intermediate ***B*** with the formation of nitrile oxide **2a** requires overcoming an energy barrier of 38.6 kcal/mol. Such high barriers should block the formation of adducts **3** at room temperature. This led us to the idea that the reaction mixture contains a certain catalyst that decreases the barrier of the nitrile oxide formation. We supposed that such a catalyst could be traces of HCl in DCM or FeCl_2_, which was used to obtain diazoacetylazirines **1** [47,48,49,50,51]. From the chemical properties of alkynes, it can be expected that phenylacetylene and DMAD should bind traces of HCl best of all the other acetylenic 2-π components studied. This may block the catalysis of the formation of nitrile oxide **2** and, thus, the formation of cycloadducts of **3v** and **3w**. H. Y. Yuan, Y. L. Zhao et al. reported [52] the preparation of isoxazoles by the [3+2] cycloaddition reaction of alkynes and nitrile oxides generated from (2-diazoacetyl)derivatives N_2_CHC(O)Z (Z = OR, Ar) and TBN under Cu(OAc)_2_ catalysis in the presence of DABCO in toluene at 130 °C. Meanwhile, X. Bao, X. Wan et al. [53] believe that the isoxazolines, products of formal intramolecular and intermolecular [3+2] cycloaddition of nitrile oxides to alkenes, are formed by the addition of type ***B*** nitronates, formed from 2-diazoacetates and TBN in DMF, to the C=C double bond, followed by the elimination of *t*-BuOH from the intermediate of type ***C***. However, the calculated energies for the interaction of TBN with (diazoacetyl)azirine **2a** calculated with DCM as a solvent (vide supra) change little when switching to DMF (38.1 and 39.0 kcal/mol, respectively). Furthermore, the calculated energetic parameters for the cycloaddition of nitronate ***B*** to alkynes **7a,g,i,k,l** in DCM show that, at room temperature, the reaction should be too slow, with the exception of DMAD (**7l**), and practically non-regioselective in the case of **7a** (Table 3).

**Scheme 4:**
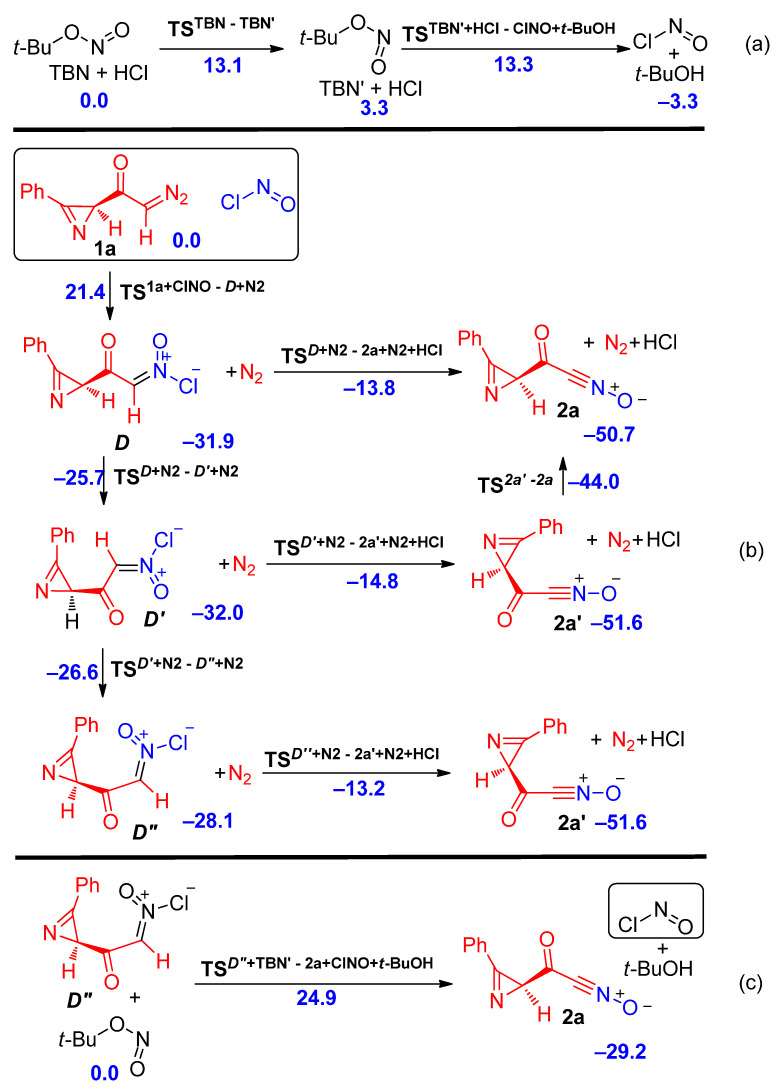
Relative Gibbs free energies for the nitrile oxide formation reactions under HCl catalysis (DFT B3LYP-D3/6-311+G(d,p) level with SMD model for DCM, in kcal/mol, 298 K).

It is known that in the presence of acids, TBN can form species that are more reactive than TBN itself [54], which can react more easily with the terminal diazo compound **1**, which has a nucleophilic character [55]. We assumed that such an electrophilic intermediate could be nitrosyl chloride (ClNO) generated by the reaction of TBN with HCl. Indeed, according to calculations, the reaction of TBN with HCl easily produces nitrosyl chloride and *t-*BuOH, even at room temperature (Scheme 4a). The calculations also demonstrated that ClNO, through a low energy barrier, yields *N*-oxoiminium salt (***D***/***D’***/***D”***), which can intramolecularly eliminate HCl to form nitrile oxide **2a** (Scheme 4b, energy profile of the reaction in the form of a diagram; see in the Supplementary Materials, Figure S3). Even easier formation of nitrile oxide **2a** from *N*-oxoiminium salt ***D”*** should occur under the reaction with TBN, in which ClNO is regenerated, continuing the catalytic cycle (Scheme 4c).

**Scheme 5:**
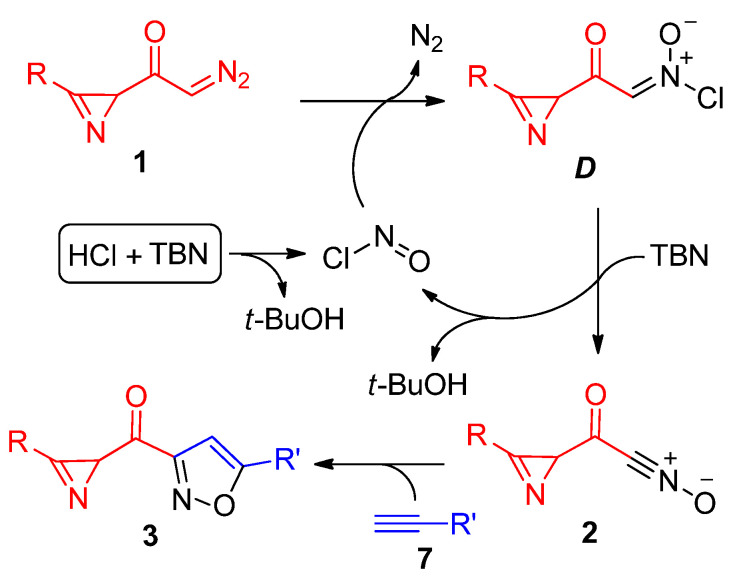
Plausible mechanism for the formation of nitrile oxide under acidic catalysis.

Based on the obtained results, one can imagine a plausible reaction mechanism, as shown in Scheme 5.

Guided by the obtained information, we started looking for a catalyst among Bronsted and Lewis acids in order to expand the scope of the three-component reaction between (diazoacetyl)azirines, TBN, and alkynes, in particular, by the involvement of aryl-substituted alkynes. The reaction of diazo compound **1q** with TBN and phenylacetylene (**7k**) was used as a test reaction to obtain cycloadduct **3x** (Table 4).

**Scheme 6:**
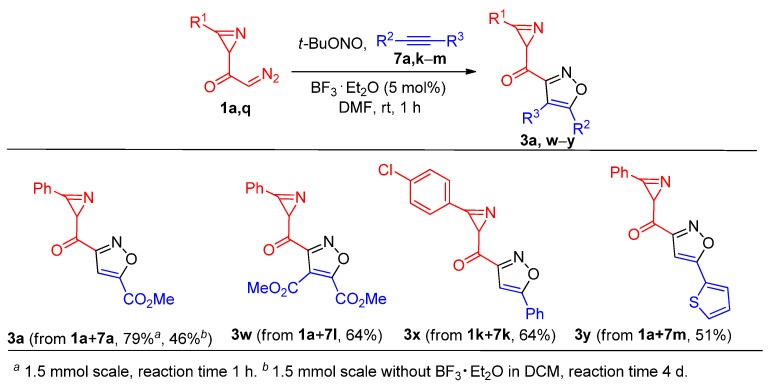
Synthesis of isoxazoles **3a,w,x,y** under **BF_3_·Et_2_O** catalysis.

To our satisfaction, the use of 10 mol% PTSA as a catalyst allowed us to obtain cycloadduct **3x** in DCM in only 3 h, albeit in a 21% yield (Table 4, entry 1). Lewis acid, BF_3_·Et_2_O, also catalyzed this reaction (Table 4, entry 2). Switching to more polar solvents increased the yield of adduct **3x**. The highest yield of the adduct was achieved using boron trifluoride etherate (5 mol%) as the catalyst in DMF. The reaction was completed after 1 h at room temperature to produce compound **3x** in a 64% yield. Using the conditions found, it was also possible to obtain cycloadducts with heterylacetylene **7m** and DMAD (Scheme 6). An additional confirmation of our assumption about the presence of trace amounts of acid catalyst (HCl), when carrying out the reaction in DCM, is the change in the time and yield of the reaction of azirine **1a** with methyl propiolate (**7a**) when scaling the reaction. Thus, when increasing the scale of the reaction from 0.27 to 1.5 mmol, the reaction time increased 4 times (from 1 d to 4 d), and the yield dropped from 91 to 46%, apparently due to the fact that a larger amount of propiolate **7a** is more effective in trapping HCl. At the same time, the 1.5 mmol scale reaction of azirine **1a** with methyl propiolate (**7a**) in the presence of BF_3_·Et_2_O (5 mol%) in DMF on a scale of 1.5 mmol produced adduct **3a** in 1 h with a yield of 79% (Scheme 4).

We then proceeded to search for options for expanding the azirine ring of the obtained cycloadducts in order to obtain a series of structurally diverse functionalized isoxazole-containing heterocyclic hybrids. Azirines containing substituents with functional groups have proven to be effective synthetic building blocks for the synthesis of a wide range of heterocyclic compounds [47,48,49,50,51,56,57,58,59,60,61,62,63]. In particular, 2-carbonyl-2*H*-azirines can be rearranged to the corresponding isoxazoles if the latter are more thermodynamically stable. This is usually the case when the isoxazole does not contain OR, NRR’, SR, or halogen substituents at C5 [63]. We performed a DFT calculation of the free energy for some 3-((2*H*-azirin-2-yl)carbonyl))isoxazoles **3**/biisoxazoles **4** pairs at the B3LYP-D3/6-311+G(d,p) level of theory with SMD solvent model for acetonitrile (for details of the calculations, see the Supplementary Materials). Biisoxazoles **4** are more thermodynamically stable than the corresponding azirinylcarbonylisoxazoles **3** by 4–5 kcal/mol: **4a/3a**—4.6; **4c/3c**—4.4; **4d/3d**—4.4; **4g/3g**—4.0; **4h/3h**—4.7; **4i/3i**—4.9 kcal/mol. This means that the isomerization of compounds **3** to biisoxazoles **4** should be a thermodynamically favorable process that can occur upon photoirradiation or heating with or without a catalyst [49,61,62,63]. The photochemical rearrangement of 2-benzoyl-3-phenyl-2*H*-azirine to 3,5-diphenylisoxazol [64] occurs via a triplet vinylnitrene intermediate [65]. 2,2-Disubstituted 2*H*-azirines containing a 2-acetyl-moiety rearrange to isoxazoles upon refluxing in xylene [66]. The addition of a Fe(II) catalyst allows the reaction temperature to be lowered [49,67,68,69,70]. We began searching for optimal conditions for the isomerization of azirines **3** to biisoxazoles **4**, using azirine **3i** as a model compound (Table 5).

**Scheme 7:**
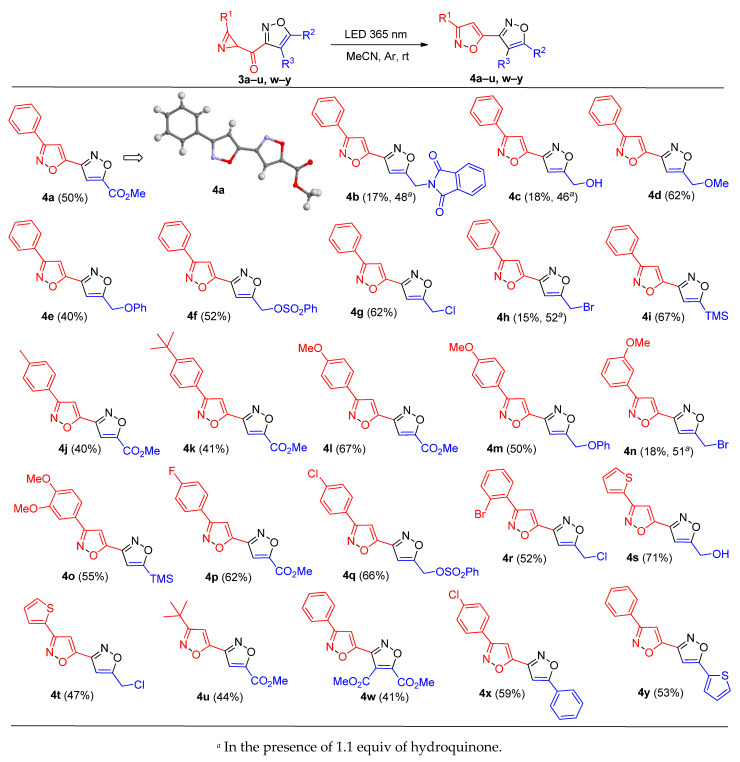
Synthesis of biisoxazoles **4**.

The most suitable conditions for the isomerization of azirine **3i** to biisoxazole **4i** were found to be irradiation of a solution of azirine **3i** in acetonitrile with LED 365 nm light for 2 d, which produced the product in a 67% yield (Table 5, entry 3). Biisoxazole **4i** was also obtained in a 56% yield by heating a solution of azirine **3i** in mesitylene at 200 °C for 1.5 h (Table 5, entry 9). When irradiation (Table 5, entry 3) was used to initiate isomerization from azirines **3a** to **u**, biisoxazoles **4a,d**–**g, i**–**m, o**–**u, x**–**z** were obtained in yields of 40–71%, whereas the yield of biisoxazoles **4b**,**c**,**h** and **n** (Scheme 3) was only 15–18%. We hypothesized that the free radical reaction initiated by the irradiation of azirines **3b**,**c**,**h** and **n** or biisoxazoles **4b**,**c**,**h** and **n** might be responsible for the observed decrease in product yield. To avoid the influence of free radicals on the yield, the reactions of azirines **3b**,**c**,**h** and **n** were carried out in the presence of the 1.1 equiv of hydroquinone as a free radical scavenger. This made it possible to increase the yield of target products by 28–37% (Scheme 7).

Compounds **4** were characterized by ^1^H and ^13^C NMR and HRMS. ^1^H NMR spectra of compounds **4** contain characteristic signals of protons at 4-C isoxazole fragments in the region of 6.5–7.5 ppm (singlets). ^13^C NMR spectra contain characteristic signals of carbon atom nuclei of 4-C isoxazole fragments in the region of 105–115 ppm. The structure of compound **4a** was confirmed by X-ray structural analysis (CCDC 2455843). Substances **4a**–**u, x**–**z** are colorless crystals; partially soluble in ethyl acetate, dichloromethane, and chloroform; and stable when stored in air at room temperature.

**Scheme 8:**
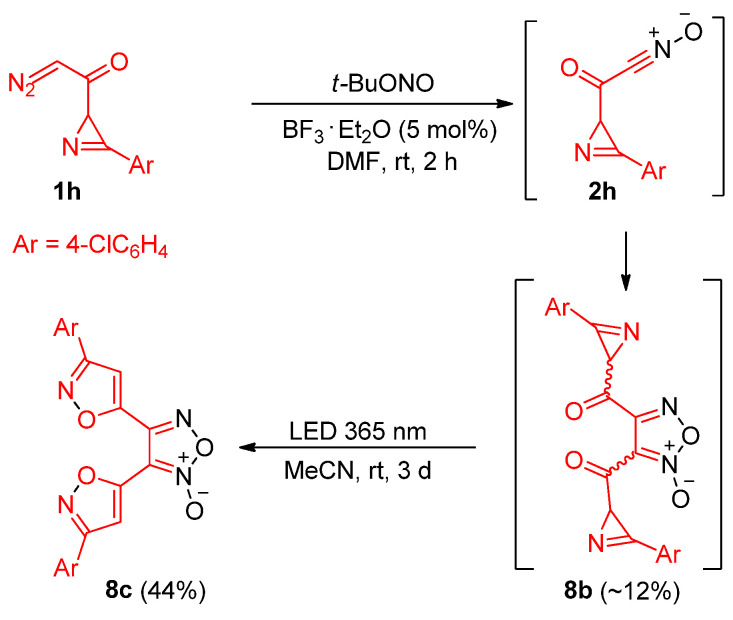
Synthesis of furoxan **8c**.

As mentioned above, the formation of furoxan dimer **8** was not observed in experiments that were carried out without acidic additive, including the reaction of **1** with TBN in the absence of a 2-π component. Apparently, the formation of furoxan **8**, the dimer of nitrile oxide **2**, did not occur due to the low concentration of nitrile oxide. However, when (diazoacetyl)azirine **1h** was reacted with TBN in the presence of boron trifluoride, which promoted the formation of nitrile oxide **2h**, furoxan **8b** was formed as a mixture of diastereomers (HRMS [**8b**+H] = 441.0148). The latter was converted without further purification into bis(isoxazolyl)-substituted furoxan **8c** by LED photoisomerization of the azirinylcarbonyl fragments of the mixture of compounds **8b** (Scheme 8).

**Scheme 9:**
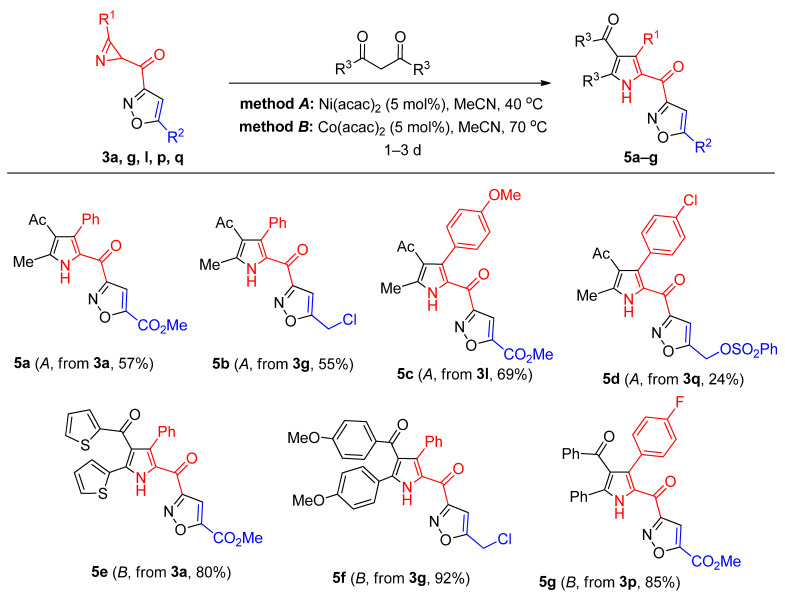
Synthesis of isoxazolyl(pyrrolyl)ketones **5**.

To obtain pyrrole–isoxazole hybrids, a catalytic reaction of compound **3** with 1,3-dicarbonyl compounds was chosen, allowing for the expansion of the azirine ring to the pyrrole ring [60,61,62,71,72,73,74]. Reactions of azirines **3a,g,l,q** with acetylacetone were carried out using Ni(acac)_2_ as a catalyst [72,73,74]. As a result, pyrroles **5a**–**d** were prepared in a 24–69% yield under mild conditions (Scheme 9). The significant decrease in yield in the case of the compound containing the OSO_2_Ph functional group (compound **5d**) is obviously due to the reactivity of this group under the reaction conditions. The use of this catalyst for the reaction of azirines **3** with 1,3-dicarbonyl compounds containing aryl or hetaryl substituents was unsuccessful, but with a Co(acac)_3_ catalyst, the azirine ring expansion occurred at 70 °C to form pyrroles **5e–g** in an 80–90% yield (Scheme 9).

Compounds **5** were characterized by ^1^H and ^13^C NMR and HRMS. ^1^H NMR spectra of compounds **5** contain a characteristic signal of an NH proton at the pyrrole fragment in the region of 10.5–11.5 ppm (br. singlet) and a signal of a proton at the 4-C isoxazole fragment in the region of 6.5–7.5 ppm (singlet). ^13^C NMR spectra contain a characteristic signal of the carbon atom nuclear of the 4-C isoxazole fragment in the region of 105–115 ppm. The IR spectrum of compound **5a** contains characteristic absorption bands of C=O stretching of carbonyl and ester groups near 1640 and 1740 cm^−1^, respectively, and an absorption band of N-H stretching near 3330 cm^−1^.

**Scheme 10:**
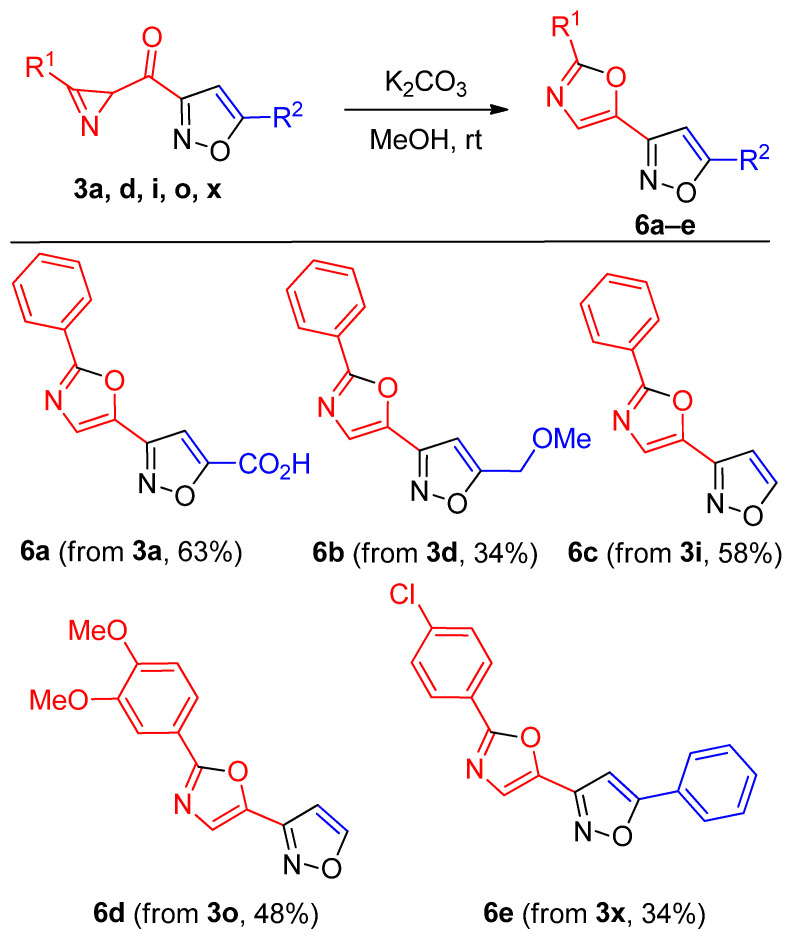
Synthesis of oxazolylisoxazoles **6**.

Our next goal was to transform 3-(azirinylcarbonyl)isoxazoles **3** into oxazol–isoxazole hybrids **6**. 2-Acyl-2*H*-azirines can be isomerized into isoxazoles under photoirradiation [56,57,58,59,60,61,62,64,65,75]. However, this process, which proceeds via the formation of nitrile ylides, is rarely used in synthetic practice, since it is complicated by the formation of isoxazoles and other compounds. Of greater synthetic importance is the isomerization of 3-alkyl-2-acylazirines to oxazoles under basic conditions [75,76,77], which proceeds via a deprotonation-initiated mechanism [77] or nucleophilic addition to the azirine C=N bond [77,78]. At the same time, only two examples of the transformation of 3-aryl-substituted azirines (2-benzoyl- and 2-benzoyl-2-methyl-3-phenyl-2*H*-azirines) into the corresponding oxazoles in methanol in the presence of potassium or sodium carbonates are known [75,77]. After some optimization, we found that 2-(isoxazole-3-ylcarbonyl)-3-arylazirines **3** can be easily isomerized to 3-(oxazol-5-yl)isoxazoles **6** in methanol in the presence of excess potassium carbonate at room temperature in a 34–63% yield (Scheme 10). Thus, azirines **3a,i,d,o,x** were transformed to 3-(oxazol-5-yl)isoxazoles **6a**–**e**, where, due to alkaline conditions, compound **6a** contains a carboxyl group instead of the ester group as a result of hydrolysis, and from TMS-substituted isoxazoles **3i,o**, desilylated oxazolylisoxazoles **6c,e** were obtained. The letter reactions illustrate the applicability of the “azirinylcarbonyl nitrile oxide strategy” for the synthesis of isoxazole-containing heterocyclic hybrids with an unsubstituted isoxazole moiety

Compounds **6** were characterized by ^1^H and ^13^C NMR and HRMS. ^1^H NMR spectra of compounds **6** contain a characteristic signal of a proton at the 4-C isoxazole fragment in the region of 6.5–7.5 ppm (singlet) and a signal of a proton at the 4-C oxazole fragment in the region of 7.5–8.0 ppm (singlet). ^13^C NMR spectra contain a characteristic signal of the carbon atom nuclear of the 4-C isoxazole fragment in the region of 100–115 ppm and a signal of the carbon atom nuclear of the 4-C oxazole fragment in the region of 120–125 ppm.

## 3. Materials and Methods

### 3.1. General Instrumentation

Melting points were determined on a melting point apparatus. ^1^H (400 MHz) and ^13^C (100 MHz) spectra were recorded on a Bruker AVANCE 400 spectrometer (Billerica, MA, USA) in CDCl_3_ or DMSO-*d*_6_. Chemical shifts (δ) are reported in parts per million downfield from tetramethylsilane (TMS, δ = 0.00). ^1^H NMR spectra were calibrated according to the residual peak of H-analogues of CDCl_3_ (7.26 ppm) and DMSO-*d*_6_ (2.50 ppm). For all new compounds, ^13^C{^1^H} and ^13^C DEPT-135 spectra were recorded and calibrated according to the peak of CDCl_3_ (77.00 ppm) and DMSO-*d*_6_ (39.51 ppm). Electrospray ionization (ESI) mass spectra were recorded on a Bruker MaXis mass spectrometer, HRMS-ESI-QTOF. Infrared (IR) data were recorded in a scan range from 400 to 4000 cm ^−1^ on a SHIMADZU IRAffinity-1 FT-IR spectrometer. The crystal structures of **3g** and **4a** were determined by the means of single-crystal XRD analysis using Agilent Technologies (Oxford Diffraction) “Supernova” and Rigaku Oxford Diffraction “XtaLAB Synergy” diffractometers. The crystals were measured at a temperature of 100 K, using monochromated Cu Kα radiation. Crystallographic data for the structures **3g** (CCDC 2455839) and **4a** (CCDC 2455843) have been deposited with the Cambridge Crystallographic Data Centre. Thin-layer chromatography (TLC) was conducted on aluminum sheets with 0.2 mm silica gel with a fluorescent indicator. All solvents were distilled and dried prior to use. Physical and spectral data of 2-(diazoacetyl)-2*H*-azirines **1a,b,d,g,h,j,k** [47]; **1c,e,i** [51]; and **1f** [74], prepared according to the published procedures, were in agreement with previously reported values.

### 3.2. General Experimental Procedures

#### 3.2.1. General Procedure A (GP-A) for the Preparation of Isoxazoles **3**

A mixture of azirine **1** (1 mmol), *tert*-butylnitrite (5 mmol), and acetylene **7** (5 mmol) in DCM was stirred at room temperature for 1–7 days, with monitoring by TLC. The solvent was evaporated, and the residue was purified by column chromatography on silica gel, using light petroleum/ethyl acetate as an eluent, to yield pure compound **3**.

#### 3.2.2. General Procedure B (GP-B) for the Preparation of Isoxazoles **3**

A mixture of azirine **1** (1 mmol), *tert*-butylnitrite (5 mmol), and acetylene **7** (5 mmol) in DMF was stirred at rt for 20 min, then boron trifluoride etherate (0.05 mmol) was added. The reaction mixture was stirred at rt for 1 h (monitored by TLC). The reaction mixture was diluted with water and extracted with ethyl acetate. The organic phase was washed with water, dried over Na_2_SO_4_, and the solvent was evaporated, and the residue was purified by column chromatography on silica gel, using light petroleum/ethyl acetate as an eluent, to yield pure compound **3**.

#### 3.2.3. General Procedure C (GP-C) for the Preparation of Biisoxazoles **4**

A solution of azirine **3** (1 mmol) in acetonitrile was flushed with argon and irradiated using LED 365 at room temperature for 1–3 days, with monitoring by TLC. The solvent was evaporated, and the residue was purified by column chromatography on silica gel, using light petroleum/ethyl acetate as an eluent, to yield pure compound **4**.

#### 3.2.4. General Procedure D (GP-D) for the Preparation of Biisoxazoles **4**

A mixture of azirine **3** (1 mmol) and hydroquinone (1.1 mmol) in acetonitrile was flushed with argon and irradiated using LED 365 nm at room temperature for 1–3 days, with monitoring by TLC. The solvent was evaporated, and the residue was purified by column chromatography on silica gel, using light petroleum/ethyl acetate as an eluent, to yield pure compound **4**.

#### 3.2.5. General Procedure E (GP-E) for the Preparation of Pyrroles **5**

A mixture of azirine **3** (1 mmol), 1,3-diketone (3 mmol), and Ni(acac)_2_ or Co(acac)_3_ (5 mol%) in acetonitrile was stirred at 40 °C or 70 °C for 1–3 days, with monitoring by TLC. The solvent was evaporated, and the residue was purified by column chromatography on silica gel, using light petroleum/ethyl acetate as an eluent, to yield pure compound **5.**

#### 3.2.6. General Procedure F (GP-E) for the Preparation of 3-(Oxazol-5-yl)isoxazoles **6**

A mixture of azirine **3** (1 mmol) and K_2_CO_3_ (4 mmol) in methanol was stirred at rt for 1–2 days (monitored by TLC). The reaction mixture was diluted with water and extracted with ethyl acetate. The organic phase was washed with water and dried over Na_2_SO_4_. The solvent was evaporated, and the residue was purified by column chromatography on silica gel, using light petroleum/ethyl acetate as an eluent, to yield pure compound **6.**

#### 3.2.7. Specific Procedures and Characterization

*Methyl 3-(3-phenyl-2H-azirine-2-carbonyl)isoxazole-5-carboxylate* (**3a**). Compound **3a** was prepared following the general procedure GP-A from 2-diazo-1-(3-phenyl-2*H*-azirin-2-yl)ethan-1-one **1a** (50 mg, 0.27 mmol), *tert*-butyl nitrite (175 μL, 1.35 mmol), and methyl propiolate **7a** (114 μL, 1.35 mmol) in DCM (5 mL) to produce pure product in 69 mg (91% yield), after column chromatography on silica (light petroleum/ethyl acetate, 4:1, (*v*/*v*)).

Compound **3a** was also prepared following the general procedure GP-B from 2-diazo-1-(3-phenyl-2*H*-azirin-2-yl)ethan-1-one **1a** (300 mg, 1.62 mmol), *tert*-butyl nitrite (1.02 mL, 8.1 mmol), methyl propiolate **7a** (811 μL, 8.1 mmol), and boron trifluoride etherate (10 μL, 81 μmol) in DMF (10 mL) to produce pure product in 348 mg (79% yield), after column chromatography on silica gel (eluent: light petroleum/ethyl acetate, 2:1, (*v*/*v*)) as a yellow solid: mp 85–87 °C (light petroleum/ethyl acetate); ^1^H NMR (CDCl_3_, 400 MHz): *δ* 7.91–7.88 (m, 2H), 7.68–7.64 (m, 1H), 7.60–7.56 (m, 2H), 7.32 (s, 1H), 4.05 (s, 1H), 4.01 (s, 3H); ^13^C{^1^H} NMR (CDCl_3_, 100 MHz): *δ* 190.5 (C), 162.1 (C), 161.4 (C), 156.6 (C), 156.0 (C), 134.2 (CH), 130.8 (CH), 129.4 (CH), 121.6 (C), 107.9 (C), 53.2 (CH), 34.5 (CH_3_); IR (KBr, cm^–1^): 3134, 2956, 1741, 1680, 1586, 1450, 1313, 1229, 1130, 998, 979, 931, 756, 687, 554; HRMS (ESI) *m*/*z* [M + H]^+^ calcd for C_14_H_11_N_2_O_4_^+^ 271.0713, found 271.0711.

*2-((3-(3-Phenyl-2H-azirine-2-carbonyl)isoxazol-5-yl)methyl)isoindoline-1,3-dione* (**3b**). Compound **3b** was prepared following the general procedure GP-A from 2-diazo-1-(3-(4-fluorophenyl)-2*H*-azirin-2-yl)ethan-1-one **1a** (50 mg, 0.27 mmol), *tert*-butyl nitrite (175 μL, 1.35 mmol), and 2-(prop-2-yn-1-yl)isoindoline-1,3-dione **7b** (250 mg, 1.35 mmol) in DCM (7 mL) to produce pure product in 63 mg (63% yield), after column chromatography on silica gel (eluent: light petroleum/ethyl acetate, 4:1, (*v*/*v*)) as a yellow solid: mp 93–94 °C (light petroleum/ethyl acetate); ^1^H NMR (CDCl_3_, 400 MHz): *δ* 7.92–7.90 (m, 2H), 7.87–7.85 (m, 2H), 7.78–7.76 (m, 2H), 7.65–7.61 (m, 1H), 7.57–7.53 (m, 2H), 6.67 (s, 1H), 5.07 (s, 2H), 3.99 (s, 1H); ^13^C{^1^H} NMR (CDCl_3_, 100 MHz): *δ* 191.1 (C), 168.2 (C), 167.0 (C), 162.0 (C), 156.2 (C), 134.5 (CH), 134.0 (CH), 131.7 (C), 130.8 (CH), 129.4 (CH), 123.8 (CH), 121.7 (C), 101.9 (CH), 34.4 (CH), 33.0 (CH_2_); HRMS (ESI) *m*/*z* [M + H]^+^ calcd for C_21_H_14_N_3_O_4_^+^ 372.0979, found 372.0971.

*(5-(Hydroxymethyl)isoxazol-3-yl)(3-phenyl-2H-azirin-2-yl)methanone* (**3c**). Compound **3c** was prepared following the general procedure GP-A from 2-diazo-1-(3-(4-fluorophenyl)-2*H*-azirin-2-yl)ethan-1-one **1a** (50 mg, 0.27 mmol), *tert*-butyl nitrite (175 μL, 1.35 mmol), and prop-2-yn-1-ol **7c** (80 μL, 1.35 mmol) in DCM (5 mL) to produce pure product in 33 mg (51% yield), after column chromatography on silica gel (eluent: light petroleum/ethyl acetate, 2:1, (*v*/*v*)) as a brown oil; ^1^H NMR (CDCl_3_, 400 MHz): *δ* 7.90–7.88 (m, 2H), 7.67–7.63 (m, 1H), 7.59–7.55 (m, 2H), 6.69 (s, 1H), 4.87–4.86 (m, 2H), 4.03 (s, 1H), 2.11 (br. s, 1H); ^13^C{^1^H} NMR (CDCl_3_, 100 MHz): *δ* 191.5 (C), 173.1 (C), 161.8 (C), 156.3 (C), 134.1 (CH), 130.8 (CH), 129.4 (CH), 121.7 (C), 100.7 (CH), 56.3 (CH_2_), 34.4 (CH); HRMS (ESI) *m*/*z* [M + H]^+^ calcd for C_13_H_11_N_2_O_3_^+^ 243.0764, found 243.0760.

*(5-(Methoxymethyl)isoxazol-3-yl)(3-phenyl-2H-azirin-2-yl)methanone* (**3d**). Compound **3d** was prepared following the general procedure GP-A from 2-diazo-1-(3-(4-fluorophenyl)-2*H*-azirin-2-yl)ethan-1-one **1a** (200 mg, 1.08 mmol), *tert*-butyl nitrite (465 μL, 5.4 mmol), and 3-methoxyprop-1-yne (560 mg, 5.4 mmol) in DCM (10 mL) to produce pure product in 164 mg (64% yield), after column chromatography on silica gel (eluent: light petroleum/ethyl acetate, 4:1, (*v*/*v*)) as a brown oil; ^1^H NMR (CDCl_3_, 400 MHz): *δ* 7.91–7.88 (m, 2H), 7.67–7.62 (m, 1H), 7.59–7.55 (m, 2H), 6.70 (s, 1H), 4.62 (s, 2H), 4.04 (s, 1H), 3.46 (s, 3H); ^13^C{^1^H} NMR (CDCl_3_, 100 MHz): *δ* 191.3 (C), 170.8 (C), 161.9 (C), 156.3 (C), 134.1 (CH), 130.8 (CH), 129.4 (CH), 121.8 (C), 101.8 (CH), 65.0 (CH_2_), 59.0 (CH), 34.3 (CH_3_); HRMS (ESI) *m*/*z* [M + H]^+^ calcd for C_14_H_13_N_2_O_3_^+^ 257.0921, found 257.0918.

*(5-(Phenoxymethyl)isoxazol-3-yl)(3-phenyl-2H-azirin-2-yl)methanone* (**3e**). Compound **3e** was prepared following the general procedure GP-A from 2-diazo-1-(3-(4-fluorophenyl)-2*H*-azirin-2-yl)ethan-1-one **1a** (150 mg, 0.81 mmol), *tert*-butyl nitrite (530 μL, 4.05 mmol), and (prop-2-yn-1-yloxy)benzene **7e** (510 μL, 4.05 mmol) in DCM (10 mL) to produce pure product in 258 mg (60% yield), after column chromatography on silica gel (eluent: light petroleum/ethyl acetate, 4:1, (*v*/*v*)) as a colorless solid: mp 70–72 °C (light petroleum/ethyl acetate); ^1^H NMR (CDCl_3_, 400 MHz): *δ* 7.90–7.88 (m, 2H), 7.67–7.63 (m, 1H), 7.59–7.55 (m, 2H), 7.35–7.31 (m, 2H), 7.05–7.01 (m, 1H), 6.98–6.96 (m, 2H), 6.78 (s, 1H), 5.24 (s, 2H), 4.04 (s, 1H); ^13^C{^1^H} NMR (CDCl_3_, 100 MHz): *δ* 191.2 (C), 169.7 (C), 161.9 (C), 157.6 (C), 156.2 (C), 134.1 (CH), 130.8 (CH), 129.8 (CH), 129.4 CH), 122.1 (CH), 121.8 (C), 114.8 (CH), 102.2 (CH), 61.0 (CH_2_), 34.5 (CH); HRMS (ESI) *m*/*z* [M + H]^+^ calcd for C_19_H_15_N_2_O_3_^+^ 319.1077, found 319.1079.

*(3-(3-Phenyl-2H-azirine-2-carbonyl)isoxazol-5-yl)methyl benzenesulfonate* (**3f**). Compound **3f** was prepared following the general procedure GP-A from 2-diazo-1-(3-phenyl-2*H*-azirin-2-yl)ethan-1-one **1a** (50 mg, 0.27 mmol), *tert*-butyl nitrite (175 μL, 1.35 mmol), and prop-2-yn-1-yl benzenesulfonate **7f** (215 μL, 1.35) in DCM (5 mL) to produce pure product in 72 mg (70% yield), after column chromatography on silica gel (eluent: light petroleum/ethyl acetate, 4:1, (*v*/*v*)) as a brown oil; ^1^H NMR (CDCl_3_, 400 MHz): *δ* 7.95–7.92 (m, 2H), 7.88–7.86 (m, 2H), 7.72–7.63 (m, 2H), 7.60–7.55 (m, 4H), 6.68 (s, 1H), 5.24 (s, 2H), 3.96 (m, 1H); ^13^C{^1^H} NMR (CDCl_3_, 100 MHz): *δ* 190.7 (C), 165.9 (C), 161.8 (C), 156.1 (C), 135.5 (C), 134.4 (CH), 134.2 (CH), 130.8 (CH), 129.5 (CH), 129.4 (CH), 128.0 (CH), 121.6 (C), 103.9 (CH), 60.6 (CH_2_), 34.4 (CH); HRMS (ESI) *m*/*z* [M + H]^+^ calcd for C_19_H_15_N_2_O_5_S^+^ 383.0696, found 383.0697.

*(5-(Chloromethyl)isoxazol-3-yl)(3-phenyl-2H-azirin-2-yl)methanoneole* (**3g**). Compound **3g** was prepared following the general procedure GP-A from 2-diazo-1-(3-phenyl-2*H*-azirin-2-yl)ethan-1-one **1a** (200 mg, 1.08 mmol), *tert*-butyl nitrite (700 μL, 5.4 mmol), and 3-chloroprop-1-yne **7g** (570 μL, 5.4 mmol) in DCM (10 mL) to produce pure product in 162 mg (57% yield), after column chromatography on silica gel (eluent: light petroleum/ethyl acetate, 4:1, (*v*/*v*)) as a light-yellow solid: mp 92–94 °C (light petroleum/ethyl acetate); ^1^H NMR (CDCl_3_, 400 MHz): *δ* 7.91–7.88 (m, 2H), 7.68–7.63 (m, 1H), 7.59–7.55 (m, 2H), 6.76 (s, 1H), 4.69 (s, 2H), 4.03 (s, 1H); ^13^C{^1^H} NMR (CDCl_3_, 100 MHz): *δ* 191.0 (C), 169.1 (C), 162.0 (C), 156.2 (C), 134.1 (CH), 130.8 (CH), 129.4 (CH), 121.7 (C), 102.6 (CH), 34.4 (CH), 34.0 (CH_2_); HRMS (ESI) *m*/*z* [M + H]^+^ calcd for C_13_H_10_ClN_2_O_2_^+^ 261.0424, found 261.0425.

*(5-(Bromomethyl)isoxazol-3-yl)(3-phenyl-2H-azirin-2-yl)methanone* (**3h**). Compound **3h** was prepared following the general procedure GP-A from 2-diazo-1-(3-phenyl-2*H*-azirin-2-yl)ethan-1-one **1a** (300 mg, 1.62 mmol), *tert*-butyl nitrite (1050 μL, 8.1 mmol), and 3-bromoprop-1-yne **7h** (900 μL, 8.1 mmol) in DCM (10 mL) to produce pure product in 311 mg (63% yield), after column chromatography on silica gel (eluent: light petroleum/ethyl acetate, 4:1, (*v*/*v*)) as a light-red–brown solid: mp 73–74 °C (light petroleum/ethyl acetate); ^1^H NMR (CDCl_3_, 400 MHz): *δ* 7.90–7.88 (m, 2H), 7.67–7.63 (m, 1H), 7.59–7.55 (m, 2H), 6.75 (s, 1H), 4.53 (s, 2H), 4.02 (s, 1H); ^13^C{^1^H} NMR (CDCl_3_, 100 MHz): *δ* 191.0 (C), 169.1 (C), 162.1 (C), 156.2 (C), 134.1 (CH), 130.8 (CH), 129.4 (CH), 121.7 (C), 102.6 (CH), 34.4 (CH), 17.8 (CH_2_); HRMS (ESI) *m*/*z* [M + H]^+^ calcd for C_13_H_10_BrN_2_O_2_^+^ 304.9927, found 304.9920.

*(3-Phenyl-2H-azirin-2-yl)(5-(trimethylsilyl)isoxazol-3-yl)methanone* (**3i**). Compound **3i** was prepared following the general procedure GP-A from 2-diazo-1-(3-phenyl-2*H*-azirin-2-yl)ethan-1-one **1a** (300 mg, 1.62 mmol), *tert*-butyl nitrite (1050 μL, 8.1 mmol), and ethynyltrimethylsilane **7i** (1150 μL, 8.1) in DCM (10 mL) to produce pure product in 370 mg (80% yield), after column chromatography on silica gel (eluent: light petroleum/ethyl acetate, 4:1, (*v*/*v*)) as a yellow–green solid: mp 47–48 °C (light petroleum/ethyl acetate); ^1^H NMR (CDCl_3_, 400 MHz): *δ* 7.90–7.88 (m, 2H), 7.66–7.62 (m, 1H), 7.58–7.54 (m, 2H), 6.88 (s, 1H), 4.06 (s, 1H), 0.39 (s, 9H); ^13^C{^1^H} NMR (CDCl_3_, 100 MHz): *δ* 191.8 (C), 180.2 (C), 160.4 (C), 156.5 (C), 134.0 (CH), 130.8 (CH), 129.4 (CH), 122.0 (C), 111.3 (CH), 34.9 (CH), –1.99 (CH_3_); HRMS (ESI) *m*/*z* [M + H]^+^ calcd for C_15_H_17_N_2_O_2_Si^+^ 285.1054, found 285.1050.

*Methyl 3-(3-(p-tolyl)-2H-azirine-2-carbonyl)isoxazole-5-carboxylate* (**3j**). Compound **3j** was prepared following the general procedure GP-A from 2-diazo-1-(3-(*p*-tolyl)-2*H*-azirin-2-yl)ethan-1-one **1b** (150 mg, 0.75 mmol), *tert*-butyl nitrite (490 μL, 3.75 mmol), and methyl propiolate **7a** (360 μL, 3.75 mmol) in DCM (10 mL) to produce pure product in 120 mg (57% yield), after column chromatography on silica gel (eluent: light petroleum/ethyl acetate, 4:1, (*v*/*v*)) as a light-red–brown solid: mp 111–113 °C (light petroleum/ethyl acetate); ^1^H NMR (CDCl_3_, 400 MHz): *δ* 7.77 (d, 2H, *J* = 8.1 Hz), 7.38 (d, 2H, *J* = 8.1 Hz), 7.32 (s, 1H), 4.01 (s, 4H), 2.46 (s, 3H); ^13^C{^1^H} NMR (CDCl_3_, 100 MHz): *δ* 190.7 (C), 162.1 (C), 161.4 (C), 156.6 (C), 155.5 (C), 145.4 (C), 130.9 (CH), 130.2 (CH), 118.7 (C), 108.0 (CH), 53.2 (CH_3_), 34.4 (CH), 22.0 (CH_3_); HRMS (ESI) *m*/*z* [M + H]^+^ calcd for C_15_H_13_N_2_O_4_^+^ 285.0870, found 285.0867.

*Methyl 3-(3-(4-(tert-butyl)phenyl)-2H-azirine-2-carbonyl)isoxazole-5-carboxylate* (**3k**). Compound **3k** was prepared following the general procedure GP-A from 1-(3-(4-(*tert*-butyl)phenyl)-2*H*-azirin-2-yl)-2-diazoethan-1-one **1c** (50 mg, 0.21 mmol), *tert*-butyl nitrite (99 μL, 1.05 mmol), and methyl propiolate **7a** (135 μL, 1.05 mmol) in DCM (5 mL) to produce pure product in 41 mg (60% yield), after column chromatography on silica gel (eluent: light petroleum/ethyl acetate, 4:1, (*v*/*v*)) as a light-brown oil; ^1^H NMR (CDCl_3_, 400 MHz): *δ* 7.82 (d, 2H, *J* = 8.4 Hz), 7.59 (d, 2H, *J* = 8.4 Hz), 7.31 (m, 1H), 4.01 (s, 4H), 1.36 (s, 9H); ^13^C{^1^H} NMR (CDCl_3_, 100 MHz): *δ* 190.7 (C), 162.1 (C), 161.3 (C), 158.4 (C), 156.6 (C), 155.5 (C), 130.8 (CH), 126.5 (CH), 118.6 (C), 108.8 (CH), 53.2 (CH), 35.4 (C), 34.4 (CH_3_), 31.0 (CH_3_); HRMS (ESI) *m*/*z* [M + H]^+^ calcd for C_18_H_19_N_2_O_4_^+^ 327.1339, found 327.1343.

*Methyl 3-(3-(4-methoxyphenyl)-2H-azirine-2-carbonyl)isoxazole-5-carboxylate* (**3l**). Compound **3l** was prepared following the general procedure GP-A from 2-diazo-1-(3-(4-methoxyphenyl)-2*H*-azirin-2-yl)ethan-1-one **1d** (300 mg, 1.4 mmol), *tert*-butyl nitrite (910 μL, 7 mmol), and methyl propiolate **7a** (665 μL, 7 mmol) in DCM (10 mL) to produce pure product in 366 mg (87% yield), after column chromatography on silica gel (eluent: light petroleum/ethyl acetate, 4:1 + 5% CHCl_3_, (*v*/*v*)) as a light-brown solid: mp 127–129 °C (light petroleum/ethyl acetate); ^1^H NMR (CDCl_3_, 400 MHz): *δ* 7.82 (d, 2H, *J* = 8.8 Hz), 7.31 (s, 1H), 7.06 (d, 2H, *J* = 8.8 Hz), 4.01 (s, 3H), 3.98 (s, 1H), 3.90 (s, 3H); ^13^C{^1^H} NMR (CDCl_3_, 100 MHz): *δ* 190.9 (C), 164.3 (C), 162.1 (C), 161.3 (C), 156.6 (C), 154.6 (C), 132.9 (CH), 115.0 (CH), 113.7 (C), 108.0 (CH), 55.7 (CH), 53.2 (CH_3_), 34.4 (CH_3_); HRMS (ESI) *m*/*z* [M + H]^+^ calcd for C_15_H_13_N_2_O_5_^+^ 301.0819, found 301.0823.

*(3-(4-Methoxyphenyl)-2H-azirin-2-yl)(5-(phenoxymethyl)isoxazol-3-yl)methanone* (**3m**). Compound **3m** was prepared following the general procedure GP-A from 2-diazo-1-(3-(4-methoxyphenyl)-2*H*-azirin-2-yl)ethan-1-one **1d** (200 mg, 0.93 mmol), *tert*-butyl nitrite (610 μL, 4.65 mmol), and (prop-2-yn-1-yloxy)benzene **7e** (580 μL, 4.65 mmol) in DCM (10 mL) to produce pure product in 220 mg (68% yield), after column chromatography on silica gel (eluent: light petroleum/ethyl acetate, 4:1 + 5% CHCl_3_, (*v*/*v*)) as a light-brown solid: mp 125–127 °C (light petroleum/ethyl acetate); ^1^H NMR (CDCl_3_, 400 MHz): *δ* 7.82 (d, 2H, *J* = 8.8 Hz), 7.34–7.30 (m, 2H), 7.05 (d, 2H, *J* = 8.8 Hz), 7.03–7.01 (m, 1H), 6.98–6.96 (m, 2H), 6.77 (s, 1H), 5.24 (s, 2H), 3.97 (s, 1H), 3.89 (s, 3H); ^13^C{^1^H} NMR (CDCl_3_, 100 MHz): *δ* 190.9 (C), 164.3 (C), 162.1 (C), 161.3 (C), 156.6 (C), 154.6 (C), 132.9 (CH), 129.7 (CH), 122.1 (CH), 115.0 (CH), 113.7 (CH), 108.0 (CH), 55.7 (CH), 53.2 (CH), 34.4 (CH_3_); HRMS (ESI) *m*/*z* [M + H]^+^ calcd for C_20_H_17_N_2_O_4_^+^ 349.1183, found 349.1187.

*(5-(Bromomethyl)isoxazol-3-yl)(3-(3-methoxyphenyl)-2H-azirin-2-yl)methanone* (**3n**). Compound **3n** was prepared following the general procedure GP-A from 2-diazo-1-(3-(3-methoxyphenyl)-2*H*-azirin-2-yl)ethan-1-one **1e** (200 mg, 0.93 mmol), *tert*-butyl nitrite (610 μL, 4.65 mmol), and 3-bromoprop-1-yne **7h** (413 μL, 4.65 mmol) in DCM (10 mL) to produce pure product in 177 mg (57% yield), after column chromatography on silica gel (eluent: light petroleum/ethyl acetate, 4:1, (*v*/*v*)) as a brown oil; ^1^H NMR (CDCl_3_, 400 MHz): *δ* 7.49–7.41 (m, 3H), 7.20–7.17 (m, 1H), 6.75 (s, 1H), 4.53 (s, 2H), 4.02 (s, 1H), 3.88 (s, 3H); ^13^C{^1^H} NMR (CDCl_3_, 100 MHz): *δ* 191.0 (C), 169.1 (C), 162.1 (C), 160.1 (C), 156.3 (C), 130.5 (CH), 123.4 (CH), 122.8 (C), 120.9 (CH), 114.5 (CH), 102.6 (CH), 55.6 (CH), 34.7 (CH_3_), 17.8 (CH_2_); HRMS (ESI) *m*/*z* [M + H]^+^ calcd for C_14_H_12_BrN_2_O_3_^+^ 335.0019, found 335.0026.

*(3-(3,4-Dimethoxyphenyl)-2H-azirin-2-yl)(5-(trimethylsilyl)isoxazol-3-yl)methanone* (**3o**). Compound **3o** was prepared following the general procedure GP-A from 2-diazo-1-(3-(3,4-dimethoxyphenyl)-2*H*-azirin-2-yl)ethan-1-one **1f** (200 mg, 0.82 mmol), *tert*-butyl nitrite (520 μL, 4.1 mmol), and ethynyltrimethylsilane **7i** (520 μL, 4.1 mmol) in DCM (10 mL) to produce pure product in 237 mg (84% yield), after column chromatography on silica gel (eluent: light petroleum/ethyl acetate, 4:1, (*v*/*v*)) as a light-yellow solid: mp 87–88 °C (light petroleum/ethyl acetate); ^1^H NMR (CDCl_3_, 400 MHz): *δ* 7.44 (d, 1H, *J* = 1.9 Hz), 7.41 (dd, 1H, *J* = 8.2, 1.9 Hz), 6.98 (d, 1H, *J* = 8.2 Hz), 6.88 (s, 1H), 4.02 (s, 1H), 3.96 (s, 3H), 3.95 (s, 3H), 0.38 (s, 9H); ^13^C{^1^H} NMR (CDCl_3_, 100 MHz): *δ* 192.2 (C), 180.2 (C), 160.5 (C), 155.6 (C), 153.9 (C), 149.7 (C), 125.8 (CH), 114.2 (C), 111.8 (CH), 111.3 (CH), 111.1 (CH), 77.2 (CH), 56.2 (CH_3_), 35.1 (CH_3_), –1.97 (CH_3_); HRMS (ESI) *m*/*z* [M + H]^+^ calcd for C_17_H_20_N_2_O_4_Si^+^ 345.1265, found 345.1262.

*Methyl 3-(3-(4-fluorophenyl)-2H-azirine-2-carbonyl)isoxazole-5-carboxylate* (**3p**). Compound **3p** was prepared following the general procedure GP-A from 2-diazo-1-(3-(4-fluorophenyl)-2*H*-azirin-2-yl)ethan-1-one **1g** (50 mg, 0.25 mmol), *tert*-butyl nitrite (160 μL, 1.25 mmol), and methyl propiolate **7a** (120 μL, 1.25 mmol) in DCM (5 mL) to produce pure product in 48 mg (68% yield), after column chromatography on silica gel (eluent: light petroleum/ethyl acetate, 4:1, (*v*/*v*)) as a light-yellow solid: mp 112–114 °C (light petroleum/ethyl acetate); ^1^H NMR (CDCl_3_, 400 MHz): *δ* 7.94–7.90 (m, 2H), 7.32 (s, 1H), 7.31–7.27 (m, 2H), 4.04 (s, 1H), 4.01 (s, 3H); ^13^C{^1^H} NMR (CDCl_3_, 100 MHz): *δ* 190.4 (C), 166.2 (d, C, *J* = 257.5 Hz), 162.0 (C), 161.4 (C), 156.5 (C), 155.1 (C), 133.3 (d, CH, *J* = 9.8 Hz), 118.0 (d, C, *J* = 3.5 Hz), 117.0 (d, CH, *J* = 22.4 Hz), 107.9 (CH), 53.2 (CH_3_), 34.5 (CH); HRMS (ESI) *m*/*z* [M + H]^+^ calcd for C_14_H_10_FN_2_O_4_^+^ 289.0619, found 289.0621.

*(3-(3-(4-Chlorophenyl)-2H-azirine-2-carbonyl)isoxazol-5-yl)methyl benzenesulfonate* (**3q**). Compound **3q** was prepared following the general procedure GP-A from 1-(3-(4-chlorophenyl)-2*H*-azirin-2-yl)-2-diazoethan-1-one **1h** (300 mg, 1.37 mmol), *tert*-butyl nitrite (860 μL, 6.83 mmol), and prop-2-yn-1-yl benzenesulfonate (1.1 mL, 6.83 mmol) in DCM (12 mL) to produce pure product in 389 mg (68% yield), after column chromatography on silica gel (eluent: light petroleum/ethyl acetate, 5:1, (*v*/*v*)) as a brown oil; ^1^H NMR (CDCl_3_, 400 MHz): *δ* 7.95–7.92 (m, 2H), 7.81 (d, 2H, *J* = 8.5 Hz), 7.71–7.68 (m, 1H), 7.60–7.55 (m, 4H), 6.69 (s, 1H), 5.24 (s, 2H), 3.96 (s, 1H); ^13^C{^1^H} NMR (CDCl_3_, 100 MHz): *δ* 185.8 (C), 161.3 (C), 157.0 (C), 150.8 (C), 136.0 (C), 130.7 (C), 129.7 (CH), 127.1 (CH), 125.2 (CH), 124.8 (CH), 123.3 (CH), 115.5 (C), 99.1 (CH), 55.8 (CH_2_), 29.8 (C); HRMS (ESI) *m*/*z* [M + H]^+^ calcd for C_19_H_14_ClN_2_O_5_S^+^ 417.0306, found 417.0304.

*(3-(2-Bromophenyl)-2H-azirin-2-yl)(5-(chloromethyl)isoxazol-3-yl)methanone* (**3r**). Compound **3r** was prepared following the general procedure GP-A from 1-(3-(2-bromophenyl)-2*H*-azirin-2-yl)-2-diazoethan-1-one **1i** (205 mg, 0.78 mmol), *tert*-butyl nitrite (500 μL, 3.88 mmol), and 3-chloroprop-1-yne **7h** (400 μL, 3.88 mmol) in DCM (10 mL) to produce pure product in 197 mg (75% yield), after column chromatography on silica gel (eluent: light petroleum/ethyl acetate, 4:1, (*v*/*v*)) as a brown oil; ^1^H NMR (CDCl_3_, 400 MHz): *δ* 7.88–7.86 (m, 1H), 7.76–7.74 (m, 1H), 7.54–7.46 (m, 2H), 6.77 (s, 1H), 4.68 (s, 2H), 4.09 (s, 1H); ^13^C{^1^H} NMR (CDCl_3_, 100 MHz): *δ* 190.9 (C), 169.1 (C), 162.0 (C), 156.6 (C), 134.8 (CH), 134.2 (CH), 133.6 (CH), 128.0 (CH), 125.9 (C), 122.3 (C), 102.6 (CH), 35.5 (CH), 34.0 (CH_2_) HRMS (ESI) *m*/*z* [M+H]^+^ calcd for C_13_H_9_BrClN_2_O_2_^+^ 340.9509, found 340.9503.

*(5-(Hydroxymethyl)isoxazol-3-yl)(3-(thiophen-2-yl)-2H-azirin-2-yl)methanone* (**3s**). Compound **3s** was prepared following the general procedure GP-A from 2-diazo-1-(3-(thiophen-2-yl)-2*H*-azirin-2-yl)ethan-1-one **1j** (200 mg, 1.05 mmol), *tert*-butyl nitrite (680 μL, 5.25 mmol), and prop-2-yn-1-ol **7c** (302 μL, 5.25 mmol) in DCM (10 mL) to produce pure product in 132 mg (51% yield), after column chromatography on silica gel (eluent: light petroleum/ethyl acetate, 2:1, (*v*/*v*)) as a brown oil; ^1^H NMR (CDCl_3_, 400 MHz): *δ* 7.88 (dd, 1H, *J* = 5.0, 1.2 Hz), 7.69 (dd, 1H, *J* = 3.7, 1.2 Hz), 7.26 (dd, 1H, *J* = 3.7, 1.2 Hz), 6.68 (s, 1H), 4.85 (s, 2H), 4.04 (s, 1H), 2.37 (s, 1H); ^13^C{^1^H} NMR (CDCl_3_, 100 MHz): *δ* 7.89–7.87 (m, 1H), 7.70–7.68 (m, 1H), 7.26–7.24 (m, 1H), 6.68 (s, 1H), 4.85 (s, 2H), 4.04 (s, 1H), 2.37 (br. s, 1H); ^13^C{^1^H} NMR (CDCl_3_, 100 MHz): *δ* 191.1 (C), 173.2 (C), 161.8 (C), 149.6 (C), 136.0 (CH), 135.9 (CH), 128.6 (CH), 123.8 (C), 100.7 (CH), 56.3 (CH_2_), 35.0 (CH); HRMS (ESI) *m*/*z* [M + H]^+^ calcd for C_11_H_8_N_2_O_3_S^+^ 249.0328, found 249.0329.

*(5-(Chloromethyl)isoxazol-3-yl)(3-(thiophen-2-yl)-2H-azirin-2-yl)methanone* (**3t**). Compound **3t** was prepared following the general procedure GP-A from 2-diazo-1-(3-(thiophen-2-yl)-2*H*-azirin-2-yl)ethan-1-one **1j** (200 mg, 1.05 mmol), *tert*-butyl nitrite (680 μL, 5.25 mmol), and 3-chloroprop-1-yne **7g** (560 μL, 5.25 mmol) in DCM (10 mL) to produce pure product in 180 mg (65% yield), after column chromatography on silica gel (eluent: light petroleum/ethyl acetate, 4:1, (*v*/*v*)) as a brown oil; ^1^H NMR (CDCl_3_, 400 MHz): *δ* 7.89 (dd, 1H, *J* = 5.0, 1.2 Hz), 7.59 (dd, 1H, *J* = 3.7, 1.2 Hz), 7.27–7.25 (m, 1H), 6.76 (s, 1H), 4.68 (s, 2H), 4.04 (s, 1H); ^13^C{^1^H} NMR (CDCl_3_, 100 MHz): *δ* 190.7 (C), 169.1 (C), 162.0 (C), 149.5 (C), 136.0 (CH), 135.9 (CH), 128.6 (C), 123.9 (C), 102.6 (CH), 35.0 (CH), 34.0 (CH_2_); HRMS (ESI) *m*/*z* [M + H]^+^ calcd for C_11_H_7_ClN_2_O_2_S^+^ 266.9990, found 266.9993.

*Methyl 3-(3-(tert-butyl)-2H-azirine-2-carbonyl)isoxazole-5-carboxylate* (**3u**). Compound **3u** was prepared following the general procedure GP-A from 1-(3-(*tert*-butyl)-2*H*-azirin-2-yl)-2-diazoethan-1-one **1k** (200 mg, 1.21 mmol), *tert*-butyl nitrite (790 μL, 6.05 mmol), and methyl propiolate **7a** (600 μL, 6.05 mmol) in DCM (10 mL) to produce pure product in 219 mg (72% yield), after column chromatography on silica gel (eluent: light petroleum/ethyl acetate, 4:1, (*v*/*v*)) as a yellow solid: mp 106–108 °C (light petroleum/ethyl acetate); ^1^H NMR (DMSO-*d*_6_, 400 MHz): *δ* 7.60 (s, 1H), 3.94 (s, 3H), 3.71 (s, 1H), 1.26 (s, 9H); ^13^C{^1^H} NMR (DMSO-*d*_6_, 100 MHz): *δ* 191.0 (C), 164.7 (C), 162.3 (C), 161.7 (C), 156.7 (C), 108.4 (CH), 53.7 (CH), 35.0 (CH_3_), 33.8 (C), 25.9 (CH_3_); HRMS (ESI) *m*/*z* [M + H]^+^ calcd for C_12_H_15_N_2_O_4_^+^ 251.1026, found 251.1030.

*Dimethyl 3-(3-phenyl-2H-azirine-2-carbonyl)isoxazole-4,5-dicarboxylate* (**3w**). Compound **3x** was prepared following the general procedure GP-B from 2-diazo-1-(3-phenyl-2*H*-azirin-2-yl)ethan-1-one **1a** (56 mg, 0.3 mmol), *tert*-butyl nitrite (193 μL, 1.5 mmol), dimethyl but-2-ynedioate **7l** (185 μL, 1.5 mmol), and boron trifluoride etherate (2 μL, 0.02 mmol) in DMF (5 mL) to produce pure product in 64 mg (64% yield), after column chromatography on silica gel (eluent: light petroleum/ethyl acetate, 4:1, (*v*/*v*)) as a light-brown oil; ^1^H NMR (CDCl_3_, 400 MHz): *δ* 7.90–7.88 (m, 2H), 7.68–7.64 (m, 1H), 7.60–7.56 (m, 2H), 4.01 (s, 3H), 3.93 (m, 4H); ^13^C{^1^H} NMR (CDCl_3_, 100 MHz): *δ* 189.8 (C), 160.3 (C), 159.0 (C), 158.6 (C), 156.0 (C), 155.6 (C), 134.3 (CH), 130.9 (CH), 129.5 (CH), 121.4 (C), 116.8 (C), 53.6 (CH_3_), 53.5 (CH_3_), 34.9 (CH); IR (KBr, cm^–1^): 3048, 2958, 1757, 1734, 1686, 1452, 1341, 1305, 1272, 1179, 1126, 1099, 983, 841, 823, 768, 692, 568; HRMS (ESI) *m*/*z* [M + Na]^+^ calcd for C_16_H_12_N_2_O_6_Na^+^ 351.0588, found 351.0587.

*(3-(4-Chlorophenyl)-2H-azirin-2-yl)(5-phenylisoxazol-3-yl)methanone* (**3x**). Compound **3x** was prepared following the general procedure GP-B from 1-(3-(4-chlorophenyl)-2*H*-azirin-2-yl)-2-diazoethan-1-one **1k** (175 mg, 0.8 mmol), *tert*-butyl nitrite (507 μL, 3.98 mmol), phenylacetylene **7k** (437 μL, 3.98 mmol), and boron trifluoride etherate (3 μL, 0.04 mmol) in DMF (5 mL) to produce pure product in 164 mg (64% yield), after column chromatography on silica gel (eluent: light petroleum/ethyl acetate, 4:1, (*v*/*v*)) as a yellow solid: mp 140–142 °C (light petroleum/ethyl acetate); ^1^H NMR (CDCl_3_, 400 MHz): *δ* 7.87–7.82 (m, 4H), 7.58–7.52 (m, 2H), 7.51–7.50 (m, 3H), 6.94 (s, 1H), 4.08 (s, 1H); ^13^C{^1^H} NMR (CDCl_3_, 100 MHz): *δ* 191.3 (C), 171.6 (C), 162.4 (C), 155.8 (C), 140.6 (C), 131.9 (CH), 130.9 (CH), 129.9 (CH), 129.2 (CH), 126.6 (C), 126.0 (CH), 120.4 (C), 98.0 (CH), 34.6 (CH); HRMS (ESI) *m*/*z* [M + Na]^+^ calcd for C_18_H_11_ClN_2_O_2_Na^+^ 345.0401, found 345.0400.

*(3-Phenyl-2H-azirin-2-yl)(5-(thiophen-2-yl)isoxazol-3-yl)methanone* (**3y**). Compound **3y** was prepared following the general procedure GP-B from 2-diazo-1-(3-phenyl-2*H*-azirin-2-yl)ethan-1-one **1a** (100 mg, 0.54 mmol), *tert*-butyl nitrite (350 μL, 2.70 mmol), 2-ethynylthiophene **7m** (270 μL, 2.70 mmol), and boron trifluoride etherate (3 μL, 0.03 mmol) in DMF (5 mL) to produce pure product in 81 mg (51% yield), after column chromatography on silica gel (eluent: light petroleum/ethyl acetate, 2:1, (*v*/*v*)) as a yellow solid: mp 85–87 °C (light petroleum/ethyl acetate); ^1^H NMR (CDCl_3_, 400 MHz): *δ* 7.92–7.90 (m, 2H), 7.67–7.63 (m, 1H), 7.59–7.56 (m, 3H), 7.52–7.51 (dd, 1H, *J* = 4.9, 1.2 Hz), 7.18–7.16 (dd, 1H, *J* = 5.0, 3.7 Hz), 6.80 (s, 1H), 4.06 (s, 1H); ^13^C{^1^H} NMR (CDCl_3_, 100 MHz): *δ* 191.3 (C), 166.5 (C), 162.4 (C), 156.3 (C), 134.1 (CH), 130.8 (CH), 129.4 (CH), 129.0 (CH), 128.3 (C), 128.3 (CH), 127.9 (CH), 121.8 (C), 97.6 (CH), 34.5 (CH); HRMS (ESI) *m*/*z* [M + Na]^+^ calcd for C_16_H_10_N_2_O_2_SNa^+^ 317.0355, found 317.0357.

*Methyl 3’-phenyl-[3,5’-biisoxazole]-5-carboxylate* (**4a**). Compound **4a** was prepared following the general procedure GP-C from methyl 3-(3-phenyl-*2H*-azirine-2-carbonyl)isoxazole-5-carboxylate **3a** (42 mg, 0.16 mmol) in acetonitrile (7 mL) to produce pure product in 21 mg (50% yield), after column chromatography on silica gel (eluent: light petroleum/ethyl acetate, 4:1, (*v*/*v*)) as a colorless solid: mp 121–122 °C (light petroleum/ethyl acetate); ^1^H NMR (CDCl_3_, 400 MHz): *δ* 7.88–7.86 (m, 2H), 7.51–7.49 (m, 3H), 7.40 (s, 1H), 7.22 (s, 1H), 4.03 (m, 3H); ^13^C{^1^H} NMR (CDCl_3_, 100 MHz): *δ* 163.3 (C), 161.4 (C), 159.6 (C), 156.5 (C), 153.3 (C), 130.6 (CH), 129.1 (CH), 128.1 (CH), 127.0 (CH), 107.6 (CH), 102.0 (CH), 53.2 (CH_3_); IR (KBr, cm^–1^): 3122, 2949, 1735, 1581, 1482, 1455, 1433, 1394, 1302, 1215, 1141, 997, 930, 834, 771, 694; HRMS (ESI) *m*/*z* [M + H]^+^ calcd for C_14_H_11_N_2_O_4_^+^ 271.0713, found 271.0709.

*2-((3’-Phenyl-[3,5’-biisoxazol]-5-yl)methyl)isoindoline-1,3-dione* (**4b**). Compound **4b** was prepared following the general procedure GP-C from 2-((3-(3-phenyl-2*H*-azirine-2-carbonyl)isoxazol-5-yl)methyl)isoindoline-1,3-dione **3b** (54 mg, 0.15 mmol) in acetonitrile (9 mL) to produce pure product in 9 mg (17% yield), after column chromatography on silica gel (eluent: light petroleum/ethyl acetate, 4:1 + 10% CHCl_3_, (*v*/*v*)).

Compound **4b** was also prepared following the general procedure GP-D from 2-((3-(3-phenyl-2*H*-azirine-2-carbonyl)isoxazol-5-yl)methyl)isoindoline-1,3-dione **3b** (52 mg, 0.15 mmol) and hydroquinone (18 mg, 0.17 mmol) in acetonitrile (9 mL) to produce pure product in 25 mg (48% yield), after column chromatography on silica (light petroleum/ethyl acetate, 4:1 + 10% CHCl_3_, (*v*/*v*)) as a colorless solid: mp 236–238 °C (light petroleum/ethyl acetate); ^1^H NMR (DMSO-*d*_6_, 400 MHz): *δ* 7.96–7.88 (m, 6H), 7.78 (s, 1H), 7.55–7.54 (m, 3H), 7.17 (s, 1H), 5.08 (s, 2H); ^13^C{^1^H} NMR (DMSO-*d*_6_, 100 MHz): *δ* 174.3 (C), 172.2 (C), 167.9 (C), 165.3 (C), 157.5 (C), 140.0 (CH), 136.8 (C), 135.8 (CH), 134.4 (CH), 133.1 (C), 132.0 (CH), 128.6 (CH), 108.6 (CH), 107.0 (C), 38.4 (CH_2_); HRMS (ESI) *m*/*z* [M + H]^+^ calcd for C_21_H_14_N_3_O_4_^+^ 372.0979, found 372.0982.

*(3’-Phenyl-[3,5’-biisoxazol]-5-yl)methanol* (**4c**). Compound **4c** was prepared following the general procedure GP-C from (5-(hydroxymethyl)isoxazol-3-yl)(3-phenyl-2*H*-azirin-2-yl)methanone **3c** (50 mg, 0.21 mmol) in acetonitrile (8 mL) to produce pure product in 9 mg (18% yield), after column chromatography on silica gel (eluent: light petroleum/ethyl acetate, 4:1, (*v*/*v*)).

Compound **4c** was also prepared following the general procedure GP-D from (5-(hydroxymethyl)isoxazol-3-yl)(3-phenyl-2*H*-azirin-2-yl)methanone **3c** (50 mg, 0.21 mmol) and hydroquinone (25 mg, 0.23 mmol) in acetonitrile (8 mL) to produce pure product in 23 mg (46% yield), after column chromatography on silica gel (eluent: light petroleum/ethyl acetate, 4:1, (*v*/*v*)) as a colorless solid: mp 140–141 °C (light petroleum/ethyl acetate); ^1^H NMR (DMSO-*d*_6_ 400 MHz): *δ* 7.97–7.94 (m, 2H), 7.80 (s, 1H), 7.57–7.55 (m, 3H), 6.97 (s, 1H), 5.85–5.82 (t, 1H, *J* = 6.1 Hz), 4.69–4.68 (d, 2H, *J* = 6.1 Hz); ^13^C{^1^H} NMR (DMSO-*d*_6_, 100 MHz): *δ* 175.5 (C), 163.1 (C), 160.9 (C), 152.3 (C), 131.2 (CH), 129.7 (CH), 128.2 (C), 127.3 (CH), 103.6 (CH), 100.9 (CH), 55.2 (CH_2_); HRMS (ESI) *m*/*z* [M + H]^+^ calcd for C_13_H_11_N_2_O_3_^+^ 243.0764, found 243.0761.

*5-(Methoxymethyl)-3’-phenyl-3,5’-biisoxazole* (**4d**). Compound **4d** was prepared following the general procedure GP-C from (5-(methoxymethyl)isoxazol-3-yl)(3-phenyl-2*H*-azirin-2-yl)methanone **3d** (53 mg, 0.21 mmol) in acetonitrile (8 mL) to produce pure product in 33 mg (62% yield), after column chromatography on silica gel (eluent: light petroleum/ethyl acetate, 3:1, (*v*/*v*)) as a colorless solid: mp 125–126 °C (light petroleum/ethyl acetate); ^1^H NMR (CDCl_3_, 400 MHz): *δ* 7.88–7.85 (m, 2H), 7.50–7.49 (m, 3H), 7.15 (s, 1H), 6.76 (s, 1H), 4.64 (s, 2H), 3.49 (s, 3H); ^13^C{^1^H} NMR (CDCl_3_, 100 MHz): *δ* 170.9 (C), 163.1 (C), 160.7 (C), 152.7 (C), 130.4 (CH), 129.1 (CH), 128.3 (C), 126.9 (CH), 101.4 (CH), 101.3 (CH), 65.2 (CH_2_), 59.1 (CH_3_); HRMS (ESI) *m*/*z* [M + H]^+^ calcd for C_15_H_13_N_2_O_4_^+^ 285.0870, found 285.0874.

*5-(Phenoxymethyl)-3’-phenyl-3,5’-biisoxazole* (**4e**). Compound **4e** was prepared following the general procedure GP-C from (5-(phenoxymethyl)isoxazol-3-yl)(3-phenyl-2*H*-azirin-2-yl)methanone **3e** (50 mg, 0.16 mmol) in acetonitrile (8 mL) to produce pure product in 20 mg (40% yield), after column chromatography on silica gel (eluent: light petroleum/ethyl acetate, 4:1 + 10% CHCl_3_, (*v*/*v*)) as a colorless solid: mp 147–148 °C (light petroleum/ethyl acetate); ^1^H NMR (CDCl_3_ 400 MHz): *δ* 7.88–7.85 (m, 2H), 7.50–7.49 (m, 3H), 7.36–7.32 (m, 2H), 7.16 (s, 1H), 7.06–7.02 (m, 1H), 7.01–6.99 (m, 2H), 6.84 (s, 1H), 5.26 (s, 2H); ^13^C{^1^H} NMR (CDCl_3_, 100 MHz): *δ* 169.7 (C), 163.1 (C), 160.6 (C), 157.6 (C), 152.8 (C), 130.5 (CH), 129.8 (CH), 129.1 (CH), 128.3 (C), 126.9 (CH), 122.2 (CH), 114.8 (CH), 101.8 (CH), 101.5 (CH), 61.2 (CH_2_); HRMS (ESI) *m*/*z* [M + H]^+^ calcd for C_19_H_15_N_2_O_3_^+^ 319.1077, found 319.1079.

*(3’-Phenyl-[3,5’-biisoxazol]-5-yl)methyl benzenesulfonate* (**4f**). Compound **4f** was prepared following the general procedure GP-C from (3-(3-phenyl-2*H*-azirine-2-carbonyl)isoxazol-5-yl)methyl benzenesulfonate **3f** (50 mg, 0.13 mmol) in acetonitrile (8 mL) to produce pure product in 26 mg (52% yield), after column chromatography on silica gel (eluent: light petroleum/ethyl acetate, 4:1, (*v*/*v*)) as a yellow solid: mp 150–151 °C (light petroleum/ethyl acetate); ^1^H NMR (CDCl_3_ 400 MHz): *δ* 7.96–7.94 (m, 2H), 7.87–7.84 (m, 2H), 7.71–7.67 (m, 1H), 7.60–7.57 (m, 2H), 7.50–7.49 (m, 3H), 7.13 (s, 1H), 6.75 (s, 1H), 5.27 (s, 2H); ^13^C{^1^H} NMR (CDCl_3_, 100 MHz): *δ* 165.9 (C), 163.2 (C), 160.0 (C), 152.8 (C), 135.5 (C), 134.4 (CH), 130.5 (CH), 129.5 (CH), 129.1 (CH), 128.1 (C), 128.0 (CH), 126.9 (CH), 103.5 (CH), 101.6 (CH), 60.7 (CH_2_); HRMS (ESI) *m*/*z* [M + H]^+^ calcd for C_19_H_15_N_2_O_5_S^+^ 383.0696, found 383.0697.

*5-(Chloromethyl)-3’-phenyl-3,5’-biisoxazole* (**4g**). Compound **4g** was prepared following the general procedure GP-C from (5-(chloromethyl)isoxazol-3-yl)(3-phenyl-2*H*-azirin-2-yl)methanone **3g** (50 mg, 0.19 mmol) in acetonitrile (8 mL) to produce pure product in 31 mg (62% yield), after column chromatography on silica gel (eluent: light petroleum/ethyl acetate, 4:1, (*v*/*v*)) as a yellow solid: mp 175–176 °C (light petroleum/ethyl acetate); ^1^H NMR (CDCl_3_ 400 MHz): *δ* 7.88–7.86 (m, 2H), 7.51–7.49 (m, 3H), 7.17 (s, 1H), 6.83 (s, 1H), 4.71 (s, 2H); ^13^C{^1^H} NMR (CDCl_3_, 100 MHz): *δ* 169.1 (C), 163.2 (C), 160.3 (C), 153.0 (C), 130.5 (CH), 129.1 (CH), 128.2 (C), 126.9 (CH), 102.2 (CH), 101.5 (CH), 34.1 (CH_2_); HRMS (ESI) *m*/*z* [M + H]^+^ calcd for C_13_H_10_ClN_2_O_2_^+^ 261.0425, found 261.0421.

*5-(Bromomethyl)-3’-phenyl-3,5’-biisoxazole* (**4h**). Compound **4h** was prepared following the general procedure GP-C from (5-(bromomethyl)isoxazol-3-yl)(3-phenyl-2*H*-azirin-2-yl)methanone **3h** (50 mg, 0.16 mmol) in acetonitrile (8 mL) to produce pure product in 8 mg (15% yield), after column chromatography on silica gel (eluent: light petroleum/ethyl acetate, 4:1, (*v*/*v*)).

Compound **4h** was also prepared following the general procedure GP-D from (5-(bromomethyl)isoxazol-3-yl)(3-phenyl-2*H*-azirin-2-yl)methanone **3h** (52 mg, 0.17 mmol) and hydroquinone (21 mg, 0.19 mmol) in acetonitrile (8 mL) to produce pure product in 28 mg (52% yield), after column chromatography on silica gel (eluent: light petroleum/ethyl acetate, 4:1, (*v*/*v*)) as a colorless solid: mp 190–192 °C (light petroleum/ethyl acetate); ^1^H NMR (CDCl_3_ 400 MHz): *δ* 7.88–7.85 (m, 2H), 7.51–7.49 (m, 3H), 7.17 (s, 1H), 6.82 (s, 1H), 4.55 (s, 2H); ^13^C{^1^H} NMR (CDCl_3_, 100 MHz): *δ* 169.1 (C), 163.2 (C), 160.3 (C), 153.0 (C), 130.5 (CH), 129.1 (CH), 128.2 (C), 127.0 (CH), 102.2 (CH), 101.5 (CH), 17.9 (CH_2_); HRMS (ESI) *m*/*z* [M + H]^+^ calcd for C_13_H_10_N_2_O_2_^+^ 304.9920, found 304.9922.

*3’-Phenyl-5-(trimethylsilyl)-3,5’-biisoxazole* (**4i**). Compound **4i** was prepared following the general procedure GP-C from (3-phenyl-2*H*-azirin-2-yl)(5-(trimethylsilyl)isoxazol-3-yl)methanone **3i** (50 mg, 0.18 mmol) in acetonitrile (8 mL) to produce pure product in 33 mg (67% yield), after column chromatography on silica gel (eluent: light petroleum/ethyl acetate, 4:1, (*v*/*v*)) as a colorless solid: mp 77–79 °C (light petroleum/ethyl acetate); ^1^H NMR (CDCl_3_, 400 MHz): *δ* 7.88–7.86 (m, 2H), 7.50–7.48 (m, 3H), 7.15 (s, 1H), 6.92 (s, 1H); ^13^C{^1^H} NMR (CDCl_3_, 100 MHz): *δ* 180.2 (C), 163.1 (C), 161.3 (C), 151.1 (C), 130.4 (CH), 129.0 (CH), 128.5 (C), 126.9 (CH), 110.8 (CH), 101.2 (CH), –1.96 (CH_3_); HRMS (ESI) *m*/*z* [M + H]^+^ calcd for C_15_H_17_N_2_O_3_Si^+^ 285.1054, found 285.1049.

*Methyl 3’-(p-tolyl)-[3,5’-biisoxazole]-5-carboxylate* (**4j**). Compound **4j** was prepared following the general procedure GP-C from methyl 3-(3-(*p*-tolyl)-2*H*-azirine-2-carbonyl)isoxazole-5-carboxylate **3j** (45 mg, 0.16 mmol) in acetonitrile (8 mL) to produce pure product in 18 mg (40% yield), after column chromatography on silica gel (eluent: light petroleum/ethyl acetate, 4:1, (*v*/*v*)) as a yellow solid: mp 177–179 °C (light petroleum/ethyl acetate); ^1^H NMR (CDCl_3_ 400 MHz): *δ* 7.76 (d, 2H, *J* = 8.1 Hz), 7.39 (s, 1H), 7.31 (d, 2H, *J* = 8.1 Hz), 7.20 (s, 1H), 4.03 (s, 3H), 2.42 (s, 3H); ^13^C{^1^H} NMR (CDCl_3_, 100 MHz): *δ* 163.2 (C), 161.4 (C), 159.4 (C), 156.6 (C), 153.3 (C), 140.9 (C), 129.8 (CH), 126.8 (CH), 125.2 (C), 107.6 (CH), 102.0 (CH), 53.2 (CH_3_), 21.5 (CH_3_); HRMS (ESI) *m*/*z* [M + H]^+^ calcd for C_15_H_13_N_2_O_4_^+^ 285.0870, found 285.0874.

*Methyl 3’-(4-(tert-butyl)phenyl)-[3,5’-biisoxazole]-5-carboxylate* (**4k**). Compound **4k** was prepared following the general procedure GP-C from methyl 3-(3-(4-(*tert*-butyl)phenyl)-2*H*-azirine-2-carbonyl)isoxazole-5-carboxylate **3k** (34 mg, 0.1 mmol) in acetonitrile (6 mL) to produce pure product in 14 mg (41% yield), after column chromatography on silica gel (eluent: light petroleum/ethyl acetate, 4:1, (*v*/*v*)) as a colorless solid: mp 137–138 °C (light petroleum/ethyl acetate); ^1^H NMR (CDCl_3_, 400 MHz): *δ* 7.80 (d, 2H, *J* = 8.4 Hz), 7.52 (d, 2H, *J* = 8.4 Hz), 7.40 (s, 1H), 7.21 (s, 1H), 4.03 (s, 3H), 1.37 (s, 9H); ^13^C{^1^H} NMR (CDCl_3_, 100 MHz): *δ* 163.2 (C), 161.4 (C), 159.4 (C), 156.5 (C), 154.0 (C), 153.3 (C), 126.7 (CH), 126.1 (CH), 125.2 (C), 107.6 (CH), 102.0 (CH), 53.2 (CH), 34.9 (CH_3_), 31.2 (CH_3_); HRMS (ESI) *m*/*z* [M + H]^+^ calcd for C_18_H_19_N_2_O_4_^+^ 327.1345, found 327.1338.

*Methyl 3’-(4-methoxyphenyl)-[3,5’-biisoxazole]-5-carboxylate* (**4l**). Compound **4l** was prepared following the general procedure GP-C from methyl 3-(3-(4-methoxyphenyl)-*2H*-azirine-2-carbonyl)isoxazole-5-carboxylate **3l** (42 mg, 0.14 mmol) in acetonitrile (8 mL) to produce pure product in 27 mg (67% yield), after column chromatography on silica gel (eluent: light petroleum/ethyl acetate, 4:1 + 10% CHCl_3_, (*v*/*v*)) as a colorless solid: mp 120–122 °C (light petroleum/ethyl acetate); ^1^H NMR (DMSO-*d*_6_, 400 MHz): *δ* 7.89–7.87 (m, 3H), 7.84 (s, 1H), 7.12 (d, 2H, *J* = 8.8 Hz), 3.96 (s, 3H), 3.84 (s, 3H); ^13^C{^1^H} NMR (DMSO-*d*_6_, 100 MHz): *δ* 162.8 (C), 161.6 (C), 161.5 (C), 159.3 (C), 156.7 (C), 153.4 (C), 128.8 (CH), 120.4 (C), 115.2 (CH), 108.8 (CH), 104.6 (CH), 55.9 (CH_3_), 53.7 (CH_3_); HRMS (ESI) *m*/*z* [M + H]^+^ calcd for C_15_H_13_N_2_O_5_^+^ 301.0819, found 301.0812.

*3’-(4-Methoxyphenyl)-5-(phenoxymethyl)-3,5’-biisoxazole* (**4m**). Compound **4m** was prepared following the general procedure GP-C from ((3-(4-methoxyphenyl)-2*H*-azirin-2-yl)(5-(phenoxymethyl)isoxazol-3-yl)methanone **3m** (43 mg, 0.12 mmol) in acetonitrile (8 mL) to produce pure product in 22 mg (50% yield), after column chromatography on silica gel (eluent: light petroleum/ethyl acetate, 4:1 + 10% CHCl_3_, (*v*/*v*)) as a light-brown solid: mp 179–181 °C (light petroleum/ethyl acetate); ^1^H NMR (DMSO-*d*_6_, 400 MHz): *δ* 7.89 (d, 2H, *J* = 8.9 Hz), 7.77 (s, 1H), 7.36 (m, 2H), 7.20 (s, 1H), 7.12–7.08 (m, 4H), 7.03–6.99 (m, 1H), 5.42 (s, 2H), 3.83 (s, 3H); ^13^C{^1^H} NMR (DMSO-*d*_6_, 100 MHz): *δ* 170.3 (C), 162.7 (C), 161.6 (C), 160.2 (C), 157.9 (C), 152.6 (C), 130.2 (CH), 128.8 (CH), 122.1 (CH), 120.5 (C), 115.3 (CH), 115.1 (CH), 103.7 (CH), 103.2 (CH), 60.6 (CH_2_), 55.8 (CH_3_); HRMS (ESI) *m*/*z* [M + H]^+^ calcd for C_20_H_17_N_2_O_4_^+^ 349.1183, found 349.1187.

*5-(Bromomethyl)-3’-(3-methoxyphenyl)-3,5’-biisoxazole* (**4n**). Compound **4n** was prepared following the general procedure GP-C from (5-(bromomethyl)isoxazol-3-yl)(3-(3-methoxyphenyl)-2*H*-azirin-2-yl)methanone **3n** (42 mg, 0.13 mmol) in acetonitrile (6 mL) to produce pure product in 8 mg (18% yield), after column chromatography on silica gel (eluent: light petroleum/ethyl acetate, 4:1, (*v*/*v*)).

Compound **4n** was also prepared following the general procedure GP-D from (5-(bromomethyl)isoxazol-3-yl)(3-(3-methoxyphenyl)-2*H*-azirin-2-yl)methanone **3n** (50 mg, 0.15 mmol) and hydroquinone (18 mg, 0.17 mmol) in acetonitrile (6 mL) to produce pure product in 26 mg (51% yield), after column chromatography on silica gel (eluent: light petroleum/ethyl acetate, 4:1, (*v*/*v*)) as a colorless solid: mp 161–163 °C (light petroleum/ethyl acetate); ^1^H NMR (CDCl_3_, 400 MHz): *δ* 7.43–7.40 (m, 3H), 7.15 (s, 1H), 7.05–7.02 (m, 1H), 6.81 (s, 1H), 4.54 (s, 2H), 3.88 (s, 3H); ^13^C{^1^H} NMR (CDCl_3_, 100 MHz): *δ* 161.1 (C), 163.1 (C), 160.3 (C), 160.1 (C), 153.0 (C), 130.2 (CH), 129.4 (C), 119.4 (CH), 116.6 (CH), 111.9 (CH), 102.2 (CH), 101.6 (CH), 55.4 (CH_3_), 17.8 (CH_2_); HRMS (ESI) *m*/*z* [M + H]^+^ calcd for C_14_H_12_BrN_2_O_3_^+^ 335.0026, found 335.0019.

*3’-(3,4-Dimethoxyphenyl)-5-(trimethylsilyl)-3,5’-biisoxazole* (**4o**). Compound **4o** was prepared following the general procedure GP-C from (3-(3,4-dimethoxyphenyl)-2*H*-azirin-2-yl)(5-(trimethylsilyl)isoxazol-3-yl)methanone **3o** (47 mg, 0.14 mmol) in acetonitrile (8 mL) to produce pure product in 26 mg (55% yield), after column chromatography on silica gel (eluent: light petroleum/ethyl acetate, 4:1, (*v*/*v*)) as a yellow solid: mp 112–114 °C (light petroleum/ethyl acetate); ^1^H NMR (CDCl_3_, 400 MHz): *δ* 7.47 (d, 1H, *J* = 2.0 Hz), 7.37 (dd, 1H, *J* = 8.3, 2.0 Hz), 7.10 (s, 1H), 6.96 (d, 1H, *J* = 8.3 Hz), 6.91 (s, 1H), 3.97 (s, 3H), 3.95 (s, 3H), 0.41 (s, 9H); ^13^C{^1^H} NMR (CDCl_3_, 100 MHz): *δ* 180.1 (C), 162.8 (C), 161.1 (C), 151.1 (C), 150.9 (C), 149.4 (C), 121.2 (C), 120.1 (CH), 111.2 (CH), 110.8 (CH), 109.4 (CH), 101.1 (CH), 56.1 (CH_3_), 56.0 (CH_3_), –1.97 (CH_3_); HRMS (ESI) *m*/*z* [M + H]^+^ calcd for C_21_H_17_N_2_O_4_Si^+^ 345.1271, found 345.1274.

*Methyl 3’-(4-fluorophenyl)-[3,5’-biisoxazole]-5-carboxylate* (**4p**). Compound **4p** was prepared following the general procedure GP-C from methyl 3-(3-(4-fluorophenyl)-2*H*-azirine-2-carbonyl)isoxazole-5-carboxylate **3p** (42 mg, 0.15 mmol) in acetonitrile (7 mL) to produce pure product in 26 mg (62% yield), after column chromatography on silica gel (eluent: light petroleum/ethyl acetate, 4:1, (*v*/*v*)) as a colorless solid: mp 202–204°C (light petroleum/ethyl acetate); ^1^H NMR (DMSO-*d*_6_, 400 MHz): *δ* 8.02–7.98 (m, 2H), 7.90 (s, 1H), 7.89 (s, 1H), 7.44–7.40 (m, 2H), 3.96 (s, 3H); ^13^C{^1^H} NMR (DMSO-*d*_6_, 100 MHz): *δ* 162.3 (C), 161.5 (C), 159.8 (C), 156.7 (C), 146.9 (d, C, *J* = 242.8 Hz), 129.7 (d, CH, *J* = 8.7 Hz), 124.6 (d, C, *J* = 3.3 Hz), 116.9 (d, CH, *J* = 22.0 Hz), 108.7 (CH), 104.7 (CH), 51.7 (CH_3_); HRMS (ESI) *m*/*z* [M + H]^+^ calcd for C_14_H_10_FN_2_O_4_^+^ 289.0619, found 289.0618.

*(3’-(4-Chlorophenyl)-[3,5’-biisoxazol]-5-yl)methyl benzenesulfonate* (**4q**). Compound **4q** was prepared following the general procedure GP-C from (3-(3-(4-chlorophenyl)-2*H*-azirine-2-carbonyl)isoxazol-5-yl)methyl benzenesulfonate **3q** (50 mg, 0.12 mmol) in acetonitrile (7 mL) to produce pure product in 33 mg (66% yield), after column chromatography on silica gel (eluent: light petroleum/ethyl acetate, 4:1, (*v*/*v*)) as a light-yellow solid: mp 158–159 °C (light petroleum/ethyl acetate); ^1^H NMR (CDCl_3_, 400 MHz): *δ* 7.96–7.94 (m, 2H), 7.80–7.78 (m, 2H), 7.71–7.67 (m, 1H), 7.61–7.57 (m, 2H), 7.49–7.46 (m, 2H), 7.10 (s, 1H), 6.76 (s, 1H), 5.27 (s, 2H); ^13^C{^1^H} NMR (CDCl_3_, 100 MHz): *δ* 166.0 (C), 162.2 (C), 160.3 (C), 152.7 (C), 136.7 (C), 135.5 (C), 134.5 (CH), 129.5 (CH), 129.4 (CH), 128.2 (CH), 128.0 (CH), 126.6 (C), 103.4 (CH), 101.5 (CH), 60.6 (CH_2_); HRMS (ESI) *m*/*z* [M + H]^+^ calcd for C_19_H_14_ClN_2_O_5_S^+^ 417.0306, found 417.0294.

*3’-(2-Bromophenyl)-5-(chloromethyl)-3,5’-biisoxazole* (**4r**). Compound **4r** was prepared following the general procedure GP-C from 3-(3-(2-bromophenyl)-2*H*-azirin-2-yl)-5-(chloromethyl)isoxazole **3r** (50 mg, 0.15 mmol) in acetonitrile (7 mL) to produce pure product in 26 mg (52% yield), after column chromatography on silica gel (eluent: light petroleum/ethyl acetate, 4:1, (*v*/*v*)) as a light-brown solid: mp 110–111 °C (light petroleum/ethyl acetate); ^1^H NMR (CDCl_3_, 400 MHz): *δ* 7.74–7.68 (m, 2H), 7.46–7.42 (m, 1H), 7.37–7.33 (m, 1H), 7.30 (s, 1H), 6.83 (s, 1H), 4.71 (s, 2H); ^13^C{^1^H} NMR (CDCl_3_, 100 MHz): *δ* 169.1 (C), 163.1 (C), 159.5 (C), 152.9 (C), 133.8 (CH), 131.4 (CH), 131.4 (CH), 129.6 (C), 127.8 (CH), 122.3 (C), 104.9 (CH), 102.2 (CH), 34.1 (CH_2_); HRMS (ESI) *m*/*z* [M + H]^+^ calcd for C_13_H_9_BrClN_2_O_2_^+^ 338.9530, found 338.9526.

*(3’-(Thiophen-2-yl)-[3,5’-biisoxazol]-5-yl)methanol* (**4s**). Compound **4s** was prepared following the general procedure GP-C from (5-(hydroxymethyl)isoxazol-3-yl)(3-(thiophen-2-yl)-2*H*-azirin-2-yl)methanone **3s** (38 mg, 0.15 mmol) in acetonitrile (8 mL) to produce pure product in 27 mg (71% yield), after column chromatography on silica gel (eluent: light petroleum/ethyl acetate, 1:1, (*v*/*v*)) as a colorless solid: mp 139–141 °C (light petroleum/ethyl acetate); ^1^H NMR (CDCl_3_, 400 MHz): *δ* 7.53 (dd, 1H, *J* = 3.6, 1.2 Hz), 7.46 (dd, 1H, *J* = 5.1, 1.2 Hz), 7.14 (dd, 1H, *J* = 5.1, 3.6 Hz), 7.06 (s, 1H), 6.73 (s, 1H), 4.84 (s, 2H), 3.48 (br. s, 1H); ^13^C{^1^H} NMR (CDCl_3_, 100 MHz): *δ* 175.5 (C), 160.8 (C), 158.6 (C), 152.2 (C), 130.2 (CH), 129.8 (CH), 129.4 (C), 128.7 (CH), 103.4 (CH), 100.9 (CH), 55.2 (CH_2_); HRMS (ESI) *m*/*z* [M + H]^+^ calcd for C_11_H_9_N_2_O_3_S^+^ 249.0328, found 249.0321.

*5-(Chloromethyl)-3’-(thiophen-2-yl)-3,5’-biisoxazole* (**4t**). Compound **4t** was prepared following the general procedure GP-C from (5-(chloromethyl)isoxazol-3-yl)(3-(thiophen-2-yl)-2*H*-azirin-2-yl)methanone **3t** (38 mg, 0.14 mmol) in acetonitrile (8 mL) to produce pure product in 18 mg (47% yield), after column chromatography on silica gel (eluent: light petroleum/ethyl acetate, 4:1, (*v*/*v*)) as a colorless solid: mp 158–160 °C (light petroleum/ethyl acetate); ^1^H NMR (CDCl_3_, 400 MHz): *δ* 7.54 (dd, 1H, *J* = 3.7, 1.2 Hz), 7.47 (dd, 1H, *J* = 5.1, 1.2 Hz), 7.16 (dd, 1H, *J* = 5.1, 3.7 Hz), 7.09 (s, 1H), 6.82 (s, 1H), 4.70 (s, 2H); ^13^C{^1^H} NMR (CDCl_3_, 100 MHz): *δ* 169.2 (C), 160.2 (C), 158.4 (C), 152.8 (C), 129.8 (C), 128.3 (CH), 128.1 (CH), 127.8 (CH), 102.2 (CH), 101.5 (CH), 34.0 (CH_2_); HRMS (ESI) *m*/*z* [M + H]^+^ calcd for C_11_H_8_ClN_2_O_2_S^+^ 266.9900, found 266.9988.

*Methyl 3’-(tert-butyl)-[3,5’-biisoxazole]-5-carboxylate* (**4u**). Compound **4u** was prepared following the general procedure GP-C from methyl 3-(3-(*tert*-butyl)-2*H*-azirine-2-carbonyl)isoxazole-5-carboxylate **3u** (50 mg, 0.2 mmol) in acetonitrile (8 mL) to produce pure product in 22 mg (44% yield), after column chromatography on silica gel (eluent: light petroleum/ethyl acetate, 4:1, (*v*/*v*)) as a colorless solid: mp 95–97 °C (light petroleum/ethyl acetate); ^1^H NMR (CDCl_3_, 400 MHz): *δ* 7.32 (s, 1H), 6.82 (s, 1H), 4.02 (s, 3H), 1.39 (s, 9H); ^13^C{^1^H} NMR (CDCl_3_, 100 MHz): *δ* 172.9 (C), 161.2 (C), 158.5 (C), 156.6 (C), 153.5 (C), 107.5 (CH), 102.1 (CH), 53.1 (CH_3_), 32.2 (C), 29.5 (CH_3_); HRMS (ESI) *m*/*z* [M + H]^+^ calcd for C_12_H_15_N_2_O_4_^+^ 251.1026, found 251.1022.

*Dimethyl 3’-phenyl-[3,5’-biisoxazole]-4,5-dicarboxylate* (**4w**). Compound **4w** was prepared following the general procedure GP-C from dimethyl 3-(3-phenyl-2*H*-azirine-2-carbonyl)isoxazole-4,5-dicarboxylate **3w** (58 mg, 0.18 mmol) in acetonitrile (10 mL) to produce pure product in 24 mg (41% yield), after column chromatography on silica gel (eluent: light petroleum/ethyl acetate, 4:1, (*v*/*v*)) as a yellow solid: mp 150–152 °C (light petroleum/ethyl acetate); ^1^H NMR (CDCl_3_, 400 MHz): *δ* 7.88–7.86 (m, 2H), 7.51–7.49 (m, 3H), 7.32 (s, 1H), 4.05 (s, 3H), 4.01 (s, 3H); ^13^C{^1^H} NMR (CDCl_3_, 100 MHz): *δ* 163.0 (C), 160.3 (C), 160.2 (C), 158.0 (C), 155.9 (C), 151.0 (C), 130.6 (CH), 129.1 (CH), 128.0 (C), 127.0 (CH), 115.1 (C), 104.4 (CH), 53.7 (CH_3_), 53.5 (CH_3_); HRMS (ESI) *m*/*z* [M + H]^+^ calcd for C_16_H_13_N_2_O_6_^+^ 329.0768, found 329.0775.

*3’-(4-Chlorophenyl)-5-phenyl-3,5’-biisoxazole* (**4x**). Compound **4x** was prepared following the general procedure GP-C from (3-(4-chlorophenyl)-2*H*-azirin-2-yl)(5-phenylisoxazol-3-yl)methanone **3x** (51 mg, 0.16 mmol) in acetonitrile (10 mL) to produce pure product in 30 mg (59% yield), after column chromatography on silica gel (eluent: light petroleum/ethyl acetate, 4:1, (*v*/*v*)) as a yellow solid: mp 203–205 °C (light petroleum/ethyl acetate); ^1^H NMR (DMSO-*d*_6_, 400 MHz): *δ* 8.03–8.01 (d, 2H, *J* = 8.6 Hz), 8.00–7.98 (m, 2H), 7.85 (s, 1H), 7.67 (s, 1H), 7.66–7.64 (d, 2H, *J* = 8.7 Hz), 7.62–7.60 (m, 3H); ^13^C{^1^H} NMR (DMSO-*d*_6_, 100 MHz): *δ* 174.4 (C), 171.3 (C), 162.3 (C), 161.0 (C), 153.2 (C), 135.9 (C), 131.7 (CH), 129.9 (CH), 129.9 (CH), 129.1 (CH), 127.1 (C), 126.5 (C), 126.4 (CH), 103.5 (CH), 99.4 (CH); HRMS (ESI) *m*/*z* [M + H]^+^ calcd for C_18_H_12_ClN_2_O_2_^+^ 323.0582, found 323.0583.

*3’-Phenyl-5-(thiophen-2-yl)-3,5’-biisoxazole* (**4y**). Compound **4y** was prepared following the general procedure GP-C from (3-phenyl-2*H*-azirin-2-yl)(5-(thiophen-2-yl)isoxazol-3-yl)methanone **3y** (38 mg, 0.13 mmol) in acetonitrile (10 mL) to produce pure product in 20 mg (53% yield), after column chromatography on silica gel (eluent: light petroleum/ethyl acetate, 4:1, (*v*/*v*)) as a colorless solid: mp 194–196 °C (light petroleum/ethyl acetate); ^1^H NMR (CDCl_3_, 400 MHz): *δ* 7.90–7.87 (m, 2H), 7.63–7.62 (m, 1H), 7.54–7.49 (m, 4H), 7.19–7.17 (m, 2H), 6.87 (s, 1H); ^13^C{^1^H} NMR (CDCl_3_, 100 MHz): *δ* 174.4 (C), 166.5 (C), 163.2 (C), 160.7 (C), 153.2 (C), 130.5 (CH), 129.1 (CH), 128.9 (CH), 128.3 (C), 128.3 (CH), 127.9 (CH), 127.0 (CH), 101.4 (CH), 97.4 (CH); HRMS (ESI) *m*/*z* [M + H]^+^ calcd for C_16_H_11_N_2_O_2_S^+^ 295.0536, found 295.0542.

*3,4-bis(3-(4-Chlorophenyl)isoxazol-5-yl)-1,2,5-oxadiazole 2-oxide* (**8c**). A mixture of 1-(3-(4-chlorophenyl)-2*H*-azirin-2-yl)-2-diazoethan-1-one **1h** (300 mg, 1.37 mmol), *tert*-butyl nitrite (533 μL, 4.11 mmol), and boron trifluoride etherate (9 μL, 70 μmol) in dimethylformamide (4 mL) was stirred at rt for 1 h (monitored by TLC). The reaction mixture was diluted with water and extracted with ethyl acetate. The organic phase was washed with water, dried over Na_2_SO_4_, and the solvent was evaporated. The residue was dissolved in acetonitrile, and the solution was flushed with argon and irradiated using LED 365 at rt for 2 days (monitored by TLC). The solvent was evaporated, and the residue was purified by column chromatography on silica gel (eluent: light petroleum/ethyl acetate, 4:1, (*v*/*v*)) or washed with diethyl ether or acetonitrile to produce pure compound **8c** in 24 mg (44% yield) as a yellow solid: mp 224–225 °C (light petroleum/ethyl acetate); ^1^H NMR (CDCl_3_, 400 MHz): *δ* 8.08–8.06 (m, 4H), 8.03 (s, 1H), 7.95 (s, 1H), 7.68–7.63 (m, 4H); ^13^C{^1^H} NMR (CDCl_3_, 100 MHz): *δ* 162.4 (C), 162.1 (C), 157.3 (C), 155.2 (C), 145.3 (C), 136.2 (C), 130.0 (CH), 129.9 (C), 129.9 (CH), 129.3 (CH), 129.3 (CH), 126.6 (C), 126.5 (C), 107.8 (C), 107.0 (CH), 105.2 (CH); HRMS (ESI) *m*/*z* [M + H]^+^ calcd for C_20_H_11_Cl_2_N_4_O_4_^+^ 441.0152, found 441.0150.

*Methyl 3-(4-acetyl-5-methyl-3-phenyl-1H-pyrrole-2-carbonyl)isoxazole-5-carboxylate* (**5a**). Compound **5a** was prepared following the general procedure GP-E from methyl 3-(3-phenyl-2*H*-azirine-2-carbonyl)isoxazole-5-carboxylate **3a** (50 mg, 0.19 mmol), acetylacetone (60 μL, 0.57 mmol), and Ni(acac)_2_ (10 mg, 0.04 mmol) in acetonitrile (5 mL) at 40 °C to produce pure product in 37 mg (57% yield), after column chromatography on silica gel (eluent: light petroleum/ethyl acetate, 4:1, (*v*/*v*)) as a light-yellow solid: mp 125–127 °C (light petroleum/ethyl acetate); ^1^H NMR (CDCl_3_ 400 MHz): *δ* 10.67 (s, 1H), 7.44–7.40 (m, 3H), 7.33–7.31 (m, 2H), 7.19 (s, 1H), 3.99 (s, 3H), 2.66 (s, 3H), 3.09 (s, 3H); ^13^C{^1^H} NMR (CDCl_3_, 100 MHz): *δ* 196.6 (C), 170.3 (C), 163.3 (C), 160.5 (C), 156.4 (C), 141.5 (C), 137.4 (C), 134.7 (C), 129.5 (CH), 128.3 (CH), 128.1 (CH), 125.0 (C), 124.8 (C), 109.5 (CH), 53.2 (CH_3_), 30.9 (CH_3_), 15.0 (CH_3_); IR (KBr, cm^–1^): 3229, 3140, 2924, 2854, 1739, 1643, 1546, 1514, 1424, 1326, 1286, 1259, 1213, 1072, 994, 890, 755, 701, 558; HRMS (ESI) *m*/*z* [M + H]^+^ calcd for C_19_H_16_N_2_O_5_Na^+^ 375.0951, found 375.0948.

*1-(5-(5-(Chloromethyl)isoxazole-3-carbonyl)-2-methyl-4-phenyl-1H-pyrrol-3-yl)ethan-1-one* (**5b**). Compound **5b** was prepared following the general procedure GP-E from (5-(chloromethyl)isoxazol-3-yl)(3-phenyl-2*H*-azirin-2-yl)methanone **3g** (55 mg, 0.21 mmol), acetylacetone (65 μL, 0.63 mmol) and Ni(acac)_2_ (11 mg, 0.04 mmol) in acetonitrile (5 mL) at 40 °C to produce pure product in 42 mg (55% yield), after column chromatography on silica gel (eluent: light petroleum/ethyl acetate, 4:1, (*v*/*v*)) as a light-yellow solid: mp 153–155 °C (light petroleum/ethyl acetate); ^1^H NMR (CDCl_3_ 400 MHz): *δ* 10.78 (s, 1H), 7.46–7.40 (m, 3H), 7.34–7.31 (m, 2H), 4.63 (s, 2H), 2.65 (s, 3H), 1.83 (s, 3H); ^13^C{^1^H} NMR (CDCl_3_, 100 MHz): *δ* 196.7 (C), 170.8 (C), 168.3 (C), 163.3 (C), 141.1 (C), 137.0 (C), 134.9 (C), 129.5 (CH), 128.2 (CH), 128.0 (CH), 125.0 (C), 124.7 (C), 104.2 (CH), 33.8 (CH_2_), 30.9 (CH_3_), 15.0 (CH_3_); HRMS (ESI) *m*/*z* [M + Na]^+^ calcd for C_18_H_15_ClN_2_O_3_Na^+^ 365.0663, found 365.0662.

*Methyl 3-(4-acetyl-3-(4-methoxyphenyl)-5-methyl-1H-pyrrole-2-carbonyl)isoxazole-5-carboxylate* (**5c**). Compound **5c** was prepared following the general procedure GP-E from methyl 3-(3-(4-methoxyphenyl)-2*H*-azirine-2-carbonyl)isoxazole-5-carboxylate **3l** (60 mg, 0.2 mmol), acetylacetone (60 μL, 0.6 mmol), and Ni(acac)_2_ (10 mg, 0.04 mmol) in acetonitrile (5 mL) at 40 °C to produce pure product in 53 mg (69% yield), after column chromatography on silica gel (eluent: light petroleum/ethyl acetate, 4:1, (*v*/*v*)) as a yellow solid: mp 159–160 °C (light petroleum/ethyl acetate); ^1^H NMR (CDCl_3_ 400 MHz): *δ* 10.63 (s, 1H), 7.23 (d, 2H, *J* = 8.6 Hz), 7.15 (s, 1H), 6.95 (d, 2H, *J* = 8.6 Hz), 3.99 (s, 3H), 3.85 (s, 3H), 2.64 (s, 3H), 1.87 (s, 3H); ^13^C{^1^H} NMR (CDCl_3_, 100 MHz): *δ* 196.8 (C), 170.1 (C), 163.4 (C), 160.4 (C), 159.5 (C), 158.5 (C), 141.3 (C), 137.3 (C), 130.8 (CH), 126.4 (C), 124.98 (C), 124.96 (C), 113.7 (CH), 109.5 (CH), 55.2 (CH_3_), 53.2 (CH_3_), 30.9 (CH_3_), 15.0 (CH_3_); HRMS (ESI) *m*/*z* [M + Na]^+^ calcd for C_20_H_19_N_2_O_6_^+^ 383.1238, found 383.1237.

*(3-(4-Acetyl-3-(4-chlorophenyl)-5-methyl-1H-pyrrole-2-carbonyl)isoxazol-5-yl)methyl benzenesulfonate* (**5d**). Compound **5d** was prepared following the general procedure GP-E from (5(3-(3-(4-chlorophenyl)-2*H*-azirine-2-carbonyl)isoxazol-5-yl)methyl benzenesulfonate **3q** (50 mg, 0.12 mmol), acetylacetone (37 μL, 0.36 mmol), and Ni(acac)_2_ (5 mg, 0.02 mmol) in acetonitrile (5 mL) at 40 °C to produce pure product in 14 mg (24% yield), after column chromatography on silica gel (eluent: light petroleum/ethyl acetate, 4:1, (*v*/*v*)) as a yellow oil; ^1^H NMR (CDCl_3_ 400 MHz): *δ* 10.75 (s, 1H), 7.94–7.92 (m, 2H), 7.71–7.67 (m, 1H), 7.60–7.56 (m, 2H), 7.42–7.40 (m, 2H), 7.26 (m, 2H), 6.66 (s, 1H), 5.21 (s, 2H), 2.64 (s, 3H), 1.86 (s, 3H); ^13^C{^1^H} NMR (CDCl_3_, 100 MHz): *δ* 196.2 (C), 170.2 (C), 165.3 (C), 163.1 (C), 141.1 (C), l35.6 (C), 135.3 (C), 134.5 (CH), 134.1 (C), 133.3 (C), 130.9 (CH), 129.5 (CH), 128.6 (CH), 128.0 (CH), 124.68 (C), 125.67 (C), 105.5 (CH), 60.3 (CH_2_), 31.0 (CH_3_), 15.0 (CH_3_); HRMS (ESI) *m*/*z* [M + H]^+^ calcd for C_24_H_20_ClN_2_O_6_S^+^ 499.0731, found 499.0723.

*Methyl 3-(3-phenyl-5-(thiophen-2-yl)-4-(thiophene-2-carbonyl)-1H-pyrrole-2-carbonyl)isoxazole-5-carboxylate* (**5e**). Compound **5e** was prepared following the general procedure GP-E from methyl 3-(3-phenyl-2*H*-azirine-2-carbonyl)isoxazole-5-carboxylate **3a** (70 mg, 0.26 mmol), 1,3-di(thiophen-2-yl)propane-1,3-dione (69 mg, 0.29 mmol), and Co(acac)_3_ (4 mg, 0.01 mmol) in acetonitrile (5 mL) at 70 °C to produce pure product in 102 mg (80% yield), after column chromatography on silica gel (eluent: light petroleum/ethyl acetate, 2:1, (*v*/*v*)) as a yellow solid: mp 214–215 °C (light petroleum/ethyl acetate); ^1^H NMR (DMSO-*d*_6_, 400 MHz): *δ* 13.00 (s, 1H), 7.87 (dd, 1H*, J* = 5.0, 1.1 Hz), 7.64 (dd, 1H, *J* = 3.6, 1.3 Hz), 7.61 (dd, 1H, *J* = 5.0, 1.1 Hz), 7.35 (dd, 1H, *J* = 3.8, 1.3 Hz), 7.15 (s, 1H), 7.12 (dd, 1H, *J* = 5.0, 3.8 Hz), 7.05 (s, 5H), 6.96 (dd, 1H, *J* = 5.0, 3.8 Hz), 3.85 (s, 3H); ^13^C{^1^H} NMR (DMSO-*d*_6_, 100 MHz): *δ* 185.4 (C), 175.0 (C), 162.6 (C), 159.4 (C), 156.6 (C), 145.1 (C), 136.6 (CH), 136.2 (CH), 135.1 (C), 132.8 (C), 132.4 (C), 131.2 (C), 130.6 (CH), 129.2 (CH), 129.0 (CH), 128.9 (CH), 128.2 (CH), 127.8 (CH), 127.8 (CH), 124.0 (C), 110.1 (C), 53.5 (CH_3_); HRMS (ESI) *m*/*z* [M + H]^+^ calcd for C_25_H_17_N_2_O_5_S_2_^+^ 489.0573, found 489.0581.

*(5-(Chloromethyl)isoxazol-3-yl)(4-(4-methoxybenzoyl)-5-(4-methoxyphenyl)-3-phenyl-1H-pyrrol-2-yl)methanone* (**5f**). Compound **5f** was prepared following the general procedure GP-E from (5-(chloromethyl)isoxazol-3-yl)(3-phenyl-2*H*-azirin-2-yl)methanone **3g** (70 mg, 0.27 mmol), 1,3-bis(4-methoxyphenyl)propane-1,3-dione (85 mg, 0.30 mmol), and Co(acac)_3_ (4 mg, 0.01 mmol) in acetonitrile (5 mL) at 70 °C to produce pure product in 131 mg (92% yield), after column chromatography on silica gel (eluent: light petroleum/ethyl acetate, 2:1, (*v*/*v*)) as a light-yellow solid: mp 86–88 °C (light petroleum/ethyl acetate); ^1^H NMR (DMSO-*d*_6_, 400 MHz): *δ* 12.61 (s, 1H), 7.57 (d, 2H, *J* = 8.9 Hz) 7.46 (d, 2H, *J* = 8.9 Hz), 7.04 (s, 5H), 6.92 (d, 2H, *J* = 8.9 Hz), 6.80 (d, 2H, *J* = 8.9 Hz), 6.61 (s, 1H), 4.79 (s, 2H), 3.73 (s, 3H), 3.71 (s, 3H); ^13^C{^1^H} NMR (DMSO-*d*_6_, 100 MHz): *δ* 192.8 (C), 175.7 (C), 168.1 (C), 163.6 (C), 162.4 (C), 160.1 (C), 138.2 (C), 135.2 (C), 133.4 (C), 132.2 (CH), 131.0 (C), 130.5 (CH), 130.0 (CH), 127.9 (C), 127.7 (CH), 127.6 (CH), 123.7 (C), 122.8 (C), 114.5 (CH), 114.2 (CH), 104.6 (CH), 55.9 (CH_3_), 55.7 (CH_3_), 34.3 (CH_2_); HRMS (ESI) *m*/*z* [M + H]^+^ calcd for C_30_H_24_ClN_2_O_5_^+^ 527.1368, found 527.1378.

*Methyl 3-(4-benzoyl-3-(4-fluorophenyl)-5-phenyl-1H-pyrrole-2-carbonyl)isoxazole-5-carboxylatebenzenesulfonate* (**5g**). Compound **5g** was prepared following the general procedure GP-E from 3-(3-(4-fluorophenyl)-2*H*-azirine-2-carbonyl)isoxazole-5-carboxylate **3p** (70 mg, 0.24 mmol), 1,3-diphenylpropane-1,3-dione (58 mg, 0.26 mmol), and Co(acac)_3_ (4 mg, 0.01 mmol) in acetonitrile (5 mL) at 70 °C to produce pure product in 101 mg (85% yield), after column chromatography on silica gel (eluent: light petroleum/ethyl acetate, 2:1, (*v*/*v*)) as a yellow solid mp 185–187 °C (light petroleum/ethyl acetate); ^1^H NMR (DMSO-*d*_6_ 400 MHz): *δ* 12.97 (s, 1H), 7.58–7.56 (m, 2H), 7.49–7.47 (m, 2H), 7.42–7.38 (m, 1H), 7.34–7.32 (m, 3H), 7.26–7.23 (m, 2H), 7.21 (s, 1H), 7.10–7.07 (m, 2H), 6.89–6.84 (m, 2H), 3.87 (s, 3H); ^13^C{^1^H} NMR (DMSO-*d*_6_, 100 MHz): *δ* 193.7 (C), 175.1 (C), 162.7 (C), 161.9 (d, C, *J* = 245.0 Hz), 159.6 (C), 156.6 (C), 139.9 (C), 138.0 (C), 134.6 (C), 133.6 (CH), 132.9 (d, CH, *J* = 8.3 Hz), 130.1 (C), 129.7 (CH), 129.43 (CH), 129.40 (C), 129.0 (CH), 128.9 (CH), 128.8 (CH), 128.5 (C), 124.1 (C), 114.7 (d, CH, *J* = 21.5 Hz), 110.0 (CH), 53.5 (CH_3_); HRMS (ESI) *m*/*z* [M + H]^+^ calcd for C_29_H_20_FN_2_O_5_^+^ 495.1351, found 495.1361.

*3-(2-phenyloxazol-5-yl)isoxazole-5-carboxylic acid* (**6a**). Compound **6a** was prepared following the general procedure GP-F from methyl 3-(3-phenyl-2*H*-azirine-2-carbonyl)isoxazole-5-carboxylate **3a** (207 mg, 0.77 mmol) and K_2_CO_3_ (425 mg, 3.08 mmol) in methanol (6 mL) to produce pure product in 124 mg (63% yield), after column chromatography on silica gel (eluent: light petroleum/ethyl acetate, 1:1 + 20% CHCl_3_, (*v*/*v*)) as a brown solid: mp 208–210 °C (light petroleum/ethyl acetate); ^1^H NMR (DMSO-*d*_6_, 400 MHz): *δ* 8.12–8.10 (m, 3H), 7.78 (s, 1H), 7.61–7.59 (m, 3H), 3.34 (br, 1H); ^13^C{^1^H} NMR (DMSO-*d*_6_, 100 MHz): *δ* 162.7 (C), 162.6 (C), 157.8 (C), 153.3 (C), 141.1 (C), 132.0 (CH), 130.9 (CH), 129.8 (CH), 127.0 (CH), 126.5 (C), 107.8 (CH); HRMS (ESI) *m*/*z* [M + H]^+^ calcd for C_13_H_9_N_2_O_4_^+^ 257.0557, found 257.0559.

*5-(Methoxymethyl)-3-(2-phenyloxazol-5-yl)isoxazole* (**6b**). Compound **6b** was prepared following the general procedure GP-F from (5-(methoxymethyl)isoxazol-3-yl)(3-phenyl-2*H*-azirin-2-yl)methanone **3d** (97 mg, 0.38 mmol) and K_2_CO_3_ (210 mg, 1.52 mmol) in methanol (4 mL) to produce pure product in 33 mg (34% yield), after column chromatography on silica gel (eluent: light petroleum/ethyl acetate, 4:1, (*v*/*v*)) as a yellow solid: mp 71–72 °C (light petroleum/ethyl acetate); ^1^H NMR (CDCl_3_, 400 MHz): *δ* 8.15–8.12 (m, 2H), 7.66 (s, 1H), 7.51–7.49 (m, 3H), 6.60 (s, 1H), 4.62 (s, 2H), 3.49 (s, 3H); ^13^C{^1^H} NMR (CDCl_3_, 100 MHz): *δ* 170.2 (C), 162.8 (C), 152.5 (C), 141.6 (C), 131.1 (CH), 128.9 (CH), 128.4 (CH), 126.8 (CH), 126.7 (C), 100.6 (CH), 65.3 (CH_2_), 59.0 (CH_3_); HRMS (ESI) *m*/*z* [M + H]^+^ calcd for C_14_H_13_N_2_O_3_^+^ 257.0921, found 257.0926.

*3-(2-Phenyloxazol-5-yl)isoxazole* (**6c**). Compound **6c** was prepared following the general procedure GP-F from (3-phenyl-2*H*-azirin-2-yl)(5-(trimethylsilyl)isoxazol-3-yl)methanone **3i** (165 mg, 0.58 mmol) and K_2_CO_3_ (321 mg, 2.32 mmol) in methanol (4 mL) to produce pure product in 71 mg (58% yield), after column chromatography on silica gel (eluent: light petroleum/ethyl acetate, 4:1, (*v*/*v*)) as a yellow solid: mp 115–117 °C (light petroleum/ethyl acetate); ^1^H NMR (CDCl_3_, 400 MHz): *δ* 8.52–8.51 (d, 1H, *J* = 1.7 Hz), 8.15–8.13 (m, 2H), 7.68 (s, 1H), 7.51–7.49 (s, 3H), 6.70 (d, 1H, *J* = 1.8 Hz); ^13^C{^1^H} NMR (CDCl_3_, 100 MHz): *δ* 162.8 (C), 159.1 (CH), 151.7 (C), 141.6 (C), 131.1 (CH), 128.9 (CH), 128.4 (CH), 126.8 (CH), 126.7 (C), 10.3 (CH); HRMS (ESI) *m*/*z* [M + H]^+^ calcd for C_12_H_9_N_2_O_2_^+^ 213.0659, found 213.0662.

*3-(2-(3,4-Dimethoxyphenyl)oxazol-5-yl)isoxazole* (**6d**). Compound **6d** was prepared following the general procedure GP-F from (3-(3,4-dimethoxyphenyl)-2*H*-azirin-2-yl)(5-(trimethylsilyl)isoxazol-3-yl)methanone **3o** (176 mg, 0.51 mmol) and K_2_CO_3_ (282 mg, 2.04 mmol) in methanol (4 mL) to produce pure product in 67 mg (48% yield), after column chromatography on silica gel (eluent: light petroleum/ethyl acetate, 4:1, (*v*/*v*)) as a yellow solid: mp 150–152 °C (light petroleum/ethyl acetate); ^1^H NMR (CDCl_3_, 400 MHz): *δ* 8.51–8.50 (d, 1H, *J* = 1.7 Hz), 7.75–7.73 (dd, 1H, *J* = 8.4, 2.0 Hz), 7.63 (s, 2H), 6.97–6.95 (d, 1H, *J* = 8.3 Hz), 6.68 (d, 1H, *J* = 1.7 Hz), 3.99 (s, 3H), 3.96 (s, 3H); ^13^C{^1^H} NMR (CDCl_3_, 100 MHz): *δ* 163.0 (C), 159.0 (CH), 151.7 (C), 151.6 (C), 149.3 (C), 141.1 (C), 128.5 (CH), 120.3 (CH), 119.5 (C), 111.1 (CH), 109.4 (CH), 102.2 (CH), 56.1 (CH_3_), 56.0 (CH_3_); IR (KBr, cm^–1^): 3110, 2917, 1609, 1496, 1438, 1281, 1248, 1139, 1024, 877, 815, 768, 737; HRMS (ESI) *m*/*z* [M + H]^+^ calcd for C_14_H_13_N_2_O_4_^+^ 273.0870, found 273.0875.

*3-(2-(4-Chlorophenyl)oxazol-5-yl)-5-phenylisoxazole* (**6e**). Compound **6e** was prepared following the general procedure GP-F from (3-(4-chlorophenyl)-2*H*-azirin-2-yl)(5-phenylisoxazol-3-yl)methanone **3x** (99 mg, 0.31 mmol) and K_2_CO_3_ (171 mg, 1.24 mmol) in methanol (4 mL) to produce pure product in 34 mg (34% yield), after column chromatography on silica gel (eluent: light petroleum/ethyl acetate, 4:1, (*v*/*v*)) as a yellow solid: mp 190–192 °C (light petroleum/ethyl acetate); ^1^H NMR (DMSO-*d*_6_, 400 MHz): *δ* 8.12–8.10 (d, 2H, *J* = 8.6 Hz), 8.04 (s, 1H), 7.95–7.93 (m, 2H), 7.70–7.68 (d, 2H, *J* = 8.6 Hz), 7.61 (s, 1H), 7.60–7.56 (m, 3H); ^13^C{^1^H} NMR (DMSO-*d*_6_, 100 MHz): *δ* 170.5 (C), 161.5 (C), 153.2 (C), 142.1 (C), 136.6 (C), 131.4 (CH), 130.0 (CH), 129.9 (CH), 129.9 (CH), 128.6 (CH), 126.7 (C), 126.2 (CH), 125.5 (C), 98.9 (CH); HRMS (ESI) *m*/*z* [M + H]^+^ calcd for C_18_H_12_ClN_2_O_2_^+^ 323.0582, found 323.0586.

## 4. Conclusions

A method for generating azirinyl-substituted nitrile oxides by the reaction of 2-(diazoacetyl)-2*H*-azirines with *tert*-butyl nitrite, while preserving the azirine ring, has been developed. Terminal acetylenes with azirinyl-substituted nitrile oxides produce [3+2] cycloaddition products, (2*H*-azirin-2-yl)(isoxazol-3-yl)methanones, in a 51–91% yield at room temperature in DCM. The reaction allows one to obtain azirine–isoxazole hybrids with various substituents at position 3 of azirine and position 5 of isoxazole. DFT calculations and experimental data are consistent with the assumption that the formation of azirinyl-substituted nitrile oxides is accelerated by the acid catalyst. Cycloadducts of nitrile oxides with aryl/hetarylacetylenes and DMAD can be obtained using catalysis with boron trifluoride etherate, which significantly expands the scope of the reaction. Expansion of the azirine ring of the prepared cycloadducts allows obtaining a wide range of structurally diverse functionalized isoxazole-containing heterocyclic hybrids. A total of 365 nm LED light causes isomerization of the azirinecarbonyl fragment of (2*H*-azirin-2-yl)(isoxazol-3-yl)methanones into the corresponding isoxazole, which made it possible to obtain a set of substituted 3,5’-biisoxazoles in a 40–71% yield. The reaction of 1,3-diketones with the azirine moiety of (2*H*-azirin-2-yl)(isoxazol-3-yl)methanones, catalyzed by Ni(acac)_2_ (in the case of acetylacetone) or Co(acac)_3_ (in the case of aryl/hetaryl-substituted 1,3-diketones), allows the preparation of heterocyclic hybrids containing substituted pyrrole and isoxazole moieties in a 24–92% yield. 2-(Isoxazole-3-ylcarbonyl)-3-arylazirines were also easily isomerized in methanol in the presence of excess K_2_CO_3_ at room temperature to produce 3-(oxazol-5-yl)isoxazoles in a 34–63% yield.

## Figures and Tables

**Table 1:** Optimization of isoxazole **3a** synthesis *^a^*.

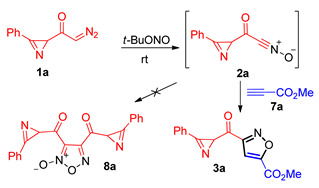			
**Entry**	**Solvent**	**Time, d**	**Yield of 3a (%) *^b^***
1	CHCl_3_	1	77
2	MeCN	1	61
3	THF	2	69
4	DCM	2	91
5	DCE	2	65

*^a^* Reaction conditions: 1a (0.3 mmol), TBN (1.5 mmol), and alkyne **7a** (1.5 mmol) in 5 mL of solvent. *^b^* Isolated yields.

**Table 2:** Relative Gibbs energy for the reaction of nitrile oxide **2a** with alkynes **7a,g,i**–**l** *^a.^*

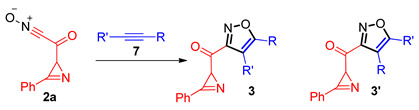				
** 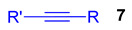 **	**TS ^(^** **^2a+7→3)^**	**3**	**TS ^(^** **^2a+7→3^** **^’)^**	**3’**
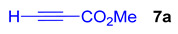	22.2	−57.9 (**3a**)	25.5	−56.1 (**3’a**)
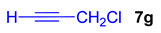	23.8	−58.0 (**3g**)	27.8	−55.6 (**3’g**)
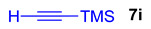	21.7	−50.9 (**3o**)	25.6	−51.3 (**3’o**)
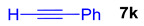	21.0 (21.8) *^b^*	−57.5 (−57.6) *^b^* (**3v**)	30.0 (30.3) *^b^*	−50.6 (−51.7) *^b^* (**3’v**)
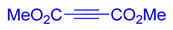	24.8	−54.8 (**3w**)		

*^a^* DFT calculations at the B3LYP-D3/6-311+G(d,p) level of theory with SMD solvent model for DCM at 298 K (kcal/mol). *^b^* SMD solvent model for DMF.

**Table 3:** Relative Gibbs free energy for the reaction of nitronate ***B*** with alkynes **7a,g,i,k,l** *^a.^*

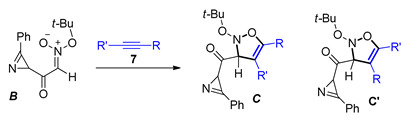				
** 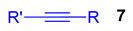 **	**TS ^(*B*+7→*C*)^**	***C***	**TS ^(*B* +7→*C*’)^**	***C*’**
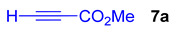	25.8	−25.6 (***C*a**)	26.6	−29.0 (***C*’a**)
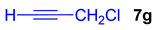	26.0	−24.1 (***C*g**)	28.9	−25.0 (***C*’g**)
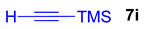	26.4	−19.0 (***C*o**)	28.1	−19.2 (***C*’o**)
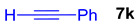	27.4 (27.4) *^b^*	−23.4 (−23.8) *^b^* (***C*v**)	30.5 (30.7)*^b^*	−20.6 (−22.0) *^b^* (***C*’v**)
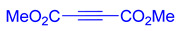	24.5	−26.7 (***C*w**)		

*^a^* DFT calculations at the B3LYP-D3/6-311+G(d,p) level of theory with SMD solvent model for DCM at 298 K (kcal/mol). *^b^* SMD solvent model for DMF.

**Table 4:** Optimization of catalytic conditions for the synthesis of azirinyl(isoxazolyl)ketone **3x** *^a**.**^*

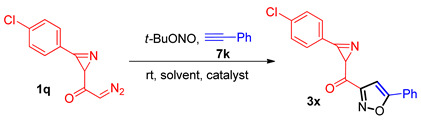				
**Entry**	**Solvent**	**Catalyst**	**Time, h**	**Yield of 3x (%) *^b^***
1	DCM	PTSA (10 mol%)	3	21
2	DCM	BF_3_·Et_2_O (10 mol%)	1	15
3	MeCN	PTSA (110 mol%)	1	26
4	DMF	PTSA (10 mol%)	3	44
5	DMF	BF_3_·Et_2_O (10 mol%)	1	46
**6**	**DMF**	**BF_3_·Et_2_O (5 mol%)**	**1**	**64**
7	DMF	PPTS (10 mol%)	3	45
8	DMF	FeCl_2_ (20 mol%)	3	32

*^a^* Reaction conditions: 1q (0.2 mmol), TBN (0.6 mmol), and acetylene 7k (0.6 mmol) in 2 mL of solvent. *^b^* Isolated yield.

**Table 5:** Optimization of biisoxazole **4i** synthesis *^a^*.

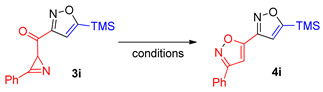					
**Entry**	**Solvent**	**Catalyst or LED**	**T,** **°** **C**	**Time**	**Yield of 4i (%) *^b^***
1	MeCN	254 nm	25	1.5 h	-*^c^*
2	MeOH	254 nm	25	1.5 h	-*^c^*
**3**	**MeCN**	**365 nm**	**25**	**2 d**	**67**
4	MeOH	365 nm	25	2 d	-*^d^*
5	Et_2_O	365 nm	25	2 d	- *^d^*
6	MeCN	405 nm	25	1 d	- *^e^*
7	MeCN	Cold White	25	1 d	- *^e^*
8	mesitylene	-	180	0.5 h	- *^c^*
9	mesitylene	-	200	1.5 h	56
10	MeCN	FeCl_2_·4H_2_O (20 mol%)	25–60	2 h	- *^c^*
11	MeCN	FeSO_4_·2H_2_O (10 mol%)	40	2 h	- *^e^*
12	MeCN	NiCl_2_·6H_2_O (10 mol%)	40	2 h	- *^e^*
13	MeCN	Co(acac)_3_ (10 mol%)	40	2 h	- *^e^*
14	MeCN	Rh_2_(OAc)_4_ (5 mol%)	40	2 h	- *^e^*

*^a^* 0.03M solution of **3i**. *^b^* Isolated yield. *^c^* Resignification, decomposition of **3i**. *^d^* Incomplete conversion of **3i**. *^e^* No reaction.

## Data Availability

Data are contained within the article.

## Supplementary Materials

The following supporting information can be downloaded at: https://www.mdpi.com/article/10.3390/molecules30132834/s1, X-Ray diffraction experiment; NMR spectra of compounds **3**; **4; 8c**; **5**; **6**; IR spectra of compounds **3a**; **3w**; **4a; 5a**; **6d**; Computational details. References [79,80,81,82,83,84,85,86,87,88,89] are cited in the Supplementary Materials.

## References

[1] Martis G.J., Gaonkar S.L. (2025). Advances in isoxazole chemistry and their role in drug discovery. RSC Adv..

[2] Wang J., Wang D.B., Sui L.L., Luan T. (2024). Natural products-isoxazole hybrids: A review of developments in medicinal chemistry. Arab. J. Chem..

[3] Shinde Y., Khairnar B., Bangale S. (2024). Exploring the Diverse Biological Frontiers of Isoxazole: A Comprehensive Review of its Pharmacological Significance. ChemistrySelect.

[4] Zawadzińska-Wrochniak K., Zavecz I., Hirka S. (2024). The recent progress in the field of the applications of isoxazoles and their hydrogenated analogs: Mini review. Sci. Radices.

[5] Pandhurnekar C.P., Pandhurnekar H.C., Mungole A.J., Butoliya S.S., Yadao B.G. (2023). A review of recent synthetic strategies and biological activities of isoxazole. J. Heterocycl. Chem..

[6] Okhovat A., Cruces W., Docampo-Palacios M.L., Ray K.P., Ramirez G.A. (2023). Psychoactive Isoxazoles, Muscimol, and Isoxazole Derivatives from the Amanita (Agaricomycetes) Species: Review of New Trends in Synthesis, Dosage, and Biological Properties. Int. J. Med. Mushrooms.

[7] Arya G.C., Kaur K., Jaitak V. (2021). Isoxazole derivatives as anticancer agent: A review on synthetic strategies, mechanism of action and SAR studies. Eur. J. Med. Chem..

[8] Agrawal N., Mishra P. (2018). The synthetic and therapeutic expedition of isoxazole and its analogs. Med. Chem. Res..

[9] Zhu J., Mo J., Lin H.Z., Chen Y., Sun H.P. (2018). The recent progress of isoxazole in medicinal chemistry. Bioorg. Med. Chem..

[10] Viegas C.J., Danuello A., Bolzani V.S., Barreiro E.J., Fraga C.M. (2007). Molecular hybridization: A useful tool in the design of new drug prototypes. Curr. Med. Chem..

[11] Meunier B. (2008). Hybrid molecules with a dual mode of action: Dream or reality?. Acc. Chem. Res..

[12] Oliveira Pedrosa M., Duarte da Cruz R., Oliveira Viana J., de Moura R., Ishiki H., Barbosa Filho J., Diniz M.F.F.M., Scotti M.T., Scotti L., Bezerra Mendonca F.J. (2016). Hybrid compounds as direct multitarget ligands: A review. Curr. Top. Med. Chem..

[13] Shaveta S.M., Singh P. (2016). Hybrid molecules: The privileged scaffolds for various pharmaceuticals. Eur. J. Med. Chem..

[14] Ivasiv V., Albertini C., Gonçalves A.E., Rossi M., Bolognesi M.L. (2019). Molecular Hybridization as a Tool for Designing Multitarget Drug Candidates for Complex Diseases. Curr. Top. Med. Chem..

[15] de Sena Murteira Pinheiro P., Franco L.S., Montagnoli T.L., Fraga C.A.M. (2024). Molecular hybridization: A powerful tool for multitarget drug discovery. Expert Opin. Drug Discov..

[16] Saeedi M., Mohtadi-Haghighi D., Mirfazli S.S., Mahdavi M., Hariri R., Lotfian H., Edraki N., Iraji A., Firuzi O., Akbarzadeh T. (2019). Design and synthesis of selective acetylcholinesterase inhibitors: Arylisoxazole-phenylpiperazine derivatives. Chem. Biodivers..

[17] Rastegari A., Safavi M., Vafadarnejad F., Najafi Z., Hariri R., Bukhari S.N.A., Iraji A., Edraki N., Firuzi O., Saeedi M. (2022). Synthesis and evaluation of novel arylisoxazoles linked to tacrine moiety: In vitro and in vivo biological activities against Alzheimer’s disease. Mol. Divers..

[18] Saeedi M., Rastegari A., Hariri R., Mirfazli S.S., Mahdavi M., Edraki N., Firuzi O., Akbarzadeh T. (2020). Design and synthesis of novel arylisoxazole-chromenone carboxamides: Investigation of biological activities associated with Alzheimer’s disease. Chem. Biodivers..

[19] Vafadarnejad F., Karimpour-Razkenari E., Sameem B., Saeedi M., Akbarzadeh T. (2019). Novel *N*-benzylpyridinium moiety linked to arylisoxazole derivatives as selective butyrylcholinesterase inhibitors: Synthesis, biological evaluation, and docking study. Bioorg. Chem..

[20] Zhang H., Peng Y., Zhuo L., Wang Y., Zeng G., Wang S., Long L., Li X., Wang Z. (2022). Recent advance on pleiotropic cholinesterase inhibitors bearing amyloid modulation efficacy. Eur. J. Med. Chem..

[21] Hawash M., Kahraman D.C., Ergun S.G., Cetin-Atalay R., Baytas S.N. (2021). Synthesis of novel indole-isoxazole hybrids and evaluation of their cytotoxic activities on hepatocellular carcinoma cell lines. BMC Chem..

[22] Güiza F.M., Duarte Y.B., Mendez-Sanchez S.C., Bohórquez A.R.R. (2019). Synthesis and in vitro evaluation of substituted tetrahydroquinoline-isoxazole hybrids as anticancer agents. Med. Chem. Res..

[23] Galenko E.E., Novikov M.S., Bunev A.S., Khlebnikov A.F. (2024). Acridine–Isoxazole and Acridine–Azirine Hybrids: Synthesis, Photochemical Transformations in the UV/Visible Radiation Boundary Region, and Anticancer Activity. Molecules.

[24] Wang M., Li L., Yang S., Guo F., Zhu G., Zhu B., Chang J. (2023). Synthesis of novel oxazol-5-one derivatives containing chiral trifluoromethyl and isoxazole moieties as potent antitumor agents and the mechanism investigation. Bioorg. Chem..

[25] Lingala A.K., Murahari K.K., Desireddi J.R., Mothe T., Mait B., Manchal R. (2023). Design, synthesis and biological evaluation of isoxazole bearing 1,3-oxazole-1,3,4-oxadiazole derivatives as anticancer agents. Chem. Data Collect..

[26] Kushwaha P.K., Srivastava K.S., Kumari N., Kumar R., Mitra D., Sharon A. (2022). Synthesis and anti-HIV activity of a new isoxazole containing disubstituted 1,2,4-oxadiazoles analogs. Bioorg. Med. Chem..

[27] Kankala S., Kankala R.K., Gundepaka P., Thota N., Nerella S., Gangula M.R., Guguloth H., Kagga M., Vadde R., Vasam C. (2013). Regioselective synthesis of isoxazole–mercaptobenzimidazole hybrids and their in vivo analgesic and anti-inflammatory activity studies. Bioorg. Med. Chem. Lett..

[28] Wang X., Hu Q., Tang H., Pan X. (2023). Isoxazole/Isoxazoline skeleton in the structural modification of natural products: A review. Pharmaceuticals.

[29] Das S., Chanda K. (2021). An overview of metal-free synthetic routes to isoxazoles: The privileged scaffold. RSC Adv..

[30] Madhavan S., Keshri S.K., Kapur M. (2021). Transition Metal-Mediated Functionalization of Isoxazoles: A Review. Asian J. Org. Chem..

[31] Bondarenko O.B., Zyk N.V. (2020). The main directions and recent trends in the synthesis and use of isoxazoles. Chem. Heterocycl. Compd..

[32] Morita T., Yugandar S., Fuse S., Nakamura H. (2018). Recent progresses in the synthesis of functionalized isoxazoles. Tetrahedron Lett..

[33] Fadda A.A., Tawfik E.H., Selim Y.A. (2020). Synthesis and biological evaluation of some new thiophene, thiazole, dithiolane derivatives and related compounds. Polycycl. Aromat. Compd..

[34] Padmavathi V., Reddy B.J.M., Subbaiah D.V. (2004). Bischalcones—Synthons for a new class of bis(heterocycles). New J. Chem..

[35] Dattolo G., Aiello E., Plescia S., Cirrincione G., Daidone G. (1977). New syntheses of condensed heterocycles from isoxazole derivatives. V. Pyrrolo [3,4-*b*] pyridin-4-ones. J. Heterocycl. Chem..

[36] Nakao K., Stevens R.W., Kawamura K., Uchida C., Koike H., Caron S. (1999). 2,3-Substituted Indole Compounds as COX-2. Inhibitors. Patent.

[37] Cannas M., Marongiu G. (1967). Crystal and molecular structure of 3-3′ biisoxazole. Z. Krist.-Cryst. Mater..

[38] Biagini S., Cannas M., Marongiu G. (1969). Crystal and molecular structure of 3,4’-biisoxazole. Acta Crystallogr. B..

[39] Laćan M., Filipović-Marinić N., Stefanović D. (1977). The Synthesis and Structure Determination of Tetrasubstituted 4, 4’-Biisoxazoles. Croat. Chem. Acta.

[40] Wingard L.A., Guzmán P.E., Johnson E.C., Sabatini J.J., Drake G.W., Byrd E.F. (2017). Synthesis of bis-Isoxazole-bis-methylene Dinitrate: A Potential Nitrate Plasticizer and Melt-castable Energetic Material. ChemPlusChem.

[41] Xiao H.-Y., Watterson S.H., Langevine C.M., Srivastava A.S., Ko S.S., Zhang Y., Cherney R.J., Guo W.-W., Gilmore J.L., Sheppeck J.E. (2016). Identification of Tricyclic Agonists of Sphingosine-1-Phosphate Receptor 1 (S1P1) Employing Ligand-Based Drug Design. J. Med. Chem..

[42] Kotoku M., Maeba T., Fujioka S., Yokota M., Seki N., Ito K., Suwa Y., Ikenogami T., Hirata K., Hase Y. (2019). Discovery of second generation RORγ inhibitors composed of an azole scaffold. J. Med. Chem..

[43] Azzali E., Girardini M., Annunziato G., Pavone M., Vacondio F., Mori G., Pasca M.R., Costantino G., Pieroni M. (2020). 2-Aminooxazole as a novel privileged scaffold in antitubercular medicinal chemistry. ACS Med. Chem. Lett..

[44] Magalhães J., Franko N., Raboni S., Annunziato G., Tammela P., Bruno A., Bettati S., Armao S., Spadini C., Cabassi C.S. (2021). Discovery of Substituted (2-Aminooxazol-4-yl)Isoxazole-3-carboxylic Acids as Inhibitors of Bacterial Serine Acetyltransferase in the Quest for Novel Potential Antibacterial Adjuvants. Pharmaceuticals.

[45] Elmegeed G.A., Baiuomy A.R., Abdelhalim M.M., Hana H.Y. (2010). Synthesis and antidepressant evaluation of five novel heterocyclic tryptophan-hybrid derivatives. Arch. Pharm. Chem. Life Sci..

[46] De Angelis L., Crawford A.M., Su Y.L., Wherritt D., Arman H., Doyle M.P. (2021). Catalyst-Free Formation of Nitrile Oxides and Their Further Transformations to Diverse Heterocycles. Org Lett..

[47] Sakharov P.A., Novikov M.S., Khlebnikov A.F. (2018). 2-Diazoacetyl-2*H*-azirines: Source of a Variety of 2*H*-Azirine Building Blocks with Orthogonal and Domino Reactivity. J. Org. Chem..

[48] Bodunov V.A., Galenko E.E., Sakharov P.A., Novikov M.S., Khlebnikov A.F. (2019). Selective Cu-Catalyzed Intramolecular Annulation of 3-Aryl/Heteryl-2-(diazoacetyl)-1*H*-pyrroles: Synthesis of Benzo/Furo/Thieno[*e*]-Fused 1*H*-Indol-7-oles and Their Transformations. J. Org. Chem..

[49] Galenko E.E., Bodunov V.A., Kryukova M.A., Novikov M.S., Khlebnikov A.F. (2021). Buchner Reaction/Azirine Modification Approach Toward Cycloheptatriene Containing Nitrogen Heterocyclic Scaffolds. J. Org. Chem..

[50] Zanakhov T.O., Galenko E.E., Kryukova M.A., Novikov M.S., Khlebnikov A.F. (2021). Isomerization of 5-(2*H*-Azirin-2-yl)oxazoles: An Atom-Economic Approach to 4*H*-Pyrrolo [2,3-*d*]oxazoles. Molecules.

[51] Zanakhov T.O., Galenko E.E., Novikov M.S., Khlebnikov A.F. (2022). Diazo Strategy for Intramolecular Azirine Ring Expansion: Rh(II)-Catalyzed Synthesis of 2-Hydroxy-3-oxo-2,3-dihydro-1*H*-pyrrole-2-carboxylates. J. Org. Chem..

[52] Wang X.D., Zhu L.H., Liu P., Wang X.Y., Yuan H.Y., Zhao Y.L. (2019). Copper-catalyzed cascade cyclization reactions of diazo compounds with *tert*-butyl nitrite and alkynes: One-pot synthesis of isoxazoles. J. Org. Chem..

[53] Ma L., Kou L., Jin F., Cheng X., Tao S., Jiang G., Bao X., Wan X. (2021). Acyclic nitronate olefin cycloaddition (ANOC): Regio-and stereospecific synthesis of isoxazolines. Chem. Sci..

[54] Dahiya A., Sahoo A.K., Alam T., Patel B.K. (2019). *tert*-Butyl Nitrite (TBN), a Multitasking Reagent in Organic Synthesis. Chem. Asian J..

[55] Zhang Y., Wang J. (2009). Recent Development of Reactions with α-Diazocarbonyl Compounds as Nucleophiles. Chem. Commun..

[56] Heimgartner H. (1991). 3-Amino-2*H*-Azirines. Synthons for α,α-Disubstituted α-Amino Acids in Heterocycle and Peptide Synthesis. Angew. Chem. Int. Ed..

[57] Palacios F., Ochoa de Retana A.M., Martínez de Marigorta E., de los Santos J.M. (2001). 2*H*-Azirines as Synthetic Tools in Organic Chemistry. Eur. J. Org. Chem..

[58] Palacios F., de Retana A.M., Martínez de Marigorta E., Manuel de los Santos J. (2002). Preparation, properties and synthetic applications of 2*H*-azirines. A review. Org. Prep. Proced. Int..

[59] Padwa A. (2010). Cycloaddition and cyclization chemistry of 2*H*-azirines. Adv. Heterocycl. Chem..

[60] Khlebnikov A.F., Novikov M.S. (2013). Recent Advances in 2*H*-Azirine Chemistry. Tetrahedron.

[61] Khlebnikov A.F., Novikov M.S., Rostovskii N.V. (2019). Advances in 2*H*-Azirine Chemistry: A Seven-Year Update. Tetrahedron.

[62] Xu F., Zeng F.-W., Luo W.-J., Zhang S.-Y., Huo J.-Q., Li Y.-P. (2024). 2*H*-Azirines: Recent Progress in Synthesis and Applications. Eur. J. Org. Chem..

[63] Galenko E.E., Khlebnikov A.F., Novikov M.S. (2016). Isoxazole-Azirine Isomerization as a Reactivity Switch in the Synthesis of Heterocycles. Chem. Heterocycl. Compd..

[64] Ullman E.F., Singh B. (1966). Photochemical Transposition of Ring Atoms in Five-Membered Heterocycles. The Photorearrangement of 3,5-Diphenylisoxazol. J. Am. Chem. Soc..

[65] Gamage D.W., Li Q., Ranaweera R.A.A.U., Sarkar S.K., Weragoda G.K., Carr P.L., Gudmundsdottir A.D. (2013). Vinylnitrene Formation from 3,5-Diphenyl-isoxazole and 3-Benzoyl-2-phenyl-azirine. J. Org. Chem..

[66] Li X., Du Y., Liang Z., Li X., Pan Y., Zhao K. (2009). Simple Conversion of Enamines to 2*H*-Azirines and Their Rearrangements under Thermal Conditions. Org. Lett..

[67] Zheng Y., Yang C., Zhang-Negrerie D., Du Y., Zhao K. (2013). One-pot synthesis of isoxazoles from enaminones: An application of Fe (II) as the catalyst for ring expansion of 2*H*-azirine intermediates. Tetrahedron Lett..

[68] Sun X., Lyu Y., Zhang-Negrerie D., Du Y., Zhao K. (2013). Formation of Functionalized 2*H*-Azirines through PhIO-Mediated Trifluoroethoxylation and Azirination of Enamines. Org. Lett..

[69] Yuan Y., Hou W., Zhang-Negrerie D., Zhao K., Du Y. (2014). Direct Oxidative Coupling of Enamines and Electron-Deficient Amines: TBAI/TBHP-Mediated Synthesis of Substituted Diaminoalkenes under Metal-Free Conditions. Org. Lett..

[70] Serebryannikova A.V., Galenko E.E., Novikov M.S., Khlebnikov A.F. (2019). Synthesis of Isoxazole- and Oxazole-4-carboxylic Acids Derivatives by Controlled Isoxazole-Azirine-Isoxazole/Oxazole Isomerization. J. Org. Chem..

[71] Galenko E.E., Galenko A.V., Khlebnikov A.F., Novikov M.S. (2015). Domino transformation of isoxazoles to 2,4-dicarbonylpyrroles under Fe/Ni relay catalysis. RSC Adv..

[72] Galenko A.V., Khlebnikov A.F., Novikov M.S., Avdontseva M.S. (2015). Synthesis of 3-(1,2-dioxoethyl)- and 2,3-dicarbonyl-containing pyrroles. Tetrahedron.

[73] Krivolapova Y.V., Tomashenko O.A., Funt L.D., Spiridonova D.V., Novikov M.S., Khlebnikov A.F. (2022). Azirine-triazole hybrids: Selective synthesis of 5-(2*H*-azirin-2-yl)-, 5-(1*H*-pyrrol-2-yl)-1*H*-1,2,3-triazoles and 2-(5-(2*H*-azirin-2-yl)-1*H*-1,2,3-triazol-1-yl)pyridines. Org. Biomol. Chem..

[74] Zanakhov T.O., Galenko E.E., Novikov M.S., Khlebnikov A.F. (2023). Divergent Diazo Approach toward Alkyl 5/4-Hydroxy-3*H*-benzo[*e*]indole-4/5-carboxylates. J. Org. Chem..

[75] Singh B., Ullman E.F. (1967). Photochemical Transposition of Ring Atoms in 3,5-Diarylisoxazoles. An Unusual Example of Wavelength Control in a Photochemical Reaction of Azirines. J. Am. Chem. Soc..

[76] Beccalli E.M., Majori L., Marchesini A., Torricelli C. (1980). Synthesis of Oxazolo-and imidazolo-phanes. Chem. Lett..

[77] Ning Y., Otani Y., Ohwada T. (2017). Base-Induced Transformation of 2-Acyl-3-alkyl-2*H*-azirines to Oxazoles: Involvement of Deprotonation-Initiated Pathways. J. Org. Chem..

[78] Galenko E.E., Bodunov V.A., Taishev A.E., Novikov M.S., Khlebnikov A.F. (2025). Synthesis of Azirinyl Ethynyl Ketones and Their Use in the Preparation C(O)C≡CR-Substituted NH-Pyrroles/NH-Imidazoles and Azole–Azine/Azole–Azole Heterocyclic Hybrids. J. Org. Chem..

[79] Dolomanov O.V., Bourhis L.J., Gildea R.J., Howard J.A.K., Puschmann H. (2009). *OLEX2*: A complete structure solution, refinement and analysis program. J. Appl. Cryst..

[80] Sheldrick G.M. (2015). *SHELXT*—Integrated space-group and crystal-structure determination. Acta Cryst..

[81] Sheldrick G.M. (2015). Crystal Structure Refinement with SHELXL. Acta Cryst..

[82] Frisch M.J., Trucks G.W., Schlegel H.B., Scuseria G.E., Robb M.A., Cheeseman J.R., Scalmani G., Barone V., Petersson G.A., Nakatsuji H. (2016). Gaussian 16.

[83] Becke A.D. (1993). Density-functional thermochemistry. III. The role of exact exchange. J. Chem. Phys..

[84] Becke A.D. (1988). Density-functional exchange-energy approximation with correct asymptotic behavior. Phys. Rev. A.

[85] Lee C., Yang W., Parr R.G. (1988). Development of the Colle-Salvetti correlation-energy formula into a functional of the electron density. Phys. Rev. B.

[86] Grimme S., Antony J., Ehrlich S., Krieg H. (2010). A Consistent and Accurate ab Initio Parametrization of Density Functional Dispersion Correction (DFT-D) for the 94 Elements H-Pu. J. Chem. Phys..

[87] Grimme S., Ehrlich S., Goerigk L. (2011). Effect of the damping function in dispersion corrected density functional theory. J. Comput. Chem..

[88] Marenich A.V., Cramer C.J., Truhlar D.G. (2009). Universal Solvation Model Based on Solute Electron Density and on a Continuum Model of the Solvent Defined by the Bulk Dielectric Constant and Atomic Surface Tensions. J. Phys. Chem. B.

[89] Gonzalez C., Schlegel H.B. (1989). An improved algorithm for reaction path following. J. Chem. Phys..

