# Supplementary material for: Azirinyl-Substituted Nitrile Oxides: Generation and Use in the Synthesis of Isoxazole Containing Heterocyclic Hybrids
Source: Molecules. 2025 Jul 2;30(13):2834. doi: 10.3390/molecules30132834 (PMC12250923; doi:10.3390/molecules30132834)
Supplement: Supplementary file 1 [file molecules-30-02834-s001.zip › SI.pdf]

Supplementary Materials for

Azirinyl-substituted nitrile oxides: generation and use in the synthesis isoxazole containing heterocyclic hybrids

Alexander S. Dudik, Timur O. Zanakhov, Ekaterina E. Galenko, Mikhail S. Novikov, and Alexander F. Khlebnikov*

Saint Petersburg State University, Institute of Chemistry, 7/9 Universitetskaya Naberezhnaya, St. Petersburg, 199034 Russia

e-mail: a.khlebnikov@spbu.ru

Table of Contents

X-Ray Diffraction Experiments S2

NMR Spectra of Compounds **3** S13

NMR Spectra of Compounds **4** S82

NMR Spectra of Compound **8c** S154

NMR Spectra of Compounds **5** S157

NMR Spectra of Compounds **6** S178

Computational details S193

X-RAY DIFFRACTION EXPERIMENTS

Crystal structures of 3g and 4a were determined by single crystal X-ray diffraction analysis. Suitable crystals were selected and fixed on micro-amounts and the diffraction data were collected on diffractometer. The crystals 3g and 4a were measured at temperature 100 K, using monochromated CuKα radiation. The unit cell parameters and refinement characteristics of the crystal structures of 3g and 4a are given below. Using Olex2[1], the structure was solved with the ShelXT [2] structure solution program using Intrinsic Phasing and refined with the ShelXL [3] refinement package using Least Squares minimization.

References

1. Dolomanov, O. V.; Bourhis, L. J.; Gildea, R. J.; Howard, J .A. K.; Puschmann, H. J. Appl. Cryst. 2009,

42, 339.

2. Sheldrick, G. M. Acta Cryst. 2015, A71, 3.

3. Sheldrick, G. M. Acta Cryst. 2015, C71, 3.

(5-(Chloromethyl)isoxazol-3-yl)(3-phenyl-2*H*-azirin-2-yl)methanoneole 3g

Single crystals of **3g** were obtained by slow recrystallization from acetonitrile at room temperature (CCDC 2455839)

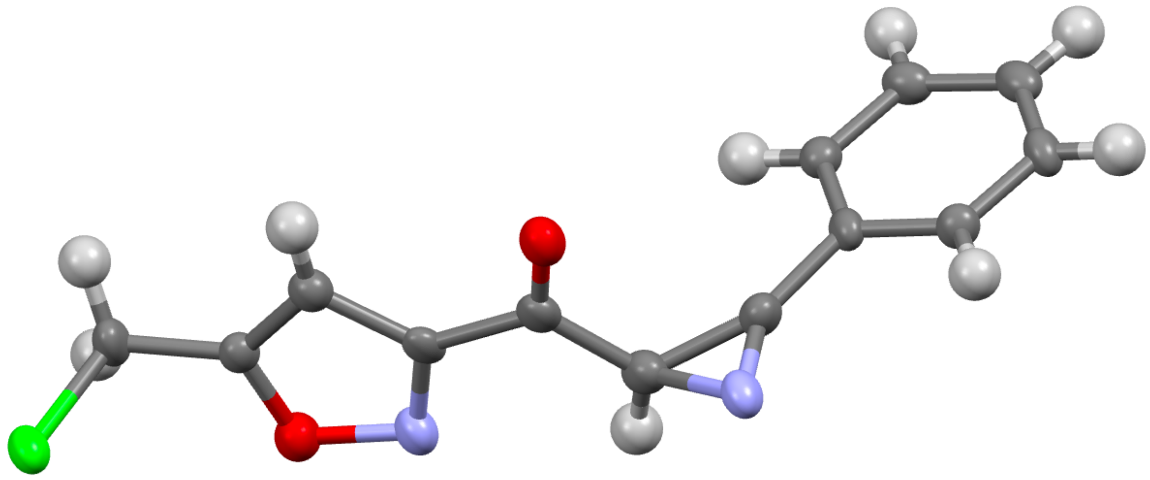

Figure S1. Molecular structure of compound 3g, displacement parameters are drawn at 50% probability level.

Table S1. Crystal data and structure refinement for 3g.

Identification code	3g (HB_2)
Empirical formula	$C_{13}H_9ClN_2O_2$
Formula weight	260.67
Temperature/K	100.00(10)
Crystal system	tetragonal
Space group	P-4$2_1$c
a/Å	15.3451(2)
b/Å	15.3451(2)
c/Å	10.2061(2)
α/°	90
β/°	90
γ/°	90
Volume/Å^3	2403.25(8)
Z	8
ρ_{calc}g/cm^3	1.441
μ/mm^{-1}	2.786
F(000)	1072.0
Crystal size/mm^3	0.1 × 0.06 × 0.04
Radiation	Cu Kα (λ = 1.54184)
2Θ range for data collection/°	8.148 to 141.286
Index ranges	-18 ≤ h ≤ 18, -10 ≤ k ≤ 18, -12 ≤ l ≤ 12
Reflections collected	12417
Independent reflections	2303 [R_{int} = 0.0772, R_{sigma} = 0.0408]
Data/restraints/parameters	2303/0/157
Goodness-of-fit on F^2	1.058
Final R indexes [I>=2σ (I)]	R_1 = 0.0554, wR_2 = 0.1362
Final R indexes [all data]	R_1 = 0.0571, wR_2 = 0.1373
Largest diff. peak/hole / e Å^{-3}	0.51/-0.29
Flack parameter	0.03(2)

Table S2. Fractional Atomic Coordinates (×10^4) and Equivalent Isotropic Displacement Parameters (Å^2×10^3) for 3g. U_{eq} is defined as 1/3 of the trace of the orthogonalised U_{IJ} tensor.

Atom	*x*	*y*	*z*	U(eq)
Cl1	4662.6(8)	7733.1(8)	-1521.9(13)	30.2(3)
O1	5142(3)	6292(3)	828(4)	33.2(9)
O2	7172(2)	7411(2)	3513(4)	32.0(8)
N1	5423(3)	6239(3)	2121(5)	31.5(10)
N2	6421(3)	6791(3)	5916(5)	30.2(3)
C4	6109(3)	6741(4)	2181(5)	24.7(11)
C2	5685(3)	6818(3)	163(5)	24.5(11)
C3	6316(4)	7153(4)	935(5)	28.3(11)
C7	6960(4)	6210(4)	5642(5)	30.7(12)
C8	7692(3)	5720(3)	6154(5)	24.1(10)
C10	8729(4)	4560(4)	5911(6)	29.6(11)
C11	8981(3)	4675(4)	7207(6)	29.6(12)
C5	6592(3)	6873(4)	3439(6)	28.5(11)
C13	7959(3)	5847(3)	7451(5)	24.8(11)
C9	8080(3)	5086(4)	5379(5)	25.6(11)
C12	8602(3)	5322(3)	7967(5)	27.4(11)
C6	6324(4)	6315(4)	4553(6)	34.3(13)
C1	5509(4)	6927(4)	-1254(5)	33.9(14)

Table S3. Anisotropic Displacement Parameters (Å^2×10^3) for 3g. The Anisotropic displacement factor exponent takes the form: $-2\pi^2[h^2a^{*2}U_{11}+2hka^*b^*U_{12}+\ldots]$.

Atom	U_{11}	U_{22}	U_{33}	U_{23}	U_{13}	U_{12}
Cl1	28.9(6)	32.5(6)	29.4(5)	4.5(5)	-7.0(5)	-4.2(5)
O1	33(2)	37(2)	30(2)	0.2(18)	-1.8(17)	-0.7(17)
O2	33(2)	32.7(19)	30.7(17)	-0.9(17)	-3.2(18)	3.2(16)
N1	30(2)	33(3)	31(2)	3(2)	-5(2)	3(2)
N2	28.9(6)	32.5(6)	29.4(5)	4.5(5)	-7.0(5)	-4.2(5)
C4	19(2)	30(3)	25(2)	-6(2)	0(2)	8(2)
C2	24(2)	27(3)	22(2)	-2(2)	1(2)	4(2)
C3	26(3)	30(3)	29(3)	0(2)	0(2)	3(2)
C7	30(3)	38(3)	24(2)	2(2)	4(2)	5(2)
C8	24(2)	25(2)	23(2)	7.4(19)	-3(2)	-2(2)
C10	26(3)	24(3)	38(3)	1(2)	2(2)	1(2)
C11	23(3)	28(3)	38(3)	9(2)	-1(2)	-2(2)
C5	22(3)	34(3)	29(2)	0(2)	1(2)	9(2)
C13	29(3)	19(2)	26(2)	-2(2)	1(2)	2(2)
C9	25(3)	27(3)	25(2)	1(2)	1(2)	-2(2)
C12	28(3)	26(2)	28(2)	4(2)	-9(2)	-3(2)
C6	28(3)	39(3)	35(3)	8(3)	2(2)	5(2)
C1	32(3)	48(3)	21(3)	0(2)	-4(2)	9(3)

Table S4 Bond Lengths for 3g.

Atom	Atom	Length/Å	Atom	Atom	Length/Å
Cl1	C1	1.814(6)	C2	C1	1.481(7)
O1	N1	1.391(6)	C7	C8	1.449(7)
O1	C2	1.344(7)	C7	C6	1.489(8)
O2	C5	1.216(7)	C8	C13	1.398(7)
N1	C4	1.305(8)	C8	C9	1.389(7)
N2	C7	1.249(8)	C10	C11	1.389(8)
N2	C6	1.579(8)	C10	C9	1.392(8)
C4	C3	1.455(7)	C11	C12	1.388(8)
C4	C5	1.497(7)	C5	C6	1.482(8)
C2	C3	1.350(8)	C13	C12	1.379(7)

Table S5. Bond Angles for 3g.

Atom	Atom	Atom	Angle/°	Atom	Atom	Atom	Angle/°
C2	O1	N1	108.7(4)	C9	C8	C7	119.4(5)
C4	N1	O1	105.1(5)	C9	C8	C13	120.8(5)
C7	N2	C6	62.3(4)	C11	C10	C9	119.8(5)
N1	C4	C3	113.0(5)	C12	C11	C10	120.4(5)
N1	C4	C5	121.3(5)	O2	C5	C4	120.5(5)
C3	C4	C5	125.7(5)	O2	C5	C6	123.3(5)
O1	C2	C3	112.3(5)	C6	C5	C4	116.2(5)
O1	C2	C1	116.6(5)	C12	C13	C8	119.3(5)
C3	C2	C1	131.1(5)	C8	C9	C10	119.4(5)
C2	C3	C4	100.8(5)	C13	C12	C11	120.3(5)
N2	C7	C8	143.5(5)	C7	C6	N2	47.9(3)
N2	C7	C6	69.8(4)	C5	C6	N2	112.5(5)
C8	C7	C6	146.5(5)	C5	C6	C7	116.9(5)
C13	C8	C7	119.7(5)	C2	C1	Cl1	110.8(4)

Table S6. Torsion Angles for 3g.

A	B	C	D	Angle/°	A	B	C	D	Angle/°
O1	N1	C4	C3	0.1(6)	C3	C4	C5	C6	-173.7(5)
O1	N1	C4	C5	178.6(4)	C3	C2	C1	Cl1	-98.8(7)
O1	C2	C3	C4	1.6(6)	C7	N2	C6	C5	-106.7(5)
O1	C2	C1	Cl1	81.9(6)	C7	C8	C13	C12	174.0(5)
O2	C5	C6	N2	33.1(7)	C7	C8	C9	C10	-174.1(5)
O2	C5	C6	C7	-19.8(8)	C8	C7	C6	N2	175.1(11)
N1	O1	C2	C3	-1.7(6)	C8	C7	C6	C5	-88.1(11)
N1	O1	C2	C1	177.7(5)	C8	C13	C12	C11	0.1(8)
N1	C4	C3	C2	-1.1(6)	C10	C11	C12	C13	1.2(8)
N1	C4	C5	O2	-172.3(5)	C11	C10	C9	C8	-0.1(8)
N1	C4	C5	C6	8.0(7)	C5	C4	C3	C2	-179.5(5)
N2	C7	C8	C13	3.4(12)	C13	C8	C9	C10	1.5(8)
N2	C7	C8	C9	179.0(8)	C9	C8	C13	C12	-1.5(8)
N2	C7	C6	C5	96.8(6)	C9	C10	C11	C12	-1.2(8)
C4	C5	C6	N2	-147.2(4)	C6	N2	C7	C8	-175.4(10)
C4	C5	C6	C7	159.9(5)	C6	C7	C8	C13	-168.8(8)
C2	O1	N1	C4	0.9(5)	C6	C7	C8	C9	6.8(12)
C3	C4	C5	O2	6.0(8)	C1	C2	C3	C4	-177.6(6)

Table S7. Hydrogen Atom Coordinates (Å×10^4) and Isotropic Displacement Parameters (Å^2×10^3) for 3g.

Atom	*x*	*y*	*z*	U(eq)
H3	6769.8	7549.77	722.22	34
H10	8998.4	4123.62	5389.62	35
H11	9415.9	4308.25	7574.82	36
H13	7699.84	6291.25	7969.68	30
H9	7904.94	5011.94	4492.8	31
H12	8786.51	5403.18	8846.94	33
H6	5852.99	5872.14	4427.35	41
H1A	5324.14	6362.41	-1632.56	41
H1B	6048.99	7112.64	-1704.86	41

Methyl 3'-phenyl-[3,5'-biisoxazole]-5-carboxylate 4a

Single crystals of **4a** were obtained by slow recrystallization from acetonitrile at room temperature (CCDC 2455843)

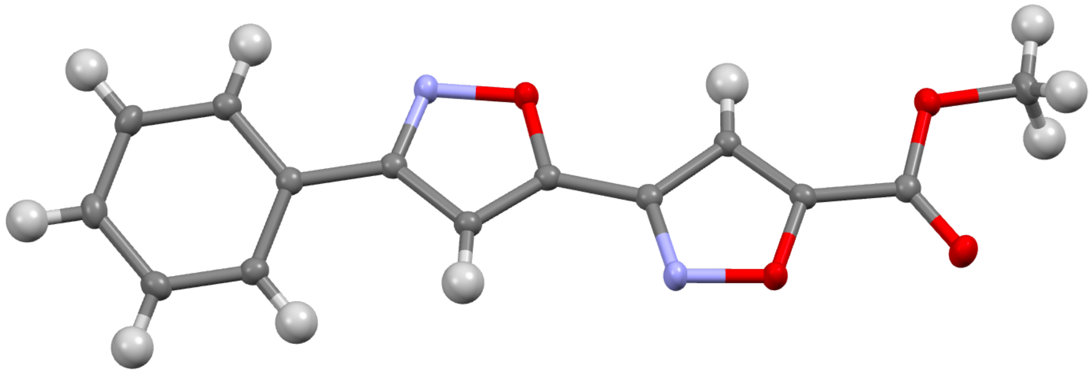

Figure S2. Molecular structure of compound 4a, displacement parameters are drawn at 50% probability level.

Table S8. Crystal data and structure refinement for 4a.

Identification code	4a (HB_1)
Empirical formula	$C_{14}H_{10}N_2O_4$
Formula weight	270.24
Temperature/K	99.99(10)
Crystal system	monoclinic
Space group	$P2_1/c$
a/Å	13.5255(3)
b/Å	5.73100(10)
c/Å	15.3411(3)
α/°	90
β/°	92.936(2)
γ/°	90
Volume/Å^3	1187.60(4)
Z	4
ρ_{calc}g/cm^3	1.511
μ/mm^{-1}	0.952
F(000)	560.0
Crystal size/mm^3	0.16 × 0.14 × 0.1
Radiation	Cu Kα (λ = 1.54184)
2Θ range for data collection/°	6.544 to 144.862
Index ranges	$-15 \le h \le 16, -6 \le k \le 7, -18 \le l \le 18$
Reflections collected	7552
Independent reflections	2328 [R_{int} = 0.0244, R_{sigma} = 0.0234]
Data/restraints/parameters	2328/0/182
Goodness-of-fit on F^2	1.090
Final R indexes [I>=2σ (I)]	R_1 = 0.0349, wR_2 = 0.0945
Final R indexes [all data]	R_1 = 0.0369, wR_2 = 0.0969
Largest diff. peak/hole / e Å^{-3}	0.36/-0.23

Table S9. Fractional Atomic Coordinates (×10^4) and Equivalent Isotropic Displacement Parameters (Å^2×10^3) for 4a. U_{eq} is defined as 1/3 of the trace of the orthogonalised U_{IJ} tensor.

Atom	*x*	*y*	*z*	U(eq)
O1	5389.8(6)	3532.9(14)	6218.6(5)	17.5(2)
O2	3147.3(6)	8687.3(14)	7172.7(5)	18.4(2)
O4	1901.0(6)	3943.5(14)	8084.1(5)	19.6(2)
O3	1361.9(6)	7640.5(15)	7870.2(5)	22.3(2)
N1	6245.6(7)	3251.1(17)	5748.8(6)	18.0(2)
N2	4026.6(7)	8674.1(17)	6733.4(6)	18.7(2)
C7	6504.7(8)	5369.5(19)	5513.7(7)	14.7(2)
C8	5843.1(8)	7087.6(19)	5812.4(7)	15.9(2)
C10	4291.3(8)	6465.1(19)	6696.1(7)	15.2(2)
C9	5174.1(8)	5841.7(19)	6241.5(7)	14.9(2)
C12	2927.2(8)	6459.9(19)	7381.8(7)	15.3(2)
C6	7384.4(8)	5708.7(19)	4999.2(7)	15.2(2)
C11	3618.9(7)	4970.9(19)	7106.5(7)	14.9(2)
C5	7519.8(8)	7801(2)	4553.7(7)	17.6(2)
C1	8084.0(8)	3933(2)	4942.5(7)	17.7(2)
C13	1975.8(8)	6133(2)	7806.6(7)	16.2(2)
C4	8345.1(8)	8103(2)	4060.1(7)	19.4(2)
C3	9043.5(8)	6342(2)	4014.4(7)	20.0(3)
C2	8908.4(8)	4250(2)	4455.2(7)	20.1(3)
C14	963.2(8)	3345(2)	8438.3(8)	21.9(3)

Table S10. Anisotropic Displacement Parameters (Å^2×10^3) for 4a. The Anisotropic displacement factor exponent takes the form: $-2\pi^2[h^2a^{*2}U_{11}+2hka^*b^*U_{12}+\ldots]$.

Atom	U_{11}	U_{22}	U_{33}	U_{23}	U_{13}	U_{12}
O1	17.4(4)	14.4(4)	21.2(4)	0.8(3)	6.2(3)	0.3(3)
O2	16.6(4)	14.8(4)	24.2(4)	1.7(3)	6.2(3)	1.0(3)
O4	16.6(4)	18.6(4)	23.9(4)	4.3(3)	5.1(3)	0.0(3)
O3	19.5(4)	19.6(5)	28.3(5)	-0.9(3)	5.6(3)	2.7(3)
N1	16.7(5)	17.1(5)	20.7(5)	0.6(4)	5.7(4)	1.0(4)
N2	16.2(5)	17.6(5)	23.0(5)	1.6(4)	6.6(4)	0.5(4)
C7	15.6(5)	14.3(5)	14.1(5)	-0.5(4)	-0.5(4)	0.2(4)
C8	16.8(5)	13.4(5)	17.6(5)	0.7(4)	1.2(4)	0.1(4)
C10	15.2(5)	15.4(5)	14.9(5)	0.1(4)	-0.5(4)	-0.3(4)
C9	15.4(5)	13.9(5)	15.4(5)	-0.3(4)	-0.3(4)	0.2(4)
C12	16.3(5)	14.2(5)	15.3(5)	1.5(4)	0.7(4)	-1.6(4)
C6	15.8(5)	15.7(5)	14.0(5)	-1.9(4)	-0.3(4)	-1.0(4)
C11	15.8(5)	14.1(5)	14.9(5)	0.2(4)	0.4(4)	-0.9(4)
C5	17.8(5)	15.7(5)	19.2(5)	-0.2(4)	0.1(4)	0.8(4)
C1	19.9(5)	15.5(5)	17.7(5)	-0.3(4)	0.8(4)	0.6(4)
C13	16.3(5)	17.0(5)	15.2(5)	-1.0(4)	0.4(4)	-0.4(4)
C4	21.1(6)	17.9(5)	19.1(5)	1.5(4)	1.0(4)	-2.8(4)
C3	18.1(5)	24.6(6)	17.6(5)	-3.4(4)	3.2(4)	-2.5(4)
C2	18.1(5)	20.4(6)	21.9(5)	-3.5(5)	1.9(4)	3.4(4)
C14	18.0(6)	24.4(6)	23.9(6)	3.6(5)	4.6(4)	-3.6(5)

Table S11 Bond Lengths for 4a.

Atom	Atom	Length/Å	Atom	Atom	Length/Å
O1	N1	1.4038(11)	C8	C9	1.3502(15)
O1	C9	1.3558(13)	C10	C9	1.4578(15)
O2	N2	1.3968(12)	C10	C11	1.4197(15)
O2	C12	1.3531(13)	C12	C11	1.3499(15)
O4	C13	1.3306(14)	C12	C13	1.4838(15)
O4	C14	1.4466(13)	C6	C5	1.3967(15)
O3	C13	1.2057(14)	C6	C1	1.3954(16)
N1	C7	1.3190(15)	C5	C4	1.3914(15)
N2	C10	1.3176(15)	C1	C2	1.3858(16)
C7	C8	1.4221(15)	C4	C3	1.3865(17)
C7	C6	1.4741(14)	C3	C2	1.3931(17)

Table S12. Bond Angles for 4a.

Atom	Atom	Atom	Angle/˚	Atom	Atom	Atom	Angle/˚
C9	O1	N1	108.05(8)	O2	C12	C13	115.44(9)
C12	O2	N2	108.20(8)	C11	C12	O2	110.97(9)
C13	O4	C14	115.24(9)	C11	C12	C13	133.48(10)
C7	N1	O1	105.79(8)	C5	C6	C7	120.32(10)
C10	N2	O2	105.43(9)	C1	C6	C7	120.37(10)
N1	C7	C8	111.65(9)	C1	C6	C5	119.30(10)
N1	C7	C6	120.06(10)	C12	C11	C10	103.15(10)
C8	C7	C6	128.29(10)	C4	C5	C6	120.14(10)
C9	C8	C7	103.85(10)	C2	C1	C6	120.31(10)
N2	C10	C9	119.16(10)	O4	C13	C12	109.96(9)
N2	C10	C11	112.25(10)	O3	C13	O4	125.86(10)
C11	C10	C9	128.56(10)	O3	C13	C12	124.16(10)
O1	C9	C10	115.74(9)	C3	C4	C5	120.30(11)
C8	C9	O1	110.67(9)	C4	C3	C2	119.68(10)
C8	C9	C10	133.59(11)	C1	C2	C3	120.27(11)

Table S13. Torsion Angles for 4a.

A	B	C	D	Angle/°	A	B	C	D	Angle/°
O1	N1	C7	C8	-0.07(12)	C7	C6	C1	C2	179.70(10)
O1	N1	C7	C6	-179.71(8)	C8	C7	C6	C5	-16.60(16)
O2	N2	C10	C9	177.67(9)	C8	C7	C6	C1	164.24(11)
O2	N2	C10	C11	-0.55(12)	C9	O1	N1	C7	0.07(11)
O2	C12	C11	C10	-0.56(11)	C9	C10	C11	C12	-177.31(11)
O2	C12	C13	O4	-171.82(9)	C12	O2	N2	C10	0.18(11)
O2	C12	C13	O3	9.43(16)	C6	C7	C8	C9	179.65(10)
N1	O1	C9	C8	-0.04(12)	C6	C5	C4	C3	-0.70(17)
N1	O1	C9	C10	179.14(8)	C6	C1	C2	C3	-0.28(17)
N1	C7	C8	C9	0.05(12)	C11	C10	C9	O1	-1.08(16)
N1	C7	C6	C5	162.97(10)	C11	C10	C9	C8	177.86(11)
N1	C7	C6	C1	-16.19(15)	C11	C12	C13	O4	12.53(17)
N2	O2	C12	C11	0.26(12)	C11	C12	C13	O3	-166.22(12)
N2	O2	C12	C13	-176.35(8)	C5	C6	C1	C2	0.53(16)
N2	C10	C9	O1	-178.97(10)	C5	C4	C3	C2	0.95(17)
N2	C10	C9	C8	-0.03(18)	C1	C6	C5	C4	-0.04(16)
N2	C10	C11	C12	0.70(12)	C13	C12	C11	C10	175.23(11)
C7	C8	C9	O1	0.00(12)	C4	C3	C2	C1	-0.46(17)
C7	C8	C9	C10	-178.98(11)	C14	O4	C13	O3	4.25(16)
C7	C6	C5	C4	-179.21(10)	C14	O4	C13	C12	-174.47(9)

Table S14. Hydrogen Atom Coordinates (Å×10^4) and Isotropic Displacement Parameters (Å^2×10^3) for 4a.

Atom	*x*	*y*	*z*	U(eq)
H8	5866.38	8729.01	5729.3	19
H11	3648.48	3323.56	7171.61	18
H5	7047.45	9019.93	4587.88	21
H1	7994.72	2499.35	5239.36	21
H4	8430.41	9522.3	3752.95	23
H3	9611.47	6561.12	3684.13	24
H2	9383.21	3036.32	4421.6	24
H14A	424.13	3708.24	8008.63	33
H14B	876.7	4245.4	8972.01	33
H14C	953.51	1674.11	8574.29	33

Methyl 3-(3-phenyl-2*H*-azirine-2-carbonyl)isoxazole-5-carboxylate (3a), ^{1}H NMR, $CDCl_3$, 400 MHz

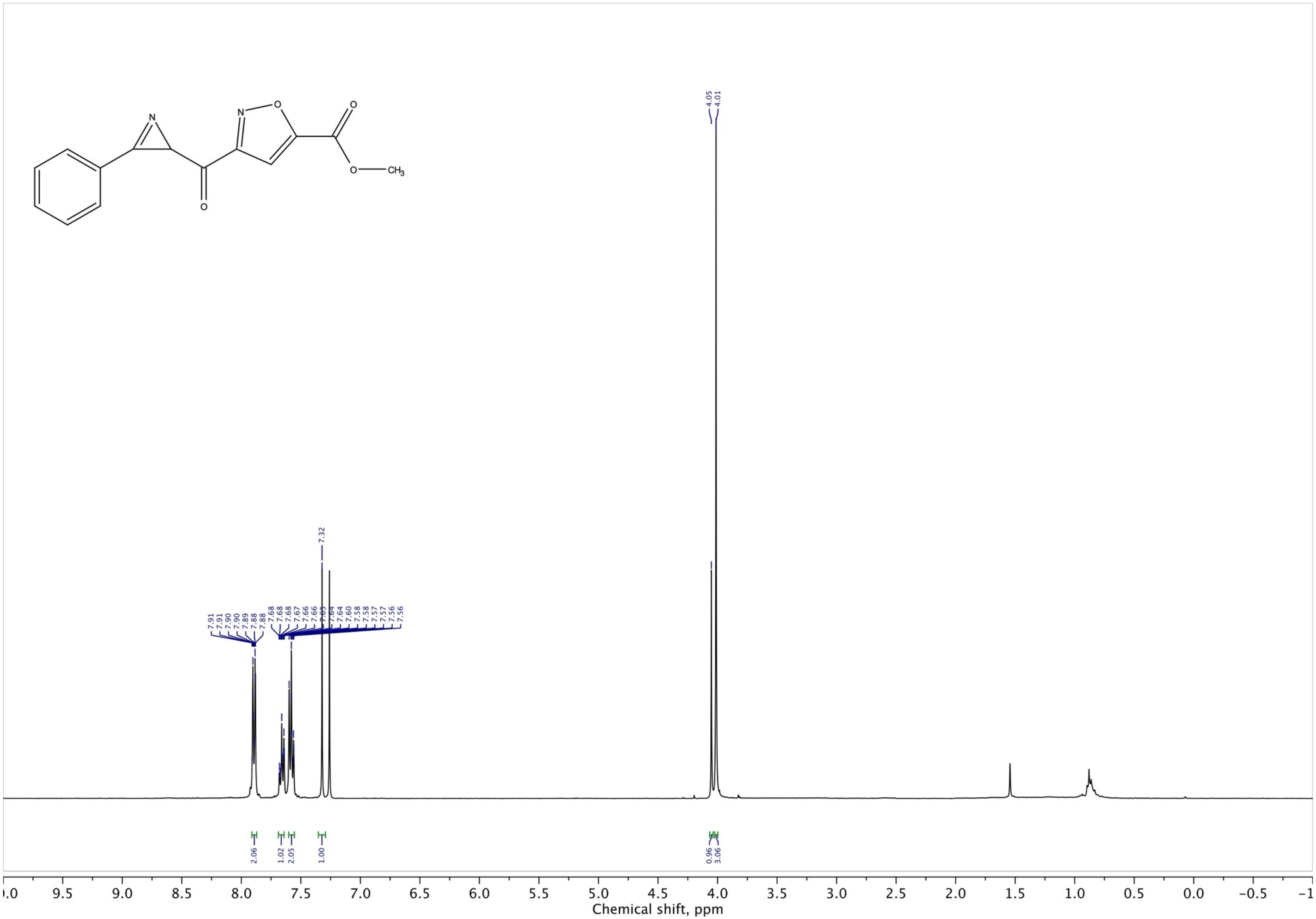

Methyl 3-(3-phenyl-2*H*-azirine-2-carbonyl)isoxazole-5-carboxylate (3a), $^{13}C\{^{1}H\}$ NMR, $CDCl_3$, 100 MHz

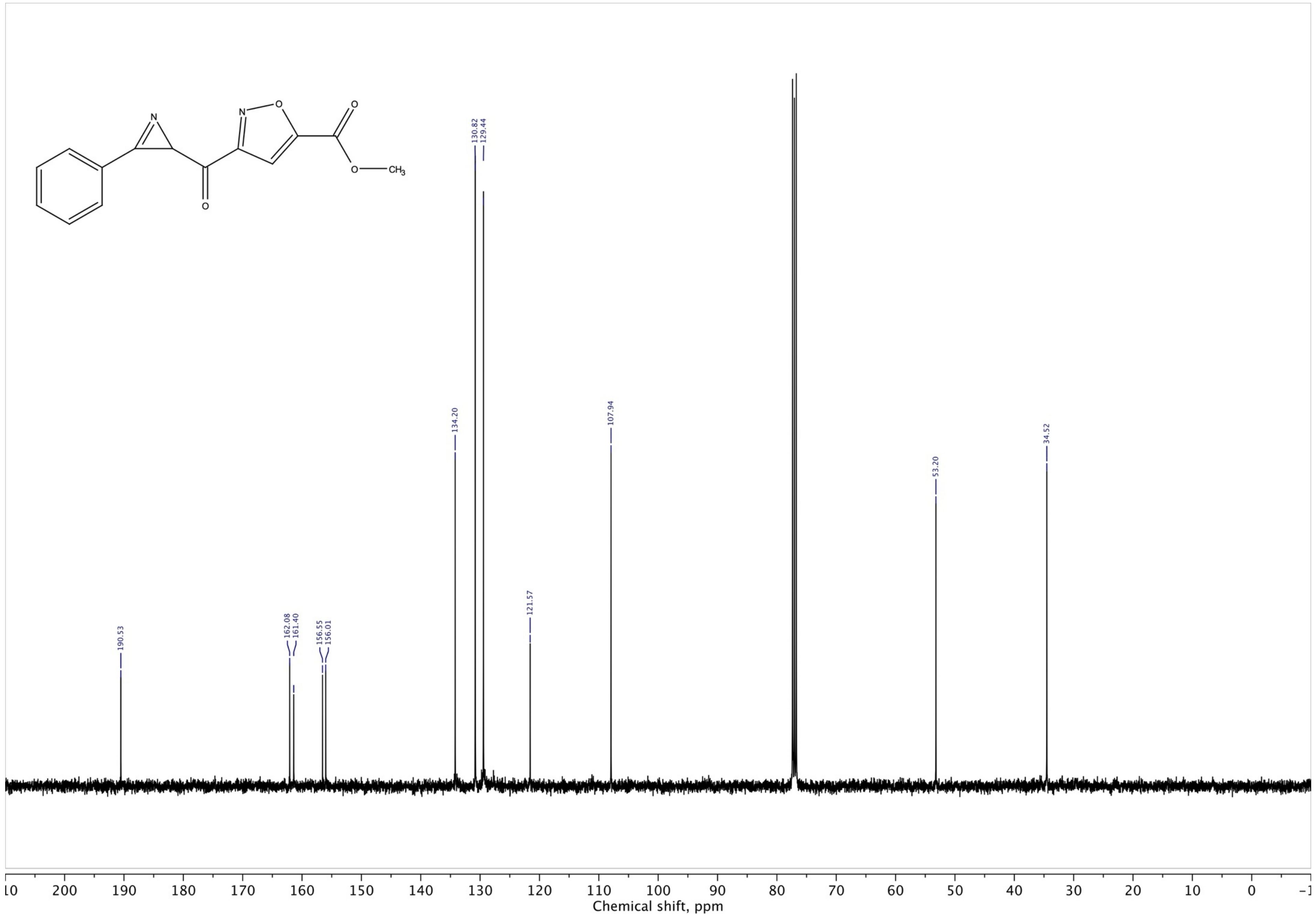

Methyl 3-(3-phenyl-2*H*-azirine-2-carbonyl)isoxazole-5-carboxylate (3a), DEPT, $CDCl_3$, 100 MHz

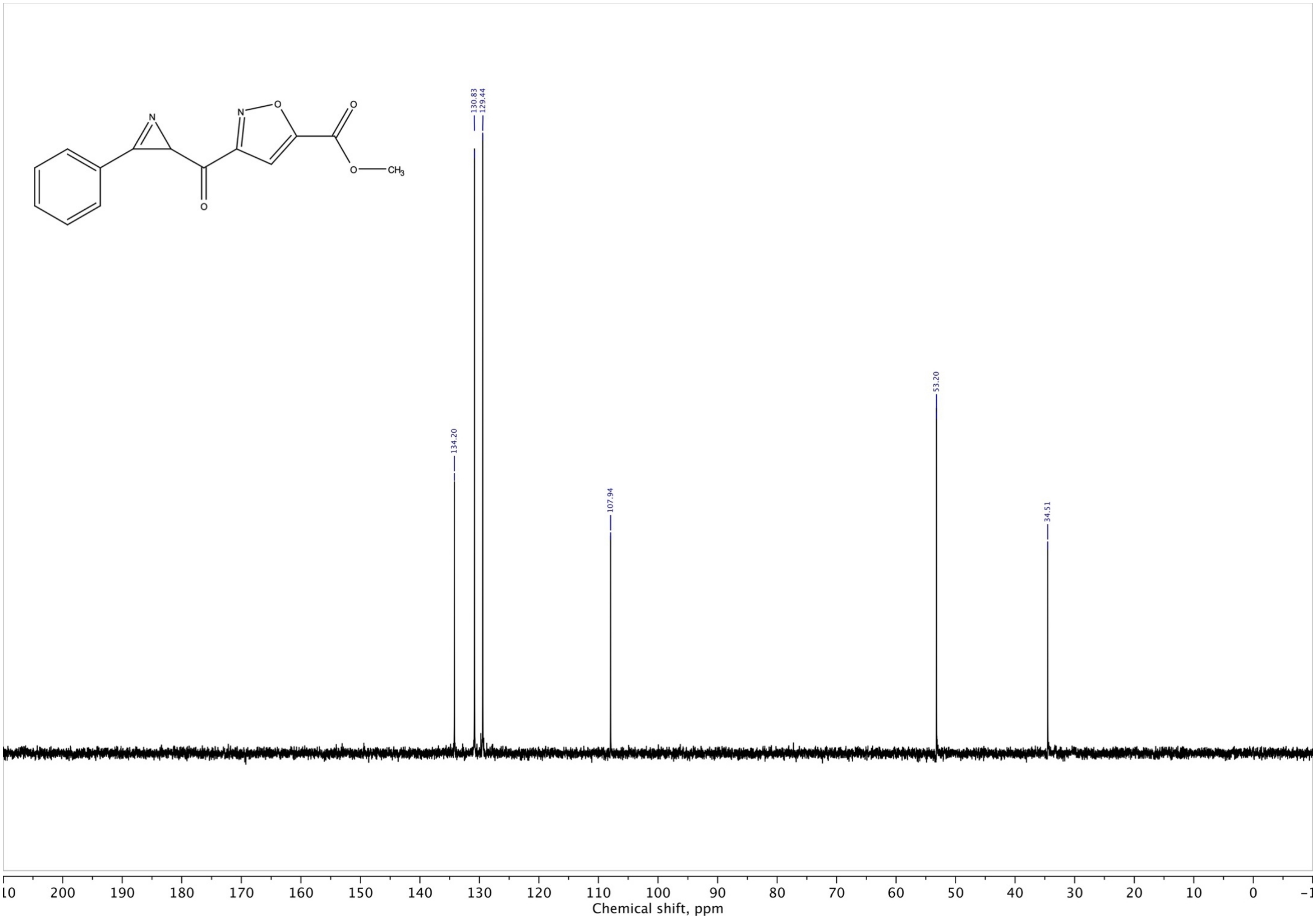

2-((3-(3-Phenyl-2*H*-azirine-2-carbonyl)isoxazol-5-yl)methyl)isoindoline-1,3-dione (3b), ^{1}H NMR, $CDCl_3$, 400 MHz

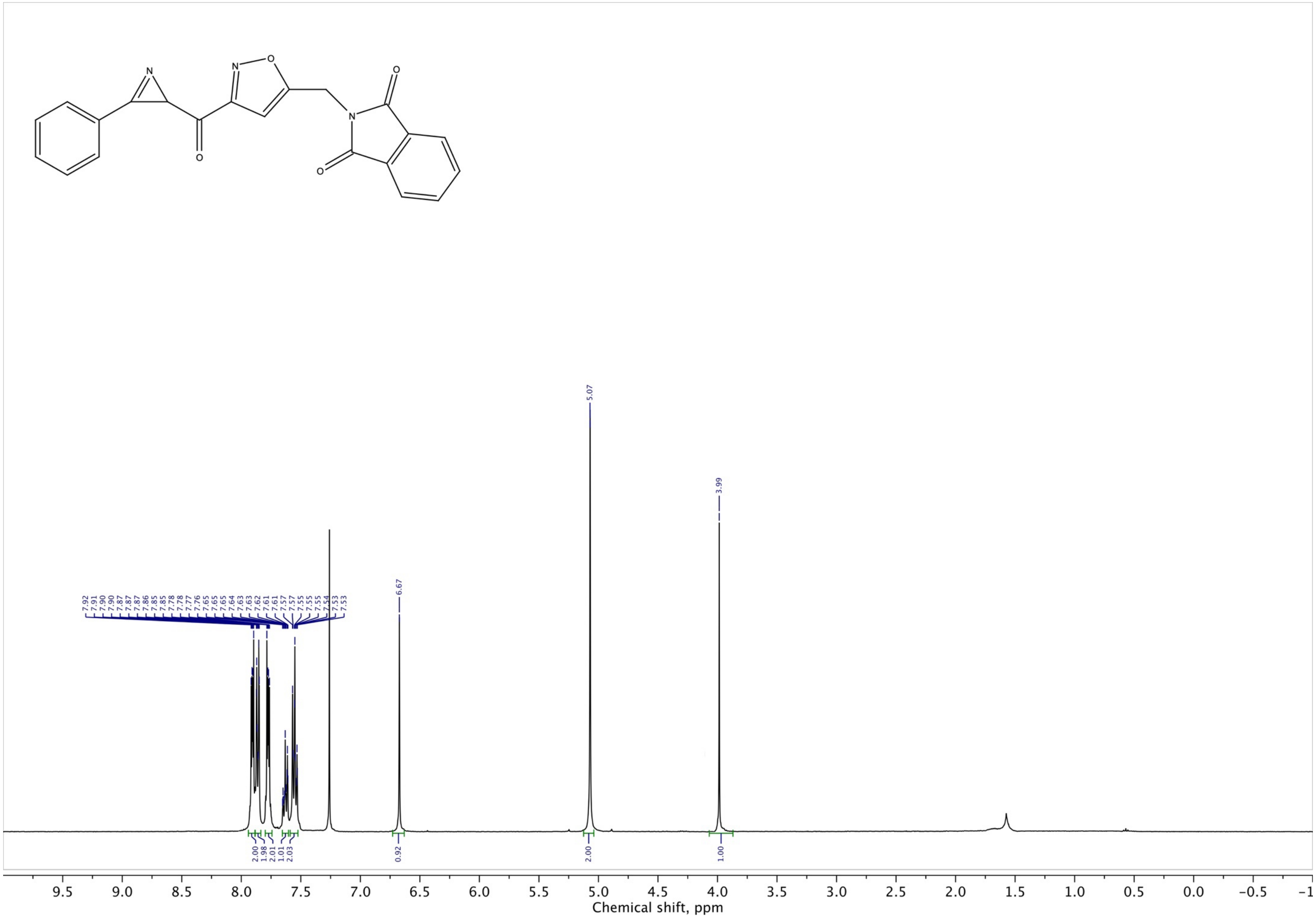

2-((3-(3-Phenyl-2*H*-azirine-2-carbonyl)isoxazol-5-yl)methyl)isoindoline-1,3-dione (3b), $^{13}C\{^1H\}$ NMR, $CDCl_3$, 100 MHz

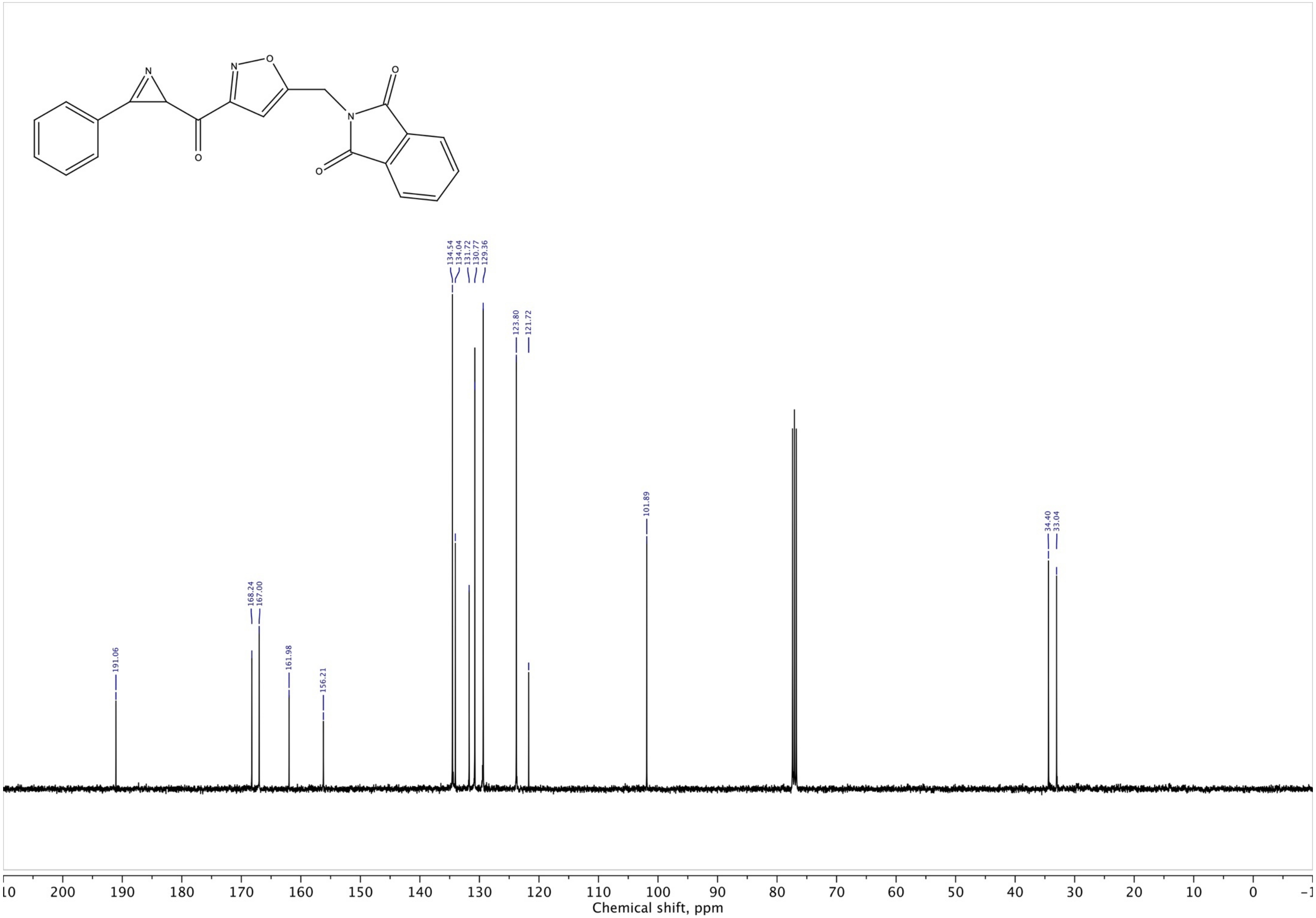

2-((3-(3-Phenyl-2*H*-azirine-2-carbonyl)isoxazol-5-yl)methyl)isoindoline-1,3-dione (3b), DEPT, $CDCl_3$, 100 MHz

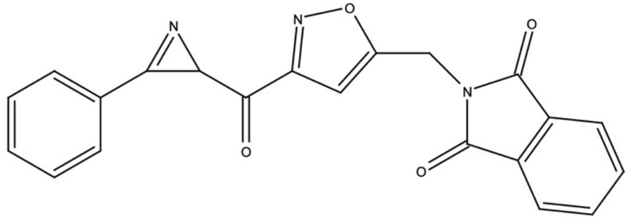

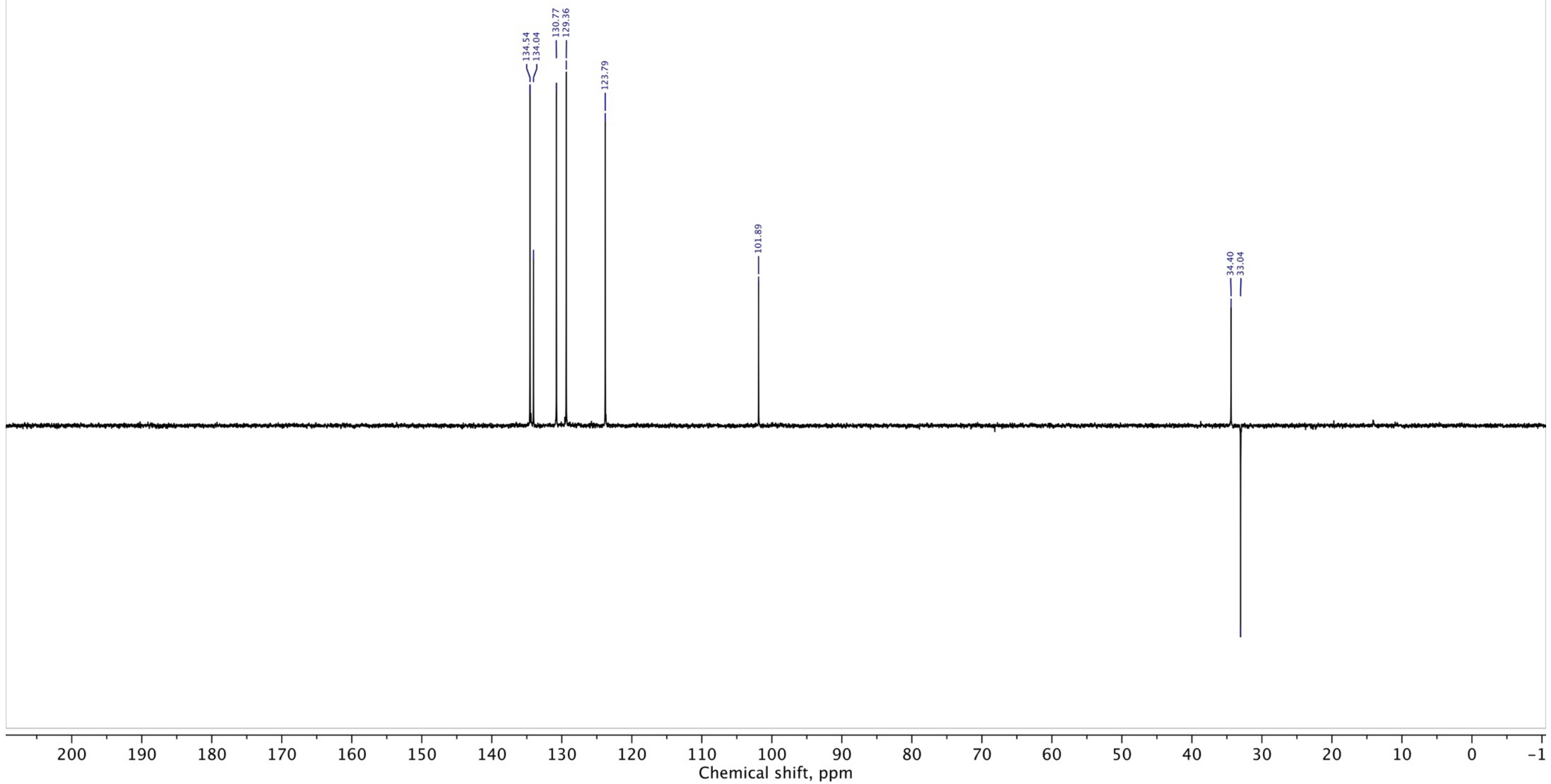

(5-(Hydroxymethyl)isoxazol-3-yl)(3-phenyl-2*H*-azirin-2-yl)methanone (3c), ^{1}H NMR, $CDCl_3$, 400 MHz

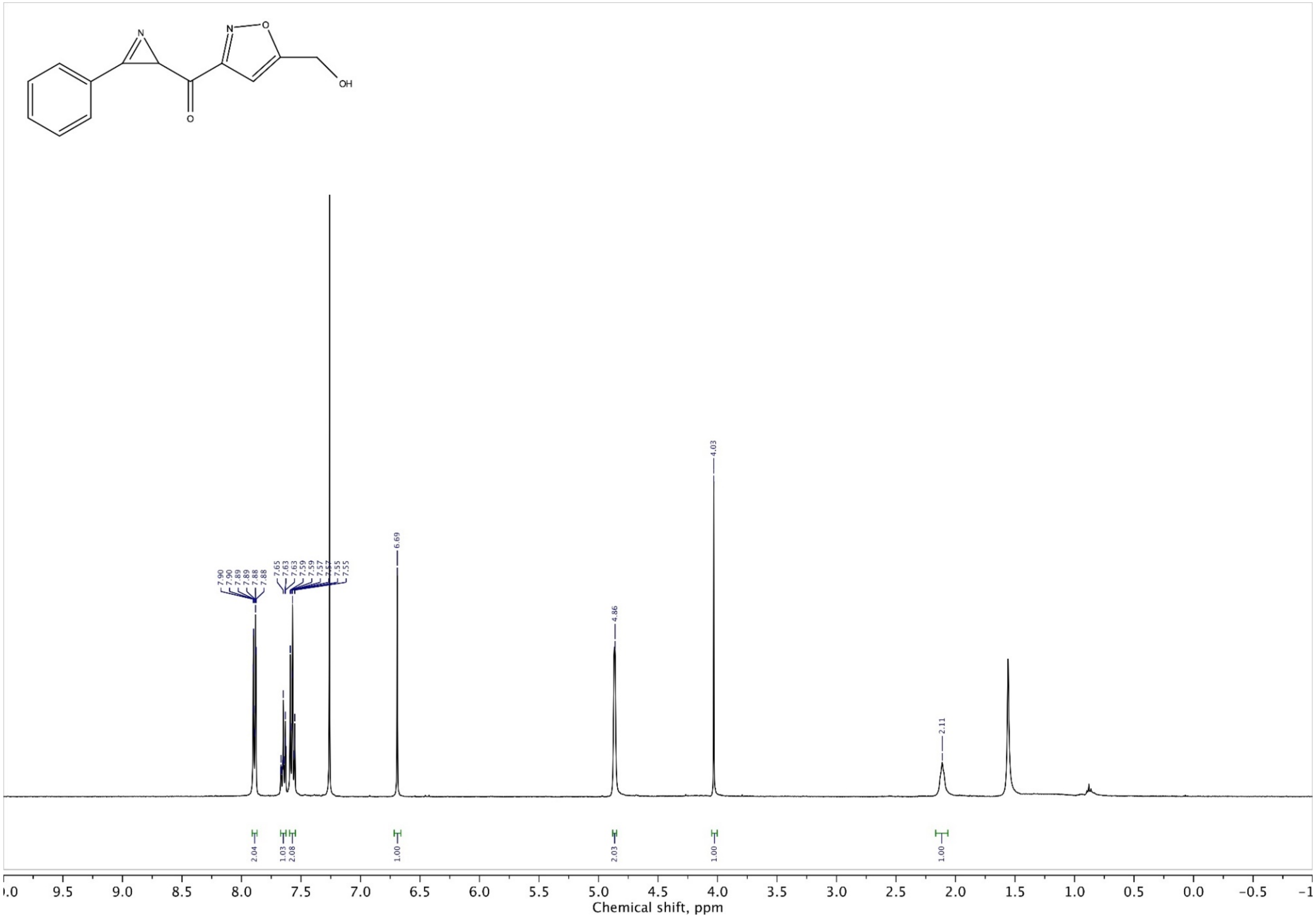

(5-(Hydroxymethyl)isoxazol-3-yl)(3-phenyl-2*H*-azirin-2-yl)methanone (3c), $^{13}C\{^1H\}$ NMR, $CDCl_3$, 100 MHz

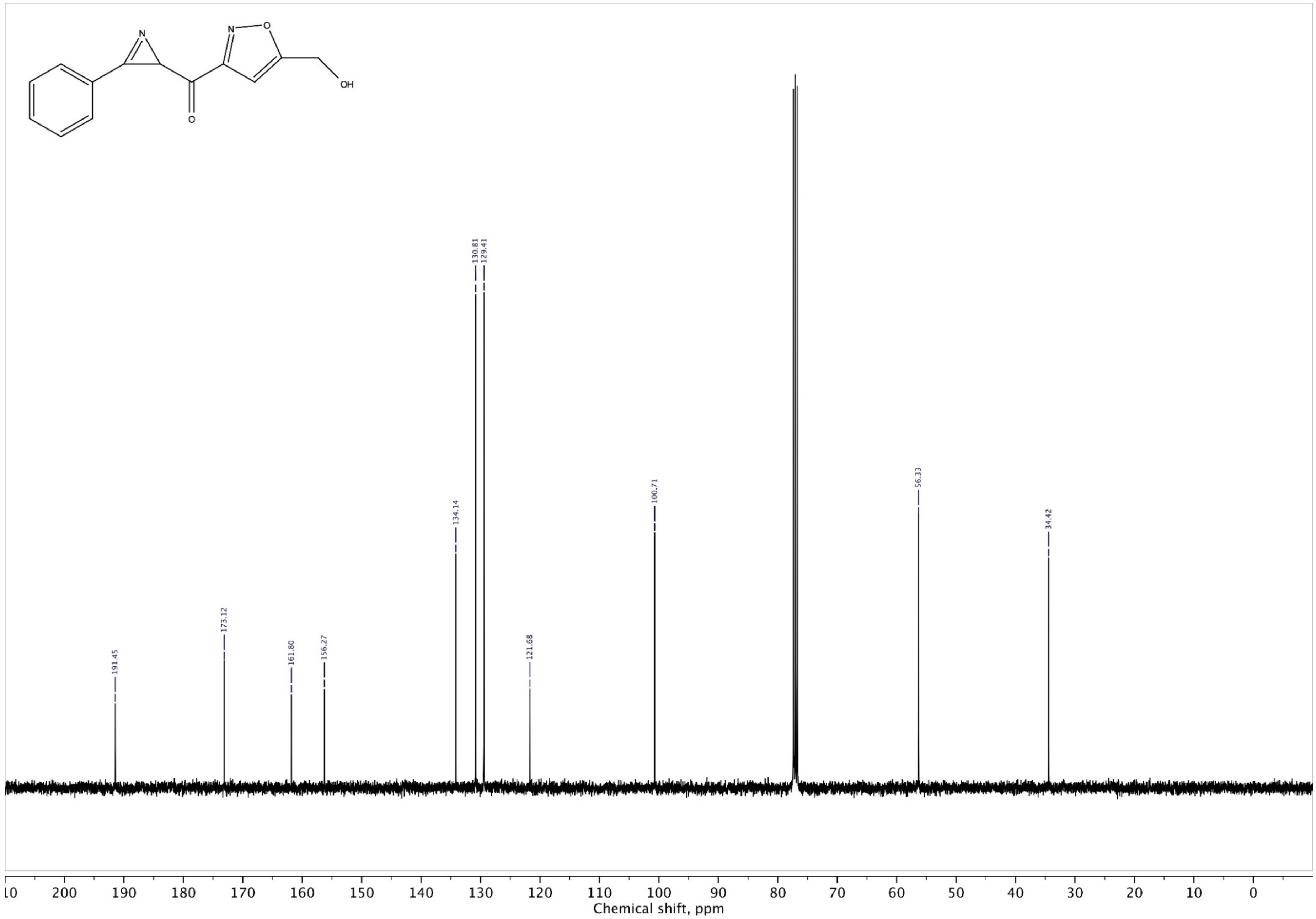

(5-(Hydroxymethyl)isoxazol-3-yl)(3-phenyl-2*H*-azirin-2-yl)methanone (3c), DEPT, $CDCl_3$, 100 MHz

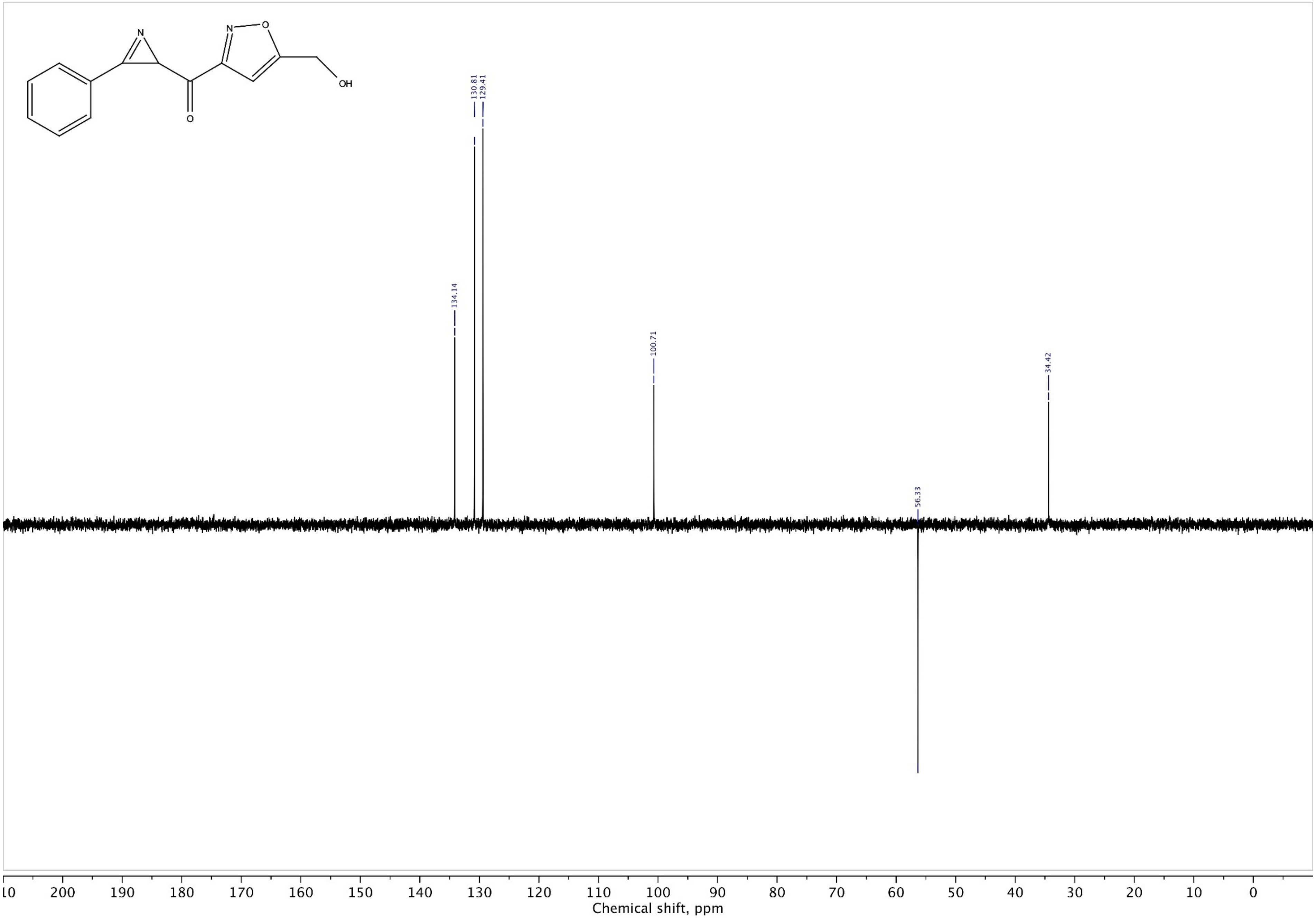

(5-(Methoxymethyl)isoxazol-3-yl)(3-phenyl-2*H*-azirin-2-yl)methanone (3d), ^{1}H NMR, $CDCl_3$, 400 MHz

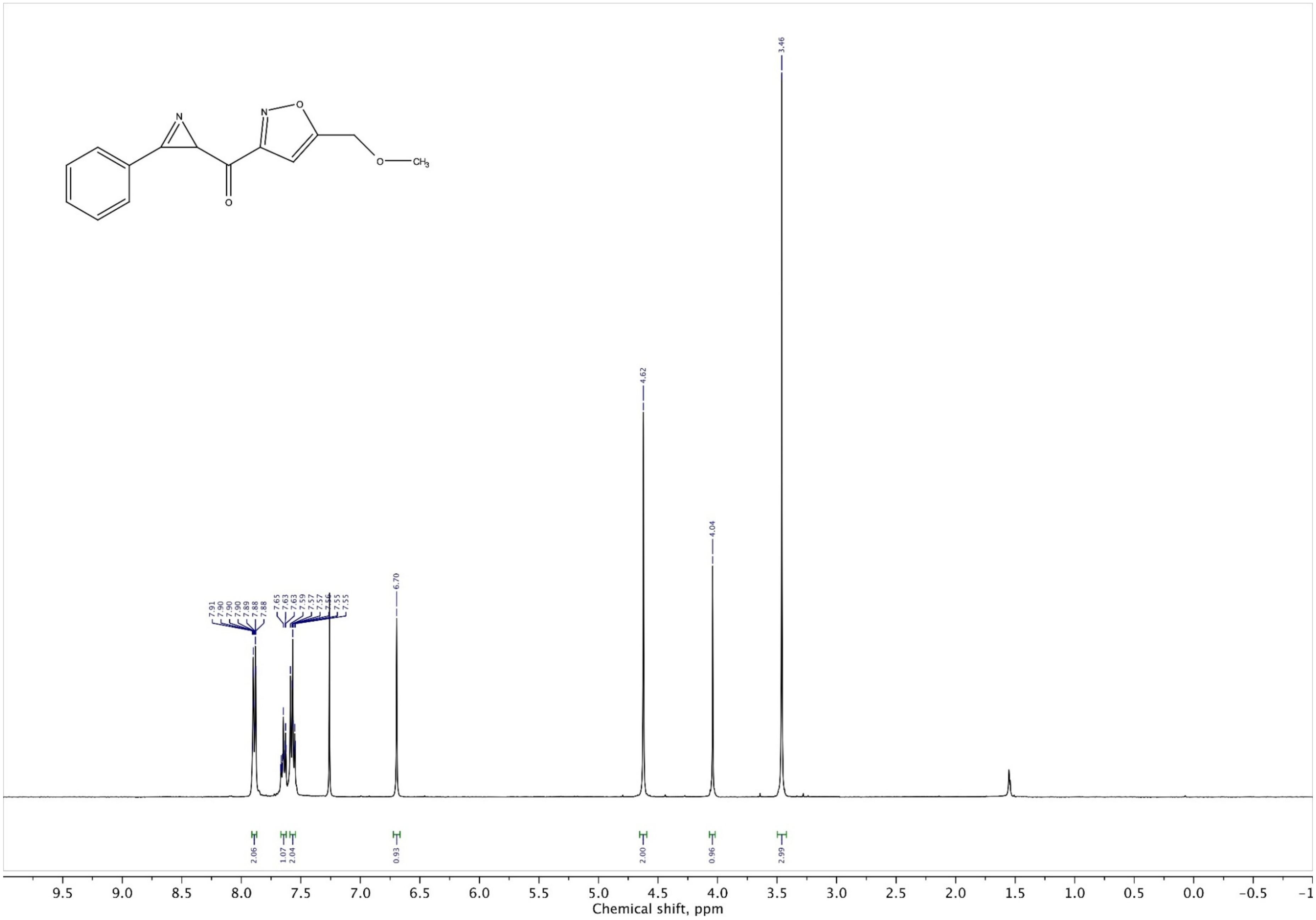

(5-(Methoxymethyl)isoxazol-3-yl)(3-phenyl-2*H*-azirin-2-yl)methanone (3d), $^{13}C\{^1H\}$ NMR, $CDCl_3$, 100 MHz

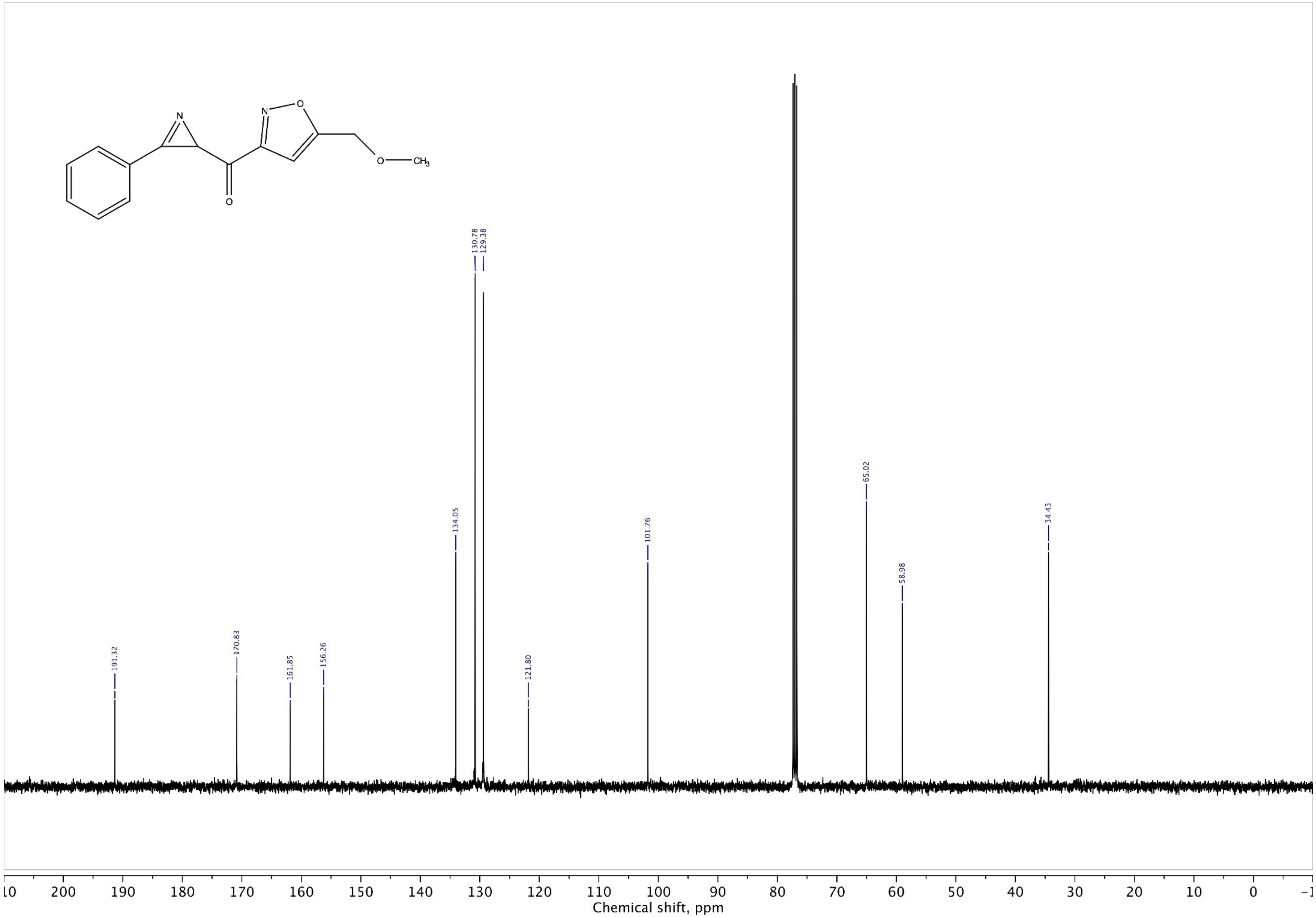

(5-(Methoxymethyl)isoxazol-3-yl)(3-phenyl-2*H*-azirin-2-yl)methanone (3d), DEPT, $CDCl_3$, 100 MHz

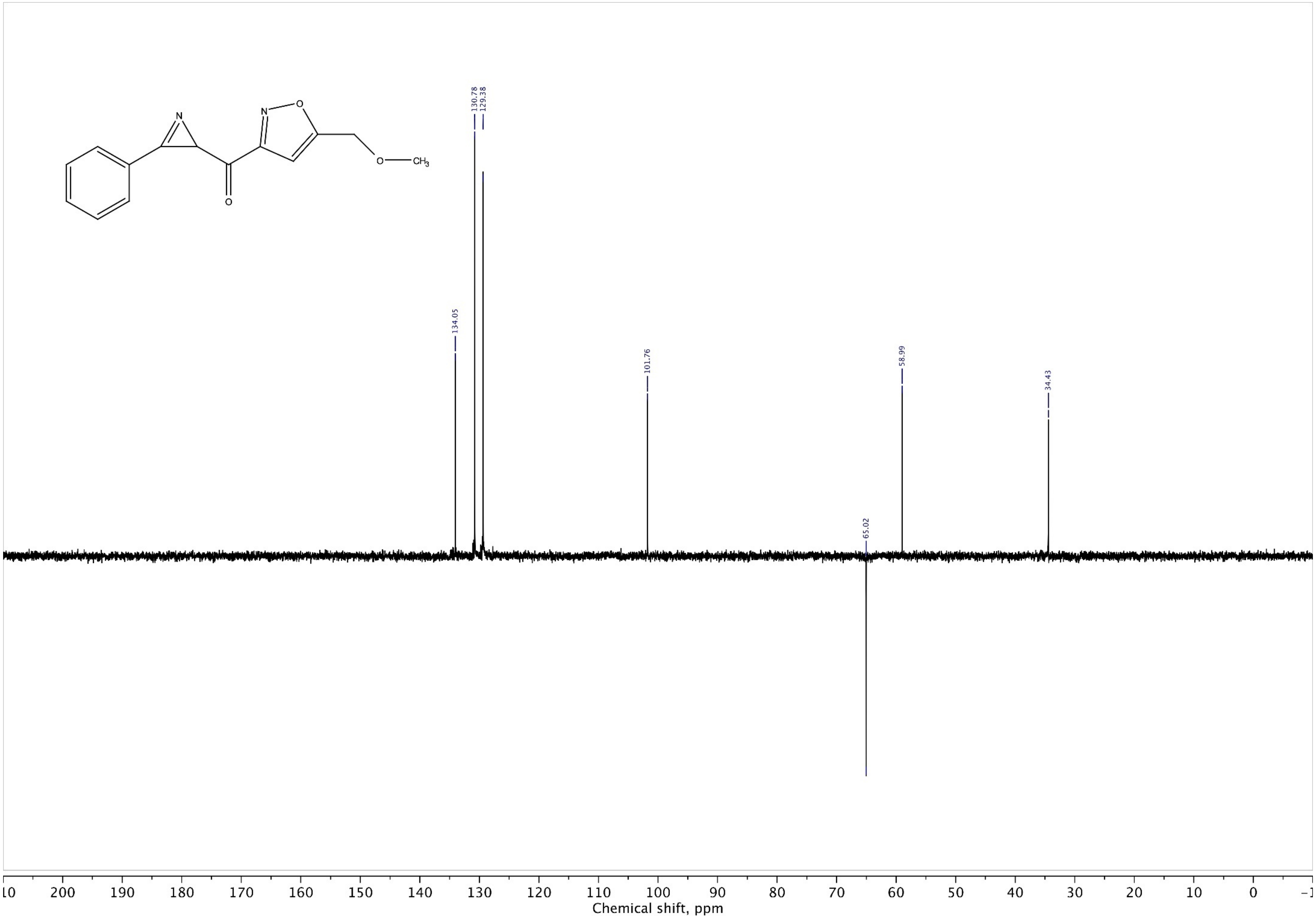

(5-(Phenoxymethyl)isoxazol-3-yl)(3-phenyl-2*H*-azirin-2-yl)methanone (3e), ^{1}H NMR, $CDCl_3$, 400 MHz

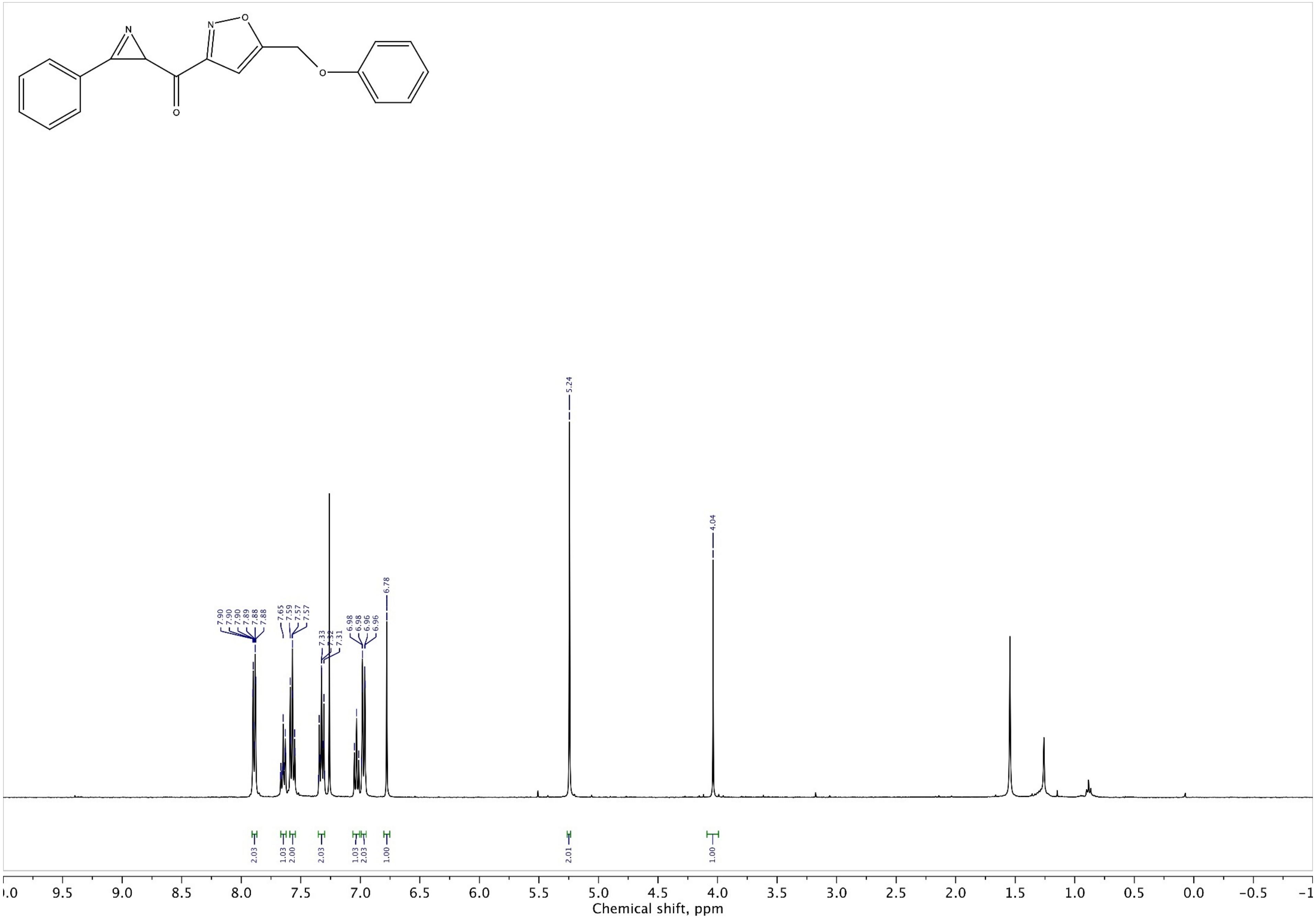

(5-(Phenoxymethyl)isoxazol-3-yl)(3-phenyl-2*H*-azirin-2-yl)methanone (3e), $^{13}C\{^1H\}$ NMR, $CDCl_3$, 100 MHz

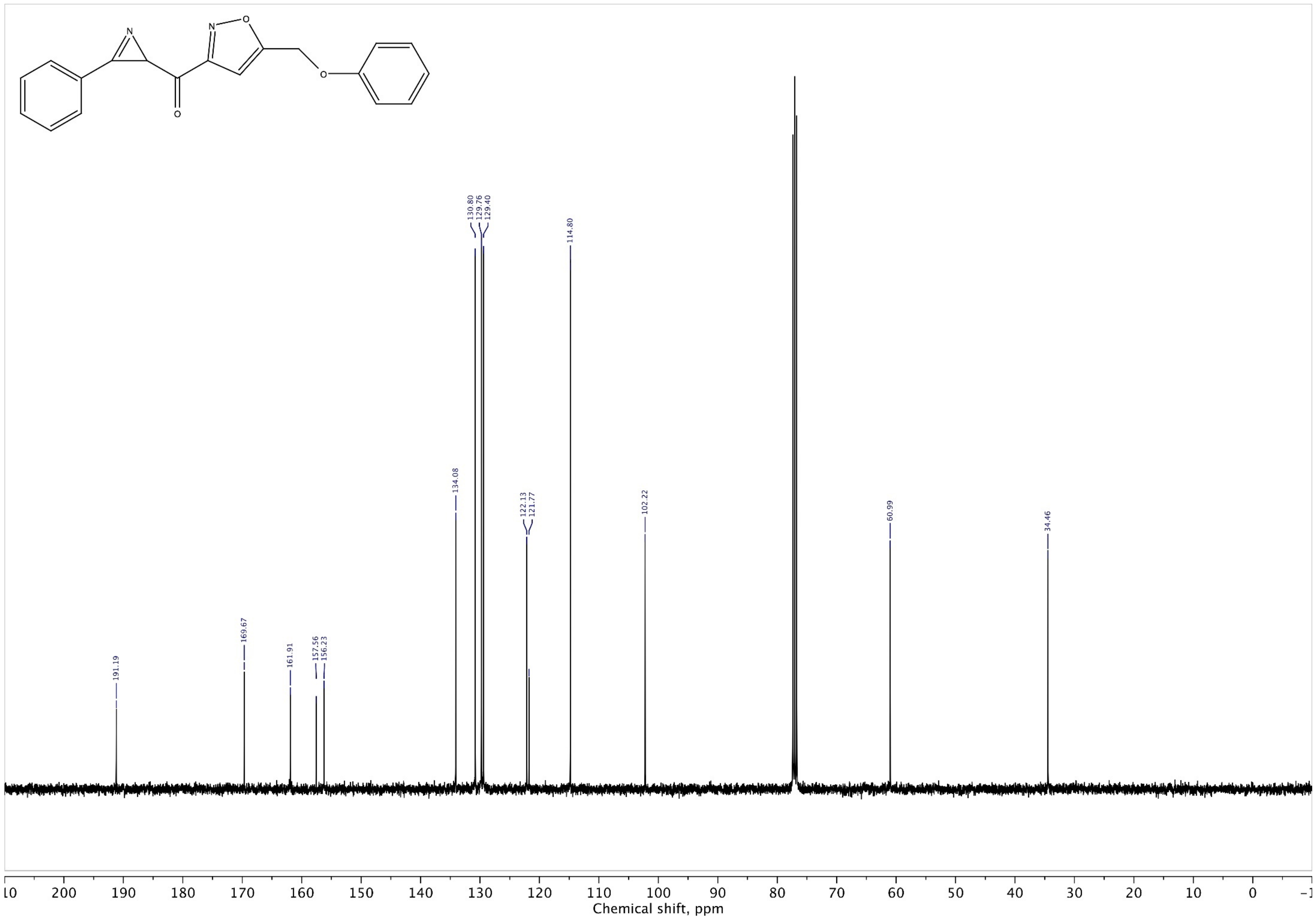

(5-(Phenoxymethyl)isoxazol-3-yl)(3-phenyl-2*H*-azirin-2-yl)methanone (3e), DEPT, $CDCl_3$, 100 MHz

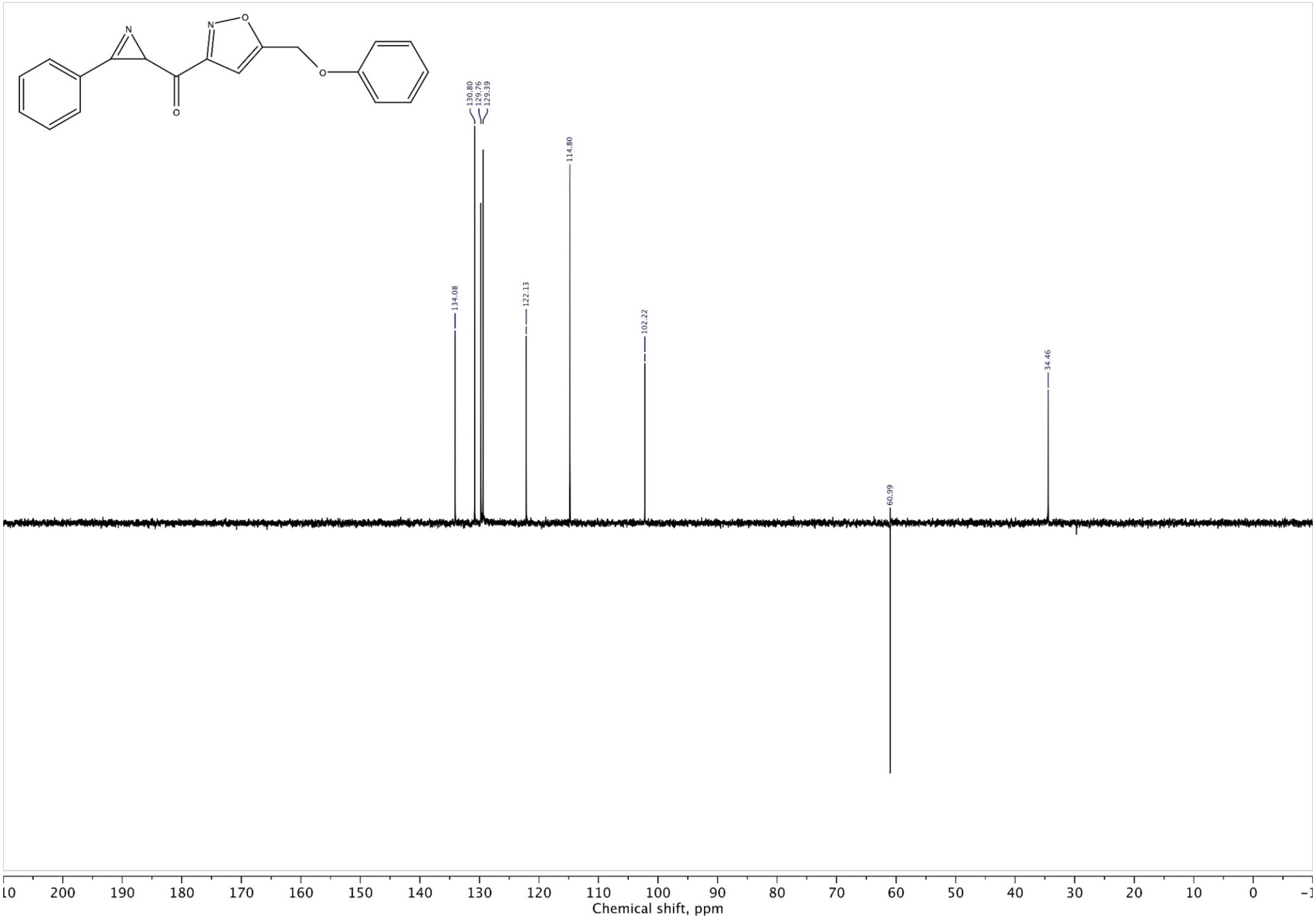

(3-(3-Phenyl-2*H*-azirine-2-carbonyl)isoxazol-5-yl)methyl benzenesulfonate (3f), ^{1}H NMR, $CDCl_3$, 400 MHz

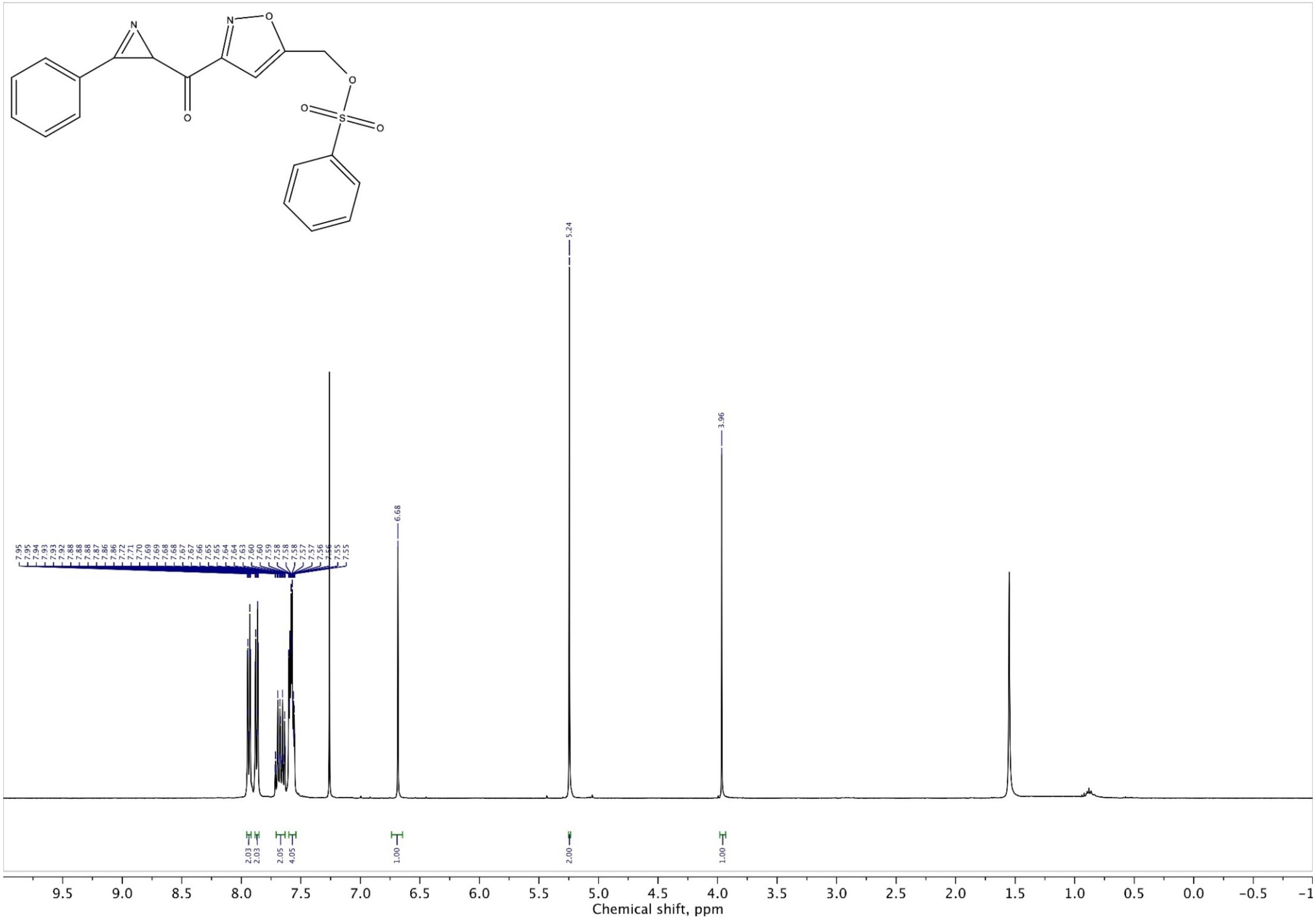

(3-(3-Phenyl-2*H*-azirine-2-carbonyl)isoxazol-5-yl)methyl benzenesulfonate (3f), $^{13}C\{^{1}H\}$ NMR, $CDCl_3$, 100 MHz

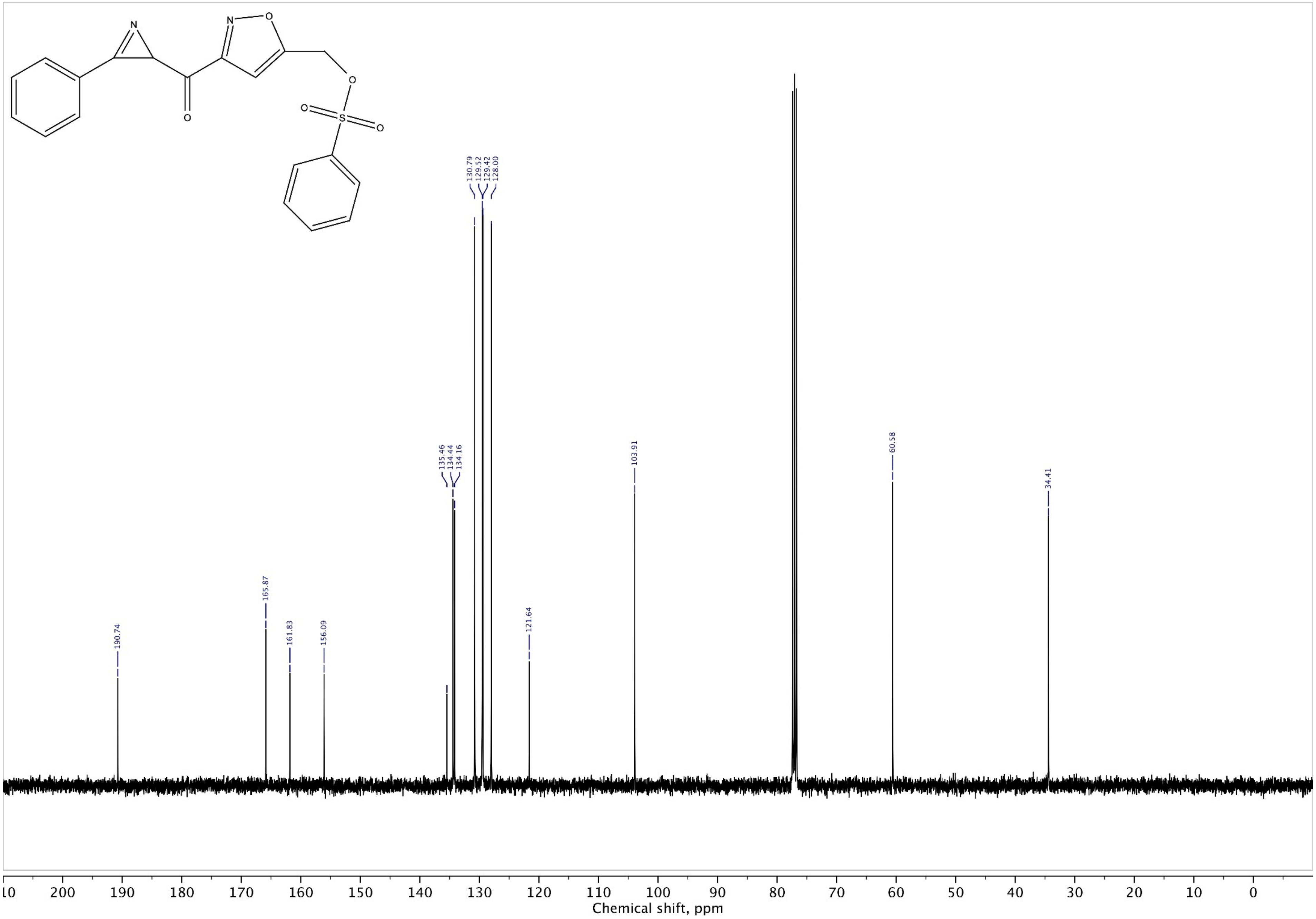

(3-(3-Phenyl-2*H*-azirine-2-carbonyl)isoxazol-5-yl)methyl benzenesulfonate (3f), DEPT, 100 MHz

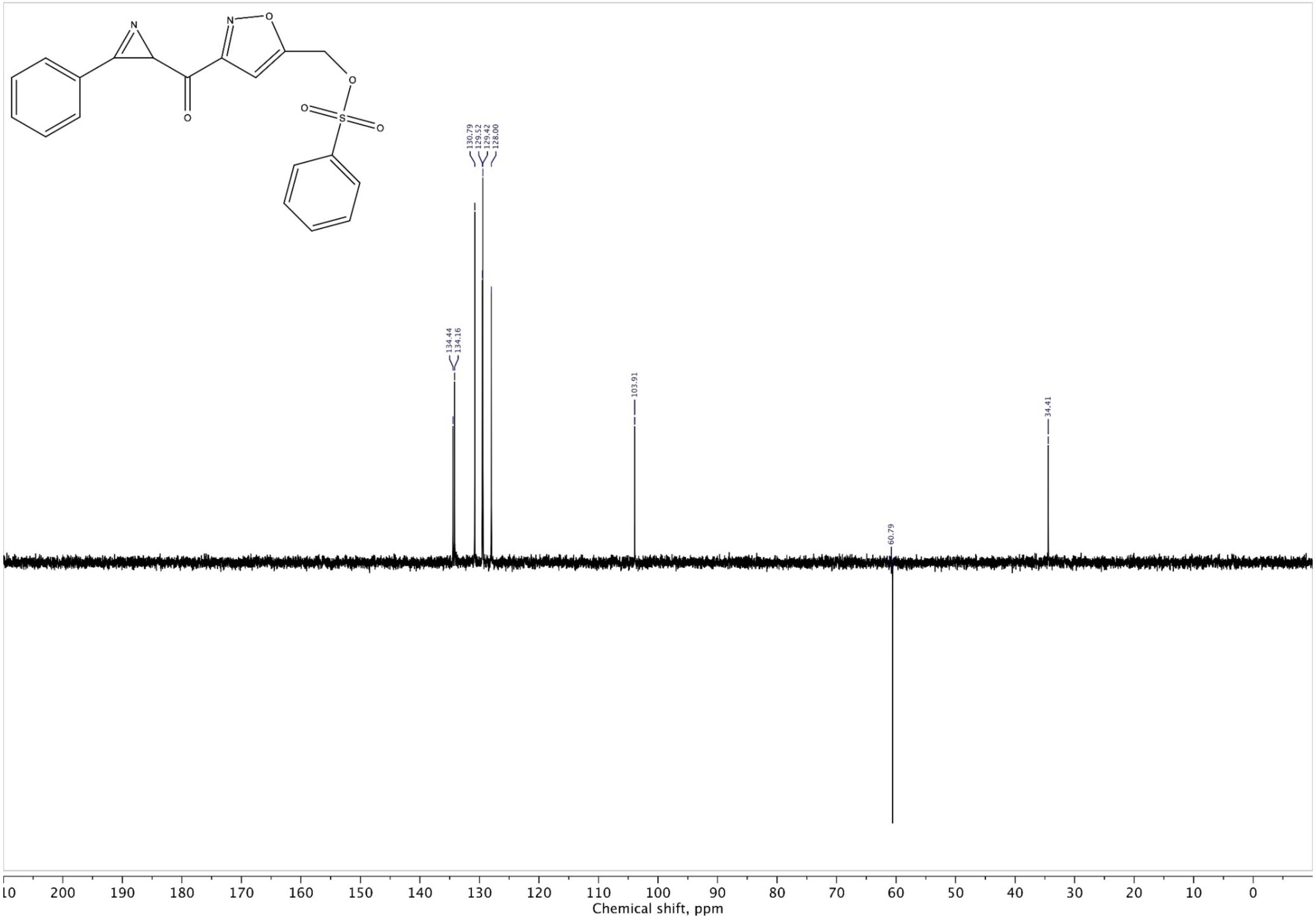

(5-(Chloromethyl)isoxazol-3-yl)(3-phenyl-2*H*-azirin-2-yl)methanoneole (3g), ^{1}H NMR, $CDCl_3$, 400 MHz

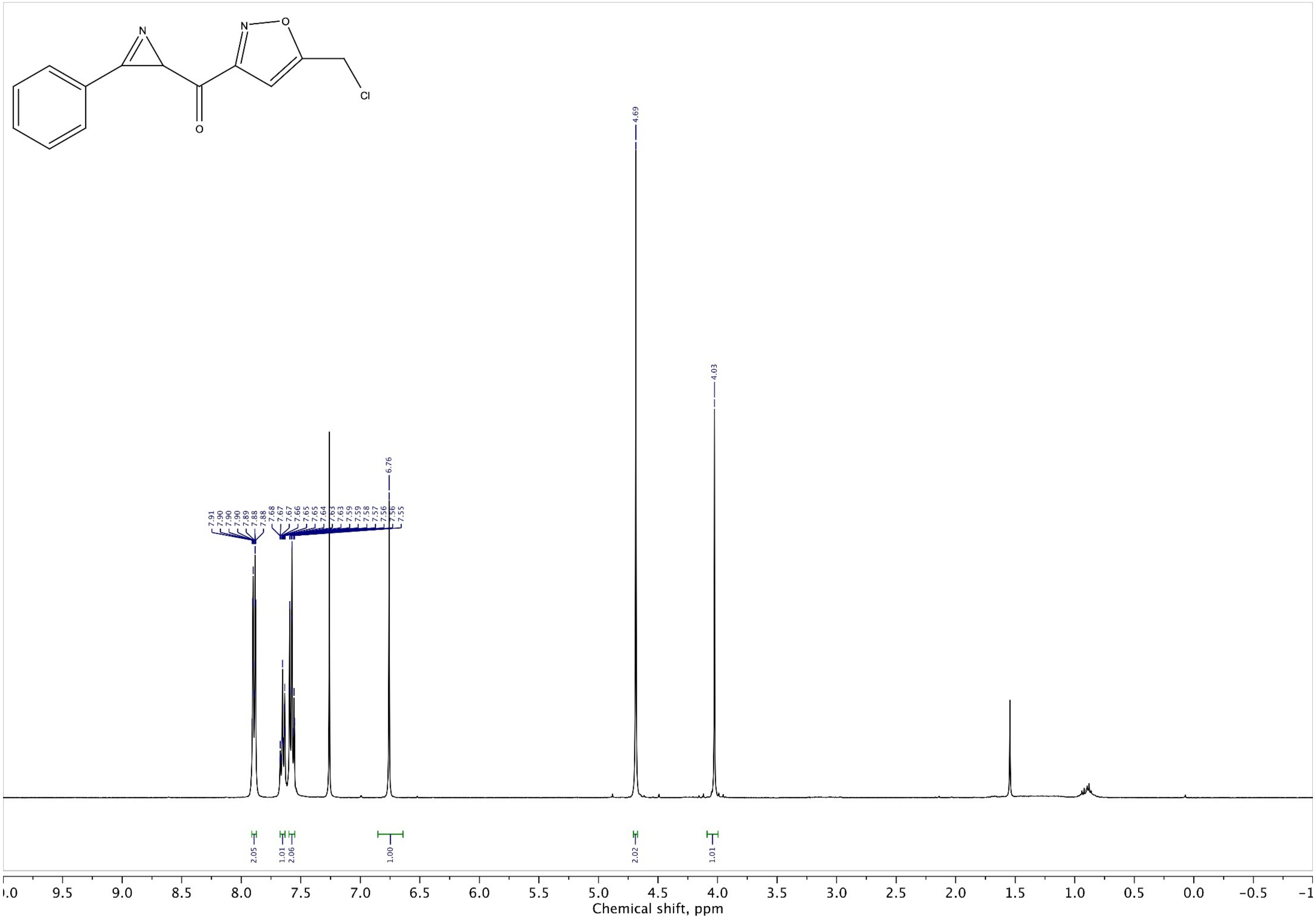

(5-(Chloromethyl)isoxazol-3-yl)(3-phenyl-2*H*-azirin-2-yl)methanoneole (3g), $^{13}C\{^{1}H\}$ NMR, $CDCl_3$, 100 MHz

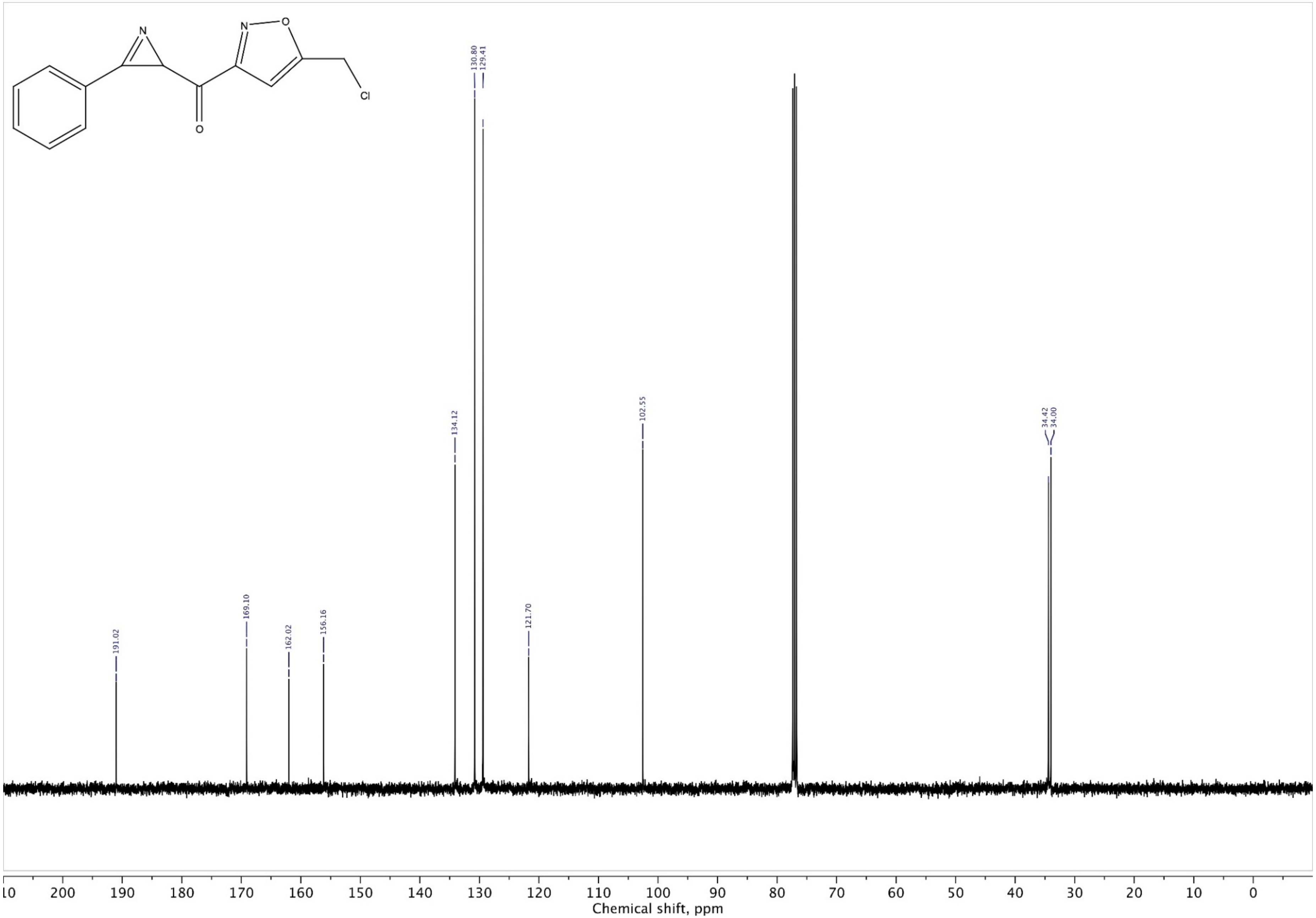

(5-(Chloromethyl)isoxazol-3-yl)(3-phenyl-2*H*-azirin-2-yl)methanoneole (3g), DEPT, $CDCl_3$, 100 MHz

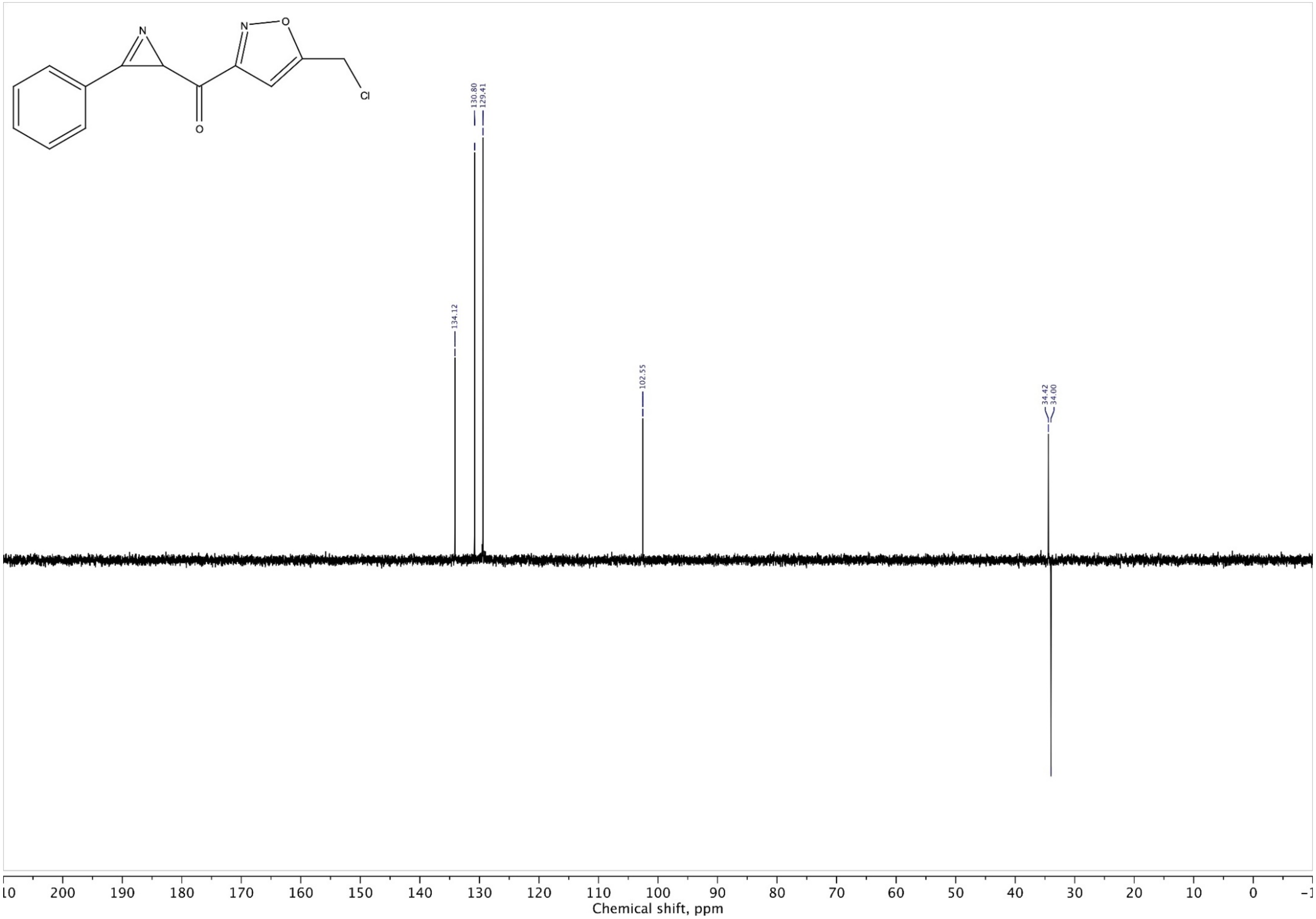

(5-(Bromomethyl)isoxazol-3-yl)(3-phenyl-2*H*-azirin-2-yl)methanone (3h), ^{1}H NMR, $CDCl_3$, 400 MHz

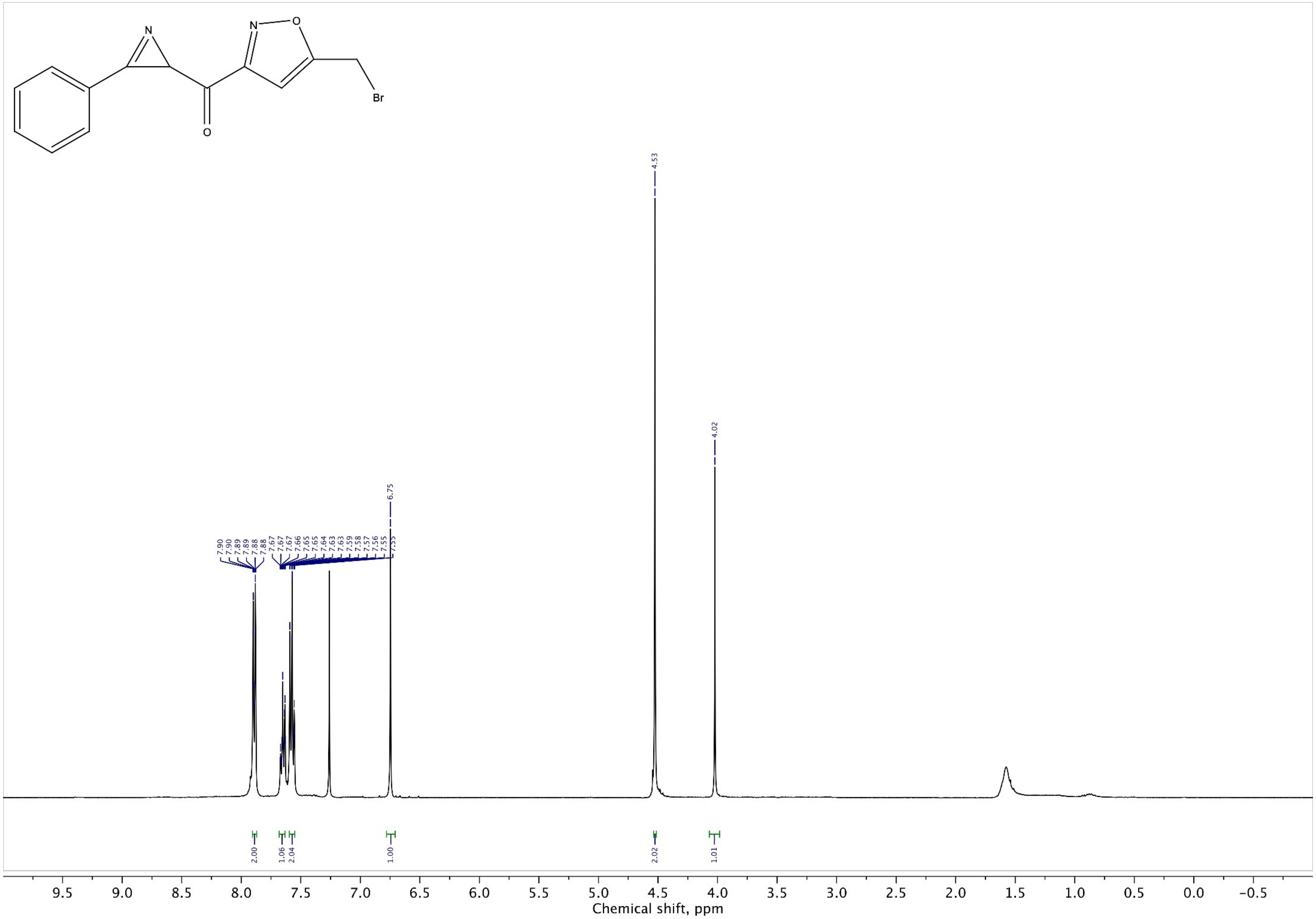

(5-(Bromomethyl)isoxazol-3-yl)(3-phenyl-2*H*-azirin-2-yl)methanone (3h), $^{13}C\{^{1}H\}$ NMR, $CDCl_3$, 100 MHz

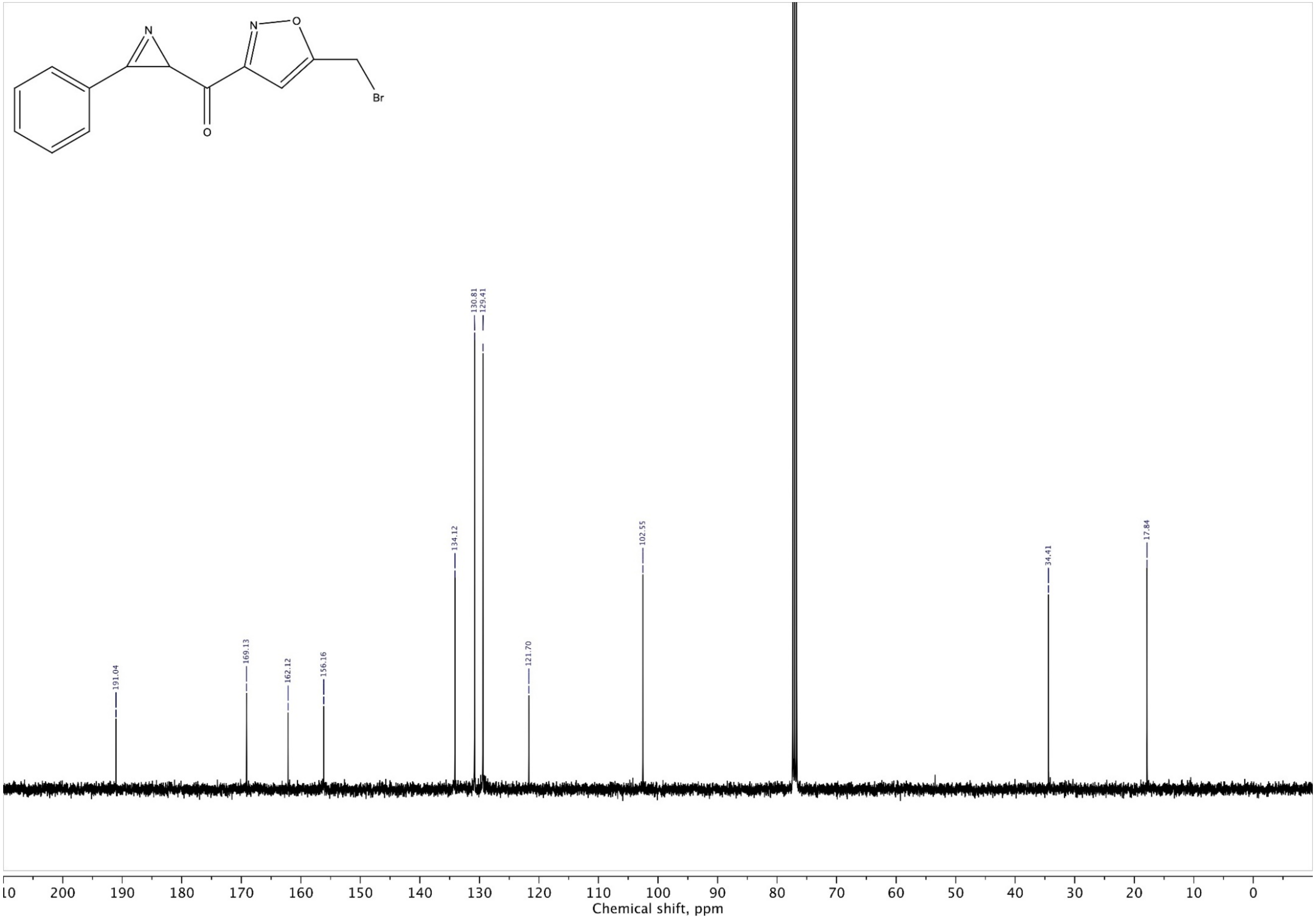

(5-(Bromomethyl)isoxazol-3-yl)(3-phenyl-2*H*-azirin-2-yl)methanone (3h), DEPT, $CDCl_3$, 100 MHz

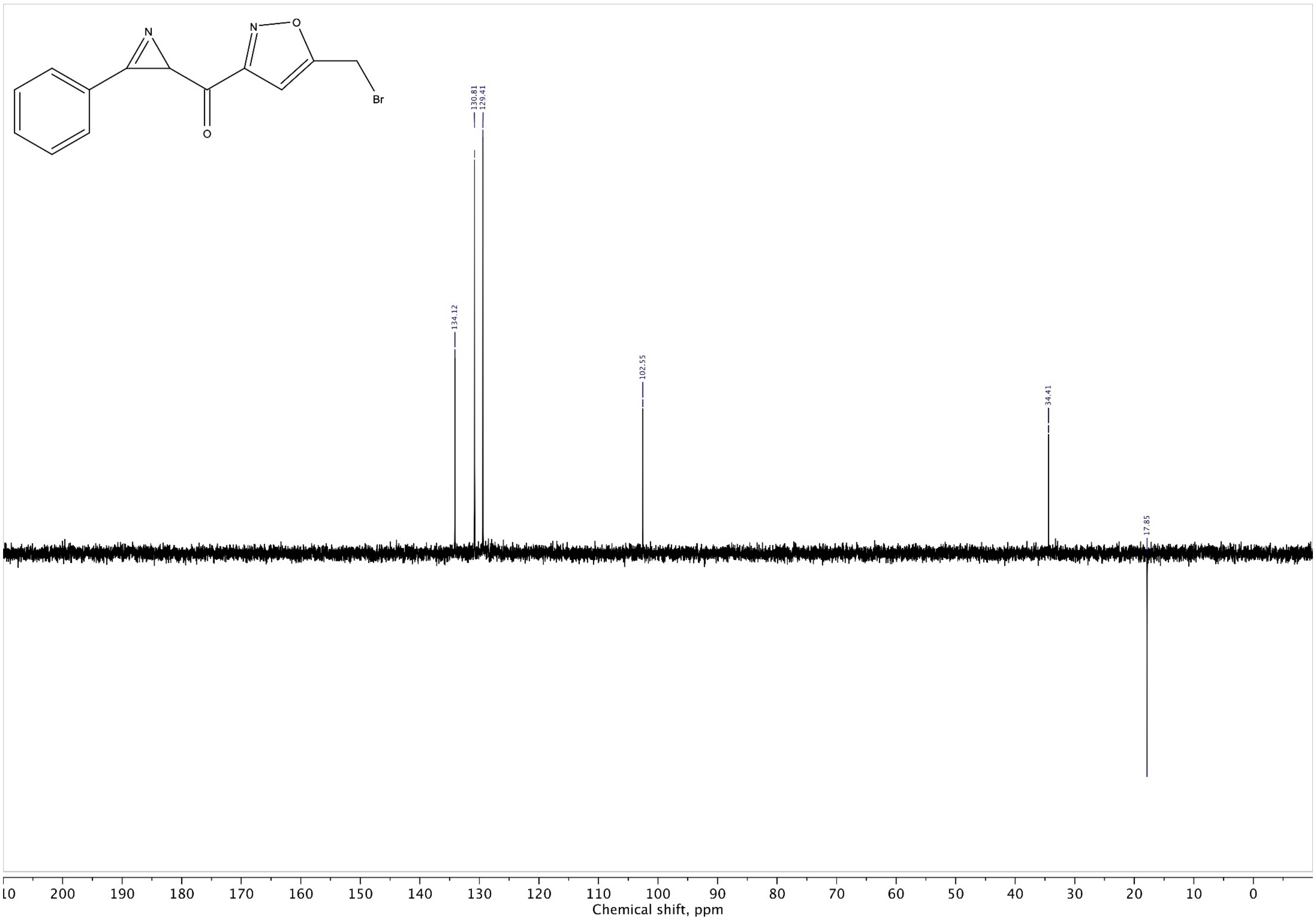

(3-Phenyl-2*H*-azirin-2-yl)(5-(trimethylsilyl)isoxazol-3-yl)methanone (3i), ^{1}H NMR, $CDCl_3$, 400 MHz

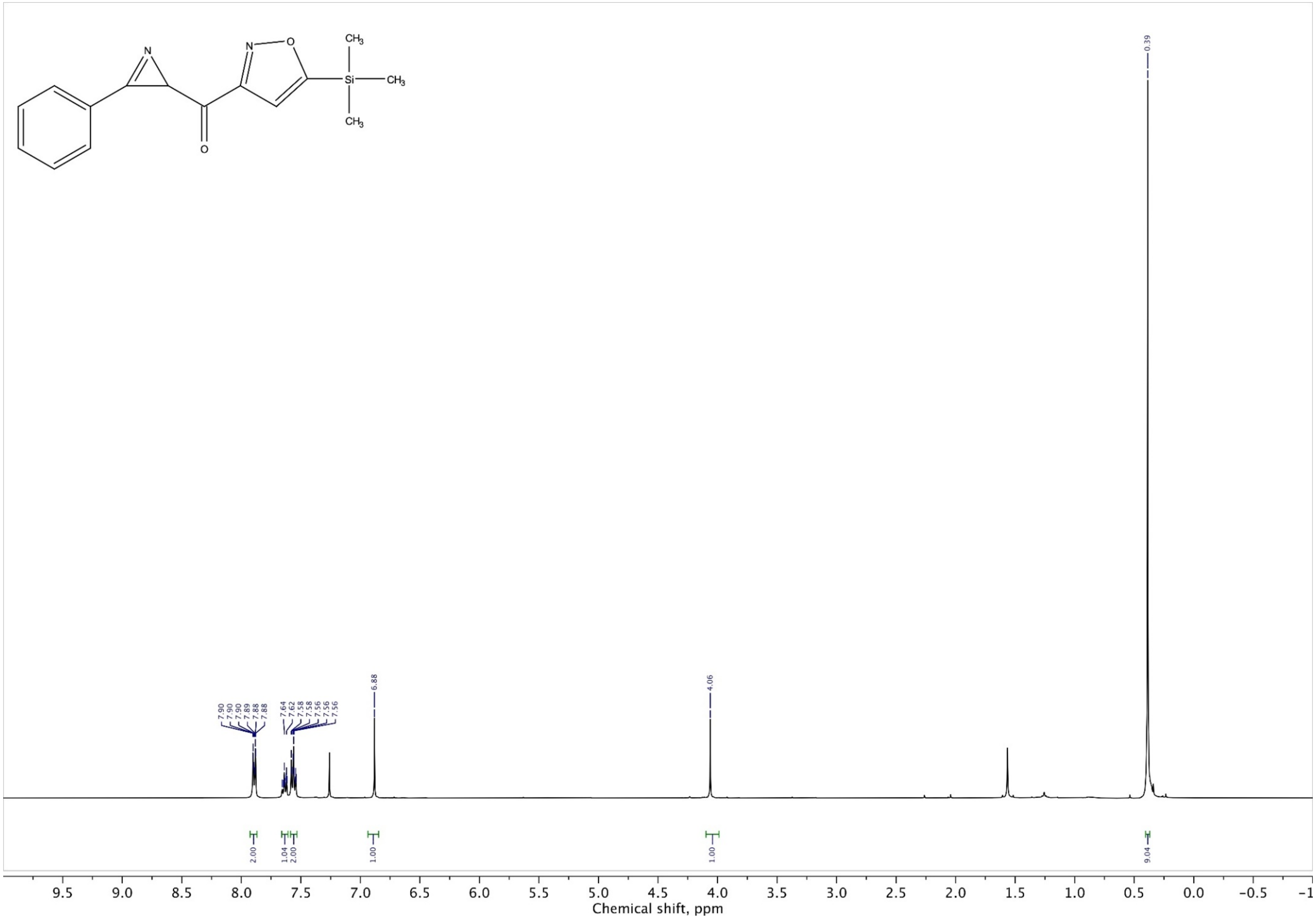

(3-Phenyl-2*H*-azirin-2-yl)(5-(trimethylsilyl)isoxazol-3-yl)methanone (3i), $^{13}C\{^{1}H\}$ NMR, $CDCl_3$, 100 MHz

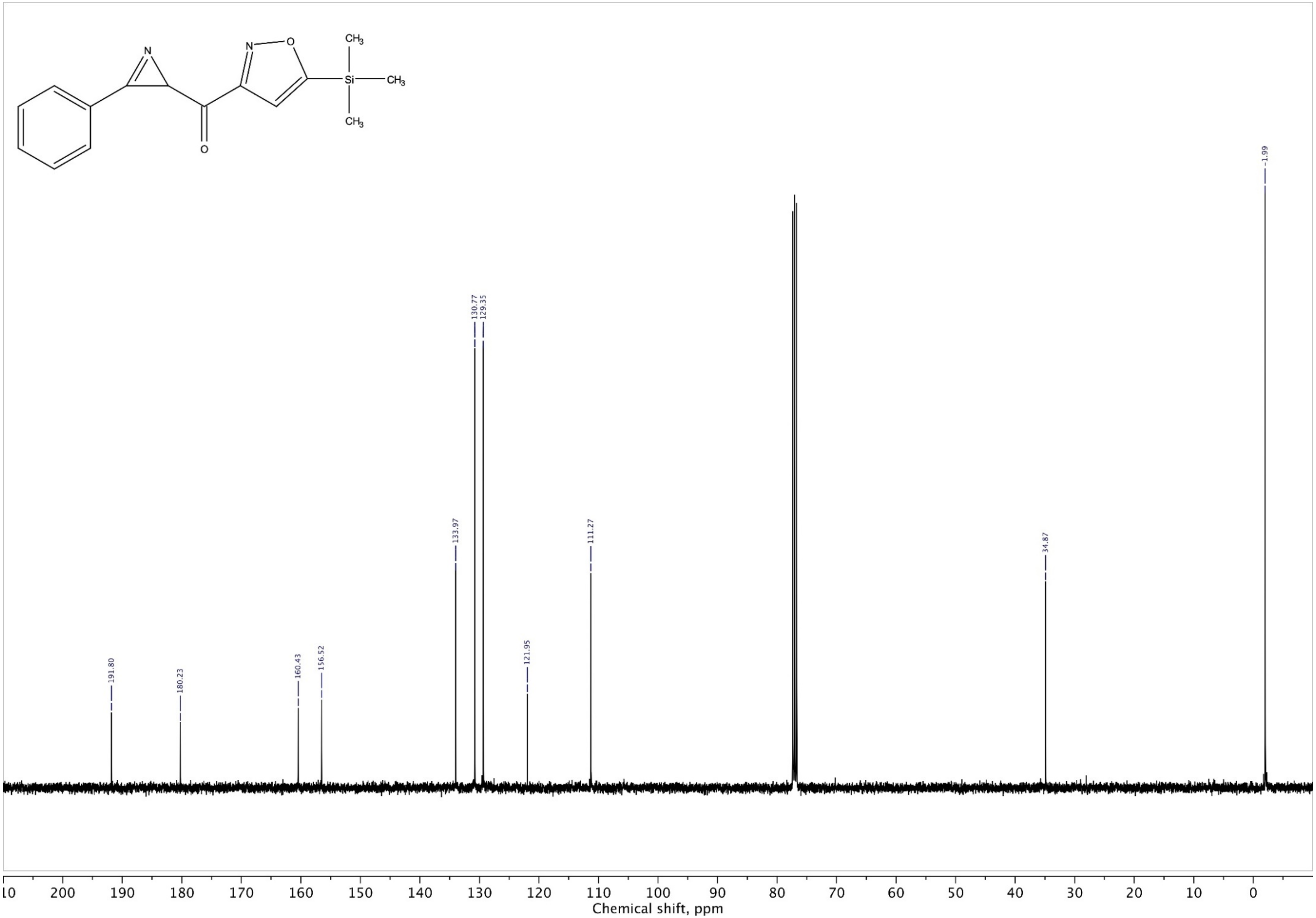

(3-Phenyl-2*H*-azirin-2-yl)(5-(trimethylsilyl)isoxazol-3-yl)methanone (3i), DEPT, $CDCl_3$, 100 MHz

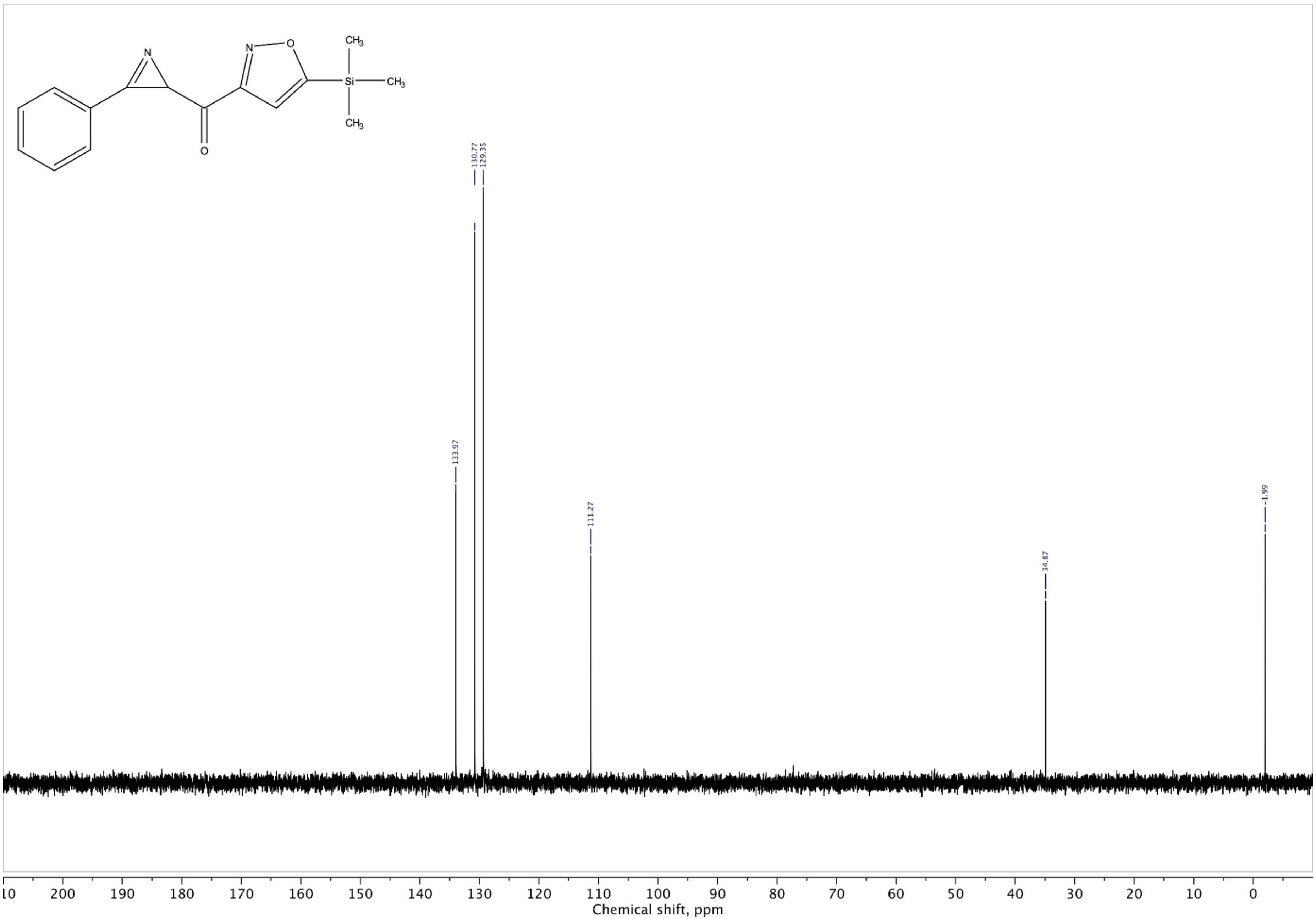

Methyl 3-(3-(p-tolyl)-2*H*-azirine-2-carbonyl)isoxazole-5-carboxylate (3j), ^{1}H NMR, $CDCl_3$, 400 MHz

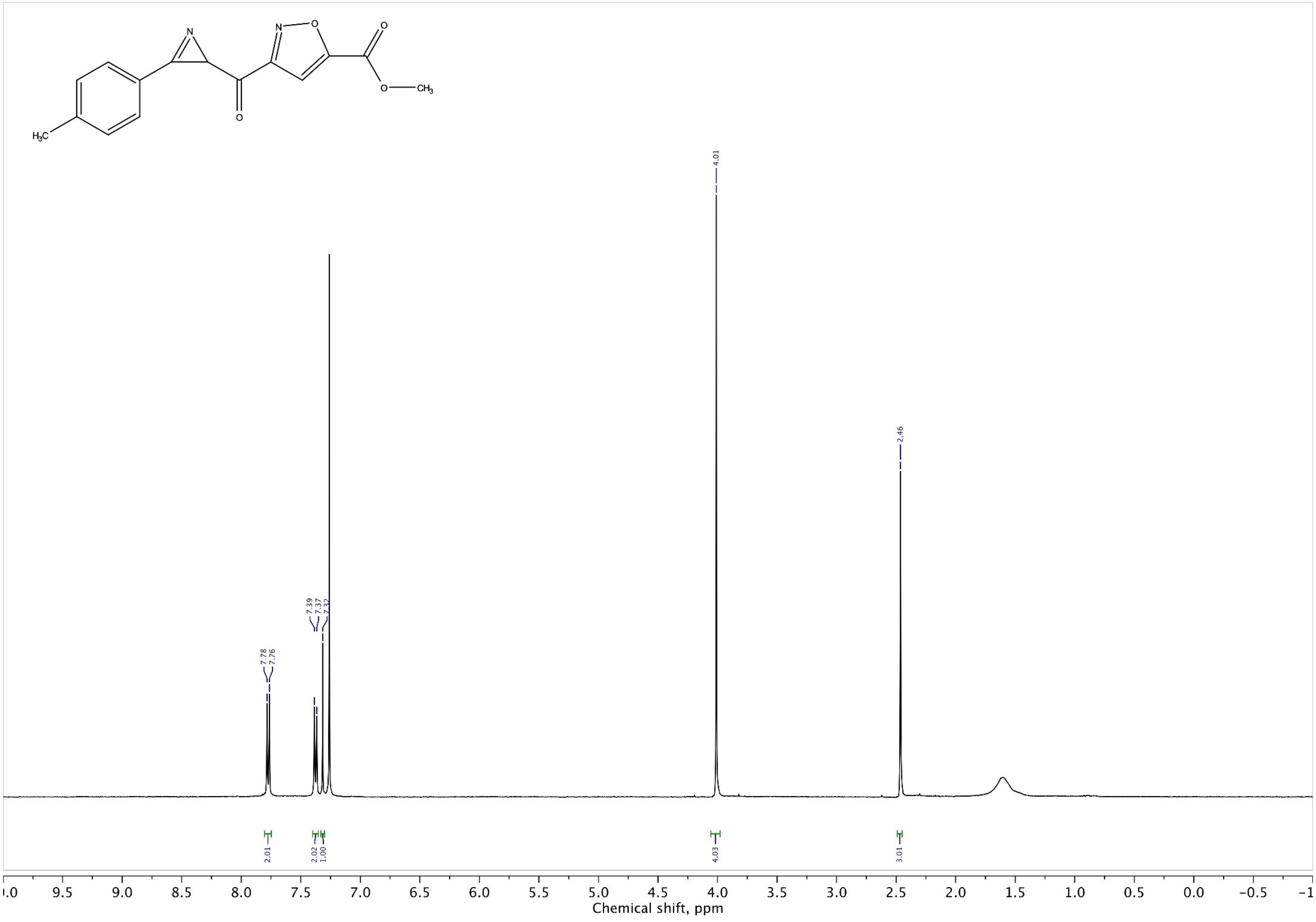

Methyl 3-(3-(p-tolyl)-2*H*-azirine-2-carbonyl)isoxazole-5-carboxylate (3j), $^{13}C\{^{1}H\}$ NMR, $CDCl_3$, 100 MHz

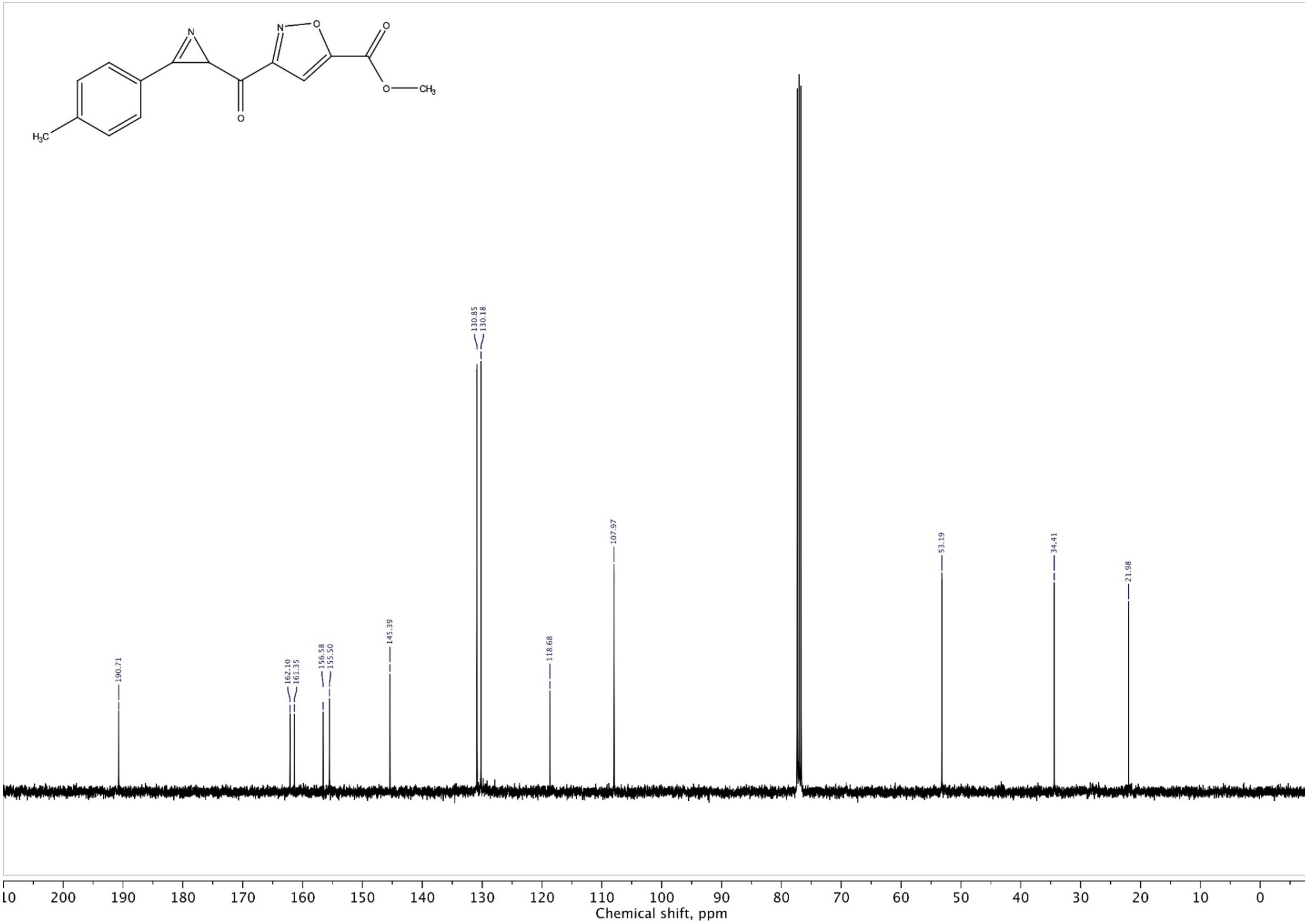

Methyl 3-(3-(p-tolyl)-2*H*-azirine-2-carbonyl)isoxazole-5-carboxylate (3j), DEPT, $CDCl_3$, 100 MHz

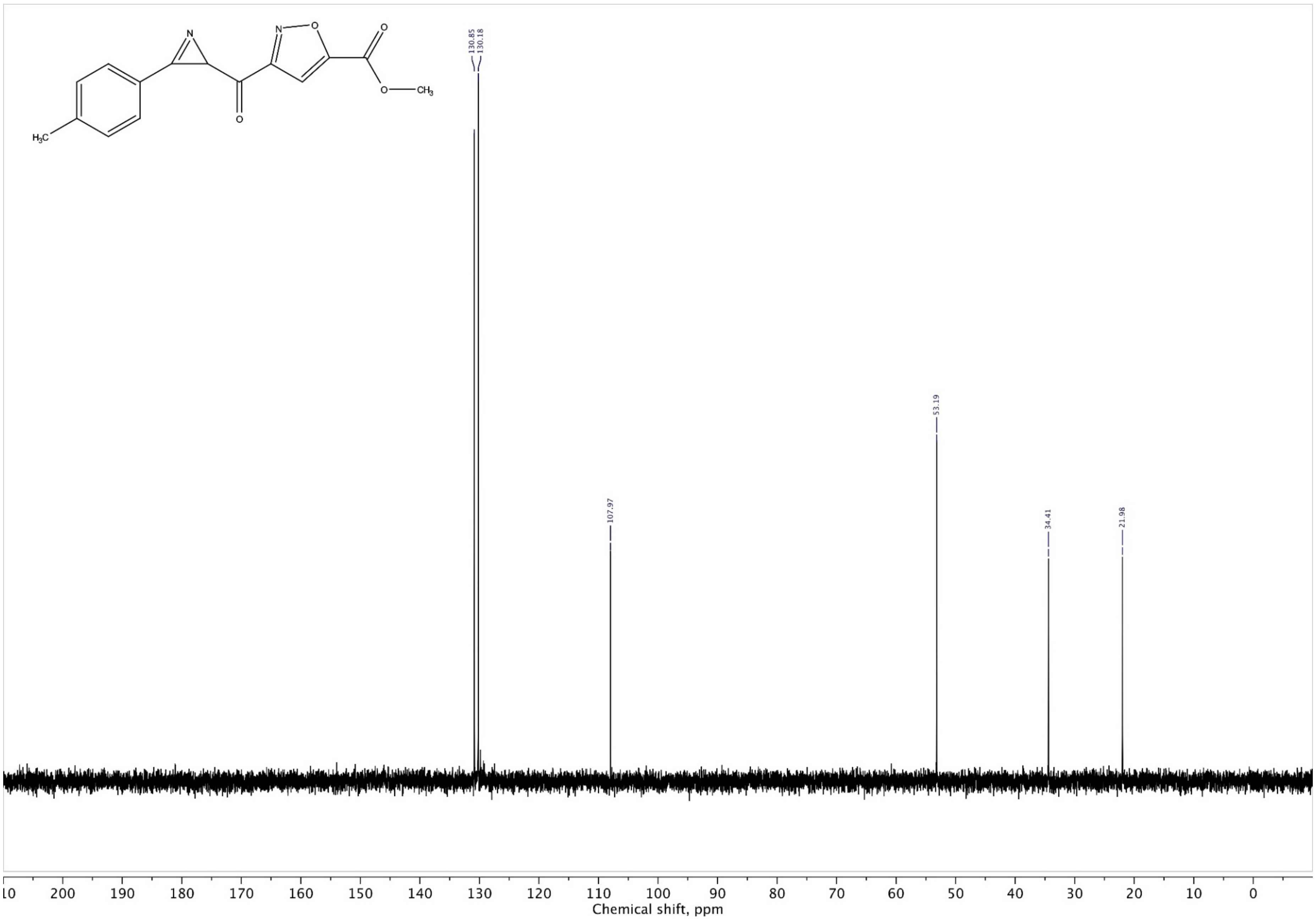

Methyl 3-(3-(4-(tert-butyl)phenyl)-2*H*-azirine-2-carbonyl)isoxazole-5-carboxylate (3k), ^{1}H NMR, $CDCl_3$, 400 MHz

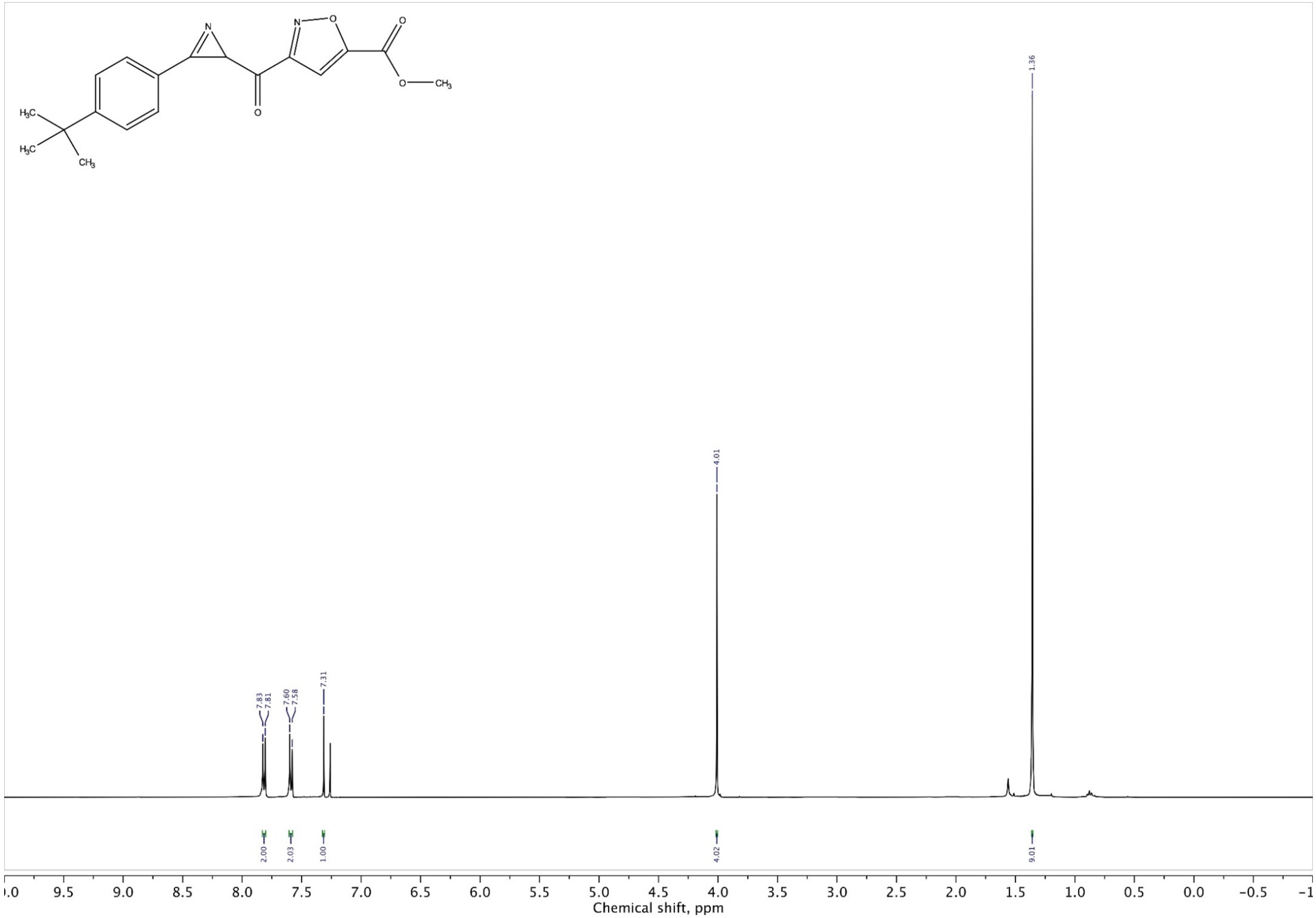

Methyl 3-(3-(4-(tert-butyl)phenyl)-2*H*-azirine-2-carbonyl)isoxazole-5-carboxylate (3k), $^{13}C\{^{1}H\}$ NMR, $CDCl_3$, 100 MHz

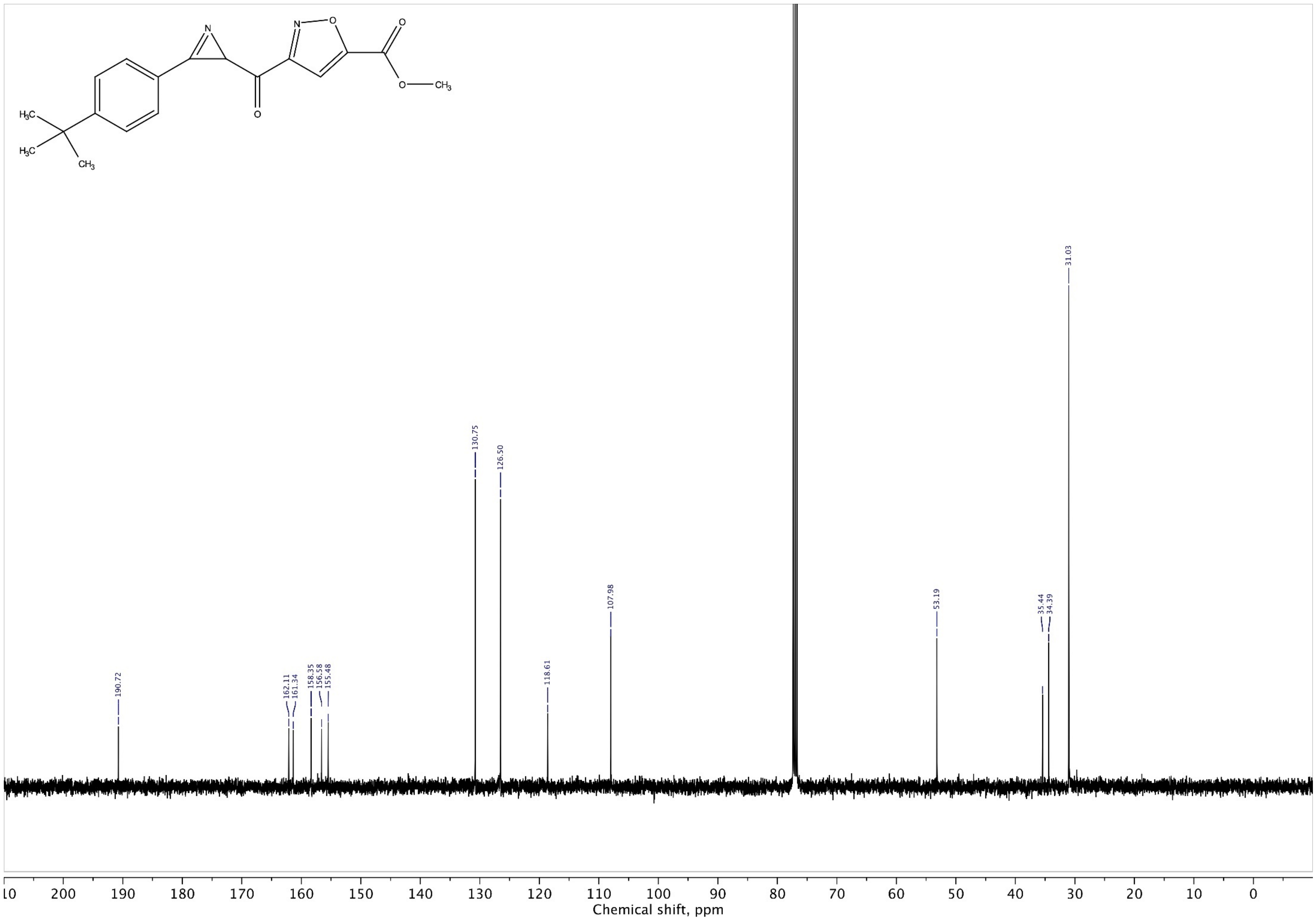

Methyl 3-(3-(4-(tert-butyl)phenyl)-2*H*-azirine-2-carbonyl)isoxazole-5-carboxylate (3k), DEPT, $CDCl_3$, 100 MHz

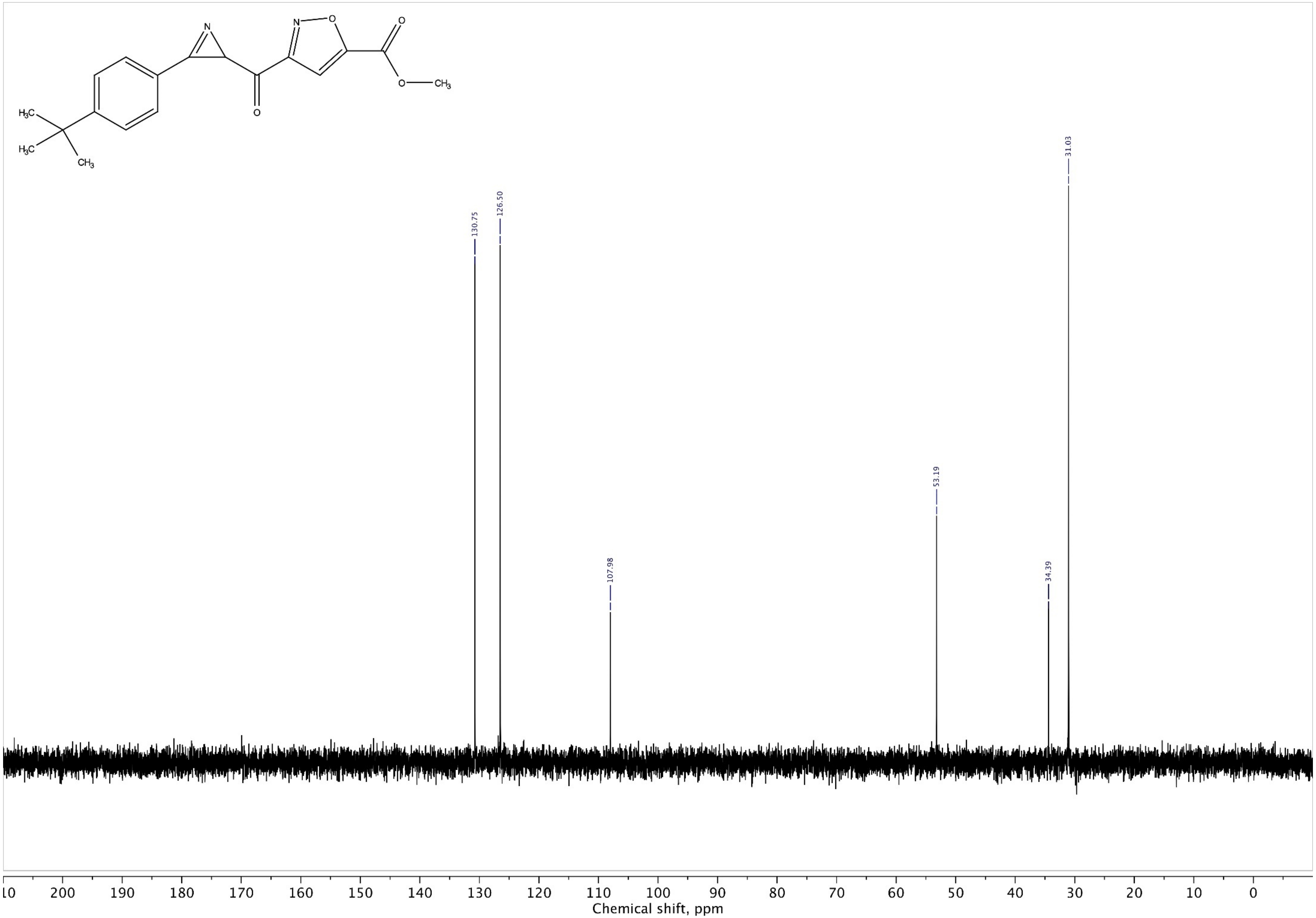

Methyl 3-(3-(4-methoxyphenyl)-2*H*-azirine-2-carbonyl)isoxazole-5-carboxylate (3l), ^{1}H NMR, $CDCl_3$, 400 MHz

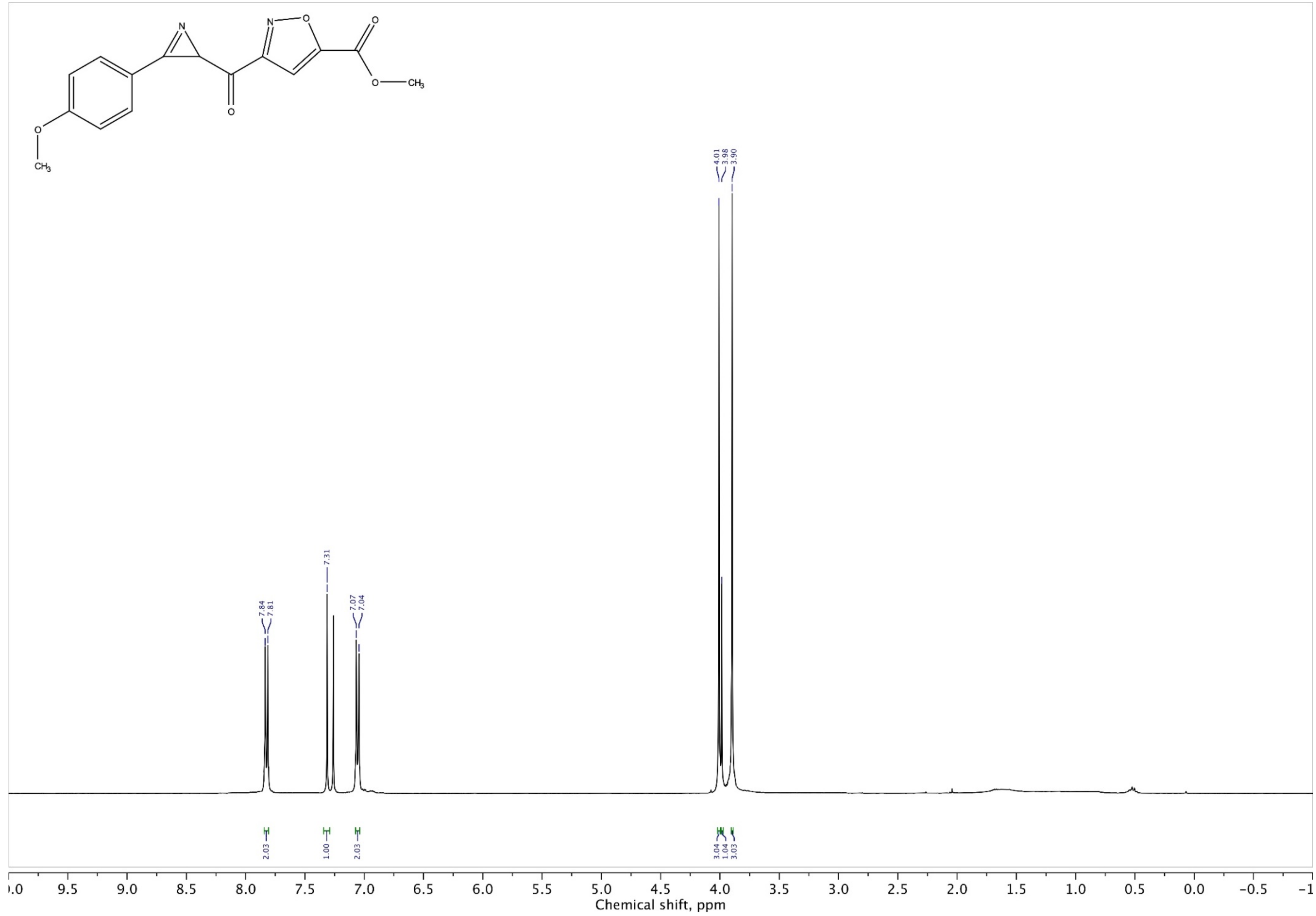

Methyl 3-(3-(4-methoxyphenyl)-2*H*-azirine-2-carbonyl)isoxazole-5-carboxylate (3l), $^{13}C\{^{1}H\}$ NMR, $CDCl_3$, 100 MHz

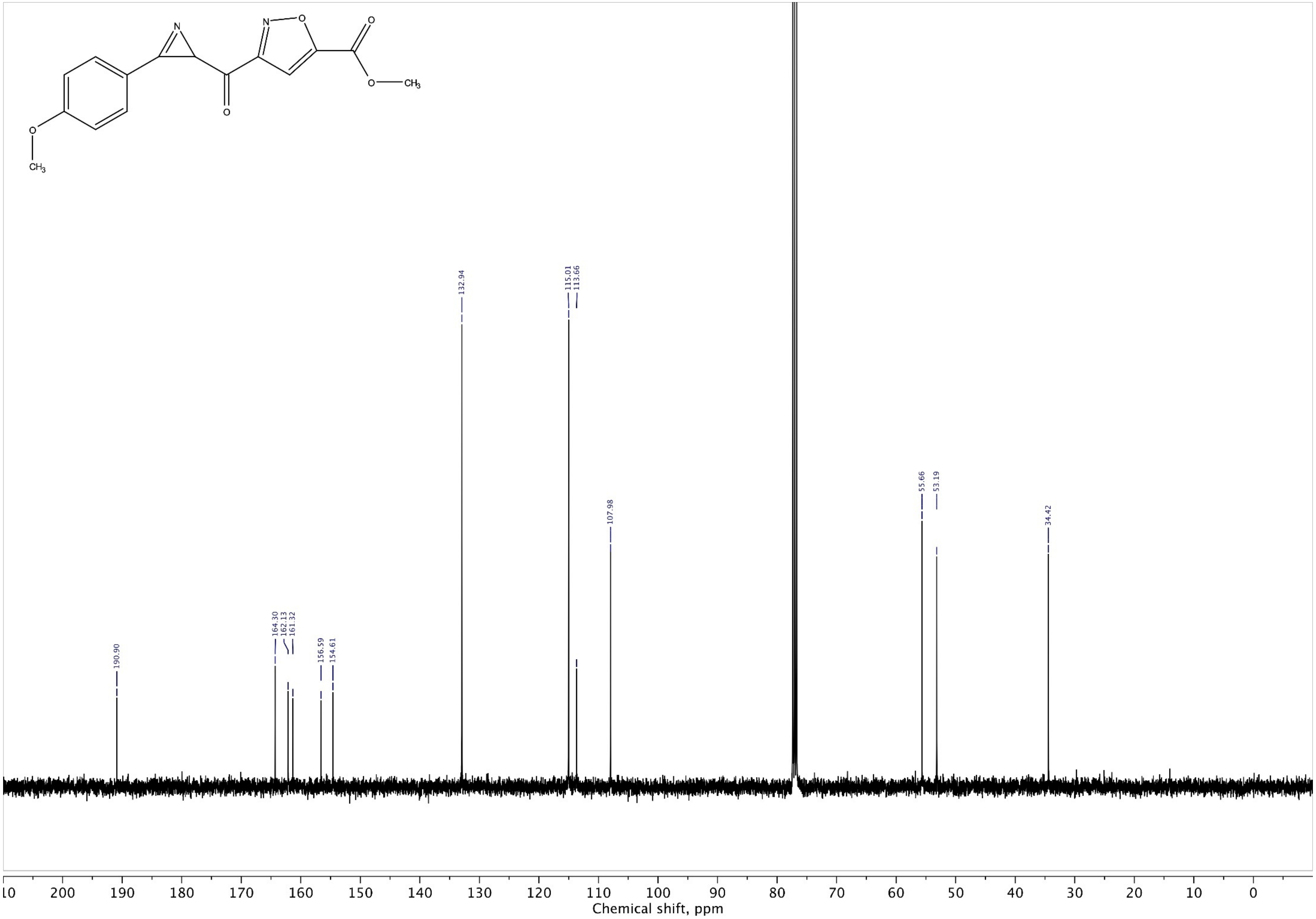

Methyl 3-(3-(4-methoxyphenyl)-2*H*-azirine-2-carbonyl)isoxazole-5-carboxylate (3l), DEPT, $CDCl_3$, 100 MHz

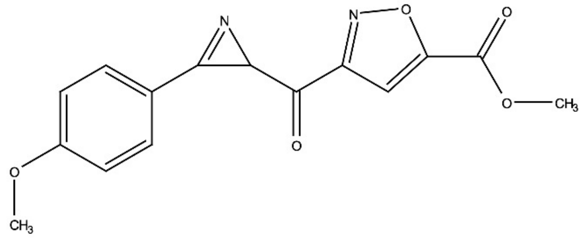

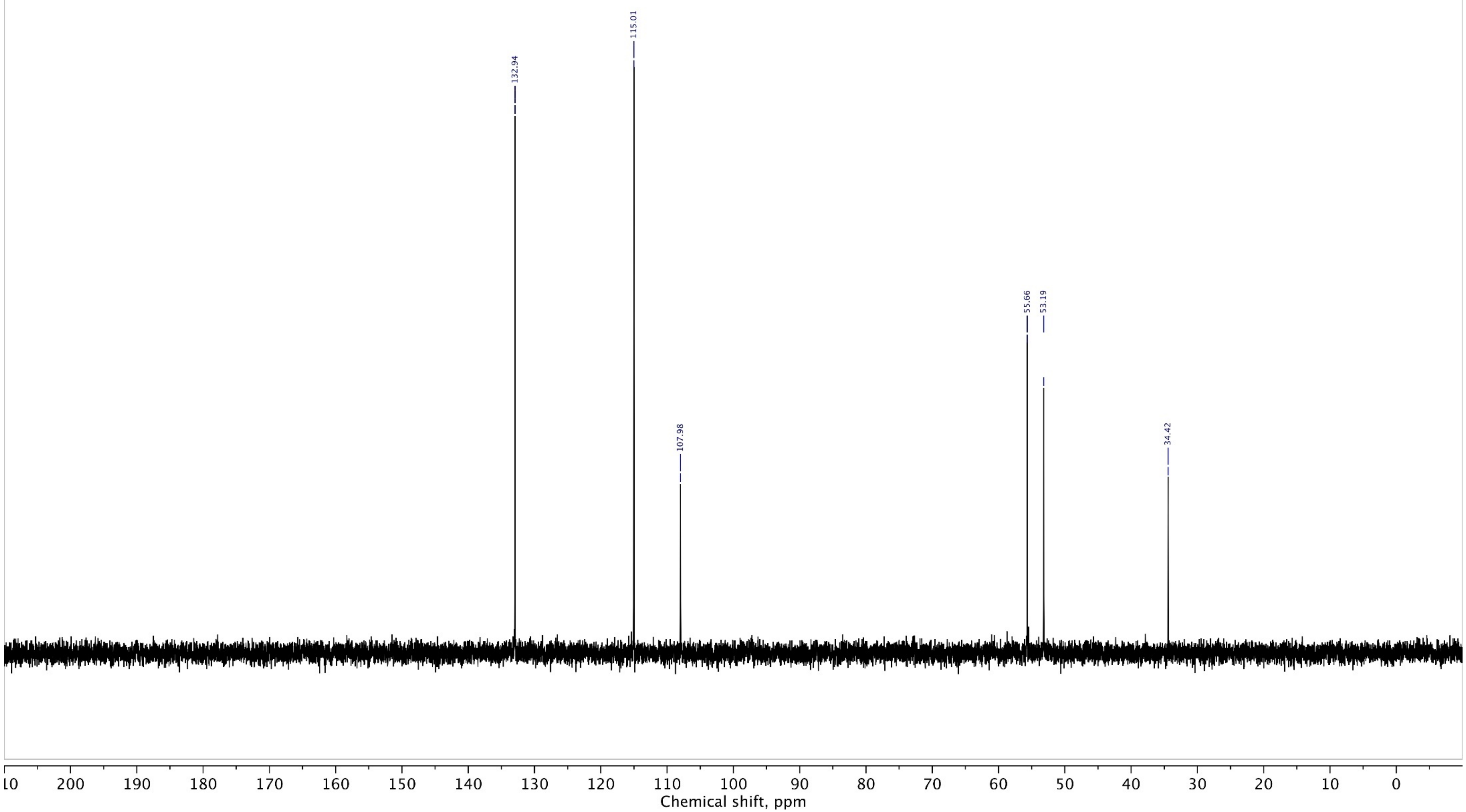

(3-(4-Methoxyphenyl)-2*H*-azirin-2-yl)(5-(phenoxymethyl)isoxazol-3-yl)methanone (3m), ^{1}H NMR, $CDCl_3$, 400 MHz

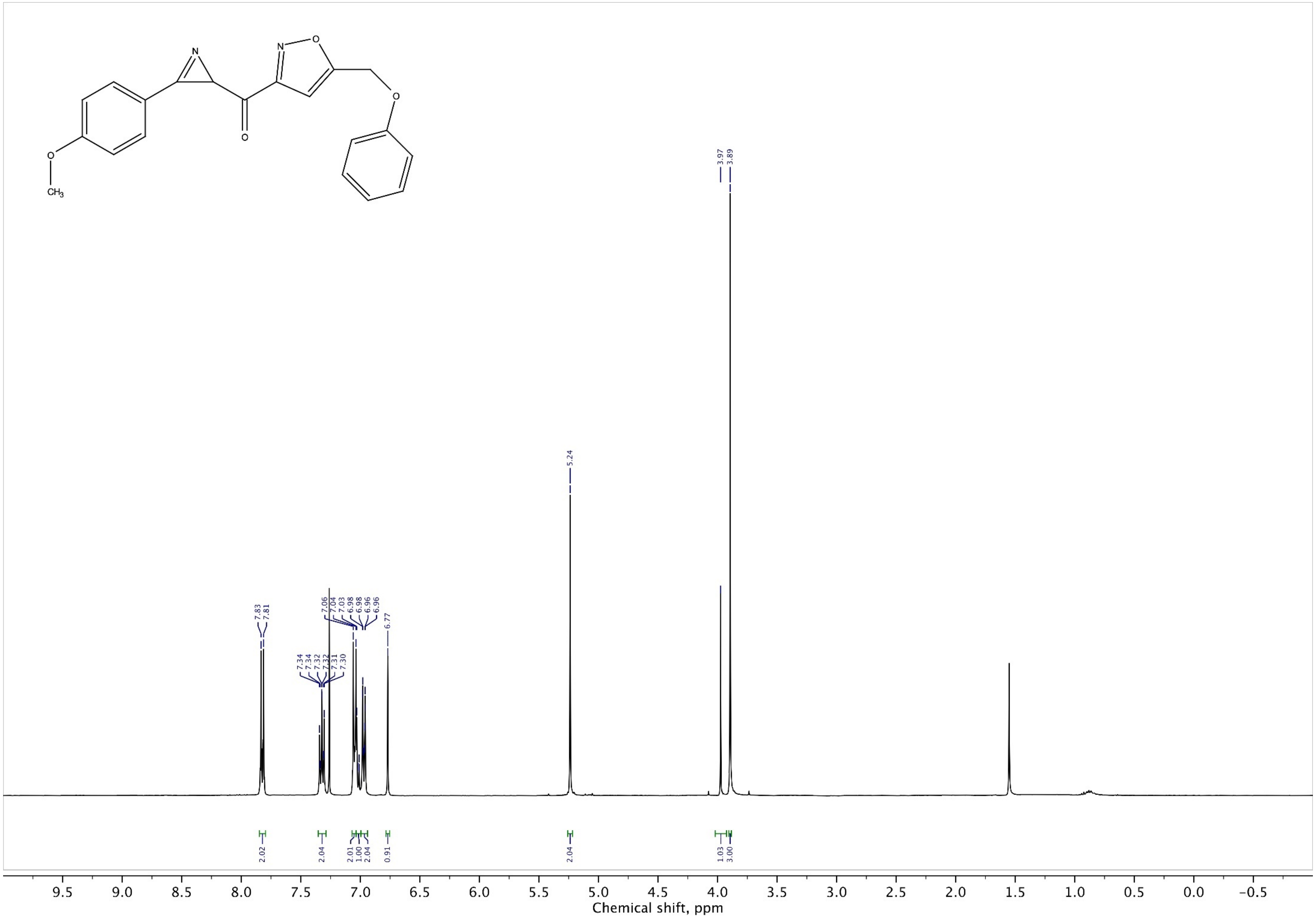

(3-(4-Methoxyphenyl)-2*H*-azirin-2-yl)(5-(phenoxymethyl)isoxazol-3-yl)methanone (3m), $^{13}C\{^{1}H\}$ NMR, $CDCl_3$, 100 MHz

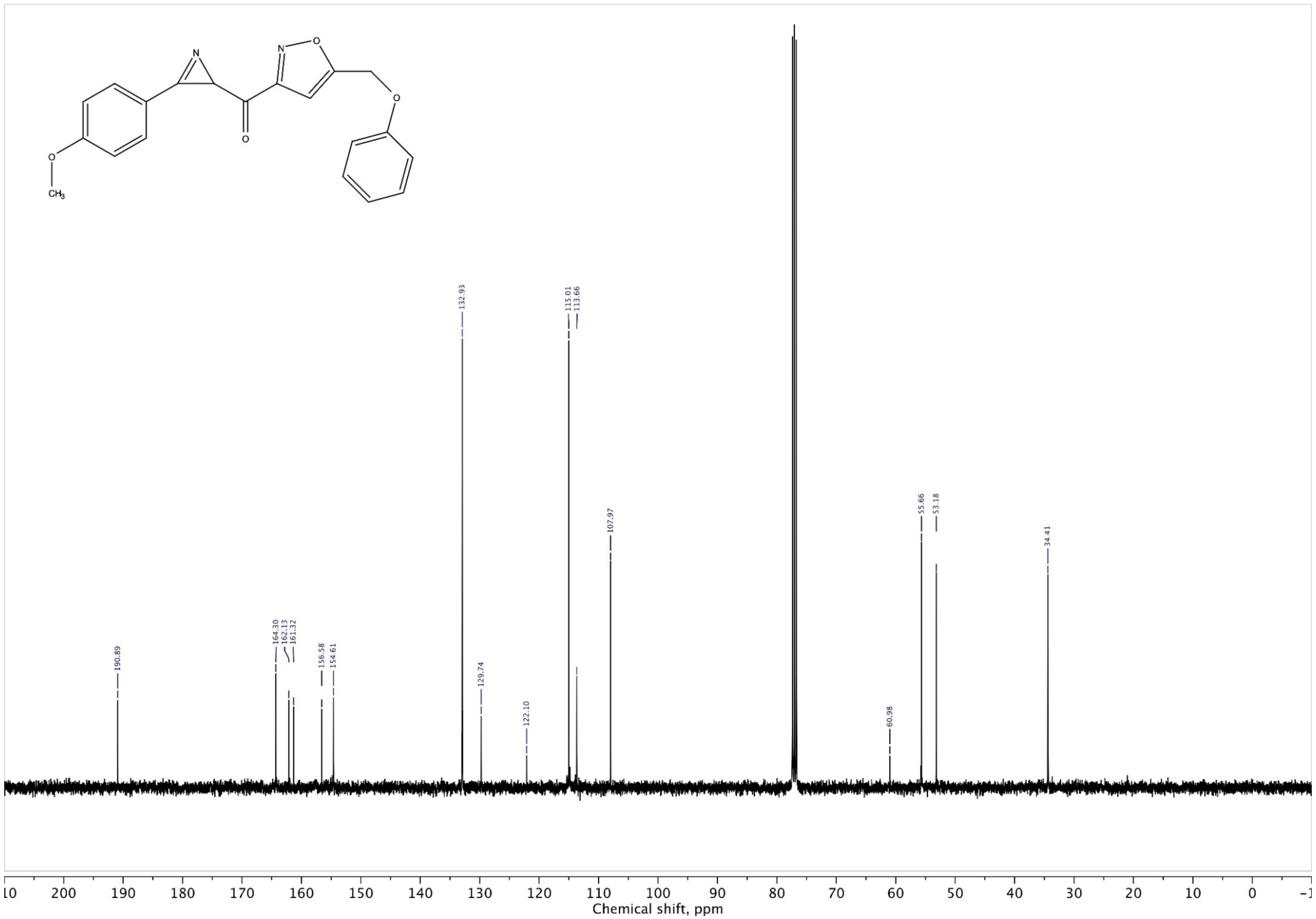

(3-(4-Methoxyphenyl)-2*H*-azirin-2-yl)(5-(phenoxymethyl)isoxazol-3-yl)methanone (3m), DEPT, $CDCl_3$, 100 MHz

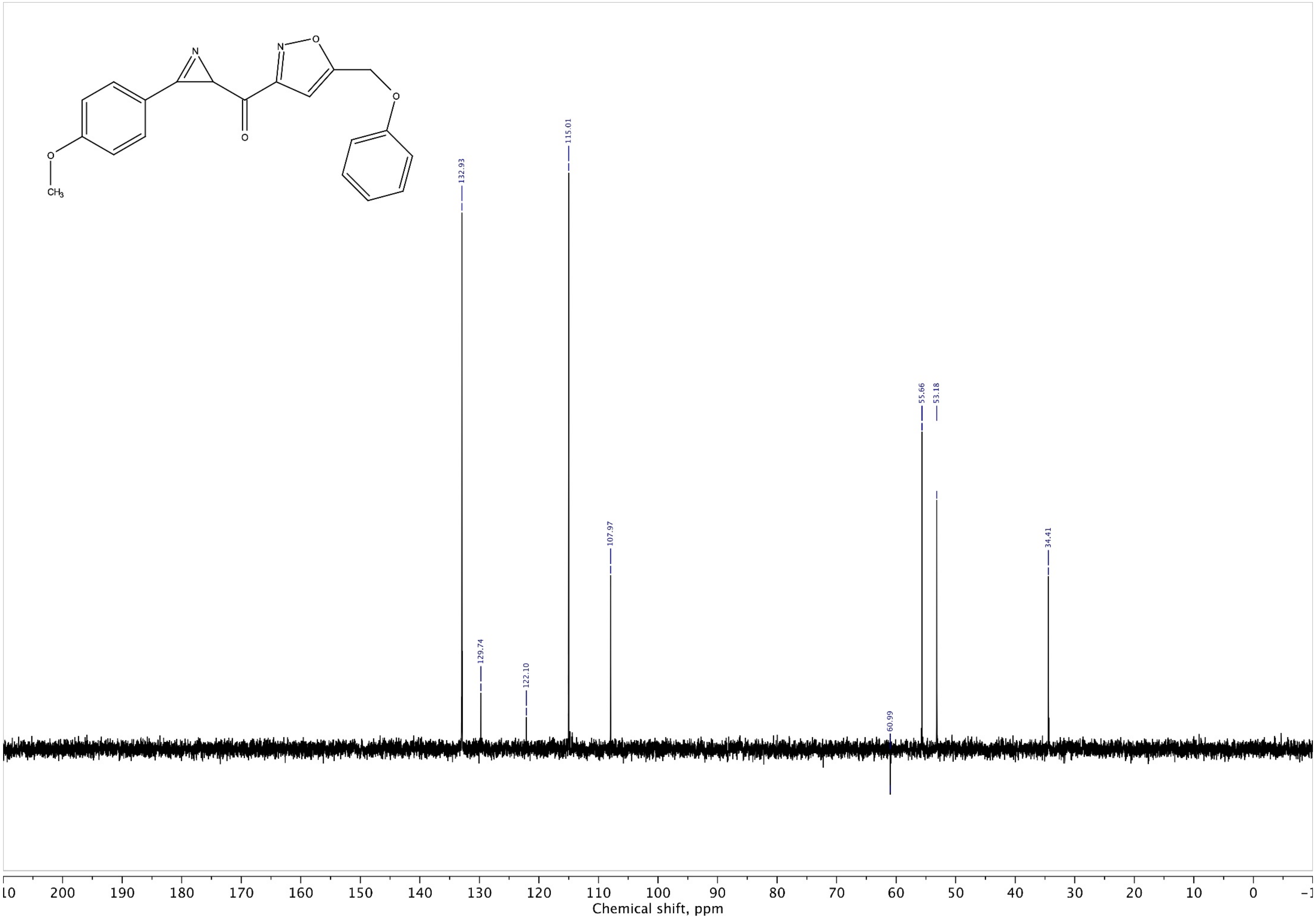

(5-(Bromomethyl)isoxazol-3-yl)(3-(3-methoxyphenyl)-2*H*-azirin-2-yl)methanone (3n), ^{1}H NMR, CDCl₃, 400 MHz

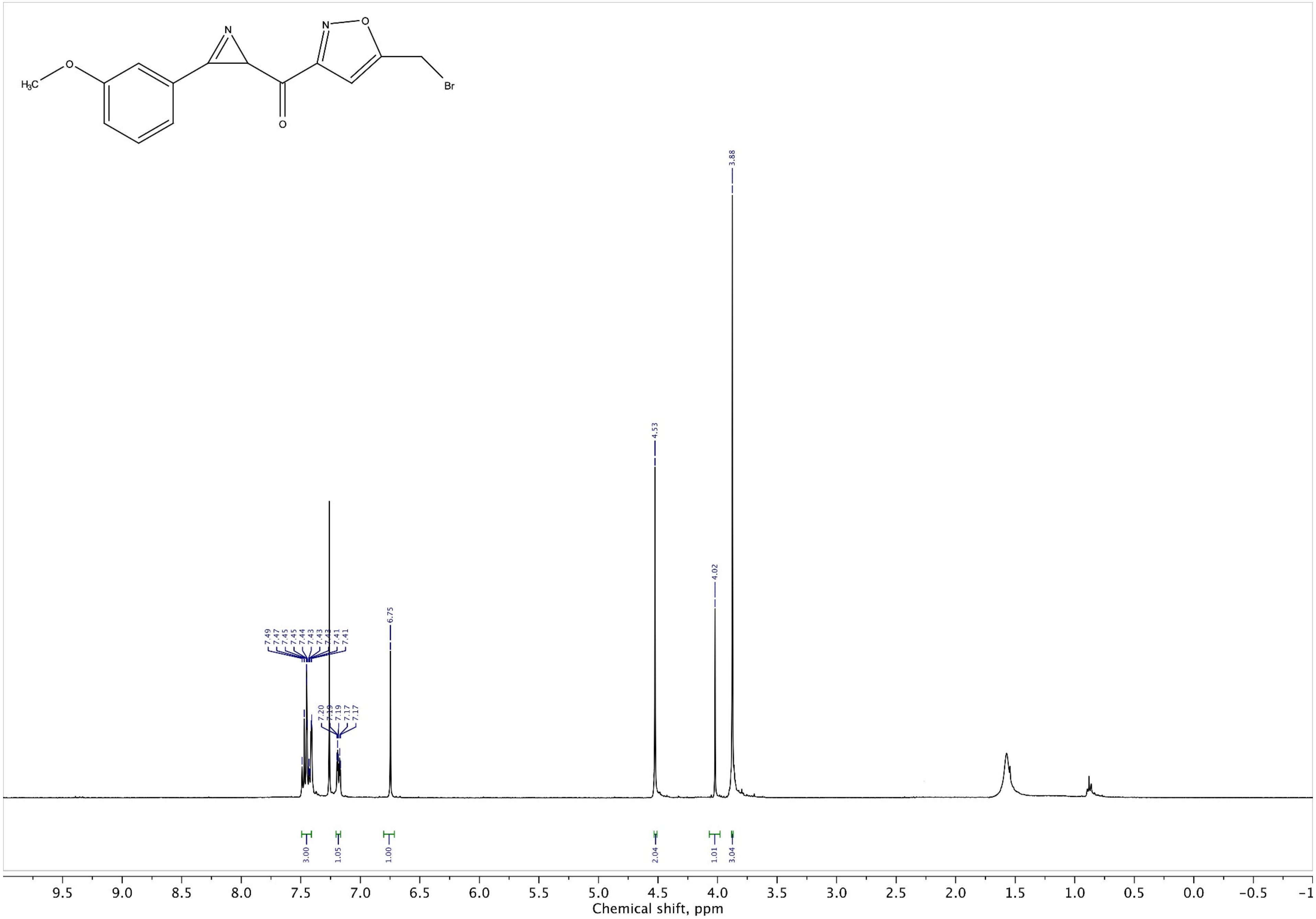

(5-(Bromomethyl)isoxazol-3-yl)(3-(3-methoxyphenyl)-2*H*-azirin-2-yl)methanone (3n), $^{13}C\{^1H\}$ NMR, $CDCl_3$, 100 MHz

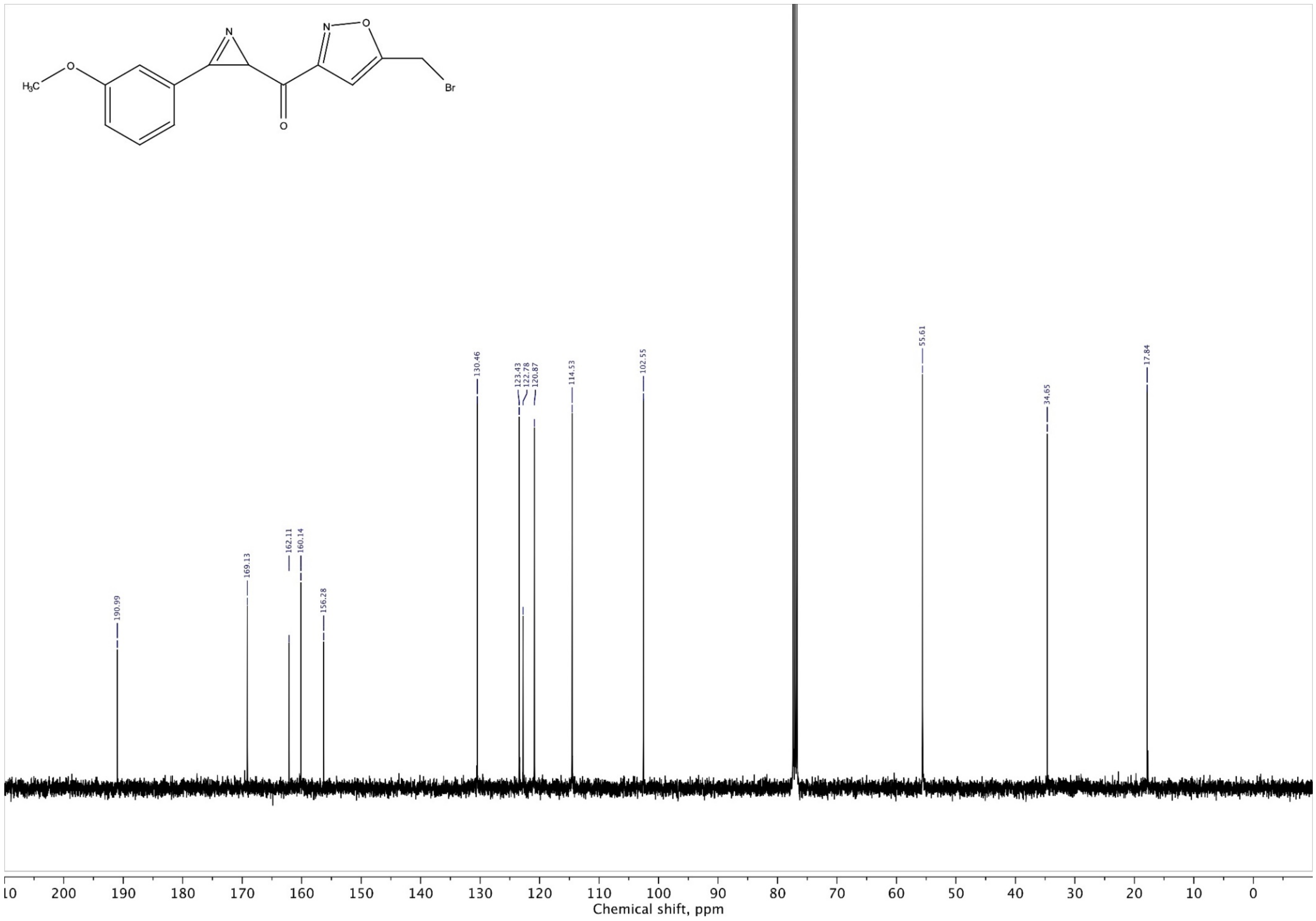

(5-(Bromomethyl)isoxazol-3-yl)(3-(3-methoxyphenyl)-2*H*-azirin-2-yl)methanone (3n), DEPT, $CDCl_3$, 100 MHz

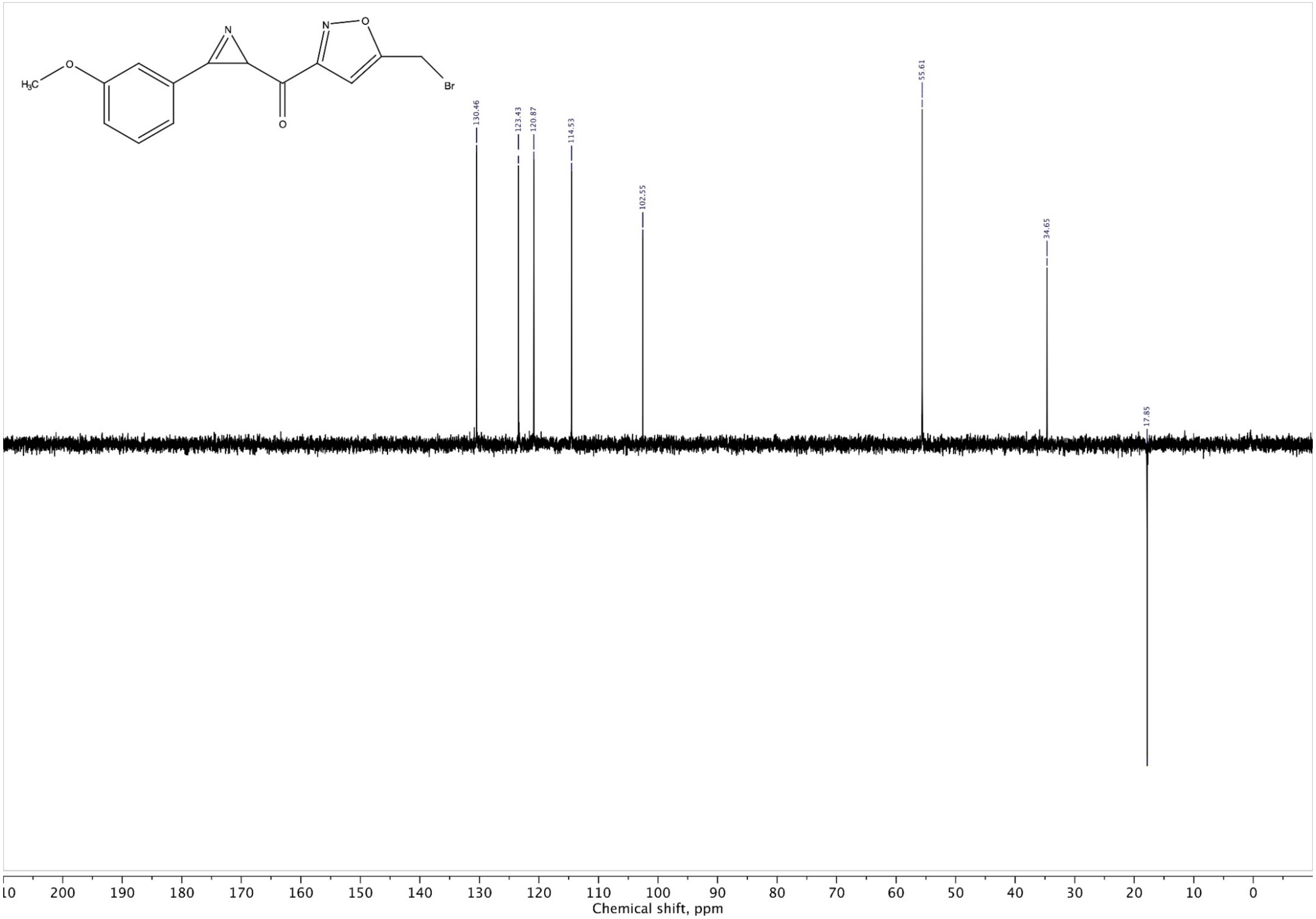

(3-(3,4-Dimethoxyphenyl)-2*H*-azirin-2-yl)(5-(trimethylsilyl)isoxazol-3-yl)methanone (3o), ^{1}H NMR, $CDCl_3$, 400 MHz

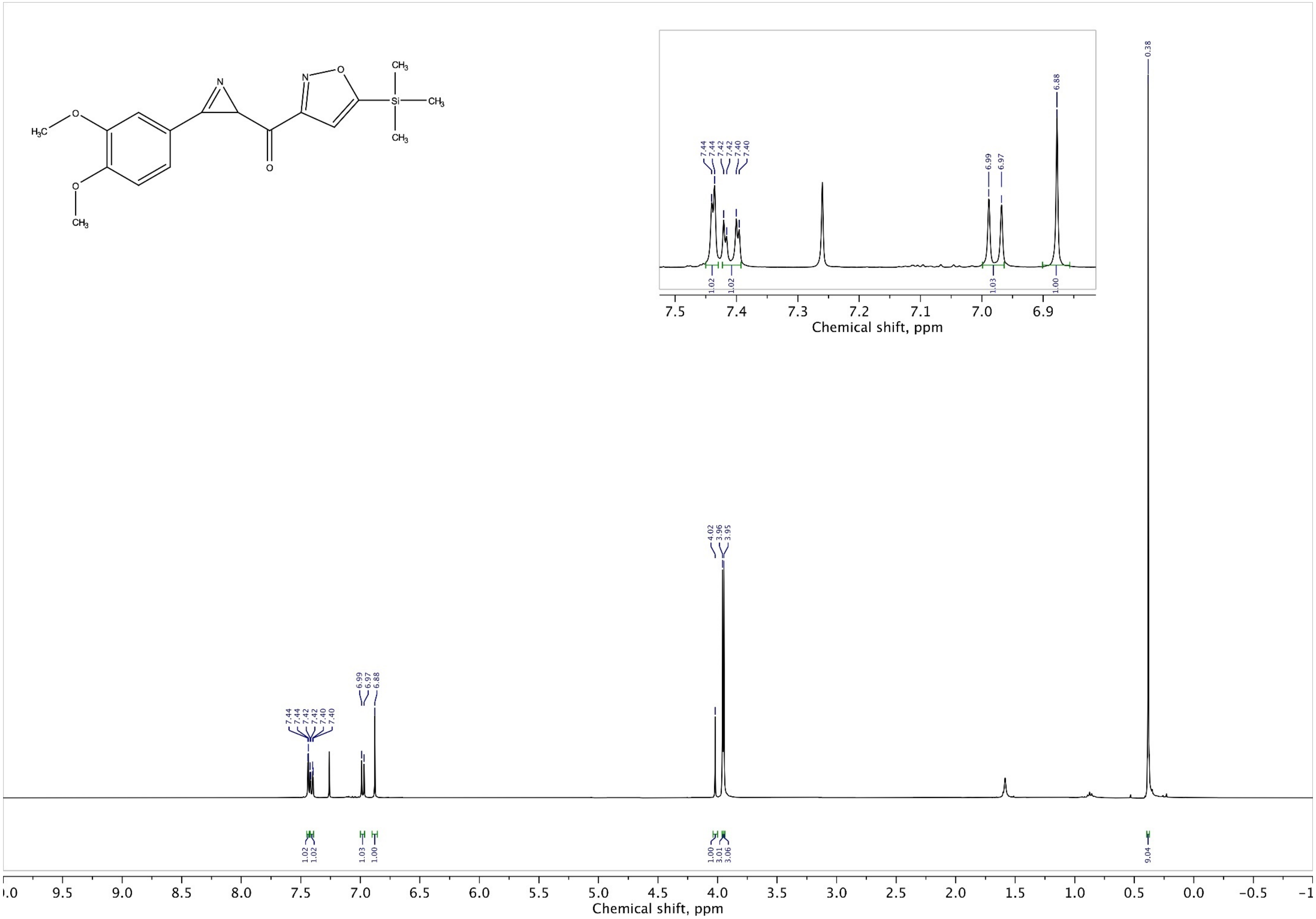

3-(3,4-Dimethoxyphenyl)-2*H*-azirin-2-yl)(5-(trimethylsilyl)isoxazol-3-yl)methanone (3o), $^{13}C\{^1H\}$ NMR, $CDCl_3$, 100 MHz

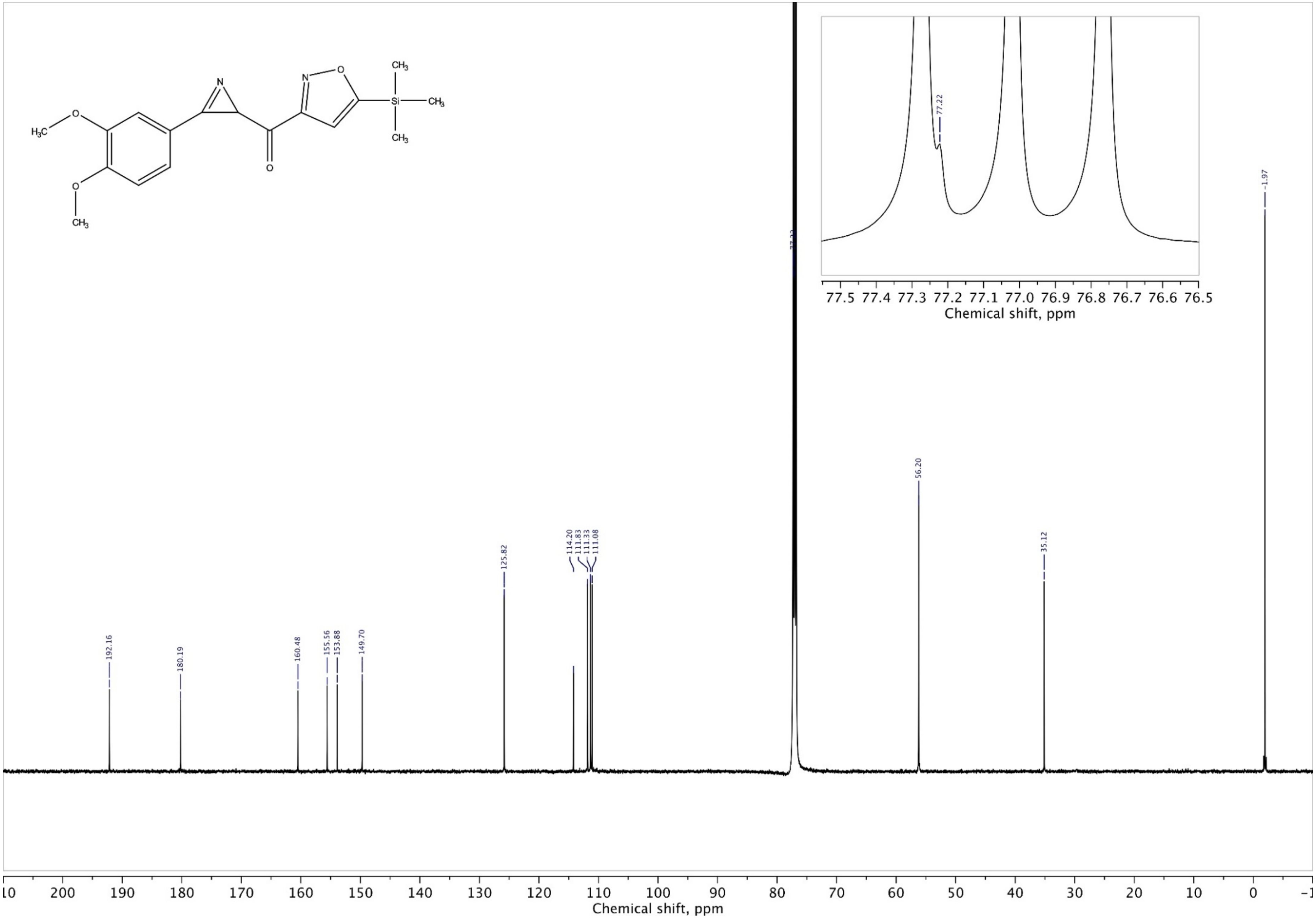

3-(3,4-Dimethoxyphenyl)-2*H*-azirin-2-yl)(5-(trimethylsilyl)isoxazol-3-yl)methanone (3o), DEPT, $CDCl_3$, 100 MHz

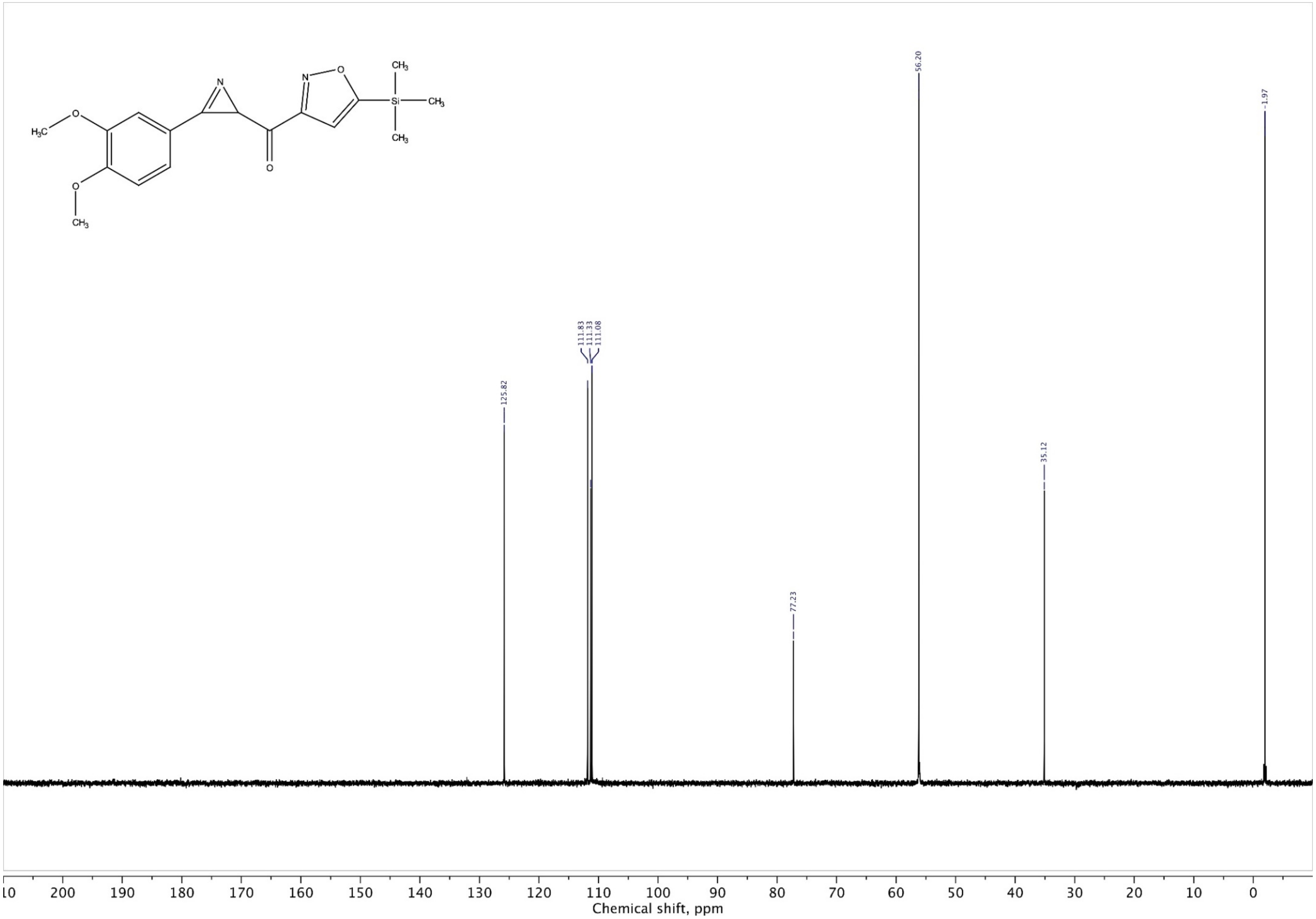

(3-(3-(4-Chlorophenyl)-2*H*-azirine-2-carbonyl)isoxazol-5-yl)methyl benzenesulfonate (3q), ^{1}H NMR, $CDCl_3$, 400 MHz

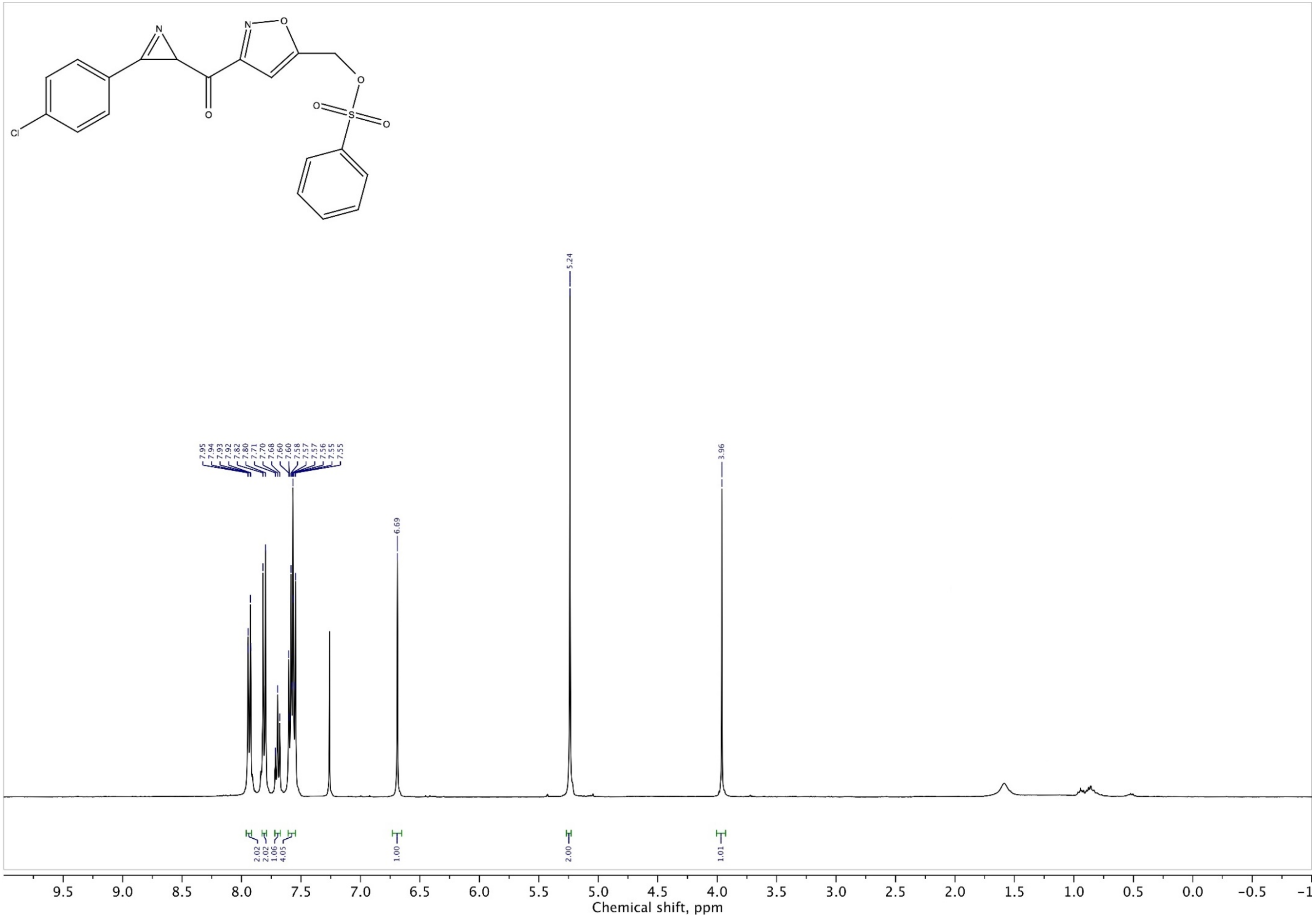

(3-(3-(4-Chlorophenyl)-2*H*-azirine-2-carbonyl)isoxazol-5-yl)methyl benzenesulfonate (3q), $^{13}C\{^1H\}$ NMR, $CDCl_3$, 100 MHz

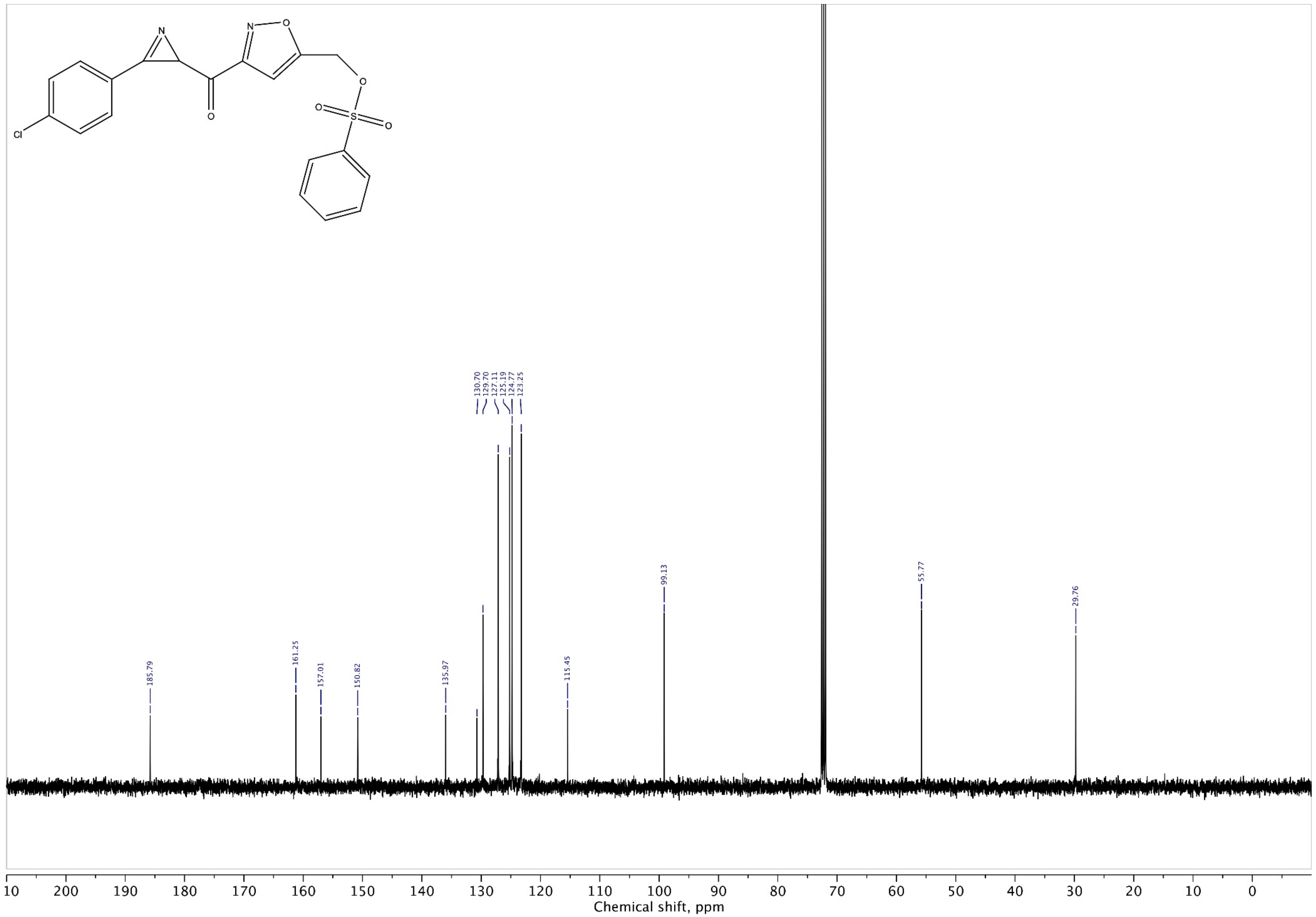

(3-(3-(4-Chlorophenyl)-2*H*-azirine-2-carbonyl)isoxazol-5-yl)methyl benzenesulfonate (3q), DEPT, $CDCl_3$, 100 MHz

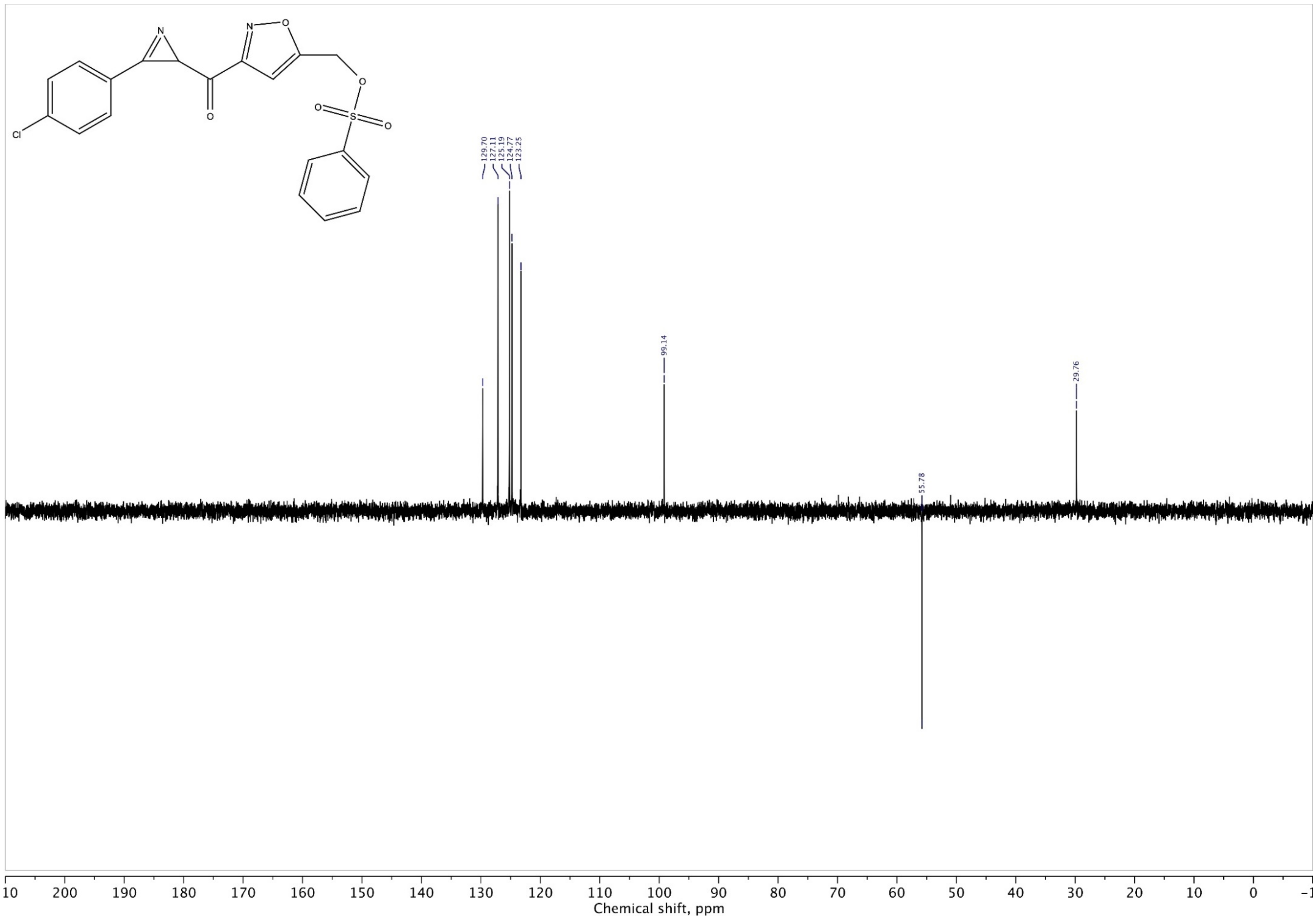

(3-(2-Bromophenyl)-2*H*-azirin-2-yl)(5-(chloromethyl)isoxazol-3-yl)methanone (3r), ^{1}H NMR, $CDCl_3$, 400 MHz

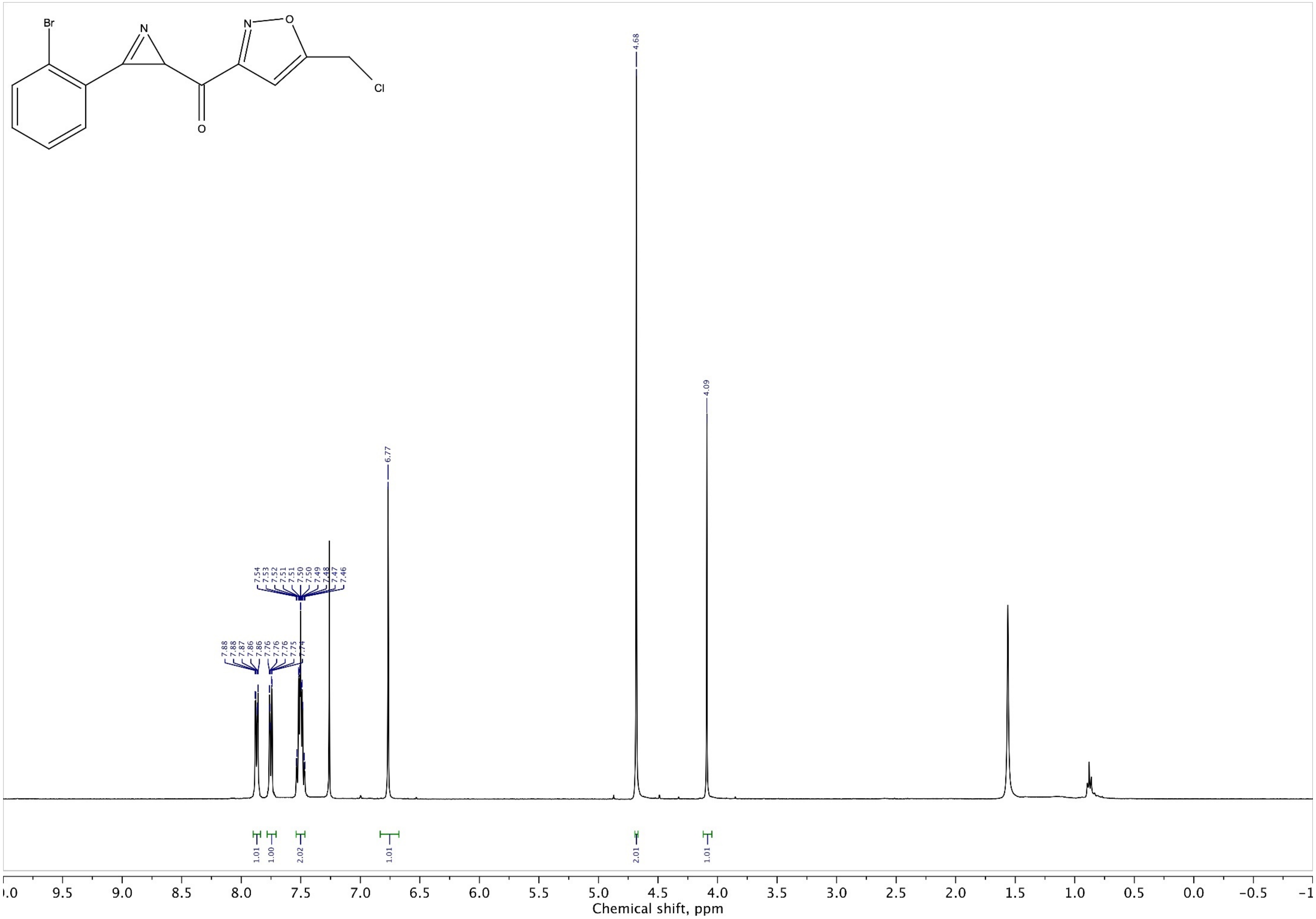

(3-(2-Bromophenyl)-2*H*-azirin-2-yl)(5-(chloromethyl)isoxazol-3-yl)methanone (3r), $^{13}C\{^1H\}$ NMR, $CDCl_3$, 100 MHz

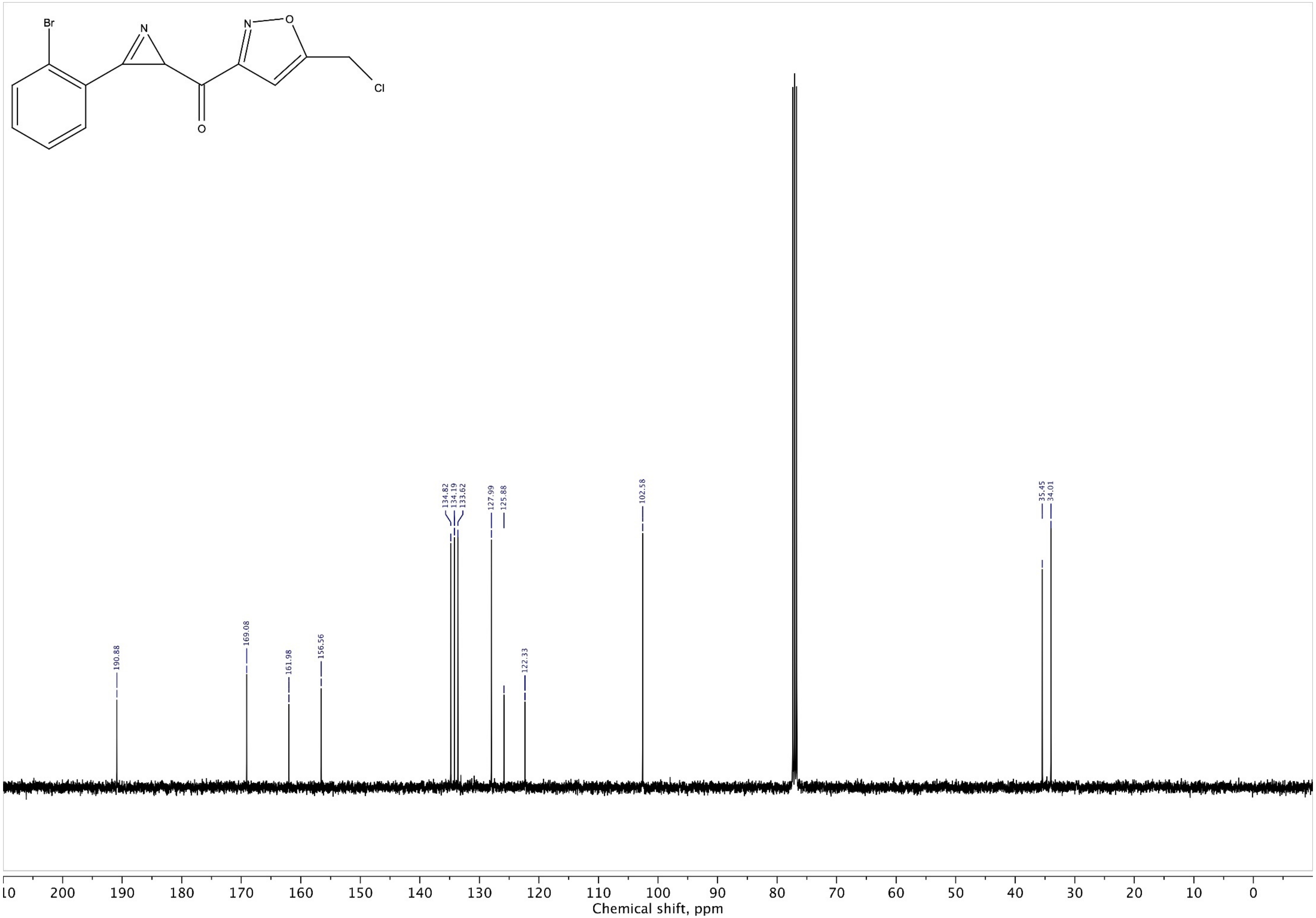

(3-(2-Bromophenyl)-2*H*-azirin-2-yl)(5-(chloromethyl)isoxazol-3-yl)methanone (3r), DEPT, $CDCl_3$, 100 MHz

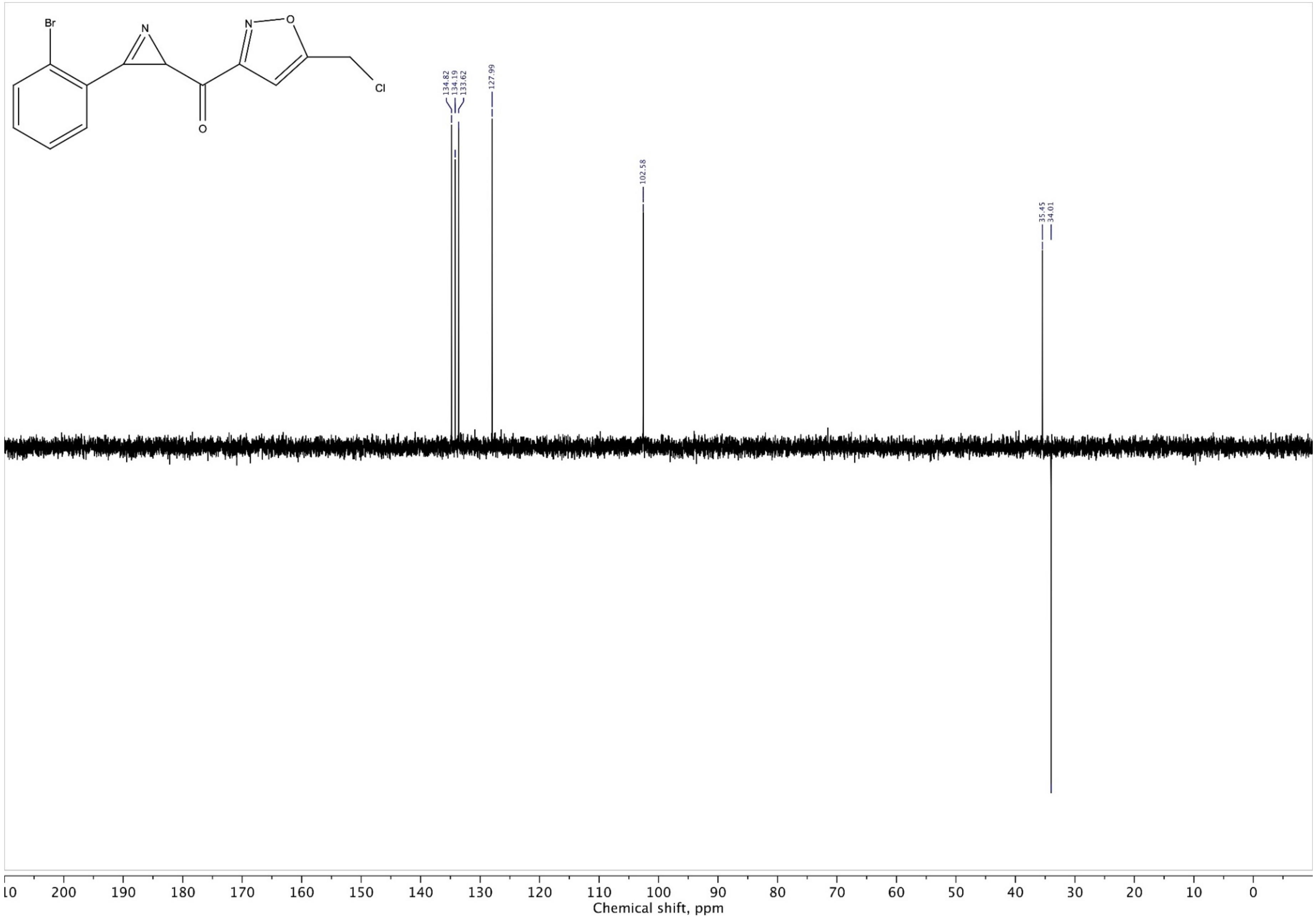

(5-(Hydroxymethyl)isoxazol-3-yl)(3-(thiophen-2-yl)-2*H*-azirin-2-yl)methanone (3s), ^{1}H NMR, $CDCl_3$, 400 MHz

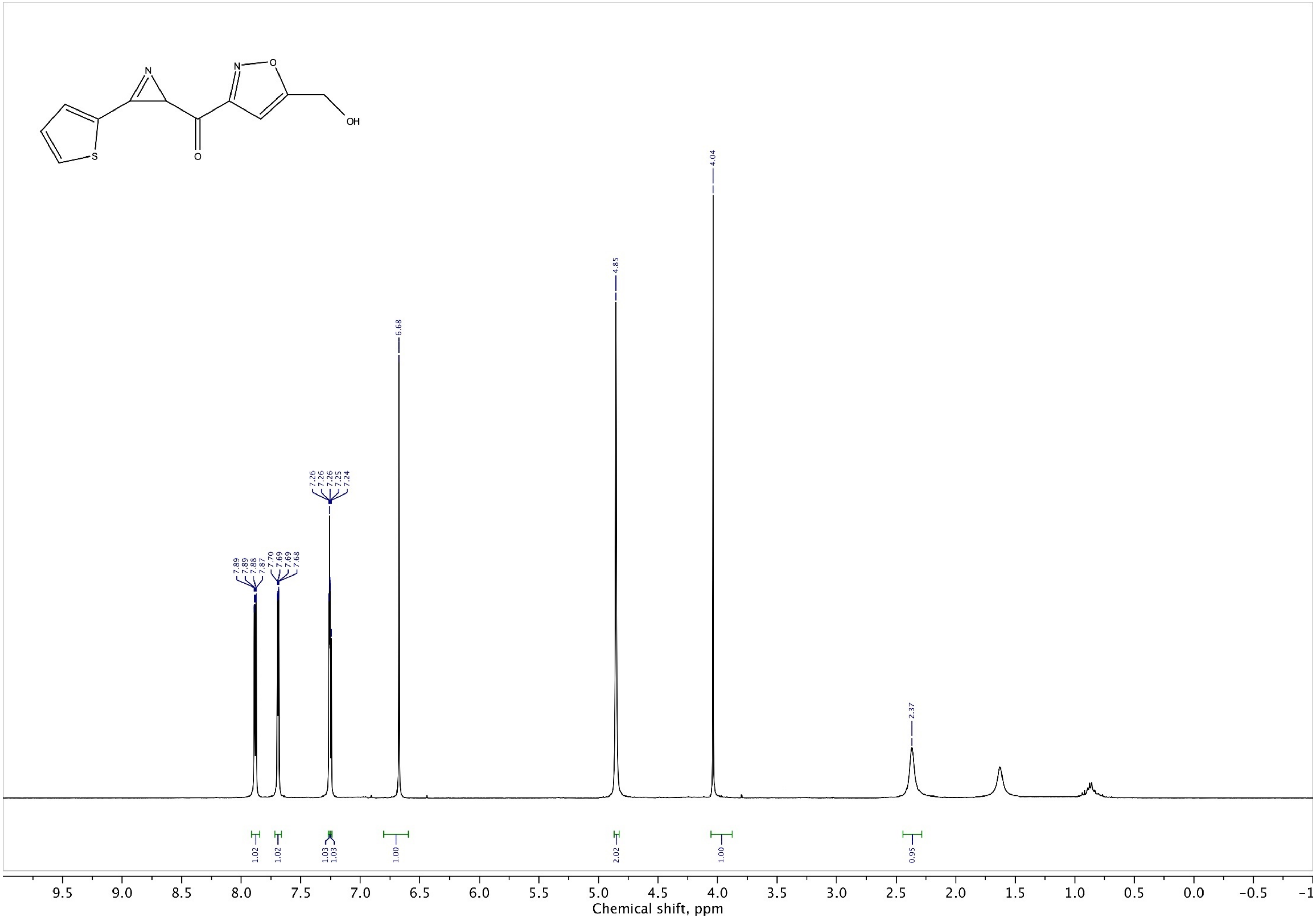

(5-(Hydroxymethyl)isoxazol-3-yl)(3-(thiophen-2-yl)-2*H*-azirin-2-yl)methanone (3s), $^{13}C\{^1H\}$ NMR, $CDCl_3$, 100 MHz

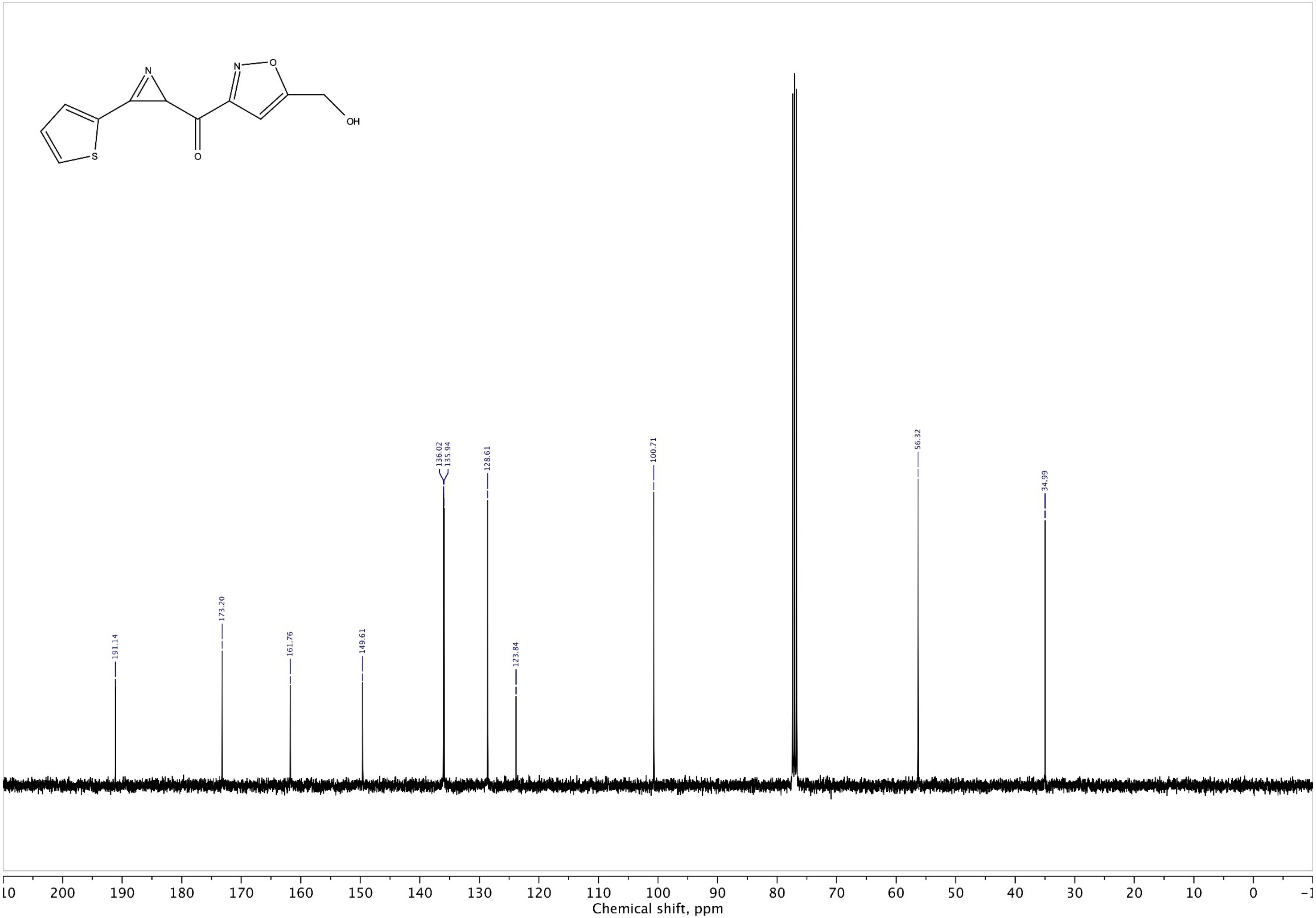

(5-(Hydroxymethyl)isoxazol-3-yl)(3-(thiophen-2-yl)-2*H*-azirin-2-yl)methanone (3s), DEPT, $CDCl_3$, 100 MHz

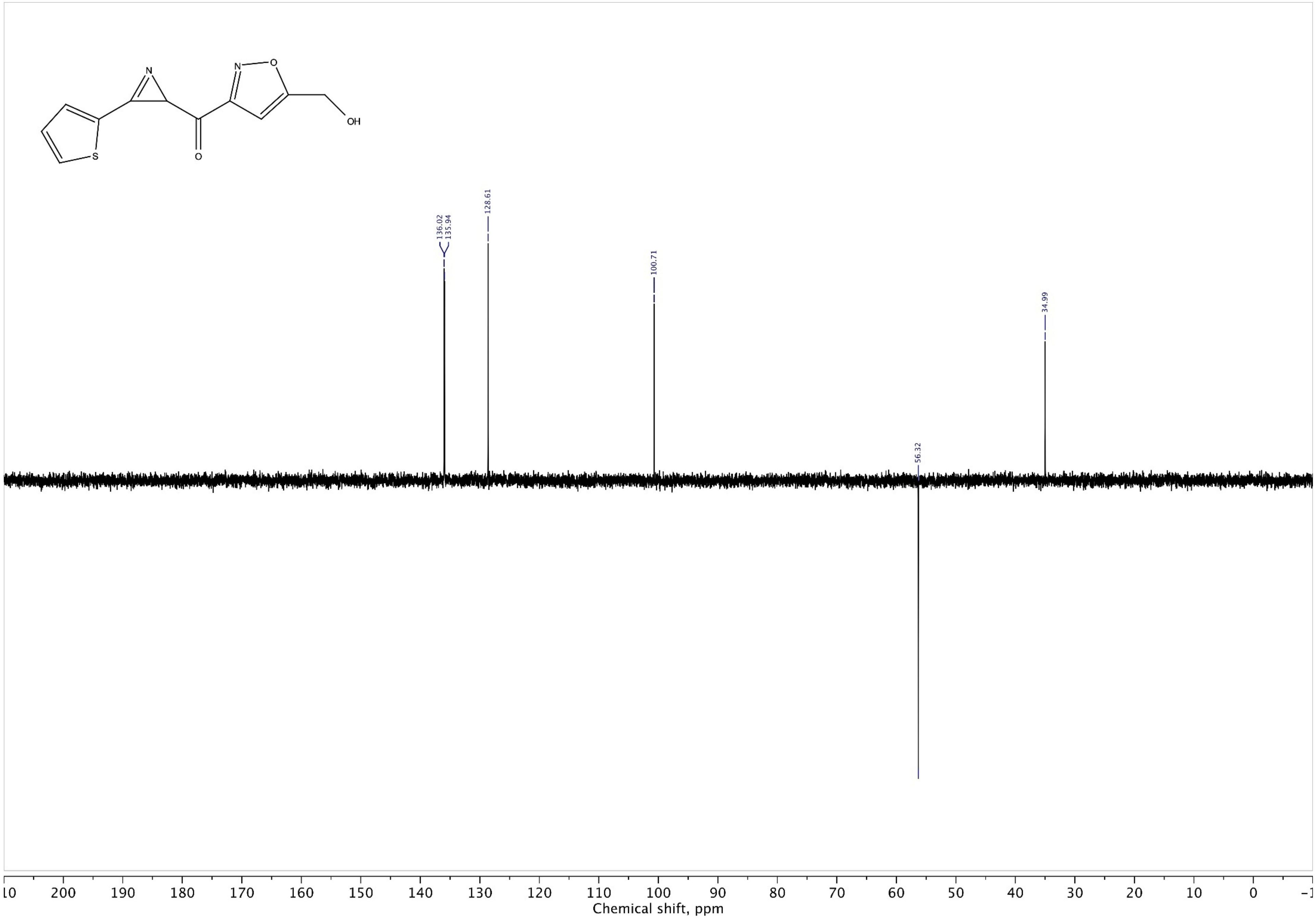

(5-(Chloromethyl)isoxazol-3-yl)(3-(thiophen-2-yl)-2*H*-azirin-2-yl)methanone (3t), ^{1}H NMR, CDCl$_3$, 400 MHz

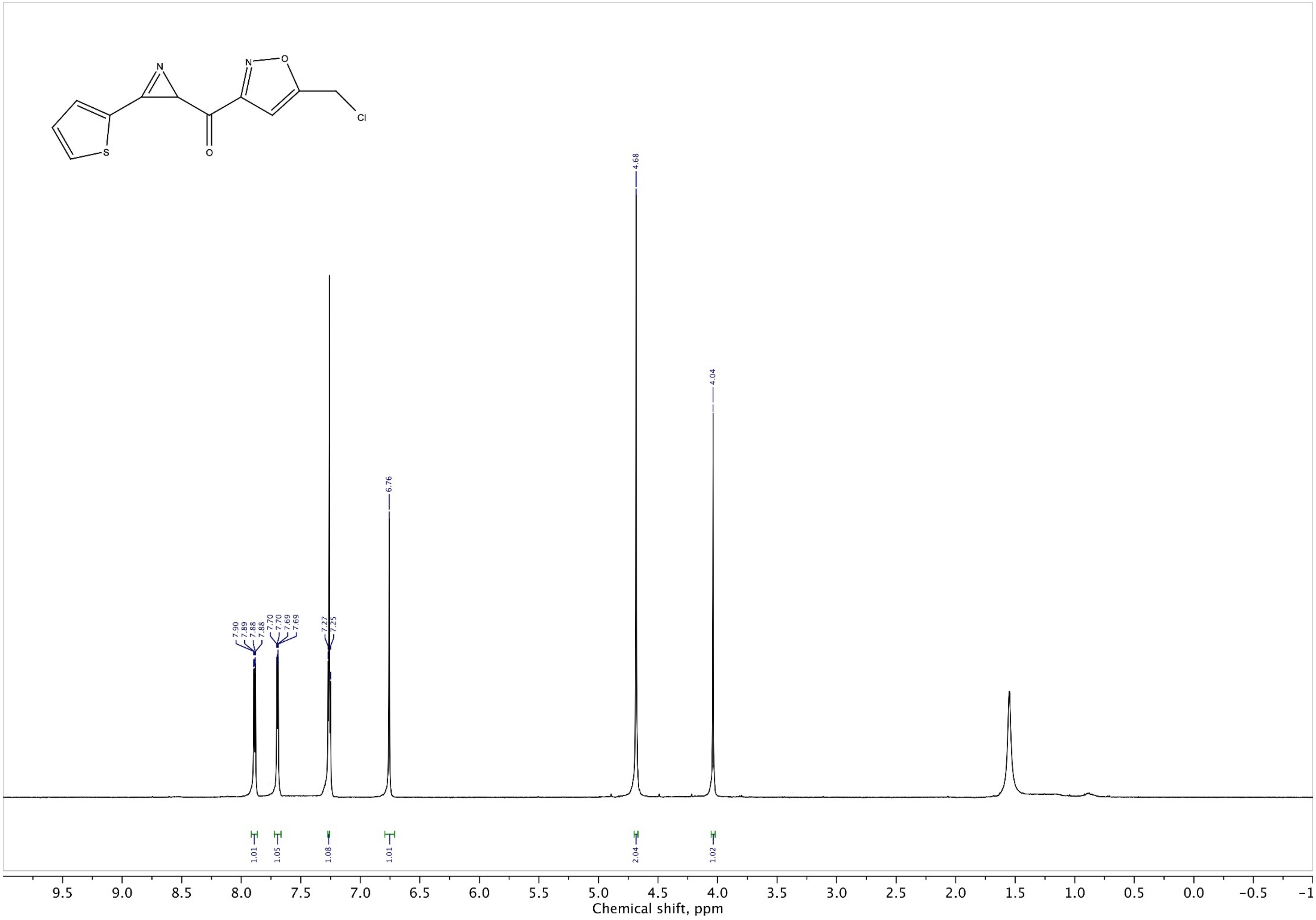

(5-(Chloromethyl)isoxazol-3-yl)(3-(thiophen-2-yl)-2*H*-azirin-2-yl)methanone (3t), $^{13}C\{^{1}H\}$ NMR, $CDCl_3$, 100 MHz

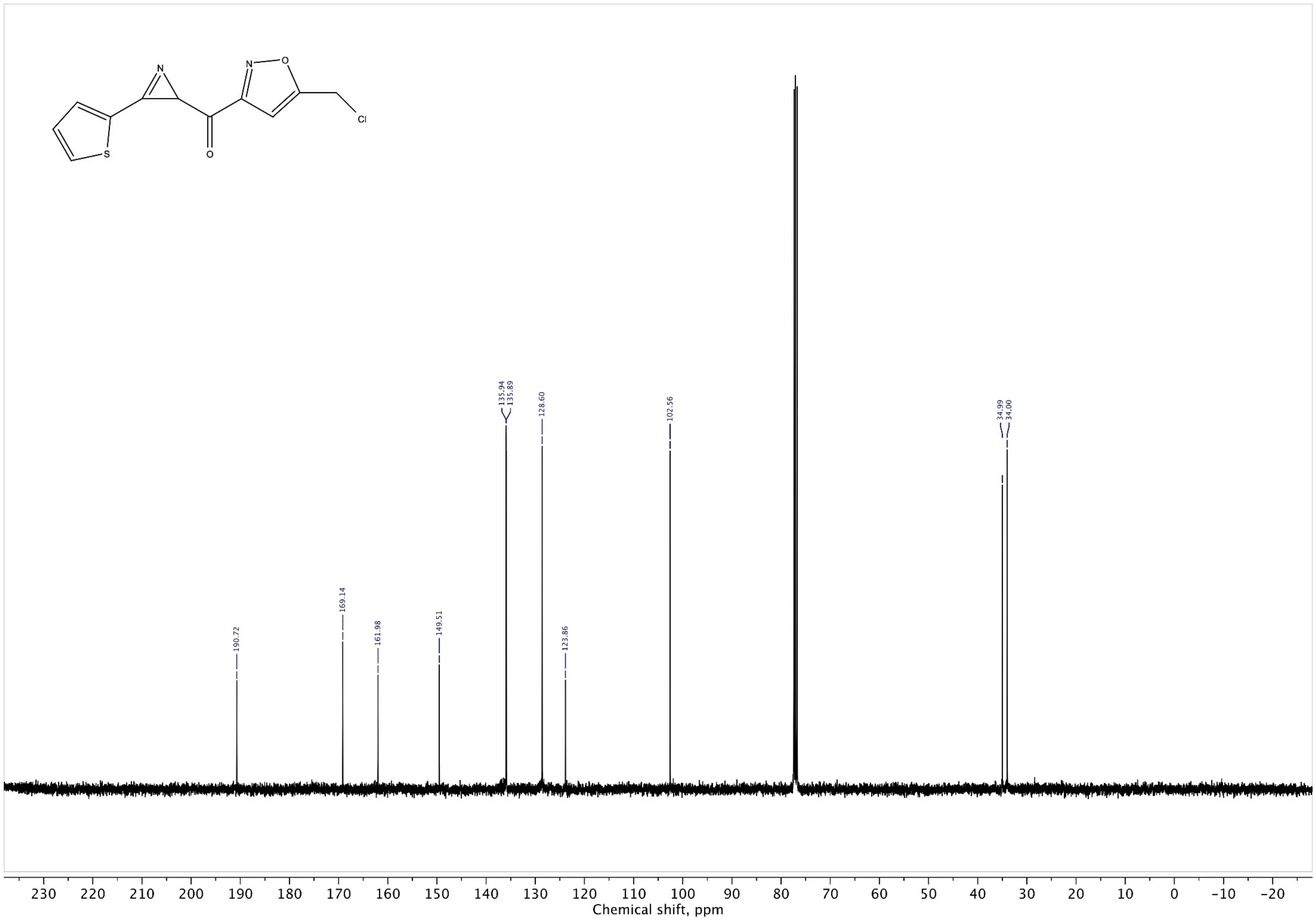

(5-(Chloromethyl)isoxazol-3-yl)(3-(thiophen-2-yl)-2*H*-azirin-2-yl)methanone (3t), DEPT NMR, $CDCl_3$, 100 MHz

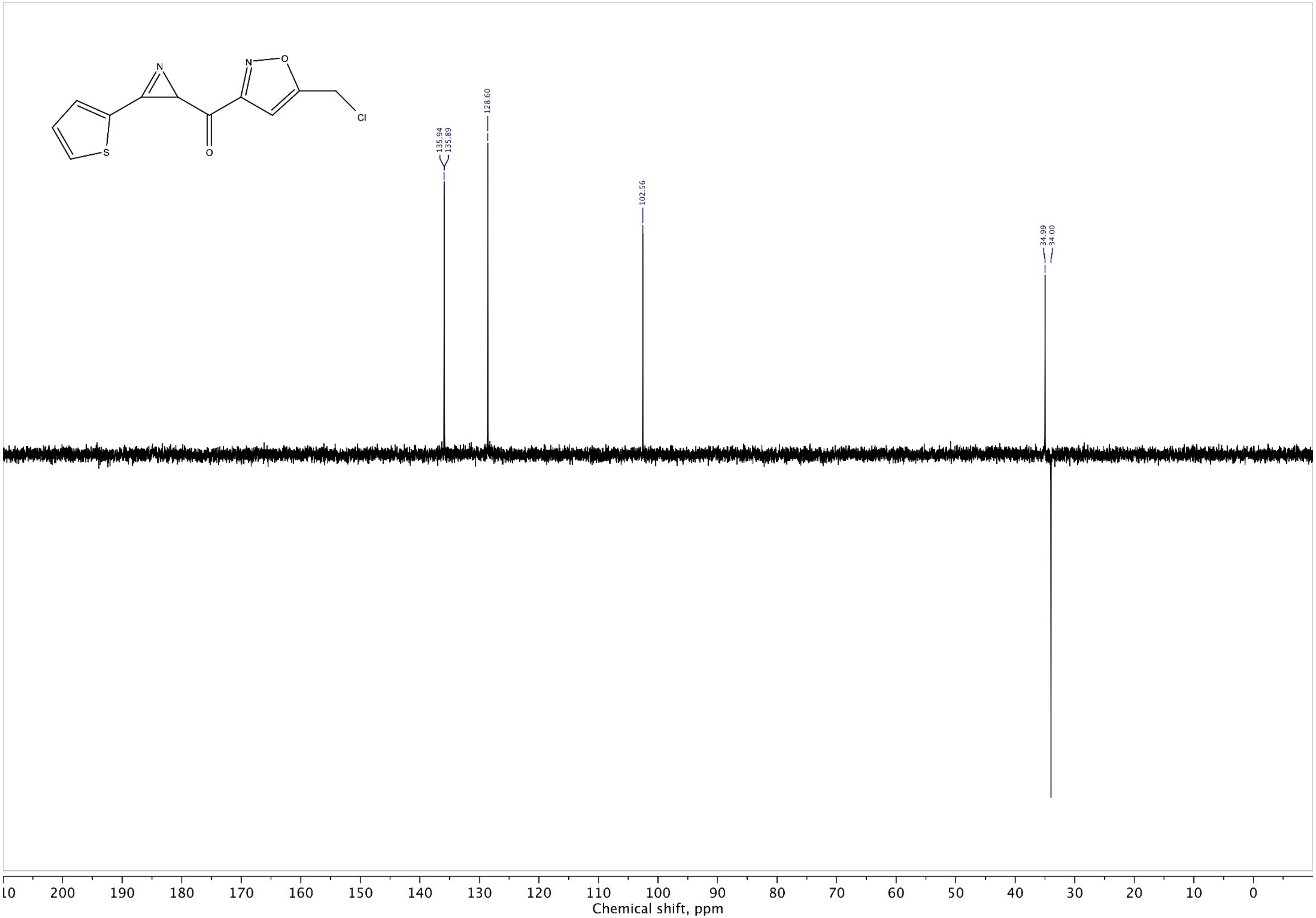

Methyl 3-(3-(tert-butyl)-2*H*-azirine-2-carbonyl)isoxazole-5-carboxylate (3u), ^{1}H NMR, DMSO-d_6, 400 MHz

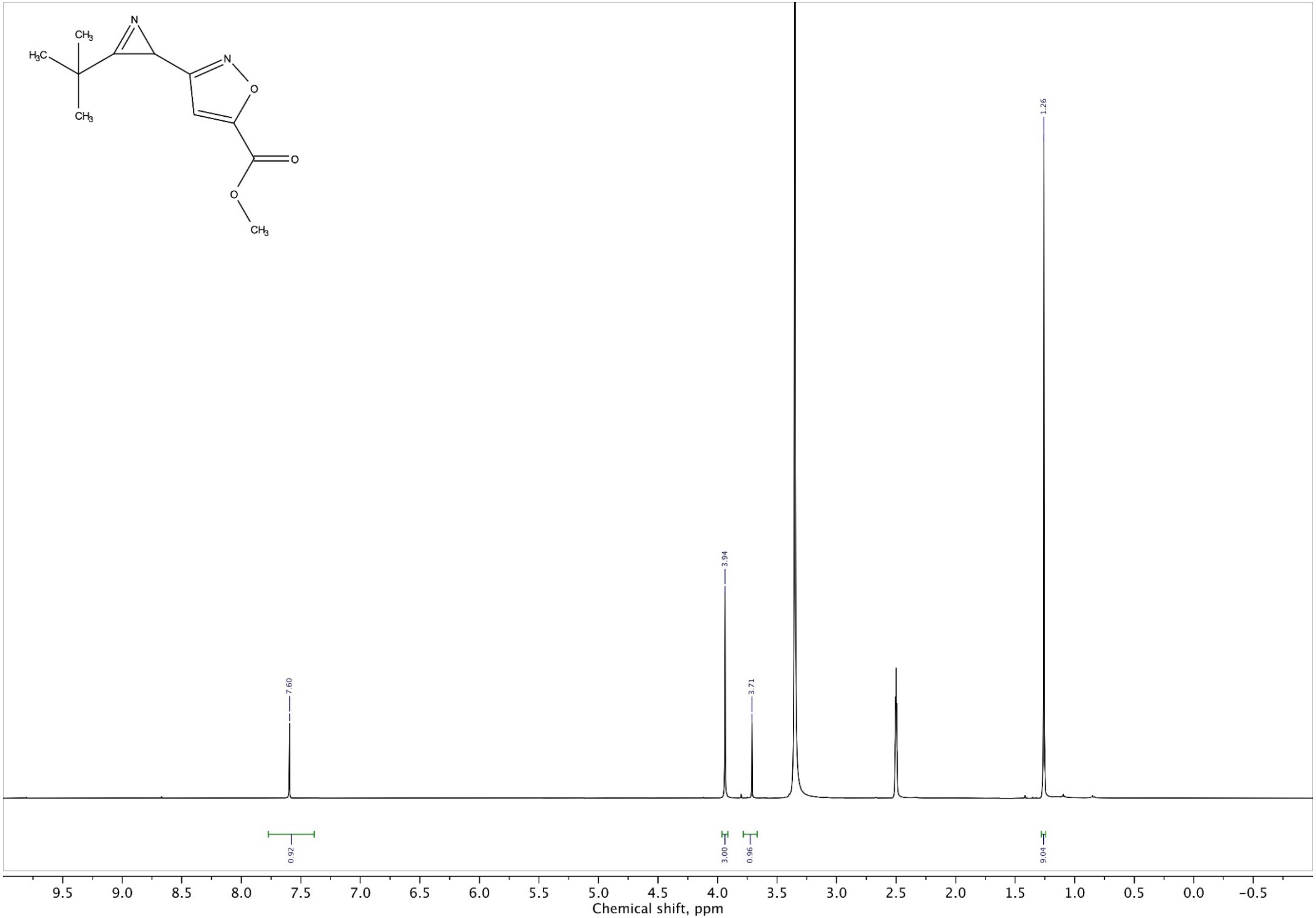

Methyl 3-(3-(tert-butyl)-2*H*-azirine-2-carbonyl)isoxazole-5-carboxylate (3u), $^{13}C\{^{1}H\}$ NMR, DMSO-d_6, 100 MHz

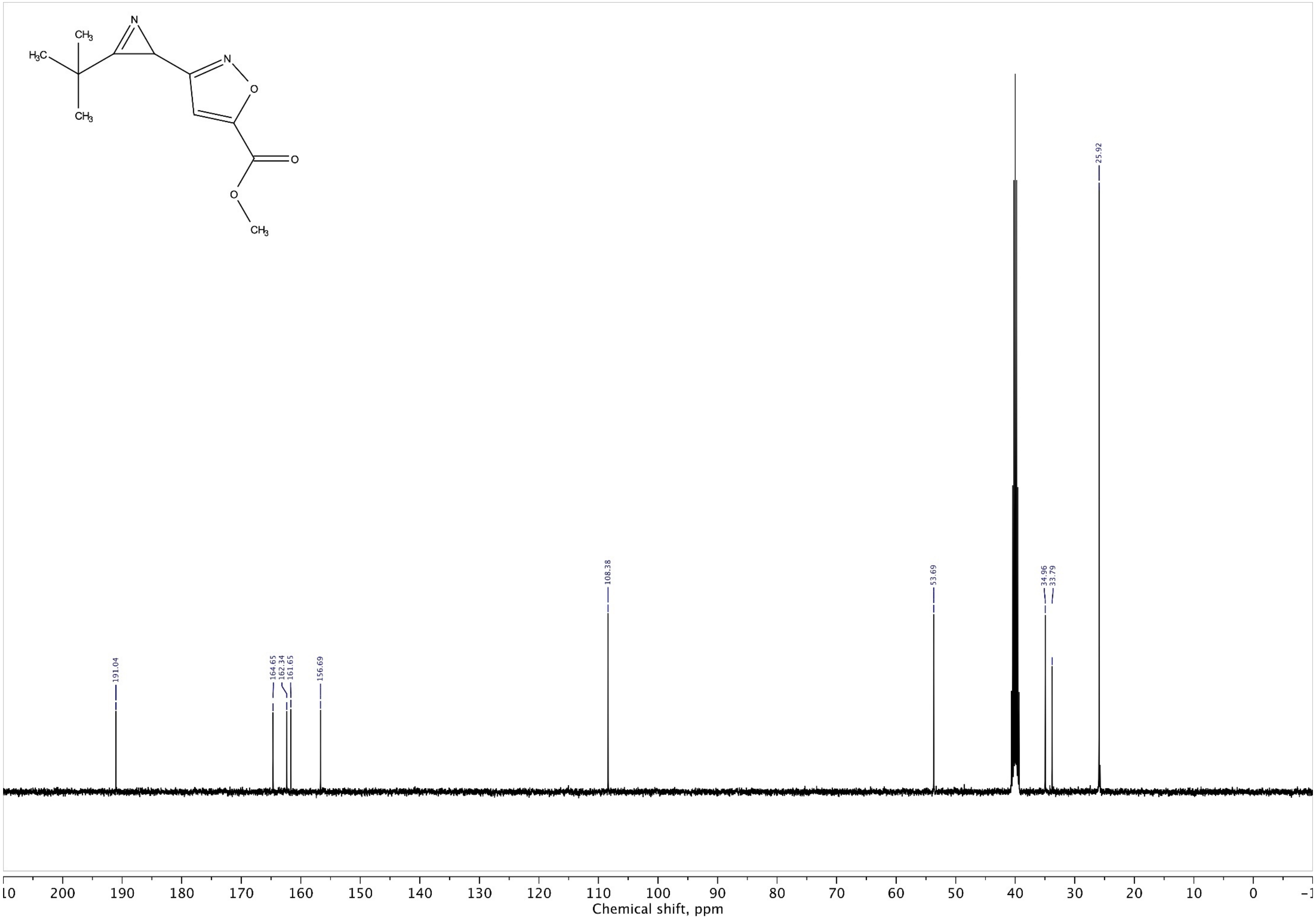

Methyl 3-(3-(tert-butyl)-2*H*-azirine-2-carbonyl)isoxazole-5-carboxylate (3u), DEPT, DMSO-d_6, 100 MHz

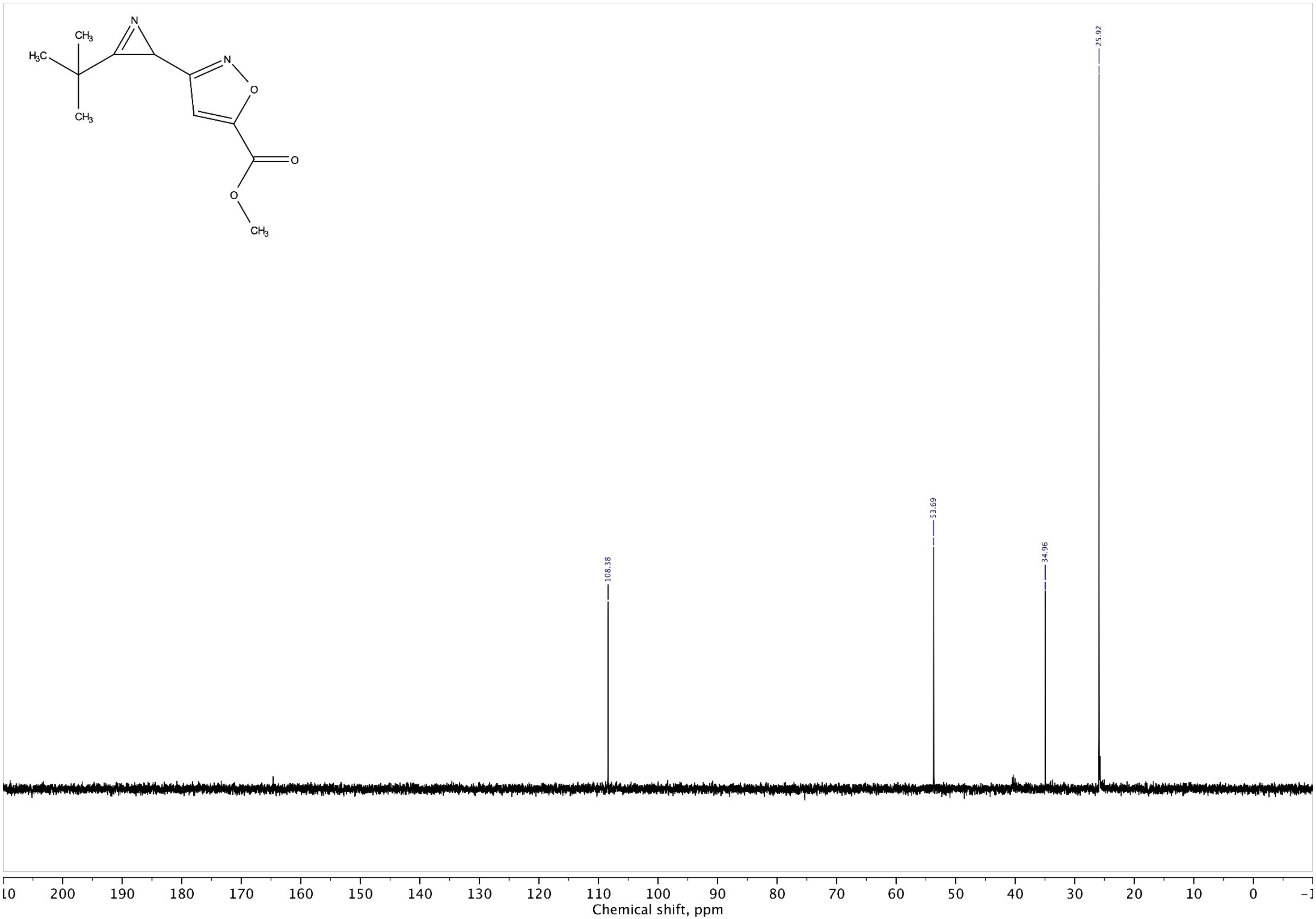

Dimethyl 3-(3-phenyl-2*H*-azirine-2-carbonyl)isoxazole-4,5-dicarboxylate (3w), ^{1}H NMR, CDCl$_3$, 400 MHz

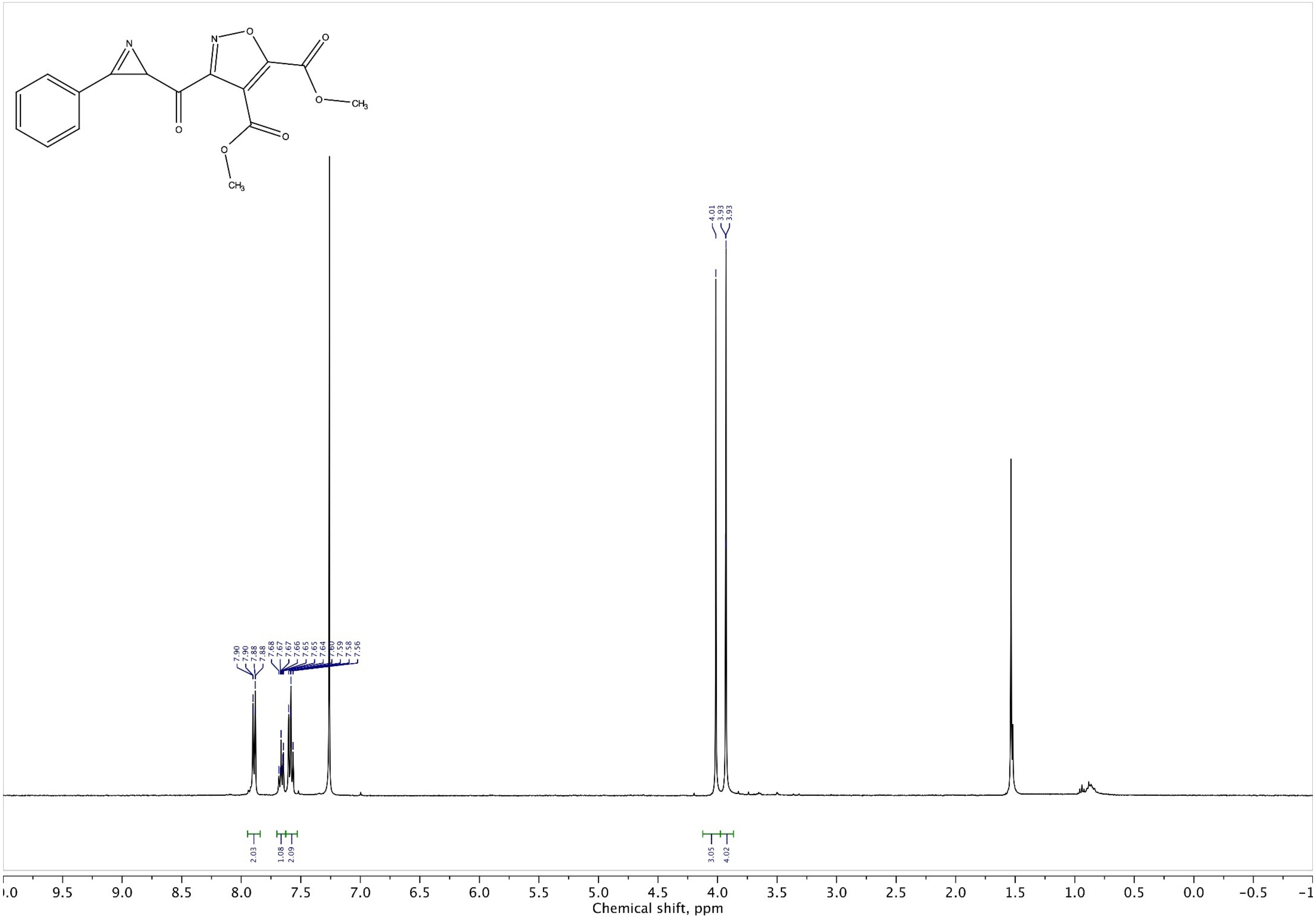

Dimethyl 3-(3-phenyl-2*H*-azirine-2-carbonyl)isoxazole-4,5-dicarboxylate (3w), $^{13}C\{^{1}H\}$ NMR, $CDCl_3$, 100 MHz

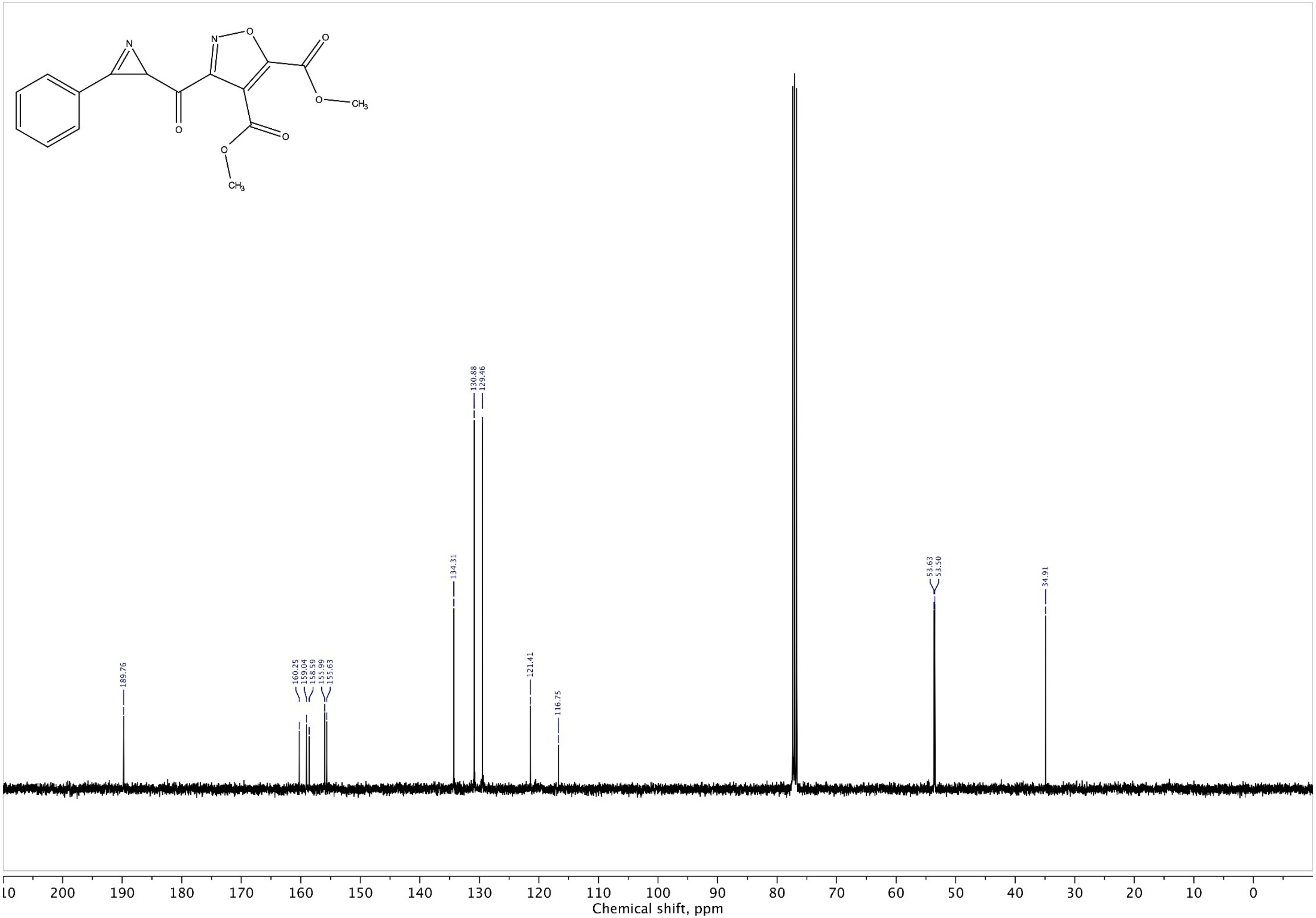

Dimethyl 3-(3-phenyl-2*H*-azirine-2-carbonyl)isoxazole-4,5-dicarboxylate (3w), DEPT, $CDCl_3$, 100 MHz

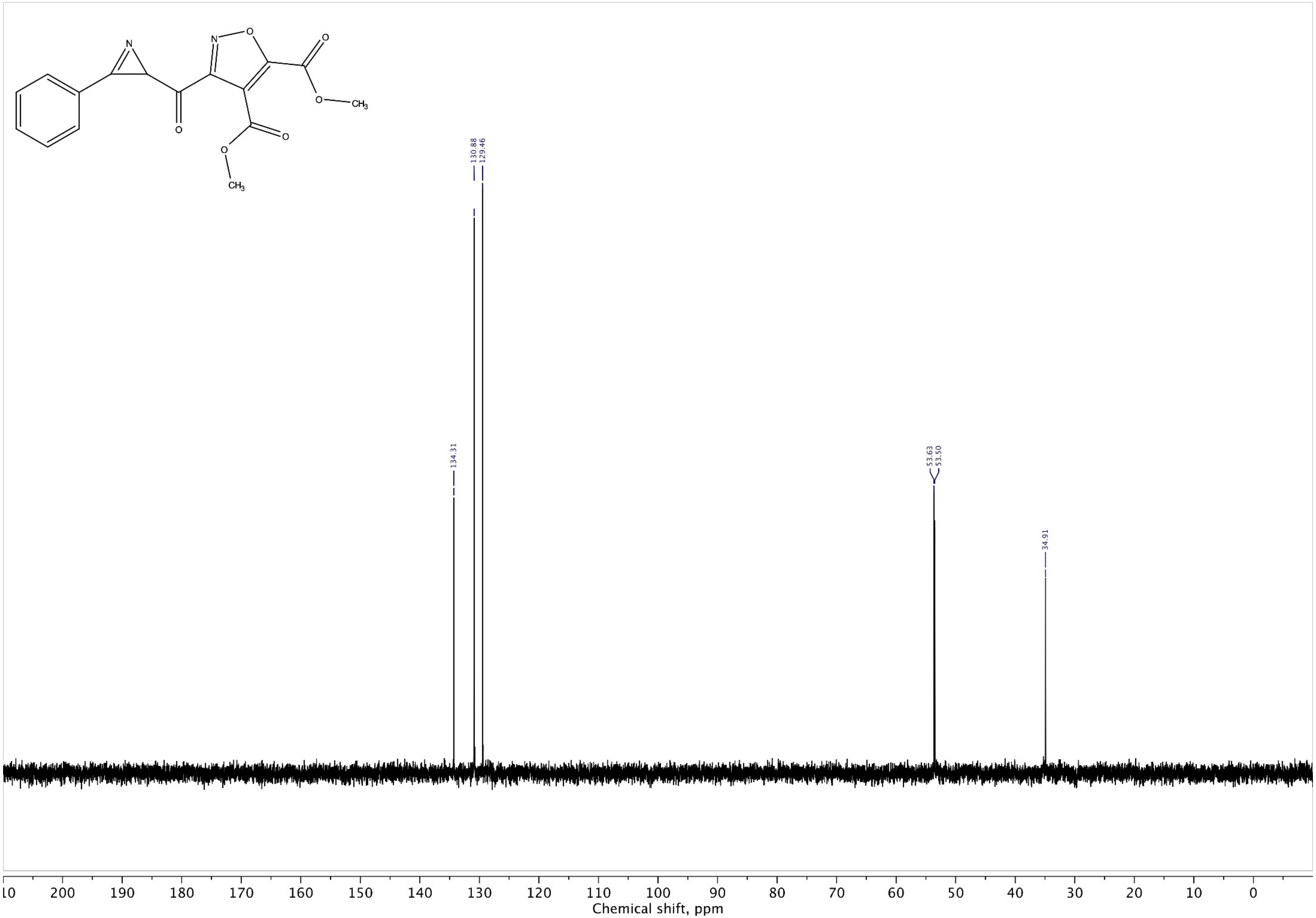

(3-(4-Chlorophenyl)-2*H*-azirin-2-yl)(5-phenylisoxazol-3-yl)methanone (3x), ^{1}H NMR, $CDCl_3$, 400 MHz

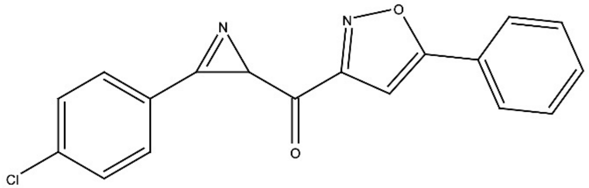

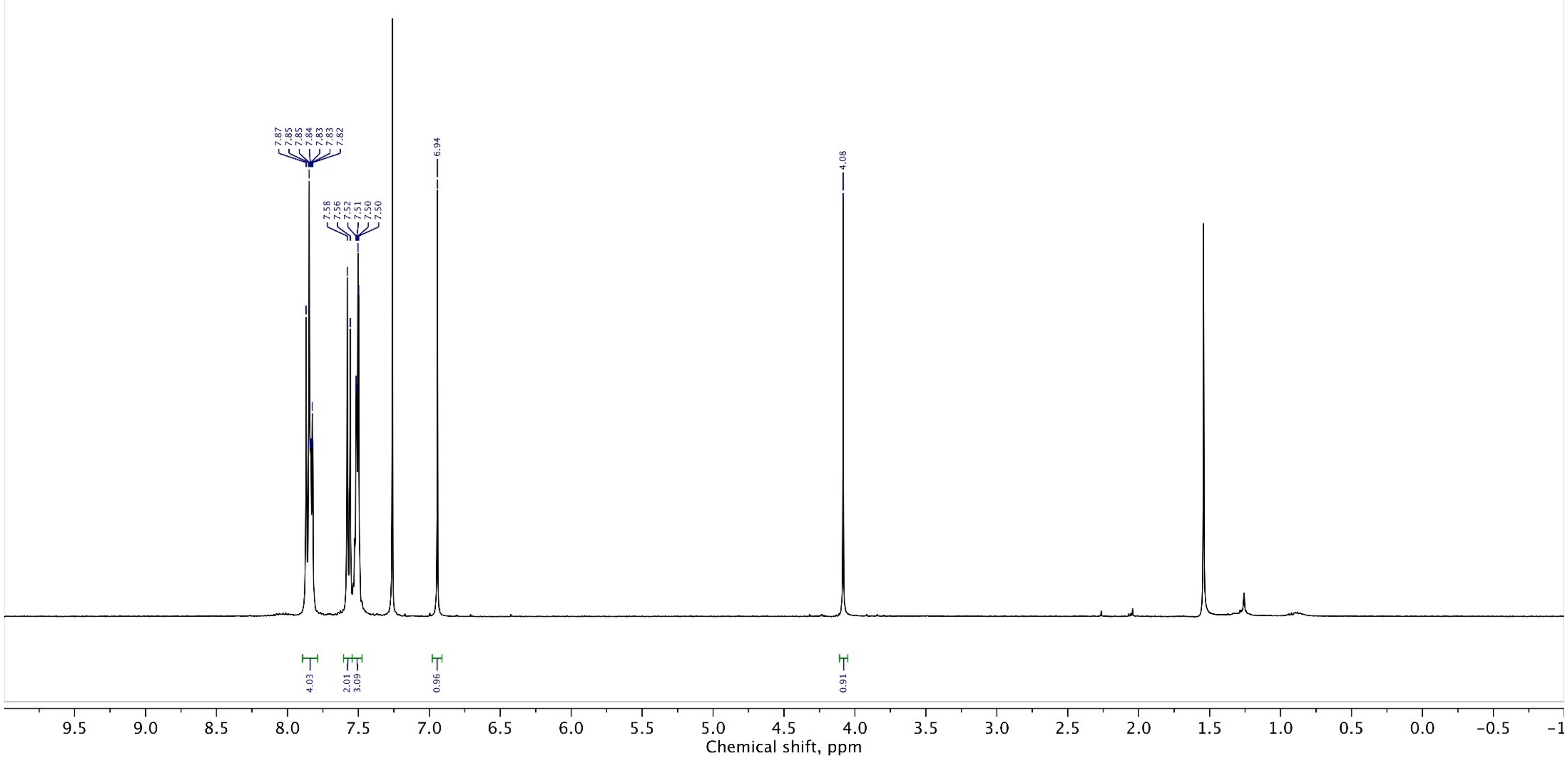

(3-(4-Chlorophenyl)-2*H*-azirin-2-yl)(5-phenylisoxazol-3-yl)methanone (3x), $^{13}C\{^1H\}$ NMR, $CDCl_3$, 100 MHz

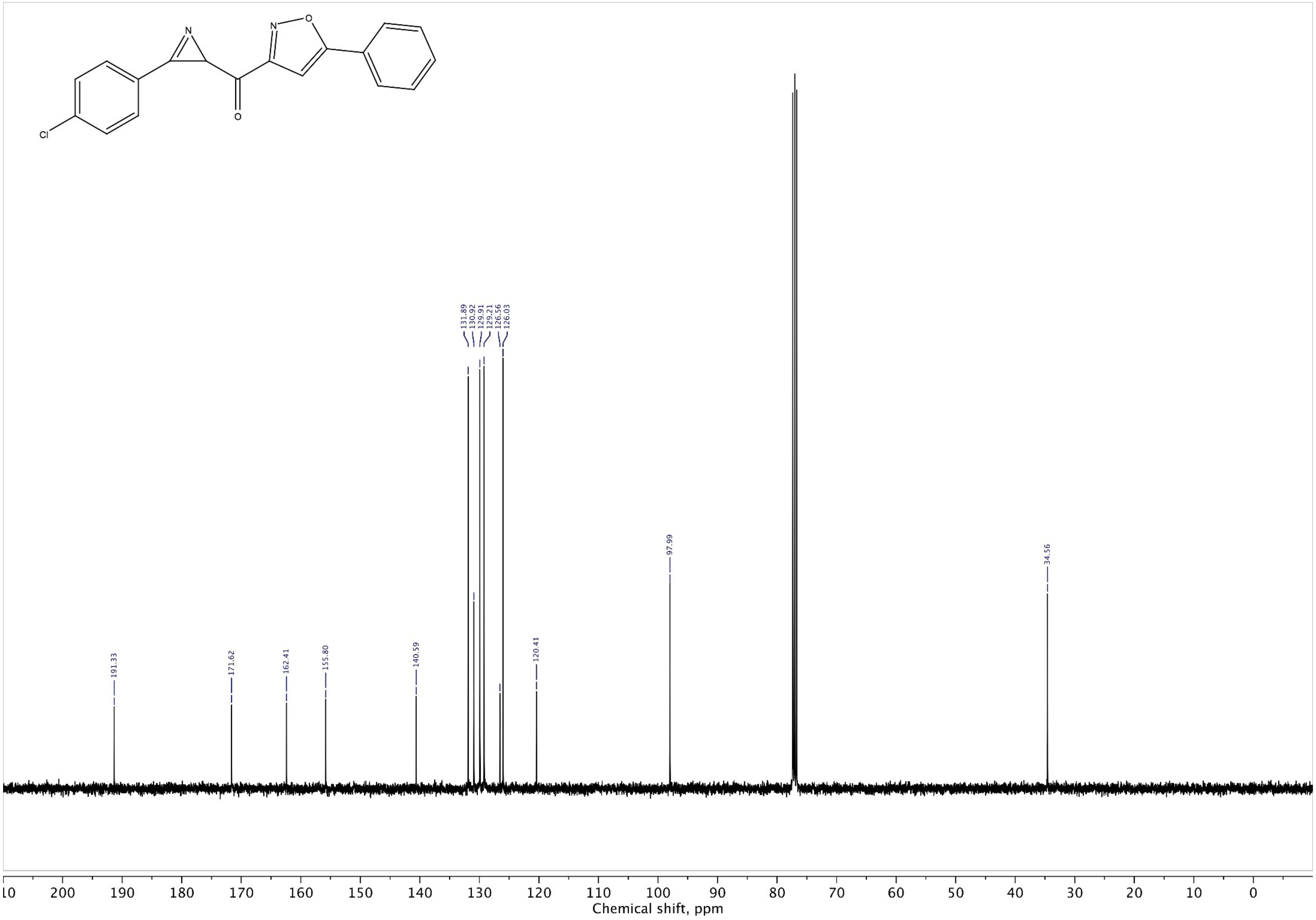

(3-(4-Chlorophenyl)-2*H*-azirin-2-yl)(5-phenylisoxazol-3-yl)methanone (3x), DEPT, $CDCl_3$, 100 MHz

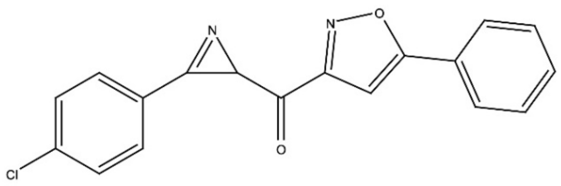

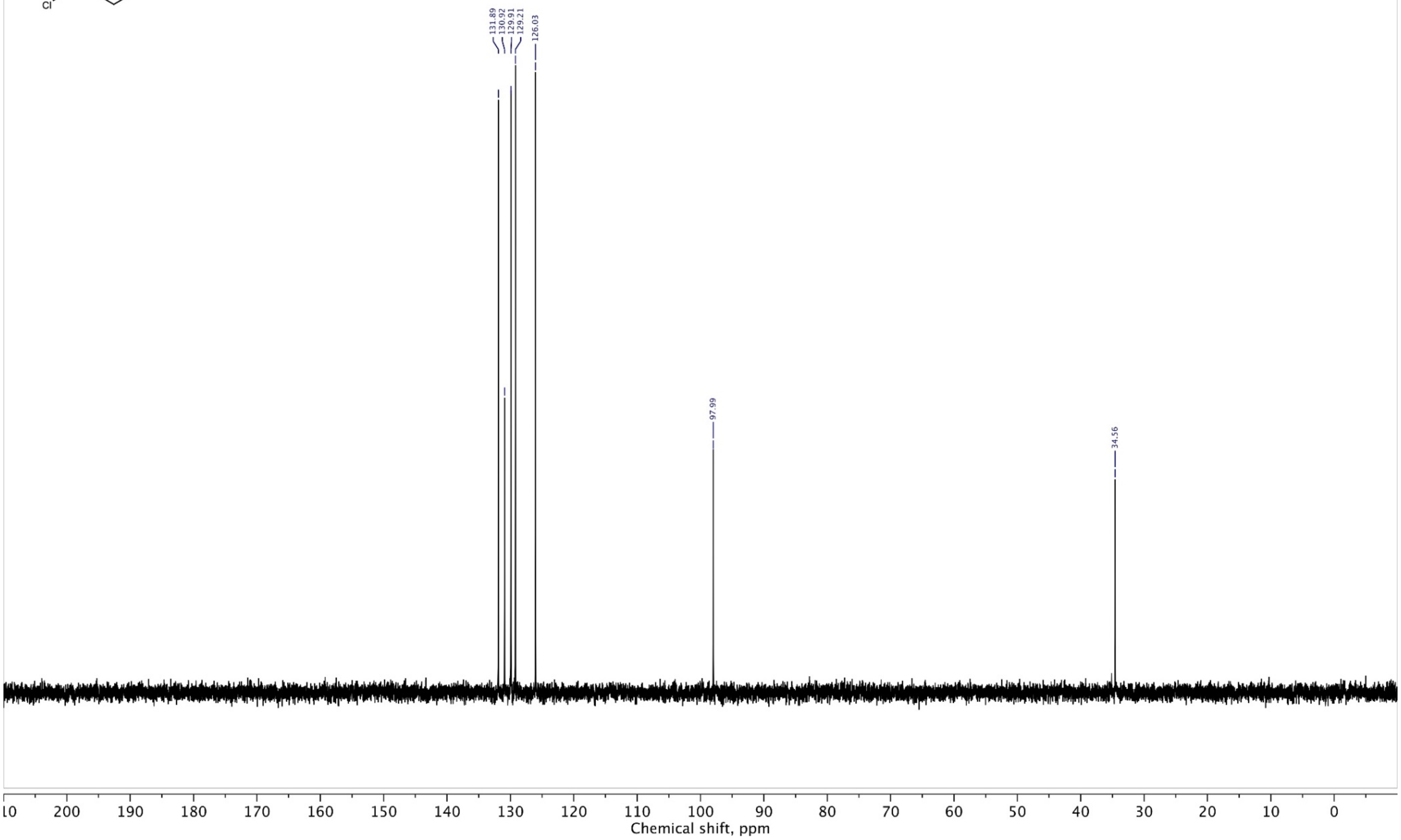

(3-Phenyl-2*H*-azirin-2-yl)(5-(thiophen-2-yl)isoxazol-3-yl)methanone (3y), ^{1}H NMR, $CDCl_3$, 400 MHz

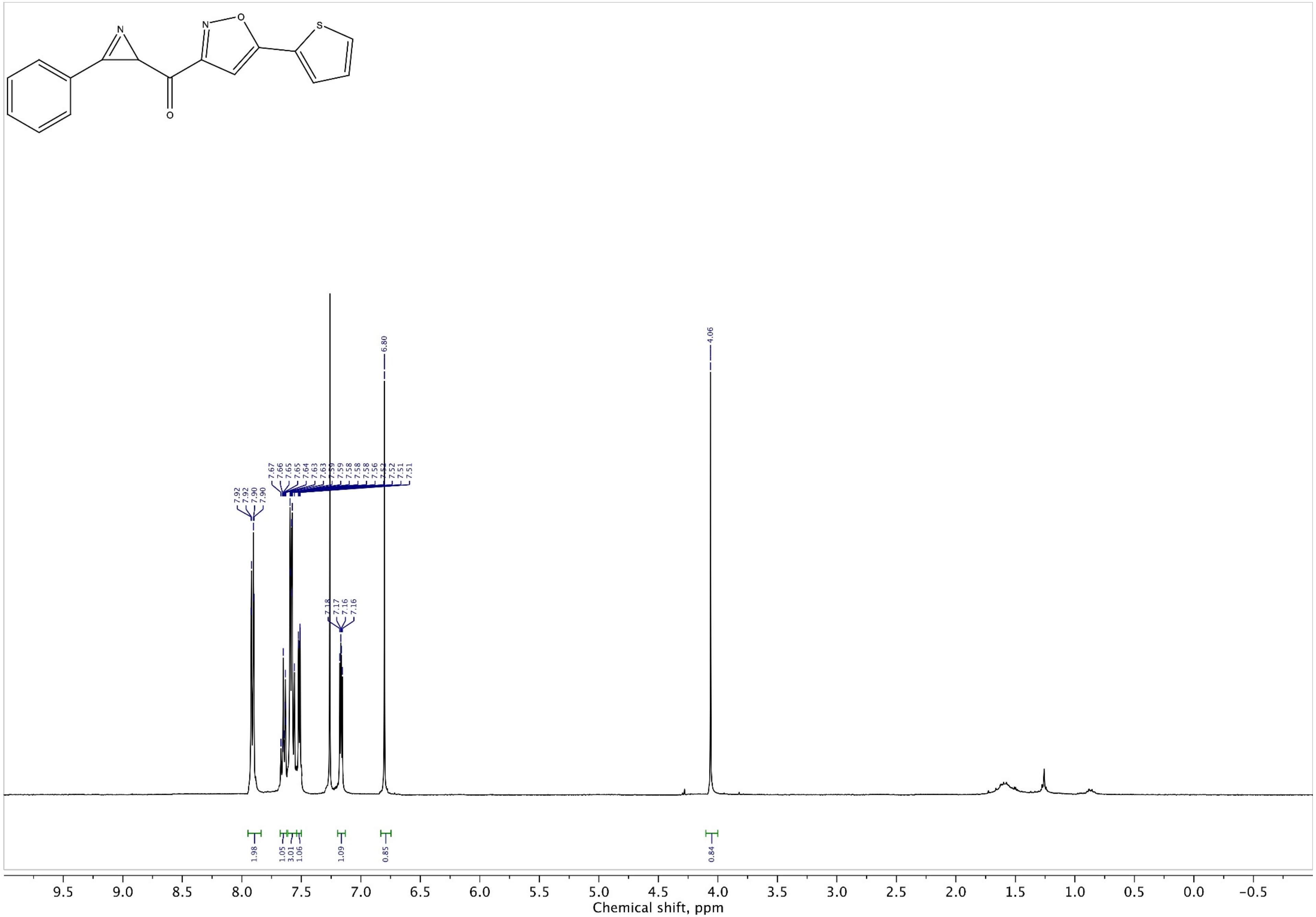

(3-Phenyl-2*H*-azirin-2-yl)(5-(thiophen-2-yl)isoxazol-3-yl)methanone (3y), $^{13}C\{^1H\}$ NMR, $CDCl_3$, 100 MHz

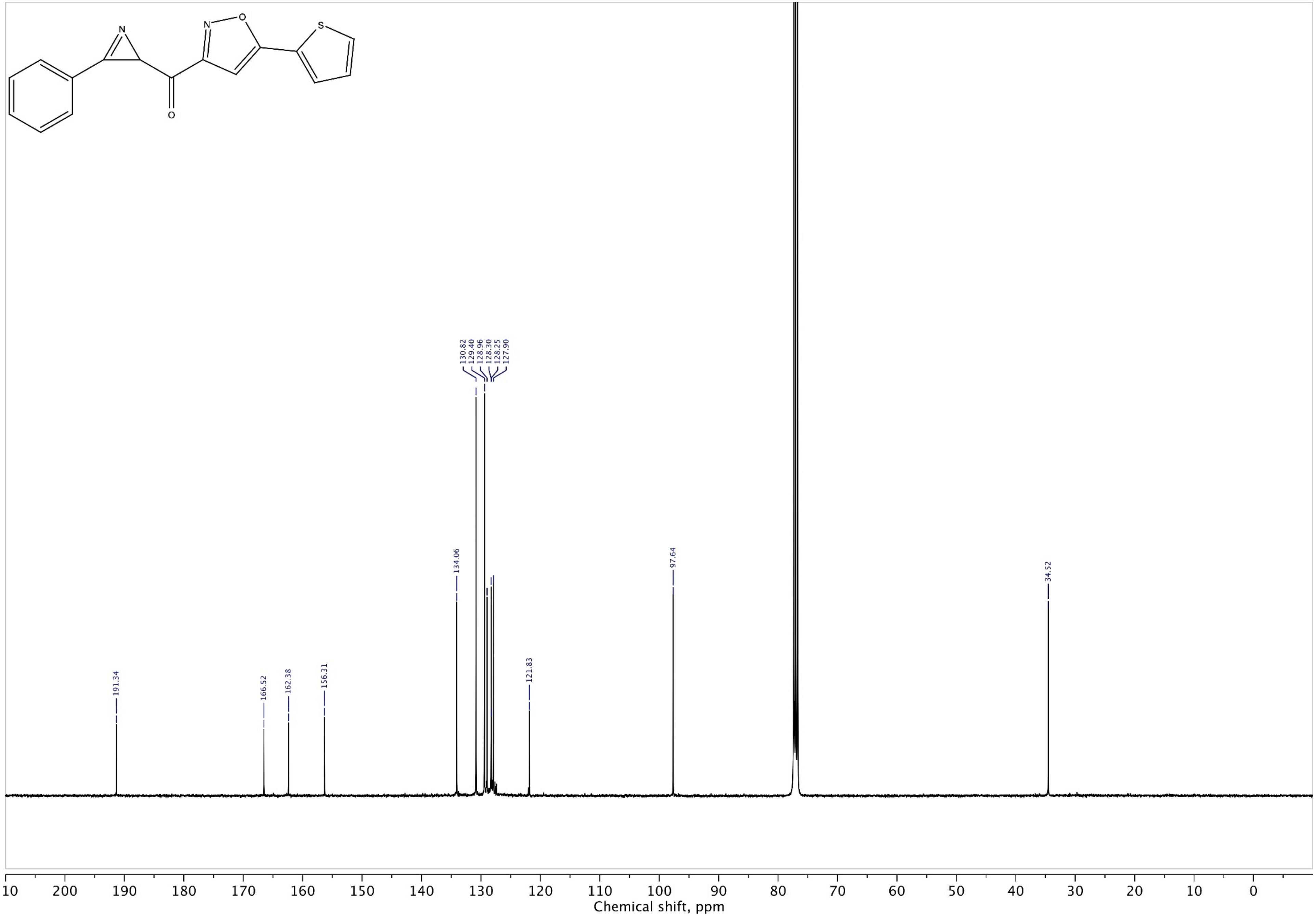

(3-Phenyl-2*H*-azirin-2-yl)(5-(thiophen-2-yl)isoxazol-3-yl)methanone (3y), DEPT, CDCl$_3$, 100 MHz

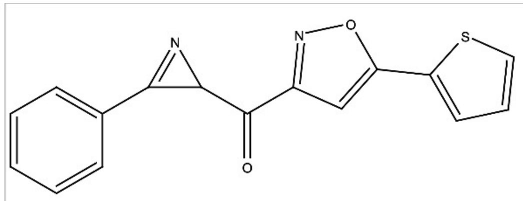

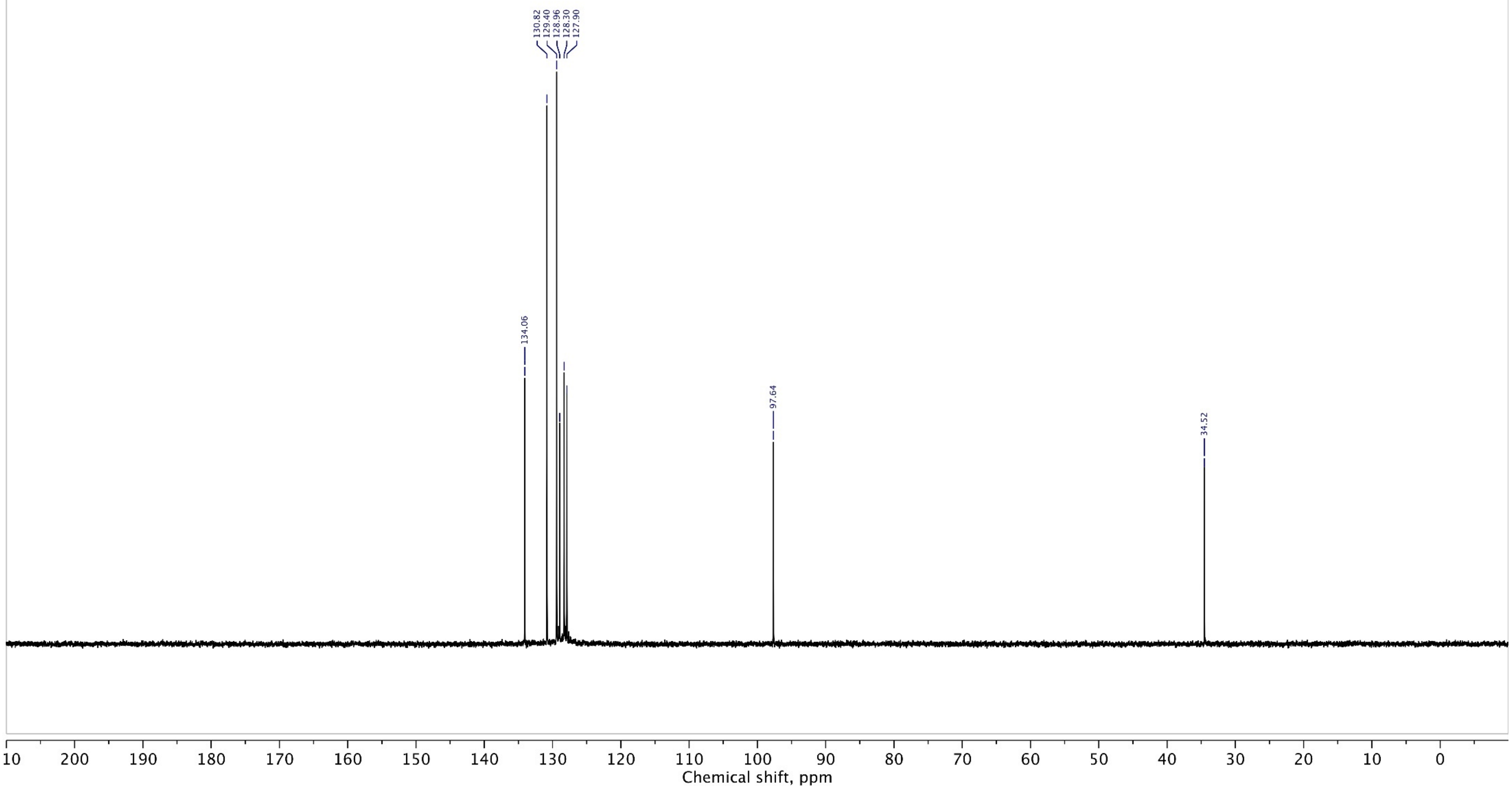

Methyl 3'-phenyl-[3,5'-biisoxazole]-5-carboxylate (4a), ^{1}H NMR, $CDCl_3$, 400 MHz

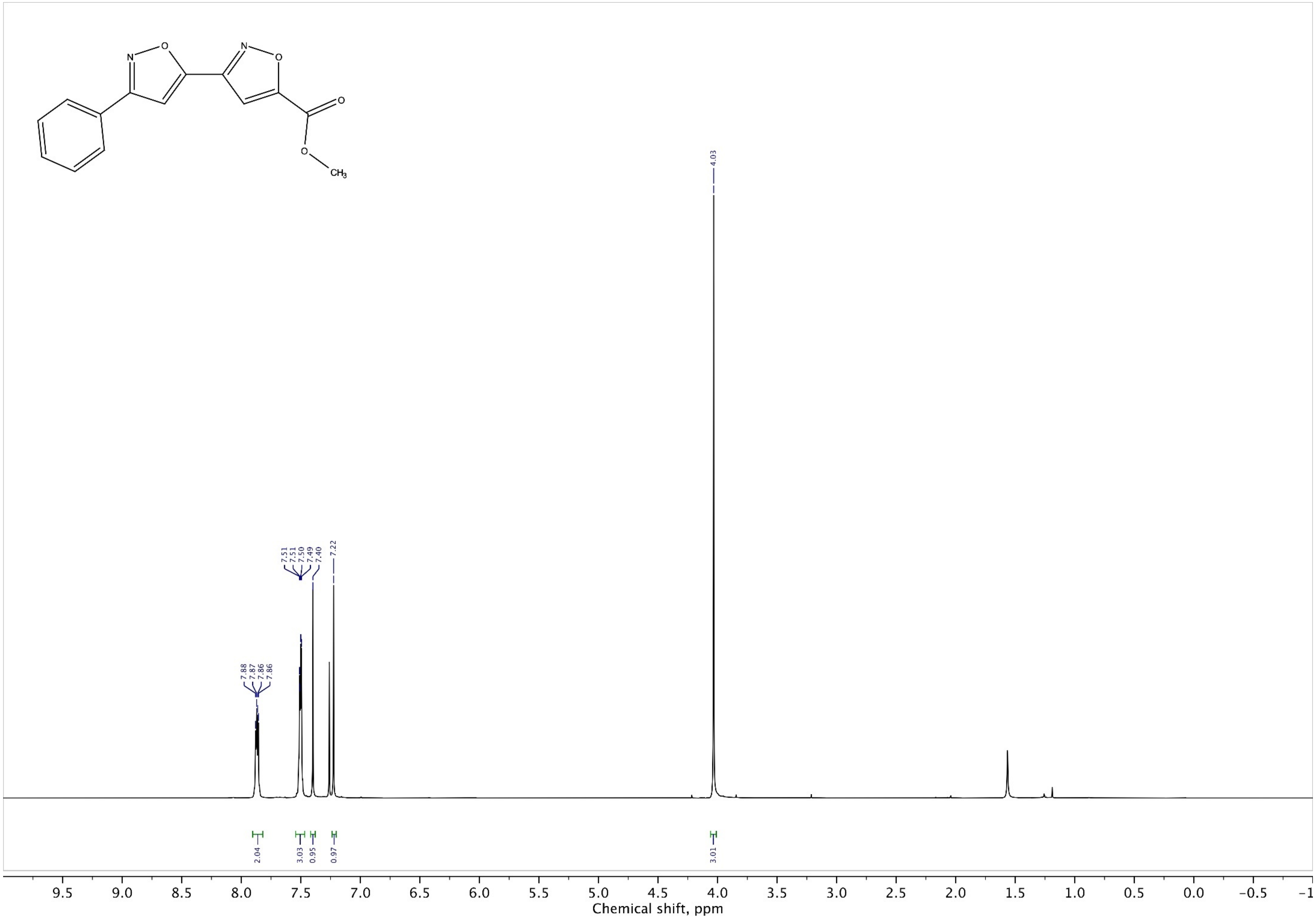

Methyl 3'-phenyl-[3,5'-biisoxazole]-5-carboxylate (4a), $^{13}C\{^1H\}$ NMR, $CDCl_3$, 100 MHz

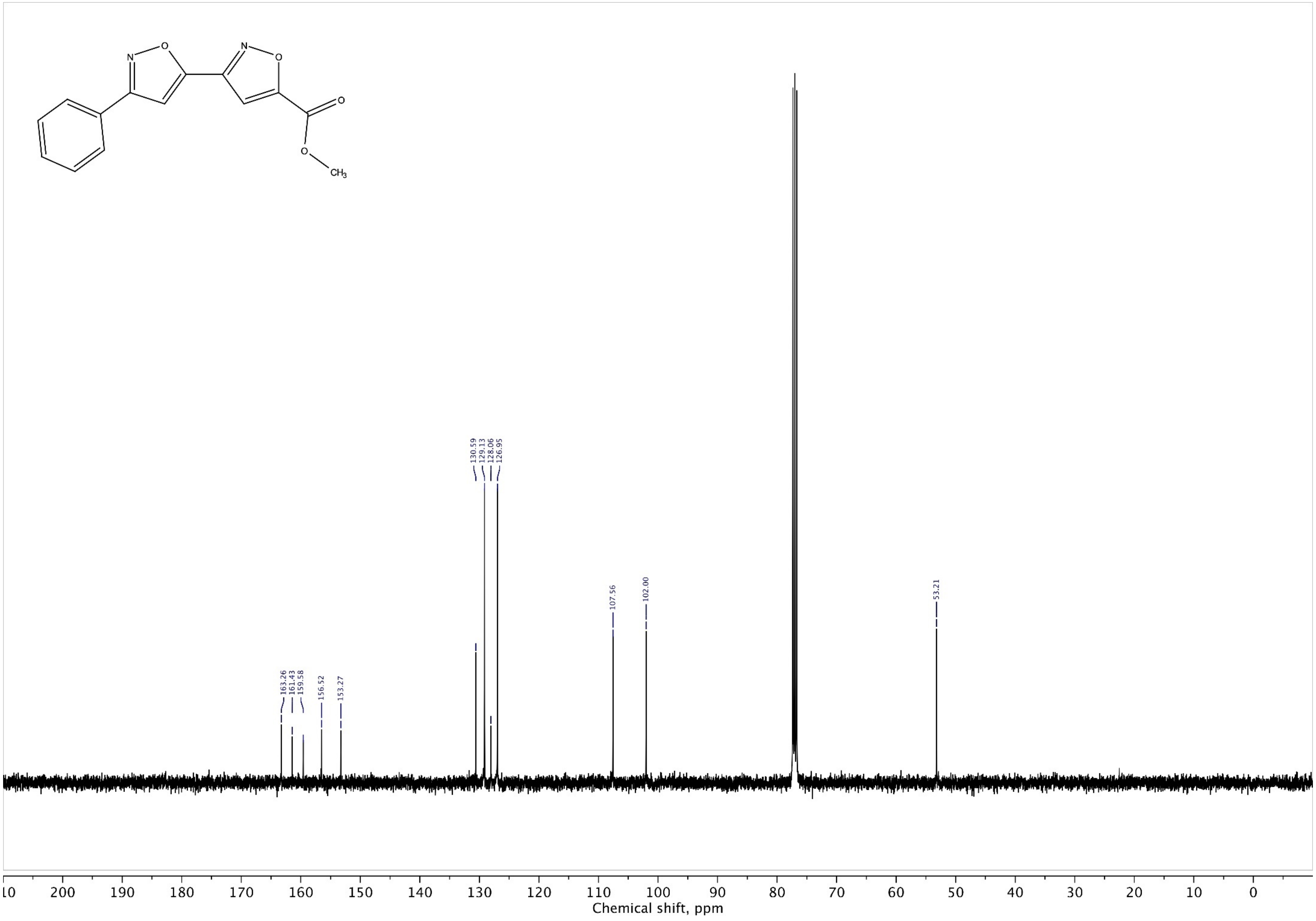

Methyl 3'-phenyl-[3,5'-biisoxazole]-5-carboxylate (4a), DEPT, $CDCl_3$, 100 MHz

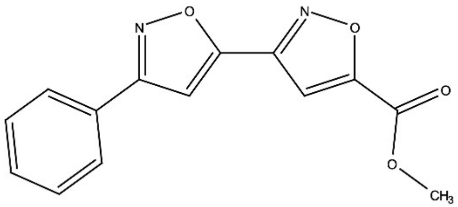

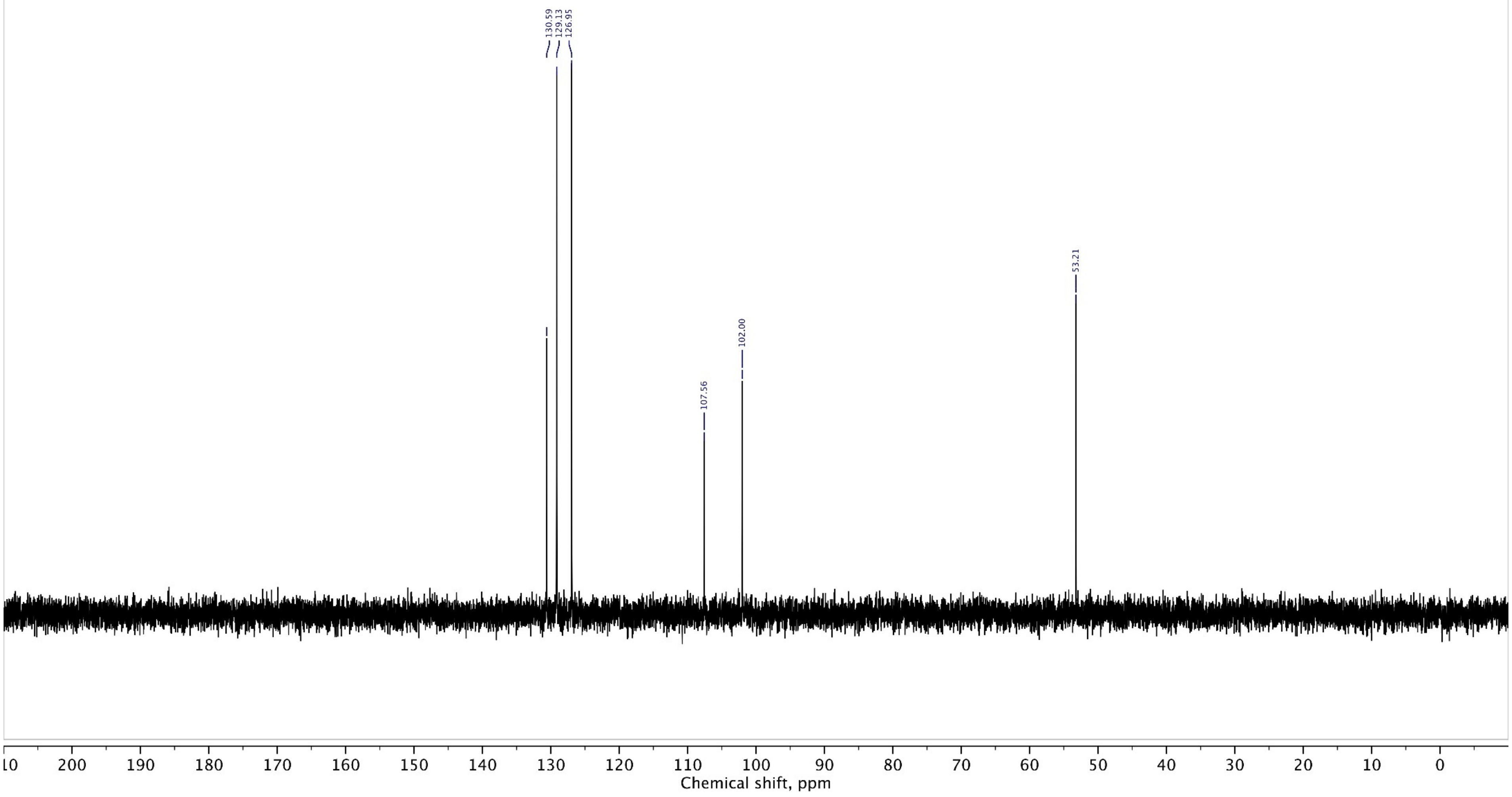

2-((3'-Phenyl-[3,5'-biisoxazol]-5-yl)methyl)isoindoline-1,3-dione (4b), ^{1}H NMR, DMSO-d_6, 400 MHz

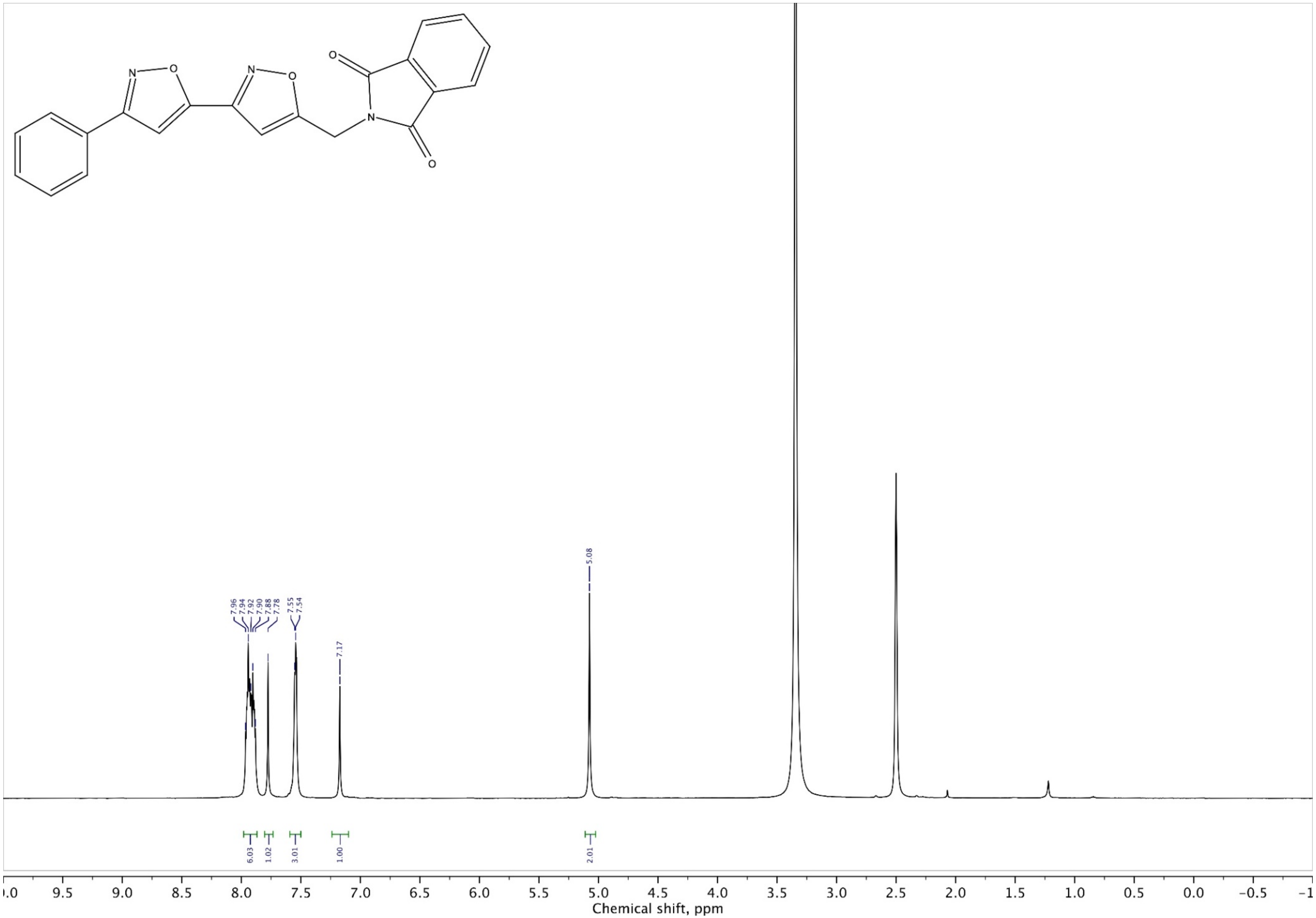

2-((3'-Phenyl-[3,5'-biisoxazol]-5-yl)methyl)isoindoline-1,3-dione (4b), $^{13}C\{^1H\}$ NMR, DMSO-d_6, 100 MHz

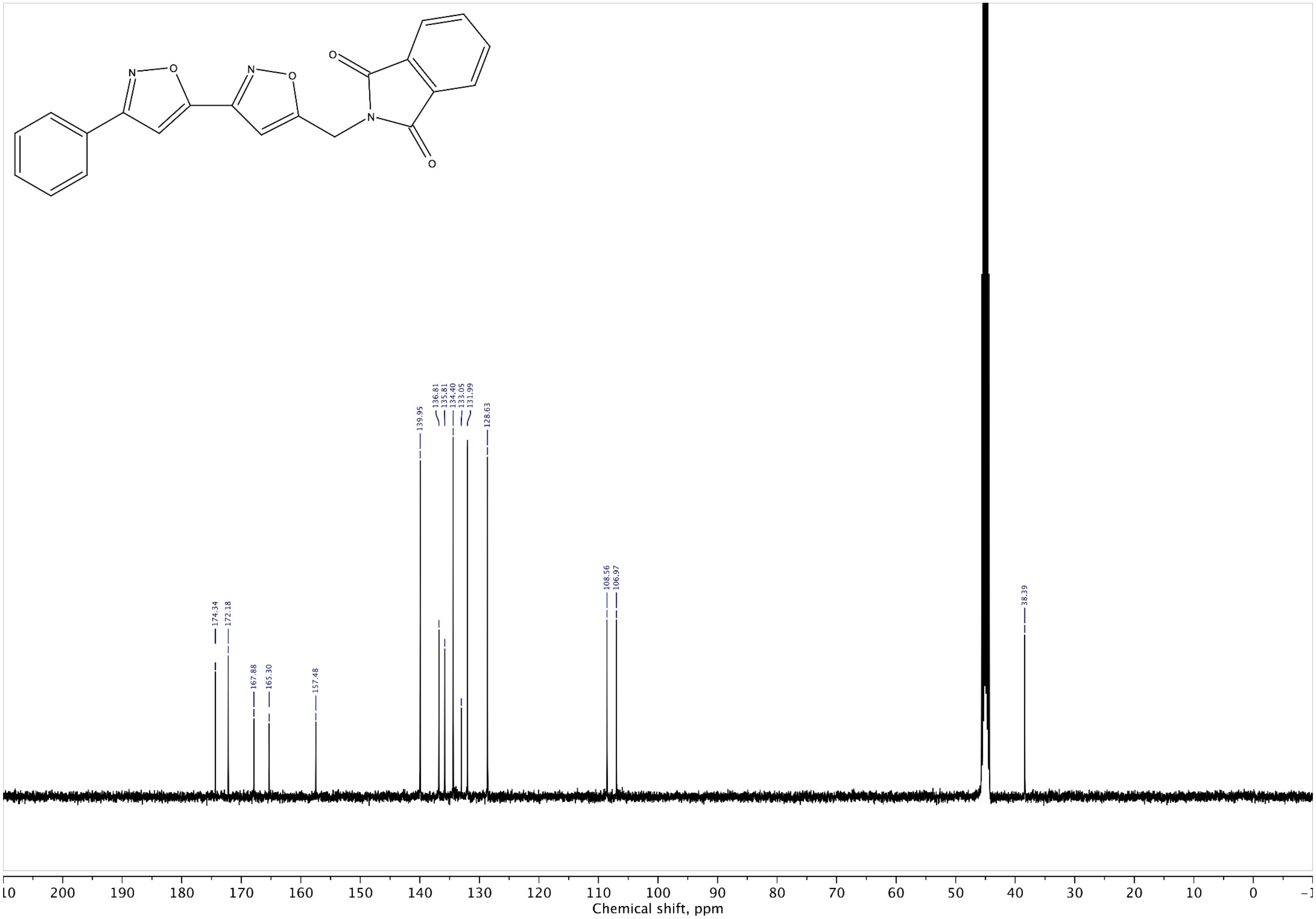

2-((3'-Phenyl-[3,5'-biisoxazol]-5-yl)methyl)isoindoline-1,3-dione (4b), DEPT, DMSO-d_6, 100 MHz

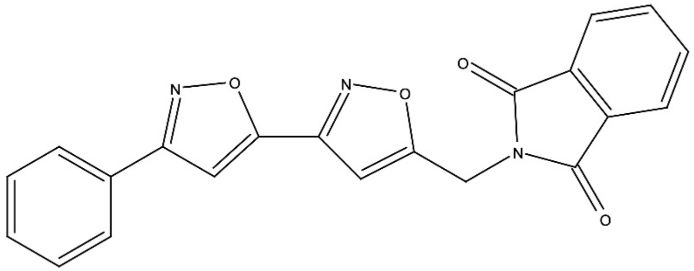

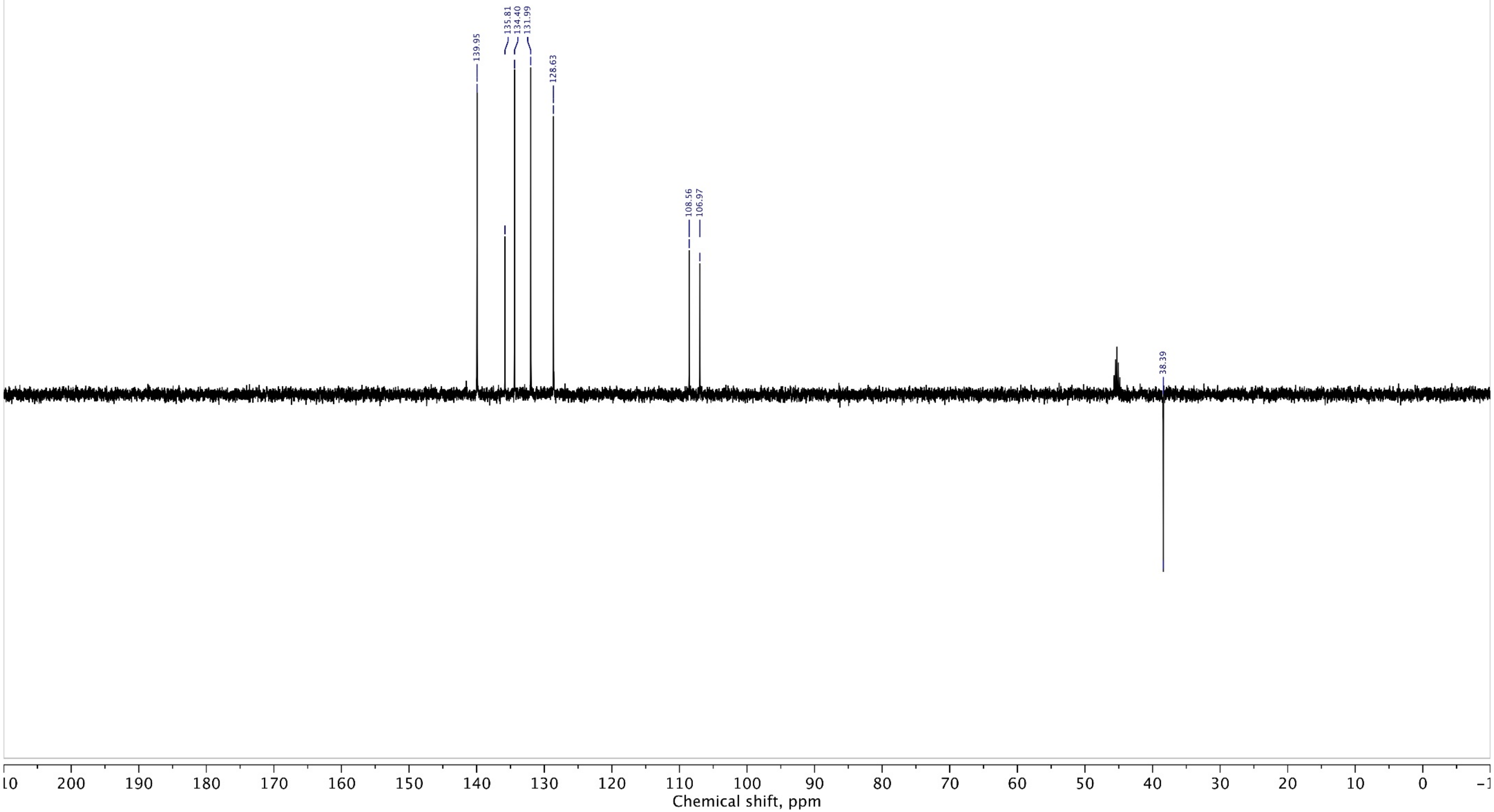

(3'-Phenyl-[3,5'-biisoxazol]-5-yl)methanol (4c), ^{1}H NMR, DMSO-d_6, 400 MHz

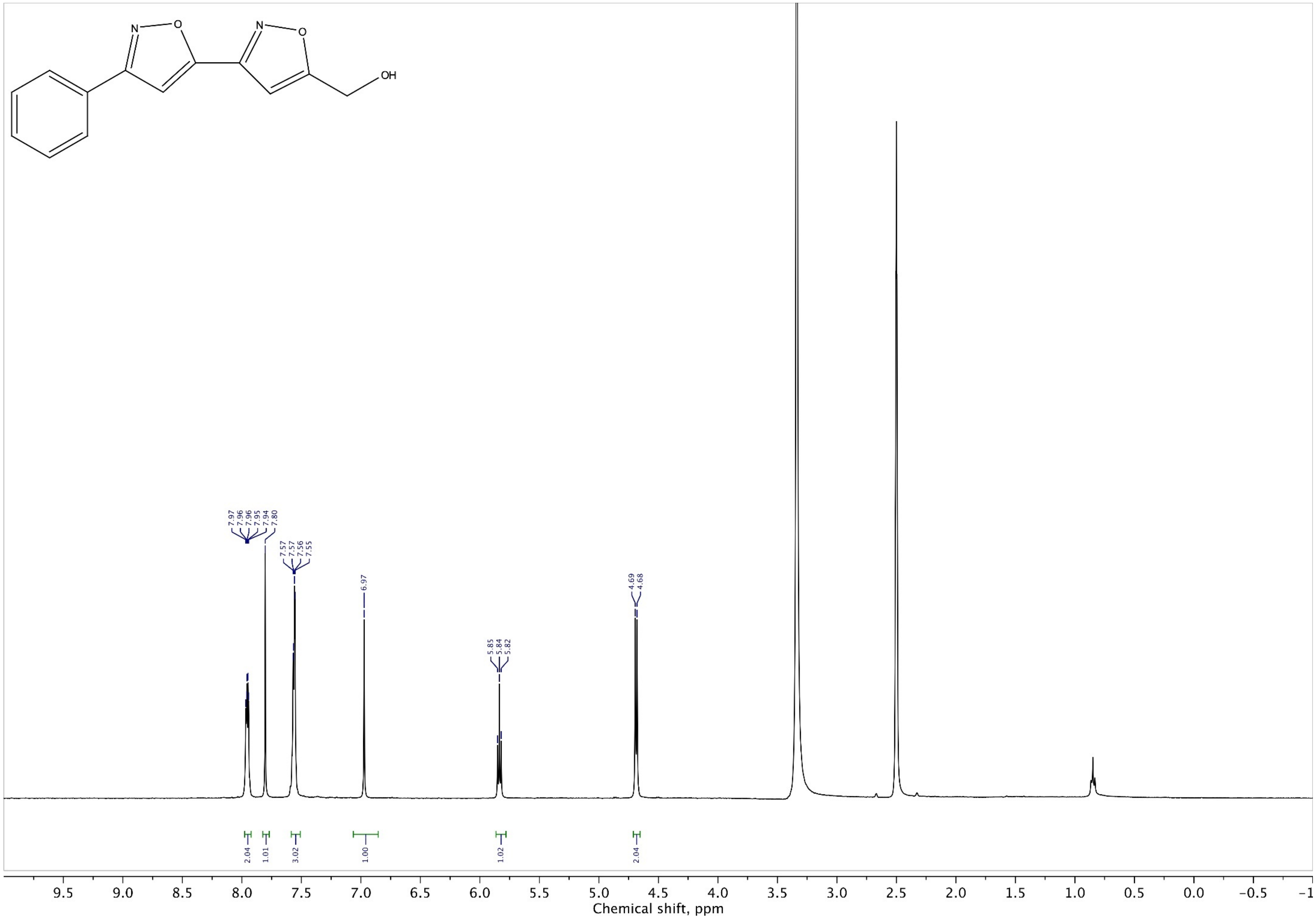

(3'-Phenyl-[3,5'-biisoxazol]-5-yl)methanol (4c), $^{13}C\{^1H\}$ NMR, DMSO-d_6, 100 MHz

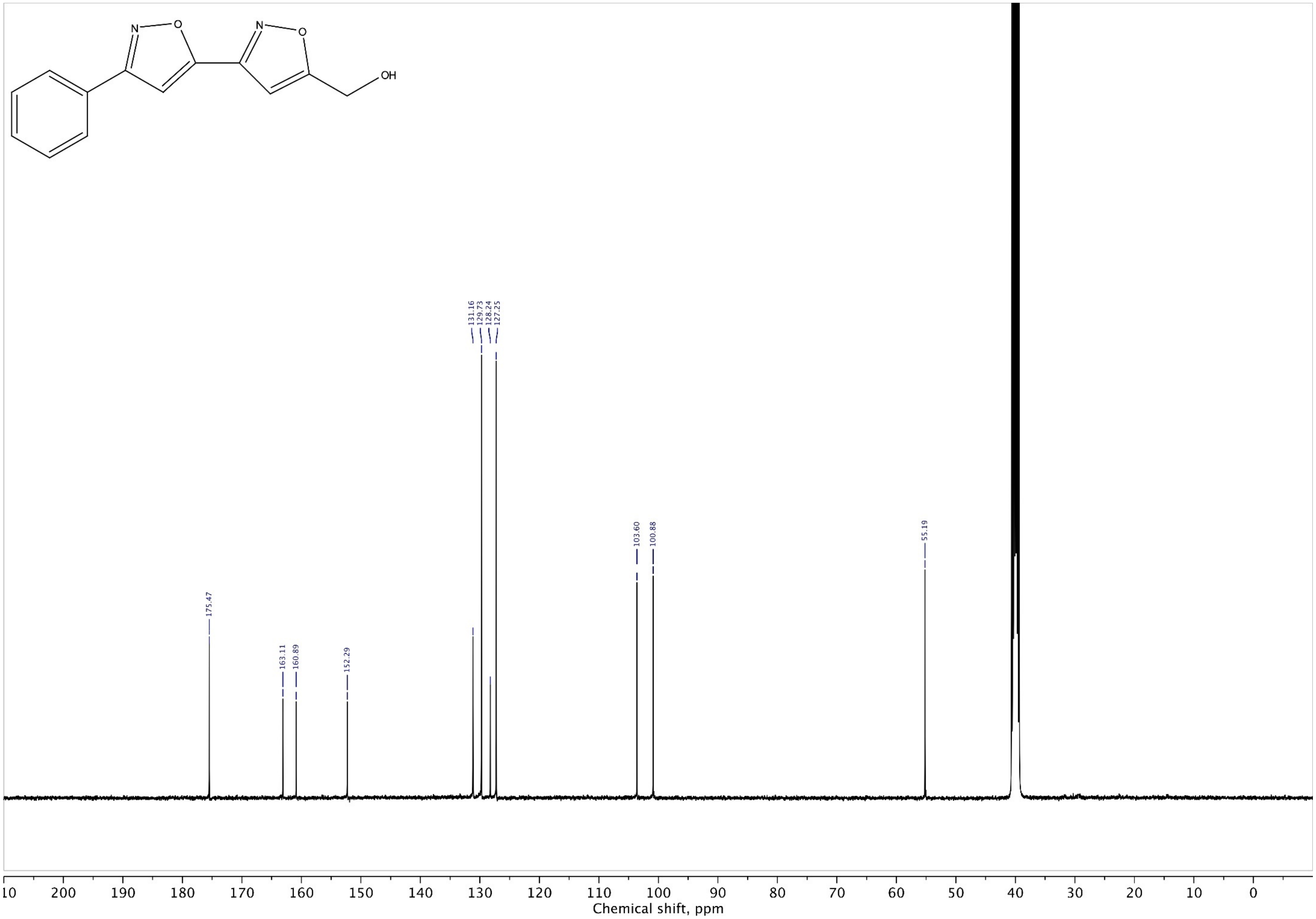

(3'-Phenyl-[3,5'-biisoxazol]-5-yl)methanol (4c), DEPT, DMSO-d_6, 100 MHz

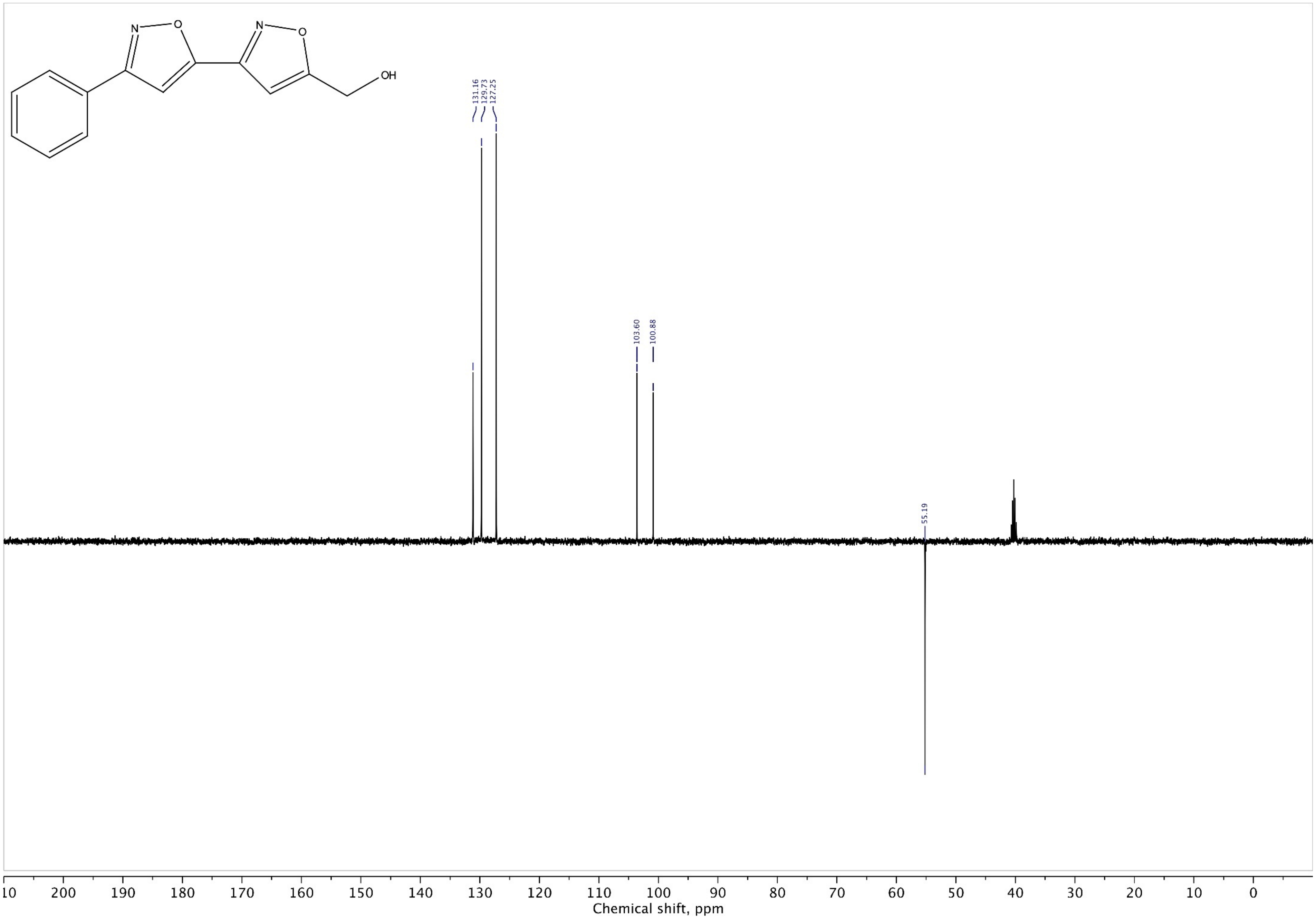

5-(Methoxymethyl)-3'-phenyl-3,5'-biisoxazole (4d), ^{1}H NMR, $CDCl_3$, 400 MHz

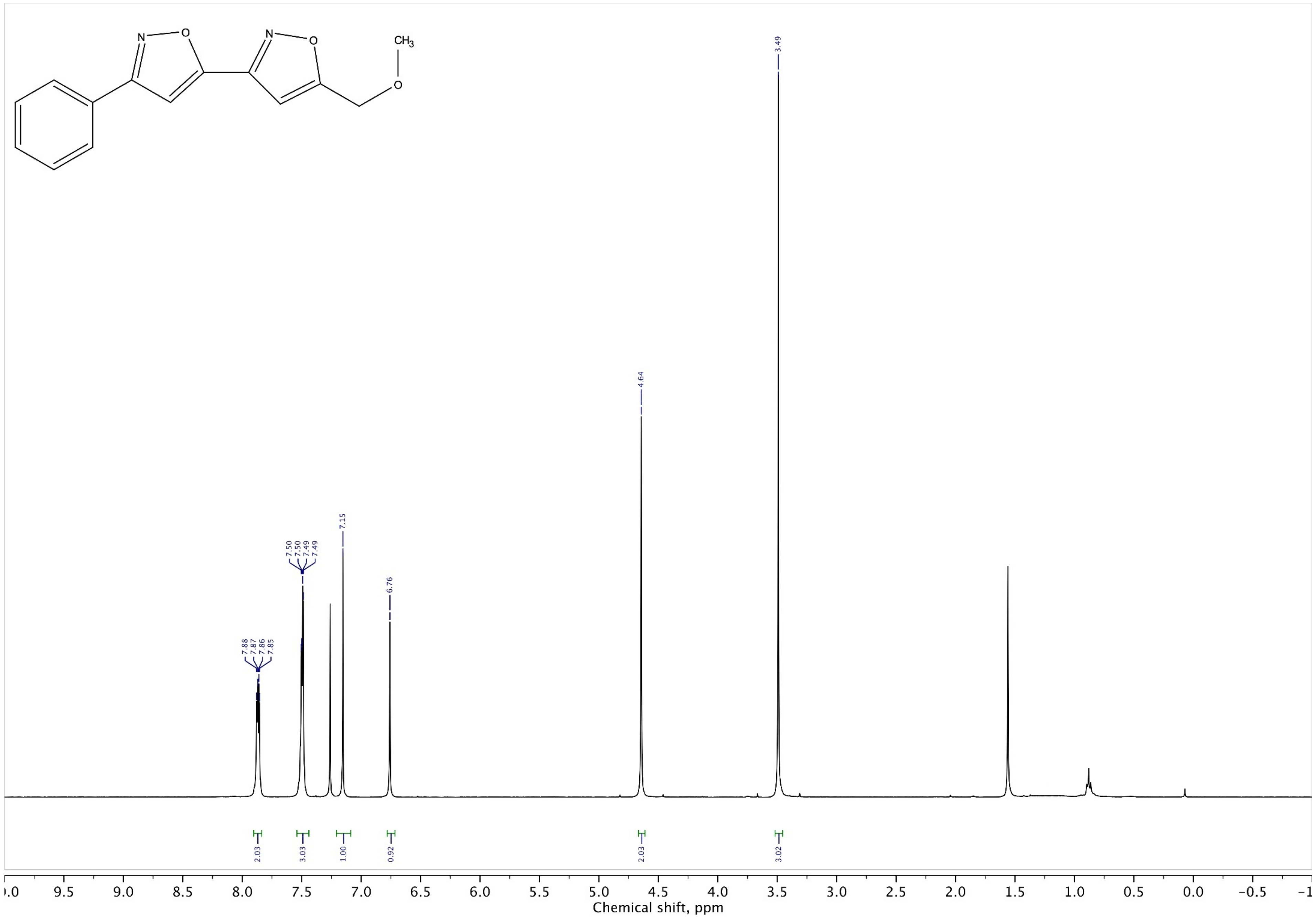

5-(Methoxymethyl)-3'-phenyl-3,5'-biisoxazole (4d), $^{13}C\{^{1}H\}$ NMR, $CDCl_3$, 100 MHz

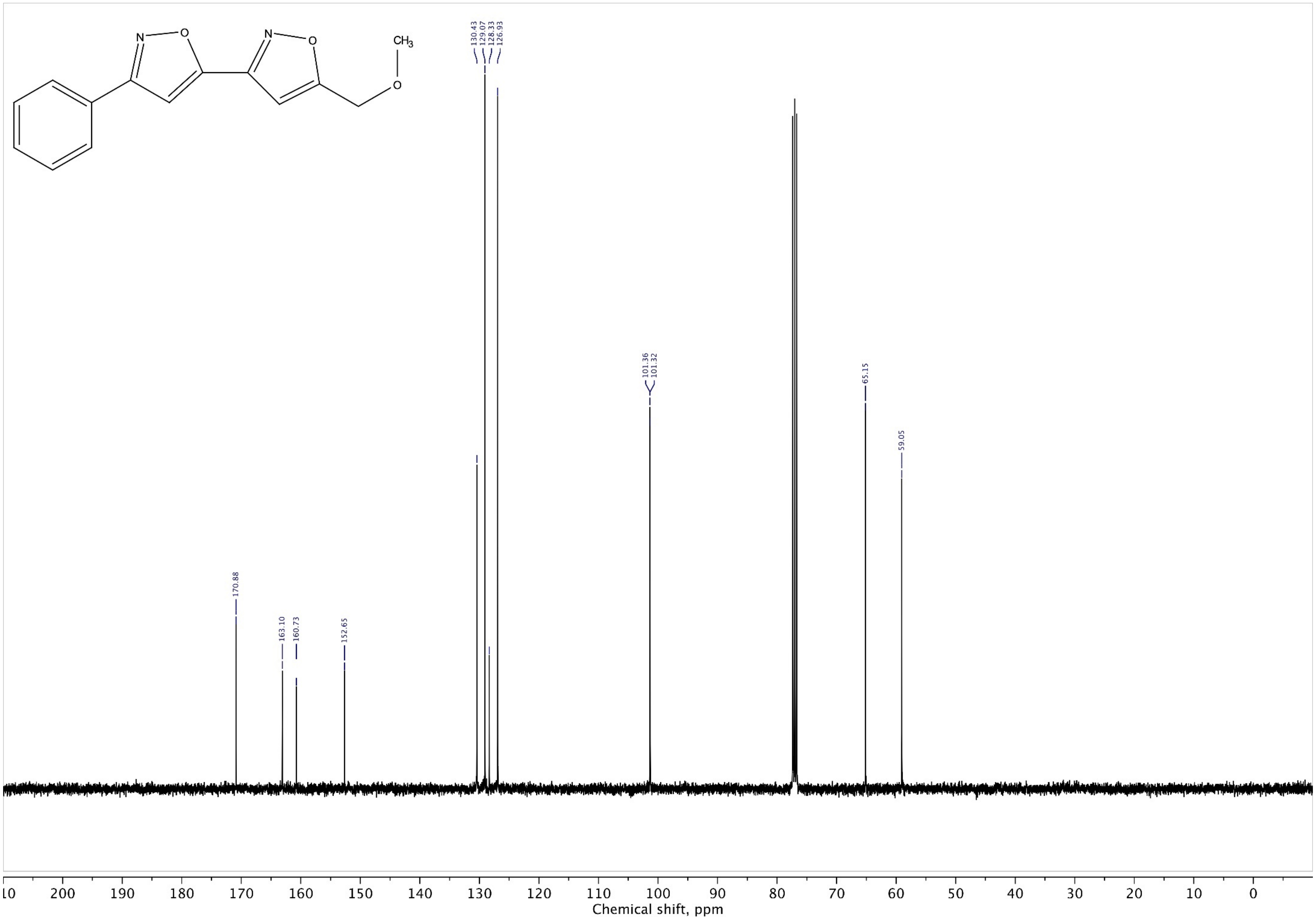

5-(Methoxymethyl)-3'-phenyl-3,5'-biisoxazole (4d), DEPT, $CDCl_3$, 100 MHz

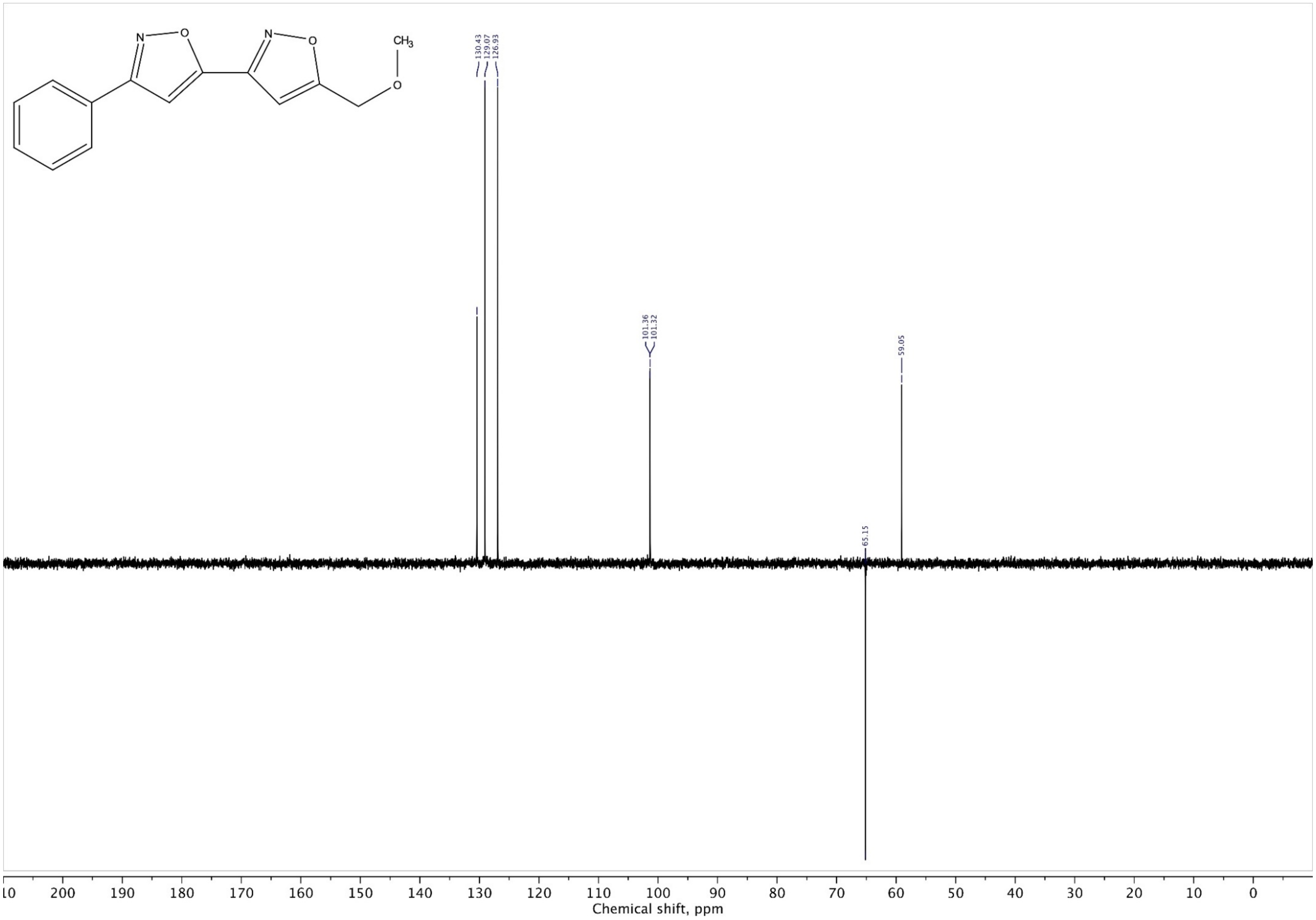

5-(Phenoxymethyl)-3'-phenyl-3,5'-biisoxazole (4e), ^{1}H NMR, $CDCl_3$, 400 MHz

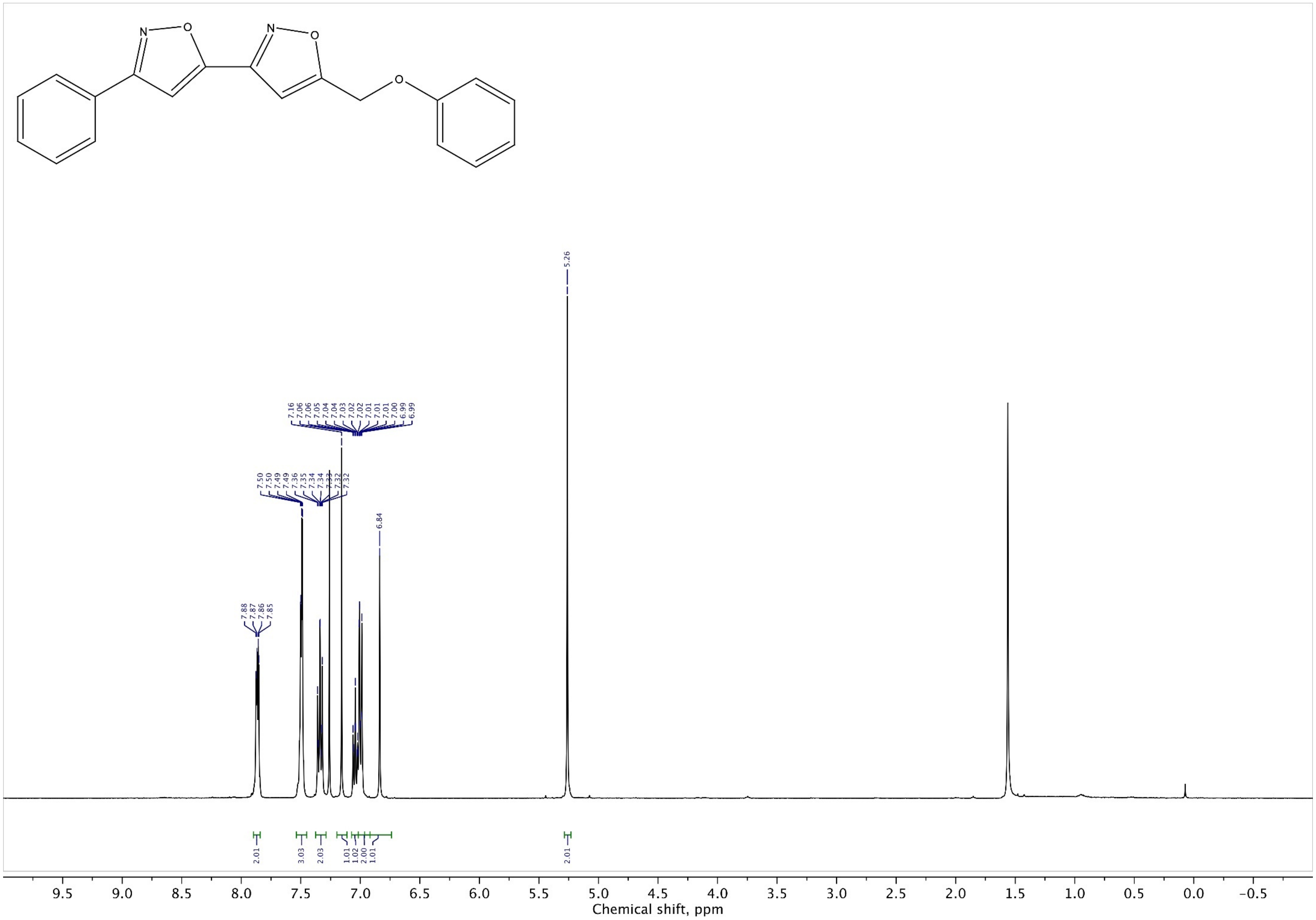

5-(Phenoxymethyl)-3'-phenyl-3,5'-biisoxazole (4e), $^{13}C\{^1H\}$ NMR, $CDCl_3$, 100 MHz

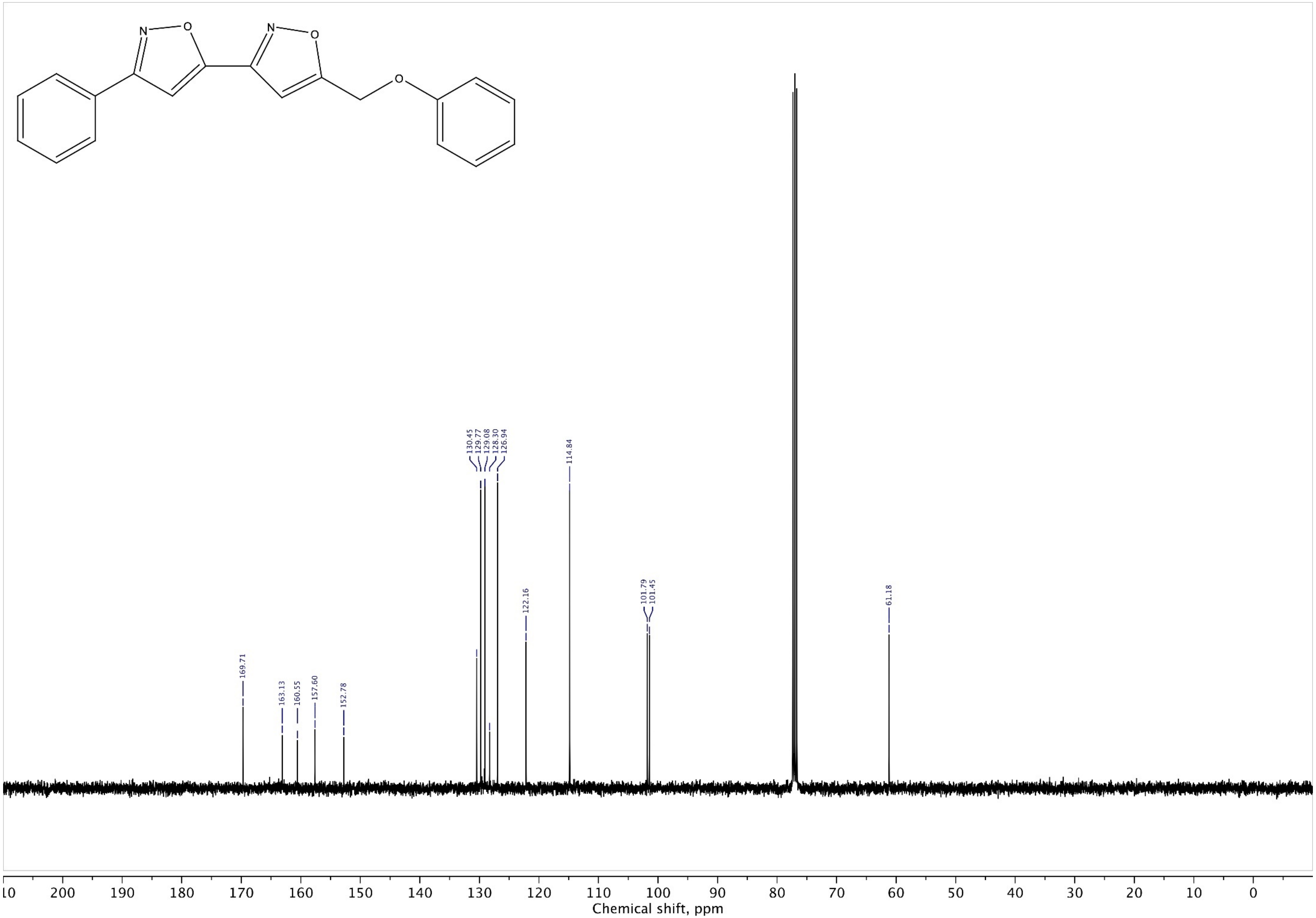

5-(Phenoxymethyl)-3'-phenyl-3,5'-biisoxazole (4e), DEPT, $CDCl_3$, 100 MHz

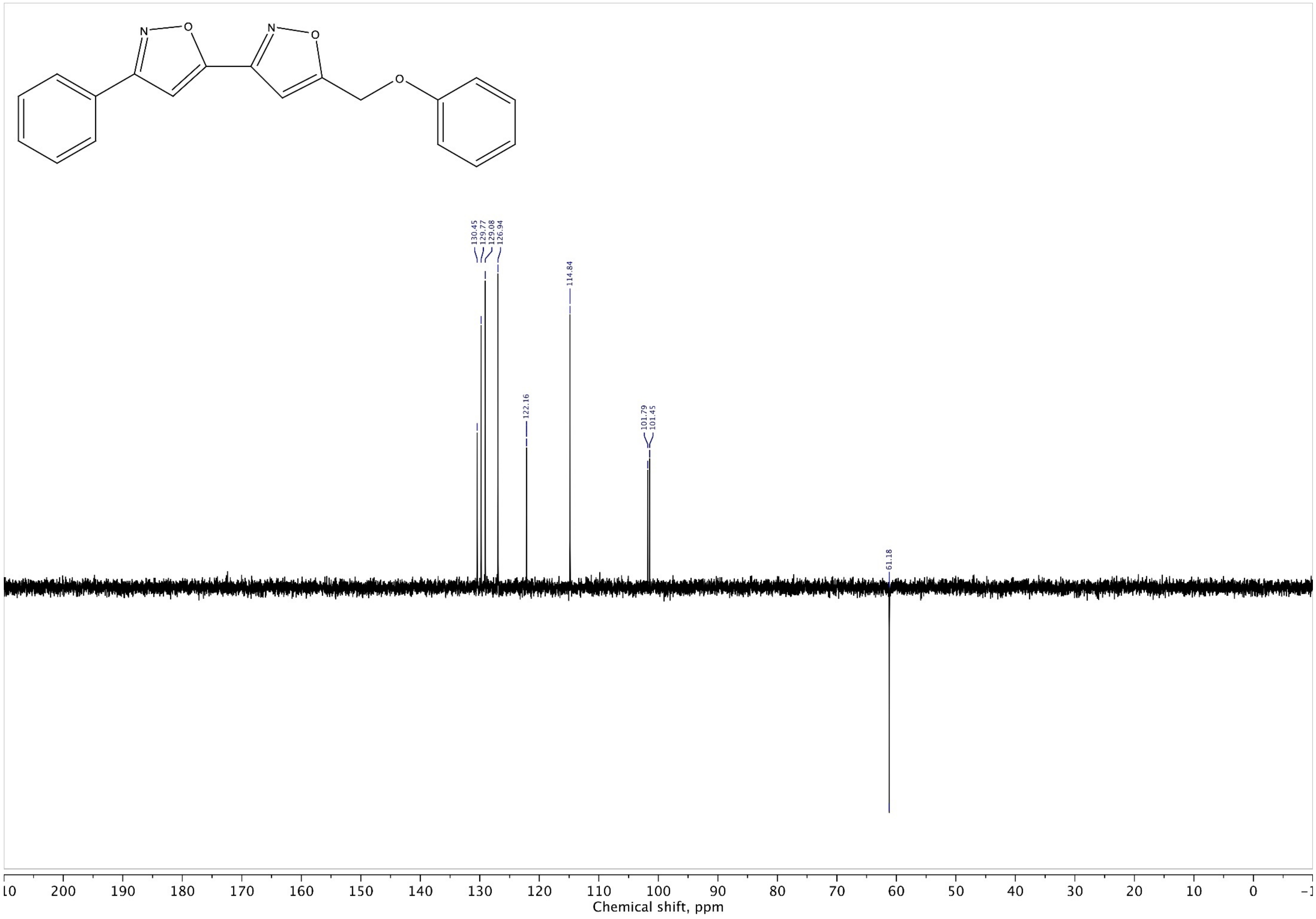

(3'-Phenyl-[3,5'-biisoxazol]-5-yl)methyl benzenesulfonate (4f), ^{1}H NMR, $CDCl_3$, 400 MHz

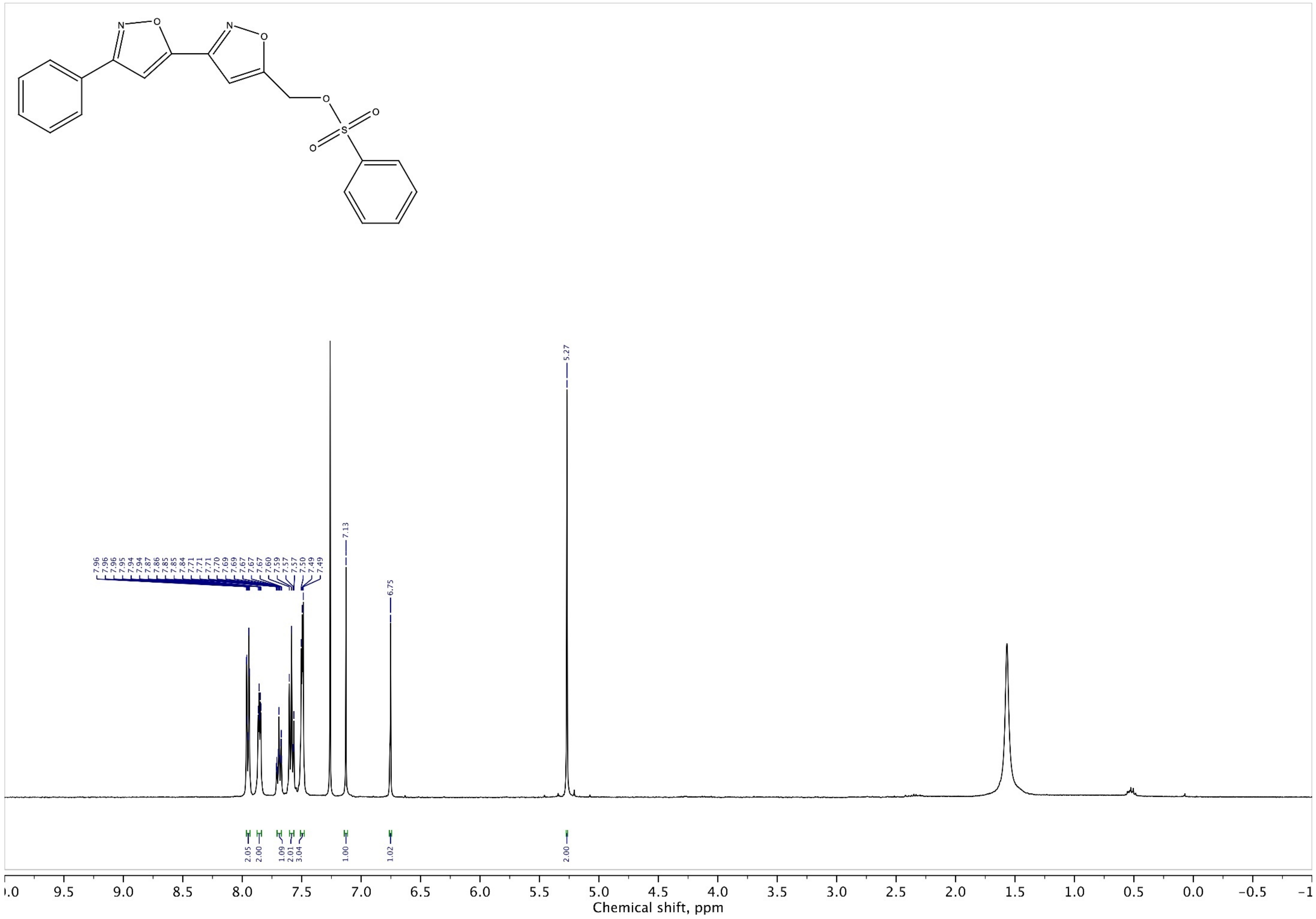

(3'-Phenyl-[3,5'-biisoxazol]-5-yl)methyl benzenesulfonate (4f), $^{13}C\{^{1}H\}$ NMR, $CDCl_3$, 100 MHz

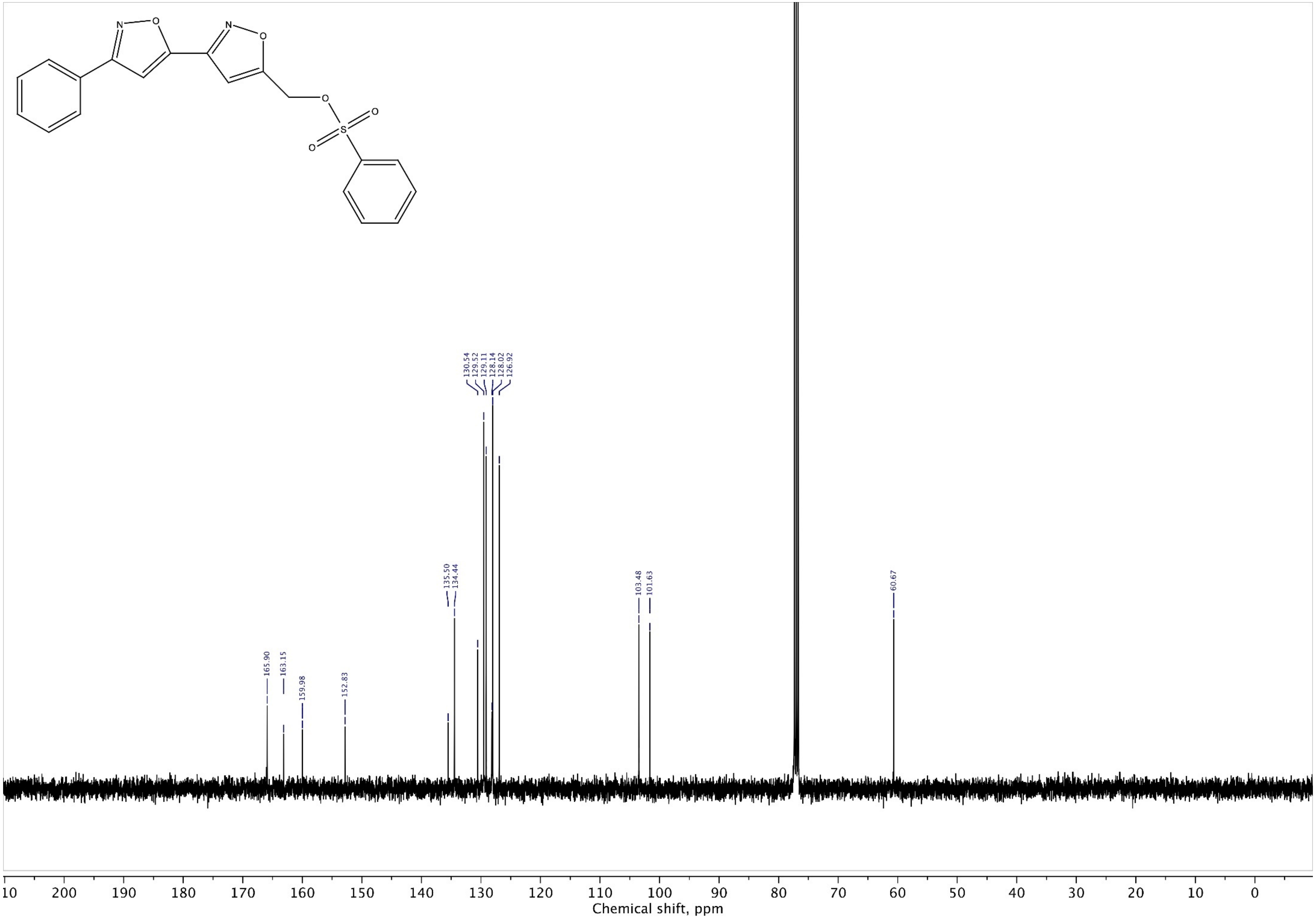

(3'-Phenyl-[3,5'-biisoxazol]-5-yl)methyl benzenesulfonate (4f), DEPT, CDCl$_3$, 100 MHz

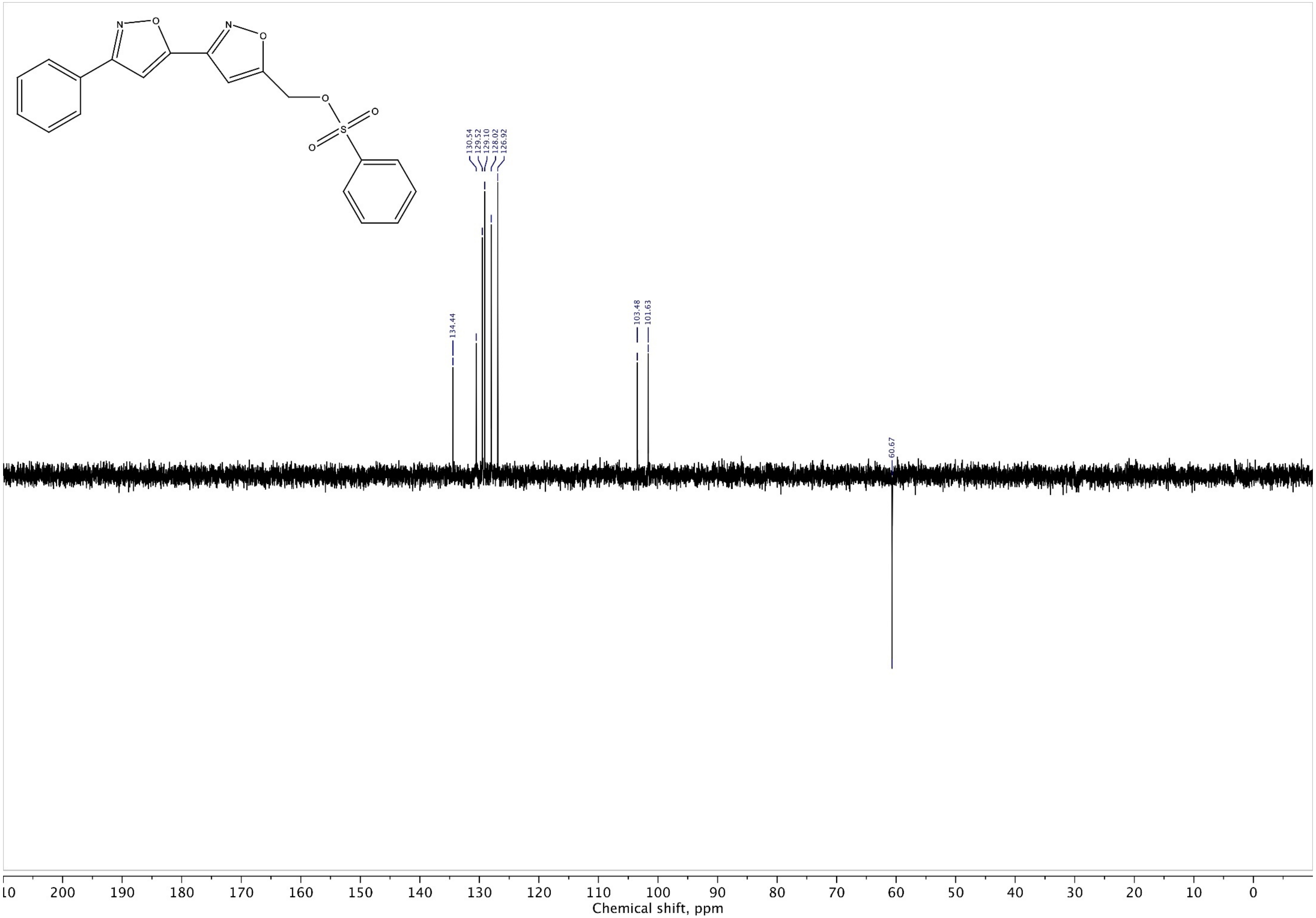

5-(Chloromethyl)-3'-phenyl-3,5'-biisoxazole (4g), ^{1}H NMR, $CDCl_3$, 400 MHz

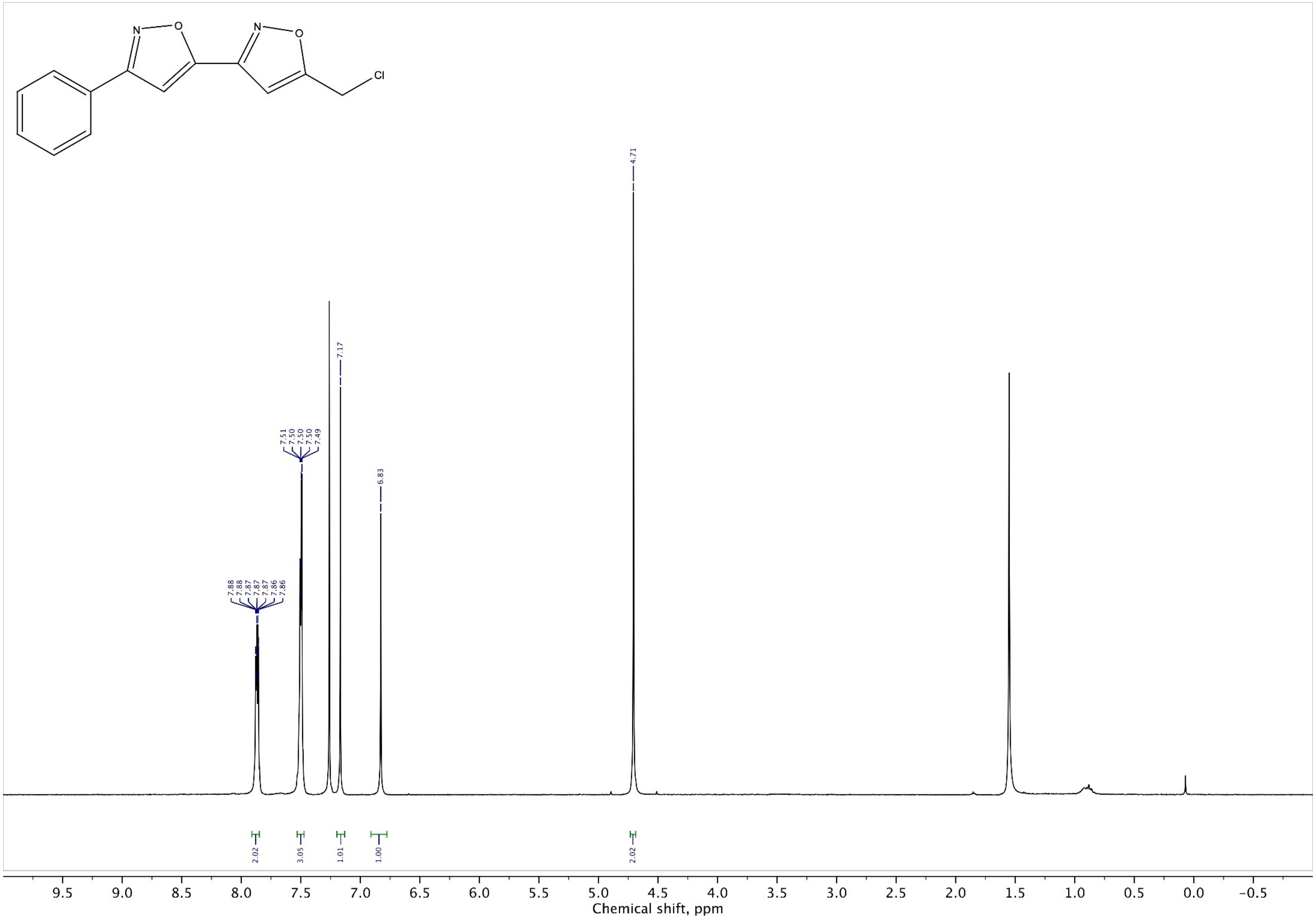

5-(Chloromethyl)-3'-phenyl-3,5'-biisoxazole (4g), $^{13}C\{^1H\}$ NMR, $CDCl_3$, 100 MHz

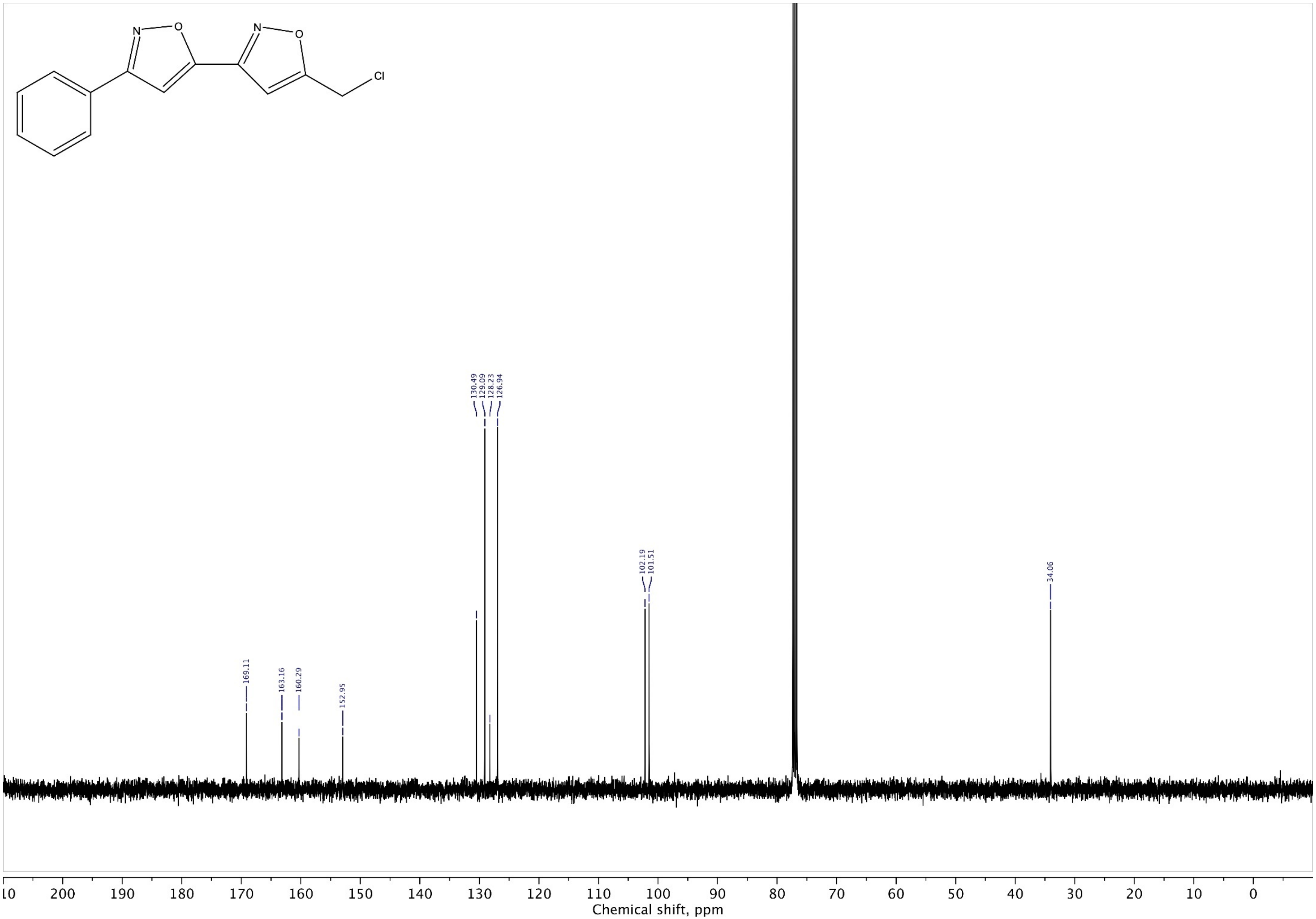

5-(Chloromethyl)-3'-phenyl-3,5'-biisoxazole (4g), DEPT, $CDCl_3$, 100 MHz

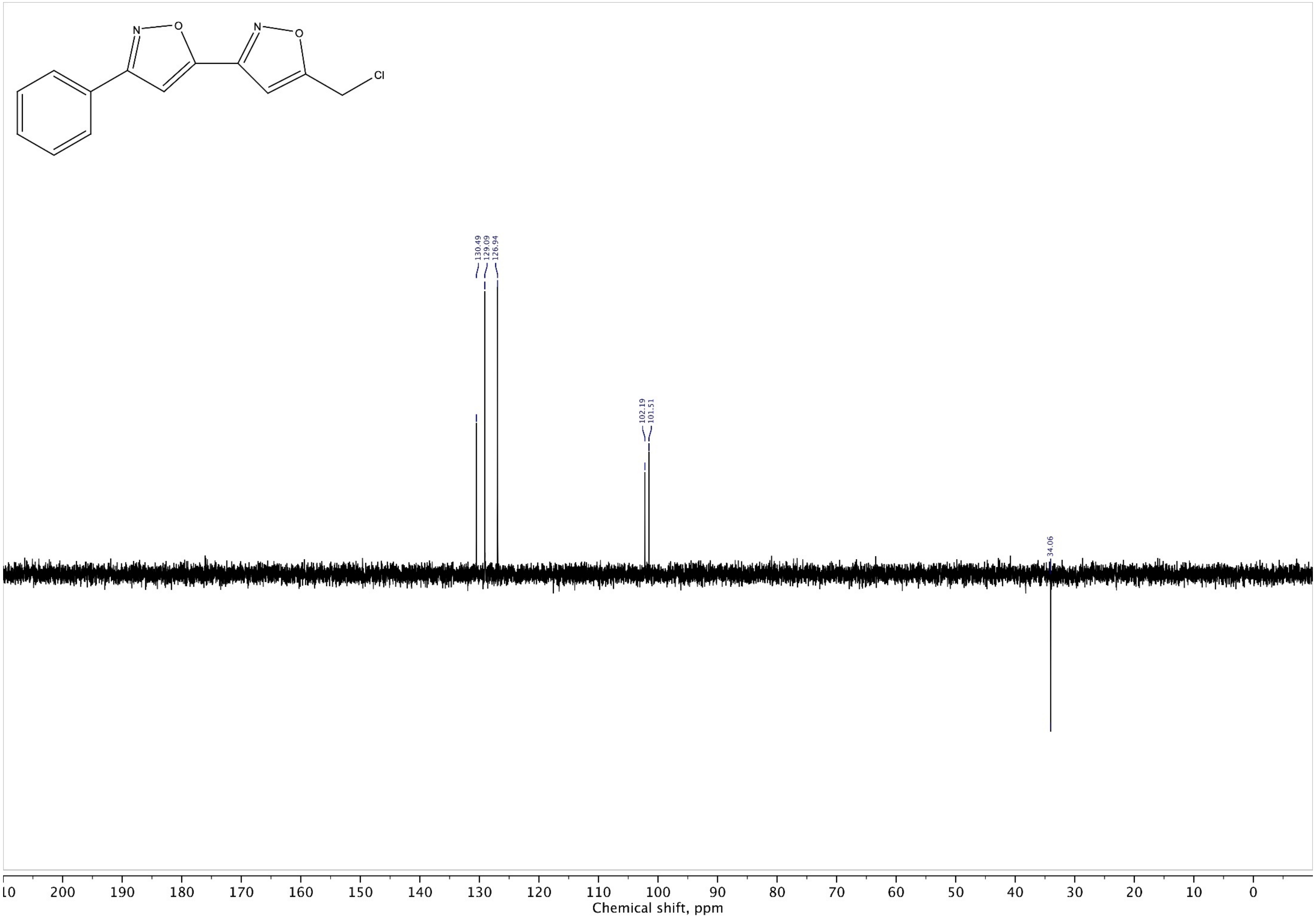

5-(Bromomethyl)-3'-phenyl-3,5'-biisoxazole (4h), ^{1}H NMR, $CDCl_3$, 400 MHz

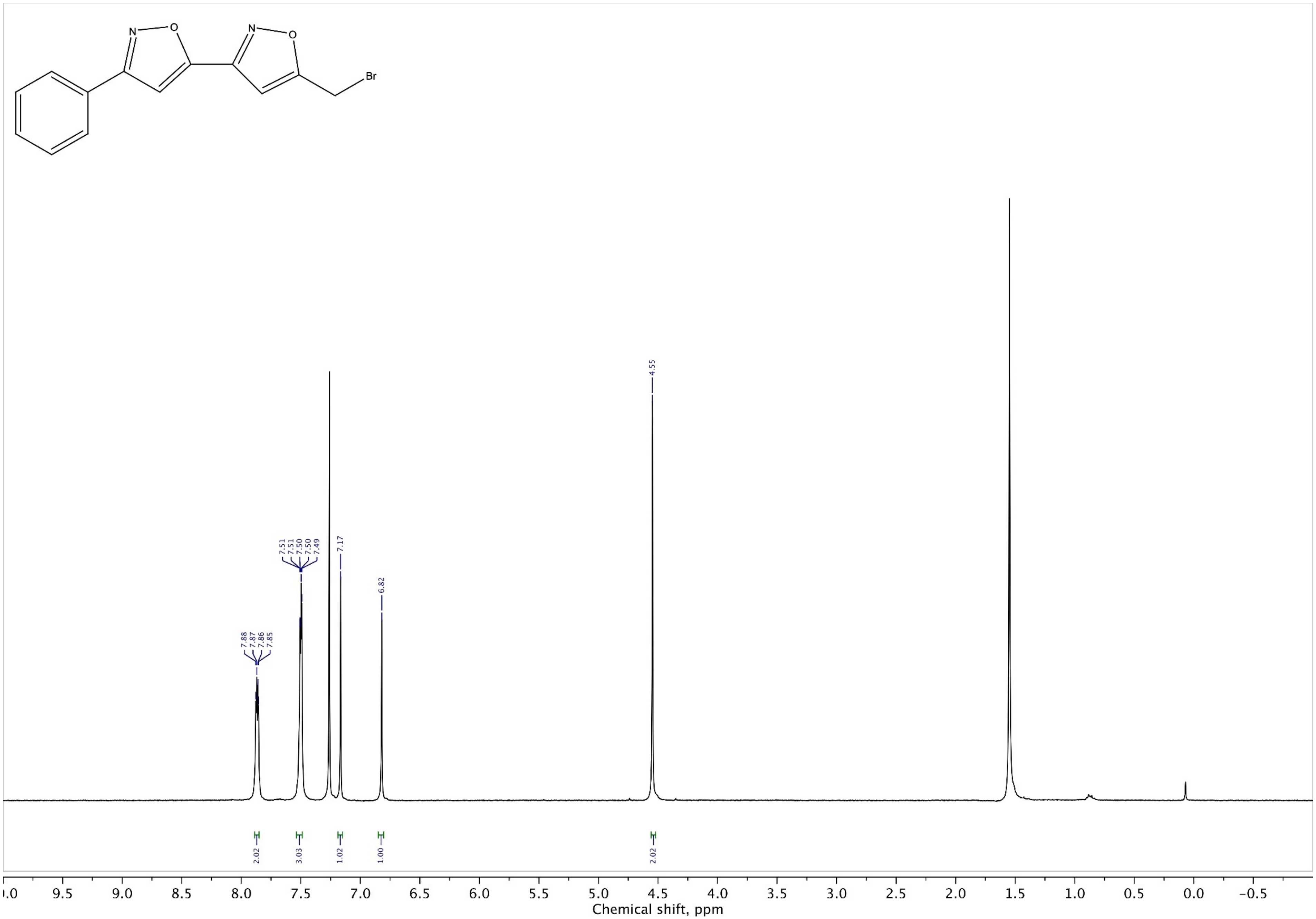

5-(Bromomethyl)-3'-phenyl-3,5'-biisoxazole (4h), $^{13}C\{^1H\}$ NMR, $CDCl_3$, 100 MHz

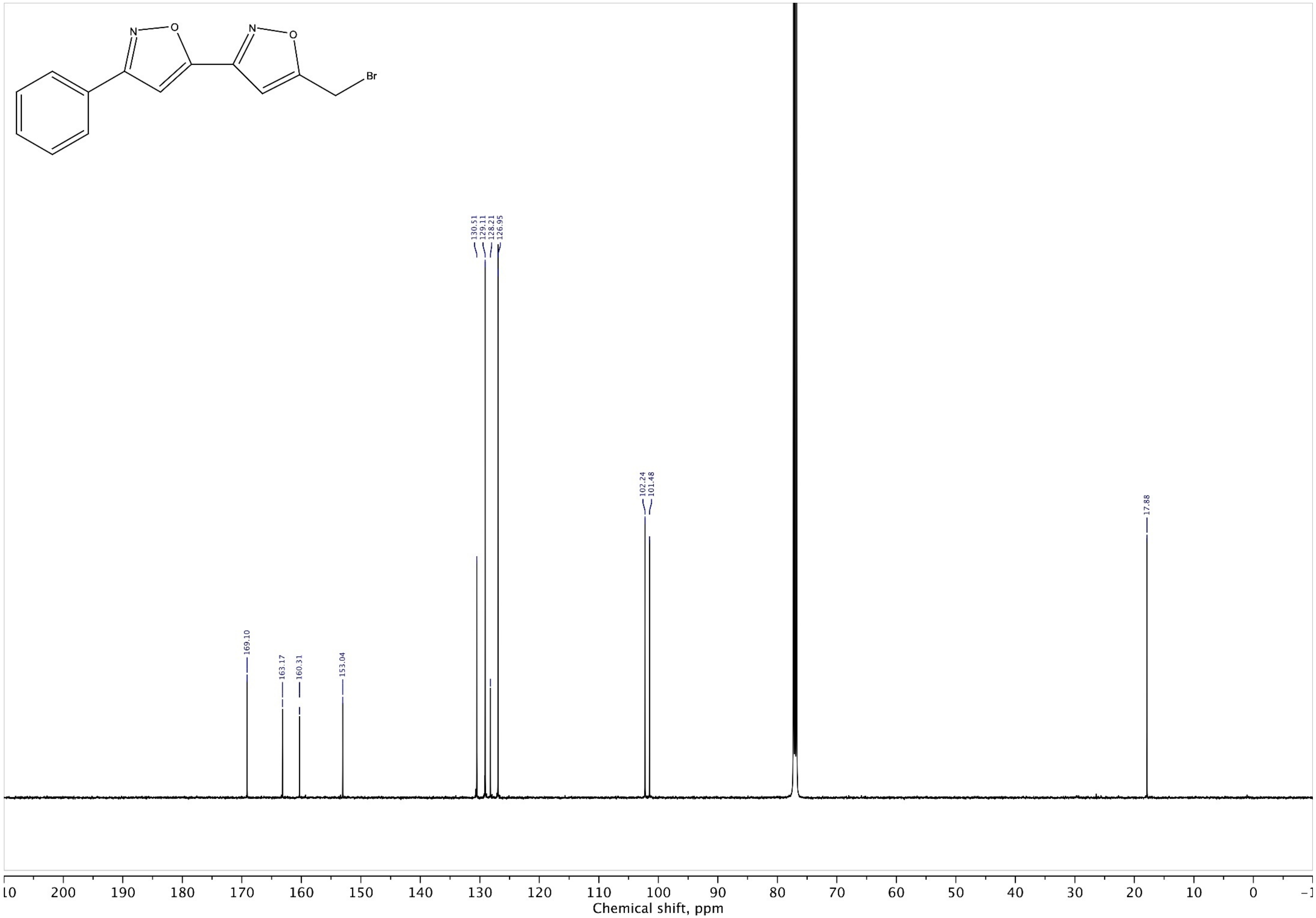

5-(Bromomethyl)-3'-phenyl-3,5'-biisoxazole (4h), DEPT, $CDCl_3$, 100 MHz

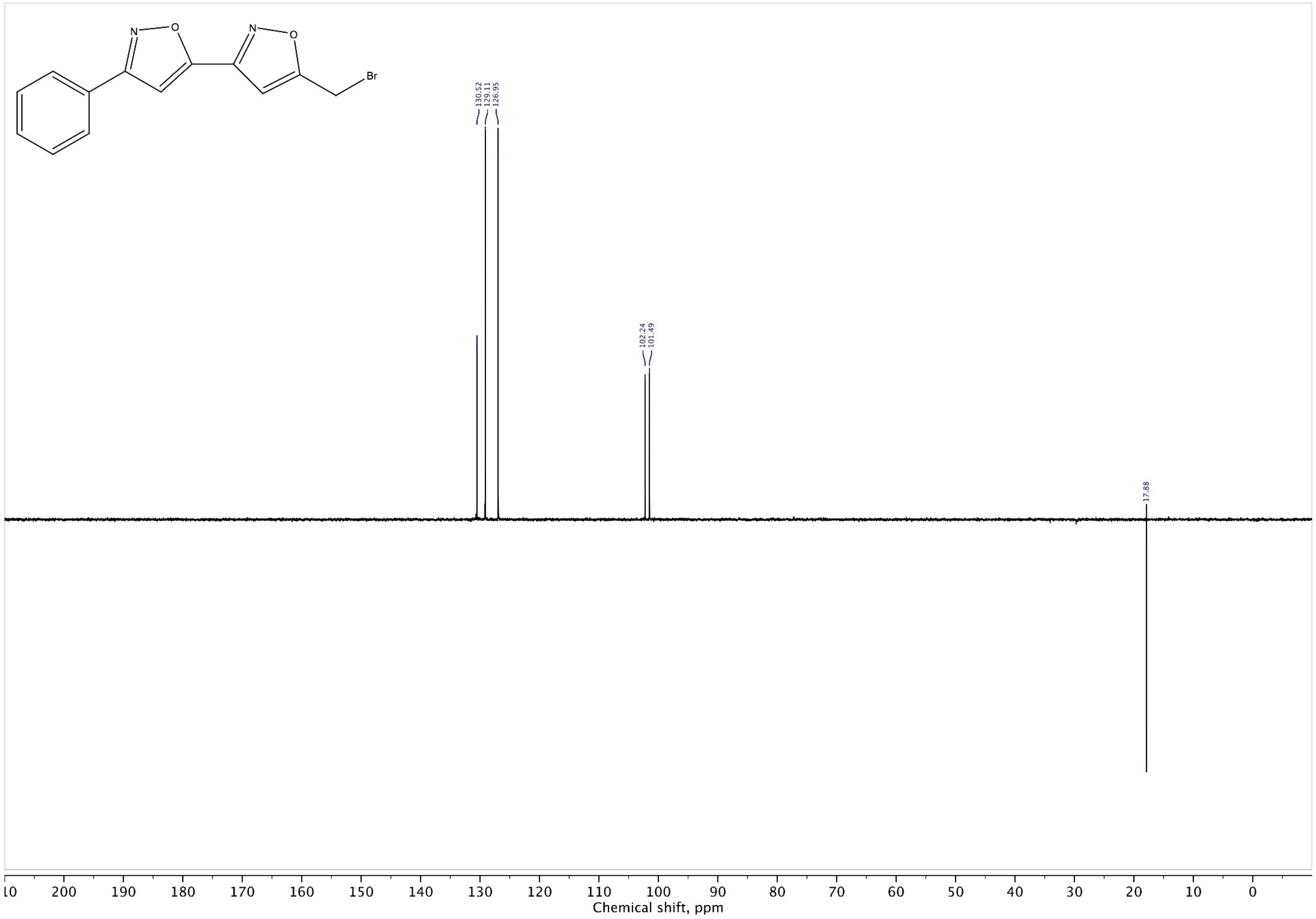

3'-Phenyl-5-(trimethylsilyl)-3,5'-biisoxazole (4i), ^{1}H NMR, $CDCl_3$, 400 MHz

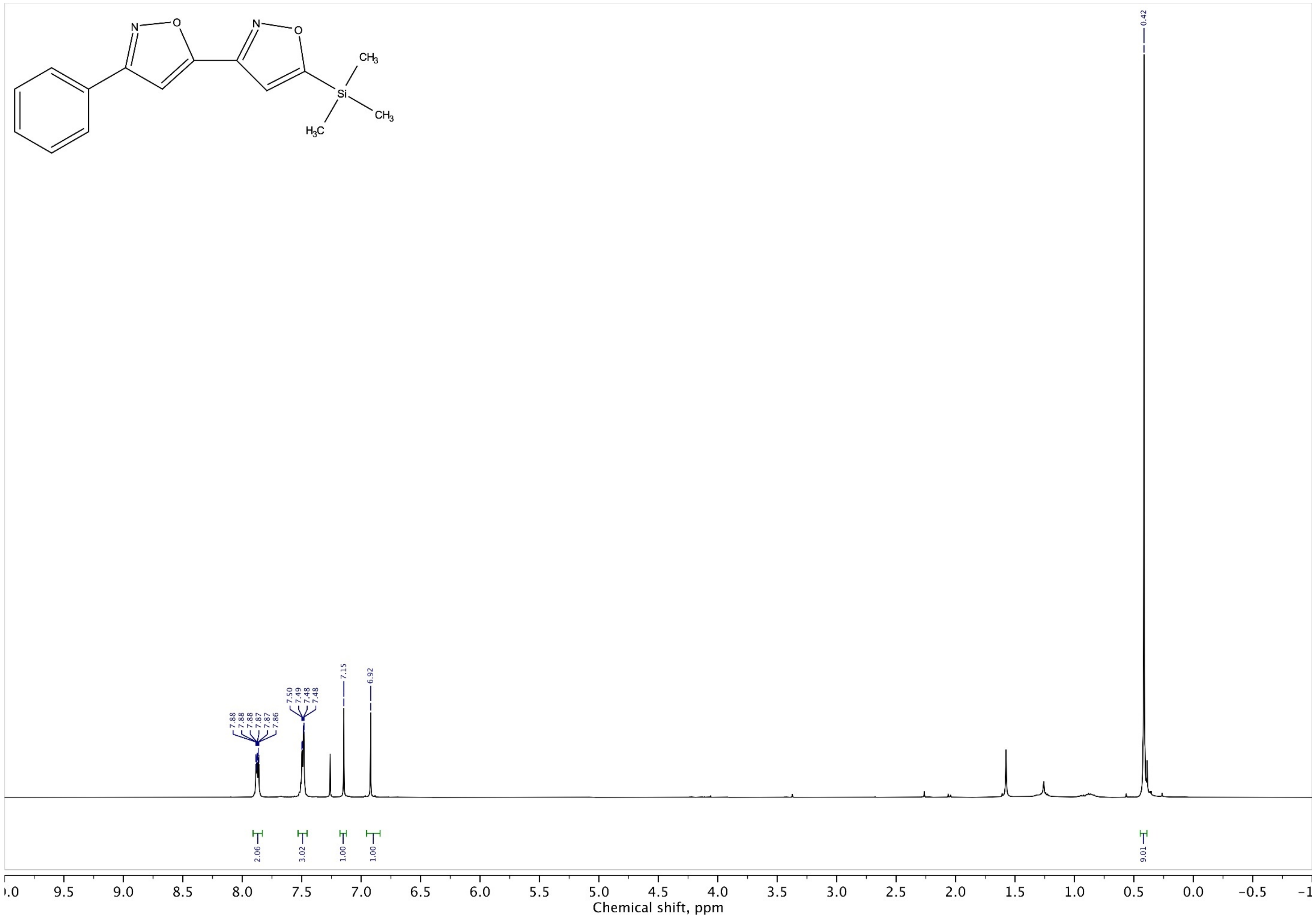

3'-Phenyl-5-(trimethylsilyl)-3,5'-biisoxazole (4i), $^{13}C\{^1H\}$ NMR, $CDCl_3$, 100 MHz

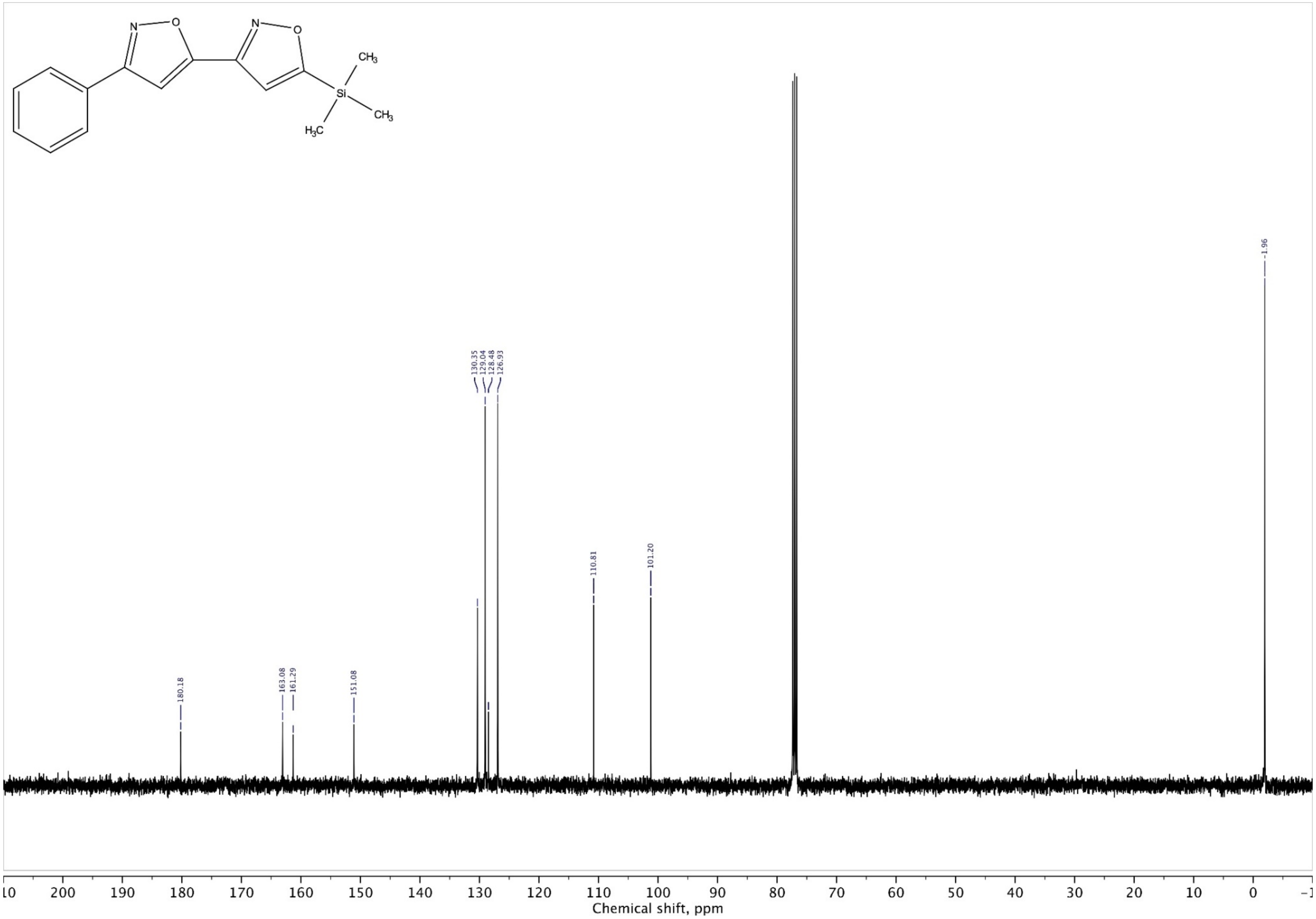

3'-Phenyl-5-(trimethylsilyl)-3,5'-biisoxazole (4i), DEPT, $CDCl_3$, 100 MHz

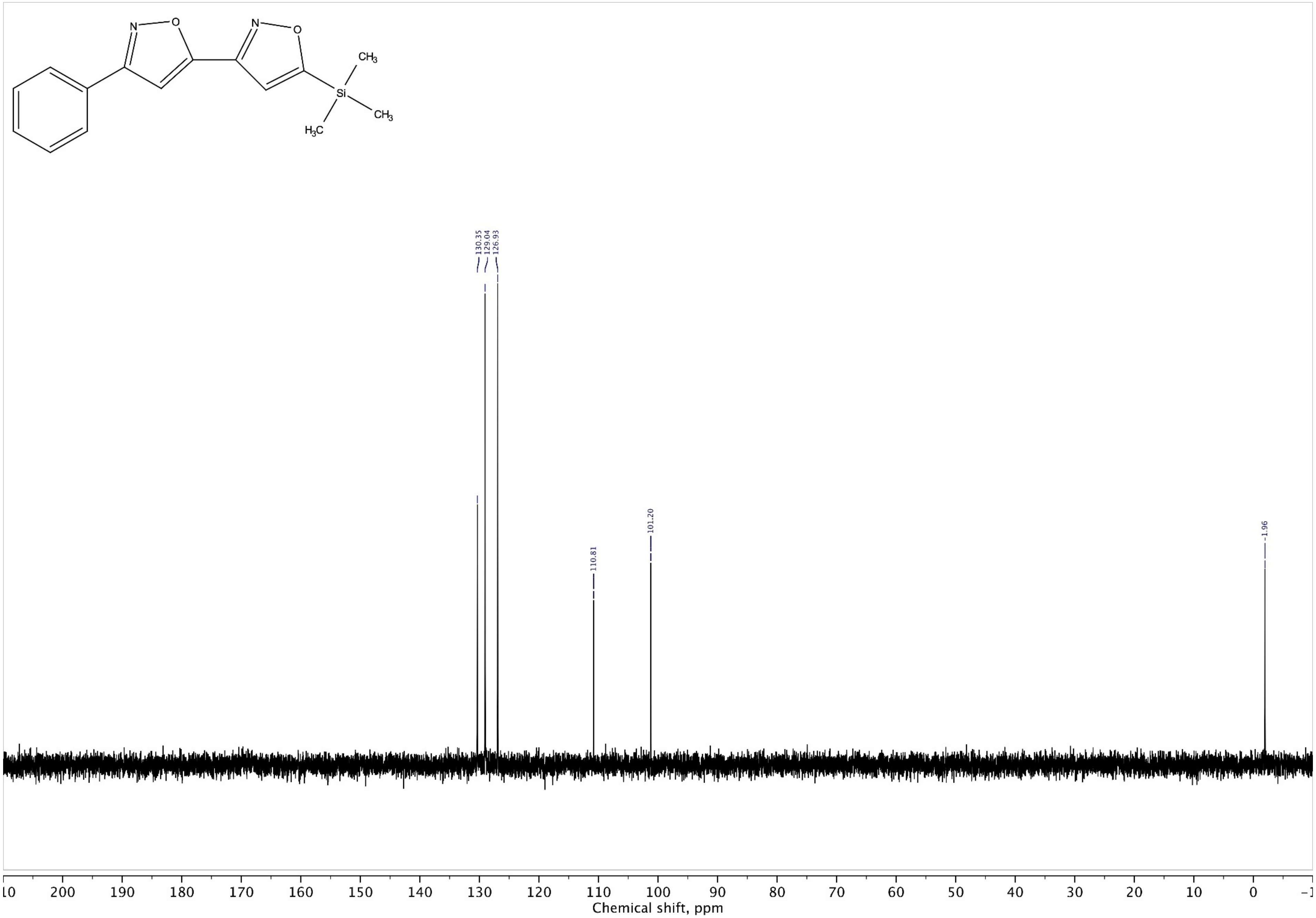

Methyl 3'-(p-tolyl)-[3,5'-biisoxazole]-5-carboxylate (4j), ^{1}H NMR, $CDCl_3$, 400 MHz

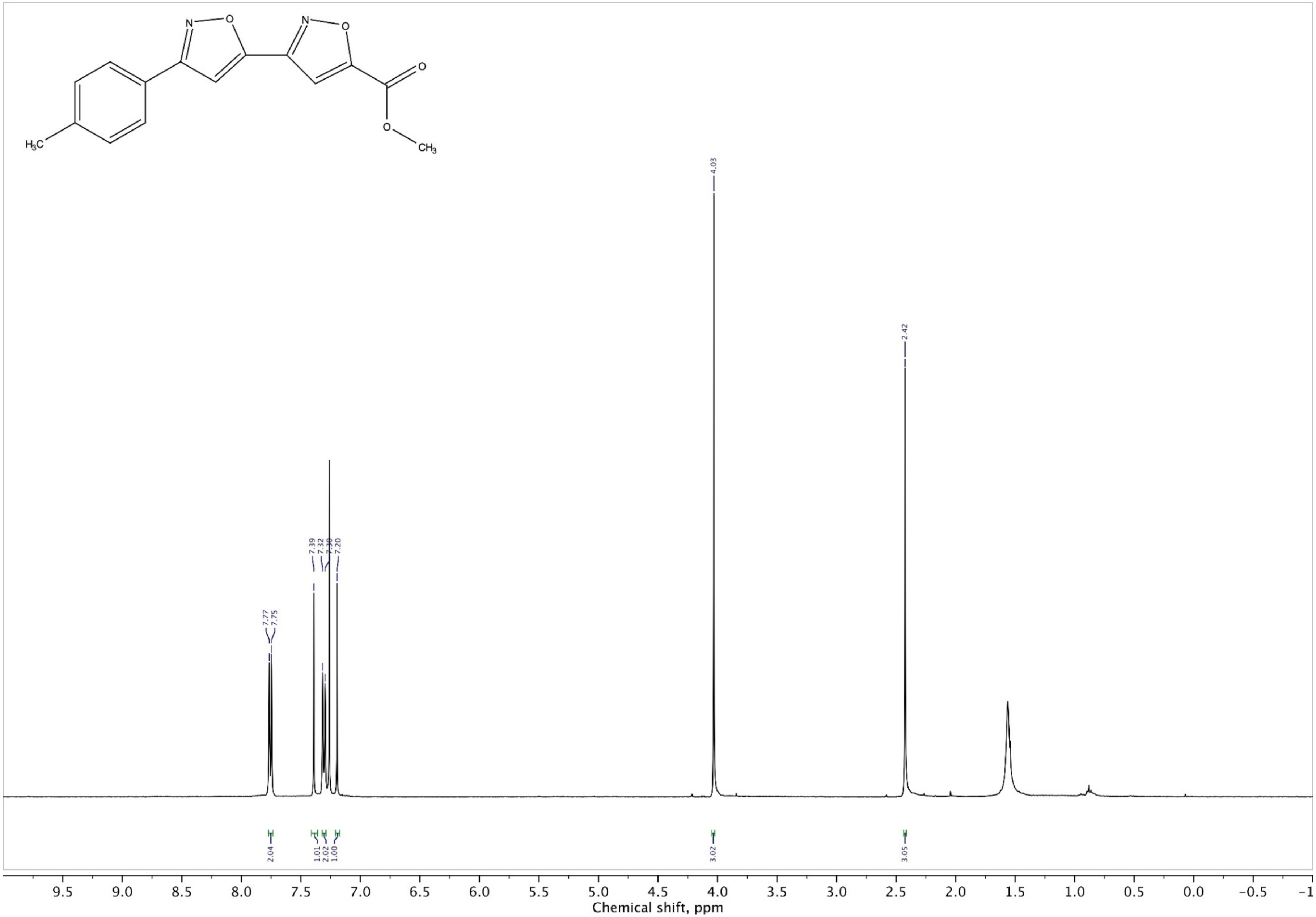

Methyl 3'-(p-tolyl)-[3,5'-biisoxazole]-5-carboxylate (4j), $^{13}C\{^1H\}$ NMR, $CDCl_3$, 100 MHz

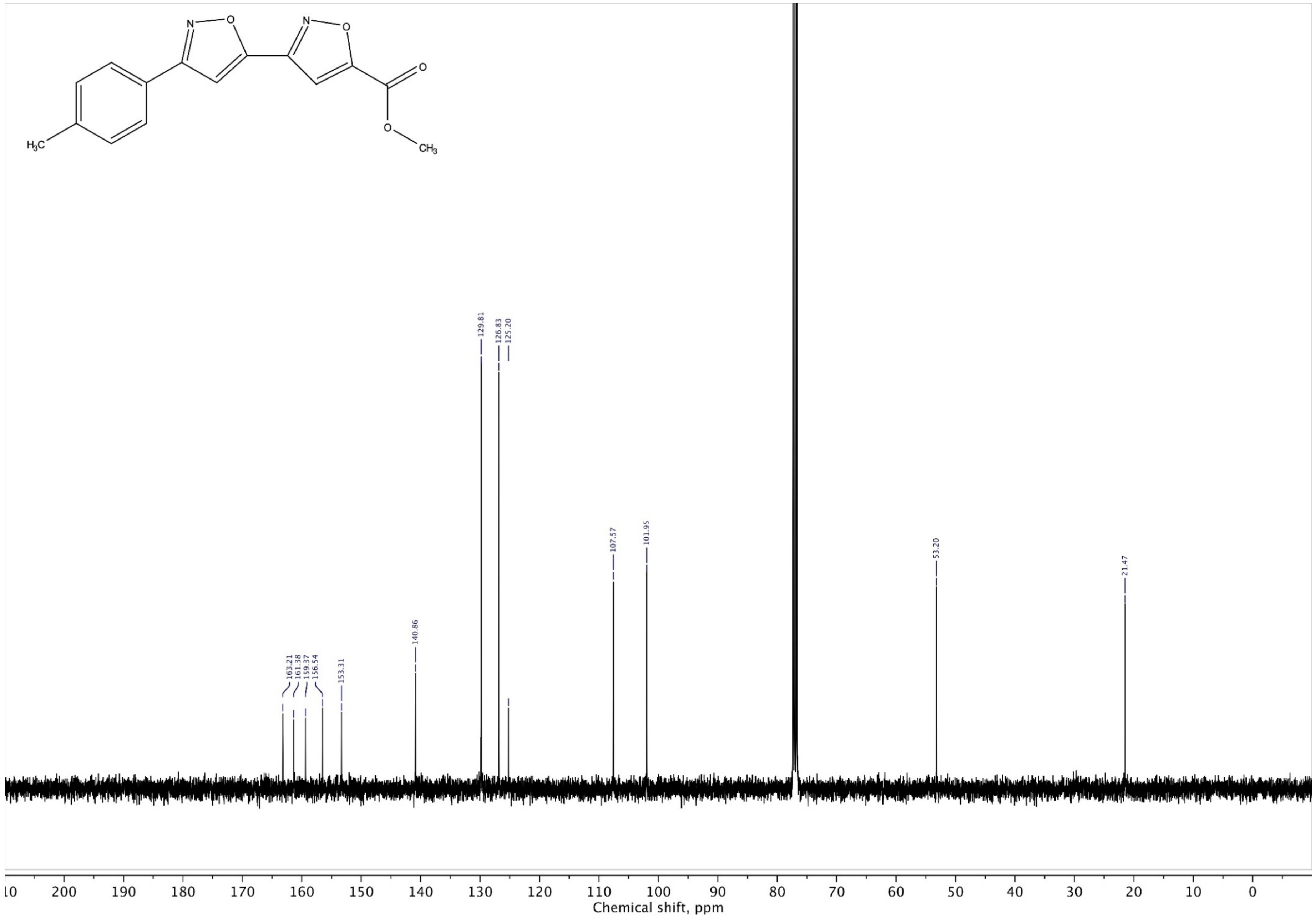

Methyl 3'-(p-tolyl)-[3,5'-biisoxazole]-5-carboxylate (4j), DEPT, $CDCl_3$, 100 MHz

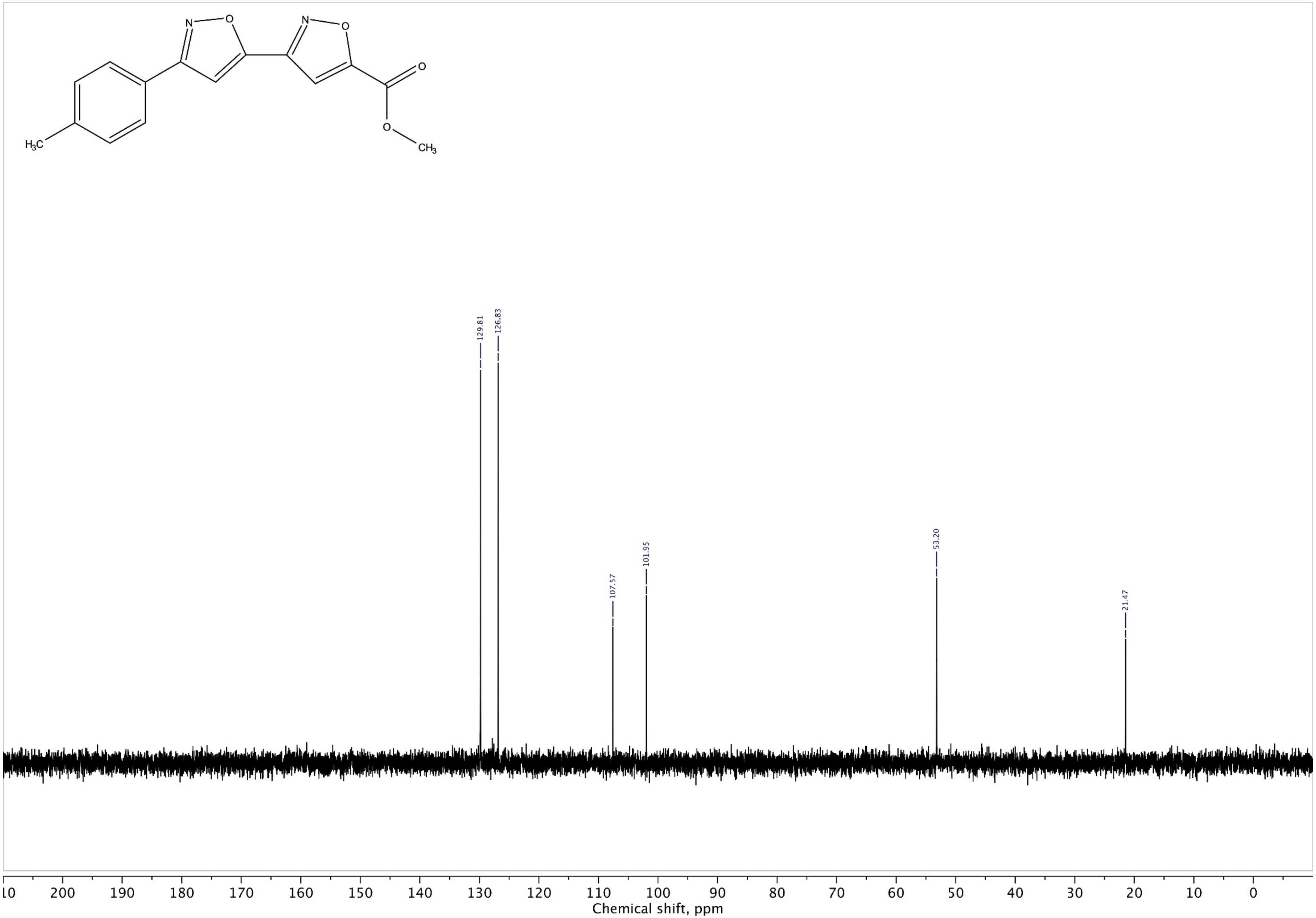

Methyl 3'-(4-(*tert*-butyl)phenyl)-[3,5'-biisoxazole]-5-carboxylate (4k), ^{1}H NMR, $CDCl_3$, 400 MHz

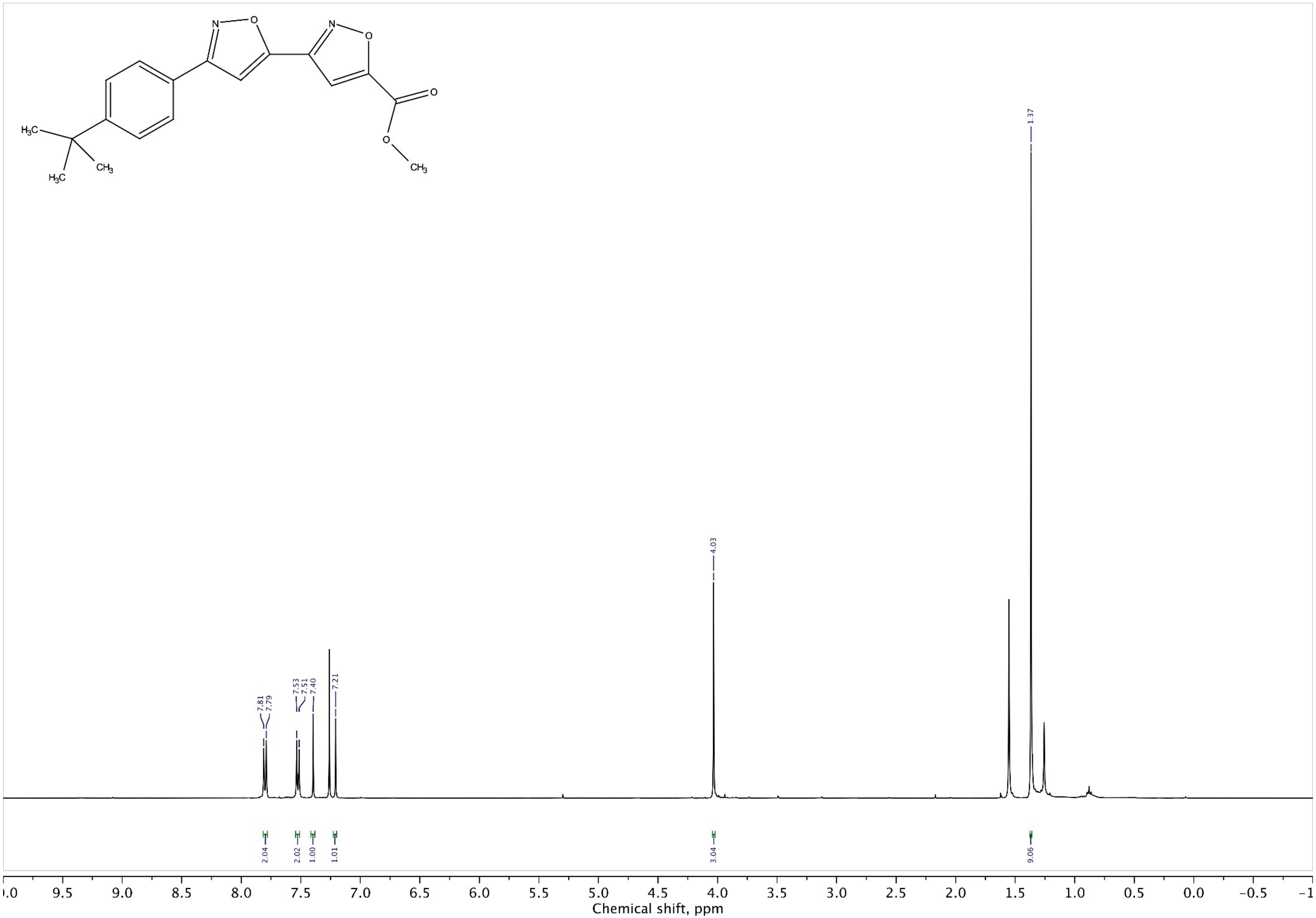

Methyl 3'-(4-(*tert*-butyl)phenyl)-[3,5'-biisoxazole]-5-carboxylate (4k), $^{13}C\{^1H\}$ NMR, $CDCl_3$, 100 MHz

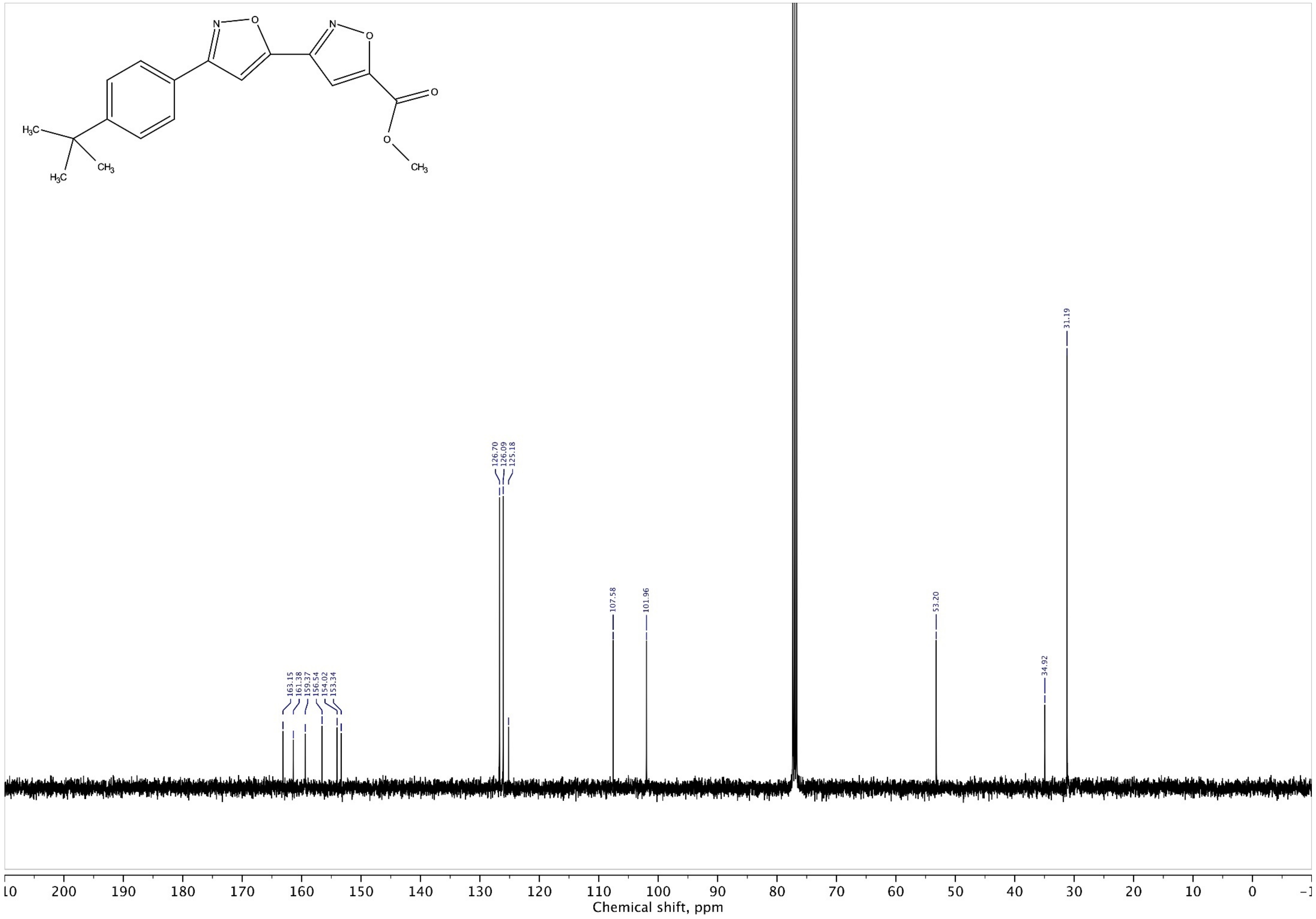

Methyl 3'-(4-(*tert*-butyl)phenyl)-[3,5'-biisoxazole]-5-carboxylate (4k), DEPT, $CDCl_3$, 100 MHz

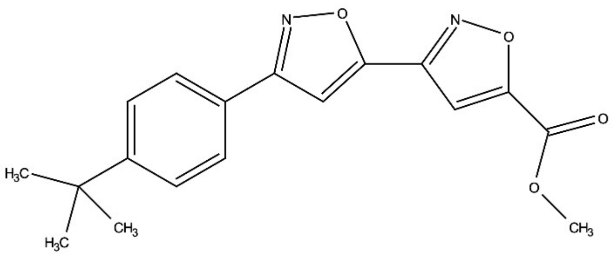

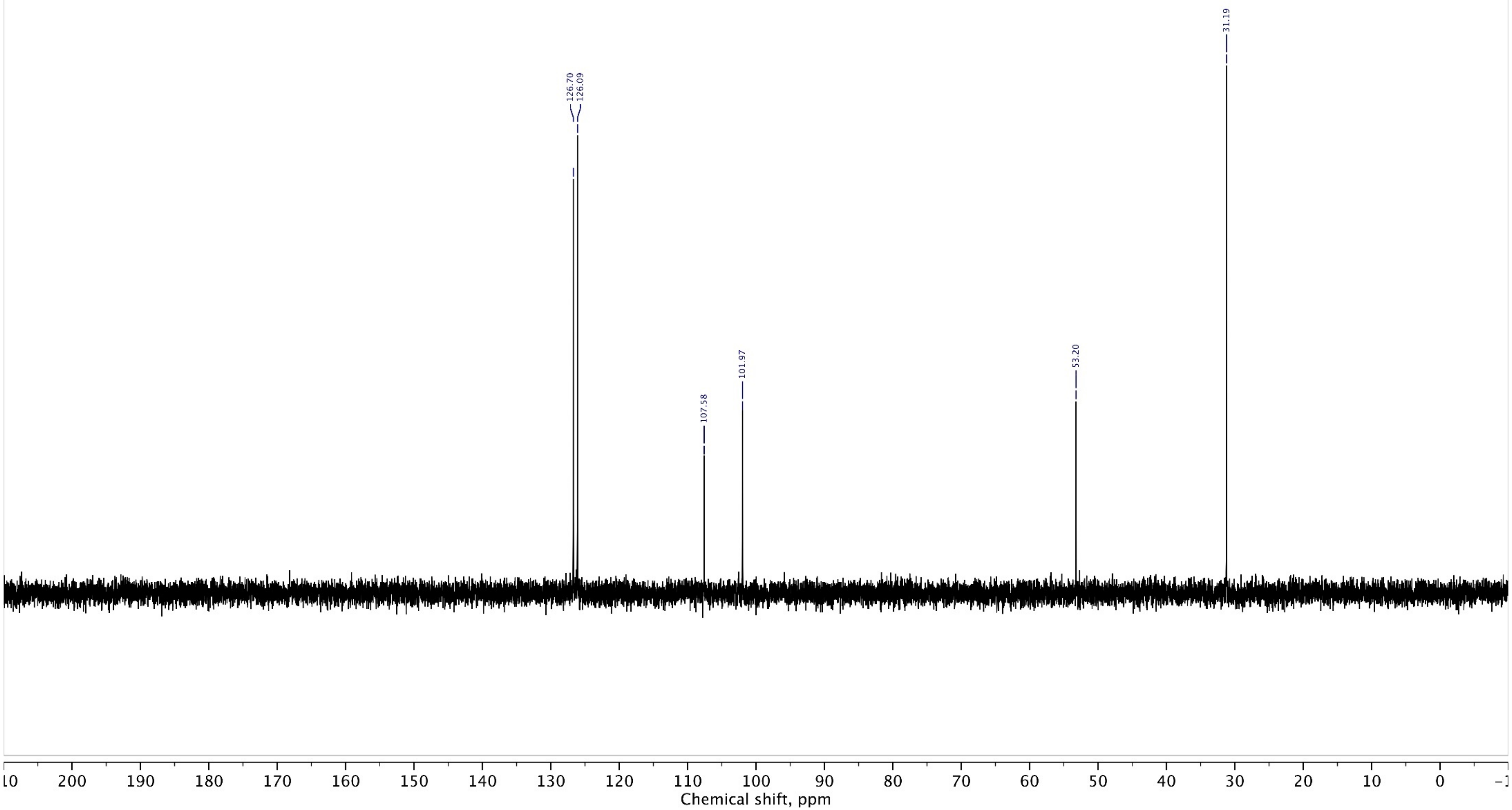

Methyl 3'-(4-methoxyphenyl)-[3,5'-biisoxazole]-5-carboxylate (4l), ^{1}H NMR, DMSO-d_6, 400 MHz

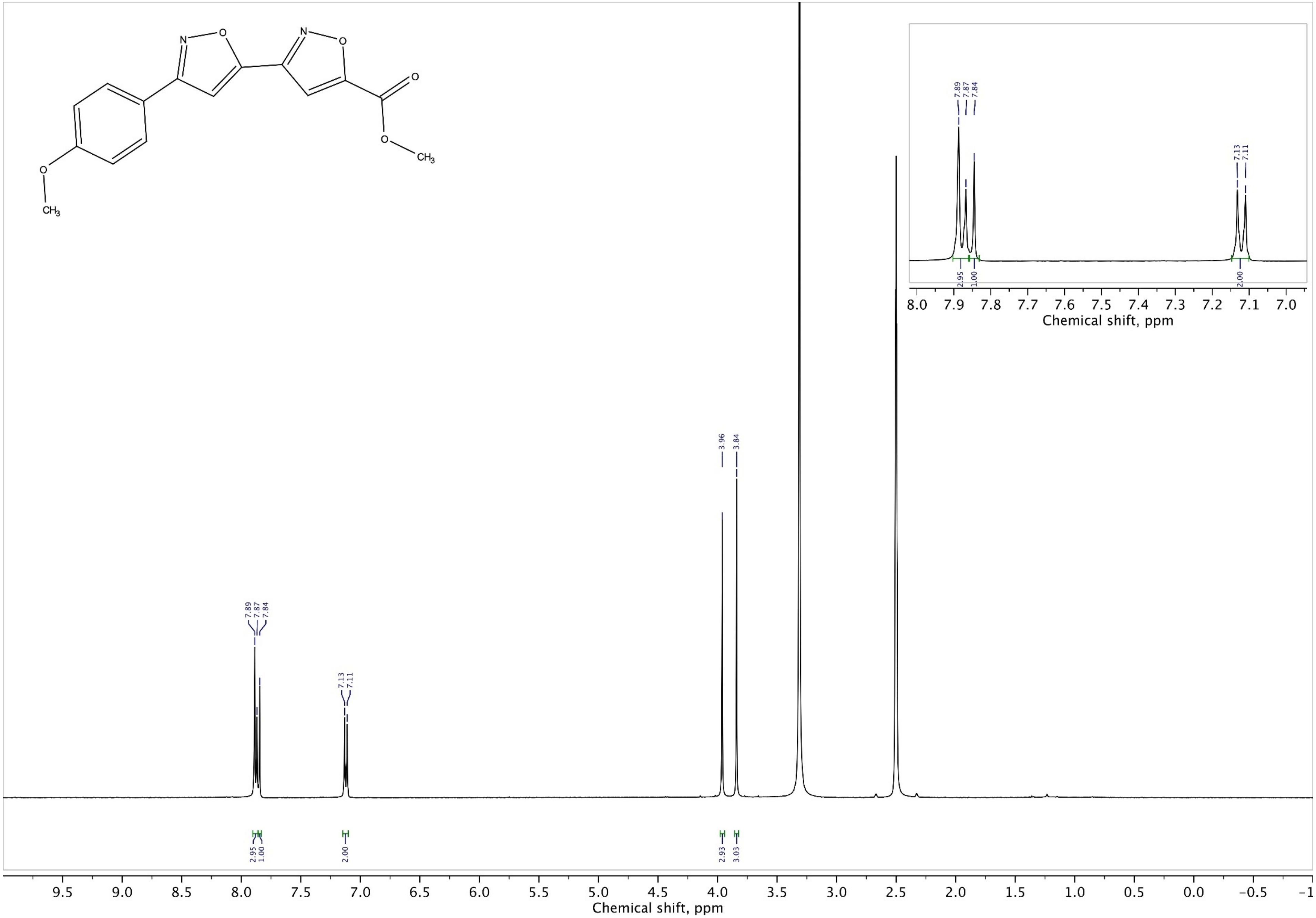

Methyl 3'-(4-methoxyphenyl)-[3,5'-biisoxazole]-5-carboxylate (4l), $^{13}C\{^1H\}$ NMR, DMSO-d_6, 100 MHz

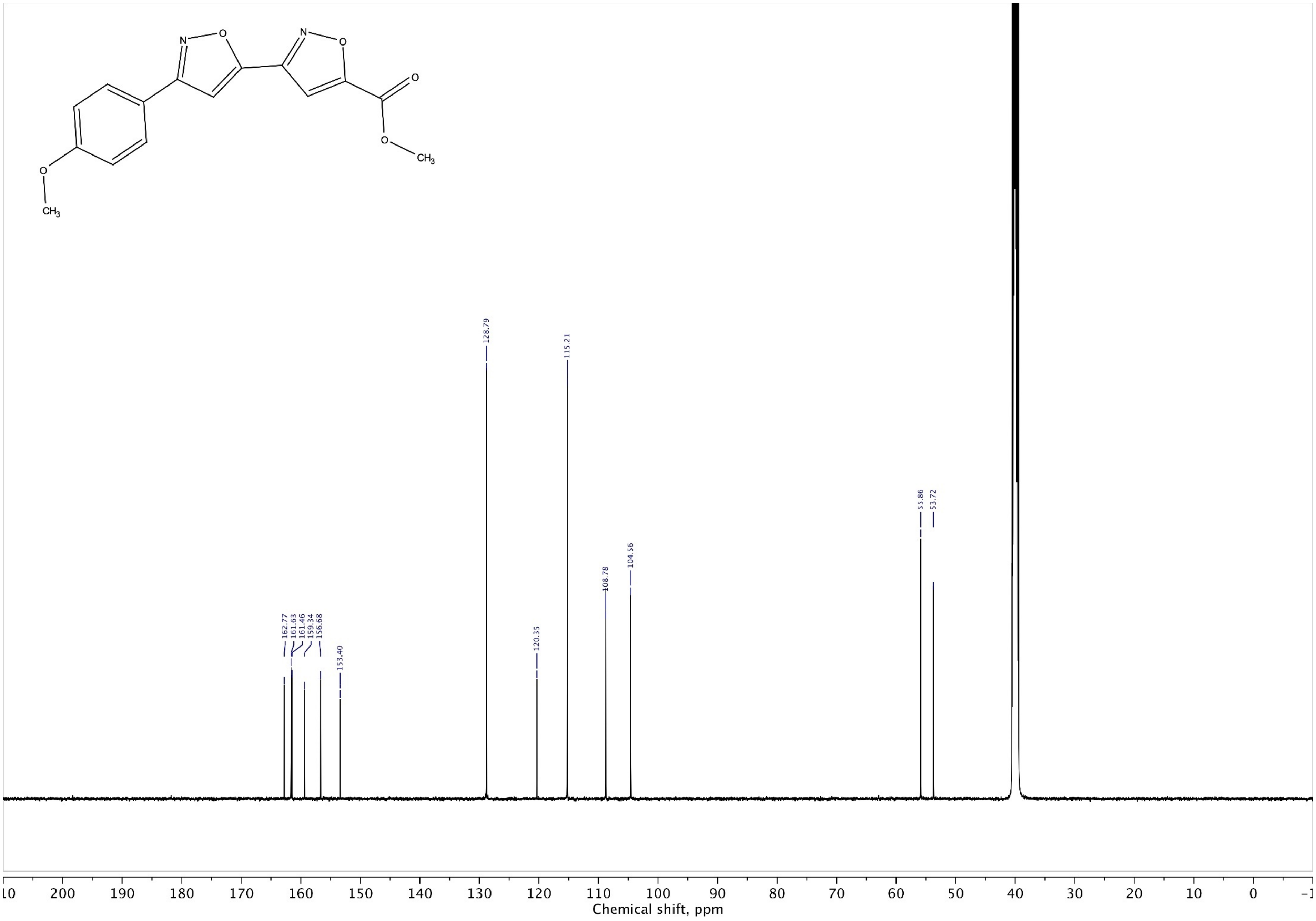

Methyl 3'-(4-methoxyphenyl)-[3,5'-biisoxazole]-5-carboxylate (4l), DEPT, DMSO-d_6, 100 MHz

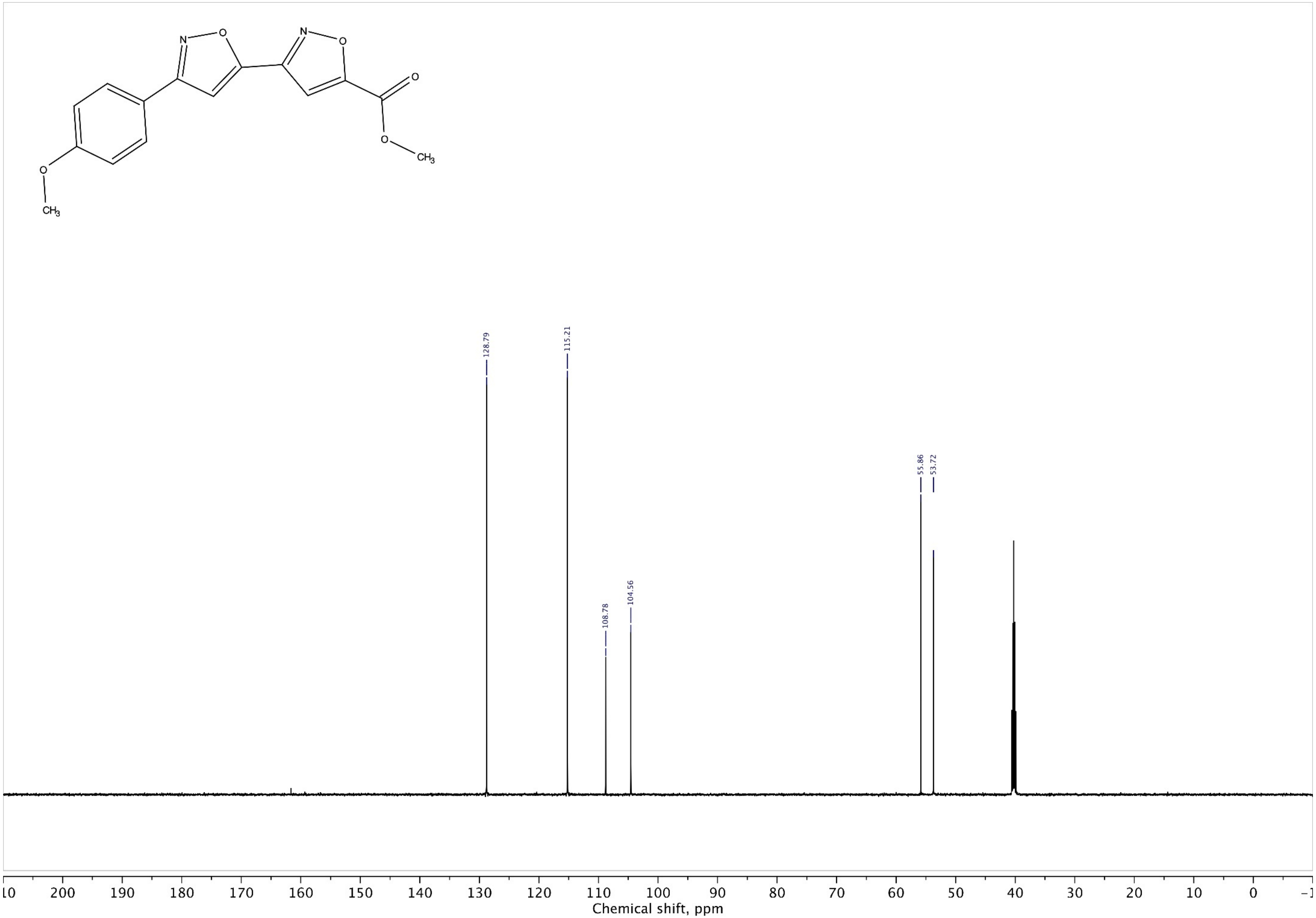

3'-(4-Methoxyphenyl)-5-(phenoxymethyl)-3,5'-biisoxazole (4m), ^{1}H NMR, DMSO-d_6, 400 MHz

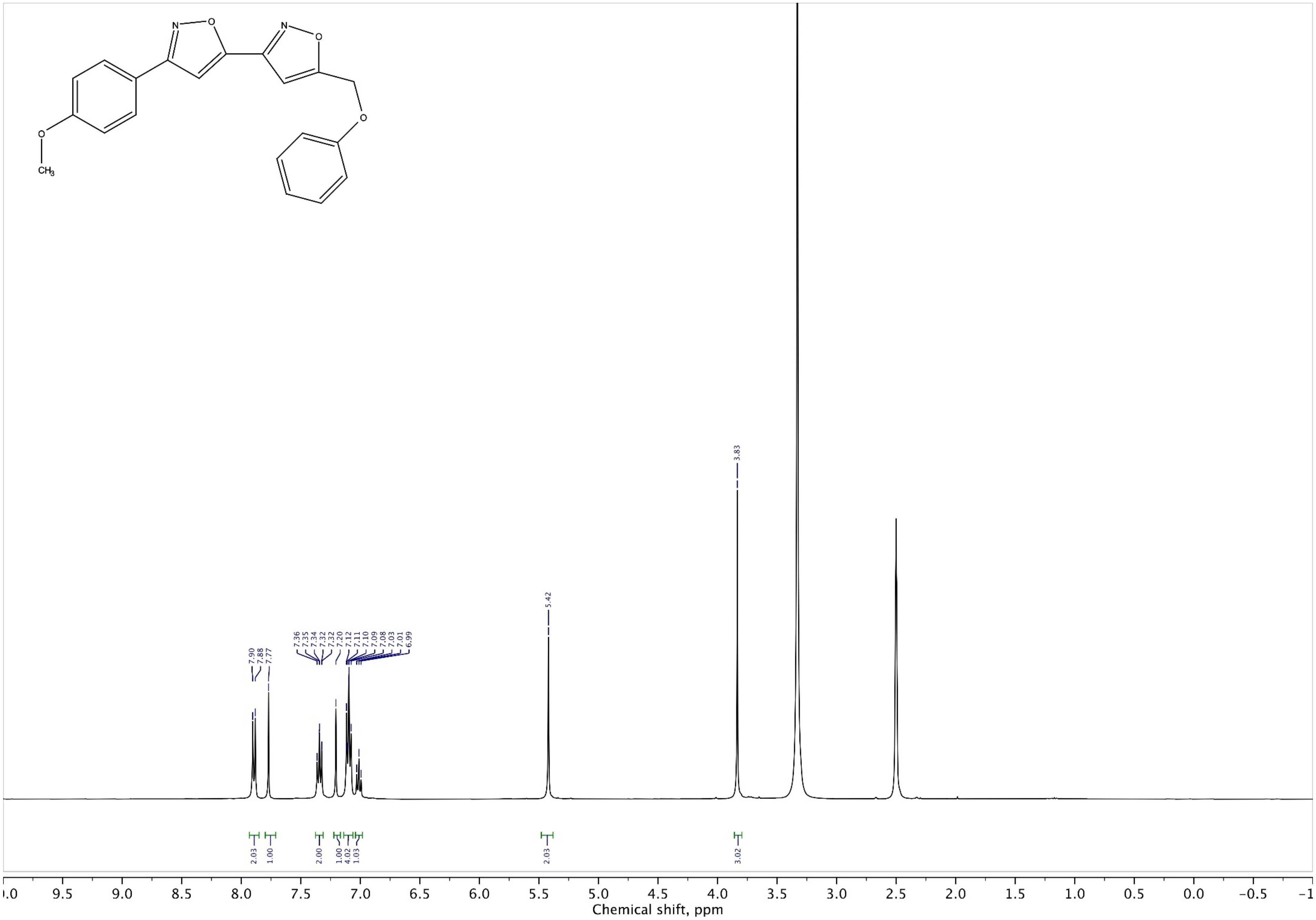

3'-(4-Methoxyphenyl)-5-(phenoxymethyl)-3,5'-biisoxazole (4m), $^{13}C\{^{1}H\}$ NMR, DMSO-d_6, 100 MHz

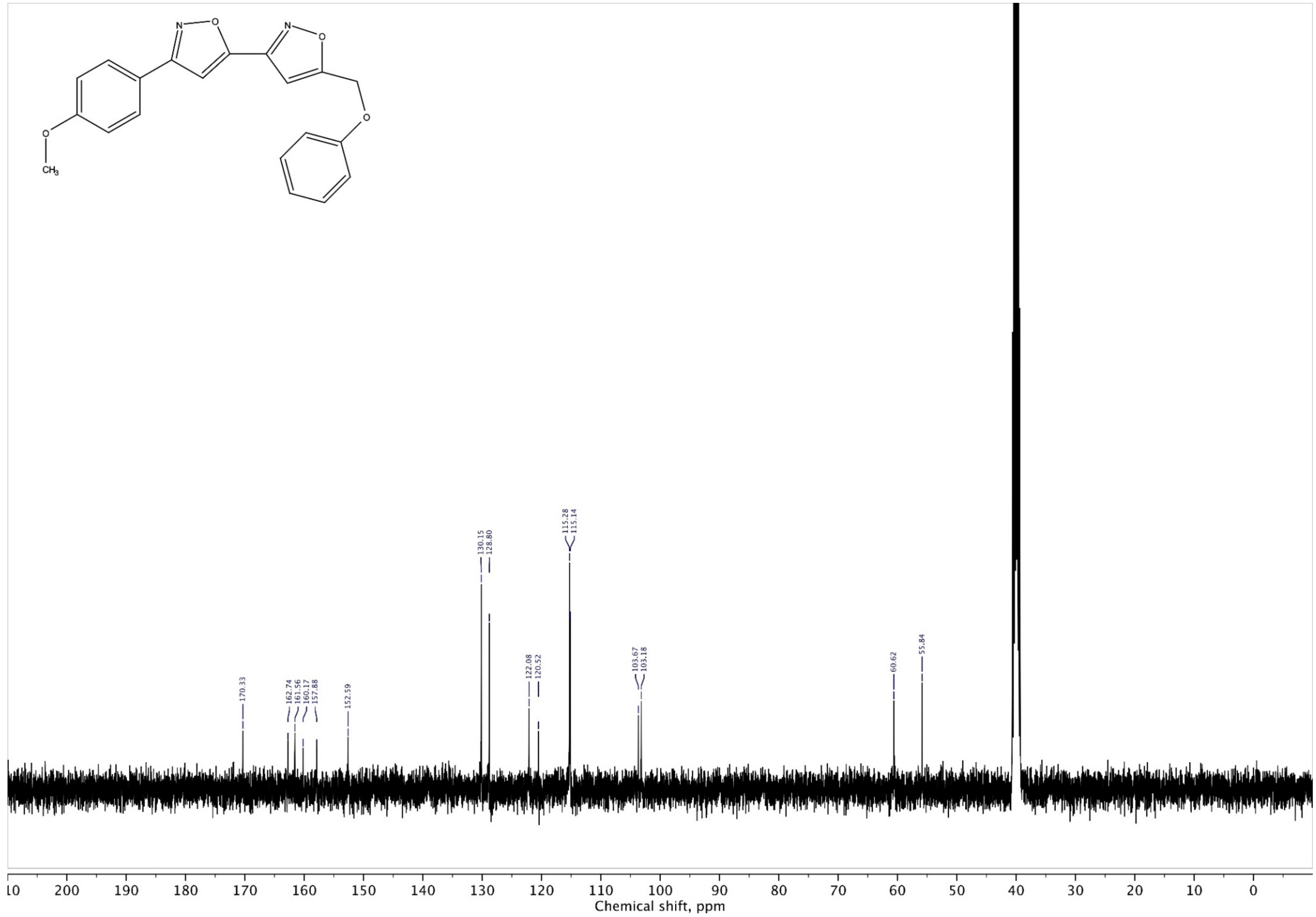

3'-(4-Methoxyphenyl)-5-(phenoxymethyl)-3,5'-biisoxazole (4m), DEPT, DMSO-d_6, 100 MHz

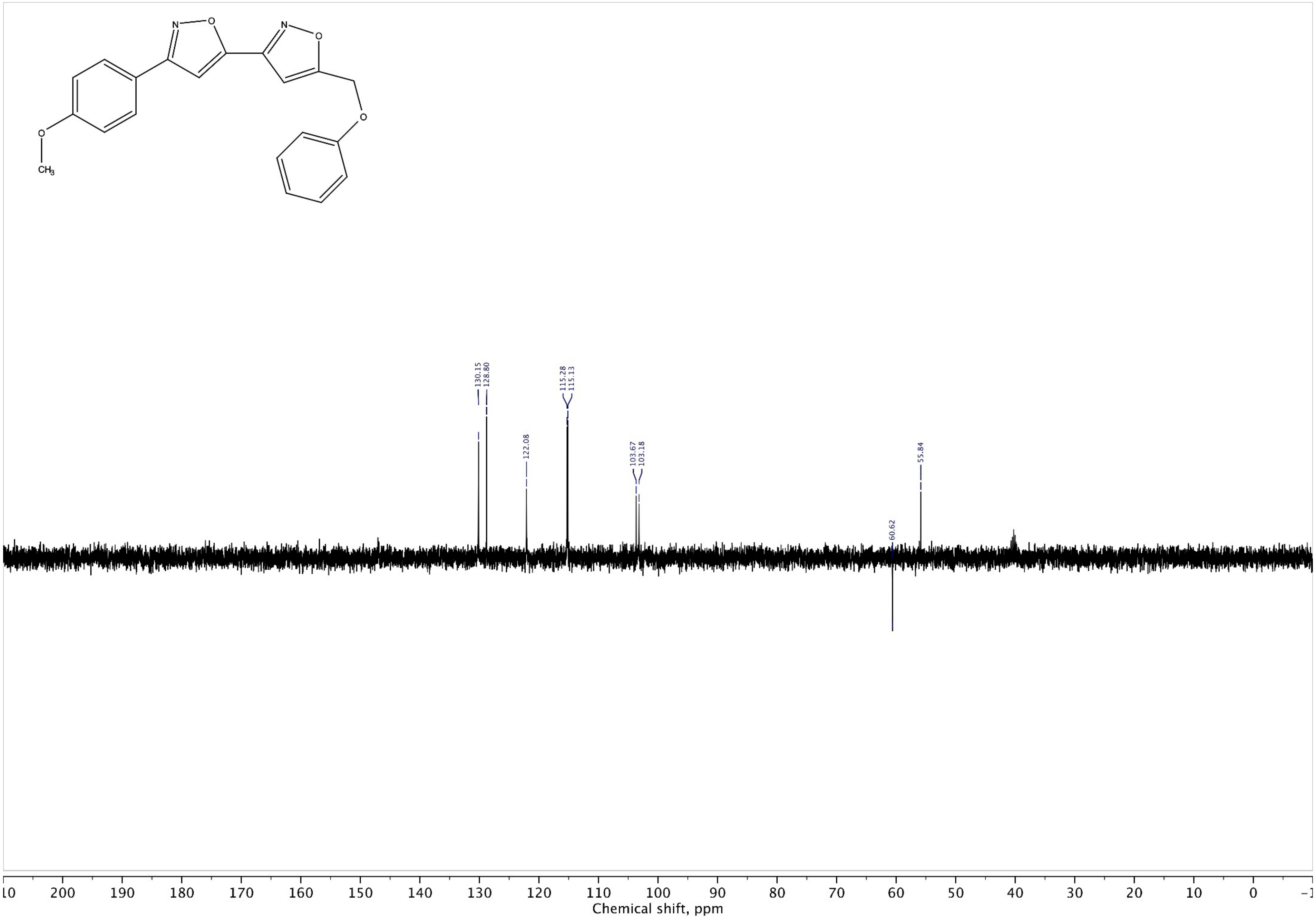

5-(Bromomethyl)-3'-(3-methoxyphenyl)-3,5'-biisoxazole (4n), ^1H NMR, $CDCl_3$, 400 MHz

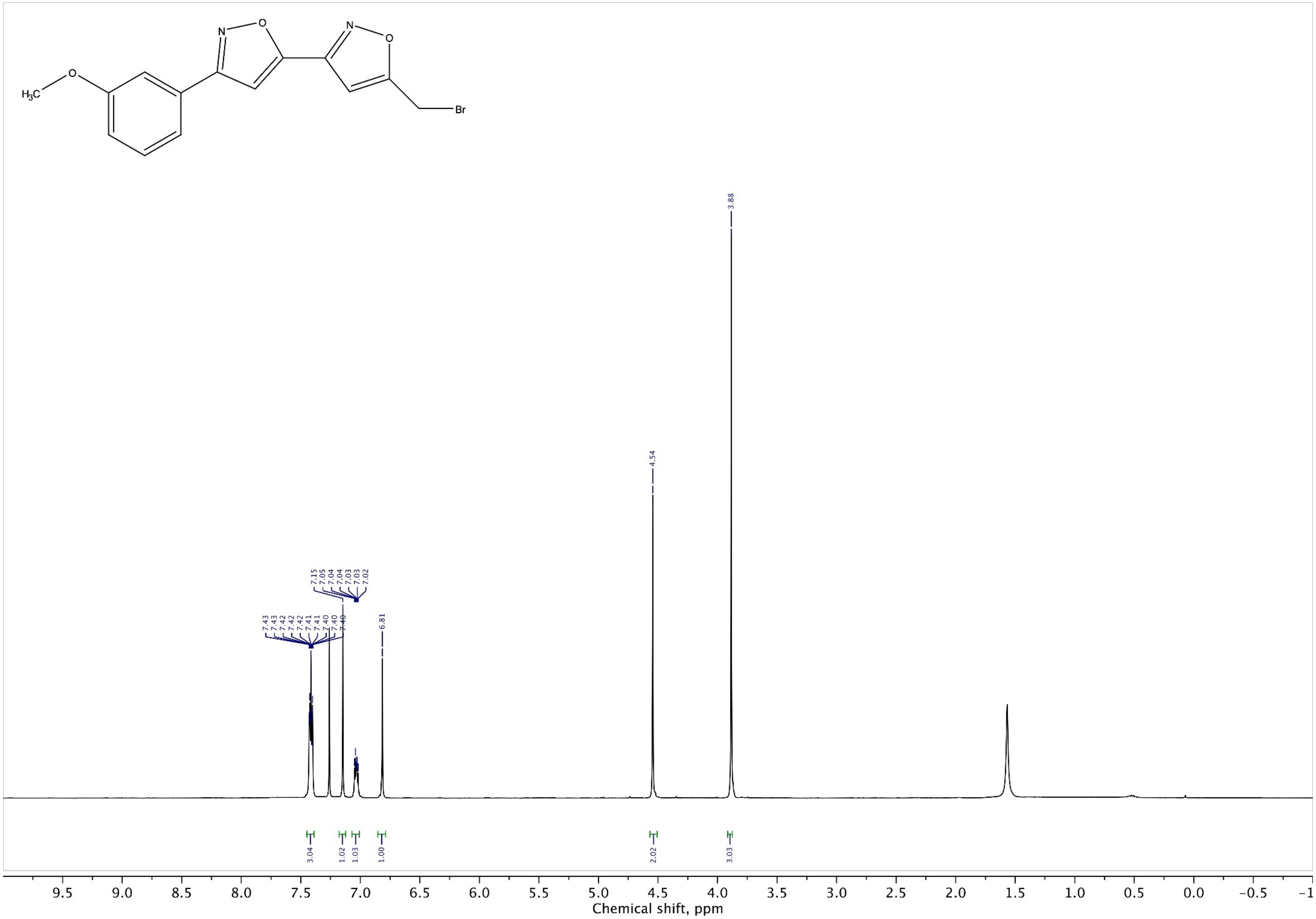

5-(Bromomethyl)-3'-(3-methoxyphenyl)-3,5'-biisoxazole (4n), $^{13}C\{^{1}H\}$ NMR, $CDCl_3$, 100 MHz

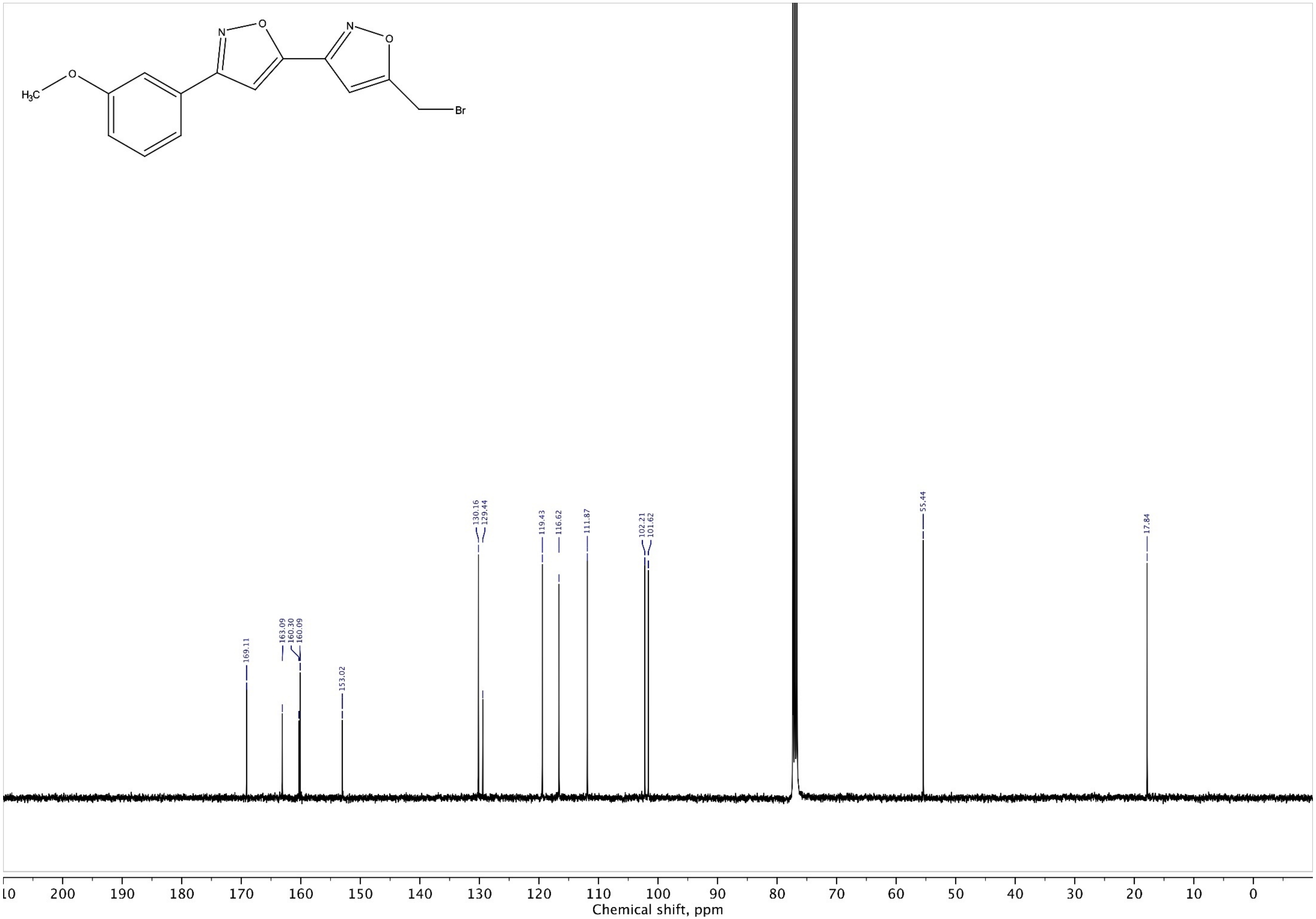

5-(Bromomethyl)-3'-(3-methoxyphenyl)-3,5'-biisoxazole (4n), DEPT, $CDCl_3$, 100 MHz

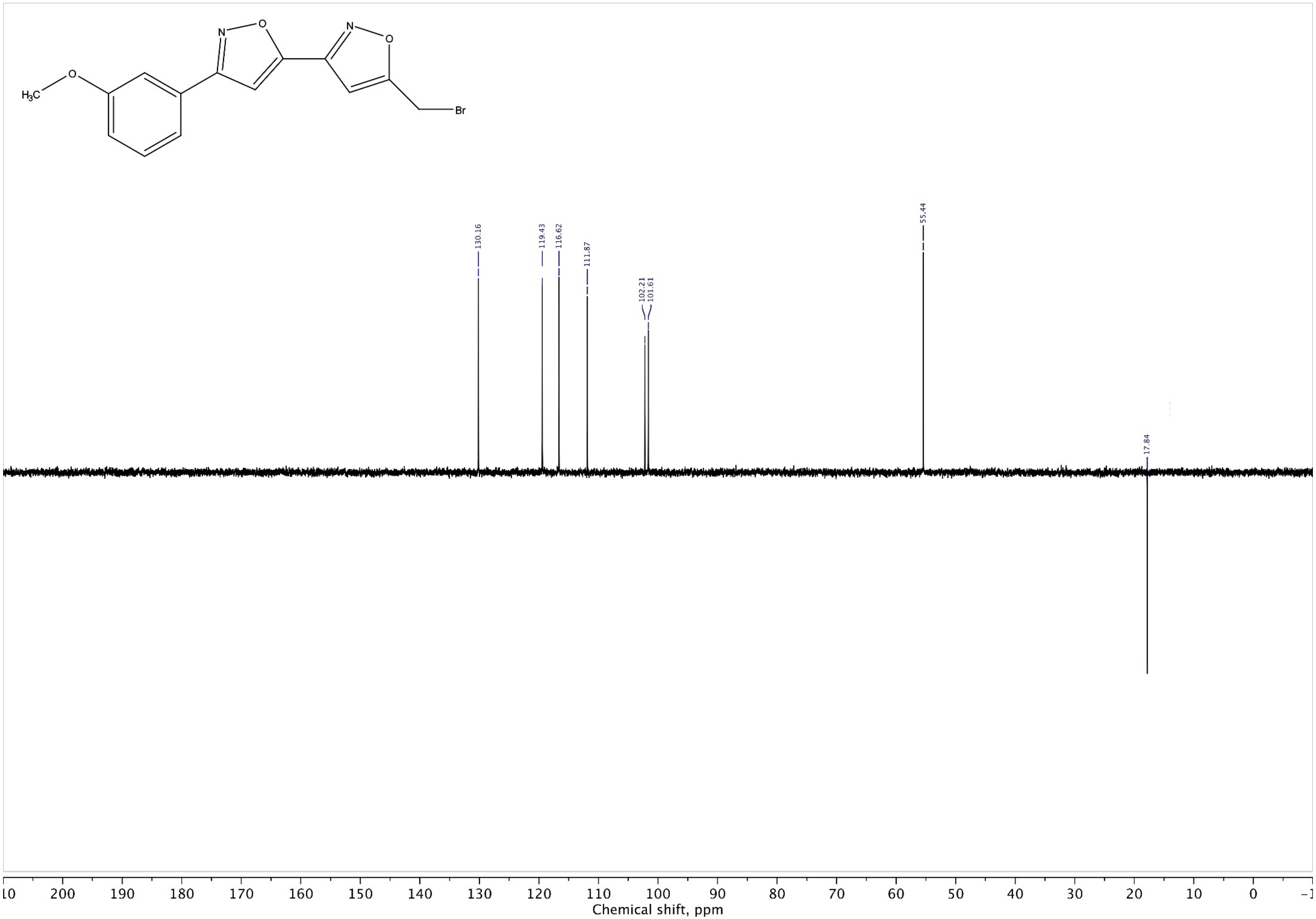

3'-(3,4-Dimethoxyphenyl)-5-(trimethylsilyl)-3,5'-biisoxazole (4o), ^{1}H NMR, CDCl₃, 400 MHz

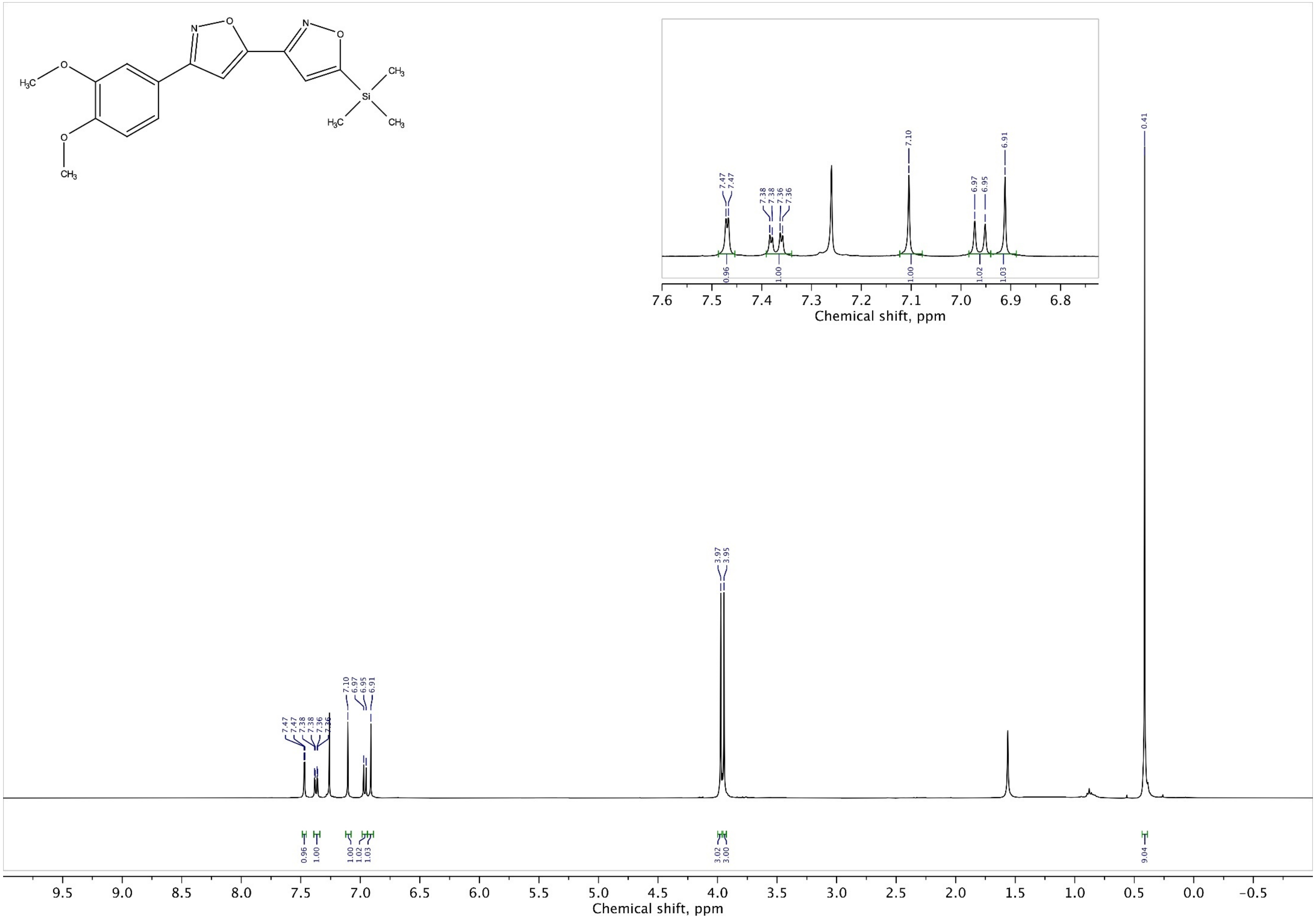

3'-(3,4-Dimethoxyphenyl)-5-(trimethylsilyl)-3,5'-biisoxazole (4o), $^{13}C\{^{1}H\}$ NMR, $CDCl_3$, 100 MHz

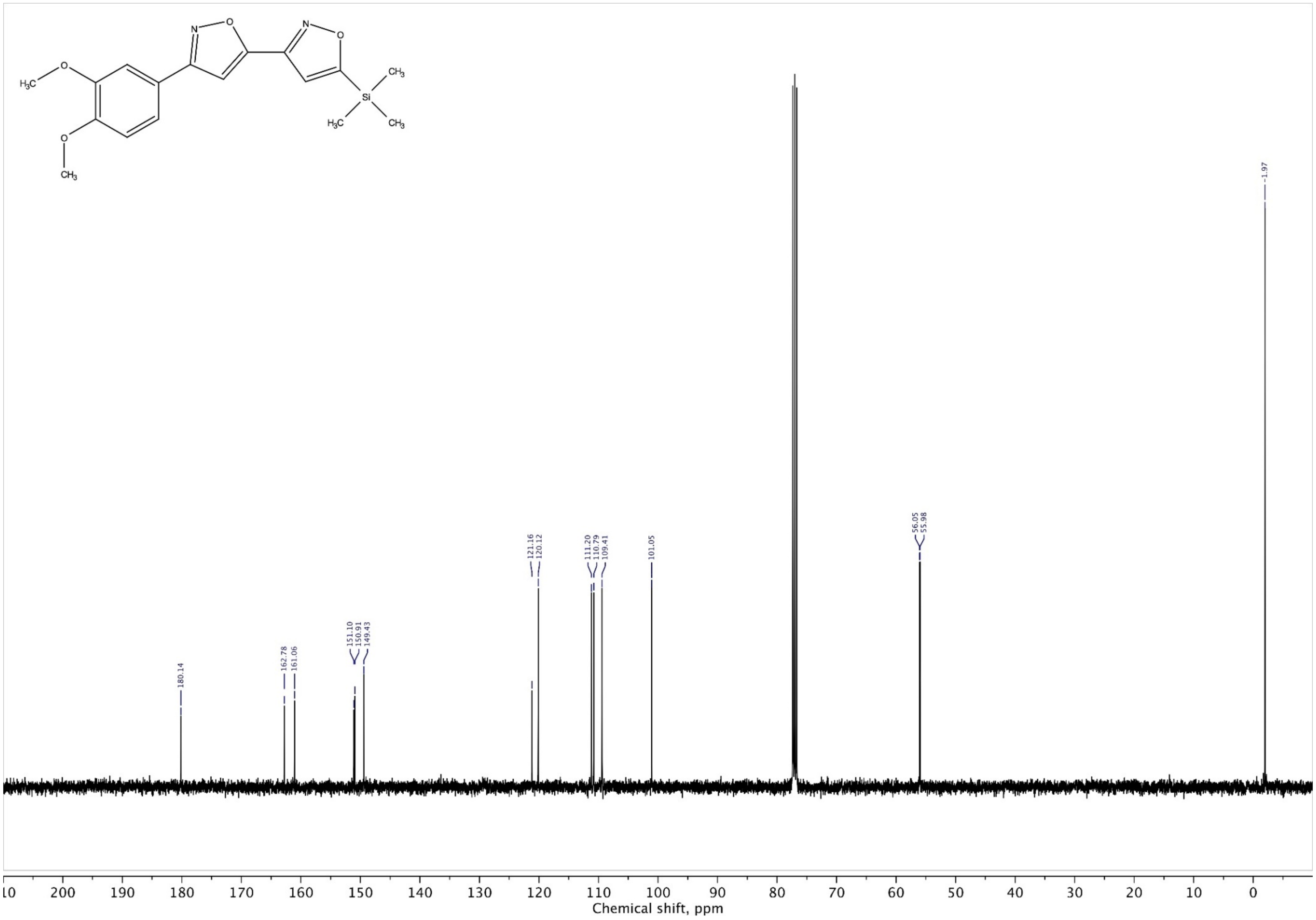

3'-(3,4-Dimethoxyphenyl)-5-(trimethylsilyl)-3,5'-biisoxazole (4o), DEPT, $CDCl_3$, 100 MHz

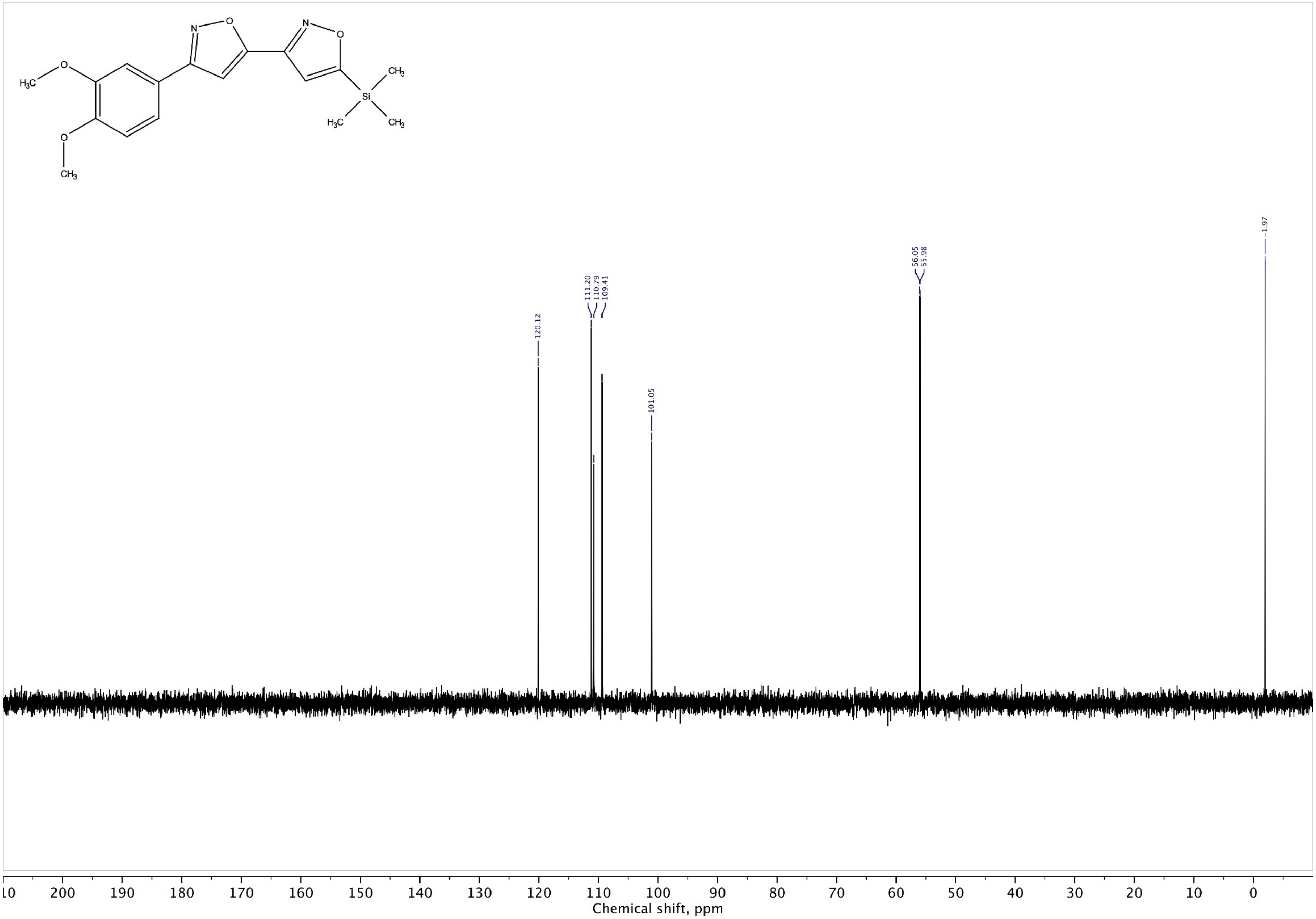

Methyl 3'-(4-fluorophenyl)-[3,5'-biisoxazole]-5-carboxylate (4p), ^{1}H NMR, DMSO-d_6, 400 MHz

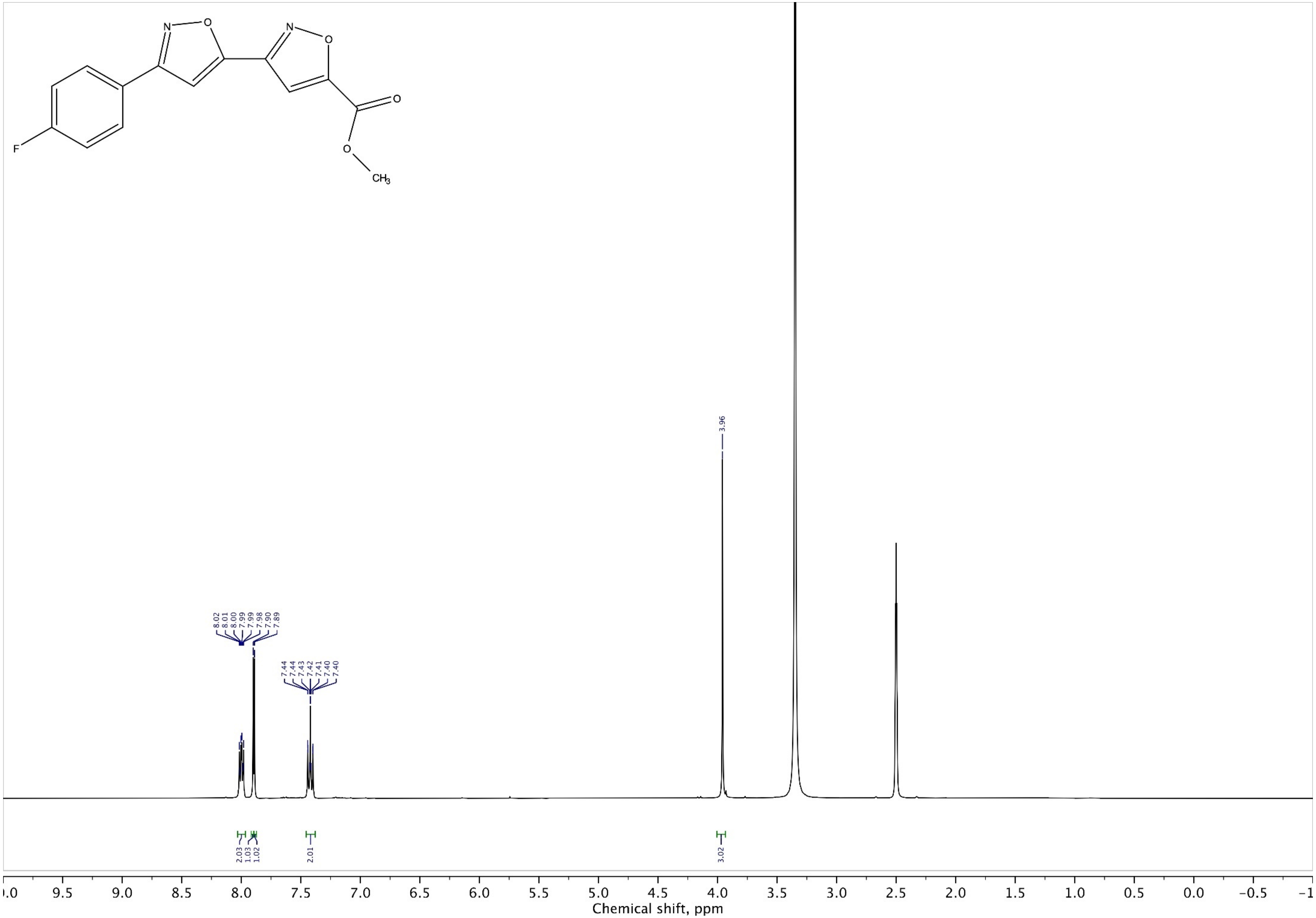

Methyl 3'-(4-fluorophenyl)-[3,5'-biisoxazole]-5-carboxylate (4p), $^{13}C\{^{1}H\}$ NMR, DMSO-d_6, 100 MHz

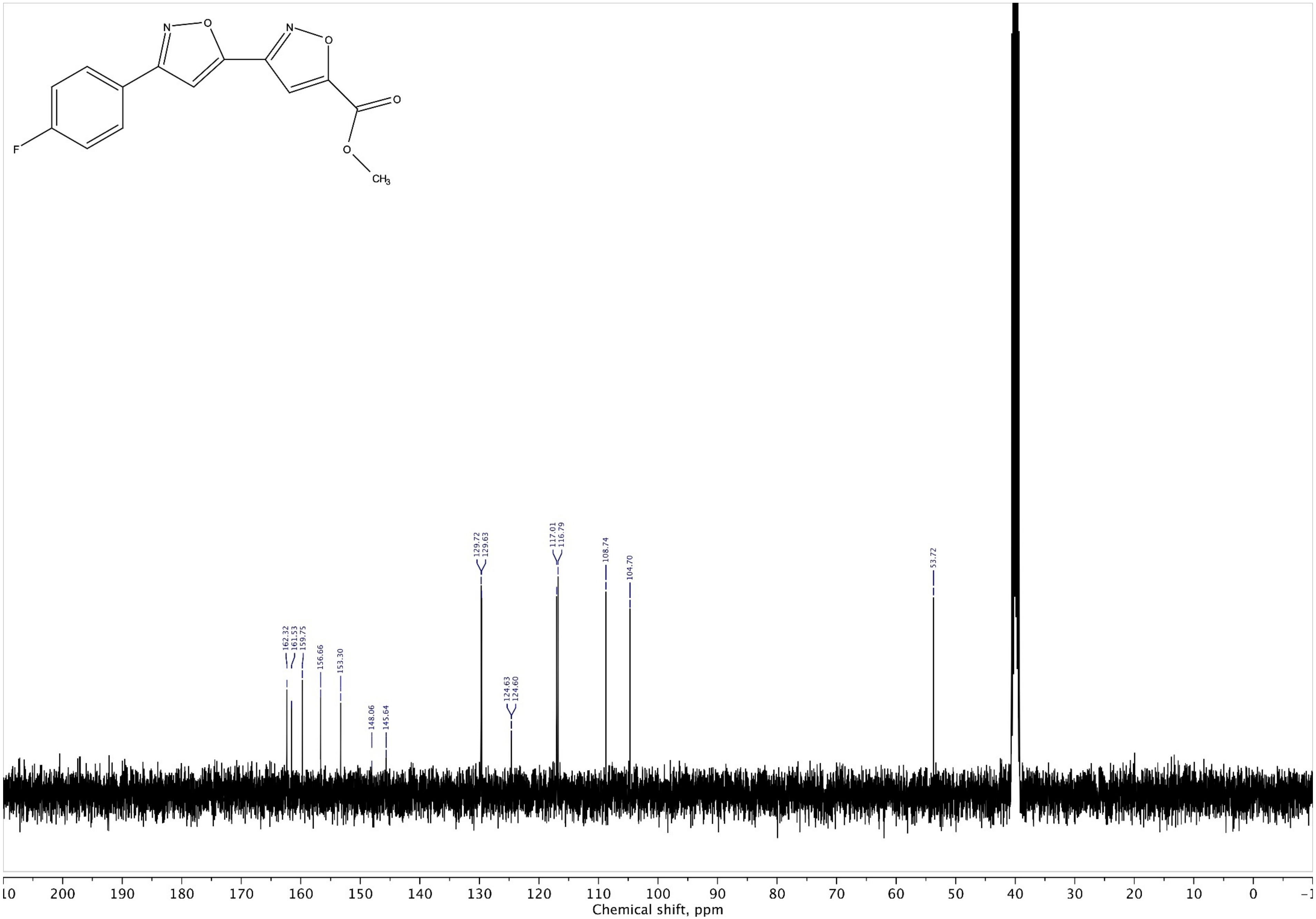

Methyl 3'-(4-fluorophenyl)-[3,5'-biisoxazole]-5-carboxylate (4p), DEPT, DMSO-d_6, 100 MHz

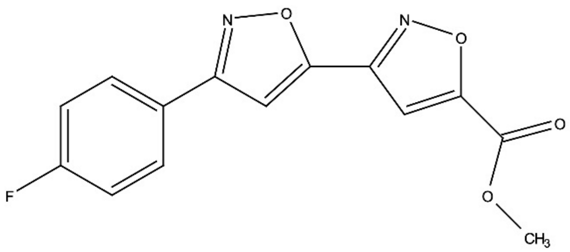

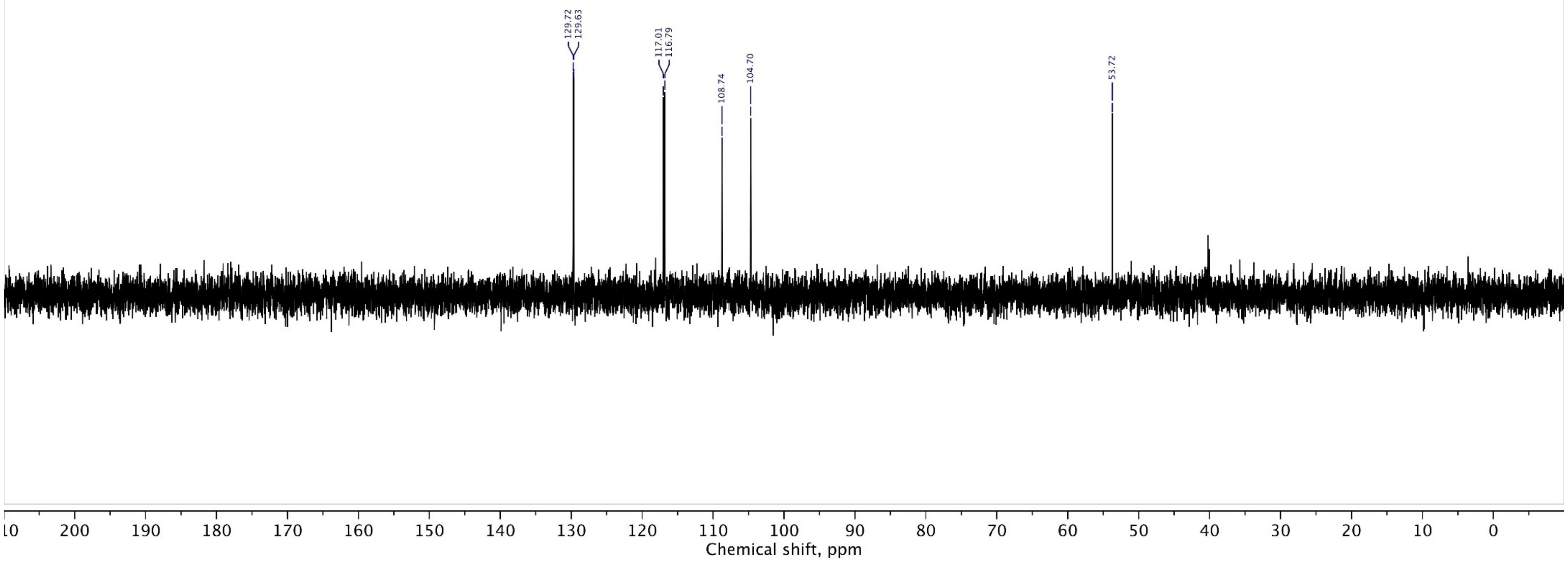

(3'-(4-Chlorophenyl)-[3,5'-biisoxazol]-5-yl)methyl benzenesulfonate (4q), ^{1}H NMR, $CDCl_3$, 400 MHz

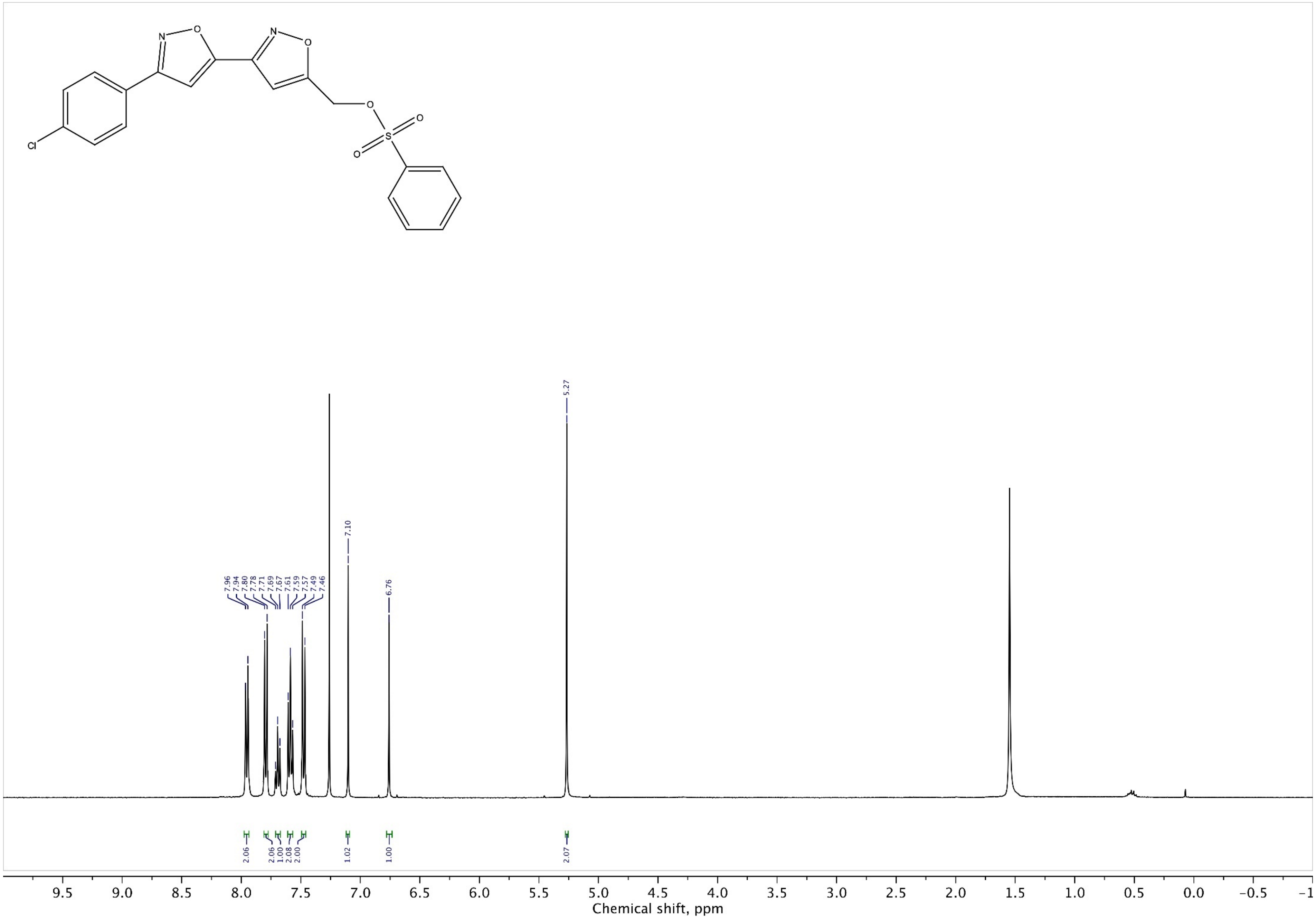

(3'-(4-Chlorophenyl)-[3,5'-biisoxazol]-5-yl)methyl benzenesulfonate (4q), $^{13}C\{^1H\}$ NMR, $CDCl_3$, 100 MHz

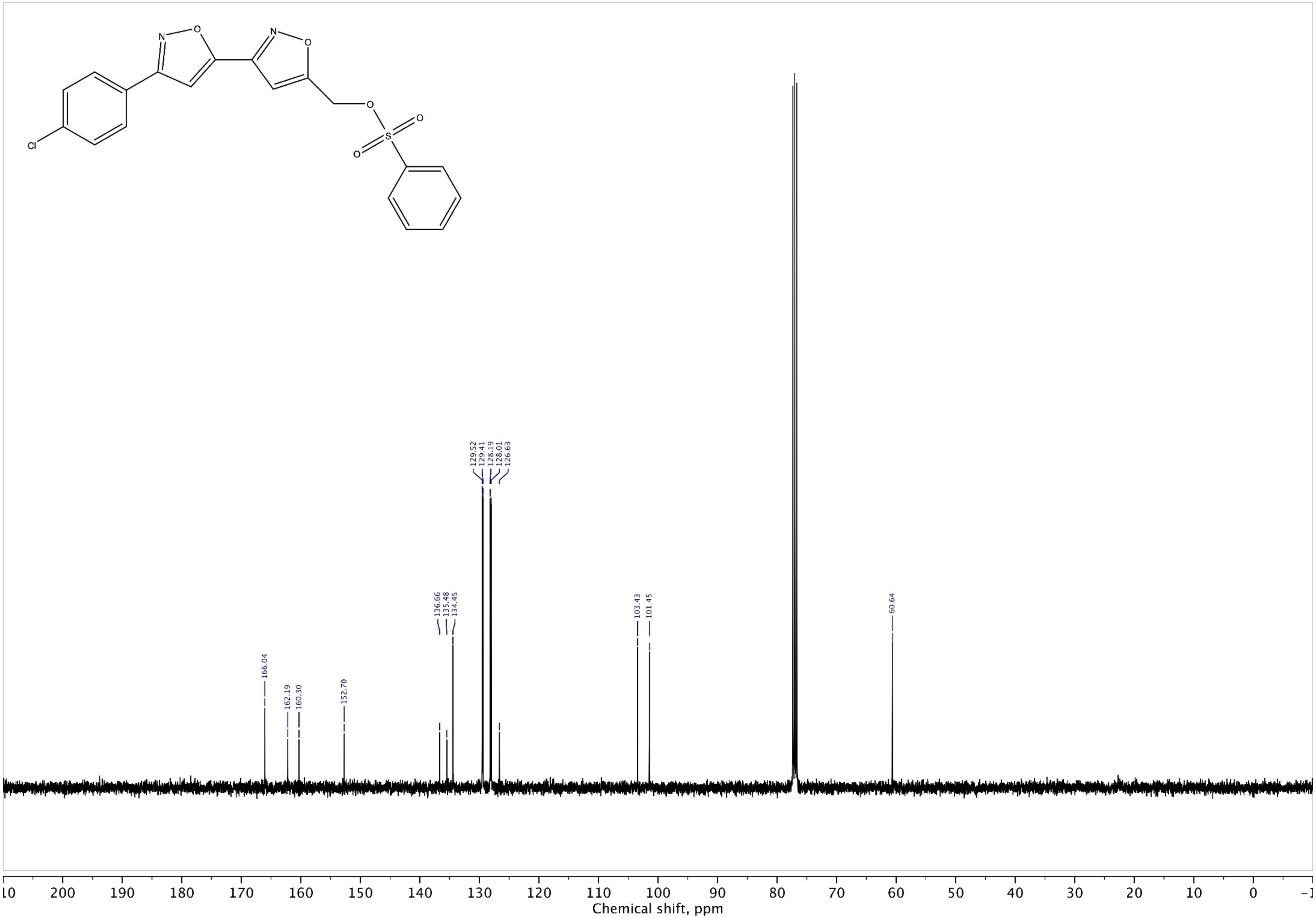

(3'-(4-Chlorophenyl)-[3,5'-biisoxazol]-5-yl)methyl benzenesulfonate (4q), DEPT, $CDCl_3$, 100 MHz

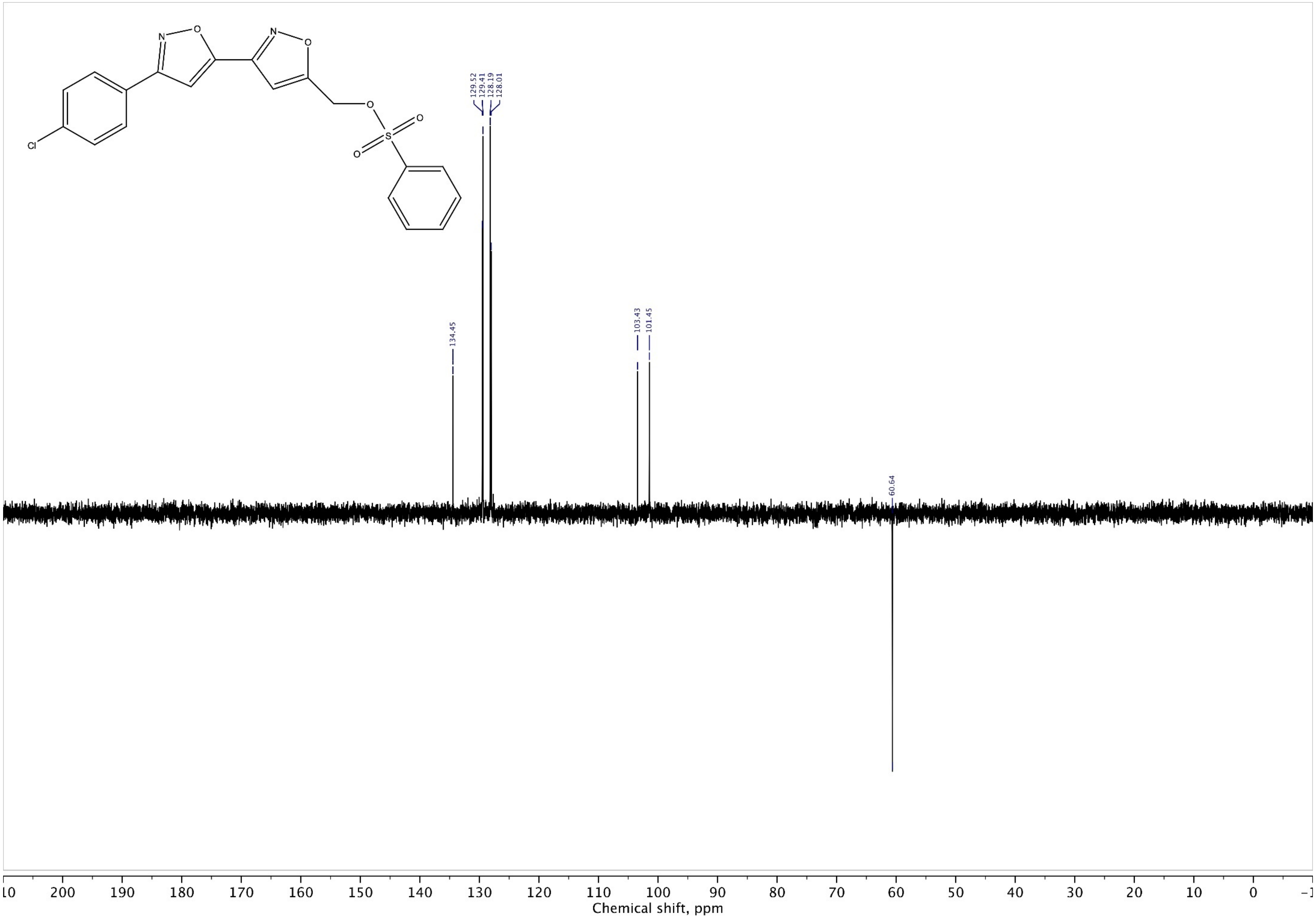

3'-(2-Bromophenyl)-5-(chloromethyl)-3,5'-biisoxazole (4r), ^{1}H NMR, $CDCl_3$, 400 MHz

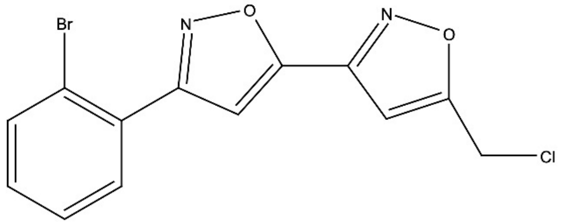

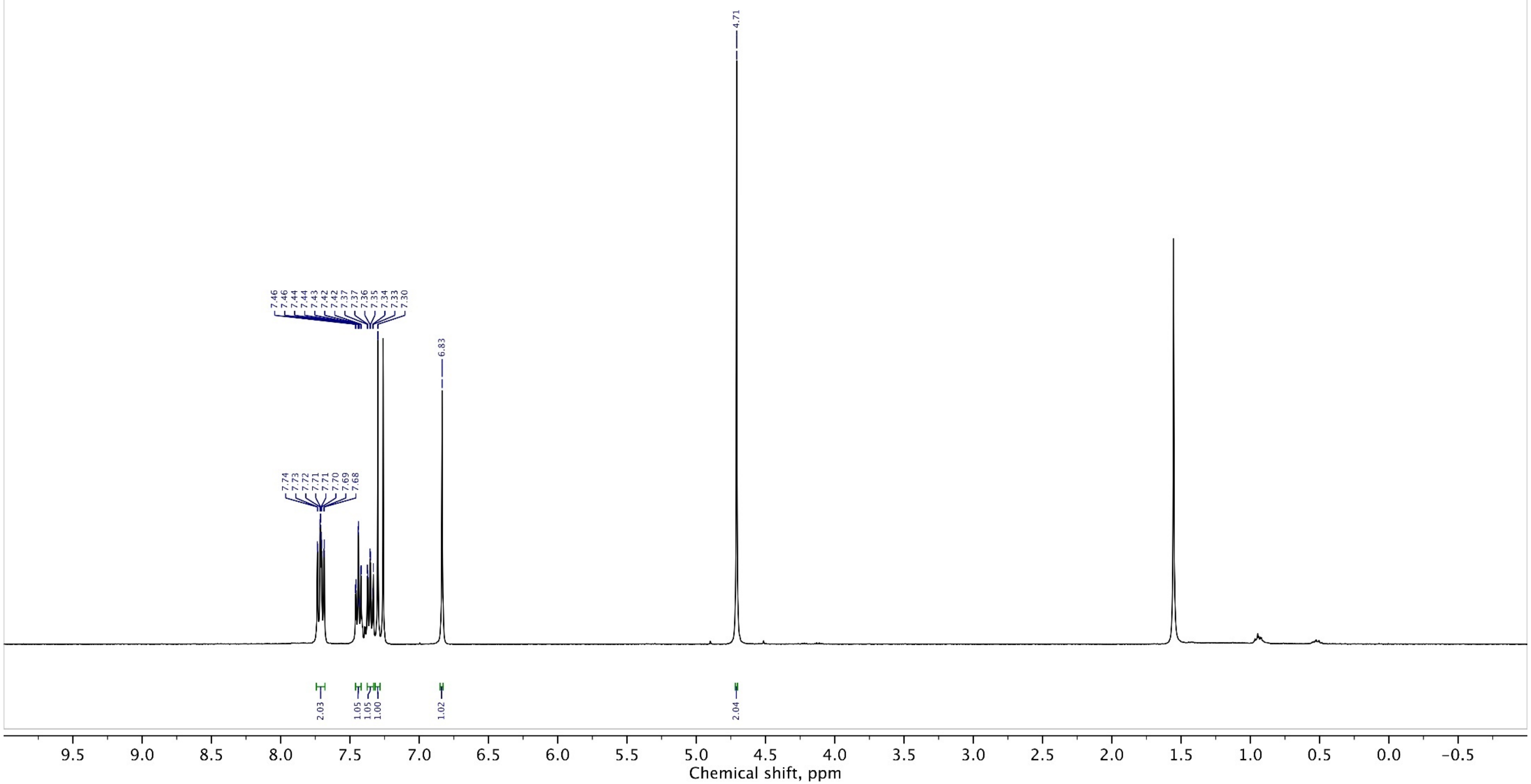

3'-(2-Bromophenyl)-5-(chloromethyl)-3,5'-biisoxazole (4r), $^{13}C\{^{1}H\}$ NMR, $CDCl_3$, 100 MHz

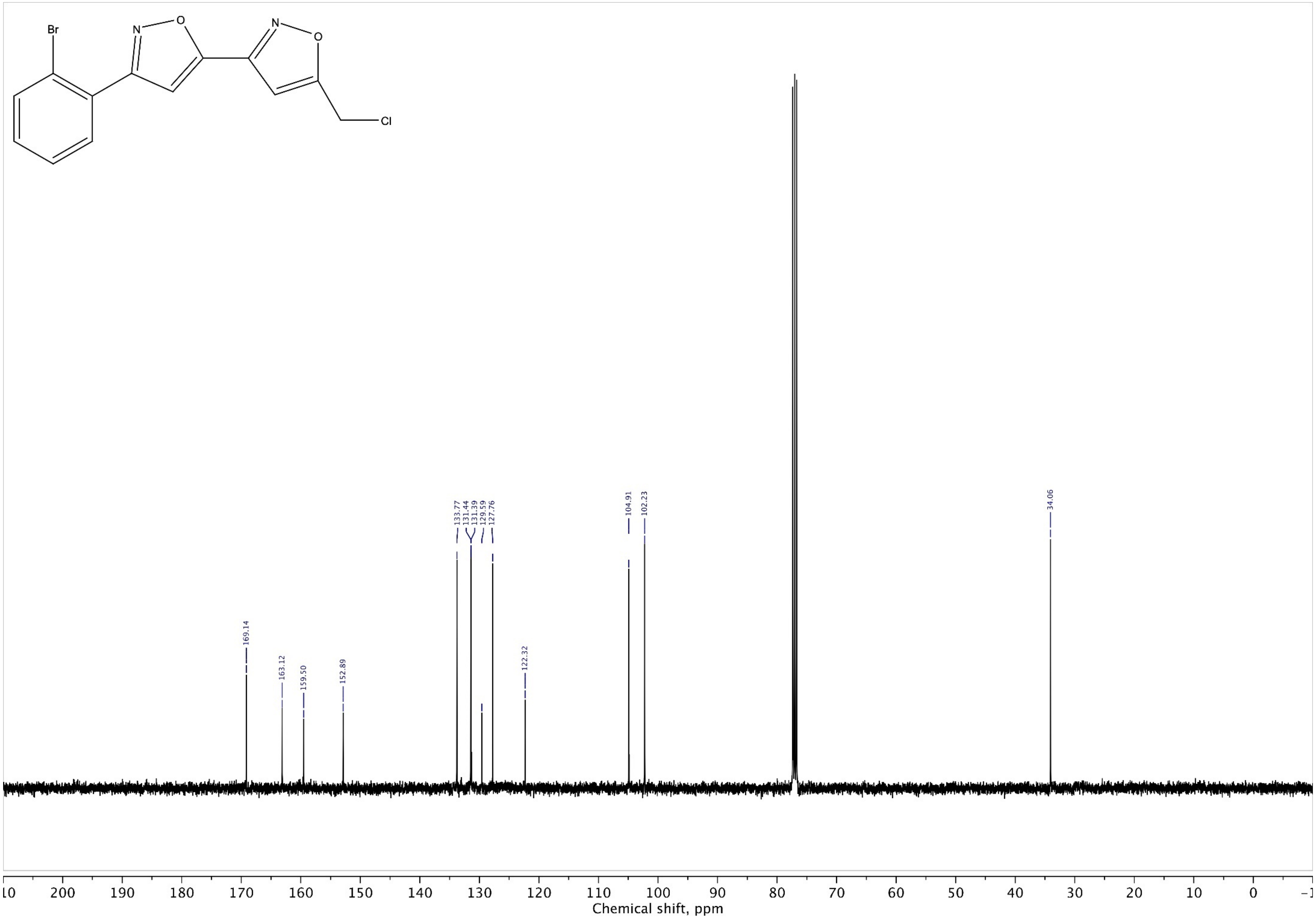

3'-(2-Bromophenyl)-5-(chloromethyl)-3,5'-biisoxazole (4r), DEPT, $CDCl_3$, 100 MHz

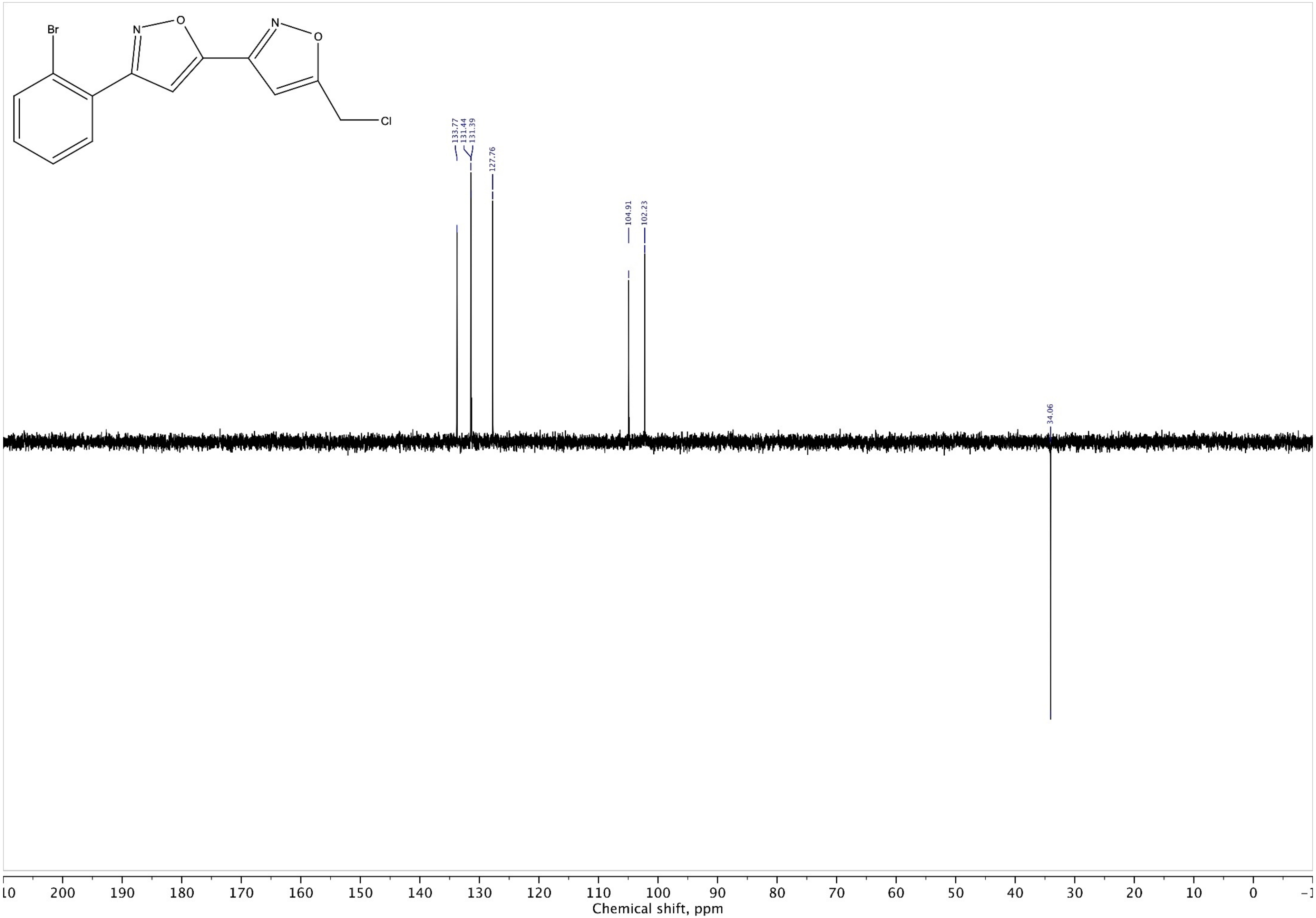

(3'-(Thiophen-2-yl)-[3,5'-biisoxazol]-5-yl)methanol (4s), ^{1}H NMR, $CDCl_3$, 400 MHz

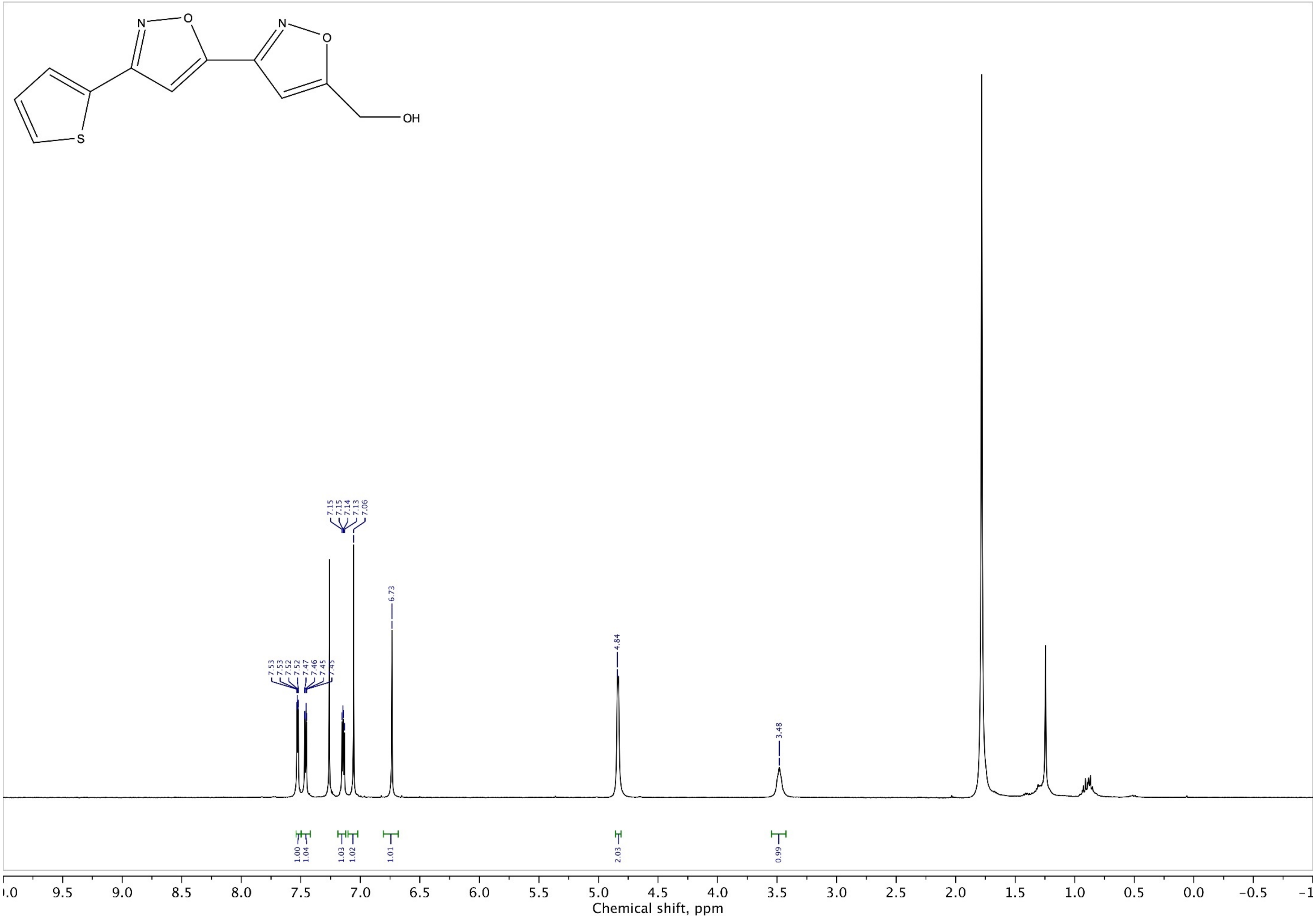

(3'-(Thiophen-2-yl)-[3,5'-biisoxazol]-5-yl)methanol (4s), $^{13}C\{^{1}H\}$ NMR, $CDCl_3$, 100 MHz

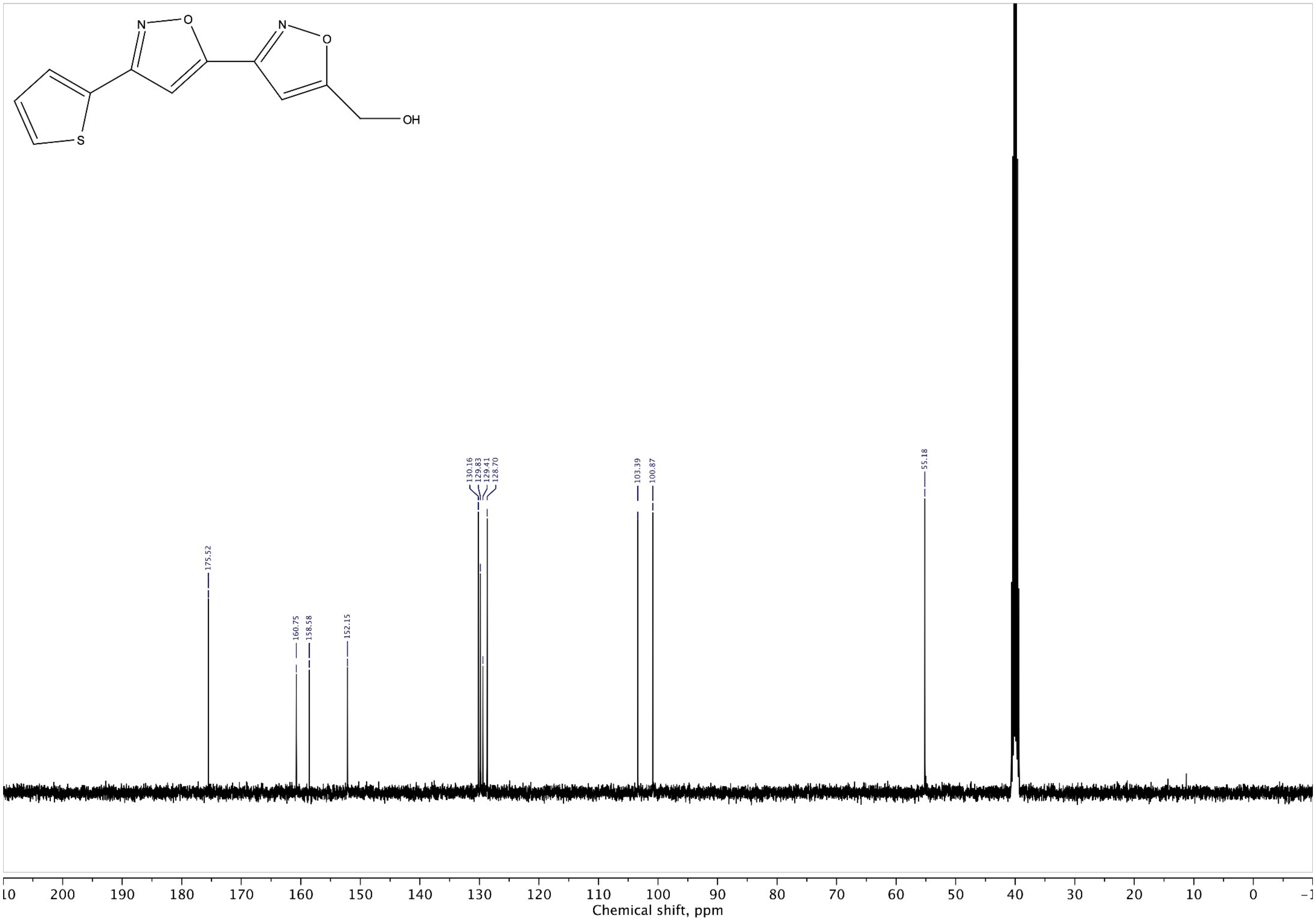

(3'-(Thiophen-2-yl)-[3,5'-biisoxazol]-5-yl)methanol (4s), DEPT, $CDCl_3$, 100 MHz

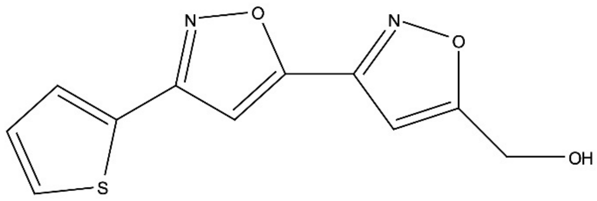

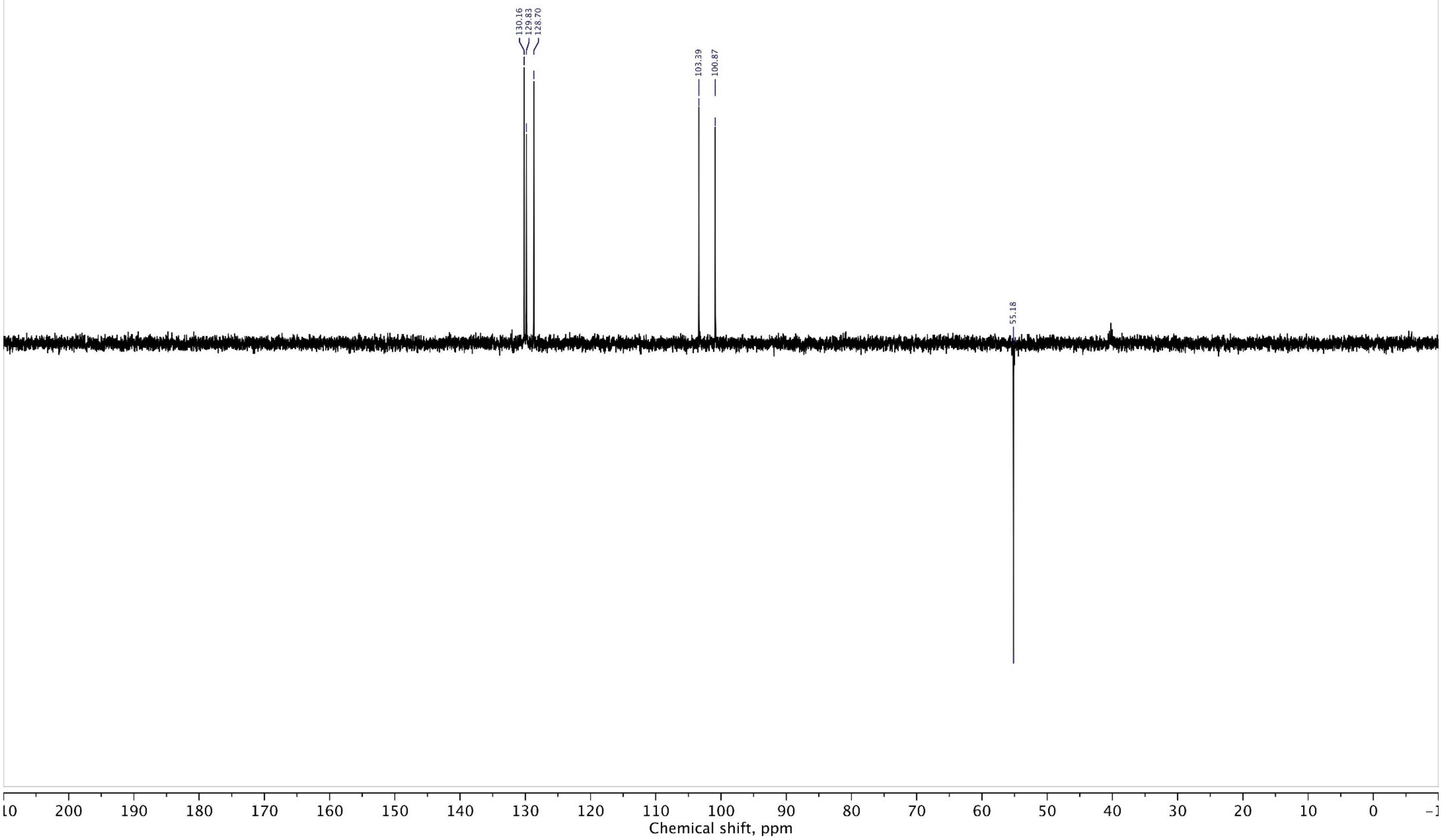

5-(Chloromethyl)-3'-(thiophen-2-yl)-3,5'-biisoxazole (4t), ^{1}H NMR, $CDCl_3$, 400 MHz

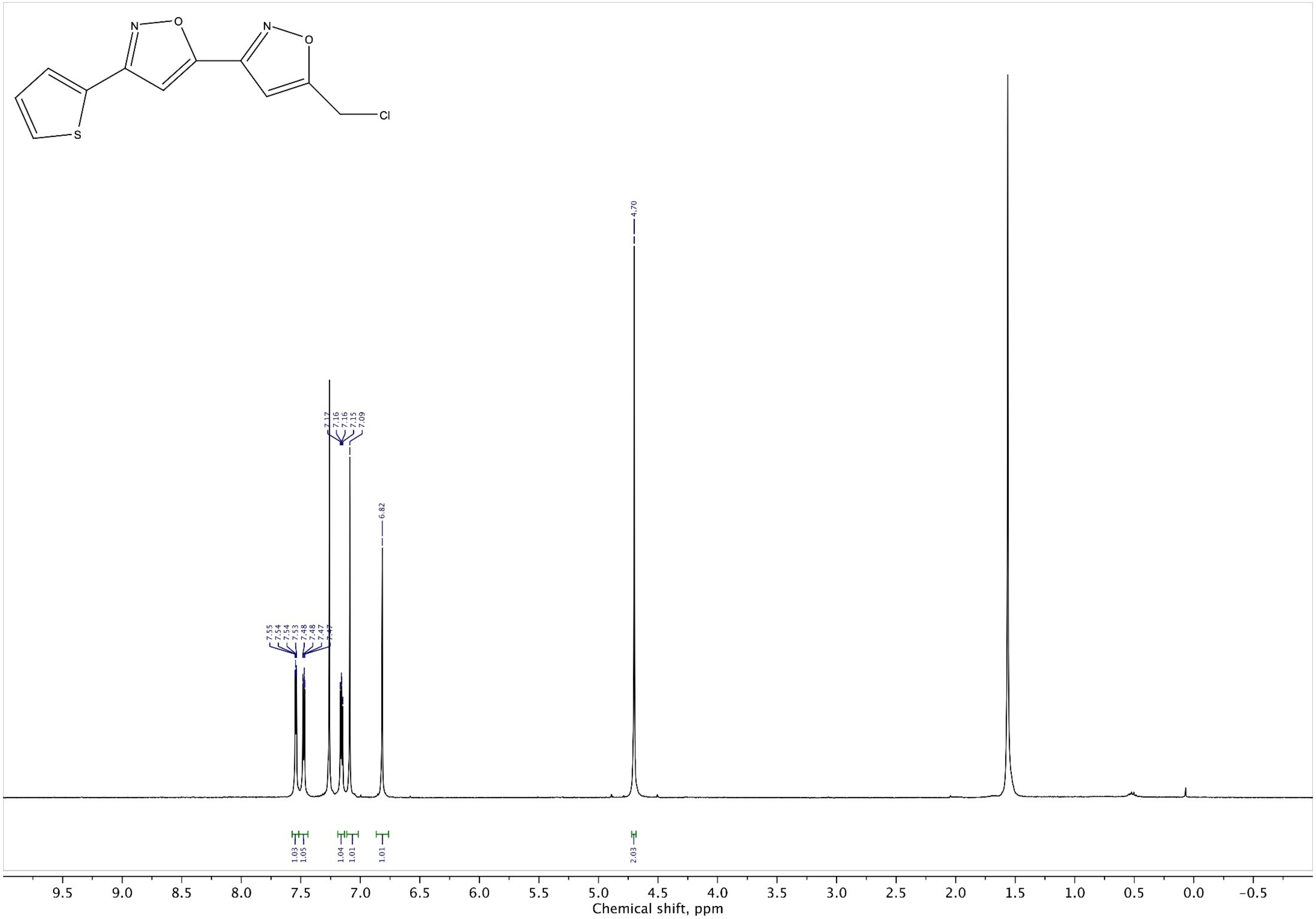

5-(Chloromethyl)-3'-(thiophen-2-yl)-3,5'-biisoxazole (4t), $^{13}C\{^1H\}$ NMR, $CDCl_3$, 100 MHz

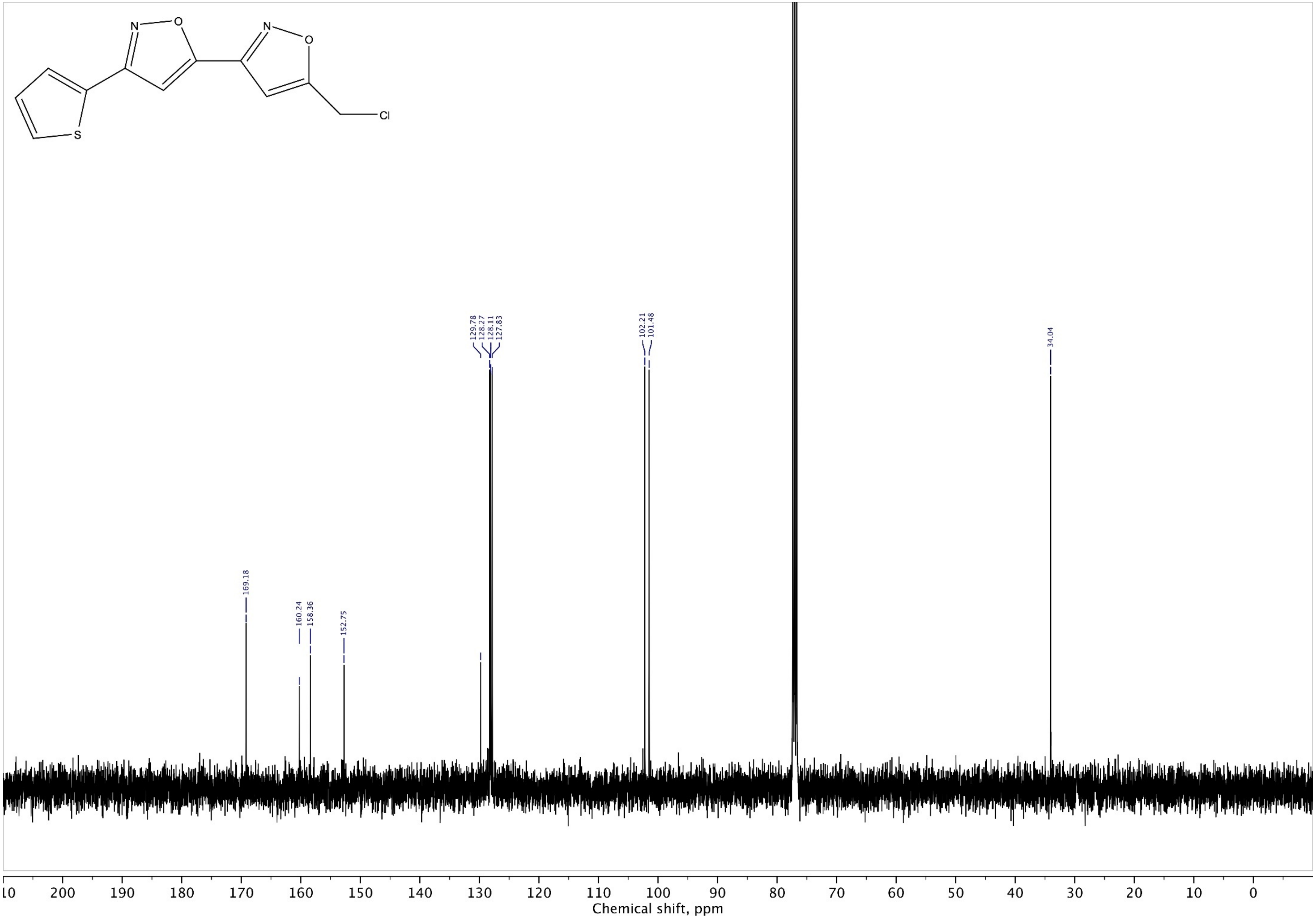

5-(Chloromethyl)-3'-(thiophen-2-yl)-3,5'-biisoxazole (4t), DEPT, $CDCl_3$, 100 MHz

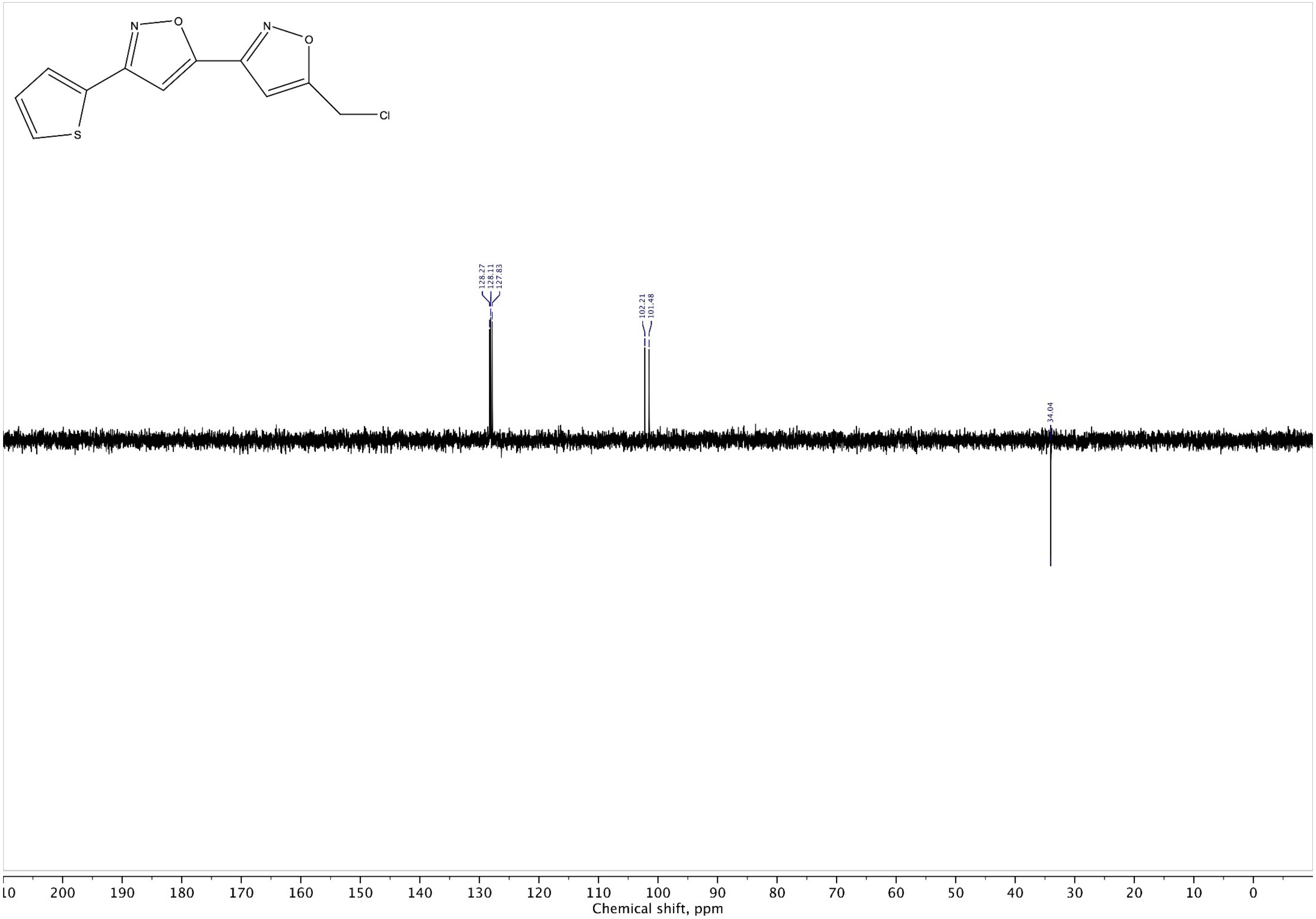

Methyl 3'-(*tert*-butyl)-[3,5'-biisoxazole]-5-carboxylate (4u), ^{1}H NMR, $CDCl_3$, 400 MHz

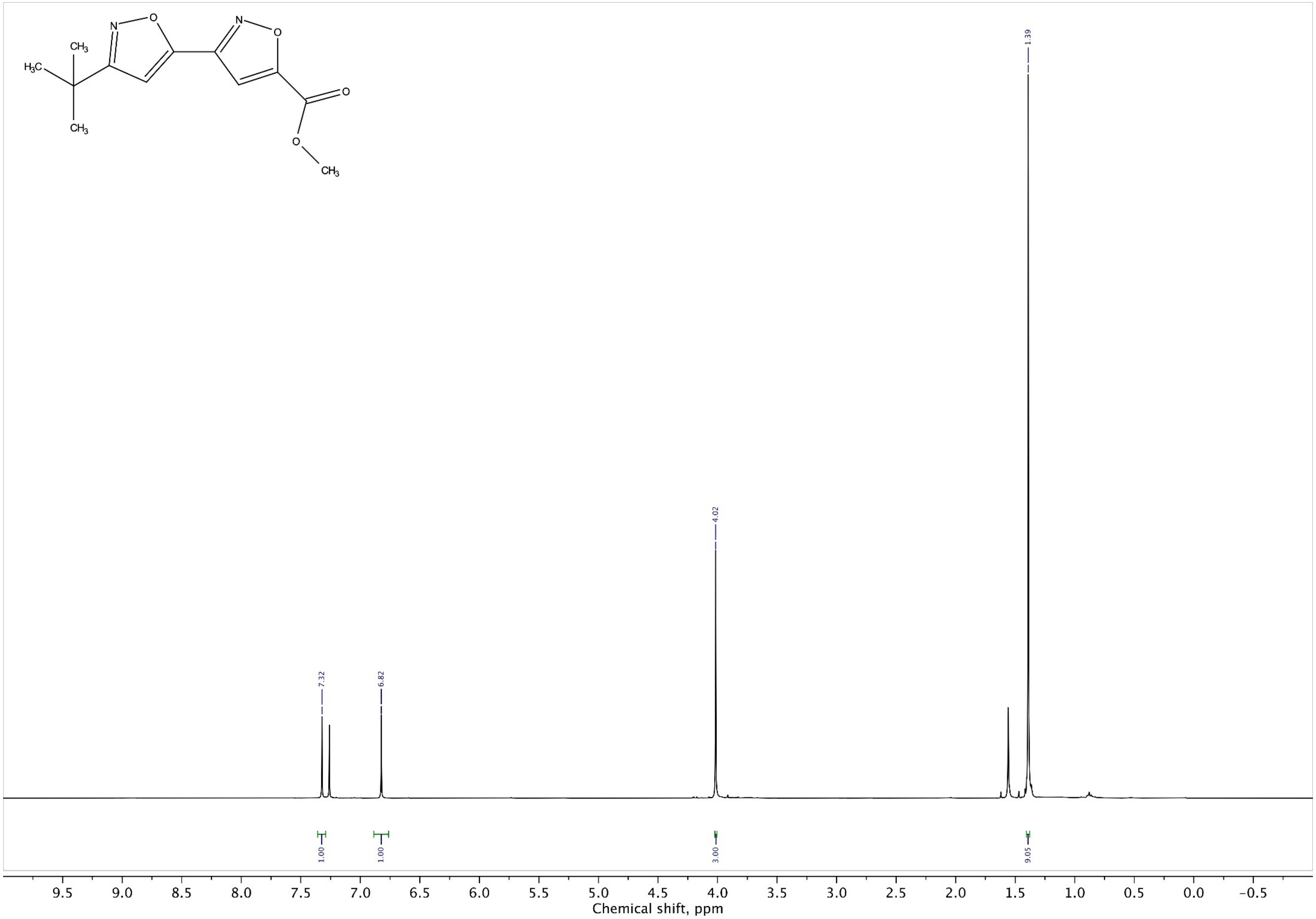

Methyl 3'-(*tert*-butyl)-[3,5'-biisoxazole]-5-carboxylate (4u), $^{13}C\{^{1}H\}$, $CDCl_3$, 100 MHz

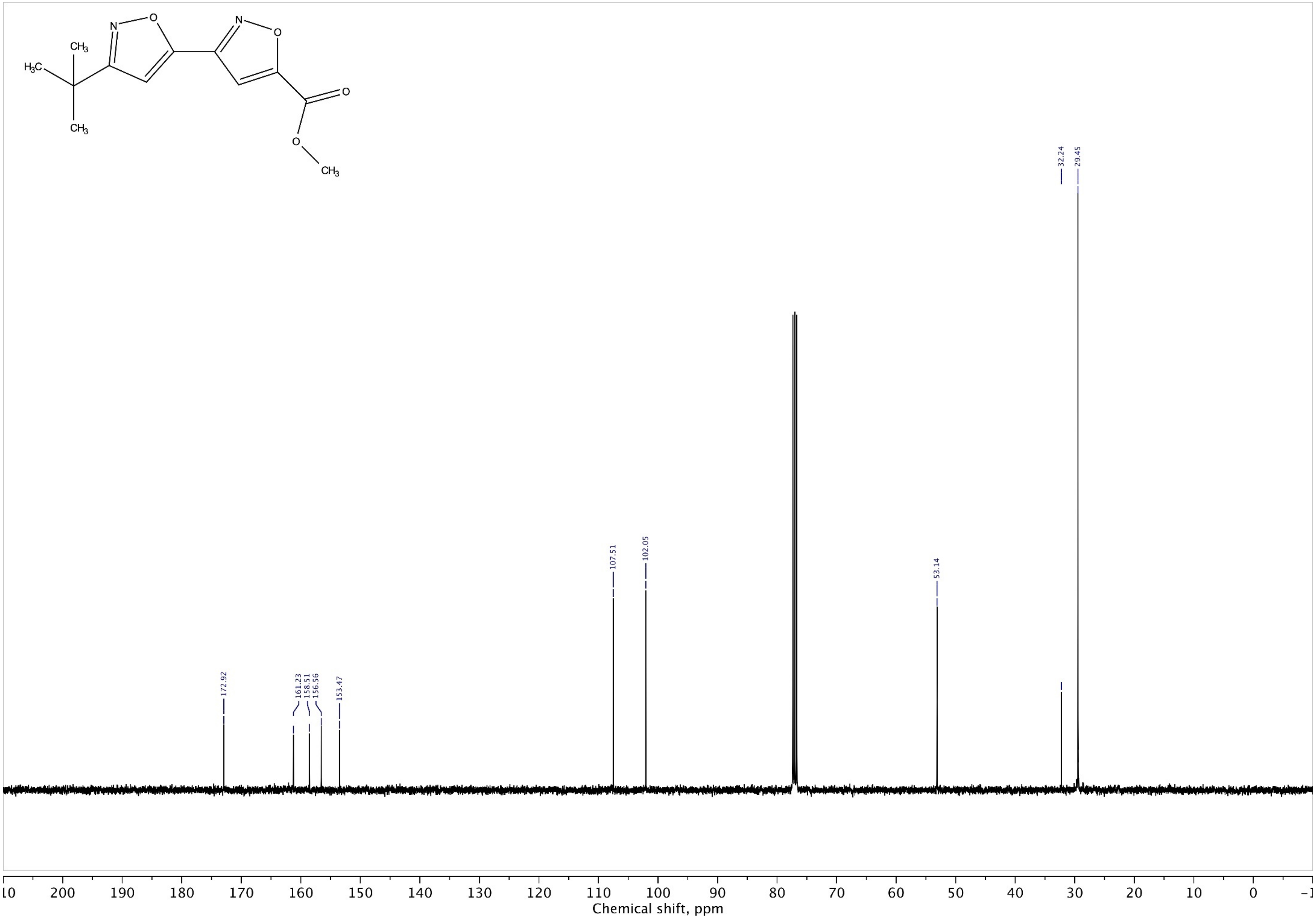

Methyl 3'-(*tert*-butyl)-[3,5'-biisoxazole]-5-carboxylate (4u), DEPT, $CDCl_3$, 100 MHz

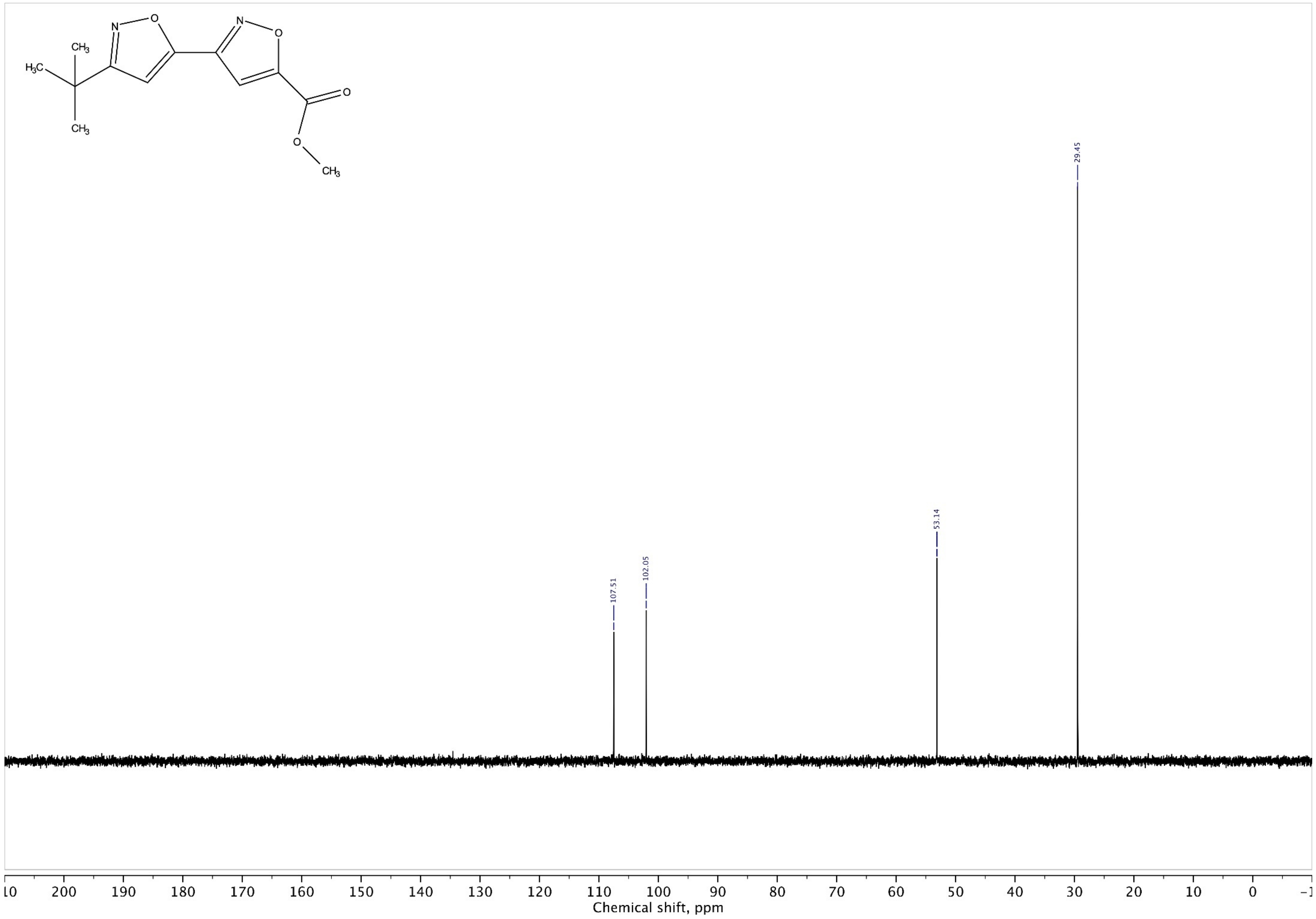

Dimethyl 3'-phenyl-[3,5'-biisoxazole]-4,5-dicarboxylate (4w), ^{1}H NMR, $CDCl_3$, 400 MHz

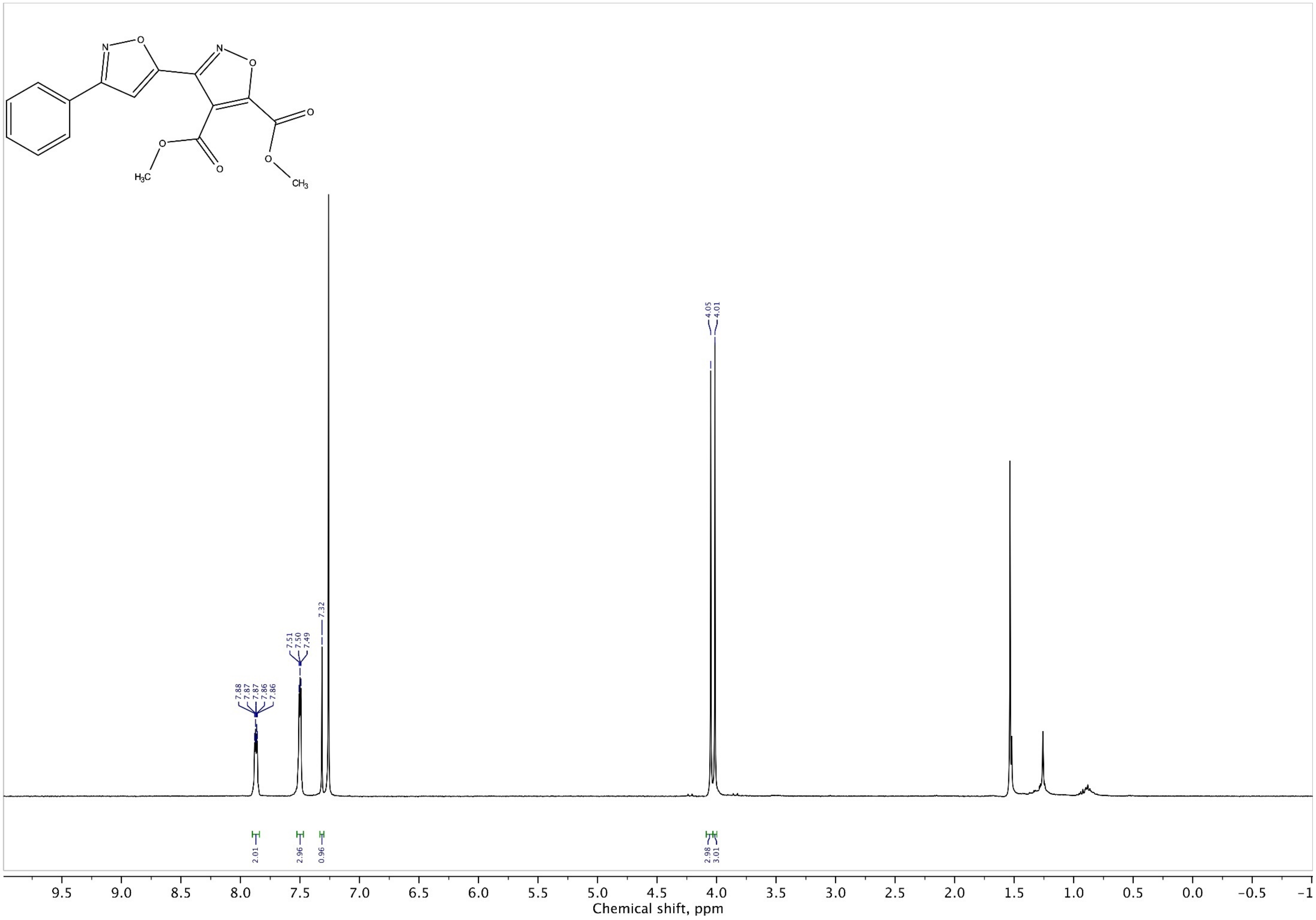

Dimethyl 3'-phenyl-[3,5'-biisoxazole]-4,5-dicarboxylate (4w), $^{13}C\{^{1}H\}$ NMR, $CDCl_3$, 100 MHz

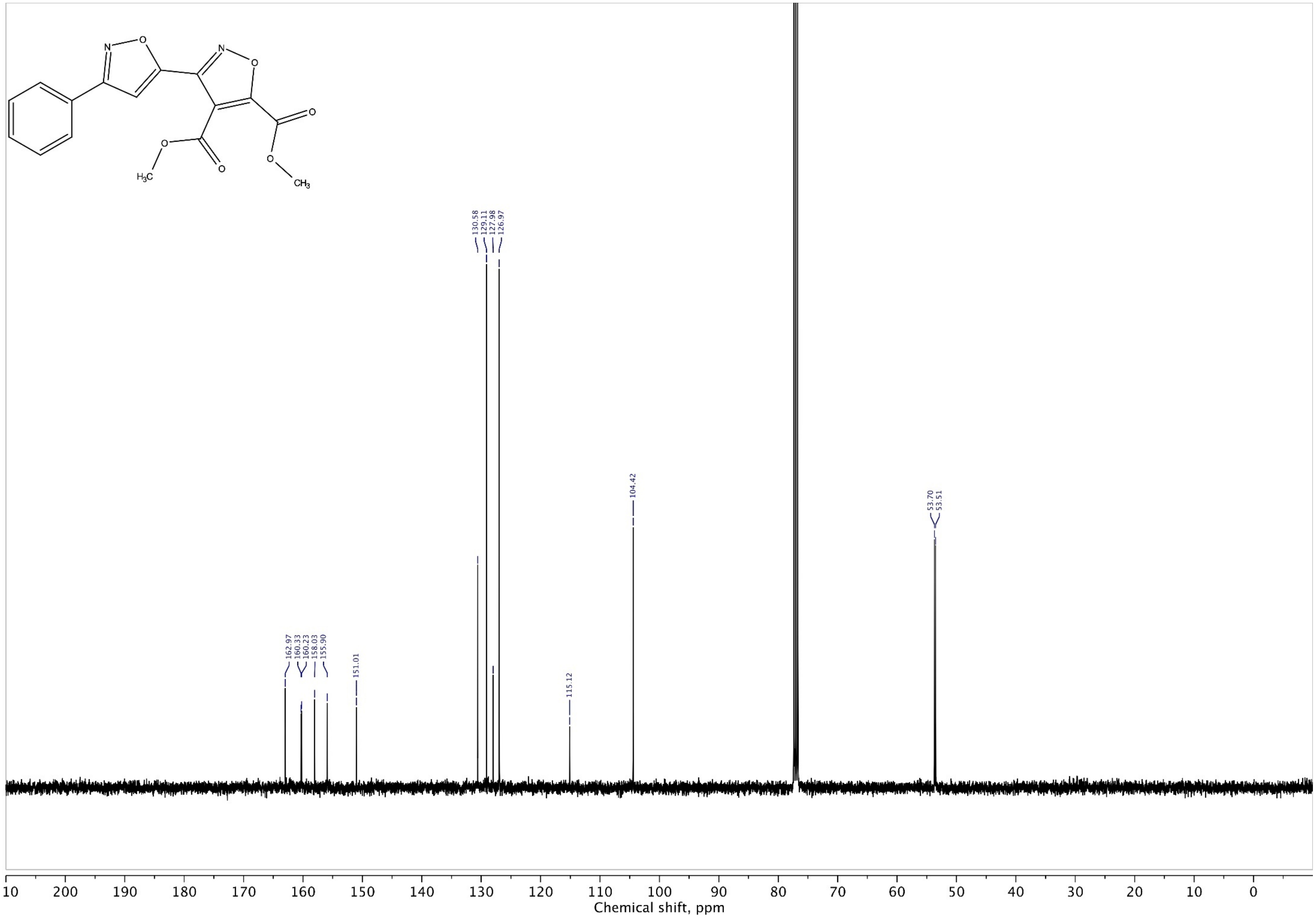

Dimethyl 3'-phenyl-[3,5'-biisoxazole]-4,5-dicarboxylate (4w), DEPT, $CDCl_3$, 100 MHz

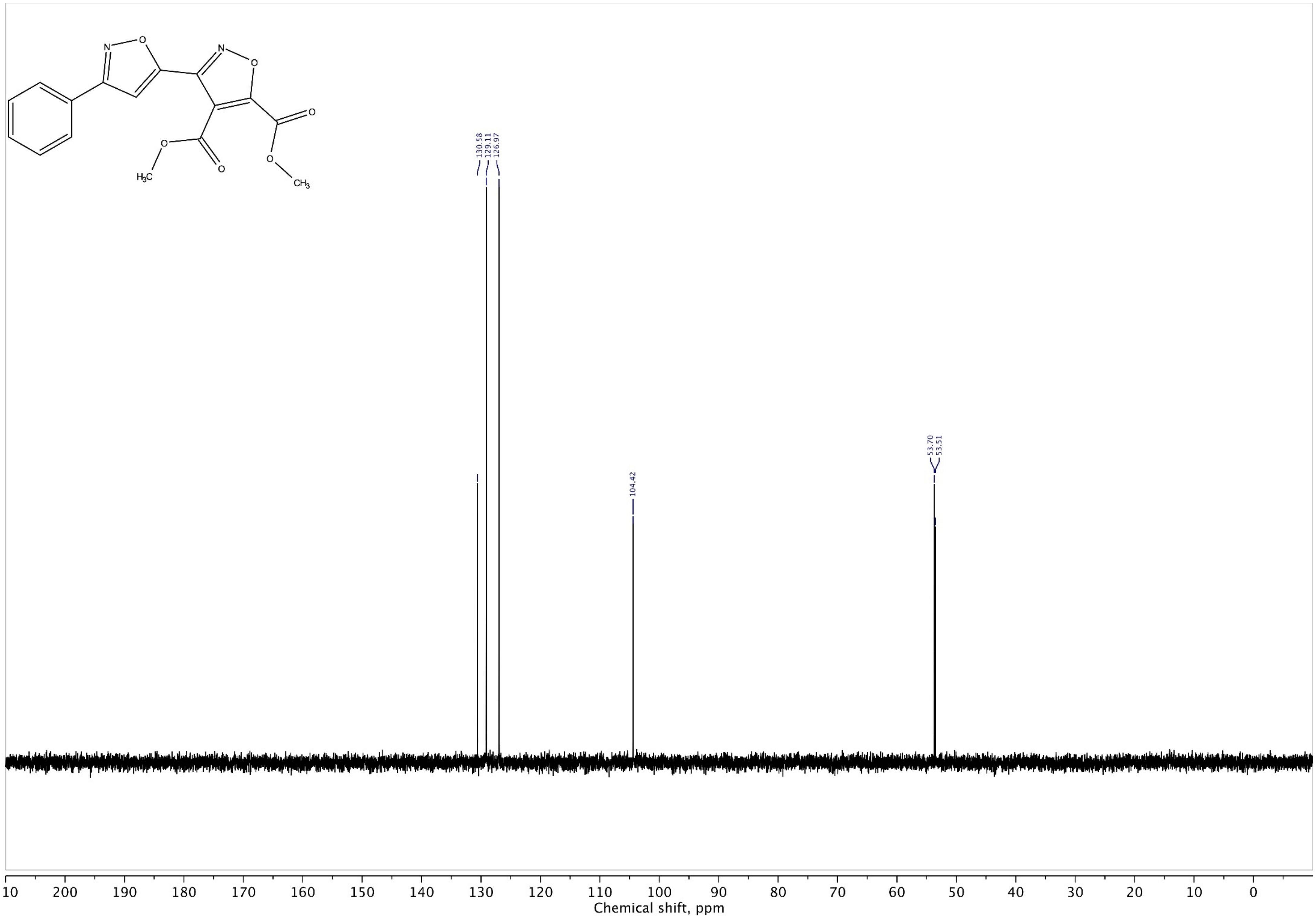

3'-(4-Chlorophenyl)-5-phenyl-3,5'-biisoxazole (4x), ^{1}H NMR, DMSO-d_6, 400 MHz

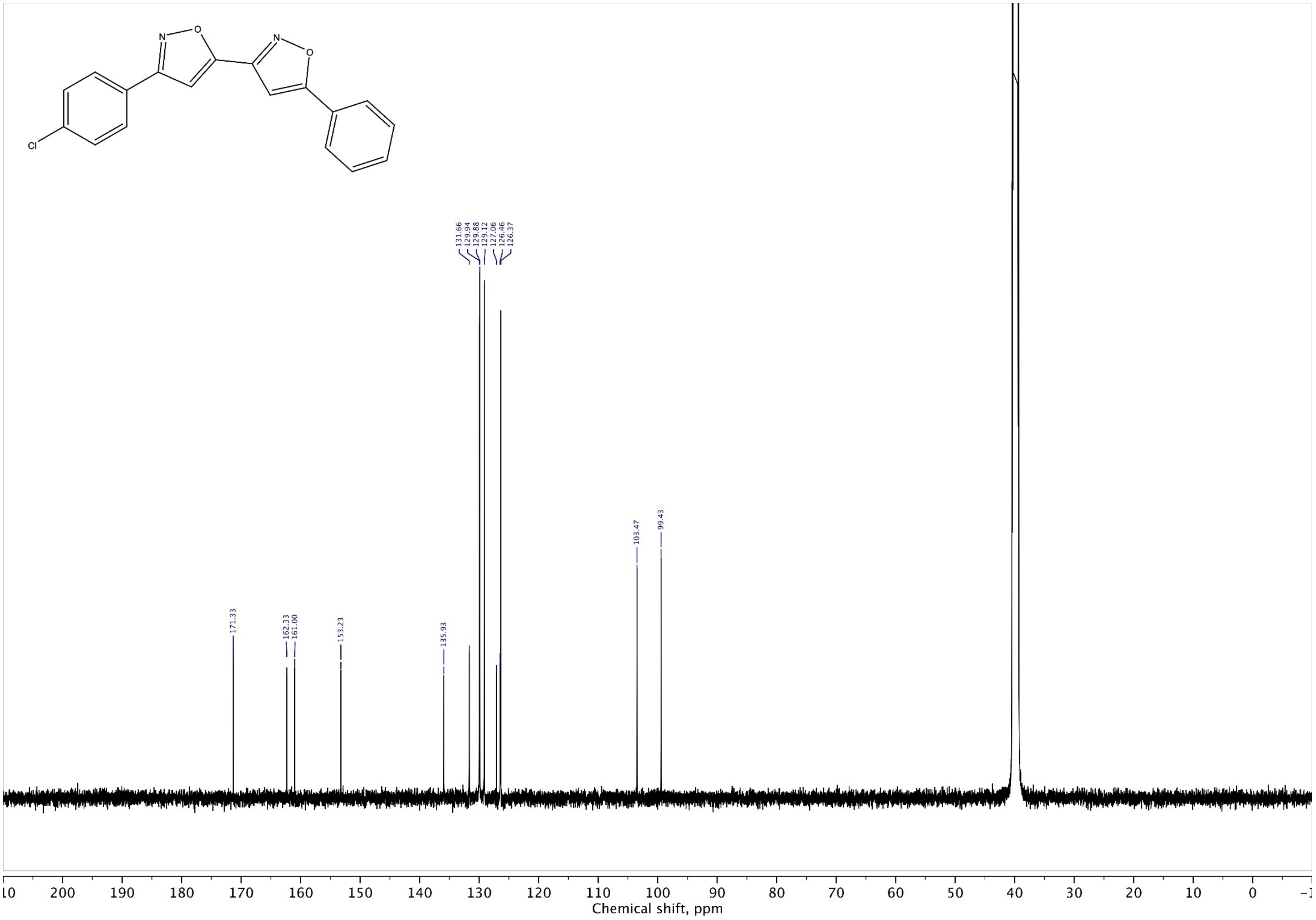

3'-(4-Chlorophenyl)-5-phenyl-3,5'-biisoxazole (4x), $^{13}C\{^{1}H\}$ NMR, DMSO-d_6, 100 MHz

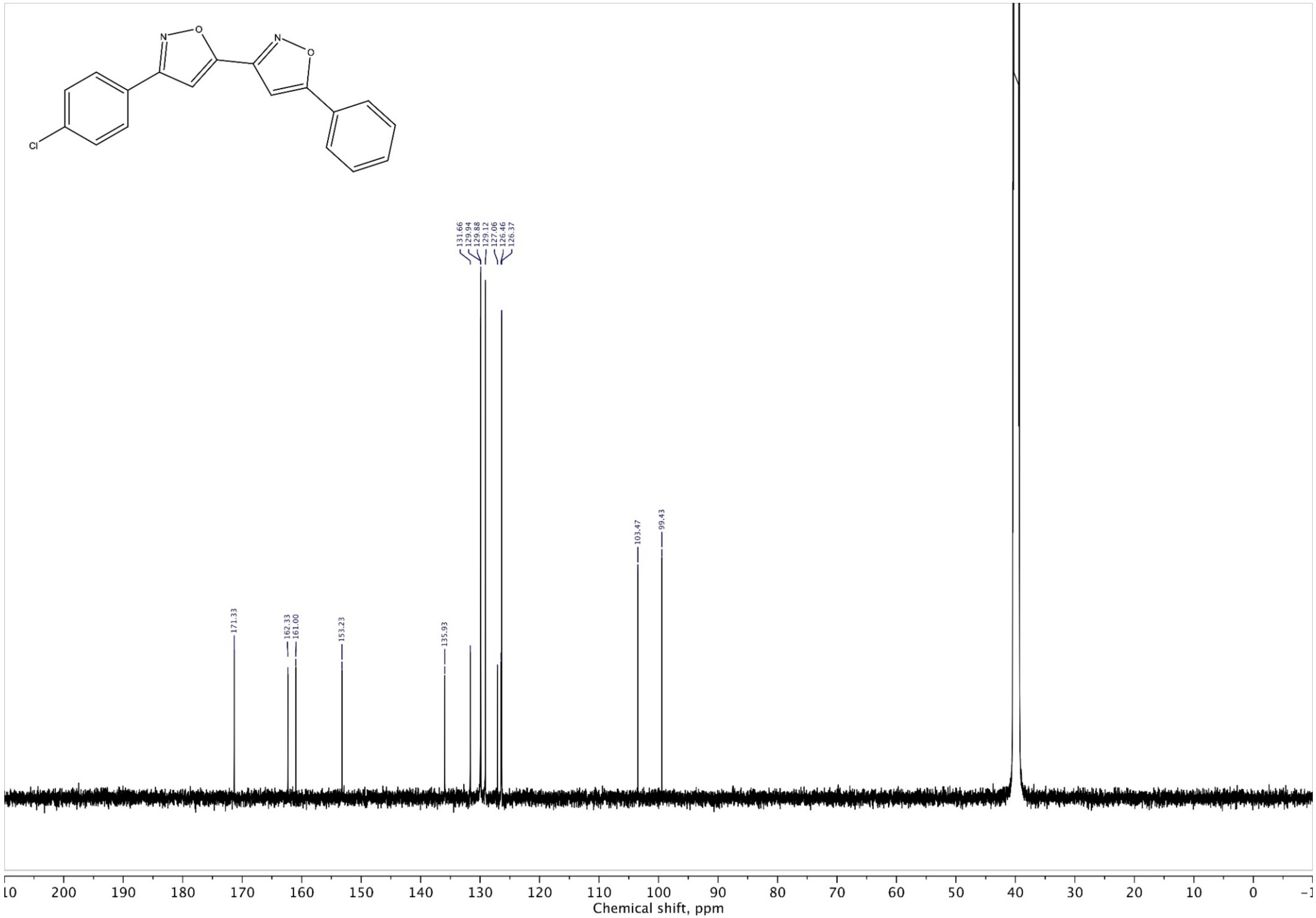

3'-(4-Chlorophenyl)-5-phenyl-3,5'-biisoxazole (4x), DEPT, DMSO-d_6, 100 MHz

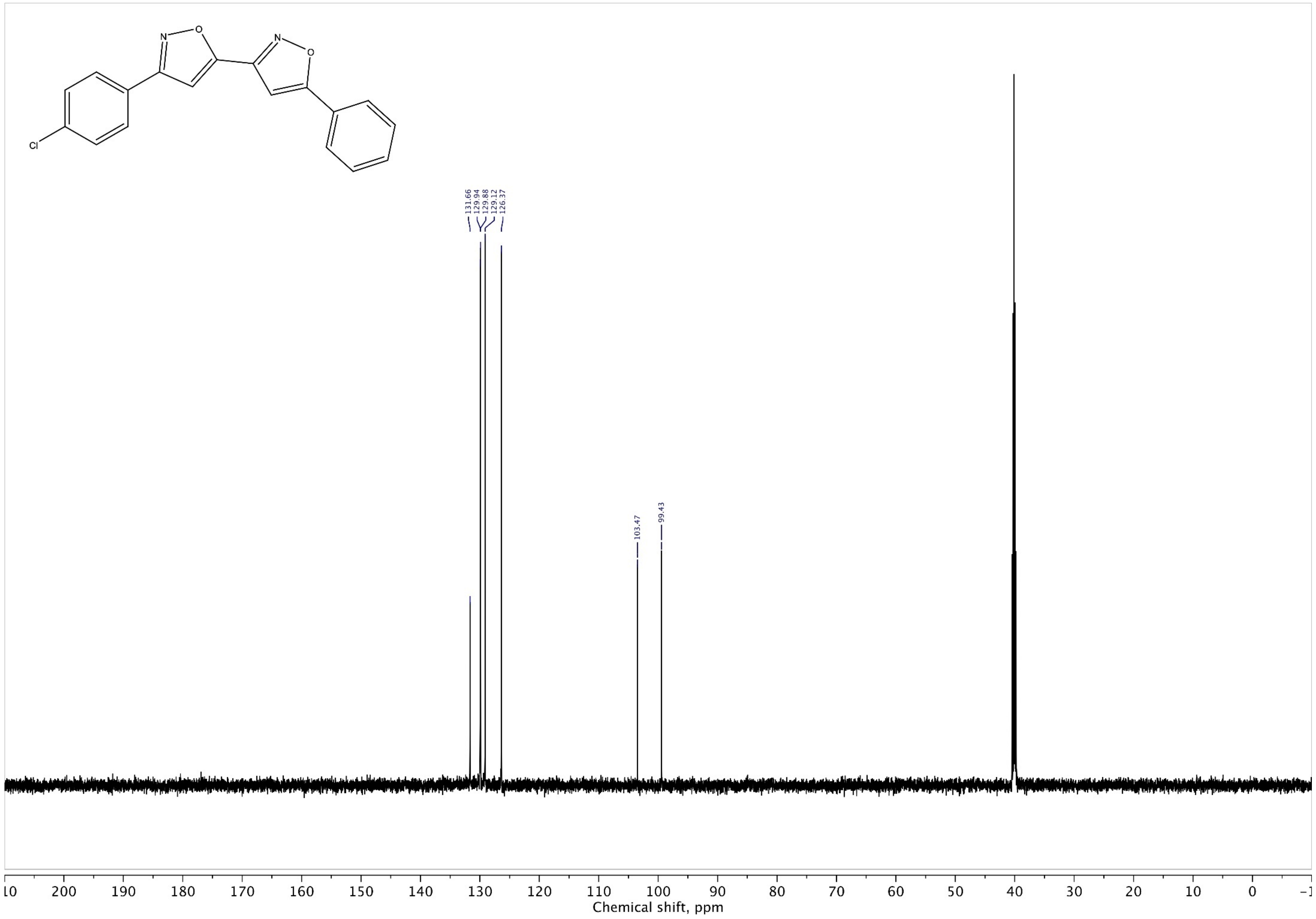

3'-Phenyl-5-(thiophen-2-yl)-3,5'-biisoxazole (4y), ^{1}H NMR, $CDCl_3$, 400 MHz

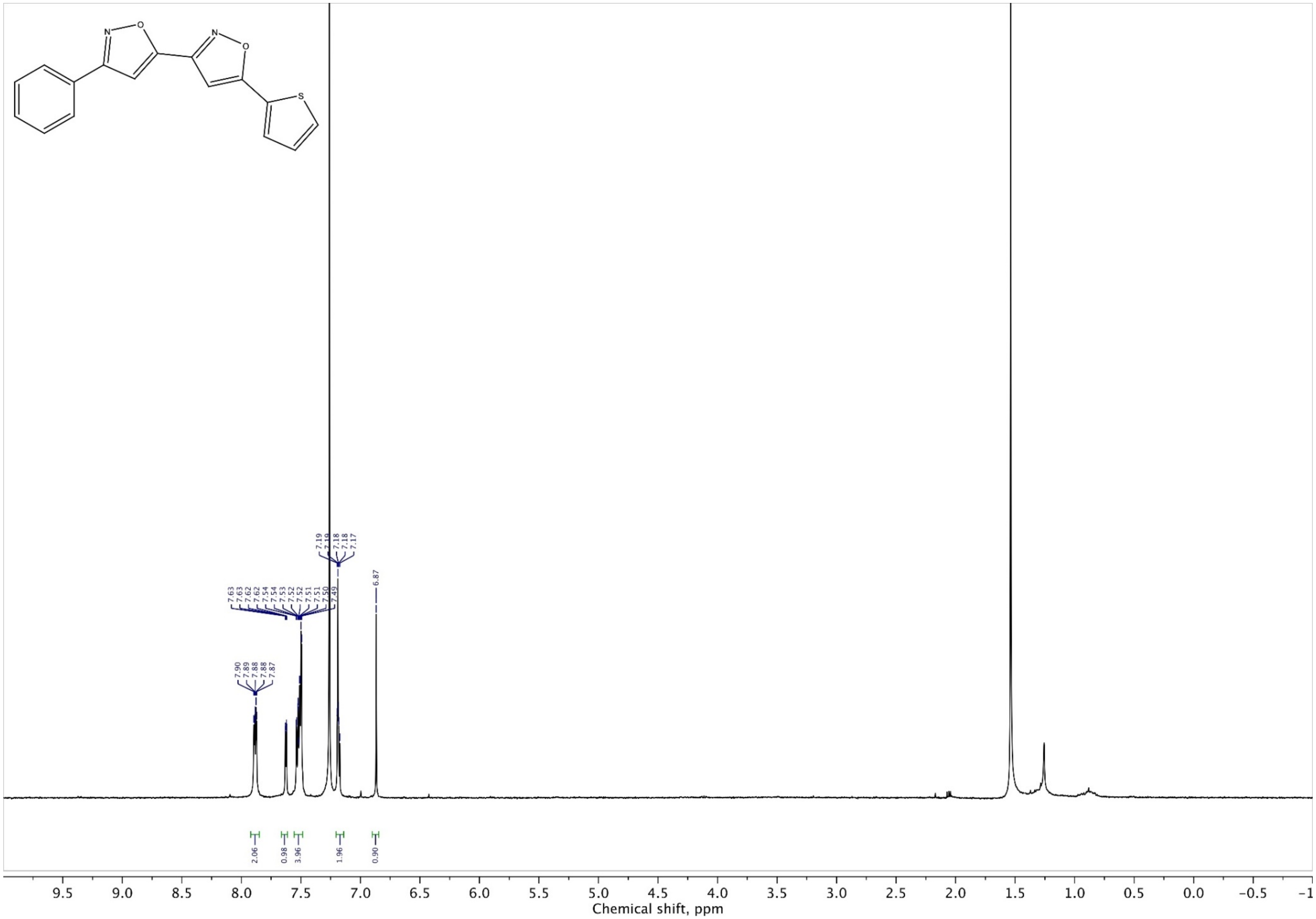

3'-Phenyl-5-(thiophen-2-yl)-3,5'-biisoxazole (4y), $^{13}C\{^{1}H\}$ NMR, $CDCl_3$, 100 MHz

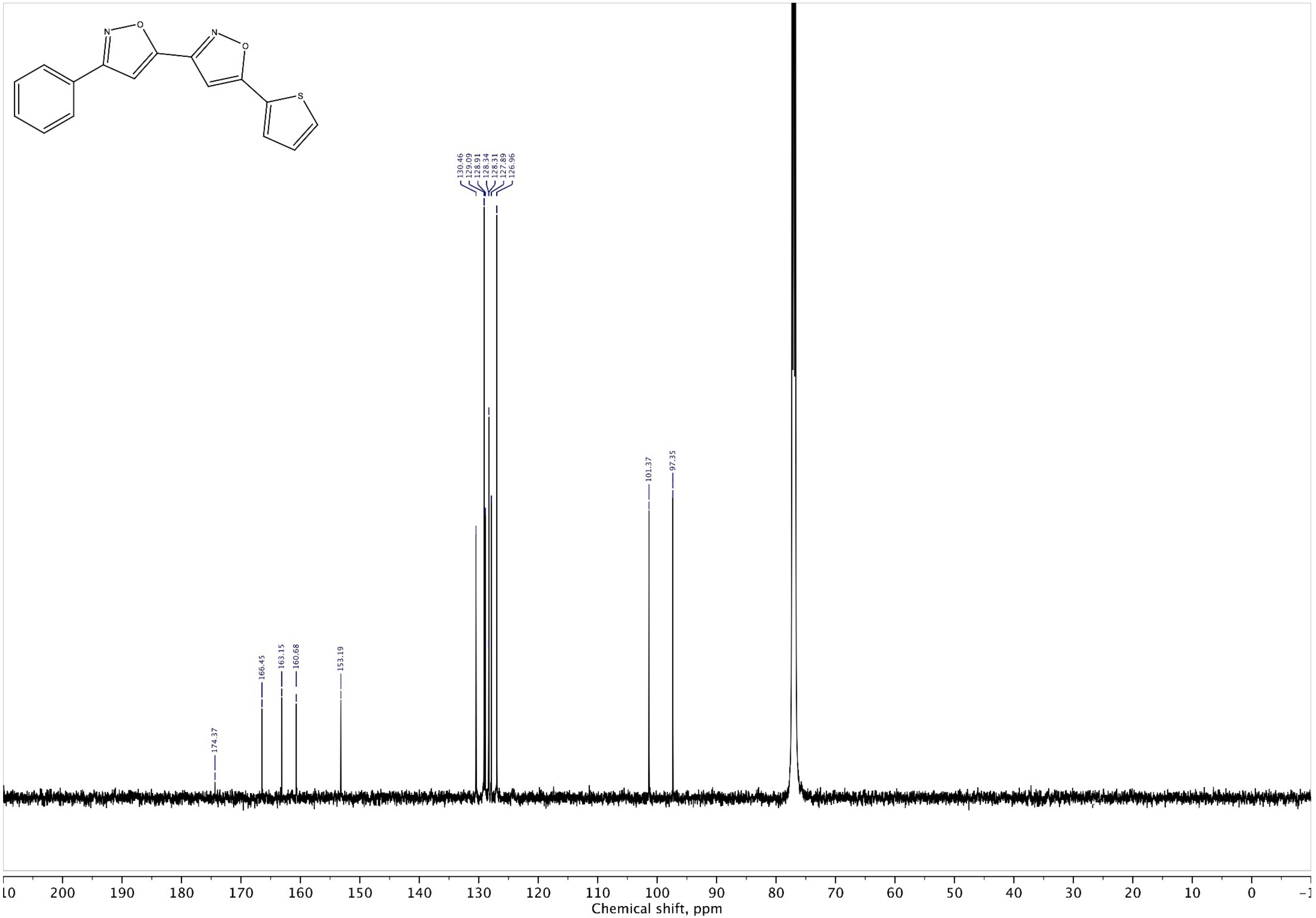

3'-Phenyl-5-(thiophen-2-yl)-3,5'-biisoxazole (4y), DEPT, $CDCl_3$, 100 MHz

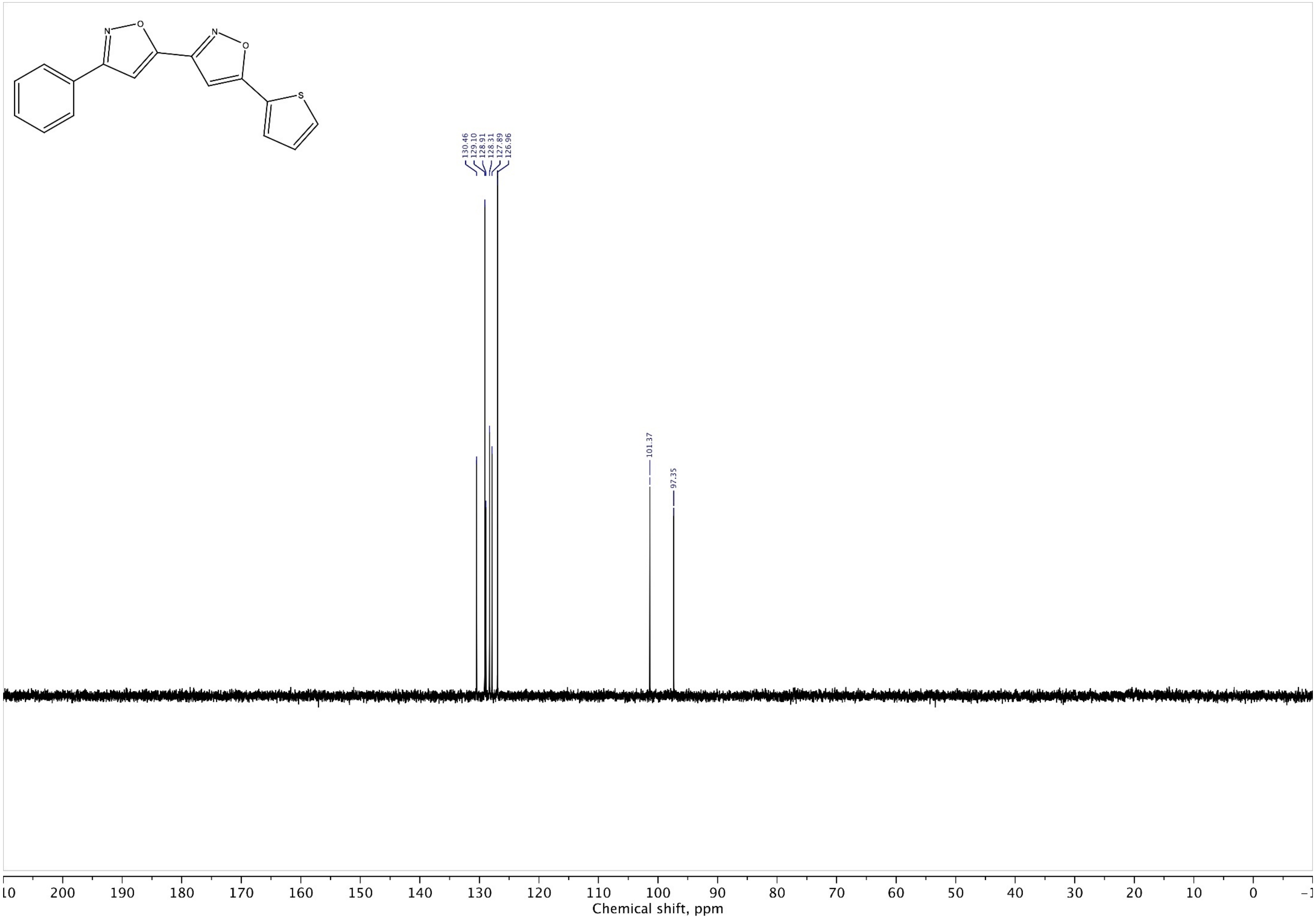

3,4-bis(3-(4-Chlorophenyl)isoxazol-5-yl)-1,2,5-oxadiazole 2-oxide (8c), ^{1}H NMR, CDCl$_3$, 400 MHz

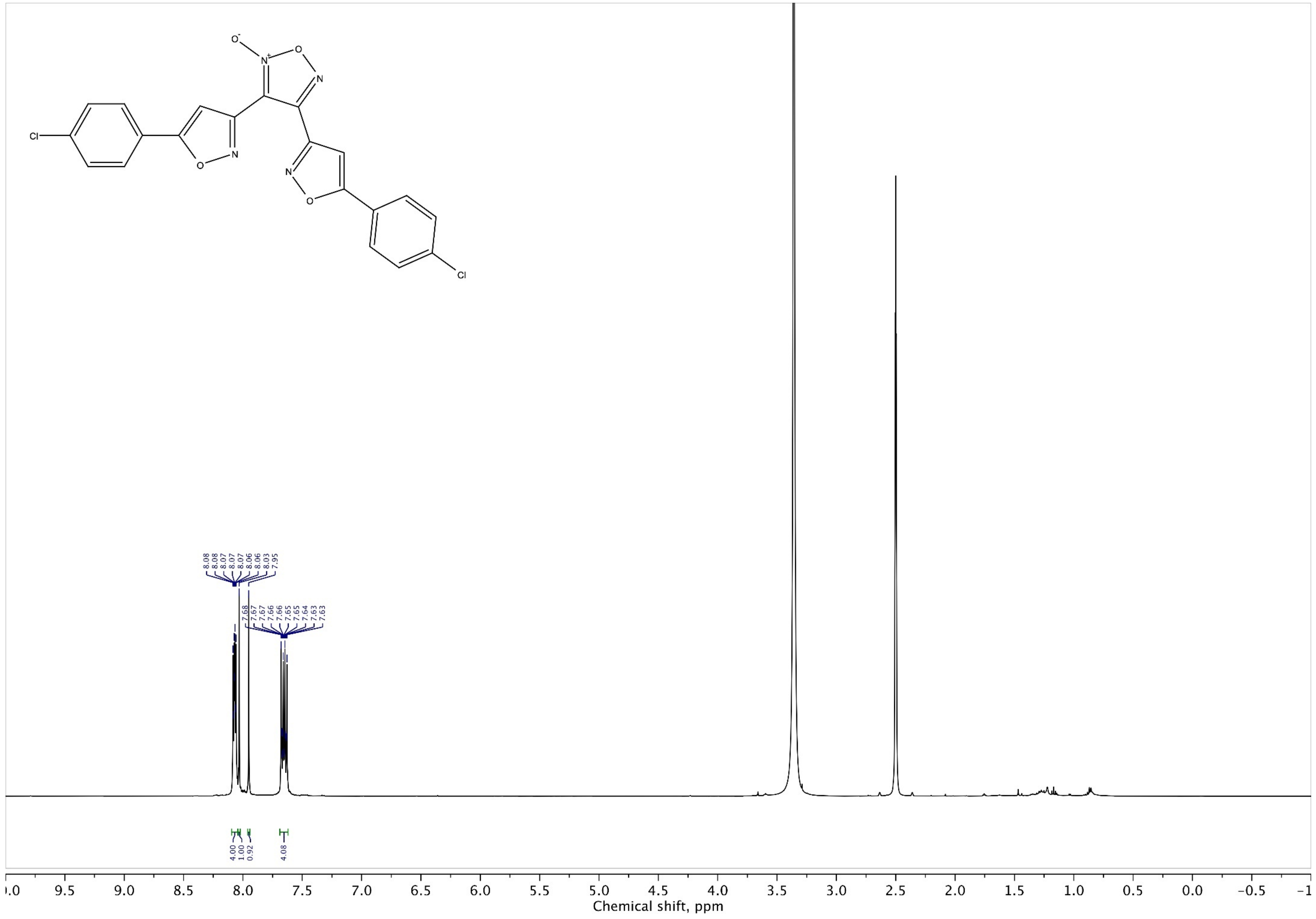

3,4-bis(3-(4-Chlorophenyl)isoxazol-5-yl)-1,2,5-oxadiazole 2-oxide (8c), $^{13}C\{^1H\}$ NMR, $CDCl_3$, 100 MHz

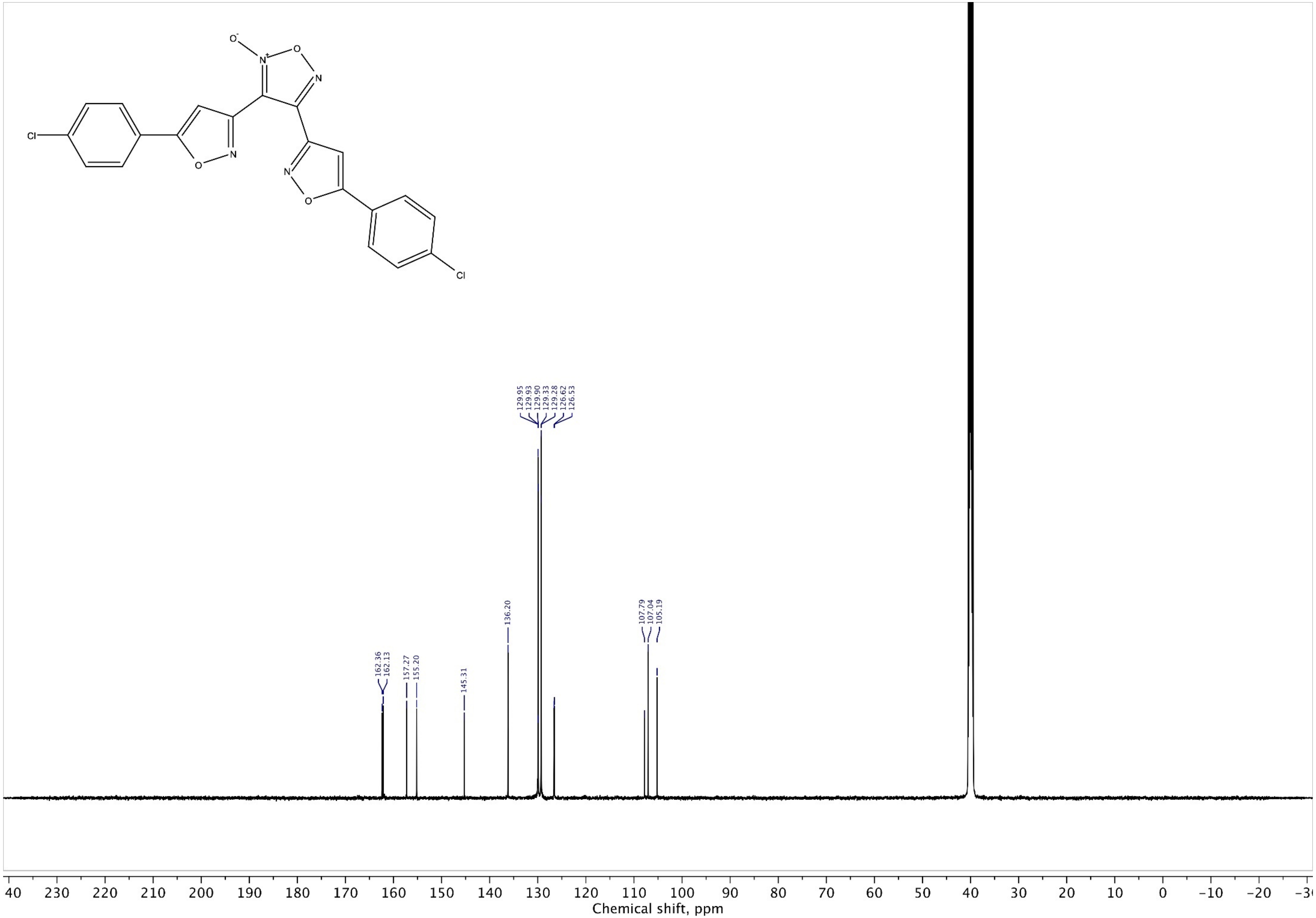

3,4-bis(3-(4-Chlorophenyl)isoxazol-5-yl)-1,2,5-oxadiazole 2-oxide (8c), DEPT, $CDCl_3$, 100 MHz

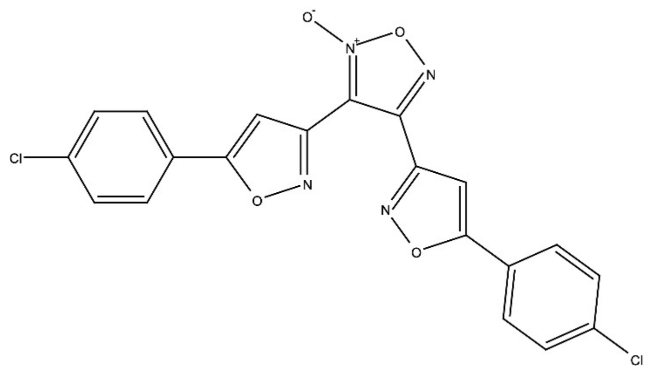

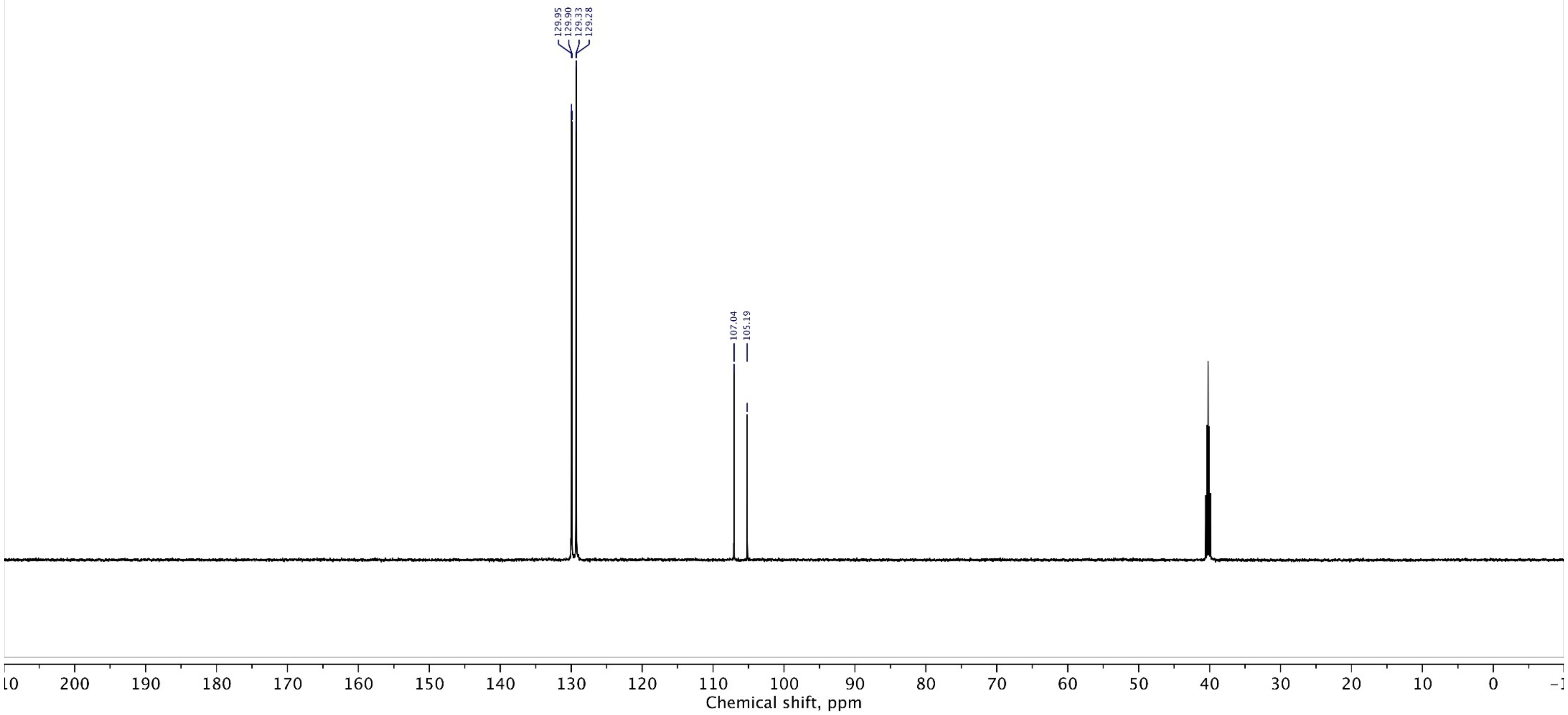

Methyl 3-(4-acetyl-5-methyl-3-phenyl-1*H*-pyrrole-2-carbonyl)isoxazole-5-carboxylate (5a), ^{1}H NMR, $CDCl_3$, 400 MHz

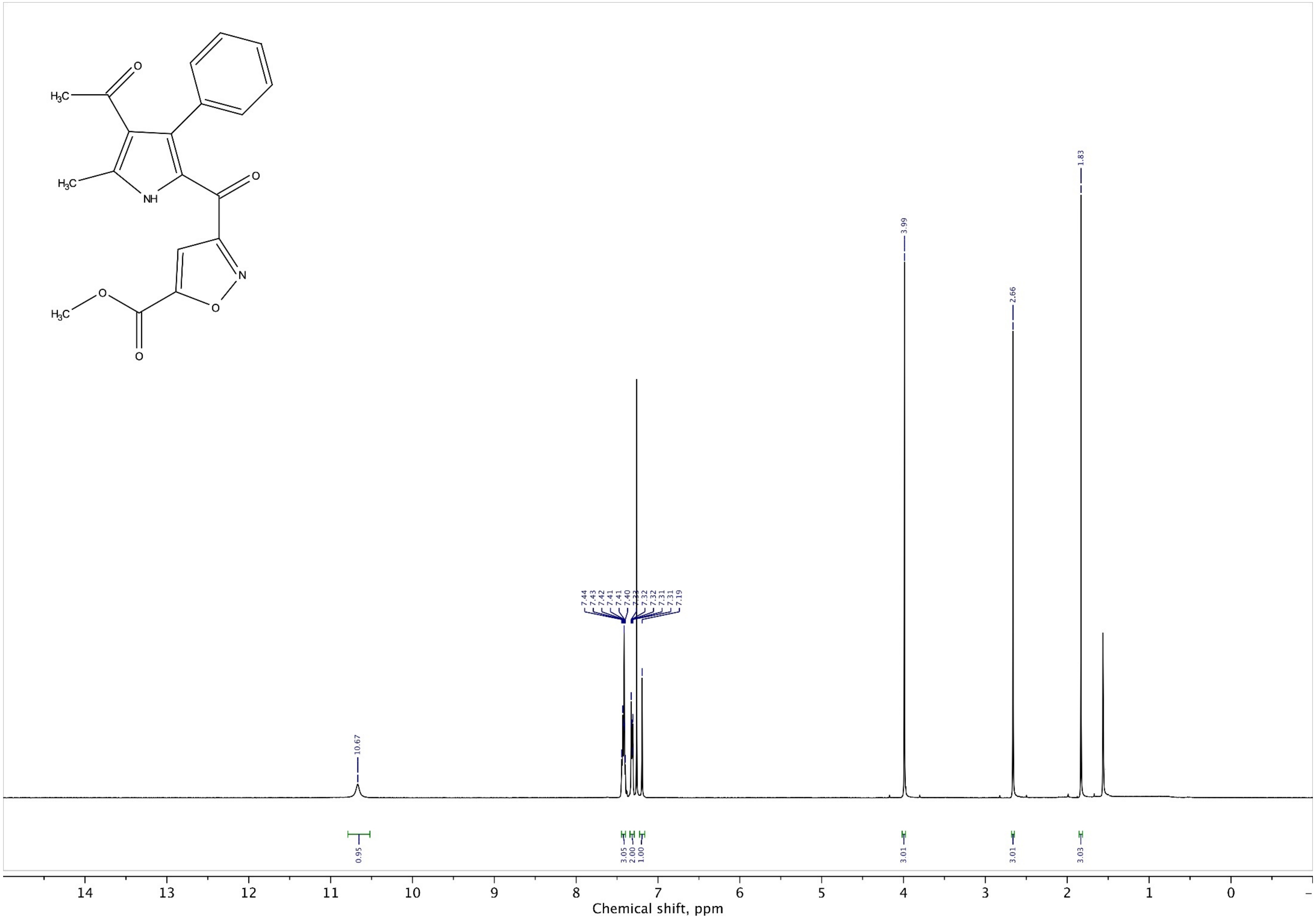

Methyl 3-(4-acetyl-5-methyl-3-phenyl-1*H*-pyrrole-2-carbonyl)isoxazole-5-carboxylate (5a), $^{13}C\{^{1}H\}$ NMR, $CDCl_3$, 100 MHz

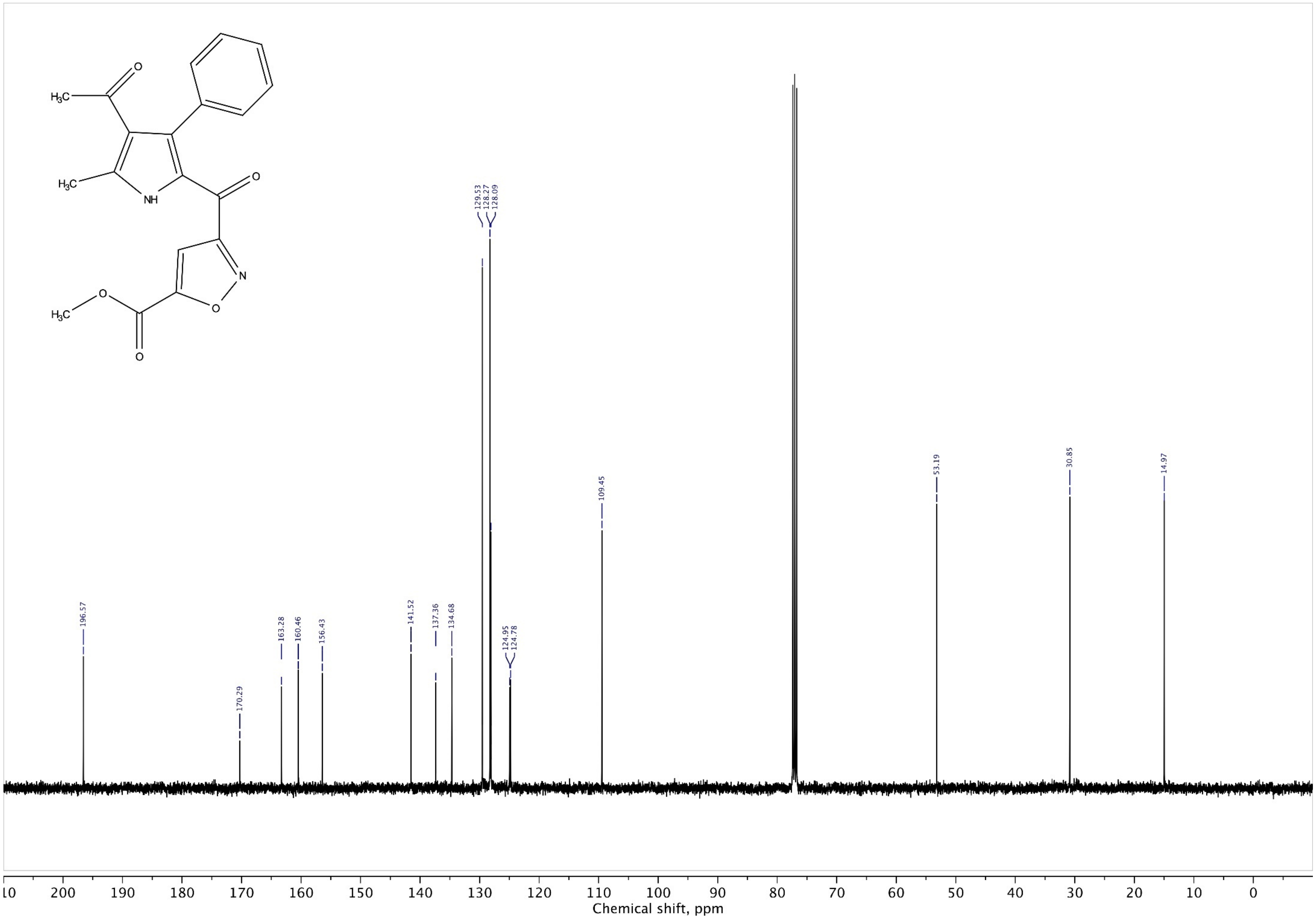

Methyl 3-(4-acetyl-5-methyl-3-phenyl-1*H*-pyrrole-2-carbonyl)isoxazole-5-carboxylate (5a), DEPT, $CDCl_3$, 100 MHz

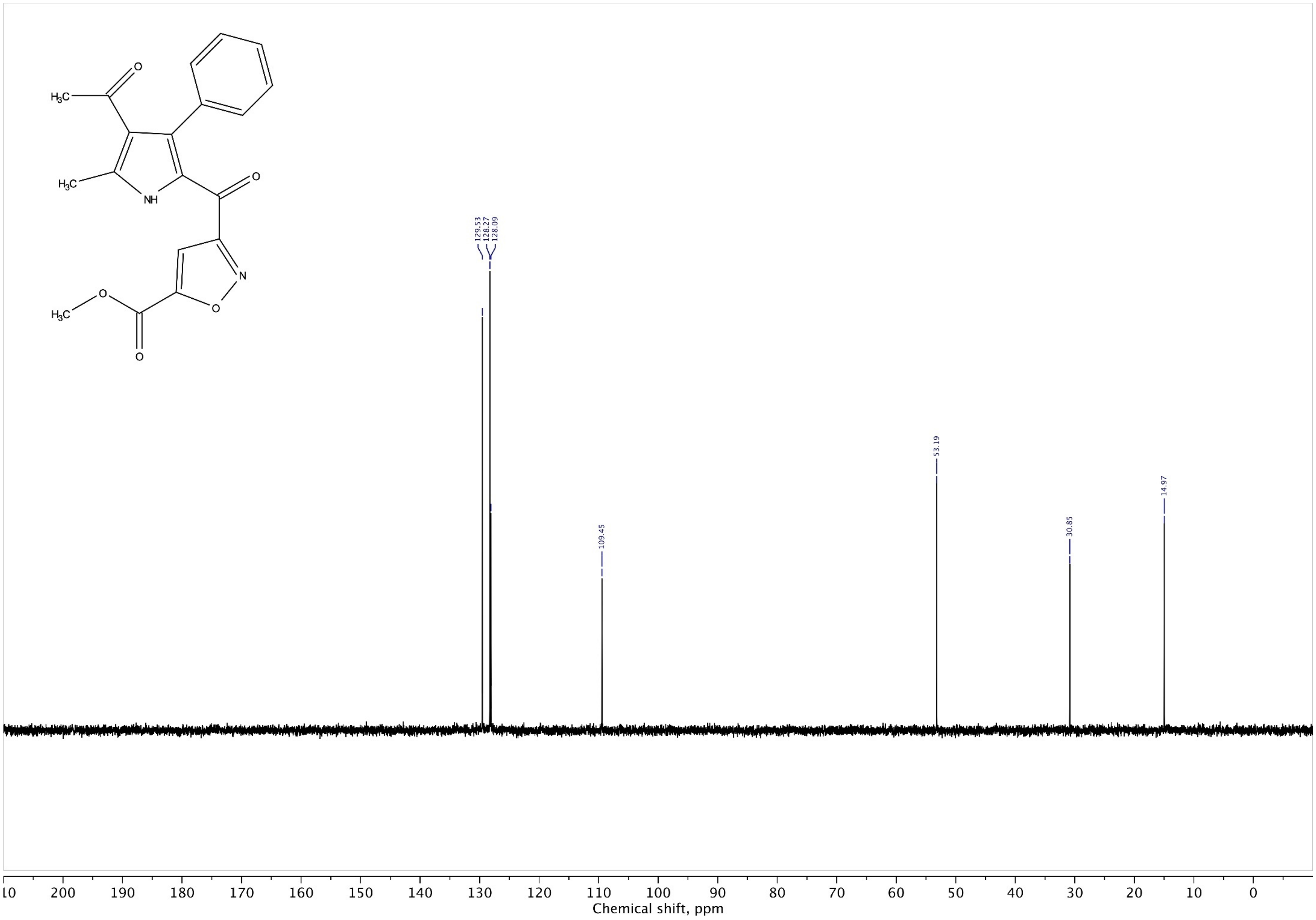

1-(5-(5-(Chloromethyl)isoxazole-3-carbonyl)-2-methyl-4-phenyl-1*H*-pyrrol-3-yl)ethan-1-one (5b), ^{1}H NMR, $CDCl_3$, 400 MHz

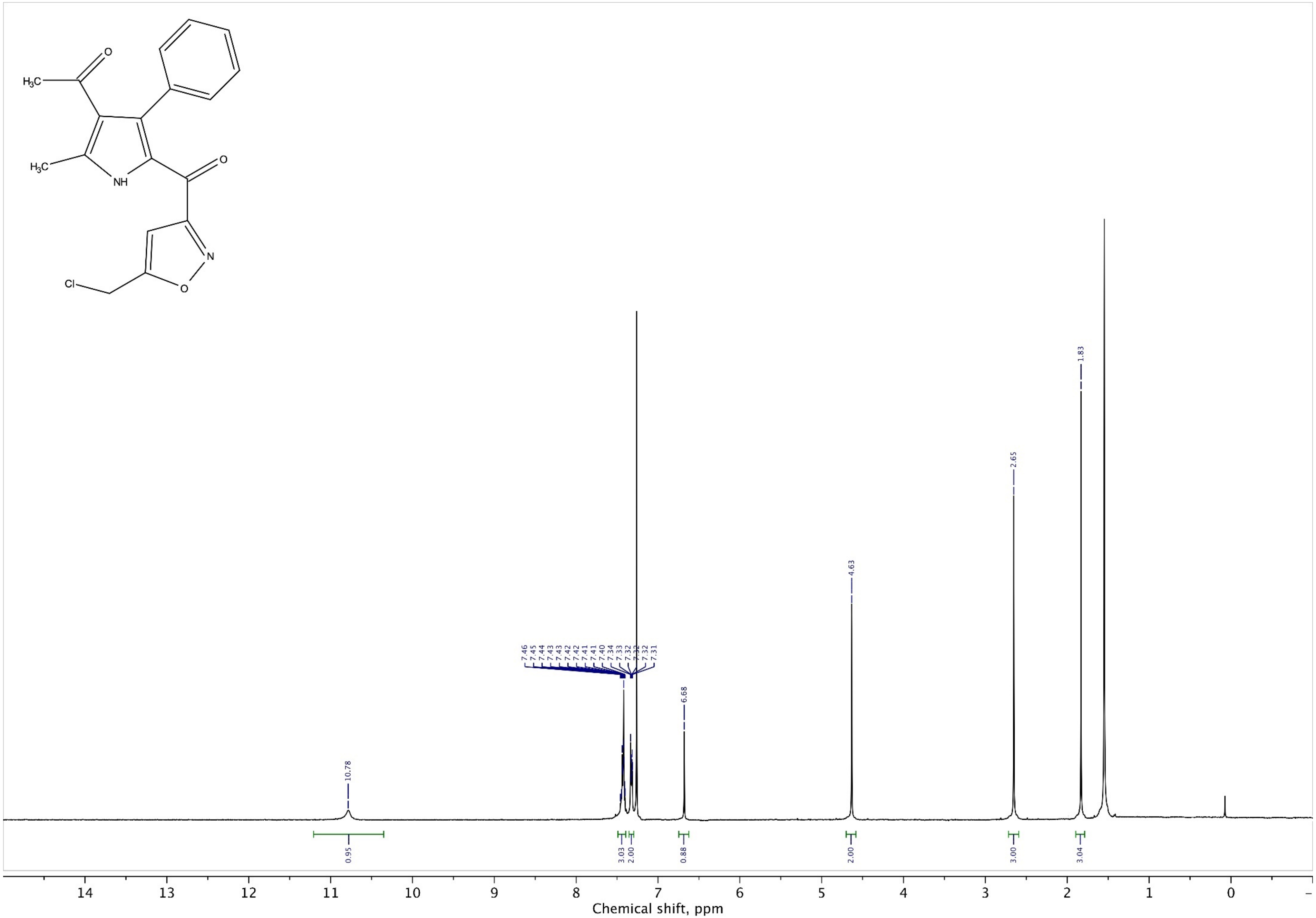

1-(5-(5-(Chloromethyl)isoxazole-3-carbonyl)-2-methyl-4-phenyl-1*H*-pyrrol-3-yl)ethan-1-one (5b), $^{13}C\{^1H\}$ NMR, $CDCl_3$, 100 MHz

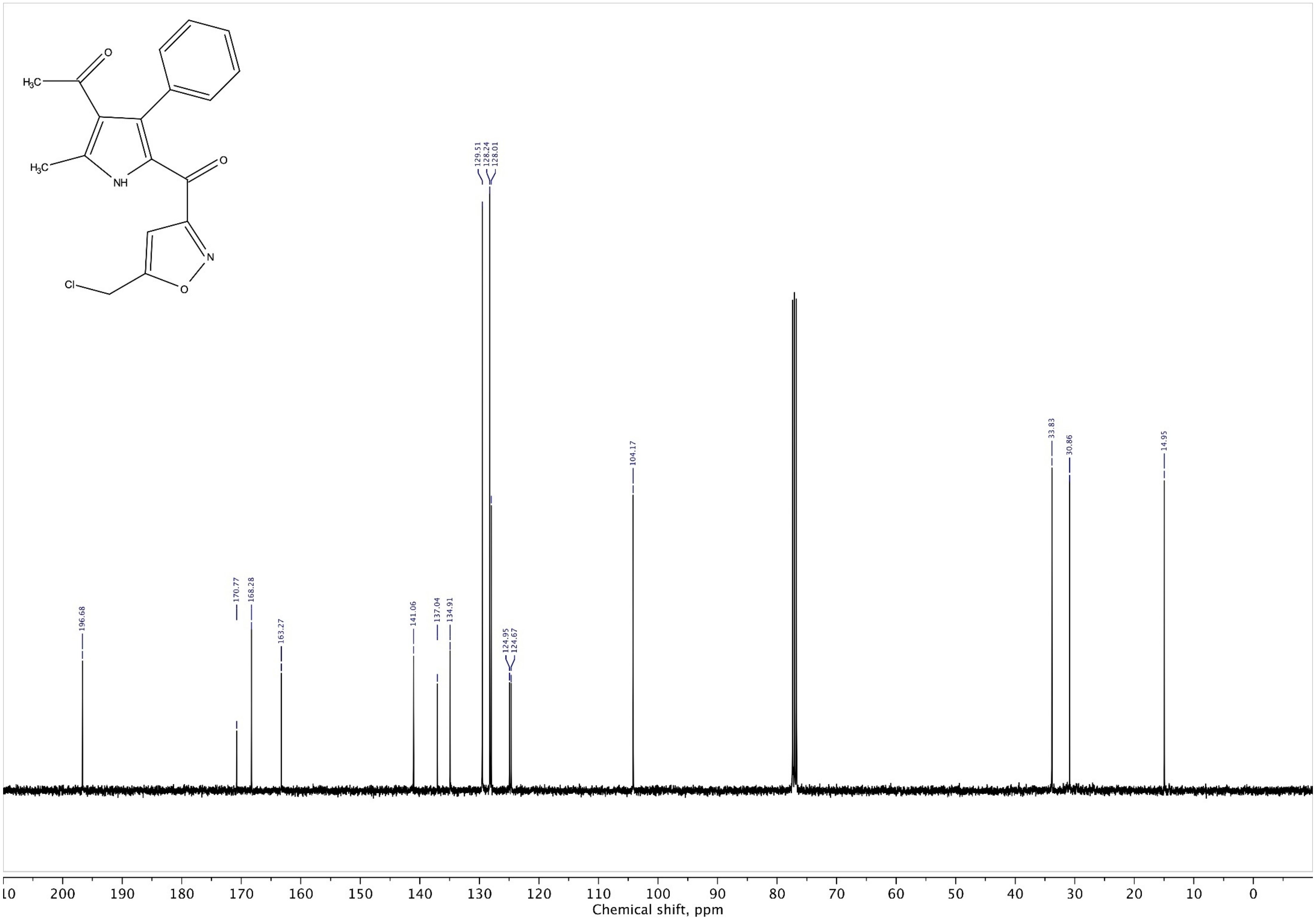

1-(5-(5-(Chloromethyl)isoxazole-3-carbonyl)-2-methyl-4-phenyl-1*H*-pyrrol-3-yl)ethan-1-one (5b), DEPT, $CDCl_3$, 100 MHz

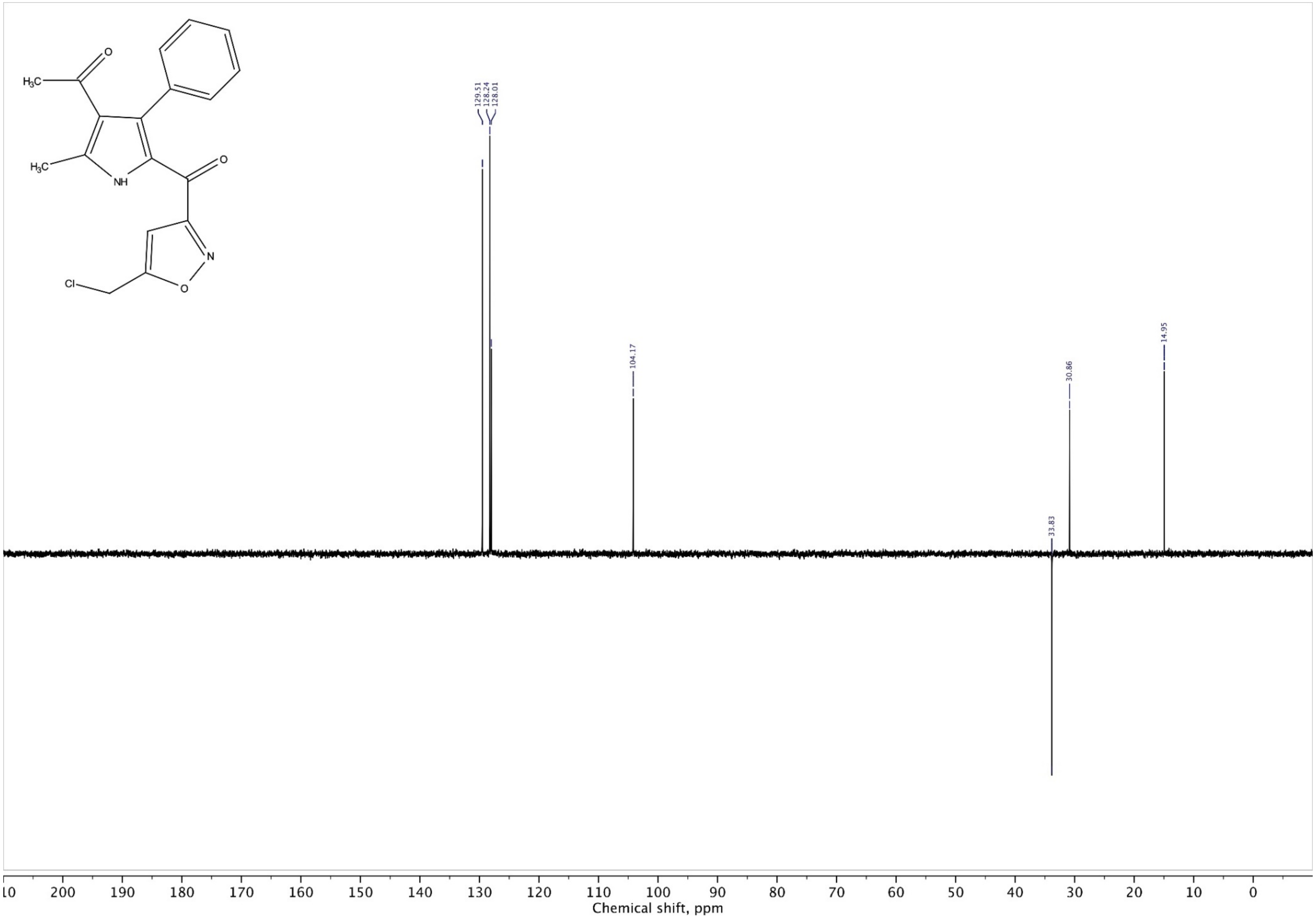

Methyl 3-(4-acetyl-3-(4-methoxyphenyl)-5-methyl-1*H*-pyrrole-2-carbonyl)isoxazole-5-carboxylate (5c), ^{1}H NMR, $CDCl_3$, 400 MHz

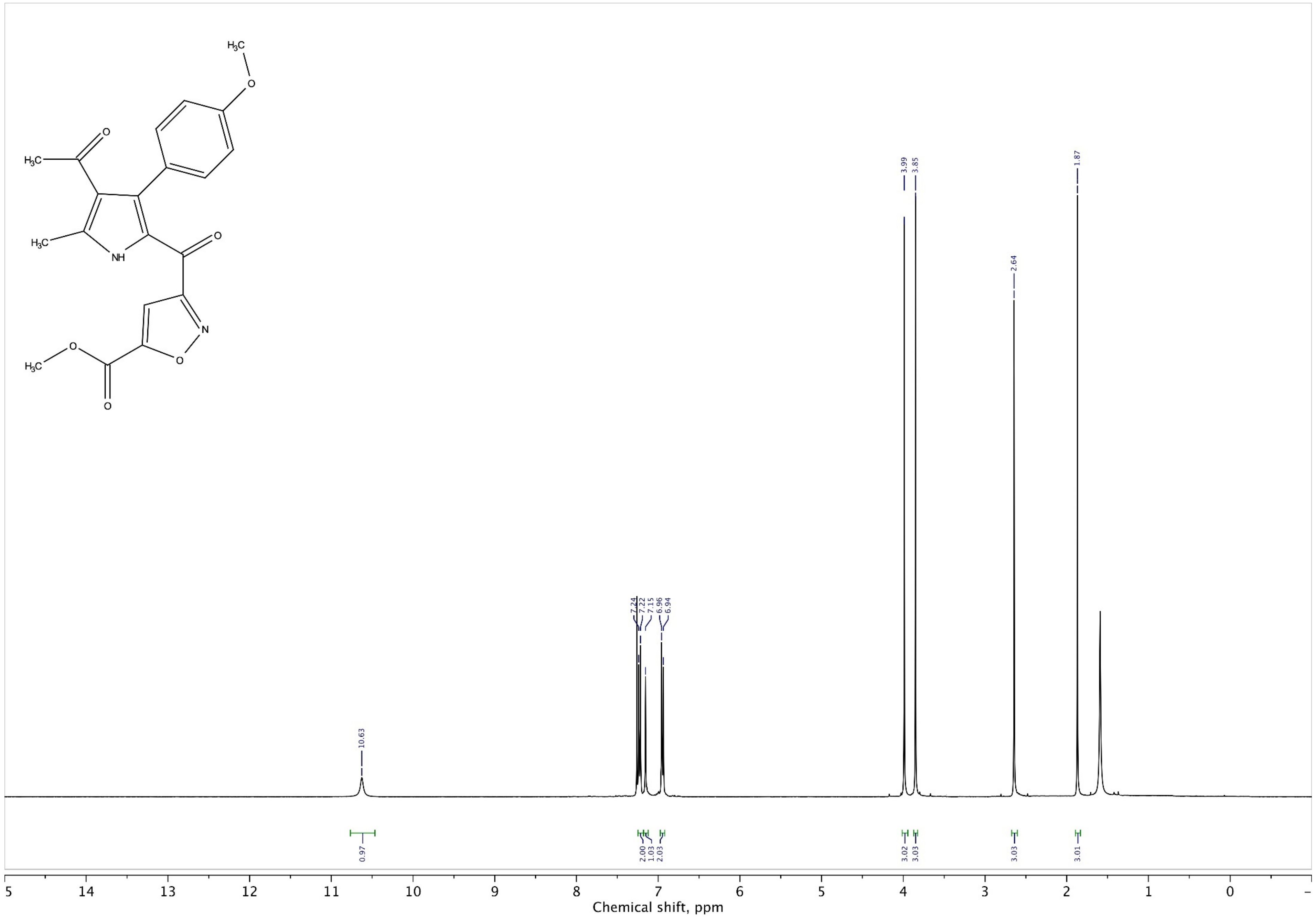

Methyl 3-(4-acetyl-3-(4-methoxyphenyl)-5-methyl-1*H*-pyrrole-2-carbonyl)isoxazole-5-carboxylate (5c), $^{13}C\{^1H\}$ NMR, $CDCl_3$, 100 MHz

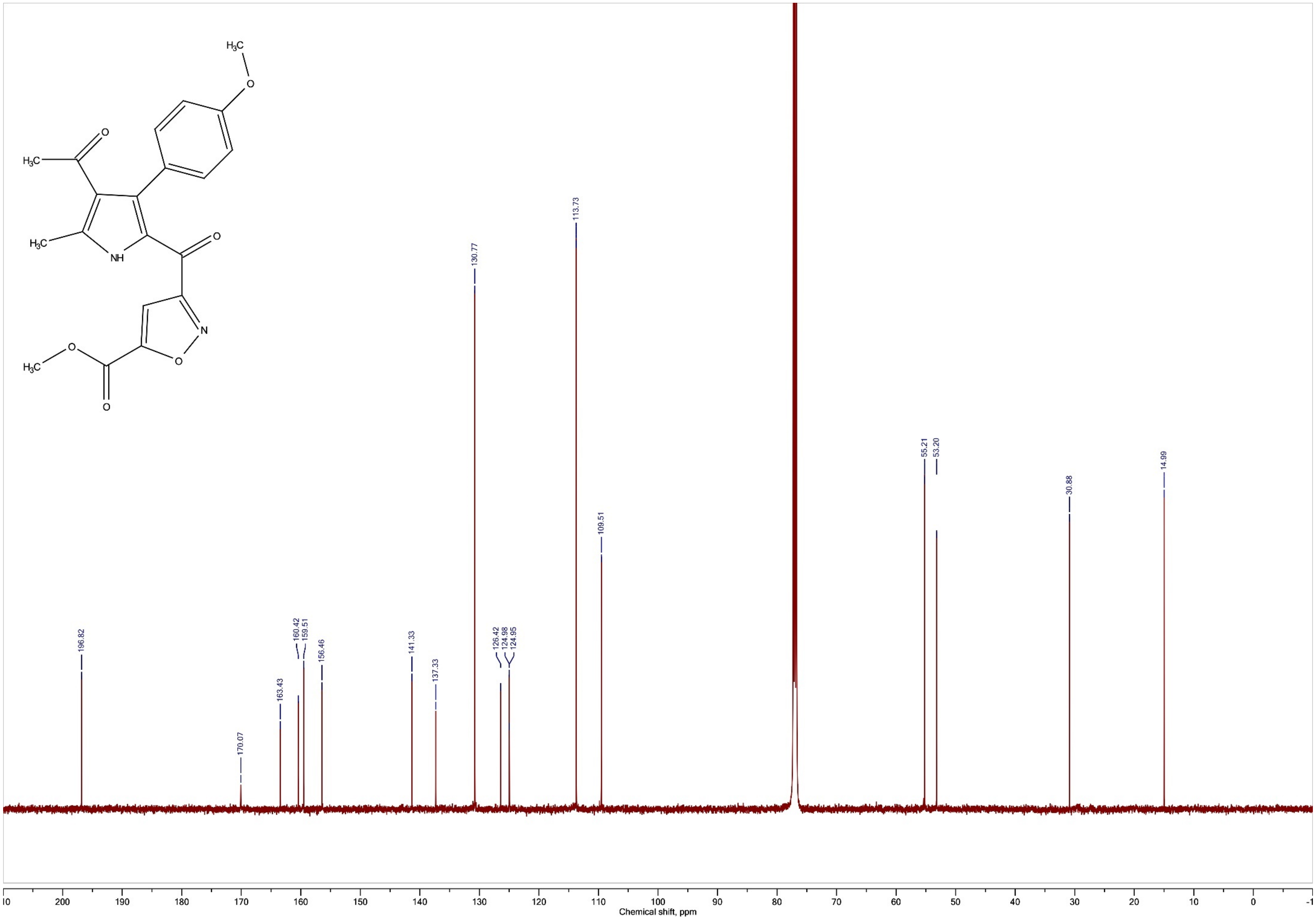

Methyl 3-(4-acetyl-3-(4-methoxyphenyl)-5-methyl-1*H*-pyrrole-2-carbonyl)isoxazole-5-carboxylate (5c), DEPT, $CDCl_3$, 100 MHz

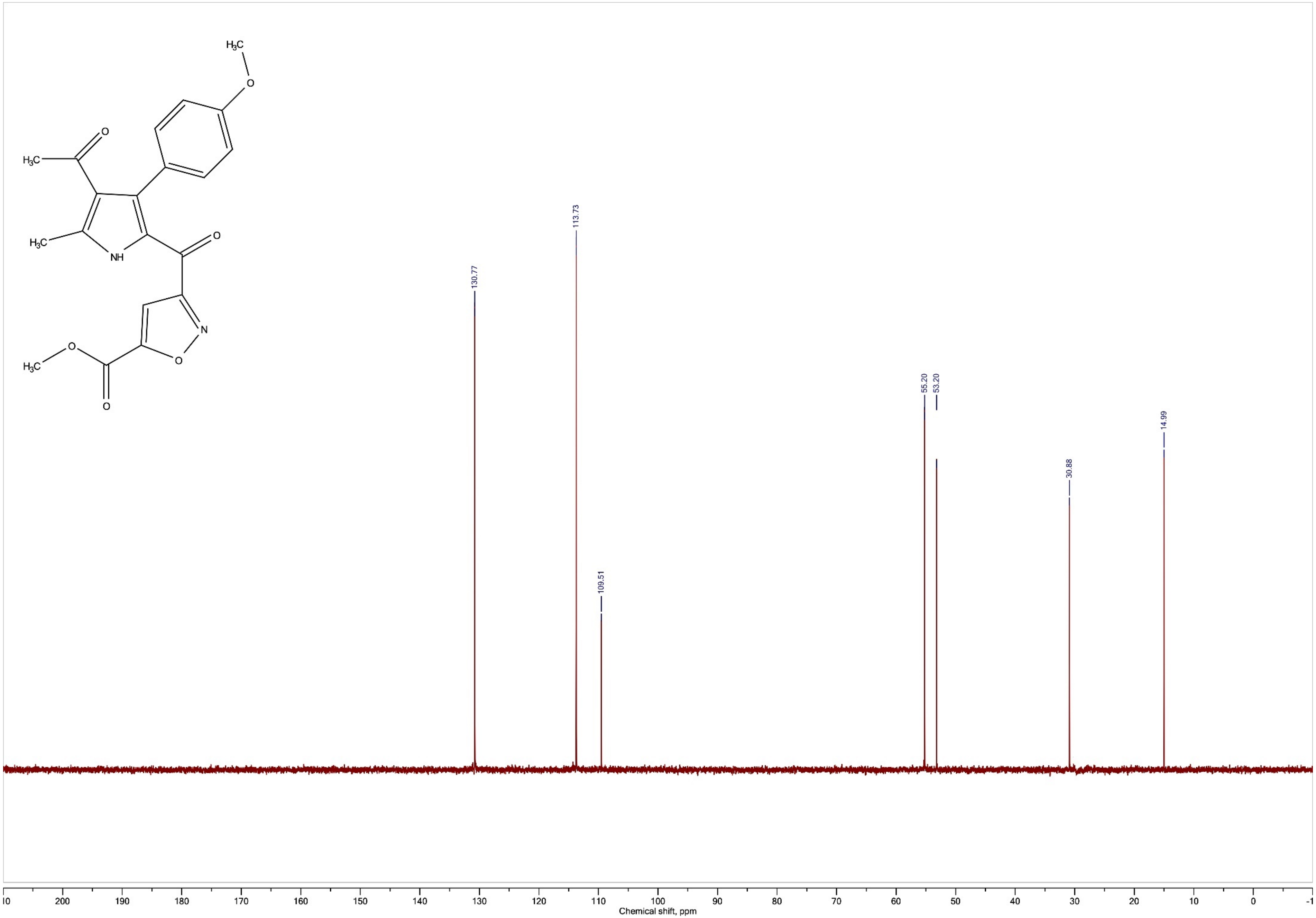

(3-(4-Acetyl-3-(4-chlorophenyl)-5-methyl-1*H*-pyrrole-2-carbonyl)isoxazol-5-yl)methyl benzenesulfonate (5d), ^{1}H NMR, $CDCl_3$, 400 MHz

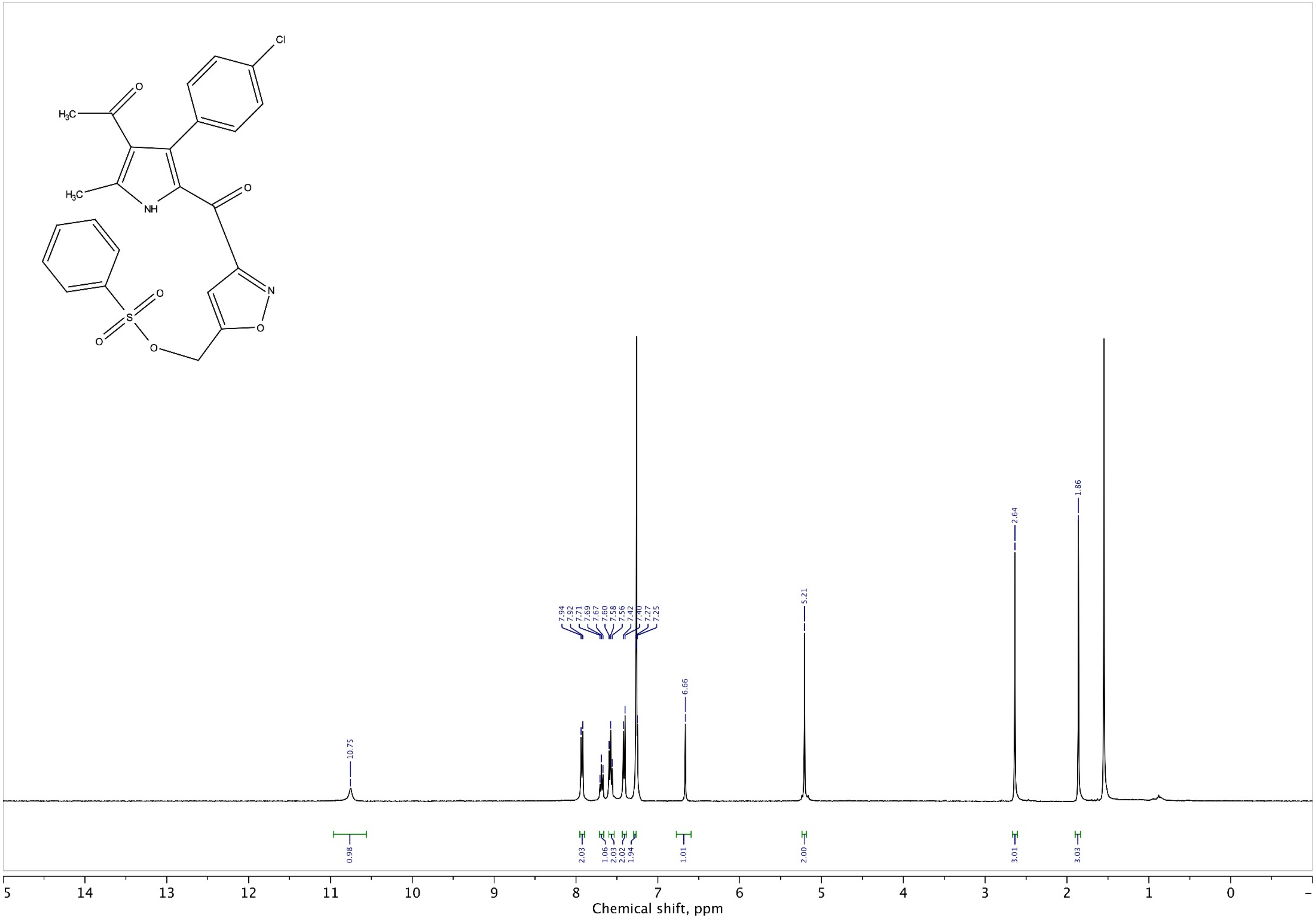

(3-(4-Acetyl-3-(4-chlorophenyl)-5-methyl-1*H*-pyrrole-2-carbonyl)isoxazol-5-yl)methyl benzenesulfonate (5d), $^{13}C\{^{1}H\}$ NMR, $CDCl_3$, 100 MHz

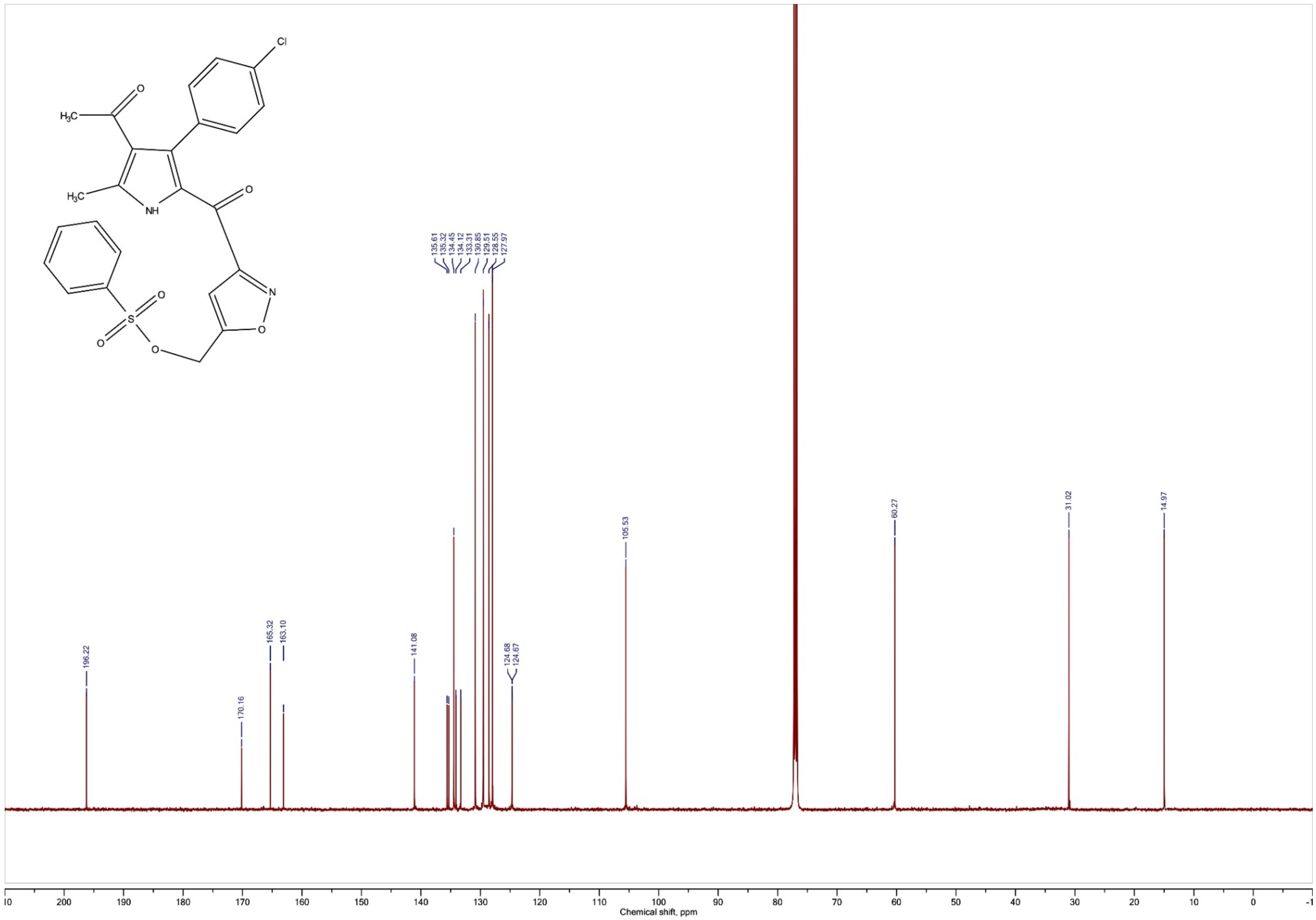

(3-(4-Acetyl-3-(4-chlorophenyl)-5-methyl-1*H*-pyrrole-2-carbonyl)isoxazol-5-yl)methyl benzenesulfonate (5d), DEPT, $CDCl_3$, 100 MHz

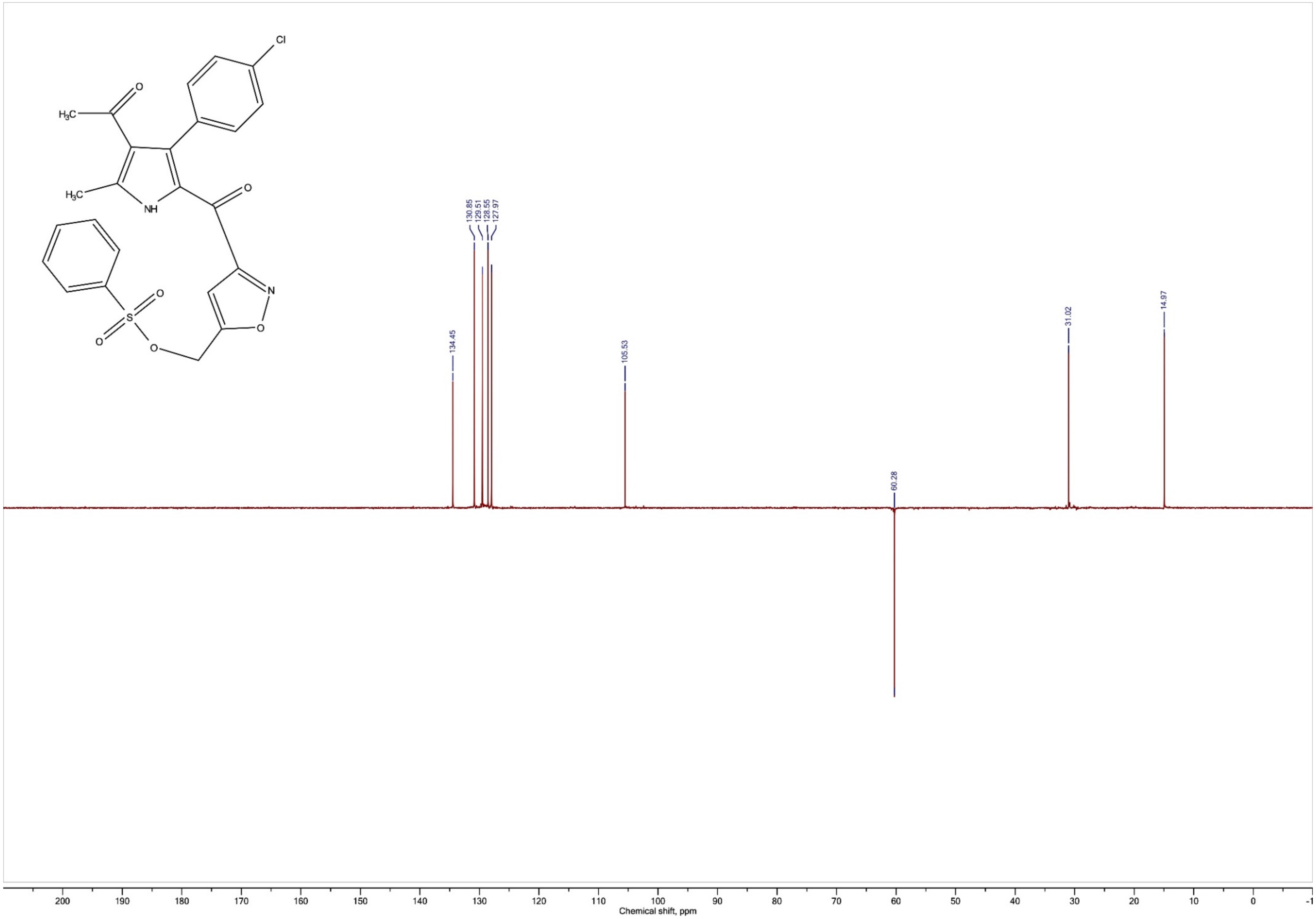

Methyl 3-(3-phenyl-5-(thiophen-2-yl)-4-(thiophene-2-carbonyl)-1*H*-pyrrole-2-carbonyl)isoxazole-5-carboxylate (5e), ^{1}H NMR, DMSO-d_6, 400 MHz

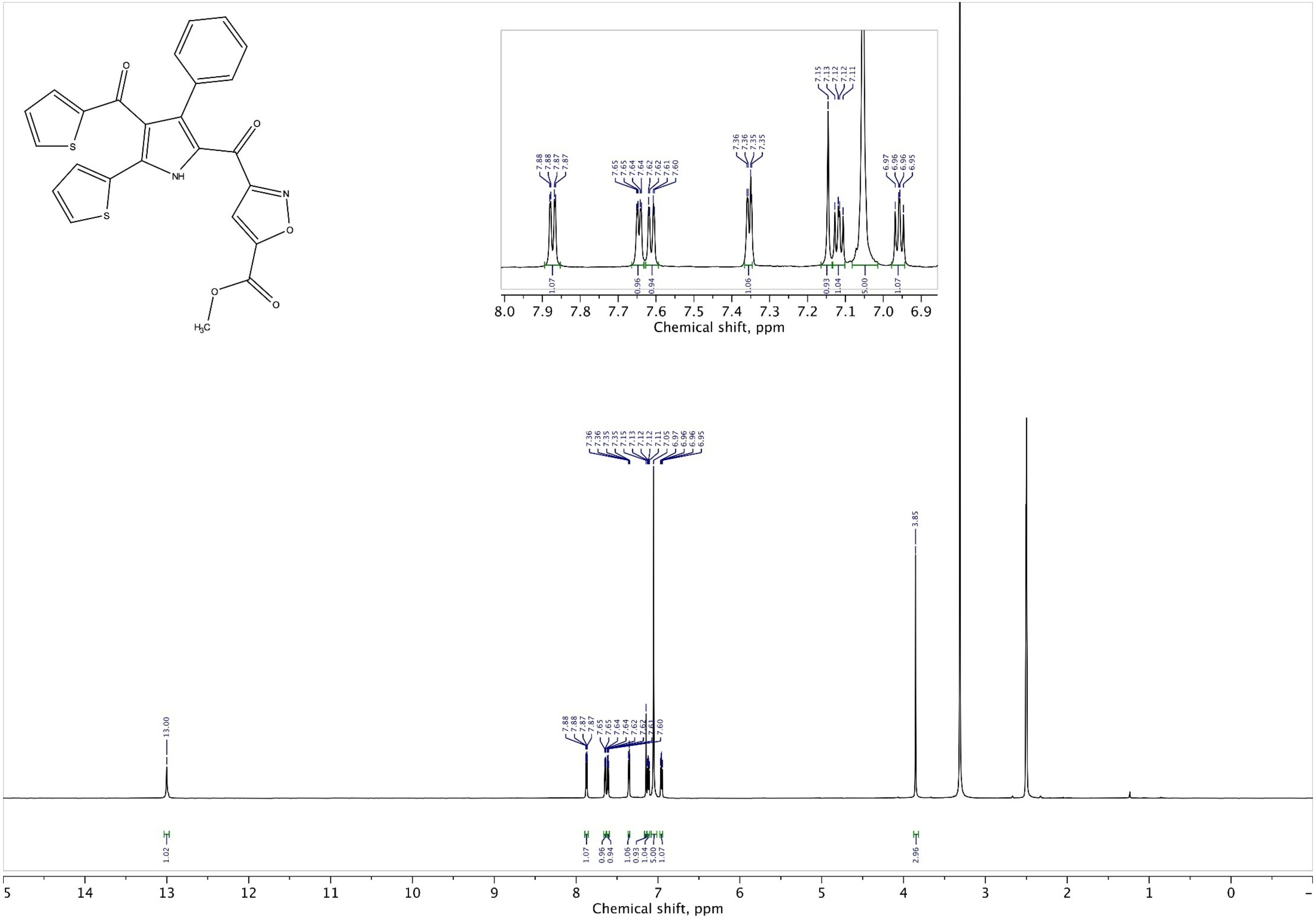

Methyl 3-(3-phenyl-5-(thiophen-2-yl)-4-(thiophene-2-carbonyl)-1*H*-pyrrole-2-carbonyl)isoxazole-5-carboxylate (5e), $^{13}C\{^1H\}$ NMR, DMSO-d_6, 100 MHz

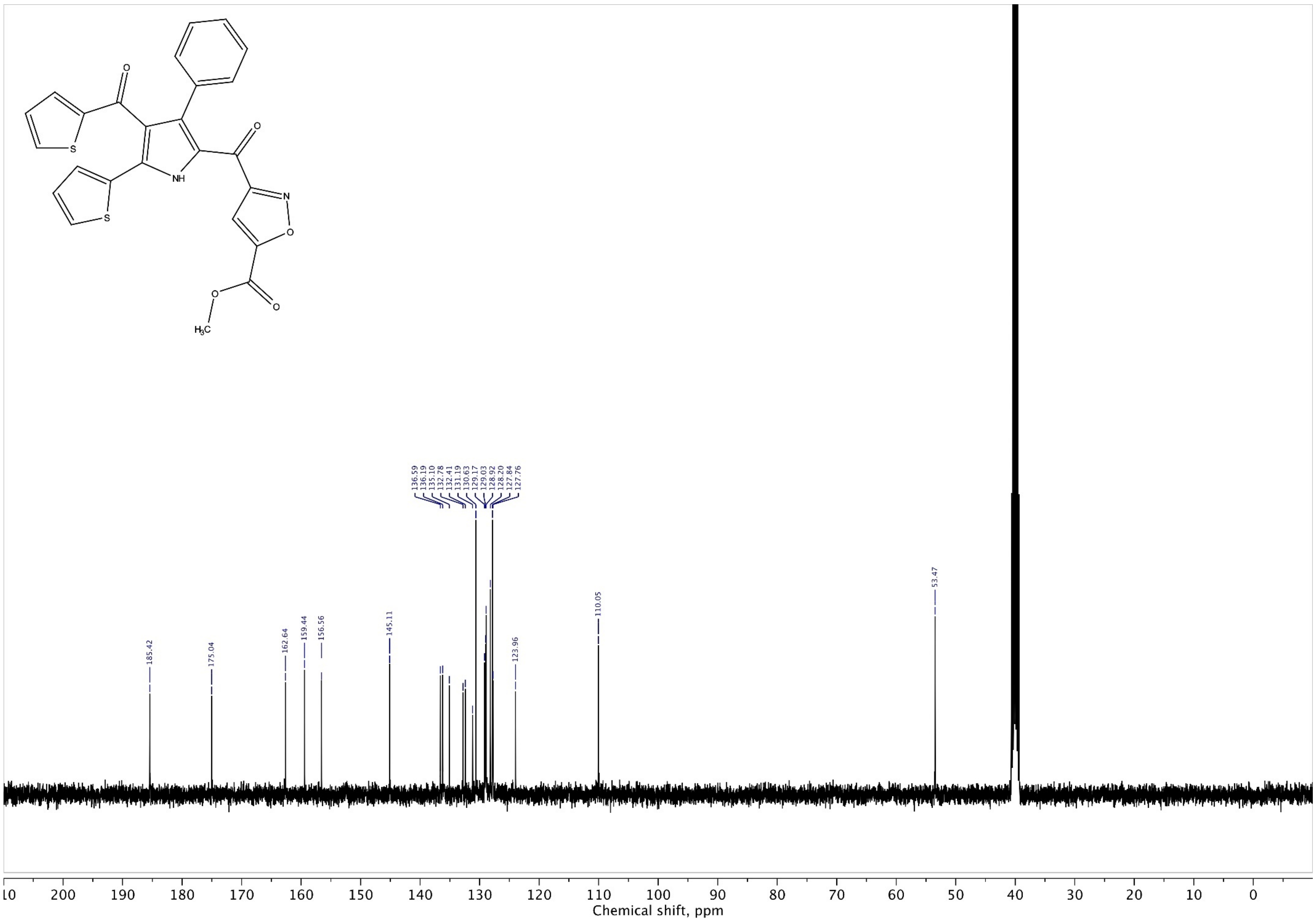

Methyl 3-(3-phenyl-5-(thiophen-2-yl)-4-(thiophene-2-carbonyl)-1*H*-pyrrole-2-carbonyl)isoxazole-5-carboxylate (5e), DEPT, DMSO-d_6, 100 MHz

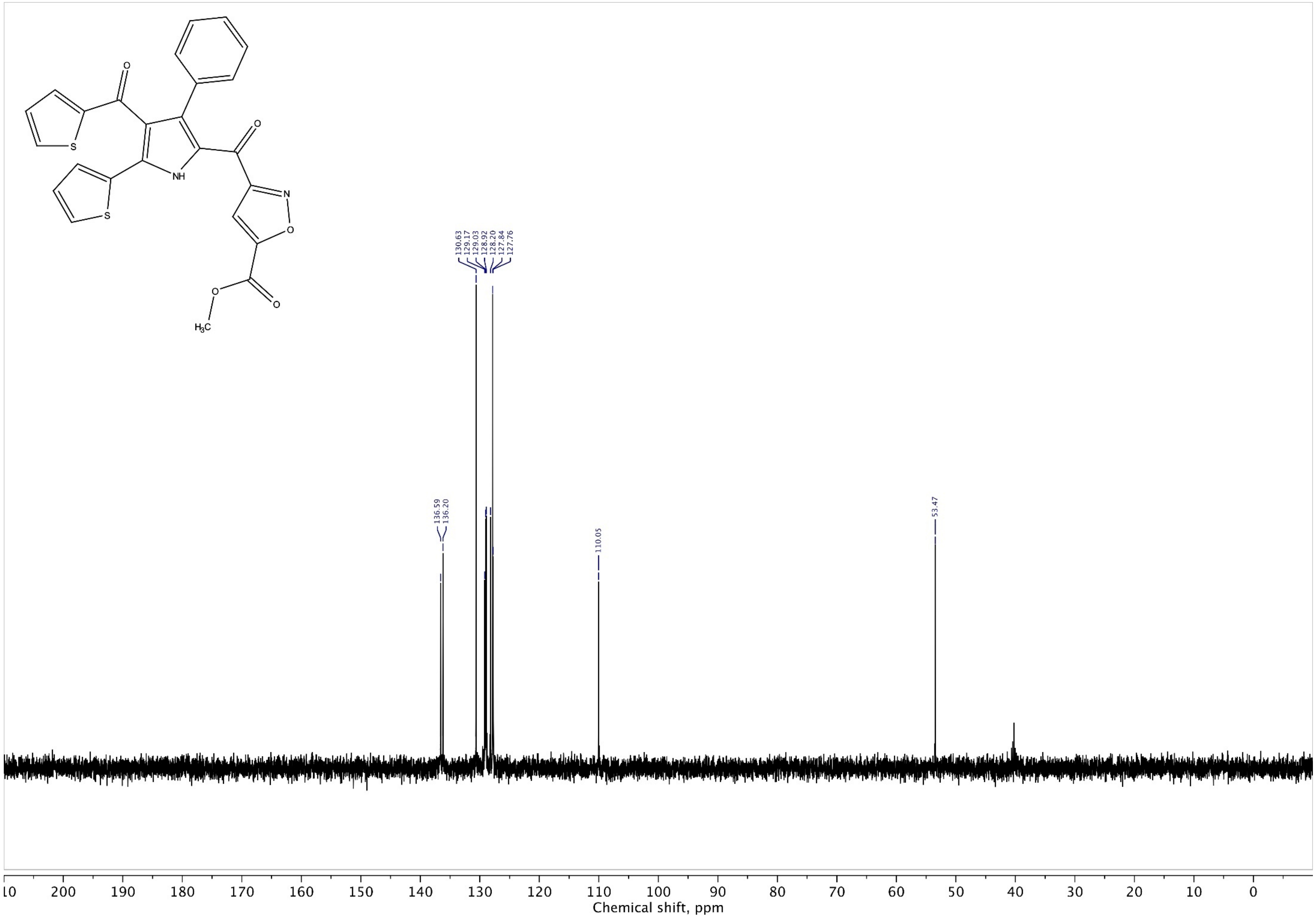

(5-(Chloromethyl)isoxazol-3-yl)(4-(4-methoxybenzoyl)-5-(4-methoxyphenyl)-3-phenyl-1*H*-pyrrol-2-yl)methanone (5f), ^{1}H NMR, DMSO-d_6, 400 MHz

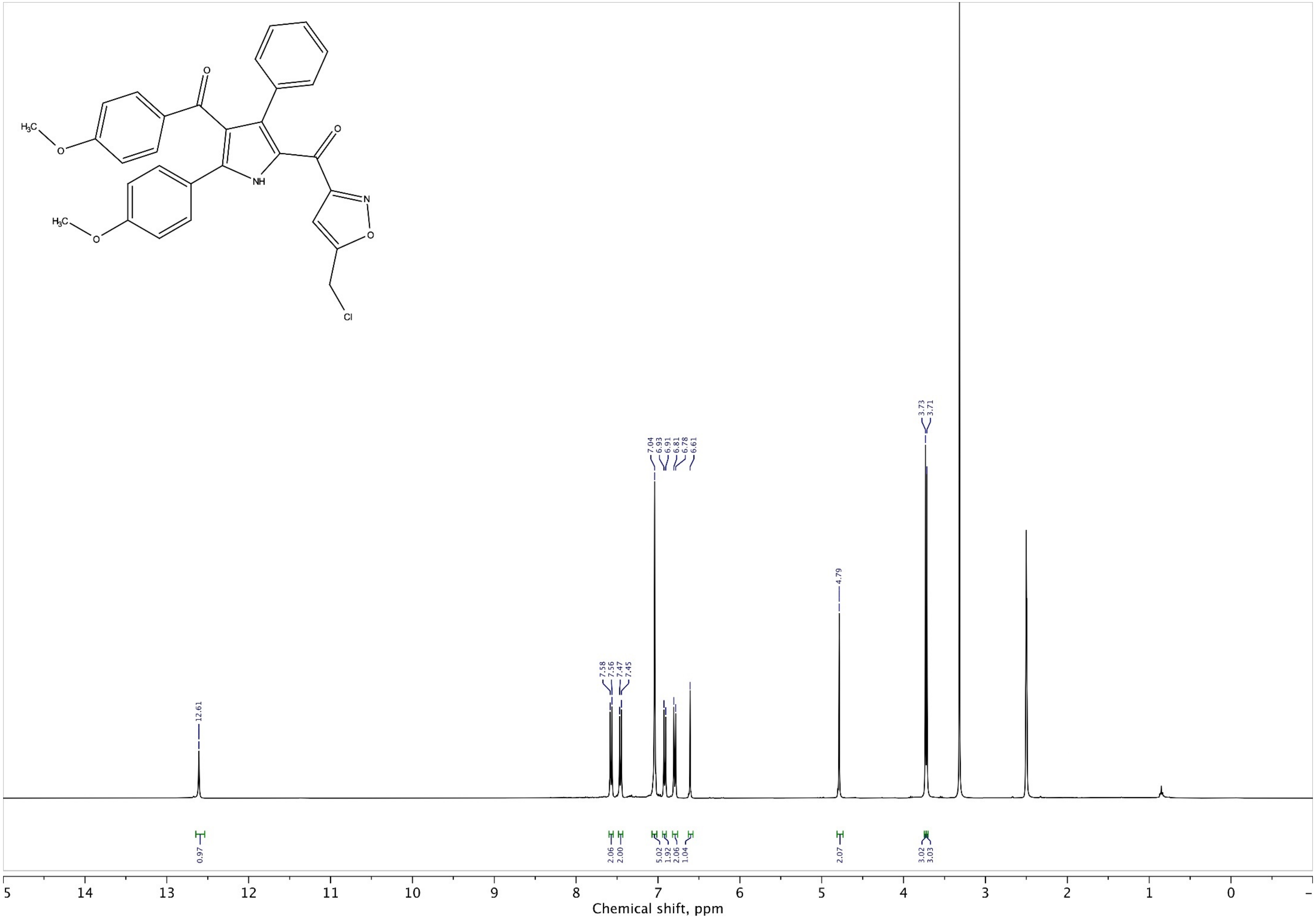

(5-(Chloromethyl)isoxazol-3-yl)(4-(4-methoxybenzoyl)-5-(4-methoxyphenyl)-3-phenyl-1*H*-pyrrol-2-yl)methanone (5f), $^{13}C\{^{1}H\}$ NMR, DMSO-d_6, 100 MHz

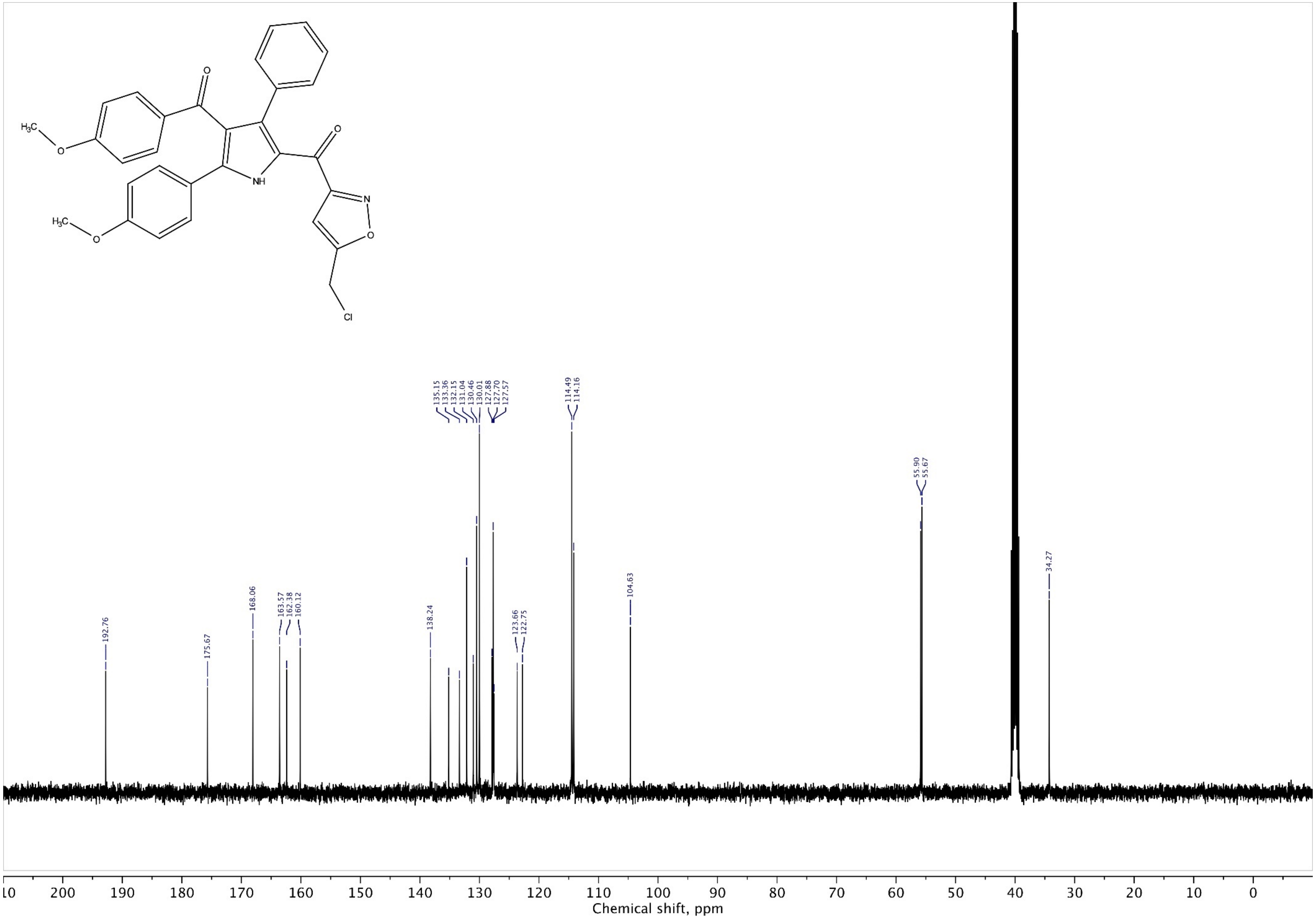

(5-(Chloromethyl)isoxazol-3-yl)(4-(4-methoxybenzoyl)-5-(4-methoxyphenyl)-3-phenyl-1*H*-pyrrol-2-yl)methanone (5f), DEPT, DMSO-d_6, 100 MHz

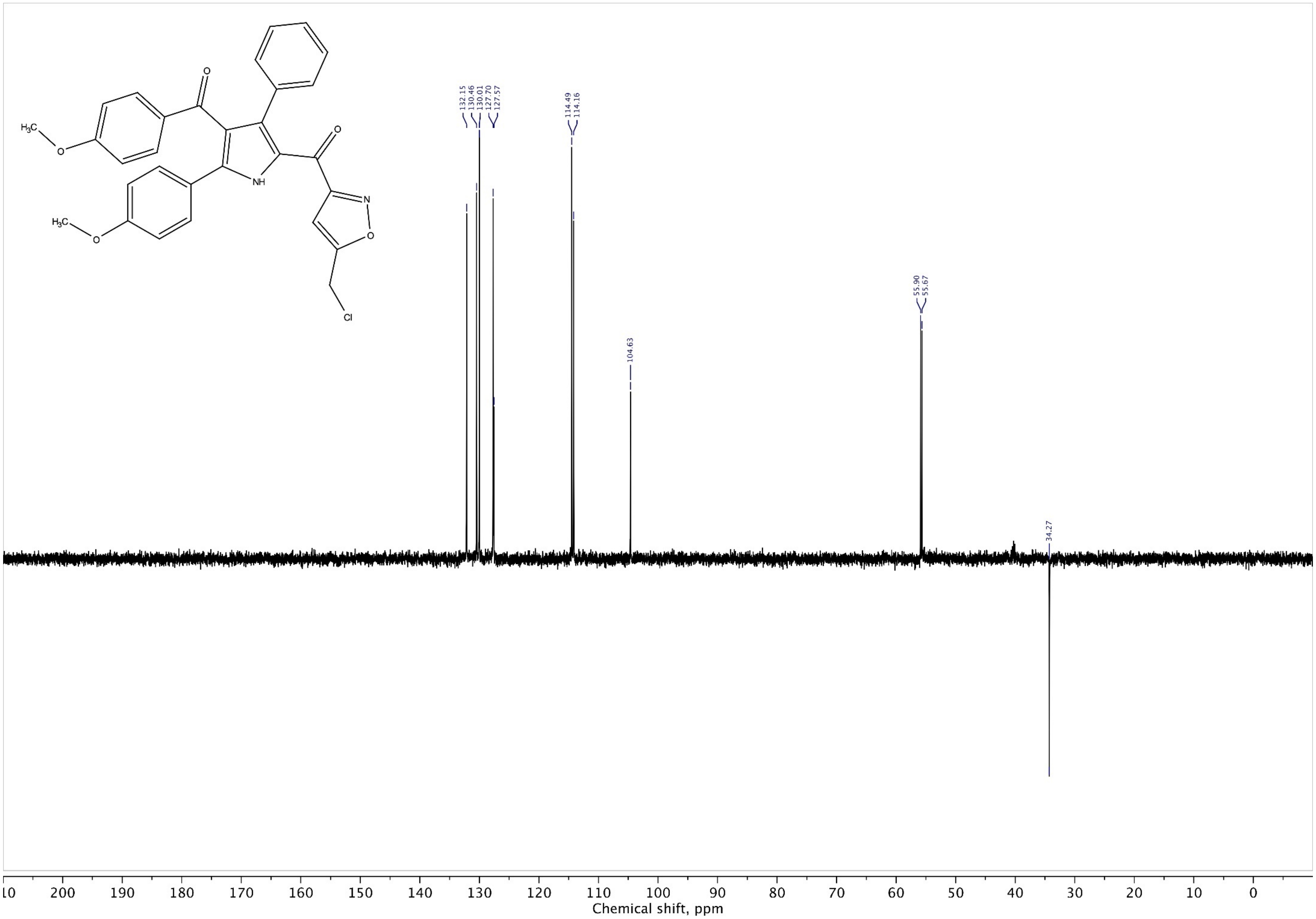

Methyl 3-(4-benzoyl-3-(4-fluorophenyl)-5-phenyl-1*H*-pyrrole-2-carbonyl)isoxazole-5-carboxylatebenzenesulfonate (5g), ^{1}H NMR, DMSO-d_6, 400 MHz

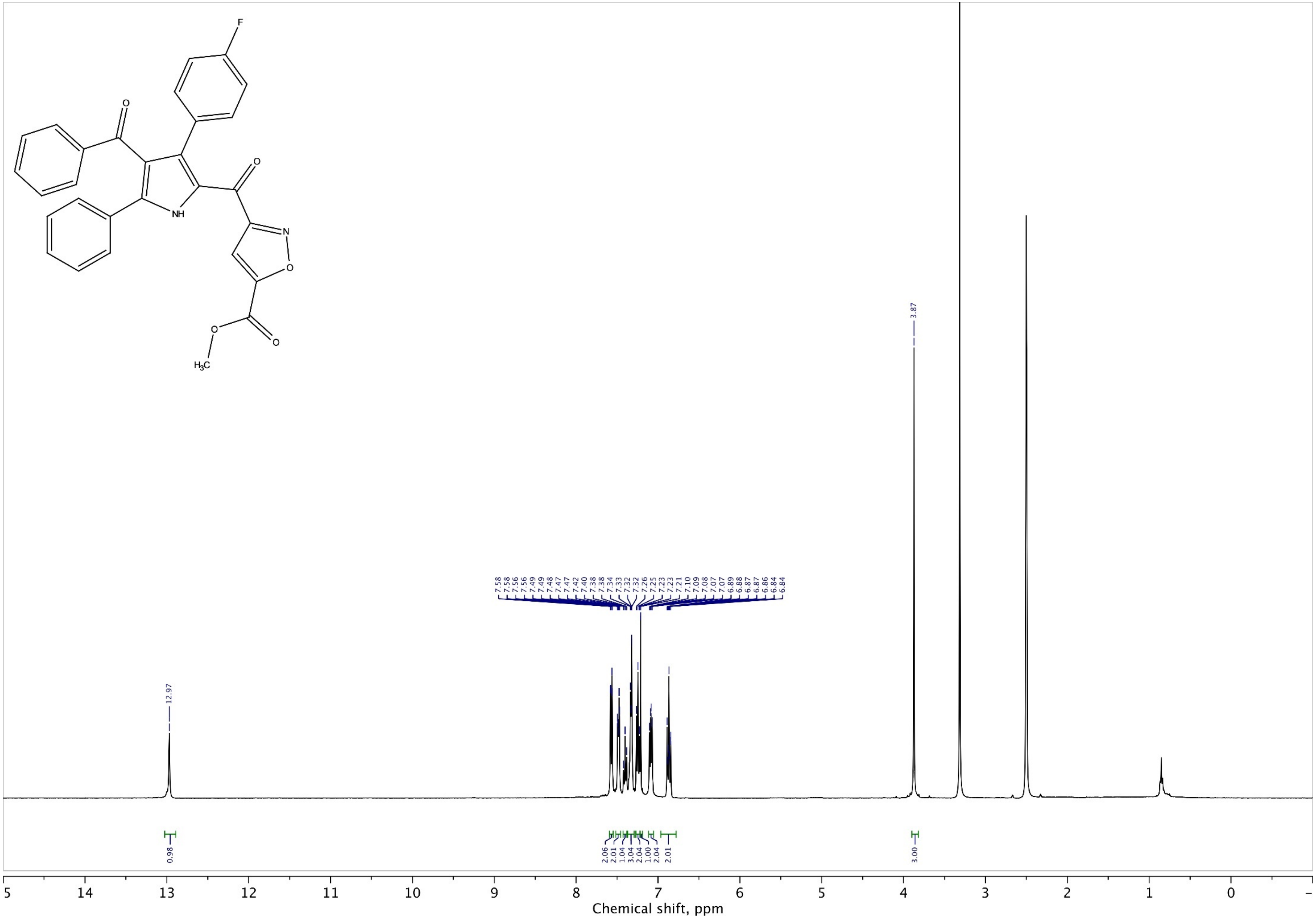

Methyl 3-(4-benzoyl-3-(4-fluorophenyl)-5-phenyl-1*H*-pyrrole-2-carbonyl)isoxazole-5-carboxylatebenzenesulfonate (5g), $^{13}C\{^{1}H\}$ NMR, DMSO-d_6, 100 MHz

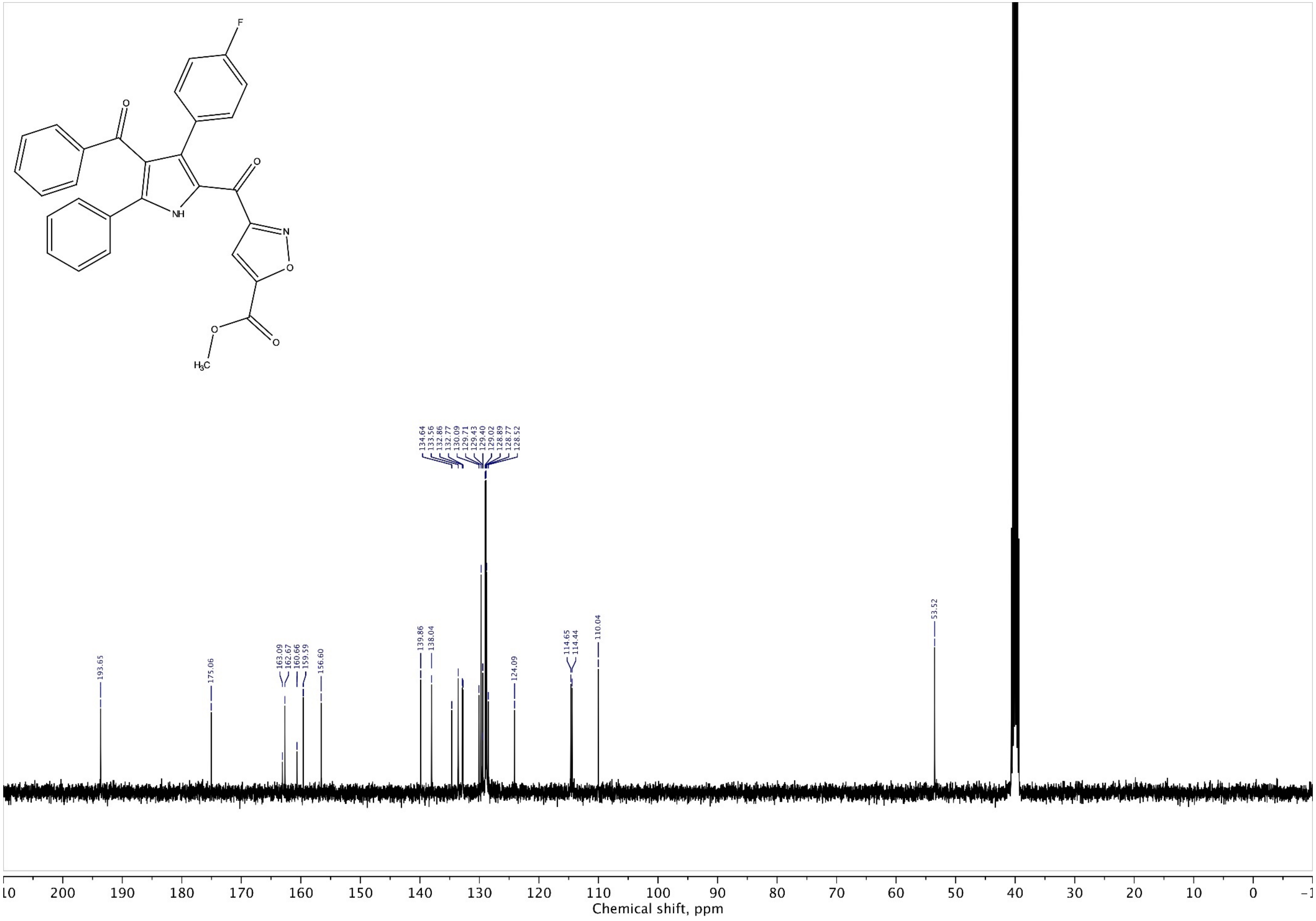

Methyl 3-(4-benzoyl-3-(4-fluorophenyl)-5-phenyl-1*H*-pyrrole-2-carbonyl)isoxazole-5-carboxylatebenzenesulfonate (5g), DEPT, DMSO-d_6, 100 MHz

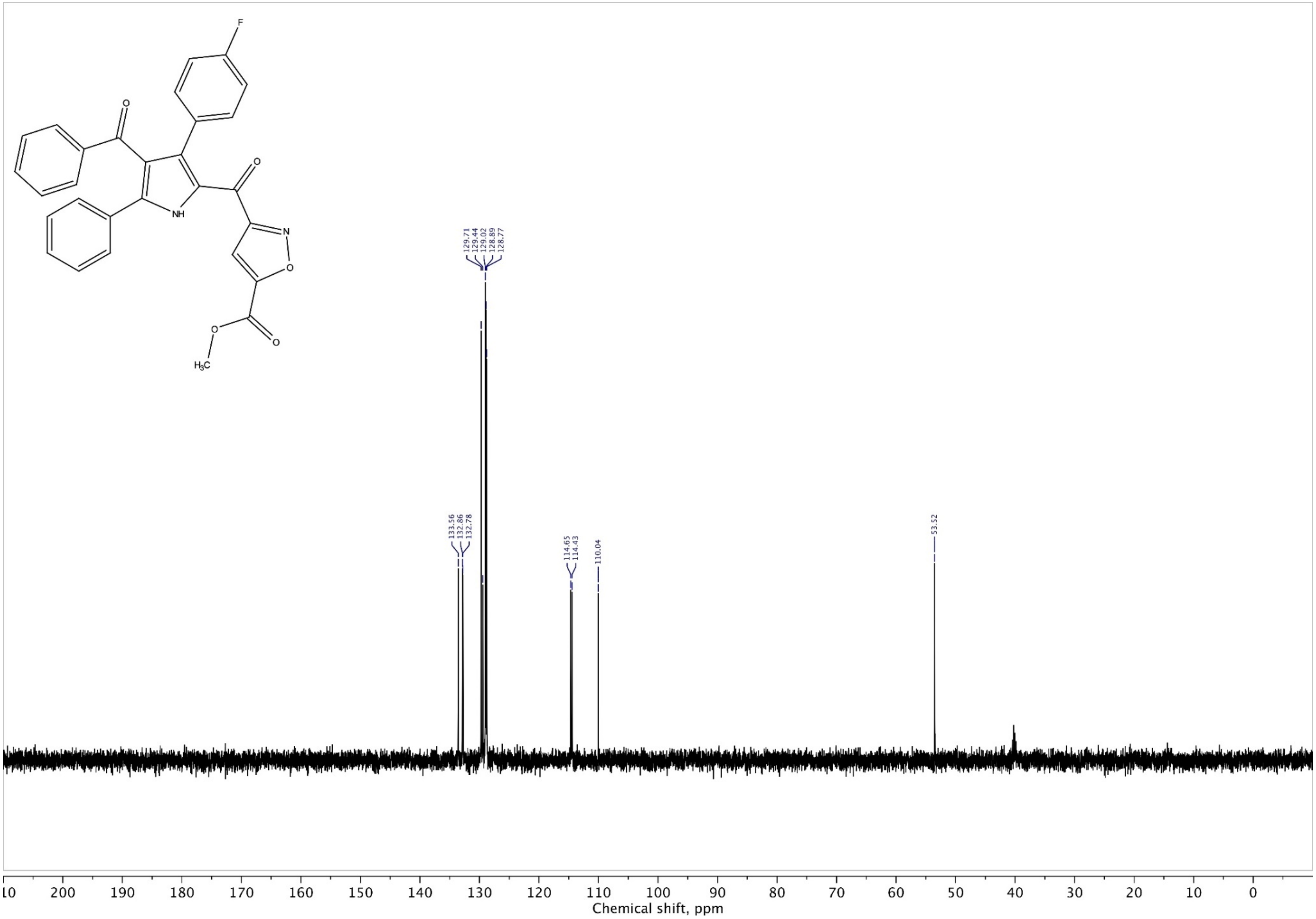

3-(2-phenyloxazol-5-yl)isoxazole-5-carboxylic acid (6a), ^{1}H NMR, DMSO-d_6, 400 MHz

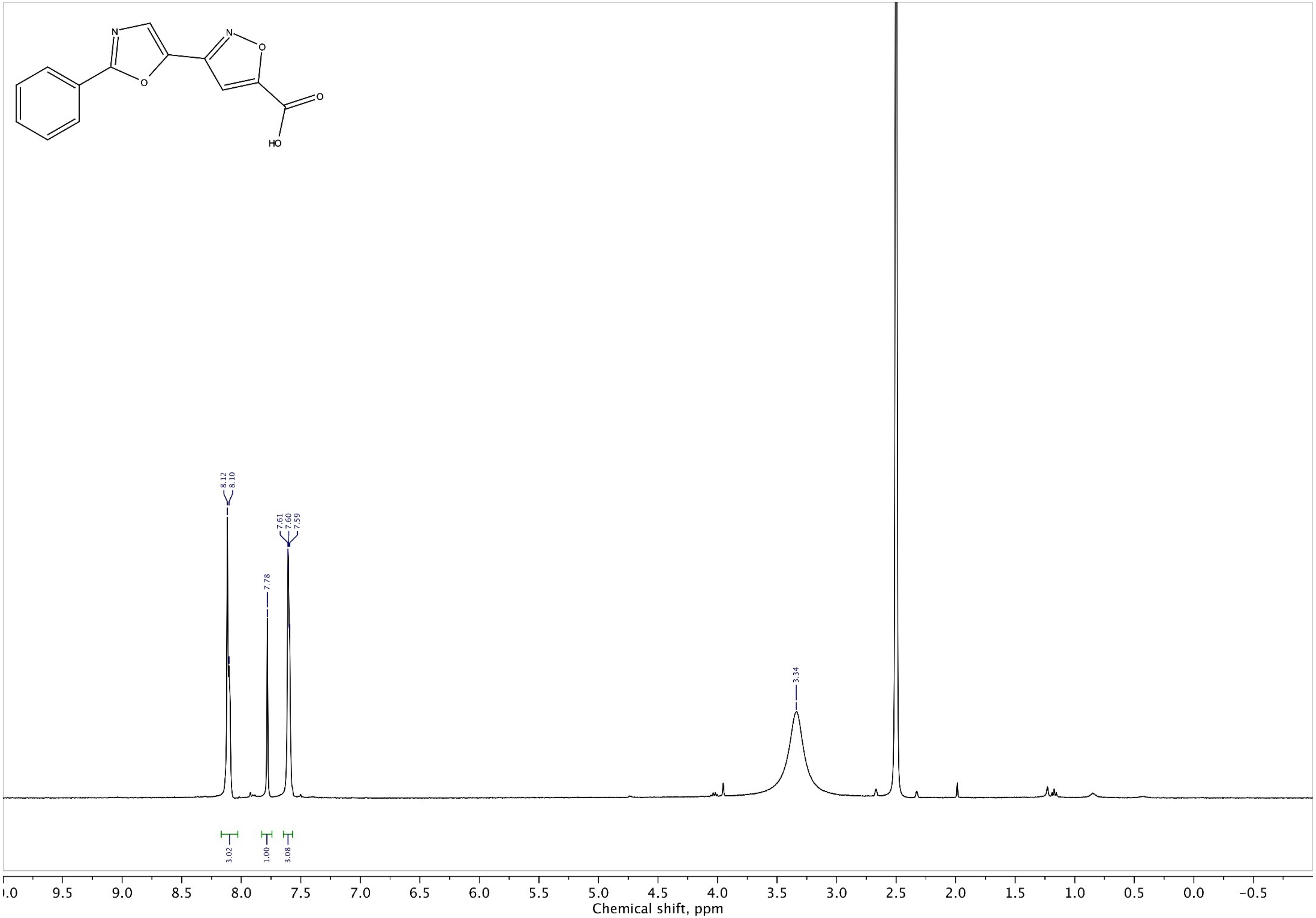

3-(2-phenyloxazol-5-yl)isoxazole-5-carboxylic acid (6a), $^{13}C\{^1H\}$ NMR, DMSO-d_6, 100 MHz

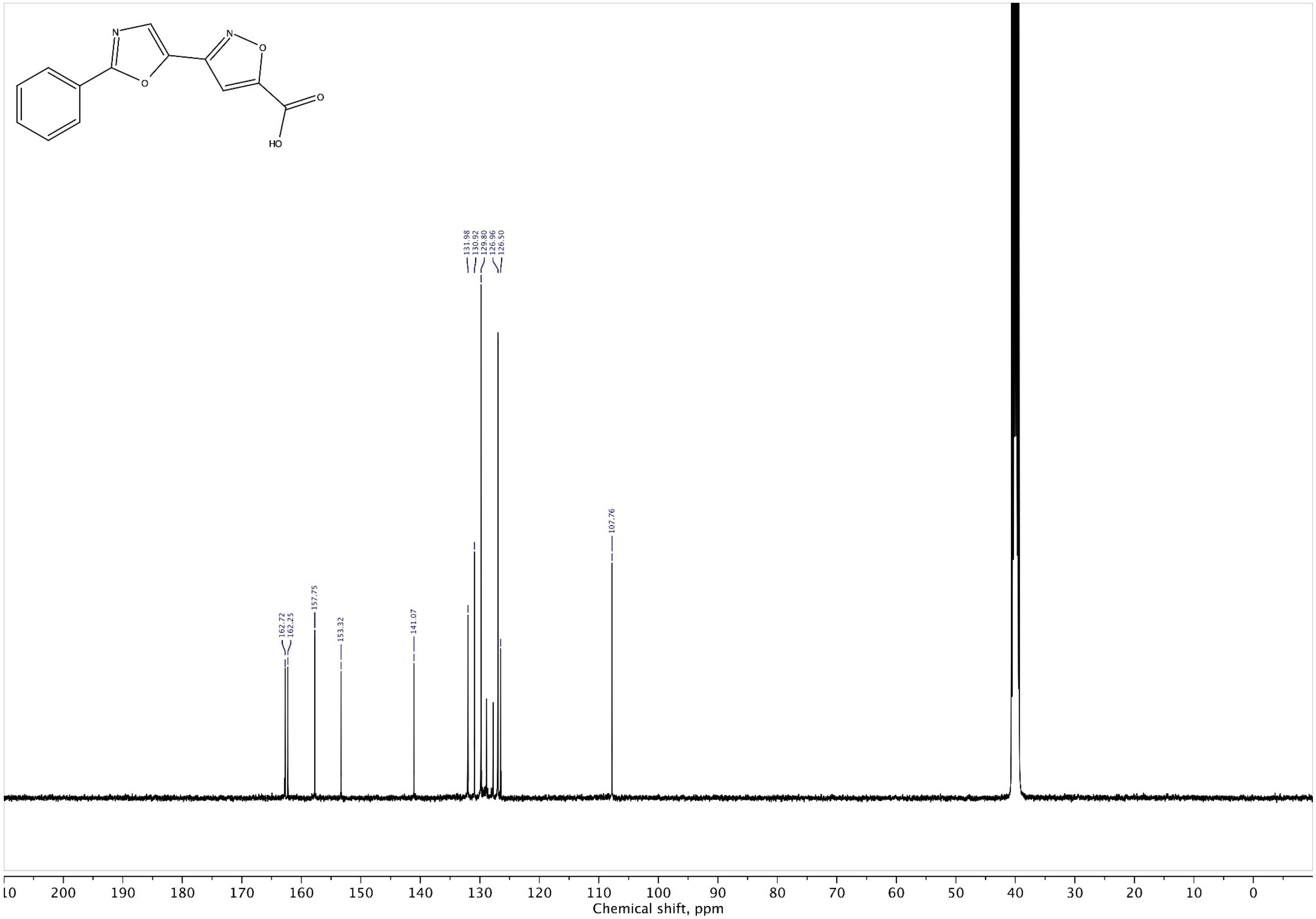

3-(2-phenyloxazol-5-yl)isoxazole-5-carboxylic acid (6a), DEPT, DMSO-d_6, 100 MHz

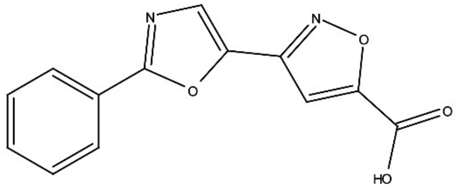

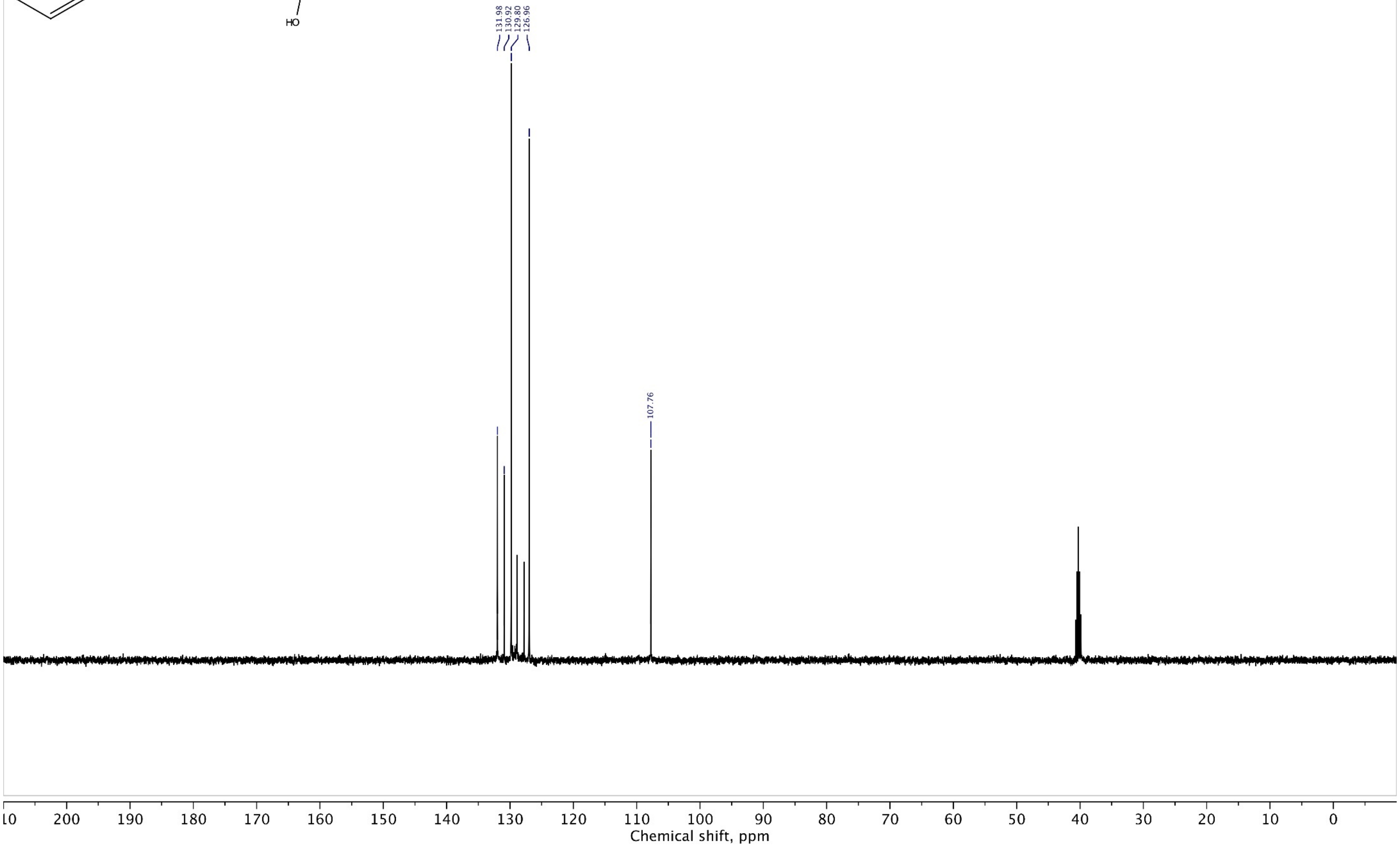

5-(Methoxymethyl)-3-(2-phenyloxazol-5-yl)isoxazole (6b), ^{1}H NMR, $CDCl_3$, 400 MHz

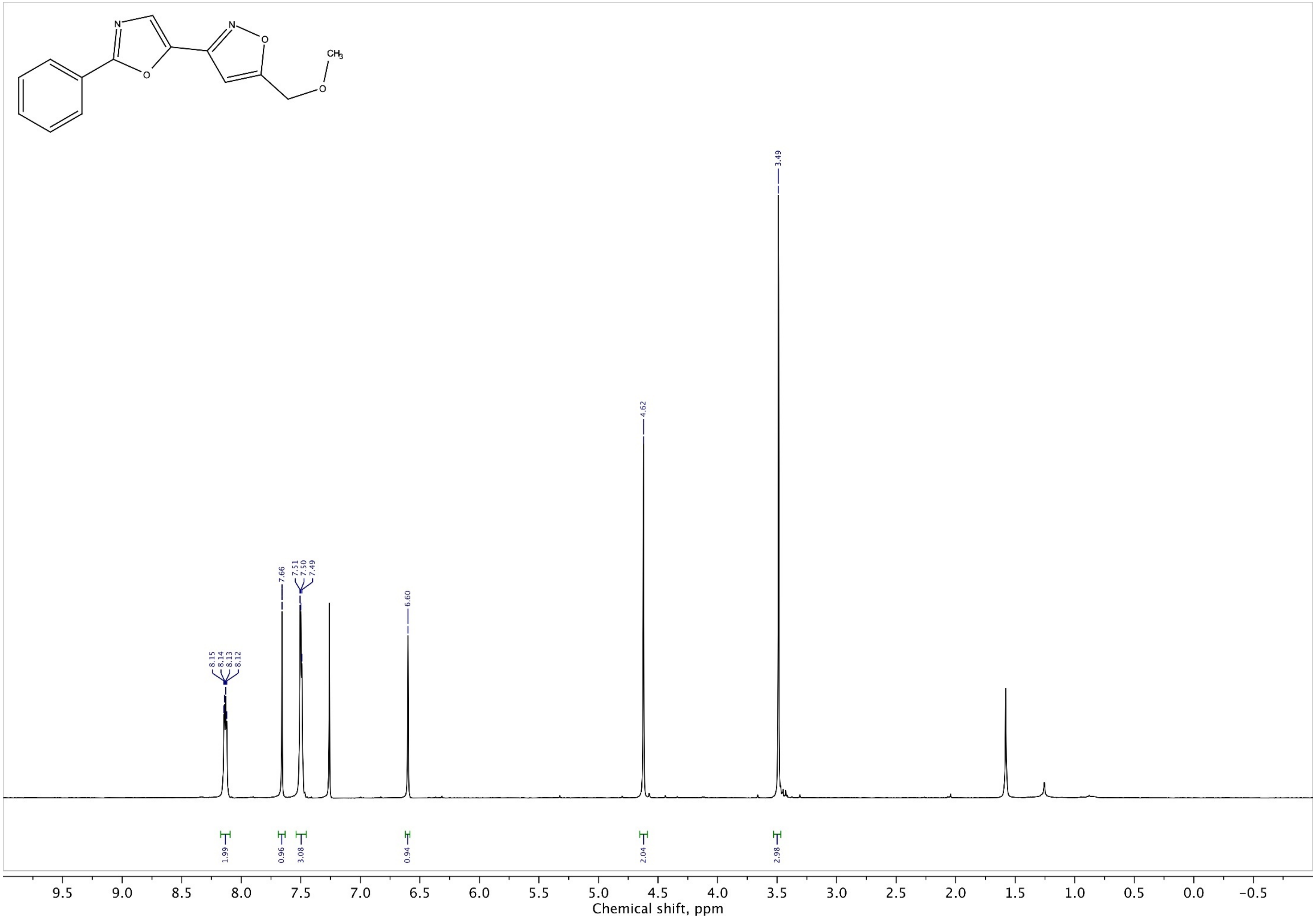

5-(Methoxymethyl)-3-(2-phenyloxazol-5-yl)isoxazole (6b), $^{13}C\{^1H\}$ NMR, $CDCl_3$, 100 MHz

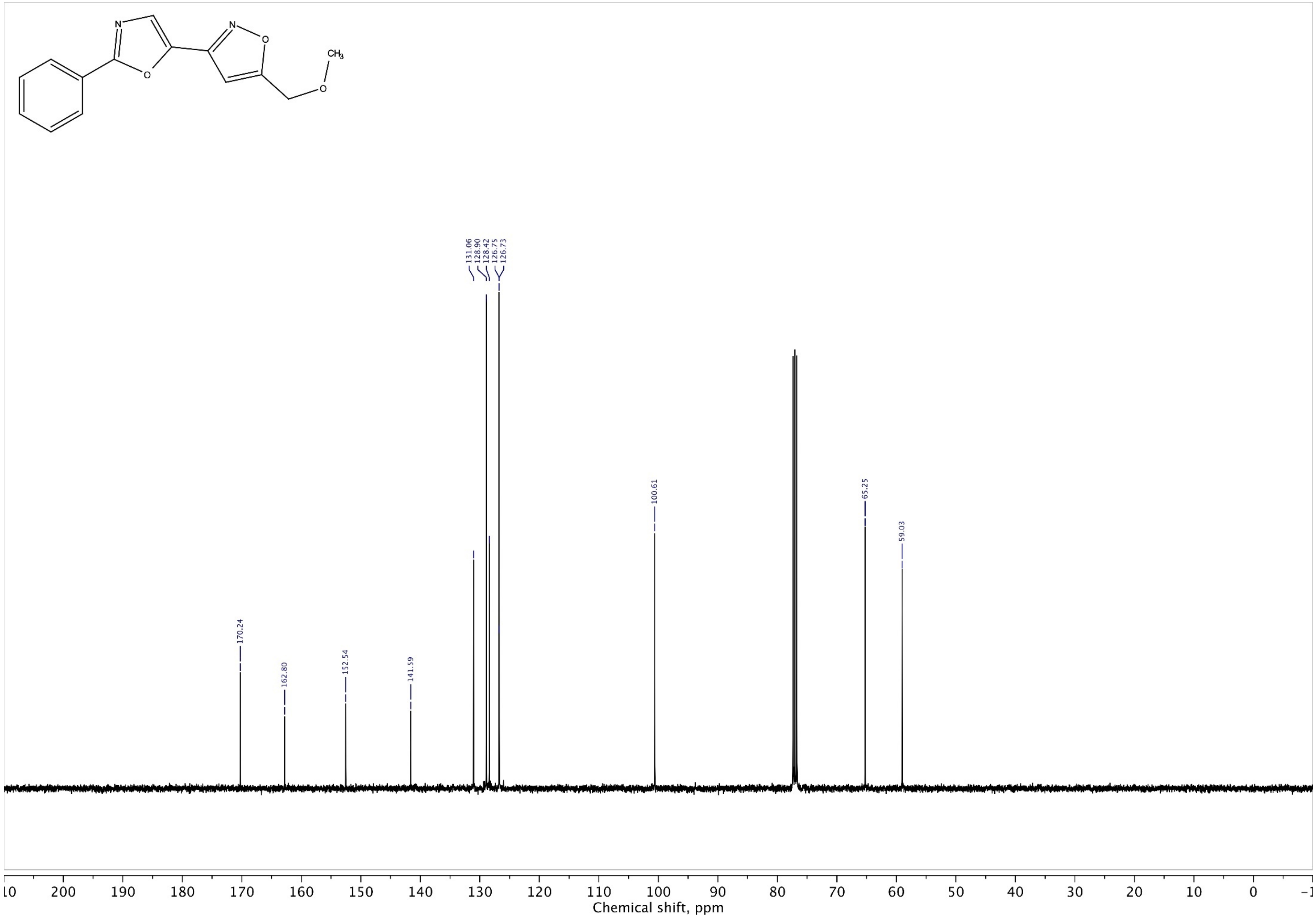

5-(Methoxymethyl)-3-(2-phenyloxazol-5-yl)isoxazole (6b), DEPT, $CDCl_3$, 100 MHz

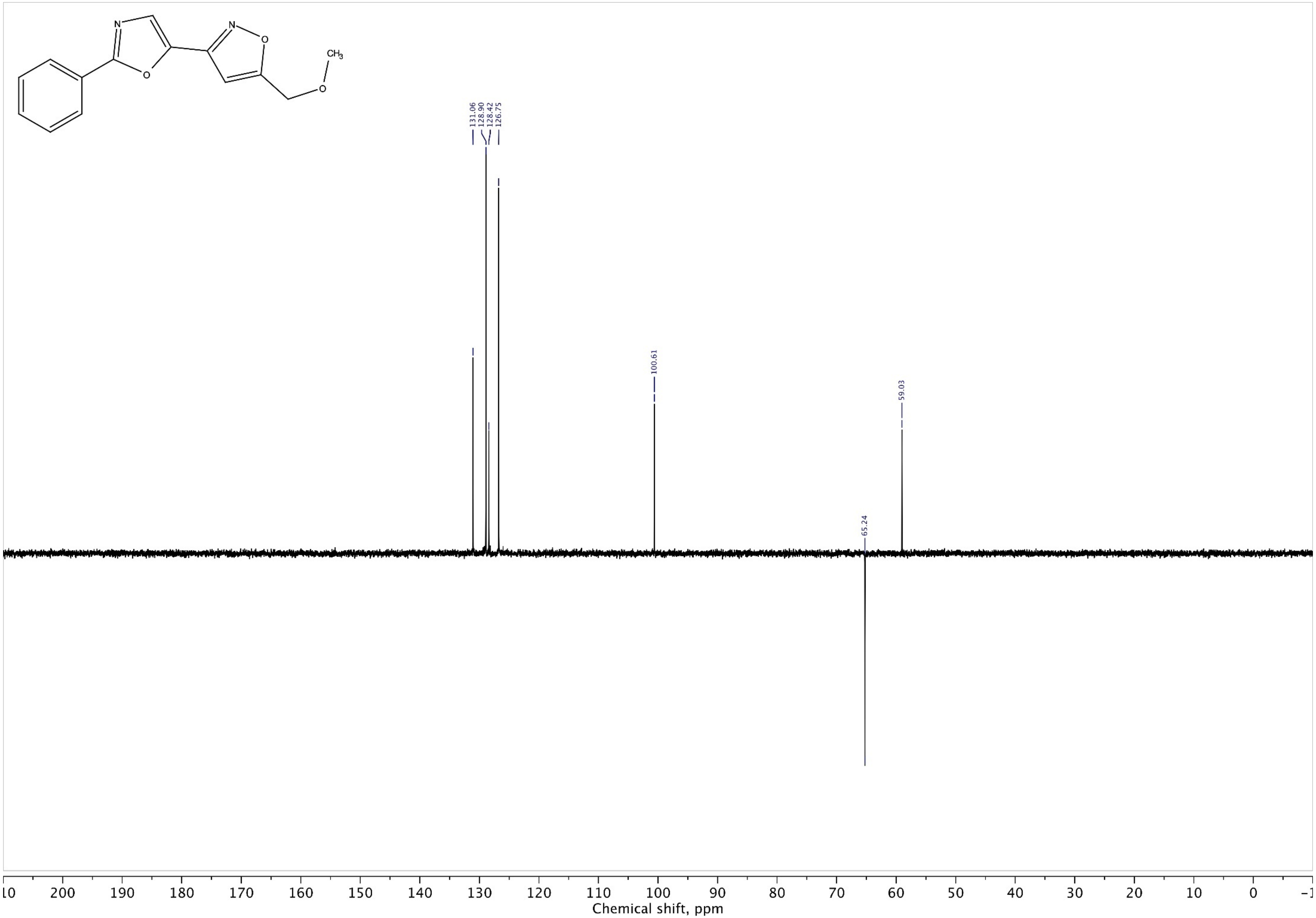

3-(2-Phenyloxazol-5-yl)isoxazole (6c), ^{1}H NMR, $CDCl_3$, 400 MHz

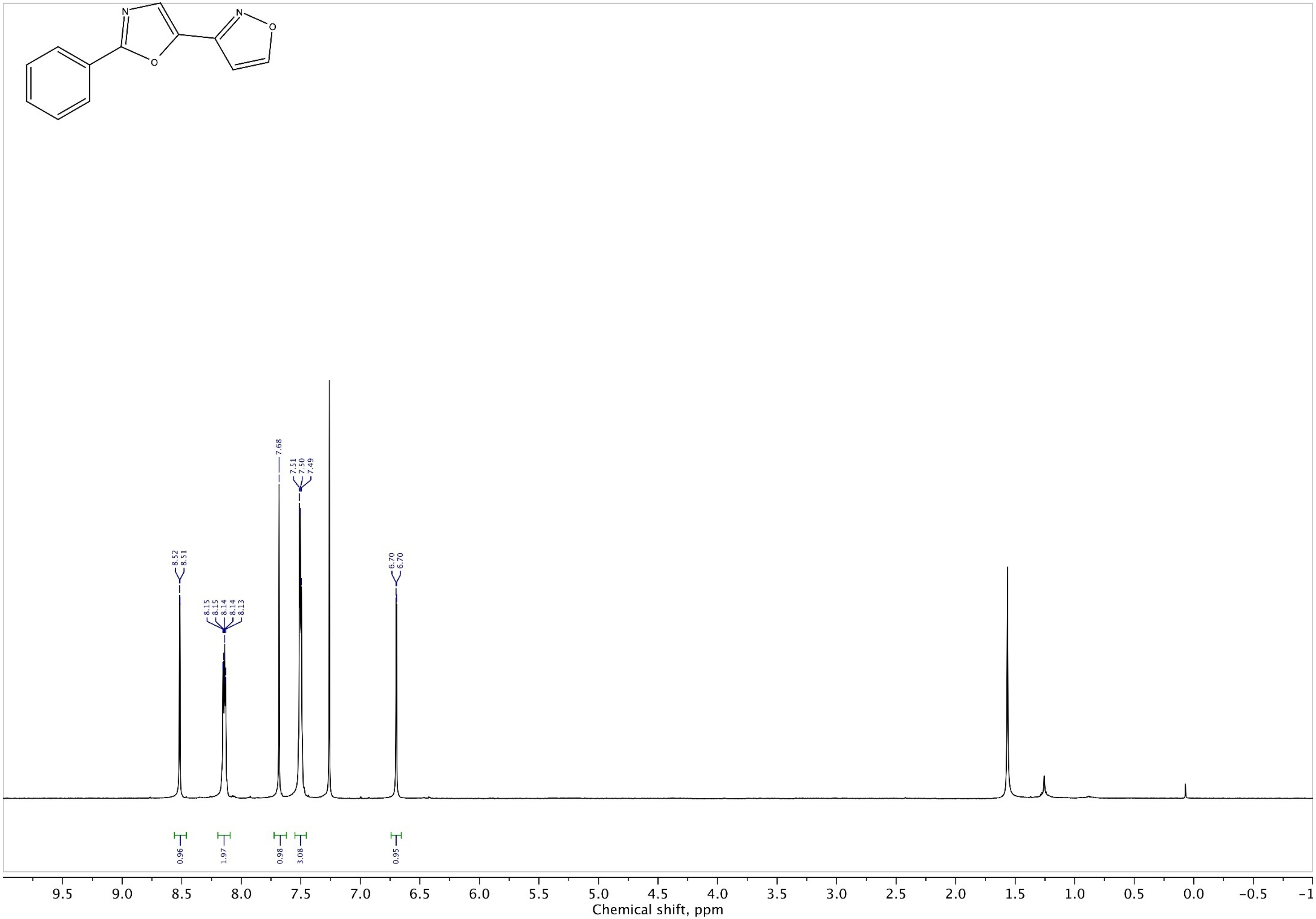

3-(2-Phenyloxazol-5-yl)isoxazole (6c), $^{13}C\{^1H\}$ NMR, $CDCl_3$, 100 MHz

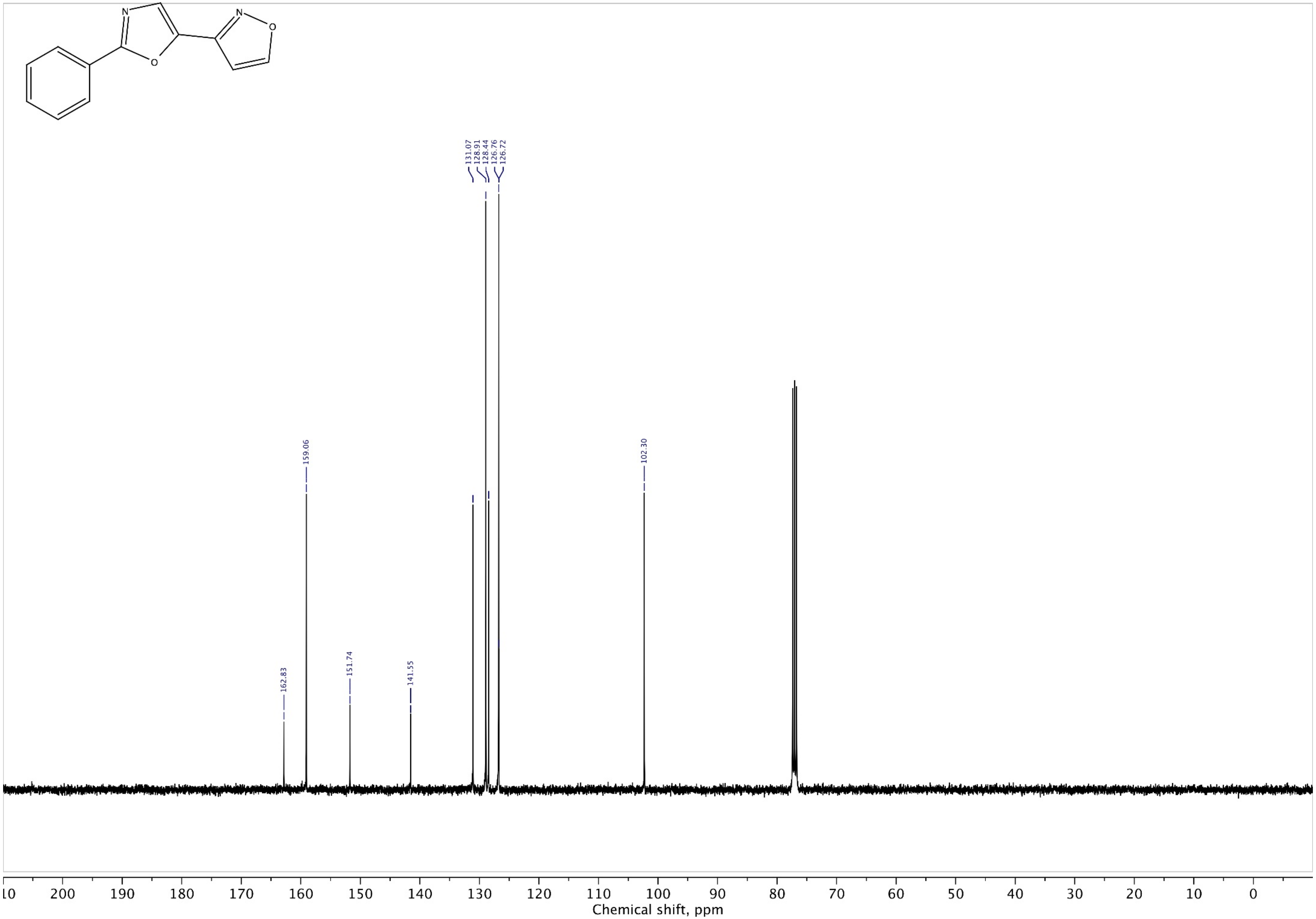

3-(2-Phenyloxazol-5-yl)isoxazole (6c), DEPT, $CDCl_3$, 100 MHz

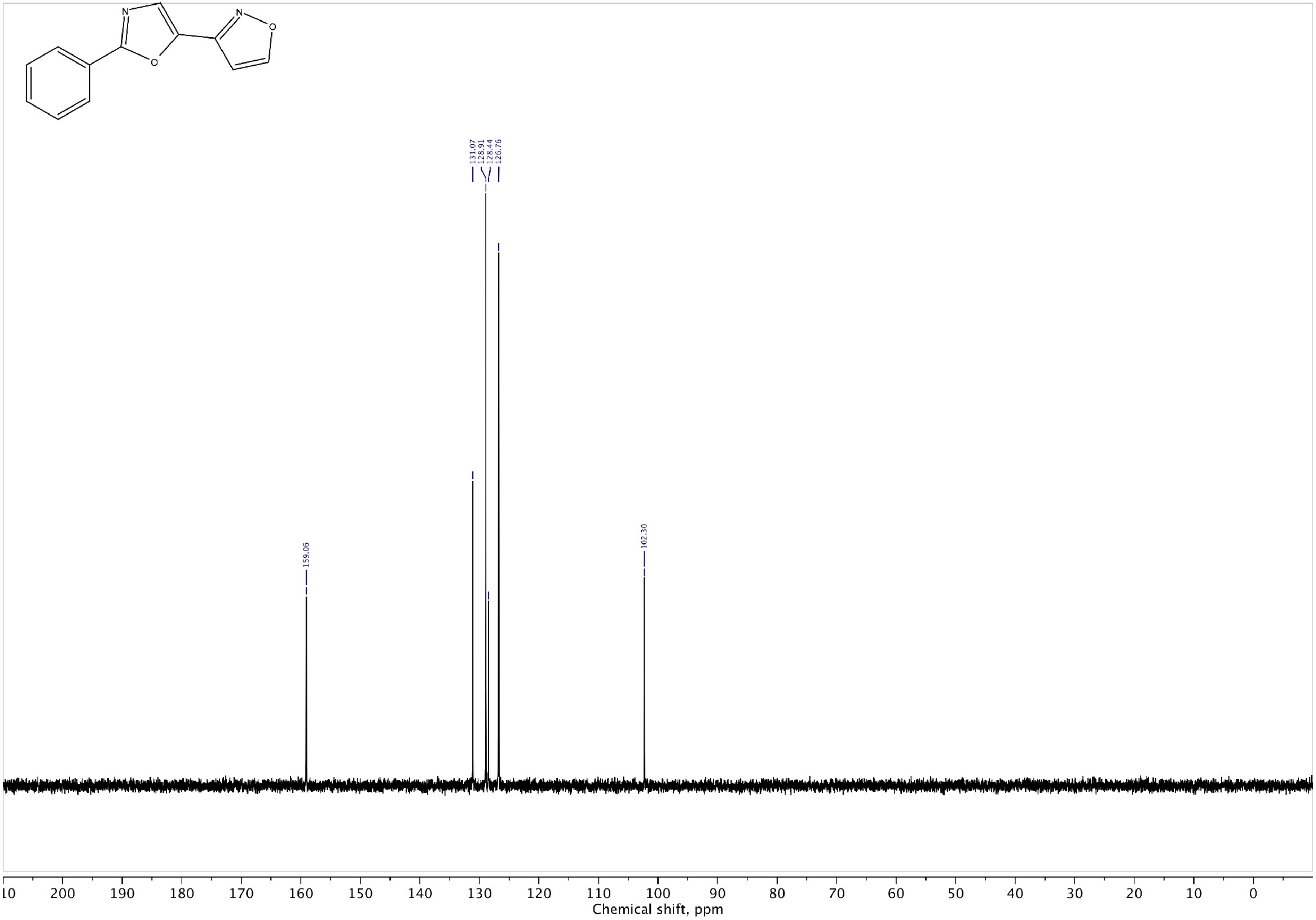

3-(2-(3,4-Dimethoxyphenyl)oxazol-5-yl)isoxazole (6d), ^{1}H NMR, $CDCl_3$, 400 MHz

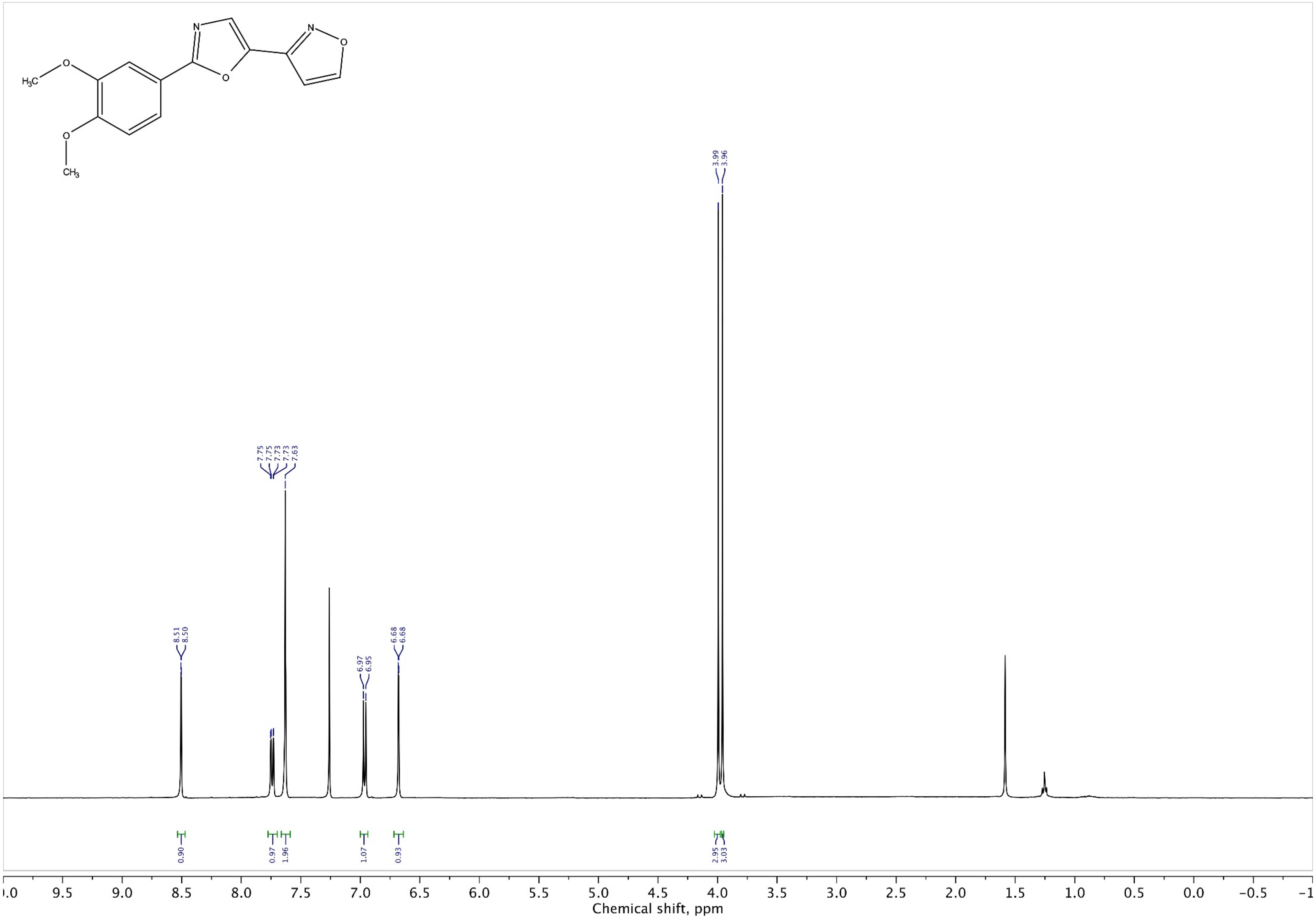

3-(2-(3,4-Dimethoxyphenyl)oxazol-5-yl)isoxazole (6d), $^{13}C\{^{1}H\}$ NMR, $CDCl_3$, 100 MHz

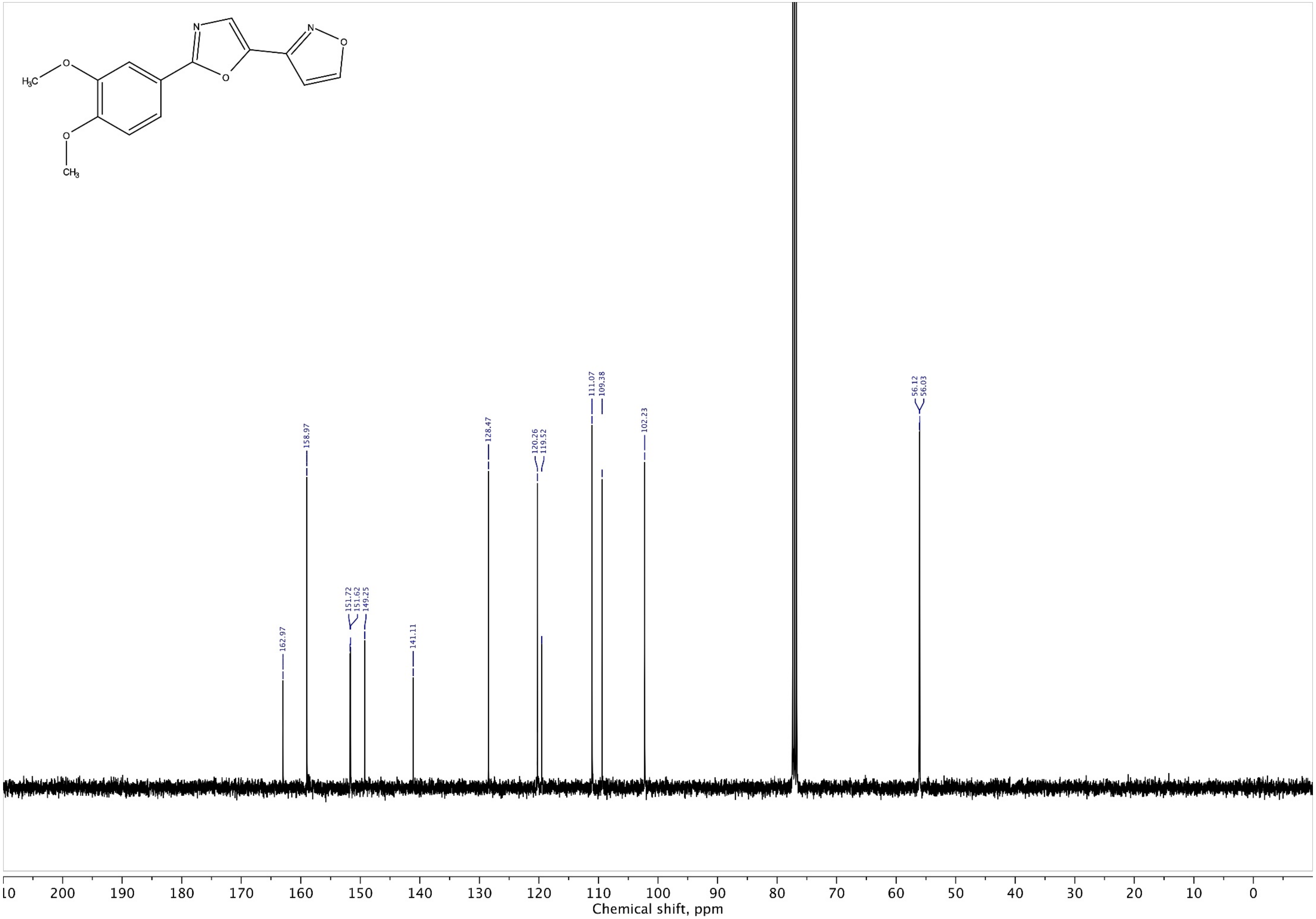

3-(2-(3,4-Dimethoxyphenyl)oxazol-5-yl)isoxazole (6d), DEPT, $CDCl_3$, 100 MHz

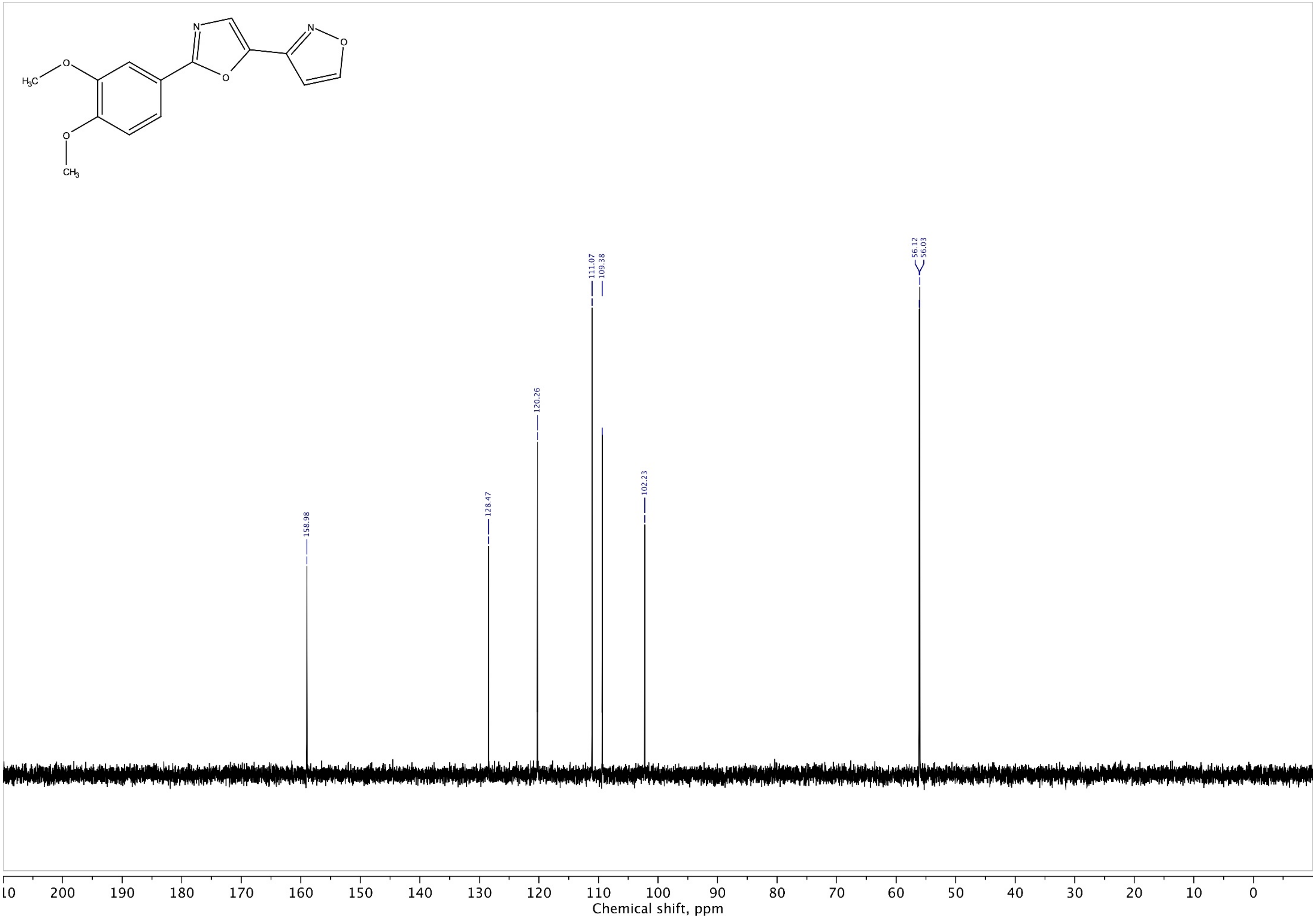

3-(2-(4-Chlorophenyl)oxazol-5-yl)-5-phenylisoxazole (6e), ^{1}H NMR, DMSO-d_6, 400 MHz

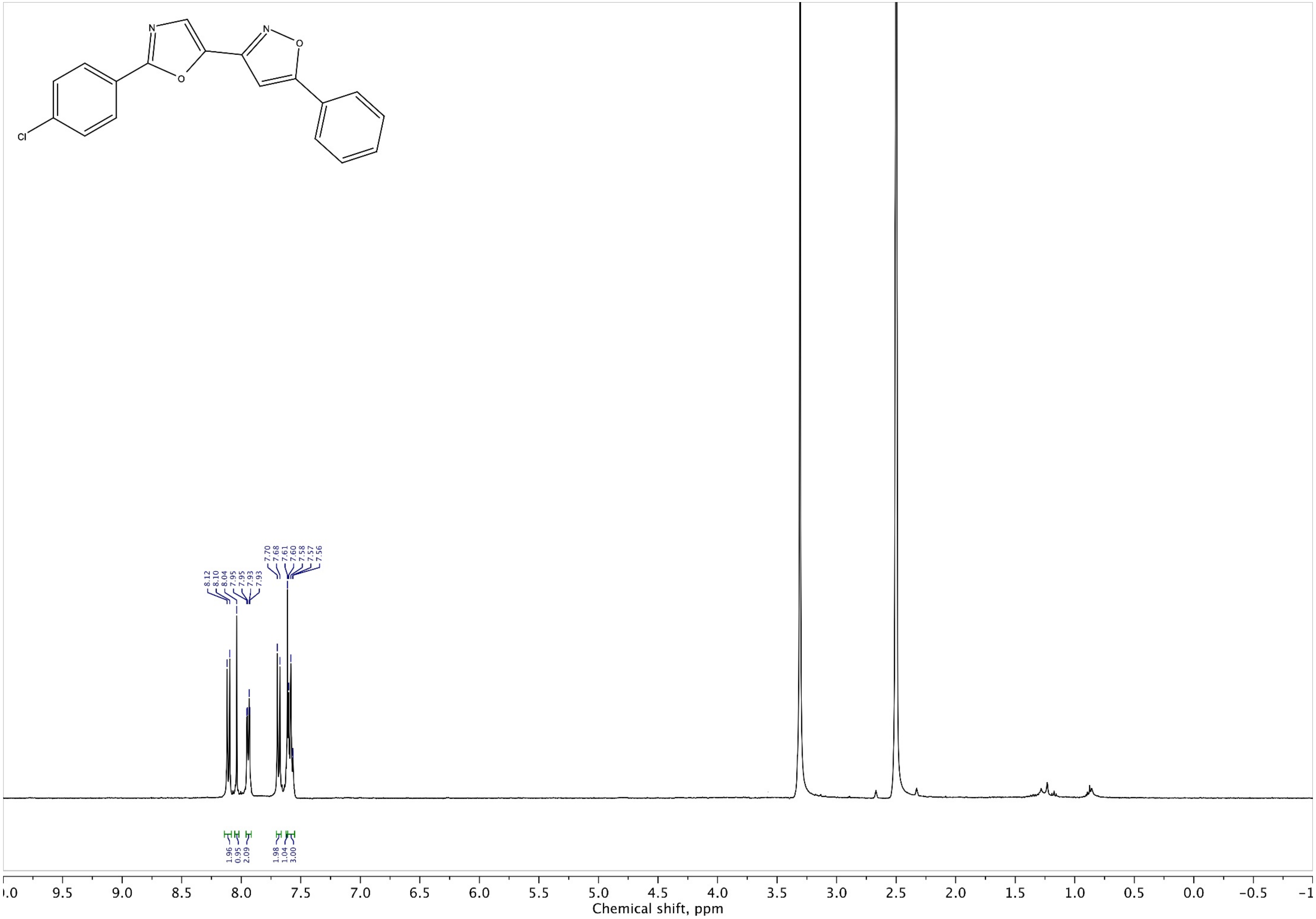

3-(2-(4-Chlorophenyl)oxazol-5-yl)-5-phenylisoxazole (6e), $^{13}C\{^1H\}$ NMR, DMSO-d_6, 100 MHz

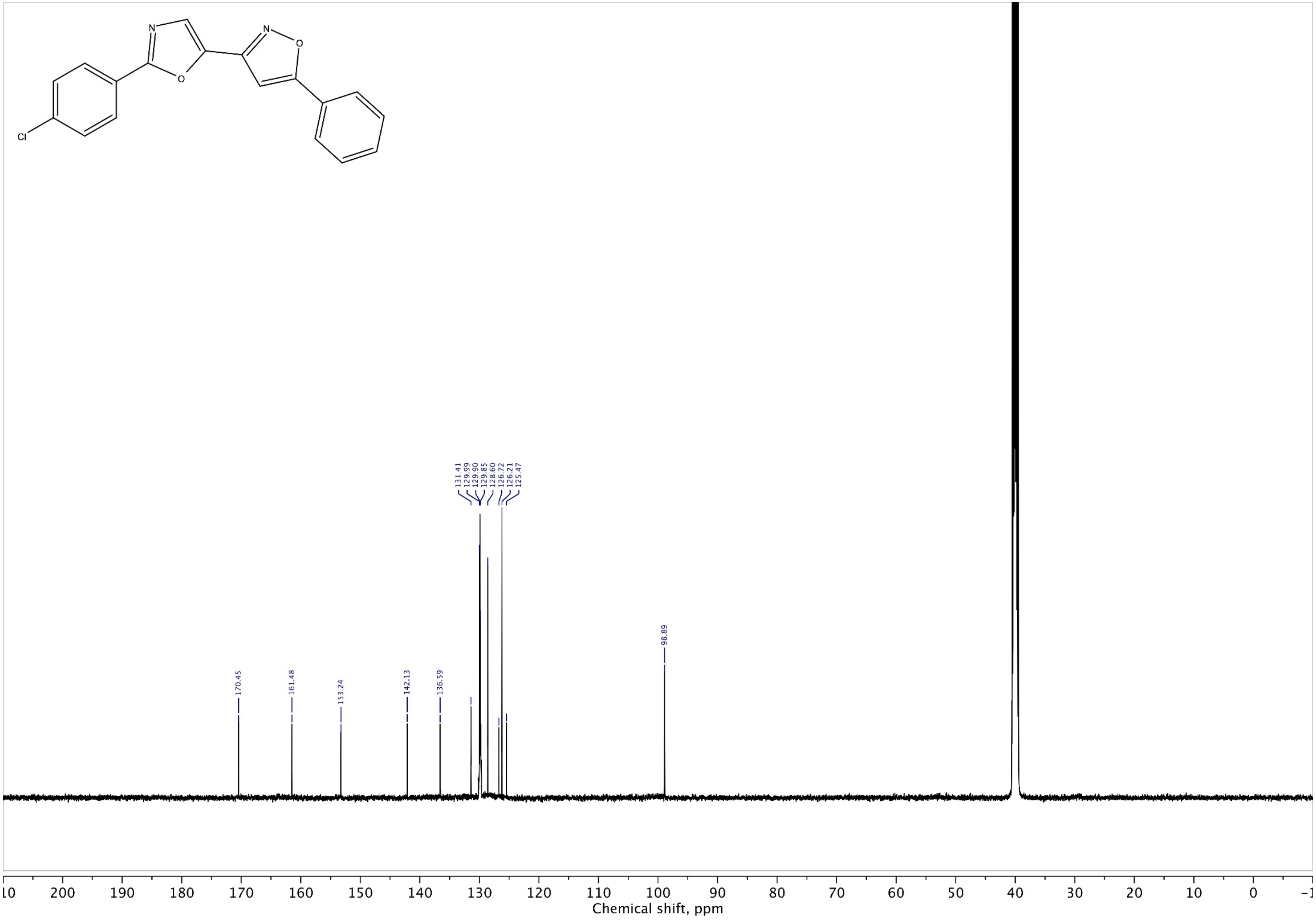

3-(2-(4-Chlorophenyl)oxazol-5-yl)-5-phenylisoxazole (6e), DEPT, DMSO-d_6, 100 MHz

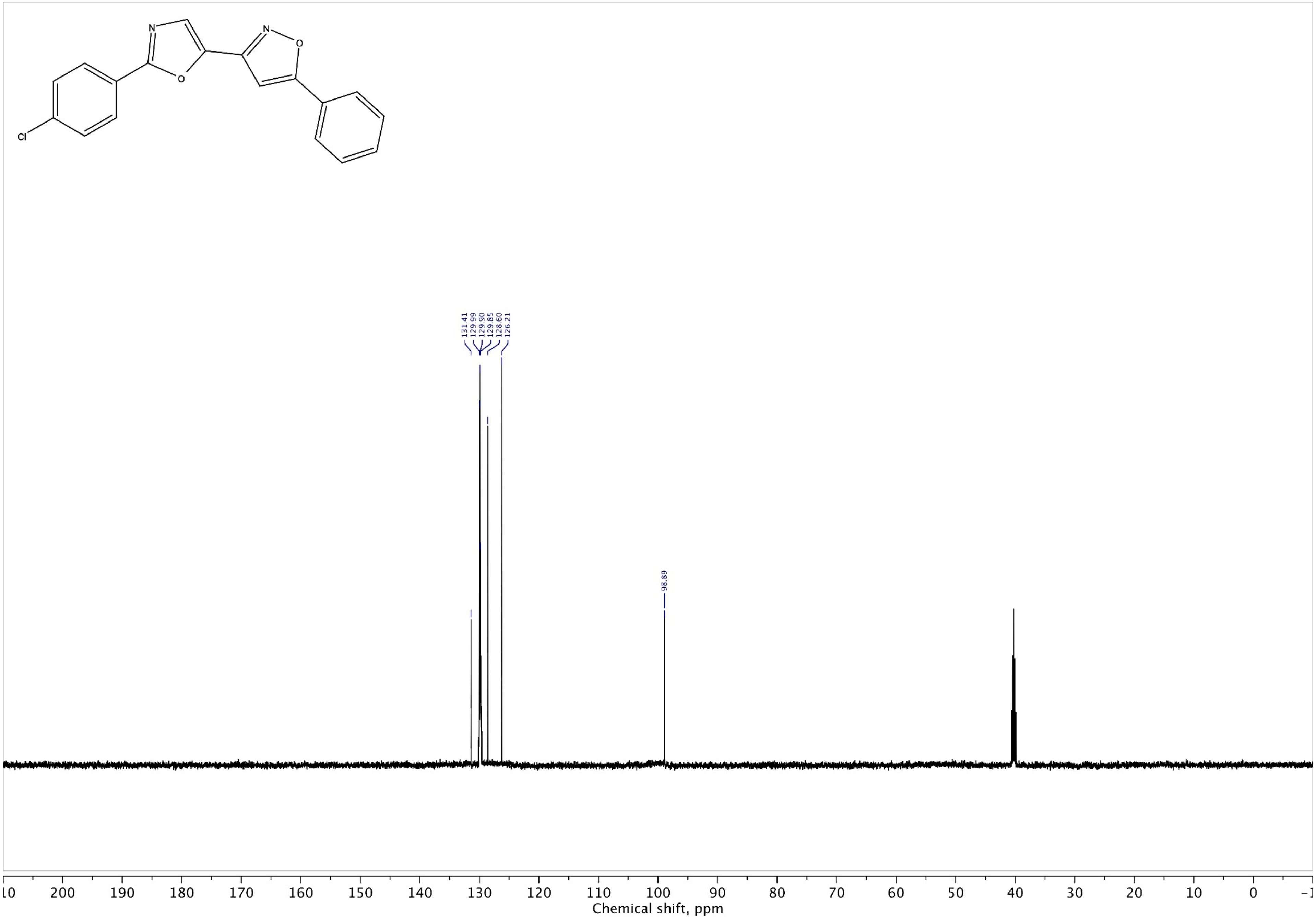

Computational Details

All calculations were performed by using the Gaussian 16 suite of quantum chemical programs[1] at Resource center "Computer center of Saint Petersburg State University". Geometry optimizations of molecules were performed with the B3LYP[2]-D3[3] density functional method and 6-311+G(d,p) basis set using SMD[4] solvent model. Stationary points on the respective potential-energy surfaces were characterized at the same level of theory by evaluating the corresponding Hessian indices. Careful verification of the unique imaginary frequencies for transition states was carried out to check whether the frequency indeed pertains to the desired reaction coordinate. Intrinsic reaction coordinates were calculated to authenticate all transition states.[5]

Table S15. B3LYP-D3/6-311+G(d,p), SMD solvent model.
Absolute Energies (au), Cartesian Coordinates of stationary points

Molecule **2a** (DCM)

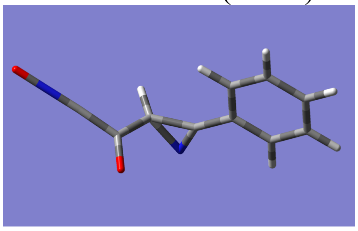

E = -644.672095, H (0K) = -644.533483,
H (298K) = -644.520458,
G (298K) = -644.574702 au.
Imaginary frequency = 0.

C	0.4376210	-0.9499590	-0.4741310
N	-0.0847460	-2.0814670	-0.5937690
C	-1.0068710	-0.8439570	-0.7165400
C	1.6642650	-0.2327170	-0.2374770
C	-1.9096570	-0.5961870	0.4312480
O	-1.6966060	-0.9565640	1.5734080
C	-3.1247520	0.1441160	0.1047370
N	-4.1013430	0.7423120	-0.0825620
O	-5.1151110	1.3626890	-0.2858720
C	1.6503170	1.1680480	-0.1969970
C	2.8350710	1.8610070	0.0315190
C	4.0266450	1.1590290	0.2196570
C	4.0403370	-0.2388490	0.1802950
C	2.8632340	-0.9397110	-0.0474740
H	-1.3847830	-0.6032760	-1.7049490
H	0.7164580	1.6987390	-0.3434250
H	2.8302790	2.9442120	0.0643320
H	4.9486580	1.7007900	0.3987390
H	4.9692480	-0.7770930	0.3288480
H	2.8592440	-2.0231960	-0.0785460

Molecule **7a** (DCM)

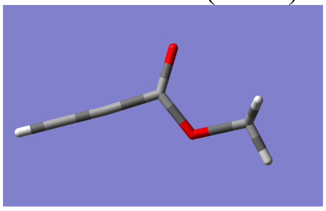

E = -305.310843, H (0K) = -305.239961,
H (298K) = -305.232635,
G (298K) = -305.269930 au.
Imaginary frequency = 0.

C	-2.6275190	-0.4183110	0.0001800
C	-1.4618930	-0.1236980	-0.0000970
O	0.7526350	-0.7405160	-0.0000660
H	-3.6614360	-0.6840390	0.0008810
C	2.1705670	-0.4302060	0.0002650
H	2.4304790	0.1385740	0.8940240
H	2.6722000	-1.3950100	0.0006640
H	2.4309750	0.1380970	-0.8936550
C	-0.0798120	0.3043310	-0.0004740
O	0.2623300	1.4667260	-0.0000790

Molecule **7g** (DCM)

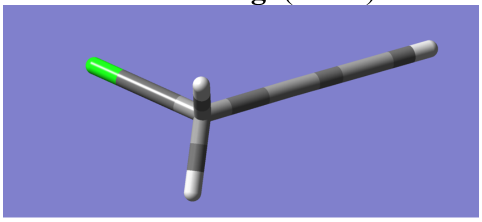

E = -576.320291, H (0K) = -576.273068,
H (298K) = -576.267553,
G (298K) = -576.300566 au.
Imaginary frequency = 0.

Molecule **7i** (DCM)

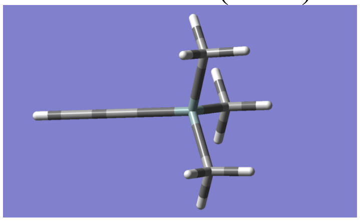

E = -486.127828, H (0K) = -485.998797,
H (298K) = -485.988085,
G (298K) = -486.032385 au.
Imaginary frequency = 0.

C	1.2944121	-0.8666062	-0.0905987
C	0.5819378	0.0943664	0.0338381
C	-0.2518917	1.2641615	0.1896626
H	1.9239976	-1.7202009	-0.2015128
H	0.0093998	2.0425136	-0.5262765
H	-0.1998652	1.6631894	1.2019599
Cl	-2.0296626	0.8758470	-0.1108181

C	-2.7555140	-0.0000260	-0.0001000
C	-1.5438330	-0.0000210	0.0001920
H	-3.8232650	0.0004170	-0.0003820
Si	0.3153610	-0.0000810	0.0001270
C	0.8919980	1.3286970	1.1967640
H	0.5305420	1.1316010	2.2108410
H	1.9866330	1.3611380	1.2274320
H	0.5323060	2.3168660	0.8933880
C	0.8919850	0.3723350	-1.7489180
H	1.9866330	0.3806760	-1.7926940
H	0.5307380	-0.3830330	-2.4537470
H	0.5323140	1.3501510	-2.0841950
C	0.8919450	-1.7009680	0.5520300
H	1.9865770	-1.7434310	0.5653570
H	0.5318360	-1.9332750	1.5591420
H	0.5311470	-2.4800840	-0.1267280

Molecule **7k** (DCM)

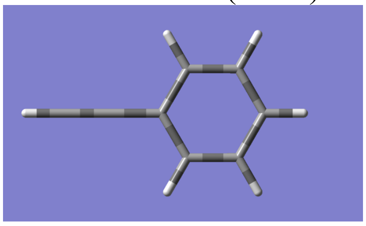

E = -308.496278, H (0K) = -308.387214,
H (298K) = -308.379848,
G (298K) = -308.417640 au.
Imaginary frequency = 0.

C	-3.2279100	0.0004170	-0.0001070
C	-2.0215230	-0.0007370	-0.0001650
H	-4.2940620	0.0001840	0.0007810
C	-0.5922280	-0.0003370	-0.0000540
C	0.1188480	-1.2135040	-0.0000230
C	0.1182170	1.2131940	-0.0000240
C	1.5105490	-1.2079410	0.0000560
H	-0.4254030	-2.1505880	-0.0000590
C	1.5099250	1.2083570	0.0000550
H	-0.4265160	2.1499980	-0.0000610
C	2.2095680	0.0003910	0.0000980
H	2.0502730	-2.1483020	0.0000840
H	2.0491590	2.1489990	0.0000820
H	3.2938740	0.0006720	0.0001540

Molecule **7l** (DCM)

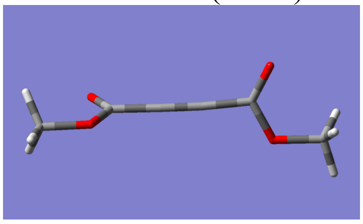

E = -533.259898, H (0K) = -533.145856,
H (298K) = -533.133895,
G (298K) = -533.184491 au.
Imaginary frequency = 0.

C	-0.5992550	0.1897390	-0.0590150
C	0.5992560	0.1897060	0.0589820
O	2.6733960	-0.6180580	-0.5037390
C	4.1216750	-0.6403870	-0.3803950
H	4.4057440	-0.9040430	0.6389990
H	4.4528510	-1.4021880	-1.0818490
H	4.5339170	0.3339360	-0.6456800
C	2.0301700	0.2529810	0.2712940
O	2.5445960	1.0128440	1.0607260
C	-2.0301790	0.2530320	-0.2712630
O	-2.5446340	1.0129410	-1.0606300
O	-2.6733760	-0.6180840	0.5037120
C	-4.1216530	-0.6404570	0.3803370
H	-4.5339470	0.3337660	0.6459160
H	-4.4056970	-0.9038080	-0.6391410
H	-4.4528000	-1.4024900	1.0815530

TS 2a+7a→3a (DCM)

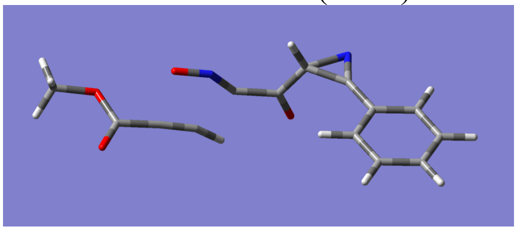

E = -949.967644, H (0K) = -949.757677,
H (298K) = -949.737635,
G (298K) = -949.809177 au.
Imaginary frequency = 1.

C	2.7483240	-1.0886170	-0.4005500

Molecule **3a** (DCM)

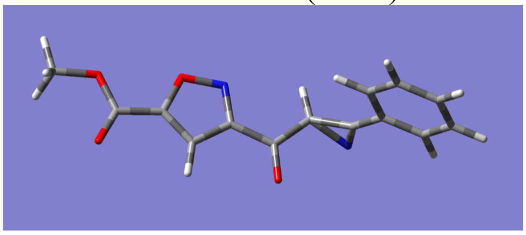

E = -950.104078, H (0K) = -949.887277,
H (298K) = -949.868842,
G (298K) = -949.936835 au.
Imaginary frequency = 0.

C	2.6913530	-1.0085340	-0.4763560

N	2.4578400	-2.3079870	-0.4018210
C	1.3332320	-1.2987950	-0.7174200
C	3.7962100	-0.1205280	-0.2033450
C	0.3261220	-1.0965480	0.3599520
O	0.5353470	-1.3012310	1.5404600
C	-0.9813020	-0.6066830	-0.0979970
N	-1.5686300	-0.2886590	-1.1044820
O	-2.6186310	0.1240680	-1.5407360
C	-2.3897730	-0.2890030	1.4171920
C	-3.4353180	0.1326360	0.9397600
C	3.5046620	1.2448910	-0.3271660
C	4.5142580	2.1836820	-0.1393380
C	5.8078190	1.7613240	0.1710130
C	6.0985320	0.3990710	0.2960200
C	5.0977330	-0.5458500	0.1106850
O	-5.5204000	-0.2473700	0.0501410
H	0.9807650	-1.2482770	-1.7421000
H	-1.7196910	-0.6146610	2.1915230
H	2.4947710	1.5571020	-0.5675760
H	4.2942260	3.2406380	-0.2334070
H	6.5934470	2.4942430	0.3168610
H	7.1052950	0.0791450	0.5383960
H	5.3095850	-1.6046230	0.2057910
C	-6.7854240	0.2162970	-0.4849030
H	-6.6167560	0.8311850	-1.3703510
H	-7.3321050	-0.6871210	-0.7458560
H	-7.3285530	0.7886140	0.2686380
C	-4.6601160	0.7029470	0.4337620
O	-4.8782170	1.8959500	0.3921600

N	2.4167920	-2.2249960	-0.5858240
C	1.2690860	-1.1977430	-0.7806530
C	3.7375270	-0.0549020	-0.2040850
C	0.2835570	-1.1147200	0.3250510
O	0.5472760	-1.4060360	1.4786160
C	-1.0950940	-0.6424880	-0.0105450
N	-1.4369770	-0.3075560	-1.2384090
O	-2.7578520	0.0770050	-1.1775620
C	-2.1815480	-0.4874490	0.8936330
C	-3.1889970	-0.0362890	0.1029350
C	3.4360220	1.3134390	-0.1858550
C	4.4429110	2.2370350	0.0785210
C	5.7443470	1.7965230	0.3236440
C	6.0454450	0.4309510	0.3059620
C	5.0470240	-0.4984070	0.0437240
O	-5.2767560	0.7211960	-0.6197650
H	0.9075000	-1.0598730	-1.7927460
H	-2.1987100	-0.6834350	1.9518780
H	2.4201710	1.6399170	-0.3773490
H	4.2146070	3.2963390	0.0944220
H	6.5277730	2.5173310	0.5293510
H	7.0582630	0.0960220	0.4978180
H	5.2671600	-1.5596690	0.0289570
C	-6.6603240	1.0931010	-0.3860650
H	-6.7093770	1.9327930	0.3084320
H	-7.0463960	1.3789310	-1.3615280
H	-7.2144070	0.2427610	0.0134820
C	-4.5864090	0.3183260	0.4431650
O	-5.0120030	0.2370460	1.5735200

TS 2a+7a→3'a (DCM)

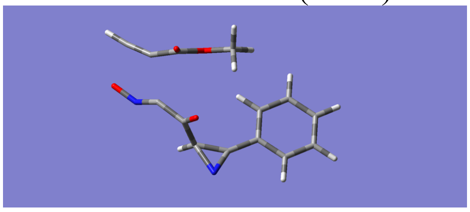

E = -949.963980, H (0K) = -949.753622,
H (298K) = -949.733846,
G (298K) = -949.803975 au.
Imaginary frequency = 1.

C	-1.7263940	-1.4543980	-0.5849390
N	-1.4191140	-2.2057930	-1.5399650
C	-0.3636170	-1.9720430	-0.4380330
C	-2.7334170	-0.5910630	-0.0247360
C	0.7555520	-1.0551420	-0.7867750
O	0.7004120	-0.2168380	-1.6649350
C	1.9495000	-1.1842170	0.0616820
N	2.5281660	-1.9858910	0.7568740
O	3.5327080	-2.1017400	1.4358300
C	3.3015420	0.5207080	0.3904880
C	4.2090990	0.1092580	1.1078570
C	-2.4451260	0.1272210	1.1439820
C	-3.4045080	0.9789870	1.6822300
C	-4.6457520	1.1111240	1.0575720
C	-4.9337250	0.3936430	-0.1078300
C	-3.9816800	-0.4573590	-0.6543640

Molecule **3'a**

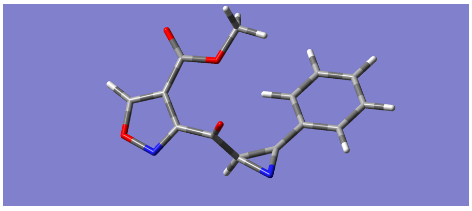

E = -950.102882, H (0K) = -949.886003,
H (298K) = -949.867645,
G (298K) = -949.934069 au.
Imaginary frequency = 0.

C	1.4767230	-1.6873220	-0.0564150
N	1.1905850	-2.8232570	0.3847540
C	0.0577960	-1.9950900	-0.2715950
C	2.5105160	-0.6982750	-0.2257580
C	-0.9418320	-1.3976450	0.6416260
O	-0.7909040	-1.2704130	1.8383740
C	-2.2125490	-0.9025000	-0.0103960
N	-3.0684540	-1.7603560	-0.4971830
O	-4.1531340	-0.9888820	-0.9601530
C	-2.6798340	0.4450700	-0.1401600
C	-3.8961170	0.2992380	-0.7394220
C	2.1936650	0.5263480	-0.8289390
C	3.1839360	1.4898580	-0.9920340
C	4.4839610	1.2321530	-0.5546470
C	4.8000900	0.0104850	0.0485060
C	3.8184420	-0.9578330	0.2158070

H	-0.1500080	-2.7949080	0.2356570
H	-1.4750330	0.0148680	1.6146560
H	-3.1862500	1.5384010	2.5844050
H	-5.3924800	1.7754150	1.4780660
H	-5.8995270	0.5025250	-0.5874160
H	-4.1904940	-1.0164280	-1.5592000
C	2.6336210	1.5986850	-0.3441320
O	3.0965760	2.1341660	-1.3230520
O	1.4693700	1.9091890	0.2317060
C	0.6673410	2.9085460	-0.4457820
H	1.1917440	3.8648390	-0.4678540
H	0.4444850	2.5768310	-1.4602250
H	-0.2456780	2.9860710	0.1404100
H	5.0527320	-0.1077640	1.7234310

H	-0.2866040	-2.3091760	-1.2513790
H	1.1763630	0.7142910	-1.1505680
H	2.9431290	2.4396520	-1.4551230
H	5.2546640	1.9842410	-0.6816390
H	5.8115490	-0.1817490	0.3871590
H	4.0499670	-1.9077340	0.6838080
C	-2.0323130	1.7214450	0.2048260
O	-2.5855080	2.7996540	0.1597880
O	-0.7539230	1.5362320	0.5538760
C	-0.0048190	2.7101430	0.9482510
H	0.0997230	3.3919690	0.1027470
H	-0.5058270	3.2157590	1.7747070
H	0.9672530	2.3377020	1.2615530
H	-4.6433620	1.0111420	-1.0572350

TS 2a+7g→3g (DCM)

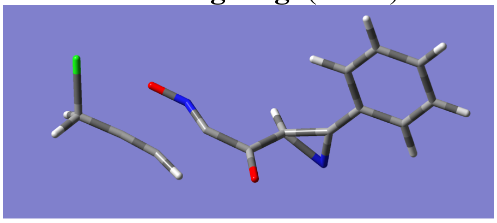

E = -1220.974397, H (0K) = -1220.787914,
H (298K) = -1220.769803,
G (298K) = -1220.837275 au.
Imaginary frequency = 1.

C	2.4501840	-1.0873870	-0.4140980
N	2.2676870	-2.3276530	-0.4374670
C	1.0671560	-1.4142580	-0.7664960
C	3.4071890	-0.0373360	-0.1758570
C	0.0198890	-1.3116840	0.2892800
O	0.2240820	-1.5224740	1.4717900
C	-1.3033830	-0.9137780	-0.1894970
N	-1.9000860	-0.6428370	-1.2067850
O	-2.9941750	-0.3102580	-1.6214160
C	-2.7750050	-0.7054590	1.3405630
C	-3.8481610	-0.3509550	0.8721030
C	-5.1790710	0.0459640	0.4716670
C	3.0027420	1.3002540	-0.2839950
C	3.9233290	2.3181830	-0.0550480
C	5.2406830	2.0021140	0.2805760
C	5.6441910	0.6674930	0.3897390
C	4.7325450	-0.3556670	0.1635410
H	0.7349200	-1.3797550	-1.7984850
H	-2.0758240	-0.9864970	2.1048900
H	-5.8705060	-0.0423220	1.3114010
H	-5.5478370	-0.5371840	-0.3691380
H	1.9756250	1.5293460	-0.5442290
H	3.6156440	3.3540750	-0.1367810
H	5.9567910	2.7966080	0.4583840
H	6.6687020	0.4299970	0.6517710
H	5.0316830	-1.3942010	0.2462950
Cl	-5.2467870	1.8082540	-0.0442470

Molecule **3g** (DCM)

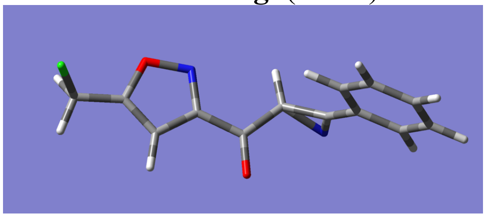

E = -1221.114094, H (0K) = -1220.920491,
H (298K) = -1220.904056,
G (298K) = -1220.967691 au.
Imaginary frequency = 0.

C	2.3676870	-1.0830060	-0.3617190
N	2.1648210	-2.3184640	-0.3490840
C	0.9631800	-1.3848490	-0.6557590
C	3.3546090	-0.0482440	-0.1792490
C	-0.0408540	-1.2444120	0.4282300
O	0.2230250	-1.4130820	1.6066030
C	-1.4327980	-0.8683650	0.0373780
N	-1.7785320	-0.7052200	-1.2227930
O	-3.1188050	-0.3716750	-1.2050670
C	-2.5295010	-0.6424230	0.9155920
C	-3.5543830	-0.3361430	0.0798140
C	-4.9741190	-0.0015130	0.3031040
C	2.9763250	1.2953210	-0.3031280
C	3.9257410	2.2972360	-0.1255240
C	5.2462930	1.9593480	0.1741270
C	5.6241280	0.6185250	0.2978830
C	4.6834300	-0.3883880	0.1229930
H	0.6083130	-1.3692280	-1.6793850
H	-2.5408220	-0.7021920	1.9904850
H	-5.2693860	-0.2578730	1.3169430
H	-5.6224690	-0.4903040	-0.4210140
H	1.9464570	1.5417960	-0.5352070
H	3.6378440	3.3378430	-0.2192230
H	5.9846910	2.7412540	0.3124310
H	6.6513710	0.3634570	0.5313370
H	4.9628540	-1.4314340	0.2177480
Cl	-5.2679450	1.8042360	0.1102530

TS 2a+7g→3’g (DCM)

Molecule **3’g** (DCM)

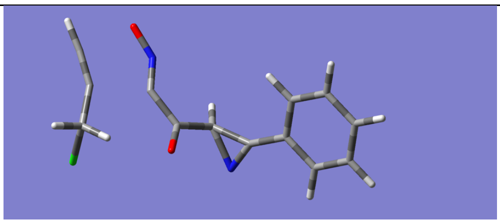

E = -1220.968911, H (0K) = -1220.781855,
H (298K) = -1220.763990,
G (298K) = -1220.831035 au.
Imaginary frequency = 0.

C	-1.8019390	-0.6111600	0.9113390
N	-1.2065940	-1.5321130	1.5192750
C	-0.4363250	-0.2189080	1.2626060
C	-3.0336000	-0.2207810	0.2739990
C	0.6178830	-0.2861290	0.2069940
O	0.6167610	-1.1060000	-0.6912680
C	1.6660080	0.7392910	0.3208350
N	1.7832500	1.8115230	0.8807800
O	2.5467830	2.7562050	0.9409580
C	3.5792740	0.6921890	-0.7832220
C	4.0832530	1.7913080	-0.5661880
C	-3.1354390	1.0498360	-0.3086280
C	-4.3237920	1.4315070	-0.9238590
C	-5.4044120	0.5487840	-0.9577060
C	-5.3025360	-0.7196030	-0.3774940
C	-4.1205550	-1.1097390	0.2388580
H	-0.2453780	0.4208730	2.1171830
H	-2.2866880	1.7234920	-0.2748650
H	-4.4081440	2.4128120	-1.3759160
H	-6.3294080	0.8476500	-1.4380980
H	-6.1455950	-1.4000730	-0.4087400
H	-4.0279140	-2.0904340	0.6911630
H	4.6119910	2.7100360	-0.4556380
C	3.6111880	-0.6529980	-1.3709590
H	2.6736150	-0.9275830	-1.8437140
H	4.4405080	-0.7415900	-2.0695930
Cl	3.9045890	-1.9243690	-0.0764190

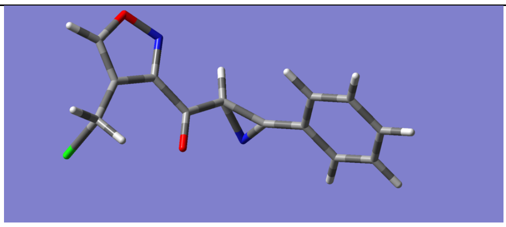

E = -1221.111443, H (0K) = -1220.917355,
H (298K) = -1220.901091,
G (298K) = -1220.963895 au.
Imaginary frequency = 0.

C	-1.8192930	-0.3139270	1.0870940
N	-1.2619630	-1.0251420	1.9538020
C	-0.4576050	0.1595020	1.3544750
C	-3.0232360	-0.1227210	0.3171500
C	0.6255420	-0.2151570	0.4099330
O	0.6053250	-1.2431650	-0.2451510
C	1.7749860	0.7290710	0.2641560
N	1.7689490	1.8955190	0.8698110
O	2.9499810	2.5219240	0.4997500
C	2.9607030	0.5504680	-0.5225000
C	3.6408060	1.7095880	-0.3202030
C	-3.0903290	0.9247190	-0.6112470
C	-4.2498020	1.1095700	-1.3584840
C	-5.3367580	0.2524550	-1.1801270
C	-5.2701360	-0.7930090	-0.2536630
C	-4.1170930	-0.9852900	0.4966310
H	-0.2952310	1.0127860	2.0017330
H	-2.2374630	1.5817550	-0.7382920
H	-4.3064090	1.9180970	-2.0780030
H	-6.2392190	0.3975940	-1.7634220
H	-6.1175780	-1.4555660	-0.1208840
H	-4.0520150	-1.7929830	1.2166940
H	4.5879190	2.0809420	-0.6820090
C	3.3751060	-0.5995390	-1.3584810
H	2.5345850	-1.0588670	-1.8678260
H	4.1501350	-0.3160060	-2.0659610
Cl	4.1121510	-1.9485200	-0.3325000

TS 2a+7i→3i (DCM)

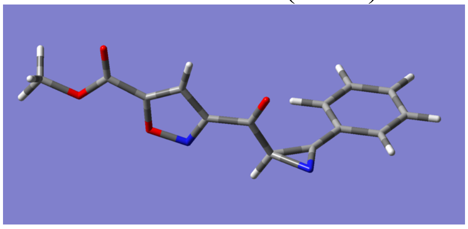

E = -950.104347, H (0K) = -949.887607,
H (298K) = -949.869201,
G (298K) = -949.937109 au.
Imaginary frequency = 1.

C	2.6970260	-1.0445730	-0.4398270

Molecule **3i** (DCM)

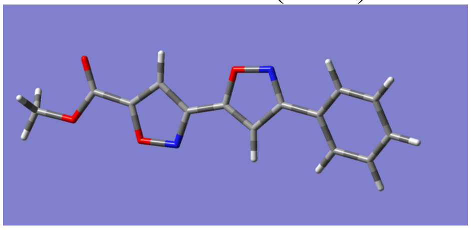

E = -950.115506, H (0K) = -949.896671,
H (298K) = -949.879122,
G (298K) = -949.944396 au.
Imaginary frequency = 0.

C	-2.3974200	-0.3240960	-0.0477790

TS 2a+7i→3'i (DCM)

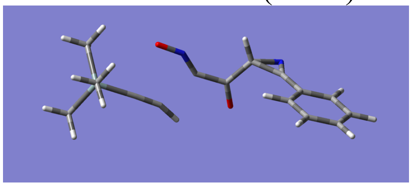

Molecule **3'i** (DCM)

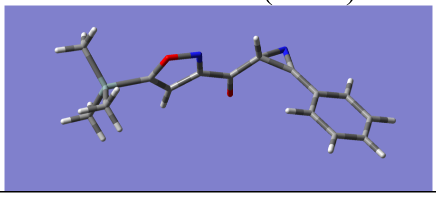

E = -1130.786233, H (0K) = -1130.517834,
H (298K) = -1130.494441,
G (298K) = -1130.572402 au.
Imaginary frequency = 1.

C	3.1329370	-1.1027680	-0.3265380
N	2.8927480	-2.3324850	-0.2786080
C	1.7395560	-1.3857370	-0.6753770
C	4.1379540	-0.0874510	-0.1401660
C	0.6878030	-1.1732300	0.3605620
O	0.8763700	-1.3196570	1.5549820
C	-0.6155930	-0.7527140	-0.1583180
N	-1.1632320	-0.5107850	-1.2093900
O	-2.2134330	-0.1557680	-1.7086530
C	-2.0747100	-0.4173930	1.3619360
C	-3.1363680	-0.0610940	0.8506110
C	3.8013550	1.2591870	-0.3341440
C	4.7690960	2.2430520	-0.1559110
C	6.0660380	1.8842980	0.2144900
C	6.4019450	0.5407550	0.4090510
C	5.4429050	-0.4485020	0.2338940
H	1.4194570	-1.3983050	-1.7116730
H	-1.3802530	-0.6821320	2.1370590
H	2.7893990	1.5223450	-0.6202580
H	4.5139140	3.2858440	-0.3039720
H	6.8188080	2.6523190	0.3529580
H	7.4109790	0.2698350	0.6974450
H	5.6897570	-1.4934440	0.3825600
Si	-4.7757320	0.4799140	0.1542130
C	-5.4224990	-0.8685490	-0.9788610
H	-5.4946090	-1.8251570	-0.4518810
H	-6.4223170	-0.6076860	-1.3430450
H	-4.7638020	-1.0006740	-1.8410210
C	-4.5351870	2.1044710	-0.7538310
H	-5.4990250	2.4826480	-1.1120800
H	-4.0990210	2.8631140	-0.0963100
H	-3.8736180	1.9742680	-1.6141700
C	-5.9225970	0.7100790	1.6290380
H	-6.9120780	1.0353790	1.2886550
H	-6.0467130	-0.2246120	2.1848840
H	-5.5365510	1.4673340	2.3185960

E = -1130.910118, H (0K) = -1130.635235,
H (298K) = -1130.613387,
G (298K) = -1130.688137 au.
Imaginary frequency = 0.

C	3.0406980	-1.0939520	-0.3593740
N	2.7882550	-2.3205430	-0.3482550
C	1.6353030	-1.3404810	-0.6933970
C	4.0627070	-0.0981380	-0.1527180
C	0.6029470	-1.1548350	0.3590940
O	0.8212840	-1.3573450	1.5422760
C	-0.7476220	-0.6912170	-0.0722860
N	-1.0199790	-0.4357330	-1.3342160
O	-2.3433430	-0.0279680	-1.3517340
C	-1.8764890	-0.4591480	0.7640310
C	-2.8586890	-0.0438560	-0.0856790
C	3.7417540	1.2582110	-0.2981190
C	4.7243750	2.2237340	-0.1005360
C	6.0211900	1.8371100	0.2408680
C	6.3417340	0.4837090	0.3868320
C	5.3675470	-0.4870760	0.1919230
H	1.3124550	-1.3140760	-1.7273230
H	-1.9324040	-0.5887050	1.8318070
H	2.7297600	1.5425700	-0.5631070
H	4.4807610	3.2739020	-0.2115280
H	6.7857220	2.5905360	0.3941920
H	7.3507660	0.1906860	0.6528610
H	5.6030840	-1.5393230	0.3029370
Si	-4.6755220	0.4718280	0.1555080
C	-5.7337620	-0.6929730	-0.8672180
H	-5.6007350	-1.7314180	-0.5482220
H	-6.7936120	-0.4381560	-0.7595050
H	-5.4781910	-0.6274610	-1.9294770
C	-4.8460930	2.2441490	-0.4388830
H	-5.8799300	2.5867980	-0.3219120
H	-4.1994310	2.9176590	0.1321340
H	-4.5804080	2.3319680	-1.4970740
C	-5.0173940	0.3067450	1.9918800
H	-6.0546060	0.5850820	2.2067640
H	-4.8698890	-0.7221660	2.3345300
H	-4.3667160	0.9610540	2.5802610

TS 2a+7k→3k (DCM)

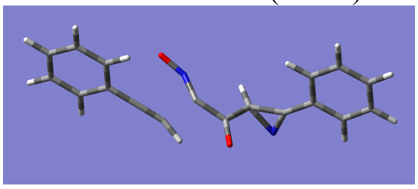

E = -953.152972, H (0K) = -952.905223,
H (298K) = -952.884828,
G (298K) = -952.958951 au.
Imaginary frequency = 1.

C	-3.3173900	-1.0615400	0.3511620
N	-3.0950110	-2.2946090	0.3016580
C	-1.9309300	-1.3654610	0.7082720
C	-4.3079140	-0.0326060	0.1613710
C	-0.8671480	-1.1682130	-0.3195000

Molecule **3k** (DCM)

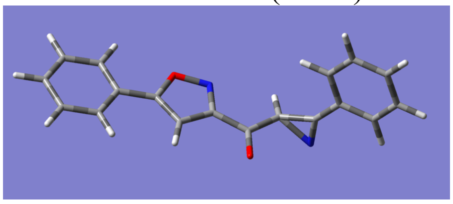

E = -953.289308, H (0K) = -953.034203,
H (298K) = -953.015673,
G (298K) = -953.084026 au.
Imaginary frequency = 0.

C	3.2382880	-1.0398530	-0.3441160
N	3.0224190	-2.2732730	-0.3359990
C	1.8438330	-1.3281150	-0.6921090
C	4.2283920	-0.0143920	-0.1273290
C	0.7969920	-1.1748700	0.3503610

O	-1.0534330	-1.3120160	-1.5145160
C	0.4405200	-0.7707570	0.2143120
N	0.9436350	-0.5312090	1.2868590
O	1.9467020	-0.1988480	1.8879050
C	1.8616950	-0.5391670	-1.3285130
C	2.9795850	-0.2066480	-0.9479890
C	-3.9557280	1.3088360	0.3630850
C	-4.9098160	2.3055740	0.1825780
C	-6.2086720	1.9647520	-0.1979020
C	-6.5601480	0.6263370	-0.4002290
C	-5.6147800	-0.3756110	-0.2227540
H	-1.6183440	-1.3856280	1.7467400
H	1.1415000	-0.8042350	-2.0800020
H	-2.9424840	1.5579860	0.6570920
H	-4.6425520	3.3444540	0.3367570
H	-6.9507780	2.7427780	-0.3380920
H	-7.5705930	0.3693080	-0.6962990
H	-5.8737160	-1.4167770	-0.3772240
C	4.2779680	0.1754680	-0.5266430
C	5.2357600	-0.8042660	-0.1974630
C	4.6318010	1.5377530	-0.4597760
C	6.5138880	-0.4238300	0.1935600
H	4.9645110	-1.8520880	-0.2474160
C	5.9133770	1.9051840	-0.0673770
H	3.8960870	2.2922020	-0.7117310
C	6.8566560	0.9286920	0.2610990
H	7.2448650	-1.1830680	0.4475810
H	6.1779060	2.9553170	-0.0159870
H	7.8547370	1.2204000	0.5681000

O	1.0093050	-1.3632520	1.5365080
C	-0.5658600	-0.7595490	-0.0960120
N	-0.8349370	-0.5310320	-1.3659300
O	-2.1696370	-0.1637610	-1.4002020
C	-1.6998440	-0.5523580	0.7302780
C	-2.6838610	-0.1787330	-0.1406690
C	3.8693550	1.3321470	-0.2741500
C	4.8214510	2.3256220	-0.0658720
C	6.1256760	1.9764920	0.2875440
C	6.4842740	0.6327860	0.4347120
C	5.5407220	-0.3656960	0.2292110
H	1.5302950	-1.3111340	-1.7291250
H	-1.7594010	-0.6682550	1.7983770
H	2.8520690	1.5874850	-0.5483450
H	4.5483190	3.3684290	-0.1777410
H	6.8662440	2.7518010	0.4493040
H	7.4988540	0.3687970	0.7099480
H	5.8054980	-1.4108590	0.3411590
C	-4.0885230	0.1806650	0.0215560
C	-4.6582360	0.1989830	1.3047620
C	-4.8827320	0.5084060	-1.0884420
C	-5.9964710	0.5381180	1.4712860
H	-4.0548440	-0.0511190	2.1694610
C	-6.2214750	0.8471690	-0.9135880
H	-4.4542510	0.4969380	-2.0829850
C	-6.7824820	0.8631130	0.3637830
H	-6.4267800	0.5490850	2.4662420
H	-6.8270130	1.0985740	-1.7769720
H	-7.8257140	1.1268760	0.4964850

TS 2a+7k→3'k (DCM)

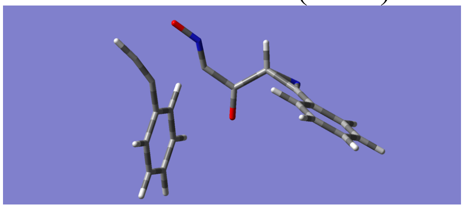

E = -953.140997, H (0K) = -952.892878,
H (298K) = -952.872846,
G (298K) = -952.944605 au.
Imaginary frequency = 1.

C	2.3228990	0.0685100	-1.1694500
N	1.7538880	-0.2677770	-2.2354450
C	1.0860660	0.8229890	-1.3706930
C	3.4576190	-0.1705520	-0.3141750
C	-0.1381650	0.3946770	-0.6231520
O	-0.3603300	-0.7580040	-0.3134250
C	-1.0543860	1.4933100	-0.2762970
N	-0.9832740	2.7073960	-0.2359590
O	-1.6612890	3.6608250	0.1082320
C	-3.1316210	1.2271670	0.4708840
C	-3.4675070	2.3790420	0.7467770
C	3.5841950	0.5497230	0.8814370
C	4.6776190	0.3199090	1.7109490
C	5.6394040	-0.6244360	1.3487270
C	5.5129420	-1.3438610	0.1560900
C	4.4248190	-1.1218250	-0.6780970

Molecule **3'k** (DCM)

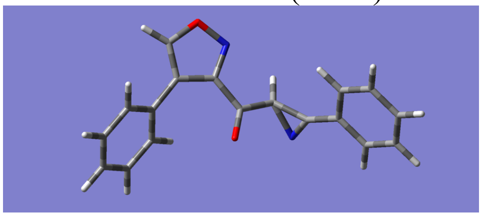

E = -953.278296, H (0K) = -953.023114,
H (298K) = -953.004639,
G (298K) = -953.072908 au.
Imaginary frequency = 0.

C	2.4488960	-0.3947950	-1.0969810
N	1.8880810	-1.0297100	-2.0194830
C	1.1805470	0.2367790	-1.4700580
C	3.5928780	-0.3435600	-0.2217030
C	-0.0200130	-0.0093870	-0.6271130
O	-0.2263700	-1.0697550	-0.0654720
C	-0.9765560	1.1304210	-0.4633370
N	-0.5306190	2.3588840	-0.6142460
O	-1.5923230	3.1909430	-0.3052030
C	-2.3540560	1.1102940	-0.0533350
C	-2.6600890	2.4357100	0.0180400
C	3.6865490	0.6846060	0.7260260
C	4.7868340	0.7388720	1.5764100
C	5.7885110	-0.2282430	1.4808390
C	5.6950680	-1.2544870	0.5352960
C	4.6004450	-1.3173490	-0.3175120

H	1.1125780	1.8392250	-1.7485110
H	2.8278060	1.2783610	1.1498110
H	4.7797180	0.8732390	2.6371480
H	6.4903660	-0.8028340	1.9965840
H	6.2632780	-2.0763170	-0.1180410
H	4.3134410	-1.6739030	-1.6042370
C	-3.3617260	-0.2014550	0.3579600
C	-3.4436840	-0.9907750	1.5132830
C	-3.5294120	-0.7959230	-0.8997780
C	-3.6921570	-2.3569900	1.4066250
H	-3.3075650	-0.5325960	2.4859130
C	-3.7877060	-2.1598510	-0.9974190
H	-3.4521090	-0.1882150	-1.7938330
C	-3.8659270	-2.9456330	0.1534430
H	-3.7504920	-2.9617490	2.3048960
H	-3.9195920	-2.6111640	-1.9745620
H	-4.0589430	-4.0096530	0.0739700
H	-3.9194680	3.3015470	1.0295470

H	1.1801000	1.1116980	-2.1091820
H	2.8996340	1.4277300	0.7879630
H	4.8636170	1.5315650	2.3114670
H	6.6449610	-0.1849110	2.1444290
H	6.4764050	-2.0026390	0.4673880
H	4.5150690	-2.1085550	-1.0535880
C	-3.2655980	-0.0185700	0.2005290
C	-4.1610040	0.0350450	1.2780230
C	-3.2857730	-1.1367270	-0.6441670
C	-5.0584670	-1.0070870	1.5045940
H	-4.1428050	0.8889550	1.9463210
C	-4.1815070	-2.1772520	-0.4146020
H	-2.6026800	-1.1894680	-1.4831140
C	-5.0707760	-2.1169100	0.6600530
H	-5.7418520	-0.9540640	2.3449260
H	-4.1863590	-3.0359430	-1.0768540
H	-5.7658720	-2.9298860	0.8381320
H	-3.5682280	2.9676420	0.2576030

TS 2a+7l→3l (DCM)

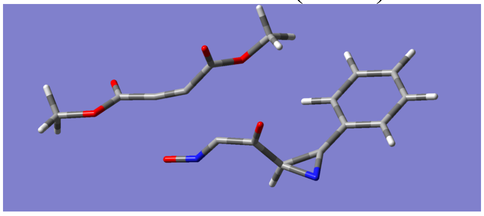

E = -1177.914616, H (0K) = -1177.661364,
H (298K) = -1177.636776,
G (298K) = -1177.719612 au.
Imaginary frequency = 1.

C	2.8375980	-1.5988630	0.2388690
N	2.6755300	-2.5236070	1.0688990
C	1.4532600	-2.0823620	0.2367910
C	3.7522970	-0.6348690	-0.3143370
C	0.4471270	-1.2497630	0.9433860
O	0.6642610	-0.6186200	1.9572290
C	-0.8702320	-1.1740910	0.2795630
N	-1.6218610	-1.8496640	-0.3811870
O	-2.7310670	-1.8661390	-0.8688110
C	-2.0317020	0.6048170	0.4293210
C	-3.1262860	0.3980320	-0.0948180
C	3.2896480	0.2694060	-1.2805390
C	4.1604390	1.2176930	-1.8077970
C	5.4854490	1.2634770	-1.3713800
C	5.9467210	0.3616060	-0.4070720
C	5.0855140	-0.5890570	0.1254310
O	-5.3387930	-0.2130020	0.0373810
H	1.0983290	-2.7540480	-0.5374500
H	2.2570000	0.2225650	-1.6061370
H	3.8077860	1.9198470	-2.5541240
H	6.1628970	2.0036970	-1.7822530
H	6.9766850	0.4045450	-0.0725990
H	5.4293150	-1.2915630	0.8758180
C	-6.6821440	-0.2860300	-0.5072740
H	-6.6767050	-0.8456610	-1.4436810
H	-7.2656730	-0.8078820	0.2475060
H	-7.0766970	0.7174380	-0.6722170

Molecule **3l** (DCM)

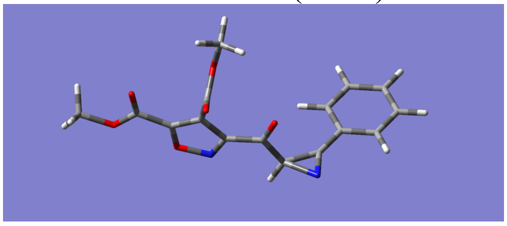

E = -1178.049346, H (0K) = -1177.790305,
H (298K) = -1177.767134,
G (298K) = -1177.846600 au.
Imaginary frequency = 0.

C	-3.1359470	-0.4938450	-1.1433040
N	-2.8560530	-0.2857310	-2.3453570
C	-1.7470360	-0.8702730	-1.4309330
C	-4.1601040	-0.4000650	-0.1337910
C	-0.6636700	0.0673960	-1.0559090
O	-0.7981640	1.2779030	-1.0231300
C	0.6628490	-0.5162550	-0.6869090
N	0.8766950	-1.8134340	-0.6422770
O	2.1844180	-1.9776990	-0.2473260
C	1.8327820	0.2130650	-0.3212520
C	2.7444630	-0.7606750	-0.0577330
C	-3.8729990	-0.7982550	1.1787860
C	-4.8585350	-0.7063110	2.1569260
C	-6.1238180	-0.2188520	1.8261880
C	-6.4102840	0.1789720	0.5164240
C	-5.4332320	0.0910090	-0.4668170
O	4.7362370	-1.8252800	0.5334860
H	-1.4860450	-1.9108940	-1.5834610
H	-2.8850290	-1.1735680	1.4201290
H	-4.6416680	-1.0120140	3.1737680
H	-6.8903490	-0.1471780	2.5897810
H	-7.3948490	0.5573980	0.2670920
H	-5.6414980	0.3973440	-1.4854780
C	6.1263360	-1.8103130	0.9564840
H	6.2154970	-1.3215310	1.9273620
H	6.4126790	-2.8568590	1.0245060

C	-4.4270850	0.3913890	-0.7252960
O	-4.6376570	0.8963530	-1.8074330
C	-1.1085930	1.5640340	1.0599570
O	-1.3500710	2.1374360	2.0932370
O	-0.0134580	1.7175640	0.3169770
C	1.0147860	2.5867220	0.8585360
H	0.6292600	3.6003040	0.9739810
H	1.3549610	2.1991070	1.8192910
H	1.8206750	2.5629850	0.1291830

H	6.7351360	-1.2906810	0.2157800
C	4.1553220	-0.6424820	0.3774540
O	4.6709410	0.4376880	0.5578800
C	2.0273960	1.6953650	-0.3060260
O	2.3872070	2.3229440	-1.2718620
O	1.7549330	2.2013430	0.8924210
C	1.8894010	3.6407920	1.0259610
H	2.9172950	3.9407040	0.8181310
H	1.2044330	4.1432110	0.3417240
H	1.6277830	3.8573990	2.0590530

TS 2a+7k→3k (DMF)

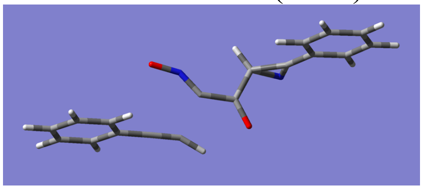

E = -953.148529, H (0K) = -952.900630,
H (298K) = -952.880318,
G (298K) = -952.953545 au.
Imaginary frequency = 1.

C	-3.3216470	-1.0663130	0.3600490
N	-3.1063760	-2.3012010	0.3153060
C	-1.9360600	-1.3759680	0.7147790
C	-4.3035340	-0.0309180	0.1649380
C	-0.8757470	-1.1839240	-0.3175290
O	-1.0647330	-1.3334950	-1.5114990
C	0.4328470	-0.7832990	0.2116410
N	0.9456180	-0.5574860	1.2824170
O	1.9550840	-0.2308690	1.8761850
C	1.8491980	-0.5081030	-1.3295820
C	2.9672310	-0.1823230	-0.9433680
C	-3.9352700	1.3087550	0.3496270
C	-4.8794100	2.3135340	0.1616800
C	-6.1837690	1.9822980	-0.2086650
C	-6.5510590	0.6454770	-0.3935410
C	-5.6158190	-0.3646430	-0.2089350
H	-1.6216250	-1.3911530	1.7527450
H	1.1308440	-0.7536070	-2.0893240
H	-2.9176230	1.5490660	0.6359100
H	-4.6002830	3.3512320	0.3024460
H	-6.9181510	2.7666820	-0.3543030
H	-7.5658570	0.3962550	-0.6814350
H	-5.8873850	-1.4046570	-0.3495350
C	4.2688560	0.1895720	-0.5226690
C	5.2357670	-0.7982990	-0.2491790
C	4.6171060	1.5496780	-0.4029750
C	6.5177070	-0.4280470	0.1398650
H	4.9695970	-1.8447300	-0.3411090
C	5.9024360	1.9067650	-0.0129820
H	3.8746920	2.3107060	-0.6123350
C	6.8550490	0.9221680	0.2603880
H	7.2560360	-1.1936500	0.3499770
H	6.1628460	2.9552080	0.0789790
H	7.8561230	1.2059100	0.5651860

Molecule **3k** (DMF)

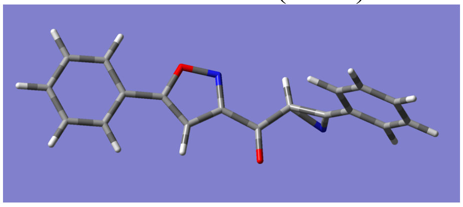

E = -953.285319, H (0K) = -953.030191,
H (298K) = -953.011652,
G (298K) = -953.080136 au.
Imaginary frequency = 0.

C	3.2401010	-1.0468620	-0.3271470
N	3.0304480	-2.2814420	-0.2981510
C	1.8469790	-1.3476690	-0.6687340
C	4.2231340	-0.0121150	-0.1272530
C	0.8002400	-1.1796340	0.3719910
O	1.0124920	-1.3506790	1.5605650
C	-0.5631080	-0.7728250	-0.0825060
N	-0.8364370	-0.5889100	-1.3589760
O	-2.1718890	-0.2193560	-1.4011960
C	-1.6932530	-0.5330980	0.7398700
C	-2.6796640	-0.1880040	-0.1401360
C	3.8508670	1.3295740	-0.2859180
C	4.7955960	2.3332010	-0.0935660
C	6.1050070	1.9988340	0.2552980
C	6.4765090	0.6598520	0.4141350
C	5.5405340	-0.3488770	0.2248640
H	1.5344060	-1.3478710	-1.7062390
H	-1.7522610	-0.6084170	1.8117280
H	2.8294570	1.5724820	-0.5562640
H	4.5128900	3.3724620	-0.2145330
H	6.8398780	2.7821500	0.4042590
H	7.4952060	0.4077440	0.6853990
H	5.8157490	-1.3904980	0.3453130
C	-4.0822590	0.1811330	0.0171750
C	-4.6496150	0.2253510	1.3009600
C	-4.8764000	0.4933070	-1.0973660
C	-5.9856960	0.5752340	1.4630130
H	-4.0467070	-0.0135680	2.1691670
C	-6.2130180	0.8427100	-0.9267740
H	-4.4506990	0.4628630	-2.0927280
C	-6.7717550	0.8848170	0.3510320
H	-6.4143700	0.6064000	2.4582980
H	-6.8183790	1.0823650	-1.7936610
H	-7.8132570	1.1570520	0.4805660

TS 2a+7k→3'k (DMF)

Molecule **3'k** (DMF)

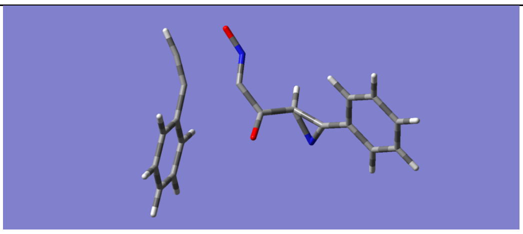

E = -953.136849, H (0K) = -952.888567,
H (298K) = -952.868586,
G (298K) = -952.939917 au.
Imaginary frequency = 1.

C	2.3045670	-0.1864570	-1.1212080
N	1.6722110	-0.7151600	-2.0668720
C	1.0603230	0.5332000	-1.3920380
C	3.4841130	-0.2701510	-0.2988010
C	-0.1157210	0.2772870	-0.5050080
O	-0.3309100	-0.7903370	0.0321050
C	-0.9996460	1.4402080	-0.3162090
N	-0.8817600	2.6480010	-0.3909370
O	-1.5250470	3.6562190	-0.1476100
C	-3.1022870	1.3176550	0.3703620
C	-3.3952580	2.4978780	0.5649500
C	3.6683390	0.6586130	0.7345220
C	4.8034090	0.5800200	1.5356510
C	5.7495860	-0.4200160	1.3056190
C	5.5656610	-1.3469380	0.2745140
C	4.4353910	-1.2774550	-0.5298060
H	1.0684570	1.4575430	-1.9591500
H	2.9236070	1.4287420	0.9005390
H	4.9504690	1.2949520	2.3367630
H	6.6336110	-0.4793460	1.9307180
H	6.3044260	-2.1212080	0.1027200
H	4.2806500	-1.9906090	-1.3313600
C	-3.4054350	-0.1009680	0.3262880
C	-3.7399900	-0.7811040	1.5060160
C	-3.4015540	-0.7923260	-0.8927720
C	-4.0653590	-2.1347270	1.4610540
H	-3.7399600	-0.2484470	2.4498550
C	-3.7408610	-2.1414260	-0.9316090
H	-3.1290330	-0.2700320	-1.8026470
C	-4.0687950	-2.8184620	0.2442730
H	-4.3193830	-2.6546560	2.3782900
H	-3.7405810	-2.6669380	-1.8801810
H	-4.3243890	-3.8717810	0.2127330
H	-3.8122830	3.4558510	0.7756440

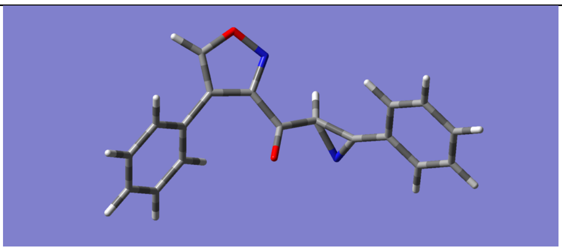

E = -953.276378, H (0K) = -953.021161,
H (298K) = -953.002712,
G (298K) = -953.070688 au.
Imaginary frequency = 0.

C	2.4439590	-0.4290200	-1.0857220
N	1.8741230	-1.0924810	-1.9825400
C	1.1717480	0.1909620	-1.4659600
C	3.5945070	-0.3524430	-0.2214880
C	-0.0195620	-0.0269690	-0.6022390
O	-0.2210010	-1.0678230	-0.0036540
C	-0.9725420	1.1199530	-0.4645230
N	-0.5192760	2.3438390	-0.6292190
O	-1.5814370	3.1859370	-0.3381100
C	-2.3523910	1.1119370	-0.0644200
C	-2.6533270	2.4394290	-0.0129940
C	3.6890290	0.6969060	0.7027400
C	4.7943080	0.7746350	1.5447960
C	5.8001110	-0.1896760	1.4636590
C	5.7059120	-1.2367130	0.5411140
C	4.6061090	-1.3235220	-0.3028980
H	1.1654750	1.0451140	-2.1327870
H	2.8984150	1.4370930	0.7534060
H	4.8719830	1.5834870	2.2620210
H	6.6606080	-0.1277670	2.1205880
H	6.4909060	-1.9820340	0.4841170
H	4.5207030	-2.1307550	-1.0215020
C	-3.2698790	-0.0098490	0.1963320
C	-4.1748990	0.0606910	1.2648530
C	-3.2852940	-1.1389220	-0.6338340
C	-5.0767340	-0.9765740	1.4972020
H	-4.1626060	0.9247670	1.9201630
C	-4.1855960	-2.1744600	-0.3985280
H	-2.5966570	-1.2030460	-1.4675190
C	-5.0840260	-2.0979110	0.6676720
H	-5.7684070	-0.9100430	2.3298480
H	-4.1879540	-3.0412440	-1.0503640
H	-5.7832490	-2.9065240	0.8498920
H	-3.5622430	2.9758210	0.2139940

Molecule **1a** (DCM)

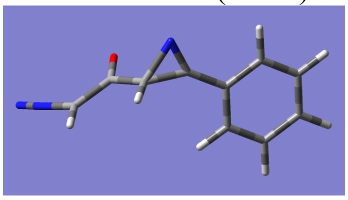

E = -624.842378, H (0K) = -624.691963,
H (298K) = -624.678992,
G (298K) = -624.732396 au.
Imaginary frequency = 0.

C	-0.4084130	1.0260990	-0.3491210

Molecule **TBN** (DCM)

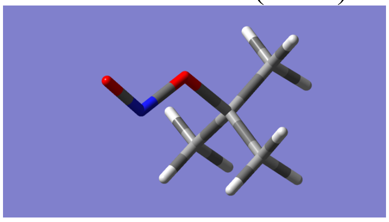

E = -363.089139, H (0K) = -362.957660,
H (298K) = -362.948346,
G (298K) = -362.989776 au.
Imaginary frequency = 0.

N	0.1570200	2.1328710	-0.1757250
C	0.9747370	0.9858500	-0.8163790
C	2.0211580	0.3550340	0.0457030
O	1.9033110	0.2718800	1.2662520
C	-1.6447380	0.3059880	-0.1624570
C	-1.7259030	-1.0391040	-0.5467020
C	-2.9162500	-1.7377700	-0.3670780
C	-4.0217650	-1.0965360	0.1936690
C	-3.9417550	0.2457130	0.5778780
C	-2.7572780	0.9501990	0.4028500
H	1.1966950	1.0878920	-1.8747240
H	-0.8582900	-1.5250850	-0.9783170
H	-2.9821720	-2.7787860	-0.6613520
H	-4.9483300	-1.6420800	0.3337340
H	-4.8034540	0.7375730	1.0142730
H	-2.6820340	1.9903090	0.6987880
C	3.1686800	-0.1513530	-0.6870360
H	3.3227740	-0.0751500	-1.7534860
N	4.1094730	-0.7471050	0.0022930
N	4.8817200	-1.2478260	0.6510170

N	-1.6156350	-0.2955430	0.0001170
O	-2.6748580	0.2398380	-0.0000540
O	-0.5912500	0.6192570	-0.0003250
C	0.7659270	-0.0118920	0.0000070
C	1.6896690	1.1996200	-0.0007540
C	0.9239820	-0.8425210	1.2703220
C	0.9238020	-0.8440360	-1.2693480
H	2.7290960	0.8625220	-0.0005710
H	1.5239970	1.8130870	-0.8895110
H	1.5240290	1.8141390	0.8872850
H	1.9475490	-1.2214340	1.3286090
H	0.2421350	-1.6952120	1.2746750
H	0.7311330	-0.2308920	2.1552450
H	0.7308500	-0.2334480	-2.1549660
H	1.9473450	-1.2230550	-1.3273300
H	0.2419050	-1.6966940	-1.2725800

TS 1a+TBN→*Bconformer*+ N_2 (DCM)

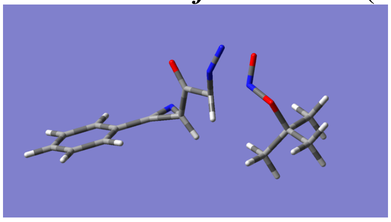

E = -987.892445, H (0K) = -987.608932,
H (298K) = -987.587482,
G (298K) = -987.660165 au.
Imaginary frequency = 1.

C	-2.1836180	-0.4674830	-0.7658950
N	-1.5050590	-0.5834360	-1.8114920
C	-0.7476870	-0.3750170	-0.4759930
C	-0.1452360	0.9614730	-0.2698990
O	-0.5725650	1.9881250	-0.7559680
C	-3.5143840	-0.4345040	-0.2147000
C	-3.6863190	-0.2359110	1.1619440
C	-4.9700470	-0.2023130	1.6976450
C	-6.0765660	-0.3661170	0.8628820
C	-5.9053370	-0.5639120	-0.5108590
C	-4.6279850	-0.5984470	-1.0551010
H	-0.2159050	-1.2319900	-0.0793070
H	-2.8171740	-0.1078440	1.7969740
H	-5.1086820	-0.0473420	2.7612330
H	-7.0763200	-0.3389040	1.2816600
H	-6.7693420	-0.6889470	-1.1531040
H	-4.4818880	-0.7490470	-2.1185200
C	1.1279860	0.9478680	0.5611880
H	1.2047650	0.2150720	1.3548350
N	1.5044330	2.2240520	0.9468280
N	2.1919750	3.0673020	0.6114590
N	2.4641860	0.5764640	-0.5718060
O	2.8333280	1.6204340	-1.1015160
O	3.5240040	0.1234760	0.3995080

Molecule ***Bconformer*** (DCM)

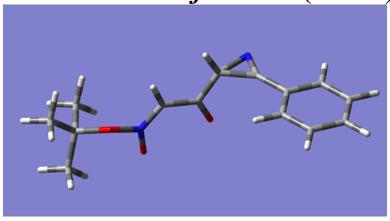

E = -878.430000, H (0K) = -878.152701,
H (298K) = -878.133120,
G (298K) = -878.202485 au.
Imaginary frequency = 0.

C	2.4830010	1.0412270	0.4767630
N	2.0731440	2.2261050	0.4602210
C	1.0684570	1.1096960	0.8410210
C	0.0303600	0.7765310	-0.1851900
O	0.2352850	0.9350870	-1.3820070
C	3.6303810	0.1962770	0.2511260
C	3.5100000	-1.1896790	0.4185500
C	4.6146240	-2.0090760	0.2049680
C	5.8345010	-1.4474970	-0.1748360
C	5.9552410	-0.0644790	-0.3434930
C	4.8578190	0.7607770	-0.1325130
H	0.7790570	1.0637640	1.8865900
H	2.5553240	-1.6117730	0.7111400
H	4.5251470	-3.0816210	0.3325160
H	6.6940340	-2.0870740	-0.3418160
H	6.9050740	0.3651880	-0.6402750
H	4.9384360	1.8339910	-0.2621020
C	-1.2066880	0.2509430	0.3914930
H	-1.3361210	0.1552690	1.4572900
N	-2.2224100	-0.1663100	-0.3416020
O	-2.3683720	-0.1406470	-1.5573330
O	-3.2279210	-0.7744730	0.4499670
C	-4.6371570	-0.3307450	0.1869930
C	-5.3324480	-0.8500460	1.4428930

C	3.8422740	-1.2928960	0.2501410
C	4.9223160	-1.5116970	1.3094220
C	2.6188880	-2.1688940	0.5345520
C	4.3917070	-1.5498240	-1.1554750
H	5.2632460	-2.5502840	1.2836000
H	5.7797110	-0.8601140	1.1238750
H	4.5319450	-1.2994230	2.3082280
H	2.9080260	-3.2232260	0.5186980
H	1.8455650	-2.0232590	-0.2216630
H	2.2017360	-1.9458180	1.5206930
H	5.2447190	-0.8961830	-1.3550730
H	4.7193440	-2.5890790	-1.2481390
H	3.6274240	-1.3645140	-1.9142130

C	-4.6892740	1.1908780	0.1073910
C	-5.1887330	-1.0103820	-1.0640270
H	-6.3987480	-0.6187590	1.3842510
H	-5.2193810	-1.9336040	1.5271830
H	-4.9209170	-0.3803020	2.3389120
H	-5.7339300	1.5048630	0.0439400
H	-4.1731500	1.5684270	-0.7774960
H	-4.2484510	1.6424200	0.9994430
H	-5.0182780	-2.0886780	-1.0153630
H	-6.2670400	-0.8346030	-1.1153160
H	-4.7286200	-0.6223630	-1.9710590

TS *Bf-t*-BuOH →2a+ *t*-BuOH (DCM)

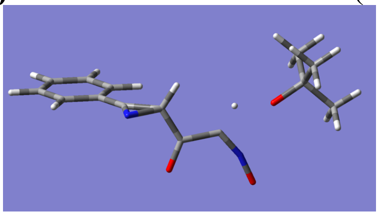

E = -878.356565, H (0K) = -878.086769,
H (298K) = -878.066134,
G (298K) = -878.140992 au.
Imaginary frequency = 1.

C	-2.3676070	-0.5934600	1.0008890
N	-2.1023750	-1.5227870	1.7969330
C	-0.9174130	-0.7853890	1.1329910
C	-0.1943440	-1.5312960	0.0787480
O	-0.6470430	-2.4403520	-0.5760720
N	1.9804510	-1.5060830	-0.9800720
O	2.4358370	-2.1580570	-1.8437240
O	3.2322570	0.2207240	0.0433650
C	4.3506850	1.0262870	0.2555280
C	4.8509530	0.8645580	1.7038730
C	5.4433260	0.5475380	-0.7289200
C	4.0037370	2.4998060	-0.0313190
C	-3.4126800	0.1647090	0.3642320
C	-3.0746530	1.1794540	-0.5415320
C	-4.0826460	1.9128760	-1.1593400
C	-5.4201980	1.6343080	-0.8739490
C	-5.7572960	0.6217600	0.0299390
C	-4.7588560	-0.1164700	0.6517770
H	-0.3282820	-0.1229920	1.7579300
H	5.7416870	1.4726360	1.8931690
H	4.0686370	1.1728640	2.4045900
H	5.0945860	-0.1832690	1.9014790
H	6.3524610	1.1459170	-0.6126990
H	5.0894150	0.6422320	-1.7590150
H	5.6863930	-0.5015080	-0.5402320
H	3.2077030	2.8327290	0.6422080
H	4.8703530	3.1539540	0.1104340
H	3.6495640	2.6078620	-1.0606240
H	-2.0310690	1.3823260	-0.7534100
H	-3.8276510	2.6981640	-1.8611370
H	-6.2044710	2.2063740	-1.3569200
H	-6.7983380	0.4111080	0.2454280

TS *Bf* →*B* (DCM)

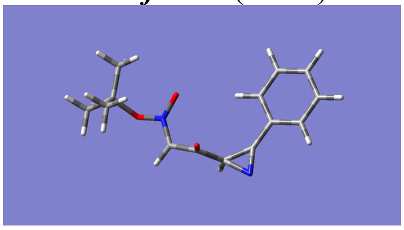

E = -878.420833, H (0K) = -878.144213,
H (298K) = -878.125358,
G (298K) = -878.192246 au.
Imaginary frequency = 1.

C	2.4000450	-1.3340770	-0.3757440
N	2.3803930	-2.5832710	-0.2961650
C	1.0787750	-1.8618950	-0.7350060
C	-0.0147040	-1.7500330	0.2574010
O	0.1311150	-1.9218790	1.4518620
C	3.1783690	-0.1346510	-0.1996810
C	2.5445250	1.1103280	-0.3142800
C	3.2838060	2.2758540	-0.1375050
C	4.6479650	2.1986240	0.1473810
C	5.2796250	0.9560500	0.2620870
C	4.5498990	-0.2136500	0.0923540
H	0.7975500	-1.9403600	-1.7806120
H	1.4812030	1.1525130	-0.5233480
H	2.7979900	3.2411030	-0.2195510
H	5.2223910	3.1082560	0.2830030
H	6.3390310	0.9042650	0.4851660
H	5.0262710	-1.1833020	0.1807520
C	-1.3554340	-1.3782470	-0.3155960
H	-1.9862340	-2.0897330	-0.8268590
N	-1.7592560	-0.1478510	-0.2201730
O	-1.1695950	0.8181260	0.3035640
O	-2.9934590	0.0950410	-0.8745430
C	-4.0817160	0.6220130	0.0085630
C	-5.3124840	0.3511360	-0.8521630
C	-4.1218020	-0.1708460	1.3098250
C	-3.8929330	2.1194190	0.2420810
H	-6.2021620	0.7231860	-0.3381960
H	-5.2314530	0.8639610	-1.8138310
H	-5.4338030	-0.7194930	-1.0317510
H	-4.9856160	0.1532090	1.8951390
H	-3.2267320	-0.0008580	1.9125270

H	-5.0063110	-0.9052100	1.3527250	H	-4.2211890	-1.2411070	1.1138800
C	1.2252610	-1.0279840	-0.1306300	H	-3.7644000	2.6378730	-0.7112540
H	1.7907670	-0.1698070	0.4157790	H	-4.7838250	2.5191620	0.7351710
				H	-3.0250830	2.3187280	0.8687720

Molecule ***B*** (DCM)

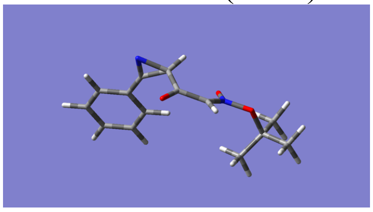

E = -878.431238, H (0K) = -878.153525,
H (298K) = -878.134160,
G (298K) = -878.202035 au.
Imaginary frequency = 0.

C	-2.2090380	1.2011750	-0.4981960
N	-2.2984130	2.4355640	-0.7075510
C	-0.8945640	1.7870760	-0.7343660
C	-0.0063050	2.1004800	0.4207640
O	-0.3980200	2.7482590	1.3857450
N	1.8534260	0.6815840	-0.3471810
O	1.2435670	-0.0398720	-1.1448220
O	3.2439620	0.5115790	-0.2210730
C	3.7088400	-0.8863720	0.0828270
C	5.1273020	-0.6111880	0.5731800
C	2.8405230	-1.4850070	1.1829880
C	3.7198240	-1.7291670	-1.1895170
C	-2.9280390	-0.0117400	-0.1983830
C	-2.2126170	-1.2123730	-0.0863400
C	-2.8897560	-2.3929140	0.2046230
C	-4.2738100	-2.3749730	0.3843600
C	-4.9875080	-1.1773900	0.2726170
C	-4.3205220	0.0060940	-0.0186600
H	-0.4240230	1.6539620	-1.7020980
H	5.6154120	-1.5596880	0.8098560
H	5.7121870	-0.1068870	-0.1998280
H	5.1158530	0.0106960	1.4708520
H	3.2624360	-2.4483470	1.4790320
H	1.8182380	-1.6594260	0.8412350
H	2.8195710	-0.8351210	2.0609550
H	4.2522690	-1.2048850	-1.9867400
H	4.2419200	-2.6686500	-0.9867790
H	2.7112400	-1.9559400	-1.5309380
H	-1.1382740	-1.2060910	-0.2344160
H	-2.3416980	-3.3239480	0.2911570
H	-4.8001210	-3.2952720	0.6122290
H	-6.0620380	-1.1710660	0.4147820
H	-4.8620560	0.9410540	-0.1059390
C	1.3909530	1.6482160	0.4160120
H	2.0862210	2.0783600	1.1195170

TS *B*+7a→*C*a (DCM)

Molecule ***C*a** (DCM)

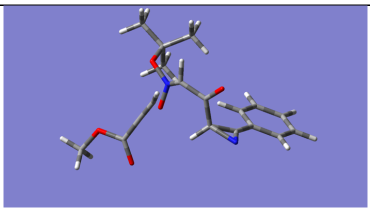

E = -1183.722110, H (0K) = -1183.372539,
H (298K) = -1183.346145,
G (298K) = -1183.430830 au.
Imaginary frequency = 1.

C	2.1812660	-1.6940170	0.3782910
N	1.8664010	-2.7142060	1.0353550
C	0.7606680	-1.6756750	0.7248940
C	0.3214930	-0.8357310	1.8709730
O	0.8502450	-0.8909570	2.9679470
N	-1.0449560	0.7243950	0.4926820
O	-1.2295400	0.0225470	-0.5206450
O	-1.7290940	1.9494330	0.5580550
C	-0.9225690	3.1091300	0.0690280
C	-1.9077530	4.2582040	0.2430730
C	0.3079970	3.2786980	0.9550200
C	-0.5483300	2.9045830	-1.3957530
C	3.2473150	-0.9774910	-0.2756850
C	2.9578150	0.2380160	-0.9110500
C	3.9753700	0.9457060	-1.5431860
C	5.2772480	0.4422960	-1.5402150
C	5.5667270	-0.7703470	-0.9064350
C	4.5568060	-1.4840960	-0.2732050
H	0.0242390	-1.9382680	-0.0214040
H	-1.4367280	5.1904920	-0.0773800
H	-2.8022390	4.0946310	-0.3624380
H	-2.2029760	4.3602810	1.2900570
H	0.8475190	4.1806430	0.6554390
H	0.9916800	2.4321920	0.8562880
H	0.0211830	3.3798500	2.0044850
H	-1.4376310	2.7132440	-1.9996120
H	-0.0595680	3.8089680	-1.7677500
H	0.1442110	2.0700040	-1.5196600
H	1.9405370	0.6124740	-0.9066360
H	3.7552670	1.8850650	-2.0370330
H	6.0701100	0.9941330	-2.0326900
H	6.5802260	-1.1545100	-0.9077960
H	4.7686740	-2.4242250	0.2231240
C	-0.8015910	0.1555240	1.6979040
H	-0.8915620	0.8429050	2.5298500
C	-2.3400570	-1.0932190	1.7695600
C	-2.7461640	-1.3784200	0.6343030
H	-2.4485840	-1.2402270	2.8291080
C	-3.2758460	-1.8603860	-0.6057810
O	-2.7991780	-2.7866520	-1.2345310
O	-4.3652760	-1.1687950	-0.9829470
C	-4.9587540	-1.5575420	-2.2442180
H	-5.8082820	-0.8908540	-2.3775550
H	-4.2416480	-1.4280540	-3.0567410
H	-5.2916430	-2.5960800	-2.2060560

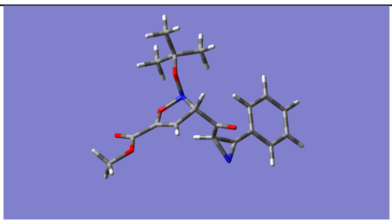

E = -1183.808935, H (0K) = -1183.455245,
H (298K) = -1183.429672,
G (298K) = -1183.512823 au.
Imaginary frequency = 0.

C	-2.3125230	-1.6673200	0.1143730
N	-1.7060920	-2.7543540	-0.0233710
C	-0.8740390	-1.4431570	-0.0488540
C	-0.3487480	-1.0289050	-1.3754510
O	-0.8283110	-1.4036570	-2.4273200
N	0.8947200	0.8263780	-0.2340740
O	1.8648920	0.2361450	0.7183860
O	1.4892900	2.0006550	-0.6981230
C	0.9531010	3.2075190	-0.0316230
C	1.7712320	4.3202250	-0.6782910
C	-0.5326990	3.3414970	-0.3571970
C	1.2013420	3.1329140	1.4728350
C	-3.6020140	-1.0486960	0.2973860
C	-3.6871250	0.3470750	0.3856110
C	-4.9273870	0.9526260	0.5628580
C	-6.0781220	0.1678670	0.6506690
C	-5.9940410	-1.2253420	0.5615550
C	-4.7601230	-1.8384930	0.3849230
H	-0.2627500	-1.2375860	0.8224580
H	1.4607710	5.2869260	-0.2746150
H	2.8361460	4.1845580	-0.4739190
H	1.6200490	4.3325300	-1.7607400
H	-0.9192690	4.2772770	0.0549510
H	-1.1003660	2.5165800	0.0766690
H	-0.6899370	3.3473320	-1.4391000
H	2.2640670	2.9945370	1.6830850
H	0.8693340	4.0632280	1.9415470
H	0.6476320	2.3065000	1.9223490
H	-2.7842130	0.9420170	0.3155730
H	-4.9973130	2.0319580	0.6317350
H	-7.0441260	0.6407580	0.7884410
H	-6.8921040	-1.8284390	0.6289640
H	-4.6817470	-2.9172170	0.3127700
C	0.8946490	-0.1218960	-1.3918800
H	0.8745050	0.4588310	-2.3137560
C	2.1451100	-0.9345600	-1.2089260
C	2.6134520	-0.6712050	0.0115780
H	2.5621910	-1.6108380	-1.9364740
C	3.8014660	-1.2080120	0.7167620
O	4.1380880	-0.8692370	1.8293370
O	4.4421350	-2.1102950	-0.0350240
C	5.6254460	-2.7064540	0.5496860
H	6.0008590	-3.3955430	-0.2035640
H	6.3677020	-1.9372720	0.7682050
H	5.3655620	-3.2432650	1.4634020

TS *B*+7a→*C'*a (DCM

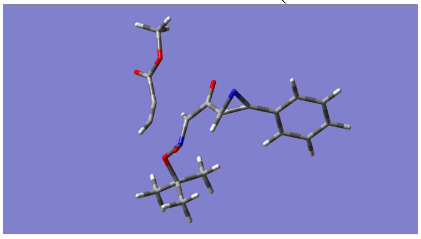

E = -1183.719700, H (0K) = -1183.370068,
H (298K) = -1183.343656,
G (298K) = -1183.429610 au.
Imaginary frequency = 1.

C	2.3580310	0.8393660	0.7250040
N	1.8481880	1.8834190	1.1919570
C	0.9125220	0.6786420	0.9048160
C	0.0414950	0.8136390	-0.2927430
O	0.3199440	1.5629130	-1.2178160
N	-1.5668560	-0.9868290	0.3972560
O	-1.5738000	-0.7866550	1.6561400
O	-2.6059240	-1.7921250	-0.0964670
C	-2.2126140	-3.2184370	-0.2723000
C	-3.5007090	-3.8252320	-0.8162110
C	-1.0746620	-3.3105880	-1.2850700
C	-1.8292560	-3.8257120	1.0742100
C	3.5836100	0.2239490	0.2783500
C	3.5594210	-1.0932730	-0.1990700
C	4.7385160	-1.6913110	-0.6337450
C	5.9370290	-0.9773940	-0.5923870
C	5.9619860	0.3372190	-0.1162090
C	4.7898050	0.9420190	0.3197430
H	0.5253680	0.1519750	1.7651190
H	-3.3519470	-4.8925630	-0.9961470
H	-4.3166710	-3.7044480	-0.0997450
H	-3.7849560	-3.3502950	-1.7582520
H	-0.8578820	-4.3616220	-1.4917080
H	-0.1619530	-2.8484950	-0.9019610
H	-1.3501020	-2.8231870	-2.2235650
H	-2.6369190	-3.7002630	1.7984990
H	-1.6390940	-4.8944690	0.9439800
H	-0.9247600	-3.3658700	1.4769330
H	2.6211700	-1.6355240	-0.2255590
H	4.7240230	-2.7096570	-1.0042930
H	6.8547200	-1.4442450	-0.9323840
H	6.8958410	0.8866930	-0.0880920
H	4.7958200	1.9611600	0.6888000
C	-1.1985300	-0.0041540	-0.4350240
H	-1.5605020	-0.1065290	-1.4498740
C	-2.5618570	0.9230960	1.7884410
C	-2.4321220	1.4866030	0.6901010
H	-2.8933540	0.7906050	2.7970870
C	-2.6764610	2.5759430	-0.2339750
O	-3.4892320	2.5440900	-1.1349870
O	-1.8685140	3.6131820	0.0237090
C	-1.9476710	4.7296340	-0.8950700
H	-2.9457690	5.1698900	-0.8746050
H	-1.2081950	5.4439990	-0.5395840

Molecule ***C'*a** (DCM)

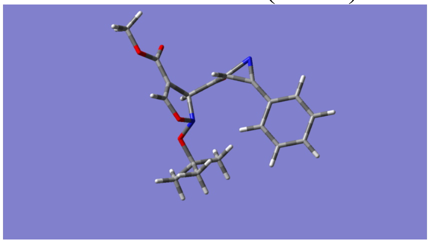

E = -1183.815873, H (0K) = -1183.461661,
H (298K) = -1183.436153,
G (298K) = -1183.518160 au.
Imaginary frequency = 0.

C	-1.4757670	-2.1893900	-0.0595490
N	-0.7646510	-3.1526400	-0.4297640
C	-0.0564550	-1.8398880	-0.0076600
C	0.5175540	-0.9999650	-1.0907100
O	0.2729600	-1.1731030	-2.2669460
N	0.5234020	1.1203690	0.1253690
O	1.0570640	1.0498480	1.5401030
O	0.8105910	2.4029270	-0.3008460
C	-0.3899370	3.2768760	-0.3139770
C	0.1935930	4.6147770	-0.7524790
C	-1.3825780	2.7320170	-1.3374830
C	-0.9927790	3.3573430	1.0853020
C	-2.8147440	-1.7051610	0.1614100
C	-2.9890010	-0.3925730	0.6215180
C	-4.2732120	0.0964170	0.8393300
C	-5.3782140	-0.7216460	0.5989200
C	-5.2043920	-2.0312730	0.1392000
C	-3.9259850	-2.5282880	-0.0818420
H	0.4576430	-1.8478140	0.9472040
H	-0.6053000	5.3567220	-0.8236430
H	0.9338480	4.9695560	-0.0311250
H	0.6710610	4.5256530	-1.7314610
H	-2.2407920	3.4047250	-1.4141340
H	-1.7462540	1.7460880	-1.0436490
H	-0.9132920	2.6534490	-2.3214970
H	-0.2554210	3.7205060	1.8047360
H	-1.8400320	4.0479930	1.0752890
H	-1.3519360	2.3810100	1.4155440
H	-2.1178650	0.2278420	0.7959970
H	-4.4122710	1.1115120	1.1928700
H	-6.3790160	-0.3399390	0.7677060
H	-6.0677210	-2.6595920	-0.0470520
H	-3.7781390	-3.5406470	-0.4400180
C	1.4057580	0.1815370	-0.6498270
H	1.7441850	0.6942590	-1.5499140
C	2.2172660	0.3800190	1.4977570
C	2.5200220	-0.1545430	0.3049110
H	2.7650570	0.3378540	2.4300440
C	3.6856070	-0.9405650	-0.0675990
O	3.8538350	-1.4099650	-1.1786570
O	4.5548190	-1.1007110	0.9500100
C	5.7349140	-1.8850900	0.6654580
H	6.3195510	-1.4208410	-0.1306690
H	6.3029770	-1.8977160	1.5936880

H	-1.7051610	4.4014190	-1.9069530	H	5.4583970	-2.9003200	0.3754480

TS *B* +7g→ *C*g (DCM)

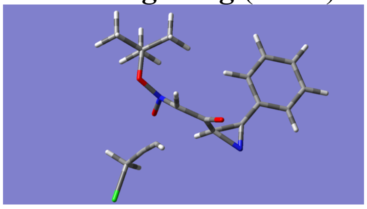

E = -1454.729723, H (0K) = -1454.404044,
H (298K) = -1454.379439,
G (298K) = -1454.461137 au.
Imaginary frequency = 1.

C	2.0989770	-1.6752030	0.0911860
N	1.5321910	-2.7341600	0.4469780
C	0.6519980	-1.4806820	0.2045950
C	0.1240850	-0.8152800	1.4260410
O	0.5522710	-1.0689970	2.5413610
N	-1.2305800	0.8737220	0.1721870
O	-1.4885610	0.2247410	-0.8746510
O	-1.9574010	2.0694500	0.3585670
C	-1.2059550	3.2836910	-0.0585570
C	-2.2192910	4.3864110	0.2262470
C	0.0437660	3.4320250	0.8056660
C	-0.8640510	3.2047920	-1.5440710
C	3.3624760	-1.0483870	-0.2086080
C	3.3843730	0.3023920	-0.5819860
C	4.5980140	0.9188760	-0.8708700
C	5.7853440	0.1899860	-0.7870930
C	5.7643500	-1.1577700	-0.4144440
C	4.5572990	-1.7813790	-0.1241220
H	0.0377290	-1.4749330	-0.6838510
H	-1.7872470	5.3552360	-0.0354750
H	-3.1259950	4.2391170	-0.3653310
H	-2.4876360	4.4010550	1.2853890
H	0.5438240	4.3733060	0.5638470
H	0.7518110	2.6197700	0.6250210
H	-0.2170930	3.4419530	1.8668250
H	-1.7646960	3.0407830	-2.1394860
H	-0.4041320	4.1460180	-1.8569470
H	-0.1590700	2.3969220	-1.7493370
H	2.4535050	0.8547420	-0.6431260
H	4.6193840	1.9632190	-1.1597220
H	6.7305470	0.6716060	-1.0115770
H	6.6903460	-1.7174460	-0.3506170
H	4.5279800	-2.8247620	0.1681050
C	-0.9491050	0.2278330	1.3238300
H	-1.0356740	0.8438650	2.2099310
C	-2.5378600	-1.0787050	1.2973180
C	-2.9149990	-1.2127320	0.1267570
C	-3.5503820	-1.5647620	-1.1150500
H	-2.6254930	-1.3284050	2.3369170
H	-2.8388140	-1.9459240	-1.8450560
H	-4.1098000	-0.7335970	-1.5399710
Cl	-4.7962040	-2.9345680	-0.8819380

Molecule ***C*g** (DCM)

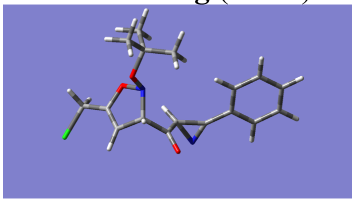

E = -1454.815837, H (0K) = -1454.485560,
H (298K) = -1454.461894,
G (298K) = -1454.541041 au.
Imaginary frequency = 0.

C	-2.1828690	-1.6046750	0.1365930
N	-1.6171530	-2.7182600	0.0422690
C	-0.7314850	-1.4421320	0.0362160
C	-0.1285460	-1.0655660	-1.2703030
O	-0.5853790	-1.4279280	-2.3379020
N	1.1247290	0.7627420	-0.1181300
O	2.0031860	0.1659490	0.9164720
O	1.8006480	1.8941960	-0.5771070
C	1.2499540	3.1449720	-0.0139260
C	2.1647840	4.2025160	-0.6226760
C	-0.1927560	3.3142550	-0.4854600
C	1.3516180	3.1275720	1.5094480
C	-3.4551400	-0.9357370	0.2527500
C	-3.4934310	0.4637990	0.3071700
C	-4.7172980	1.1165150	0.4199370
C	-5.8982370	0.3749430	0.4776020
C	-5.8607890	-1.0220410	0.4222430
C	-4.6435230	-1.6819200	0.3096400
H	-0.1521420	-1.2506530	0.9322080
H	1.8517950	5.1953070	-0.2903670
H	3.2001170	4.0425850	-0.3115150
H	2.1181000	4.1721400	-1.7142440
H	-0.5834020	4.2775010	-0.1469190
H	-0.8287740	2.5254520	-0.0798750
H	-0.2454820	3.2822810	-1.5769660
H	2.3849690	2.9690080	1.8259160
H	1.0073110	4.0859240	1.9074850
H	0.7310090	2.3369470	1.9354650
H	-2.5675140	1.0249680	0.2600820
H	-4.7511170	2.1990010	0.4617620
H	-6.8513080	0.8844900	0.5654720
H	-6.7818800	-1.5916860	0.4659890
H	-4.6010410	-2.7641360	0.2642880
C	1.1583120	-0.2302510	-1.2372570
H	1.2321370	0.3163670	-2.1768470
C	2.3494330	-1.1023030	-0.9338440
C	2.7489000	-0.8150120	0.3010660
C	3.8355410	-1.3241630	1.1830300
H	2.7685580	-1.8317150	-1.6061760
H	3.4234550	-1.6733530	2.1302190
H	4.5653690	-0.5383850	1.3806870
Cl	4.7324910	-2.7150460	0.4434860

TS *B* +7g→ *C'*g (DCM)

Molecule ***C'*g** (DCM)

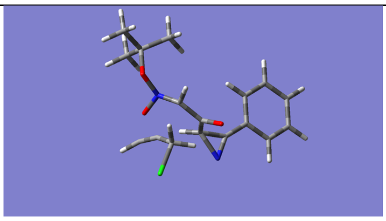

E = -1454.726418, H (0K) = -1454.400277,
H (298K) = -1454.375820,
G (298K) = -1454.456616 au.
Imaginary frequency = 1.

C	-2.3341430	-1.0757040	0.6377460
N	-1.7459790	-2.1042020	1.0431440
C	-0.8956920	-0.8325050	0.7752310
C	-0.0615760	-0.8632170	-0.4559350
O	-0.3432260	-1.5787020	-1.4096090
N	1.5039560	0.9275080	0.3217210
O	1.5862950	0.5924420	1.5454190
O	2.5029750	1.8075680	-0.1411970
C	2.0761390	3.2316620	-0.1386460
C	3.3240010	3.9281060	-0.6699630
C	0.8890100	3.4084080	-1.0820340
C	1.7459090	3.6804170	1.2825000
C	-3.6106070	-0.5191900	0.2621250
C	-3.6810770	0.8126600	-0.1676400
C	-4.9093530	1.3558600	-0.5325280
C	-6.0627600	0.5726460	-0.4686470
C	-5.9932420	-0.7566780	-0.0400280
C	-4.7713340	-1.3071910	0.3257150
H	-0.5101450	-0.3202900	1.6446860
H	3.1452850	5.0045680	-0.7248730
H	4.1754630	3.7510030	-0.0085550
H	3.5732230	3.5662200	-1.6704430
H	0.6370710	4.4694080	-1.1549080
H	0.0073750	2.8777540	-0.7151740
H	1.1309120	3.0409450	-2.0825020
H	2.5871810	3.4907610	1.9525120
H	1.5354170	4.7533060	1.2800550
H	0.8673690	3.1618710	1.6708180
H	-2.7763990	1.4082310	-0.2122150
H	-4.9681170	2.3852700	-0.8663890
H	-7.0191100	0.9967900	-0.7536480
H	-6.8927780	-1.3596350	0.0058720
H	-4.7041120	-2.3368790	0.6577350
C	1.1337680	0.0141700	-0.5913730
H	1.4169850	0.2450980	-1.6100140
C	2.6497720	-1.1215680	1.4096860
C	2.5065460	-1.5278980	0.2466580
C	2.7401470	-2.4366320	-0.8623260
H	2.9962770	-1.0989310	2.4202730
H	3.3316580	-1.9668830	-1.6472860
H	1.8120010	-2.8158600	-1.2867510
Cl	3.7004240	-3.9392010	-0.3525530

TS *B* +7i→*C*i (DCM)

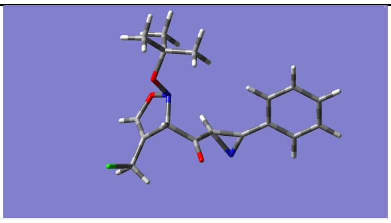

E = -1454.818776, H (0K) = -1454.488077,
H (298K) = -1454.464487,
G (298K) = -1454.542489 au.
Imaginary frequency = 0.

C	2.3077640	-1.3050870	-0.6732640
N	1.8307120	-2.3581150	-1.1550130
C	0.8640120	-1.1730840	-0.8808230
C	-0.0549530	-1.3358050	0.2760570
O	0.1699190	-2.0985870	1.1972690
N	-1.2395530	0.7382740	-0.4750600
O	-1.9119170	0.4850590	-1.7906570
O	-2.0541000	1.6513380	0.1857530
C	-1.5106930	3.0274240	0.1440520
C	-2.5683730	3.8124030	0.9121620
C	-0.1615080	3.0546420	0.8584300
C	-1.3984450	3.5029780	-1.3019610
C	3.5113180	-0.6636670	-0.2048930
C	3.4463380	0.6449360	0.2917940
C	4.6050120	1.2687670	0.7445340
C	5.8231840	0.5889480	0.7022750
C	5.8884590	-0.7175470	0.2075710
C	4.7370040	-1.3480410	-0.2466110
H	0.4949210	-0.6375700	-1.7478920
H	-2.2802040	4.8651350	0.9627770
H	-3.5386200	3.7414360	0.4143980
H	-2.6685990	3.4309350	1.9314570
H	0.2057960	4.0825240	0.9168210
H	0.5752120	2.4580950	0.3176820
H	-0.2569810	2.6616390	1.8741130
H	-2.3663440	3.4430160	-1.8046680
H	-1.0602440	4.5425500	-1.3175660
H	-0.6779020	2.9002100	-1.8581890
H	2.4928130	1.1590790	0.3195550
H	4.5590000	2.2806810	1.1298790
H	6.7250710	1.0760490	1.0559230
H	6.8374520	-1.2403860	0.1789910
H	4.7740180	-2.3609800	-0.6306430
C	-1.3733100	-0.5560910	0.2556020
H	-1.6657930	-0.3452230	1.2842480
C	-2.6351360	-0.6552720	-1.6549500
C	-2.4165230	-1.3299760	-0.5233580
C	-3.0372990	-2.5866320	-0.0899990
H	-3.2934750	-0.8876730	-2.4821890
H	-2.3273360	-3.2834930	0.3504590
H	-3.5966440	-3.0668290	-0.8886180
Cl	-4.2860340	-2.3033030	1.2803140

Molecule ***C*i** (DCM)

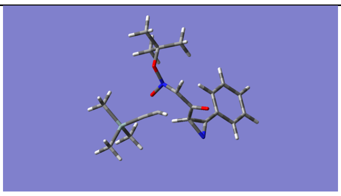

E = -1364.537349, H (0K) = -1364.129748,
H (298K) = -1364.099896,
G (298K) = -1364.192292 au.
Imaginary frequency = 1.

C	2.3781150	-1.5460970	0.3941830
N	1.8875230	-2.5312100	0.9932710
C	0.9502780	-1.3449500	0.6434290
C	0.5392410	-0.4865100	1.7879990
O	1.0845250	-0.5538890	2.8792150
N	-0.9173960	1.0094700	0.4236610
O	-1.2044860	0.2338790	-0.5271620
O	-1.6932090	2.1927800	0.5173370
C	-1.0393100	3.3707150	-0.1007350
C	-2.0903430	4.4562390	0.1068390
C	0.2519100	3.6912920	0.6496380
C	-0.7842700	3.1190630	-1.5853950
C	3.5818990	-0.9780140	-0.1612670
C	3.5268970	0.2860880	-0.7638440
C	4.6819620	0.8446820	-1.3028570
C	5.8875150	0.1442150	-1.2405760
C	5.9433860	-1.1171370	-0.6391800
C	4.7952130	-1.6821650	-0.0981890
H	0.2371120	-1.4989050	-0.1525920
H	-1.7273500	5.4010260	-0.3049710
H	-3.0219620	4.1917690	-0.3991570
H	-2.2954500	4.5979640	1.1708170
H	0.6861510	4.6128170	0.2533030
H	0.9891410	2.8937310	0.5314990
H	0.0554380	3.8324110	1.7154340
H	-1.7102750	2.8448330	-2.0950890
H	-0.3904790	4.0311050	-2.0422370
H	-0.0560450	2.3199780	-1.7351690
H	2.5825340	0.8166510	-0.8054030
H	4.6435450	1.8220190	-1.7697010
H	6.7869030	0.5803500	-1.6608130
H	6.8831200	-1.6553430	-0.5937340
H	4.8259620	-2.6583060	0.3720530
C	-0.5771180	0.5050660	1.6324170
H	-0.6195190	1.2267570	2.4387920
C	-2.0924880	-0.7919880	1.8840710
C	-2.5679340	-1.1354030	0.7833520
H	-2.1136670	-0.8977470	2.9533450
Si	-3.4246710	-1.8120470	-0.7138820
C	-4.6230450	-3.1463290	-0.1382500
H	-5.3747100	-2.7372780	0.5445030
H	-5.1477100	-3.5822120	-0.9960940
H	-4.0967150	-3.9536270	0.3810100
C	-2.1189770	-2.5515250	-1.8446050
H	-2.5929730	-3.0193320	-2.7146160
H	-1.4323810	-1.7796320	-2.2009950

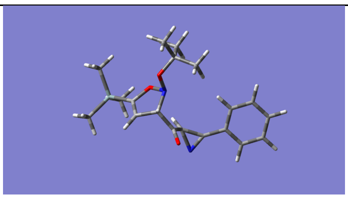

E = -1364.614571, H (0K) = -1364.203139,
H (298K) = -1364.174009,
G (298K) = -1364.264681 au.
Imaginary frequency = 0.

C	2.4209420	-1.7074900	0.1563240
N	1.8966720	-2.7769480	0.5461640
C	0.9950430	-1.5190940	0.4194980
C	0.5613740	-0.8838760	1.6941500
O	1.1170290	-1.0914560	2.7565290
N	-0.6205480	0.8845030	0.4125150
O	-1.5792650	0.2342760	-0.5160400
O	-1.2159110	2.1038120	0.7633370
C	-0.6043310	3.2470130	0.0614220
C	-1.4743390	4.4132990	0.5198080
C	0.8395490	3.4092480	0.5335540
C	-0.6898130	3.0507670	-1.4510390
C	3.6475760	-1.0787360	-0.2678810
C	3.6233410	0.2630640	-0.6710540
C	4.7995420	0.8800630	-1.0862500
C	5.9957440	0.1610420	-1.0964990
C	6.0207980	-1.1779260	-0.6932700
C	4.8509270	-1.8024230	-0.2790360
H	0.2981600	-1.5001590	-0.4107430
H	-1.1056760	5.3441080	0.0818430
H	-2.5108320	4.2676990	0.2054350
H	-1.4488740	4.5095490	1.6083200
H	1.2890340	4.2906970	0.0683540
H	1.4367720	2.5367440	0.2628050
H	0.8754140	3.5332650	1.6192090
H	-1.7253860	2.8945070	-1.7611550
H	-0.3032170	3.9402850	-1.9558560
H	-0.0983760	2.1903830	-1.7695340
H	2.6855010	0.8057910	-0.6582790
H	4.7846240	1.9174280	-1.3996770
H	6.9120600	0.6432190	-1.4184690
H	6.9534720	-1.7300770	-0.7025300
H	4.8578160	-2.8390470	0.0376060
C	-0.6700230	0.0280410	1.6325910
H	-0.6578650	0.6734350	2.5102990
C	-1.9327390	-0.7847290	1.4699430
C	-2.3778230	-0.6236140	0.2201920
H	-2.3651820	-1.3997710	2.2433290
Si	-3.8705100	-1.3597760	-0.6906430
C	-4.7686450	-2.4736250	0.5248160
H	-5.1224290	-1.9119560	1.3949060
H	-5.6406220	-2.9300980	0.0440110
H	-4.1212090	-3.2819780	0.8785580
C	-3.2128170	-2.3281260	-2.1615930
H	-4.0361100	-2.7761320	-2.7284540
H	-2.6513580	-1.6779680	-2.8399610

H	-1.5366380	-3.3200330	-1.3266160
C	-4.3549080	-0.4126020	-1.5517420
H	-3.6598600	0.3570680	-1.8962350
H	-4.9152320	-0.7887330	-2.4147820
H	-5.0671840	0.0535190	-0.8635710

H	-2.5476390	-3.1344400	-1.8364310
C	-4.9499950	0.0690180	-1.2600090
H	-4.3977090	0.7315150	-1.9338440
H	-5.8299500	-0.3017060	-1.7967390
H	-5.2968420	0.6641470	-0.4093630

TS *B* +7i→*C*'a (DCM)

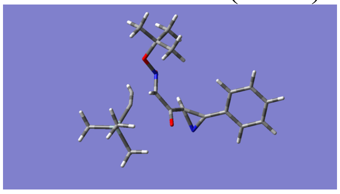

E = -1364.535050, H (0K) = -1364.127021,
H (298K) = -1364.097351,
G (298K) = -1364.189562 au.
Imaginary frequency = 1.

C	2.3270540	-1.4339090	-0.5964040
N	1.6265280	-2.4254860	-0.9050210
C	0.9390180	-1.0351010	-0.8353180
C	0.0554490	-0.8138120	0.3421170
O	0.1209270	-1.5230090	1.3376070
N	-0.8877700	1.3478200	-0.4860070
O	-0.8378270	1.1448370	-1.7422020
O	-1.7234100	2.4179070	-0.0801370
C	-0.9721100	3.6602010	0.2137980
C	-2.0960640	4.6324250	0.5566690
C	-0.0493940	3.4289850	1.4089610
C	-0.1965830	4.1154940	-1.0205060
C	3.6490920	-0.9925540	-0.2246900
C	3.8736010	0.3681420	0.0239930
C	5.1461100	0.8035660	0.3819990
C	6.1904460	-0.1157370	0.4920470
C	5.9670830	-1.4739040	0.2447000
C	4.7000740	-1.9171780	-0.1134440
H	0.6597360	-0.5799100	-1.7740470
H	-1.6729050	5.6076990	0.8091850
H	-2.7712940	4.7559440	-0.2934600
H	-2.6710310	4.2717330	1.4131890
H	0.4401840	4.3676360	1.6812060
H	0.7284860	2.6990460	1.1736590
H	-0.6164540	3.0718440	2.2723250
H	-0.8647250	4.2233380	-1.8776350
H	0.2706150	5.0828030	-0.8166170
H	0.5902810	3.4047360	-1.2804900
H	3.0524230	1.0700290	-0.0671520
H	5.3239320	1.8551630	0.5748990
H	7.1813630	0.2244250	0.7716530
H	6.7823160	-2.1827860	0.3331080
H	4.5139440	-2.9676000	-0.3059480
C	-0.9356560	0.2999520	0.3580830
H	-1.3183160	0.5349790	1.3418290
C	-2.3275100	-0.2855030	-1.9041140
C	-2.4491630	-0.7843480	-0.7678100
H	-2.5006810	-0.1023450	-2.9430130
Si	-3.3507560	-2.0339650	0.2921850
C	-3.4124110	-1.4251690	2.0682540

Molecule ***C*'i** (DCM)

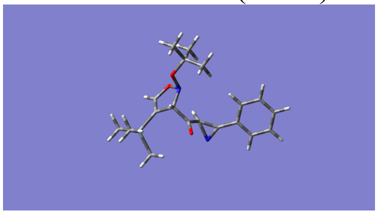

E = -1364.616025, H (0K) = -1364.204103,
H (298K) = -1364.175052,
G (298K) = -1364.265026 au.
Imaginary frequency = 0.

C	2.1142580	-1.6766600	-0.4891430
N	1.3581850	-2.6512650	-0.7084150
C	0.7288760	-1.2319580	-0.6429170
C	-0.0821880	-0.9272200	0.5662350
O	0.0548350	-1.5228750	1.6186500
N	-0.6420480	1.3130040	-0.3526560
O	-1.2889170	1.1787470	-1.6746140
O	-1.2047910	2.4593240	0.2207030
C	-0.2778300	3.6076440	0.1988130
C	-1.1261090	4.7210030	0.8048810
C	0.9334560	3.2892560	1.0735220
C	0.1277850	3.9334630	-1.2368610
C	3.4761690	-1.2766240	-0.2335690
C	3.7691000	0.0805990	-0.0454910
C	5.0800300	0.4753660	0.2041120
C	6.0945570	-0.4810900	0.2657090
C	5.8030070	-1.8359420	0.0775180
C	4.4971430	-2.2389400	-0.1720520
H	0.4063510	-0.8028900	-1.5847330
H	-0.5398430	5.6413280	0.8634540
H	-2.0098730	4.9107940	0.1906290
H	-1.4513660	4.4531520	1.8134310
H	1.5959530	4.1574440	1.1243850
H	1.4984690	2.4507090	0.6624370
H	0.6169030	3.0337090	2.0882800
H	-0.7540660	4.1228060	-1.8531810
H	0.7572570	4.8274660	-1.2440730
H	0.6936110	3.1120020	-1.6805330
H	2.9696870	0.8105030	-0.0965050
H	5.3103020	1.5242080	0.3510340
H	7.1156460	-0.1727600	0.4608700
H	6.5957790	-2.5734750	0.1275320
H	4.2577950	-3.2861880	-0.3174000
C	-1.1644190	0.1481780	0.4287540
H	-1.4195660	0.4984220	1.4286180
C	-2.3101800	0.2756740	-1.5294180
C	-2.3541480	-0.3751600	-0.3640550
H	-2.9546150	0.2044860	-2.3964190
Si	-3.6178150	-1.6501050	0.1848350
C	-4.3477310	-1.0908630	1.8282640

H	-4.0205660	-2.1021870	2.6782090
H	-2.4089590	-1.3888930	2.5013200
H	-3.8549150	-0.4258620	2.1323800
C	-2.4134670	-3.6581590	0.1747660
H	-2.9199680	-4.4322180	0.7621050
H	-2.3541610	-4.0027960	-0.8623720
H	-1.3949890	-3.5501590	0.5574560
C	-5.0892410	-2.2066240	-0.4054430
H	-5.6626520	-2.9401420	0.1723400
H	-5.6251850	-1.2528850	-0.3682340
H	-5.0626960	-2.5408840	-1.4473610

H	-5.1020030	-1.8032770	2.1800410
H	-3.5723500	-1.0209870	2.5984260
H	-4.8243770	-0.1093420	1.7380470
C	-2.7747820	-3.3196110	0.4045300
H	-3.5061870	-4.0814970	0.6965020
H	-2.2966160	-3.6473810	-0.5241980
H	-2.0050600	-3.2768540	1.1805970
C	-4.9460050	-1.7556160	-1.1441840
H	-5.7091440	-2.4874020	-0.8576020
H	-5.4450270	-0.7925770	-1.2916540
H	-4.5261680	-2.0736560	-2.1039810

TS *B* +7k→*C*k (DCM)

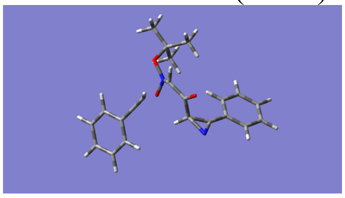

E = -1186.903186, H (0K) = -1186.515941,
H (298K) = -1186.489227,
G (298K) = -1186.575950 au.
Imaginary frequency = 1.

C	2.0799340	-1.8764670	0.5463220
N	1.5747480	-2.7185610	1.3267950
C	0.7298800	-1.4797300	0.9388100
C	0.5747780	-0.4356480	1.9895420
O	1.1386580	-0.5083610	3.0698290
N	-0.5077990	1.2052720	0.4684320
O	-0.9858930	0.4306250	-0.3964510
O	-0.9138810	2.5604690	0.3982870
C	0.0634630	3.4289170	-0.3069110
C	-0.6418450	4.7803510	-0.2752900
C	1.3754650	3.4633180	0.4738870
C	0.2699970	2.9379960	-1.7378650
C	3.2429070	-1.5085830	-0.2218730
C	3.2131860	-0.3220740	-0.9678790
C	4.3276160	0.0539830	-1.7112730
C	5.4676770	-0.7513210	-1.7098160
C	5.4979660	-1.9352860	-0.9659570
C	4.3898360	-2.3183770	-0.2204490
H	-0.0913880	-1.6269040	0.2521630
H	-0.0155320	5.5310140	-0.7634100
H	-1.5973460	4.7288290	-0.8027390
H	-0.8241020	5.0973460	0.7545030
H	2.0597860	4.1756520	0.0058970
H	1.8616130	2.4849960	0.4792040
H	1.2041430	3.7761670	1.5068900
H	-0.6841410	2.8659850	-2.2639850
H	0.9120110	3.6446860	-2.2706890
H	0.7515980	1.9586040	-1.7576130
H	2.3185770	0.2903680	-0.9598340
H	4.3082850	0.9703920	-2.2896090
H	6.3361560	-0.4579020	-2.2889800
H	6.3872300	-2.5550360	-0.9691750
H	4.4021750	-3.2323820	0.3621360
C	-0.2730920	0.7749980	1.7269460

Molecule ***C*k** (DCM)

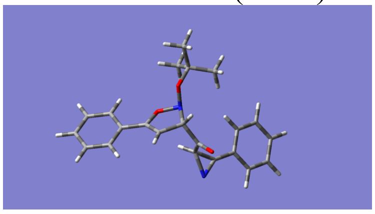

E = -1186.991672, H (0K) = -1186.599604,
H (298K) = -1186.573960,
G (298K) = -1186.657097 au.
Imaginary frequency = 0.

C	2.4157750	-1.8420840	0.1158340
N	1.8290190	-2.8593390	0.5529220
C	1.0238500	-1.5357270	0.4448770
C	0.7094210	-0.8459380	1.7252090
O	1.3069060	-1.0708070	2.7607030
N	-0.4288030	0.9762500	0.4646420
O	-1.4668700	0.3750150	-0.3830240
O	-0.9175610	2.2419810	0.8162200
C	-0.2586280	3.3239740	0.0594850
C	-1.0063950	4.5614260	0.5454860
C	1.2182830	3.3766820	0.4461300
C	-0.4466540	3.1127730	-1.4413140
C	3.6643920	-1.3176400	-0.3795150
C	3.7277570	0.0208320	-0.7894750
C	4.9261660	0.5389100	-1.2710450
C	6.0571970	-0.2757540	-1.3416370
C	5.9946590	-1.6117170	-0.9324520
C	4.8021080	-2.1376200	-0.4514420
H	0.2898200	-1.4758820	-0.3507610
H	-0.5939140	5.4532950	0.0674160
H	-2.0682810	4.4929720	0.2964260
H	-0.9070270	4.6721130	1.6283490
H	1.7060200	4.2170310	-0.0550610
H	1.7300370	2.4586210	0.1517560
H	1.3266280	3.5063330	1.5262840
H	-1.5078220	3.0450860	-1.6912480
H	-0.0132620	3.9567790	-1.9846630
H	0.0494370	2.1992790	-1.7746520
H	2.8394250	0.6385750	-0.7296310
H	4.9790110	1.5735390	-1.5894700
H	6.9910130	0.1289380	-1.7157050
H	6.8773810	-2.2381980	-0.9888720
H	4.7412630	-3.1708860	-0.1294540
C	-0.4571460	0.1536630	1.7129140

H	-0.1178000	1.5699320	2.4454140
C	-2.0623140	-0.0612670	2.1388320
C	-2.6628140	-0.4405150	1.1218360
H	-2.0794650	0.0078300	3.2105850
C	-3.5101780	-0.9895740	0.1241550
C	-3.4126770	-2.3544970	-0.2107410
C	-4.4627870	-0.1878710	-0.5355210
C	-4.2518780	-2.8988850	-1.1779110
H	-2.6799700	-2.9750690	0.2918280
C	-5.2991590	-0.7447260	-1.4954080
H	-4.5321090	0.8653080	-0.2900470
C	-5.1976180	-2.0997860	-1.8226710
H	-4.1679700	-3.9508710	-1.4274930
H	-6.0312510	-0.1194040	-1.9944720
H	-5.8502770	-2.5280890	-2.5750510

H	-0.3363180	0.8205610	2.5660430
C	-1.7806910	-0.5566260	1.6570990
C	-2.2840480	-0.3936930	0.4278180
H	-2.2241060	-1.0944120	2.4786780
C	-3.5150680	-0.8807510	-0.1975390
C	-4.2507260	-1.9214830	0.3932680
C	-3.9772200	-0.3113950	-1.3938350
C	-5.4290050	-2.3688010	-0.1936940
H	-3.8945230	-2.3852040	1.3058220
C	-5.1563770	-0.7675630	-1.9797290
H	-3.4153030	0.4892240	-1.8578450
C	-5.8867730	-1.7940750	-1.3824290
H	-5.9880510	-3.1729090	0.2716890
H	-5.5044010	-0.3184020	-2.9032730
H	-6.8035860	-2.1486950	-1.8400490

TS *B* +7k→*C'*k (DCM)

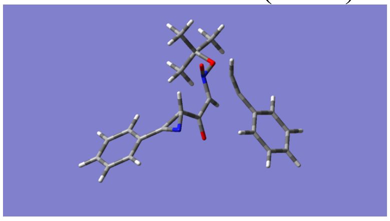

E = -1186.898331, H (0K) = -1186.510887,
H (298K) = -1186.484326,
G (298K) = -1186.571019 au.
Imaginary frequency = 1.

C	-2.4776760	-0.9953340	0.8046850
N	-1.7883360	-1.8342950	1.4300630
C	-1.1209720	-0.4917960	1.0222300
C	-0.1559740	-0.5680680	-0.1110760
O	-0.2310580	-1.4498260	-0.9587920
N	1.0388370	1.5534280	0.4715640
O	0.9818750	1.4662670	1.7437570
O	2.0036890	2.4521070	-0.0310260
C	1.4369550	3.7796060	-0.3780480
C	2.6692820	4.5188840	-0.8881090
C	0.3870230	3.6149500	-1.4744250
C	0.8586320	4.4478380	0.8670050
C	-3.7741930	-0.7354850	0.2275430
C	-4.0002230	0.4778170	-0.4361220
C	-5.2471190	0.7354920	-0.9982510
C	-6.2649320	-0.2141450	-0.8981310
C	-6.0403510	-1.4253640	-0.2361380
C	-4.7984960	-1.6909940	0.3271550
H	-0.9196900	0.2051480	1.8226480
H	2.3905490	5.5328820	-1.1847880
H	3.4307830	4.5830120	-0.1071180
H	3.0957160	4.0084500	-1.7551300
H	0.0406550	4.5997520	-1.7982580
H	-0.4790510	3.0549590	-1.1138490
H	0.8077430	3.0949410	-2.3387430
H	1.6098620	4.5063440	1.6575950
H	0.5366190	5.4618610	0.6147630
H	-0.0055220	3.9002540	1.2476050
H	-3.1989970	1.2048900	-0.5071700

Molecule ***C'*k** (DCM)

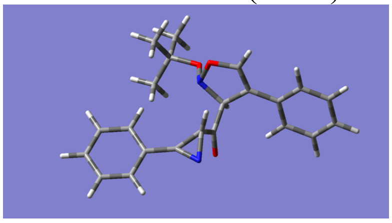

E = -1186.988663, H (0K) = -1186.595989,
H (298K) = -1186.570465,
G (298K) = -1186.652529 au.
Imaginary frequency = 0.

C	1.7377240	-2.1178570	-0.3674000
N	0.8620330	-3.0133180	-0.4120660
C	0.4074960	-1.5353560	-0.5369570
C	-0.2885060	-0.9488600	0.6384020
O	-0.2670550	-1.4573440	1.7423380
N	-0.1416930	1.3260150	-0.2864350
O	-0.6403470	1.3672540	-1.6805310
O	-0.4134330	2.5911780	0.2387800
C	0.8100920	3.4078410	0.3858800
C	0.2515410	4.7452440	0.8600270
C	1.7070170	2.7690990	1.4444660
C	1.5209470	3.5436260	-0.9583720
C	3.1444530	-1.8355430	-0.2288710
C	3.5801760	-0.5056240	-0.3043060
C	4.9346640	-0.2153530	-0.1731230
C	5.8500910	-1.2485550	0.0331690
C	5.4156350	-2.5759060	0.1081990
C	4.0652460	-2.8752970	-0.0222770
H	0.0958210	-1.2011890	-1.5201660
H	1.0716080	5.4460990	1.0341800
H	-0.4175410	5.1724990	0.1090010
H	-0.3025190	4.6226360	1.7941470
H	2.5883330	3.3936390	1.6132040
H	2.0444130	1.7811390	1.1260010
H	1.1685500	2.6660860	2.3902450
H	0.8508830	3.9694800	-1.7086520
H	2.3849730	4.2042130	-0.8474450
H	1.8743980	2.5745870	-1.3158330
H	2.8547480	0.2829390	-0.4656000

H	-5.4254520	1.6722170	-1.5135070
H	-7.2357330	-0.0126720	-1.3371870
H	-6.8348710	-2.1590810	-0.1628790
H	-4.6114030	-2.6270590	0.8409240
C	0.9208880	0.4376160	-0.2694710
H	1.3470180	0.4996780	-1.2615620
C	2.1986910	-0.0510720	2.1084380
C	2.3055770	-0.7362040	1.0758740
H	2.3965240	0.2191240	3.1242070
C	2.7971830	-1.8478830	0.3025220
C	2.1997430	-3.1131730	0.4241750
C	3.8650660	-1.6769470	-0.5951760
C	2.6763860	-4.1870720	-0.3242170
H	1.3650670	-3.2459290	1.1017800
C	4.3364090	-2.7563880	-1.3357530
H	4.3173450	-0.6978920	-0.7052380
C	3.7446290	-4.0150630	-1.2044760
H	2.2073460	-5.1593840	-0.2206290
H	5.1651820	-2.6143330	-2.0207920
H	4.1115980	-4.8527680	-1.7869790

H	5.2756700	0.8118050	-0.2304440
H	6.9053790	-1.0216000	0.1365050
H	6.1323840	-3.3728610	0.2693350
H	3.7158240	-3.8996810	0.0363090
C	-1.0549110	0.3639820	0.4014600
H	-1.3140240	0.7813470	1.3739370
C	-1.8872030	0.8120710	-1.6783210
C	-2.2402430	0.2118430	-0.5344530
H	-2.4242070	0.9212760	-2.6101230
C	-3.4900880	-0.4653250	-0.1901920
C	-4.5399740	-0.5898070	-1.1217100
C	-3.6737810	-1.0162250	1.0896170
C	-5.7217930	-1.2363030	-0.7813580
H	-4.4313940	-0.1787210	-2.1189380
C	-4.8621990	-1.6635350	1.4271940
H	-2.8846120	-0.9485270	1.8286520
C	-5.8923170	-1.7775840	0.4966180
H	-6.5156590	-1.3195410	-1.5158580
H	-4.9774720	-2.0806300	2.4217340
H	-6.8156540	-2.2820280	0.7584880

TS *B* +7l→*C*l (DCM)

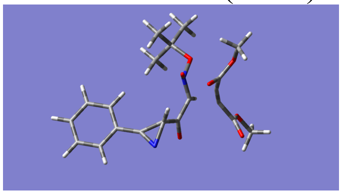

E = -1411.672216, H (0K) = -1411.279986,
H (298K) = -1411.248679,
G (298K) = -1411.347445 au.
Imaginary frequency = 1.

C	2.5259160	-1.5309510	-0.7726630
N	1.9106720	-2.5996590	-0.9924500
C	1.0774670	-1.3296410	-0.6900390
C	0.4526790	-1.2750980	0.6570120
O	0.7045170	-2.0872770	1.5316200
N	-0.5864100	0.9798870	0.3143380
O	-0.7464600	0.9571210	-0.9291010
O	-1.1946910	2.0322290	1.0112570
C	-0.2885760	3.2014690	1.2293230
C	-1.1873600	4.1337530	2.0318940
C	0.9278980	2.7546670	2.0343540
C	0.1030520	3.8129380	-0.1124410
C	3.8167840	-0.9017250	-0.6496320
C	3.8825080	0.4635030	-0.3392510
C	5.1217070	1.0834840	-0.2129730
C	6.2907790	0.3432040	-0.3958080
C	6.2258500	-1.0190290	-0.7055920
C	4.9929120	-1.6465040	-0.8331710
H	0.5497140	-0.8757300	-1.5164110
H	-0.6424180	5.0520190	2.2629800
H	-2.0806530	4.3951250	1.4598000
H	-1.4926910	3.6649590	2.9702860
H	1.5328400	3.6287560	2.2877990
H	1.5567040	2.0675780	1.4627160
H	0.6223910	2.2650020	2.9621740

Molecule ***C*l** (DCM)

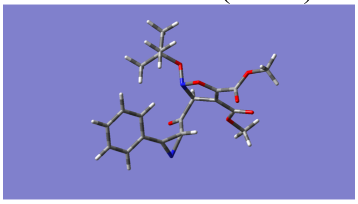

E = -1411.760961, H (0K) = -1411.364843,
H (298K) = -1411.334427,
G (298K) = -1411.429047 au.
Imaginary frequency = 0.

C	2.1124520	-2.1101610	-0.2910440
N	1.5921880	-3.1974590	0.0511270
C	0.6862440	-1.9412740	-0.0106110
C	0.2804590	-1.3333760	1.2809830
O	0.7647360	-1.6449450	2.3492420
N	-0.0562220	0.9724390	0.5605930
O	-0.8048570	1.1642010	-0.7601540
O	-0.3425240	2.0929550	1.2997360
C	0.7782860	3.0739470	1.3064330
C	0.1973740	4.2114670	2.1371840
C	1.9837770	2.4327080	1.9872560
C	1.0892070	3.5175600	-0.1194310
C	3.3177170	-1.4259140	-0.6834650
C	3.2591320	-0.0461760	-0.9248340
C	4.4121150	0.6362240	-1.2999360
C	5.6164740	-0.0560880	-1.4353610
C	5.6745480	-1.4327750	-1.1944900
C	4.5292850	-2.1228850	-0.8175060
H	-0.0128770	-1.8816900	-0.8377210
H	0.9397850	5.0067890	2.2366850
H	-0.6930770	4.6253950	1.6577620
H	-0.0713300	3.8621990	3.1372290
H	2.7907730	3.1651120	2.0712530
H	2.3532260	1.5818470	1.4124570
H	1.7193710	2.0902250	2.9909620

H	-0.7852250	4.0656840	-0.6951400
H	0.6758790	4.7268220	0.0663690
H	0.7230150	3.1326930	-0.6990210
H	2.9648810	1.0241410	-0.2013190
H	5.1770380	2.1389910	0.0267530
H	7.2561580	0.8268640	-0.2968830
H	7.1380740	-1.5873520	-0.8457520
H	4.9290620	-2.7021020	-1.0711050
C	-0.5101490	-0.1746190	1.0112500
H	-0.6704750	-0.0619380	2.0751300
C	-2.1898750	-1.0346420	0.1642030
C	-2.3967020	-0.4681210	-0.9234880
C	-2.9024860	-0.1157110	-2.2264670
O	-2.3847620	-0.4712650	-3.2644750
O	-3.9961710	0.6513830	-2.1354240
C	-4.5819910	1.0773680	-3.3917480
H	-5.4433740	1.6806410	-3.1136750
H	-3.8645980	1.6721250	-3.9592470
H	-4.8939520	0.2109000	-3.9767500
C	-2.6468420	-2.1105600	1.0534780
O	-2.9156000	-3.2254260	0.6689420
O	-2.7216840	-1.6990280	2.3216520
C	-3.1618530	-2.6846580	3.2905130
H	-3.1592170	-2.1655220	4.2461910
H	-4.1665070	-3.0318140	3.0454720
H	-2.4679790	-3.5260920	3.3081250

H	0.2015870	3.9379310	-0.5972420
H	1.8687840	4.2833910	-0.0957170
H	1.4495920	2.6828050	-0.7232060
H	2.3148580	0.4726760	-0.8075090
H	4.3731350	1.7033860	-1.4851930
H	6.5145050	0.4764950	-1.7281990
H	6.6140360	-1.9626850	-1.3009050
H	4.5612260	-3.1892680	-0.6258970
C	-0.7558340	-0.1907030	1.2110040
H	-1.0078880	0.0914320	2.2330980
C	-1.9621960	-0.4409750	0.3462040
C	-1.8818130	0.3700190	-0.7193010
C	-2.7831640	0.4565460	-1.9221250
O	-2.5286780	-0.1038560	-2.9599970
O	-3.8261200	1.2353860	-1.6796620
C	-4.7899730	1.3807100	-2.7591490
H	-5.5537350	2.0489780	-2.3692130
H	-4.3071520	1.8146850	-3.6354120
H	-5.2167440	0.4075680	-3.0046160
C	-3.0118580	-1.4252880	0.5782100
O	-3.9577790	-1.6257980	-0.1604020
O	-2.7853190	-2.1047990	1.7167840
C	-3.7334160	-3.1450720	2.0492930
H	-3.3778250	-3.5692240	2.9859080
H	-4.7315240	-2.7227210	2.1756120
H	-3.7490600	-3.9063080	1.2675690

TS *B* +7k→*C*k (DMF)

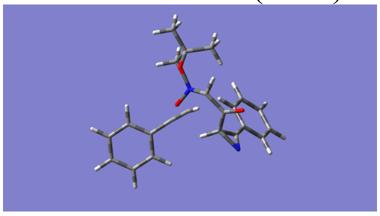

E = -1186.898617, H (0K) = -1186.511326,
H (298K) = -1186.484691,
G (298K) = -1186.570700 au.
Imaginary frequency = 1.

C	2.0373890	-1.9049810	0.5359760
N	1.5191370	-2.7502420	1.3052480
C	0.6899810	-1.4977010	0.9251730
C	0.5462800	-0.4556660	1.9802330
O	1.1019690	-0.5424850	3.0635940
N	-0.5064690	1.1980790	0.4523550
O	-1.0129560	0.4326310	-0.4034030
O	-0.8582900	2.5670890	0.3687310
C	0.1734050	3.3910260	-0.3155760
C	-0.4567030	4.7781240	-0.2741130
C	1.4787220	3.3450880	0.4751500
C	0.3657680	2.9049190	-1.7494440
C	3.2092040	-1.5364790	-0.2171580
C	3.2031300	-0.3254140	-0.9233380
C	4.3272380	0.0545950	-1.6499130
C	5.4525310	-0.7710430	-1.6715230
C	5.4589460	-1.9794210	-0.9670260
C	4.3414130	-2.3666600	-0.2379440
H	-0.1292000	-1.6300950	0.2327780

Molecule ***C*k** (DMF)

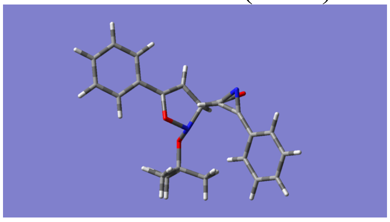

E = -1186.986515, H (0K) = -1186.594584,
H (298K) = -1186.568918,
G (298K) = -1186.652249 au.
Imaginary frequency = 0.

C	2.4065820	-1.8495660	0.1072540
N	1.8167040	-2.8690510	0.5359620
C	1.0158800	-1.5416370	0.4390120
C	0.7035080	-0.8588010	1.7240760
O	1.2937300	-1.0975180	2.7605580
N	-0.4211370	0.9729980	0.4647890
O	-1.4617370	0.3740590	-0.3857970
O	-0.9072170	2.2395600	0.8157050
C	-0.2404510	3.3193850	0.0610910
C	-0.9935860	4.5589520	0.5316450
C	1.2313970	3.3742610	0.4645560
C	-0.4103540	3.1018170	-1.4405250
C	3.6558950	-1.3227080	-0.3820150
C	3.7230710	0.0234070	-0.7657840
C	4.9223390	0.5457560	-1.2403970
C	6.0496140	-0.2723480	-1.3305140
C	5.9828630	-1.6160860	-0.9478030
C	4.7896710	-2.1463820	-0.4733800

H	0.2123240	5.4965400	-0.7539490
H	-1.4115150	4.7849040	-0.8056170
H	-0.6244670	5.0965530	0.7578020
H	2.1993320	4.0315220	0.0231780
H	1.9164060	2.3442670	0.4647570
H	1.3157990	3.6484590	1.5124180
H	-0.5849860	2.8987010	-2.2870300
H	1.0560730	3.5769140	-2.2665350
H	0.7877500	1.8985880	-1.7747430
H	2.3211690	0.3041420	-0.8952490
H	4.3266360	0.9902940	-2.1968650
H	6.3283670	-0.4745280	-2.2379530
H	6.3369760	-2.6147120	-0.9879820
H	4.3356790	-3.2997020	0.3139300
C	-0.2767040	0.7708080	1.7132650
H	-0.0987750	1.5674790	2.4243770
C	-2.0741410	-0.0262910	2.1374800
C	-2.6878000	-0.3965160	1.1245250
H	-2.0823510	0.0373670	3.2101140
C	-3.5462910	-0.9314800	0.1293120
C	-3.3895410	-2.2659190	-0.2950100
C	-4.5665410	-0.1456200	-0.4424720
C	-4.2367110	-2.7959470	-1.2630140
H	-2.6038490	-2.8736880	0.1385560
C	-5.4086930	-0.6880350	-1.4063250
H	-4.6858740	0.8841070	-0.1261500
C	-5.2482710	-2.0125300	-1.8223910
H	-4.1069660	-3.8244910	-1.5815210
H	-6.1923620	-0.0748640	-1.8376860
H	-5.9062880	-2.4296510	-2.5764290

H	0.2839270	-1.4718570	-0.3578340
H	-0.5726420	5.4479740	0.0556450
H	-2.0520110	4.4915370	0.2673700
H	-0.9091500	4.6746450	1.6153670
H	1.7252600	4.2097850	-0.0386920
H	1.7462180	2.4534600	0.1841720
H	1.3272970	3.5138830	1.5447580
H	-1.4681600	3.0278170	-1.7028870
H	0.0252870	3.9466380	-1.9807600
H	0.0949060	2.1902260	-1.7651990
H	2.8373400	0.6432430	-0.6909300
H	4.9787380	1.5862790	-1.5384170
H	6.9839730	0.1356500	-1.6996980
H	6.8626840	-2.2450580	-1.0200060
H	4.7254890	-3.1859460	-0.1726390
C	-0.4548380	0.1498020	1.7120990
H	-0.3320270	0.8152600	2.5658530
C	-1.7814460	-0.5546910	1.6550230
C	-2.2820710	-0.3893350	0.4247140
H	-2.2284260	-1.0911370	2.4757860
C	-3.5164100	-0.8703920	-0.1990520
C	-4.2606570	-1.9014440	0.3982900
C	-3.9738840	-0.3045440	-1.3989590
C	-5.4420640	-2.3432850	-0.1866620
H	-3.9097170	-2.3611050	1.3149820
C	-5.1563380	-0.7554050	-1.9827120
H	-3.4071240	0.4897130	-1.8682340
C	-5.8947820	-1.7727040	-1.3794740
H	-6.0079610	-3.1396400	0.2838550
H	-5.5005440	-0.3090530	-2.9090850
H	-6.8141730	-2.1230610	-1.8352880

TS *B* +7k→*C'*k (DMF)

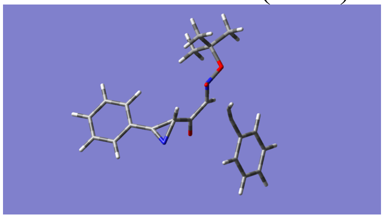

E = -1186.893241, H (0K) = -1186.505734,
H (298K) = -1186.479199,
G (298K) = -1186.565546 au.
Imaginary frequency = 1.

C	-2.4040180	-1.1778570	0.7858770
N	-1.6837730	-2.0116430	1.3833350
C	-1.0692960	-0.6294060	1.0275750
C	-0.0988930	-0.6226290	-0.1039990
O	-0.1104310	-1.4941260	-0.9645710
N	0.9217250	1.5787590	0.5096780
O	0.8671540	1.4743860	1.7793380
O	1.8028060	2.5668020	0.0200720
C	1.1089530	3.8261320	-0.3526620
C	2.2688830	4.6919160	-0.8305010
C	0.1160730	3.5478380	-1.4782530
C	0.4251690	4.4316800	0.8701250
C	-3.7066010	-0.9446980	0.2127060
C	-3.9678440	0.2783540	-0.4195940

Molecule ***C'*k** (DMF)

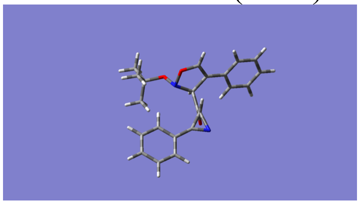

E = -1186.984093, H (0K) = -1186.591819,
H (298K) = -1186.566166,
G (298K) = -1186.649484 au.
Imaginary frequency = 0.

C	1.6332900	-2.1971870	-0.2644270
N	0.7610030	-3.0956230	-0.1999600
C	0.2943230	-1.6332750	-0.4246490
C	-0.3465690	-0.9435890	0.7262450
O	-0.3119550	-1.3759470	1.8617050
N	-0.0767160	1.2825830	-0.2712360
O	-0.5423940	1.3166740	-1.6802900
O	-0.2892740	2.5731240	0.2168360
C	0.9771360	3.3185030	0.3851320
C	0.4884280	4.6972510	0.8146560
C	1.8014440	2.6504830	1.4835380
C	1.7351340	3.3827910	-0.9381580
C	3.0399580	-1.8921310	-0.2041780
C	3.4480080	-0.5608610	-0.3654470

C	-5.2212320	0.5119460	-0.9778150
C	-6.2098930	-0.4704650	-0.9038900
C	-5.9498810	-1.6911100	-0.2725860
C	-4.7012230	-1.9335340	0.2859900
H	-0.9039880	0.0489220	1.8518340
H	1.8902850	5.6677540	-1.1439190
H	2.9944860	4.8433330	-0.0274770
H	2.7747770	4.2271160	-1.6804230
H	-0.3208310	4.4901460	-1.8185920
H	-0.6973010	2.9015640	-1.1401130
H	0.6152410	3.0715110	-2.3259740
H	1.1419480	4.5760880	1.6816270
H	0.0017460	5.4030700	0.6007460
H	-0.3853950	3.7944130	1.2288300
H	-3.1889390	1.0310180	-0.4692480
H	-5.4273730	1.4558310	-1.4691110
H	-7.1860630	-0.2870540	-1.3390140
H	-6.7224140	-2.4496430	-0.2188880
H	-4.4873440	-2.8759860	0.7773840
C	0.9024920	0.4646110	-0.2430580
H	1.3200080	0.5761040	-1.2346560
C	2.2134660	0.0468130	2.1231930
C	2.3698580	-0.5984830	1.0712080
H	2.3921620	0.3193730	3.1419580
C	2.9475250	-1.6599550	0.2840130
C	2.6339080	-2.9987960	0.5696270
C	3.8141480	-1.3727080	-0.7837790
C	3.1897040	-4.0265280	-0.1893620
H	1.9537120	-3.2247250	1.3824910
C	4.3723750	-2.4054960	-1.5309050
H	4.0435920	-0.3401910	-1.0212830
C	4.0617040	-3.7358450	-1.2389570
H	2.9389210	-5.0564030	0.0404040
H	5.0458720	-2.1714530	-2.3481830
H	4.4927420	-4.5377740	-1.8279210

C	4.8013250	-0.2432940	-0.3064730
C	5.7426080	-1.2506040	-0.0884690
C	5.3354810	-2.5793880	0.0714160
C	3.9863820	-2.9061780	0.0148460
H	-0.0571200	-1.3796250	-1.4186970
H	1.3456450	5.3486950	1.0013720
H	-0.1282810	5.1498920	0.0340410
H	-0.1006080	4.6303300	1.7330550
H	2.7162410	3.2217900	1.6622810
H	2.0832390	1.6354700	1.1980600
H	1.2317560	2.6067250	2.4157640
H	1.1150650	3.8320570	-1.7174130
H	2.6328960	3.9937100	-0.8115020
H	2.0427380	2.3878840	-1.2653730
H	2.7020970	0.2071420	-0.5319940
H	5.1208330	0.7851720	-0.4286850
H	6.7972240	-1.0026040	-0.0420590
H	6.0726920	-3.3558380	0.2403780
H	3.6584190	-3.9319780	0.1388380
C	-1.0556170	0.3876140	0.4184570
H	-1.3248110	0.8495140	1.3677150
C	-1.8080860	0.8122690	-1.6973030
C	-2.2164090	0.2571750	-0.5487090
H	-2.3180190	0.9159420	-2.6452110
C	-3.4980900	-0.3671840	-0.2251710
C	-4.5009330	-0.5404640	-1.2000560
C	-3.7603560	-0.8187880	1.0798610
C	-5.7141180	-1.1355090	-0.8758300
H	-4.3302190	-0.2094290	-2.2182020
C	-4.9792580	-1.4164500	1.4004380
H	-3.0116050	-0.7058070	1.8547140
C	-5.9628820	-1.5780730	0.4272560
H	-6.4706460	-1.2576270	-1.6436190
H	-5.1556690	-1.7559170	2.4154360
H	-6.9100880	-2.0437100	0.6756060

Molecule **1a** (DMF)

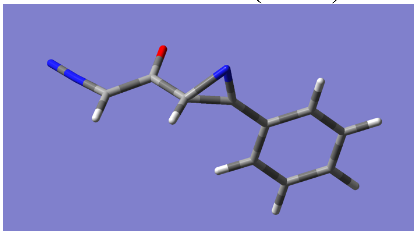

E = -624.840978, H (0K) = -624.690582,
H (298K) = -624.677624,
G (298K) = -624.730922 au.
Imaginary frequency = 0.

C	-0.4098030	1.0333940	-0.3474550
N	0.1519320	2.1423840	-0.1729160
C	0.9733960	0.9962260	-0.8132140
C	2.0208240	0.3671610	0.0492790
O	1.9149570	0.3038310	1.2725320
C	-1.6426880	0.3079710	-0.1618550
C	-1.7107350	-1.0412090	-0.5346980
C	-2.8966070	-1.7474720	-0.3548560
C	-4.0103930	-1.1095040	0.1933370

Molecule **TBN** (DMF)

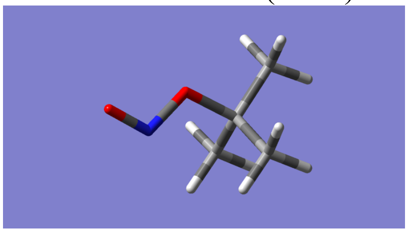

E = -363.088280, H (0K) = -362.956884,
H (298K) = -362.947569,
G (298K) = -362.988995 au.
Imaginary frequency = 0.

N	-1.6148300	-0.2949190	0.0004740
O	-2.6763680	0.2386470	-0.0004830
O	-0.5927160	0.6205440	-0.0009340
C	0.7661600	-0.0115150	0.0000010
C	1.6906110	1.1986330	-0.0009410
C	0.9234810	-0.8416200	1.2700050
C	0.9242380	-0.8441880	-1.2682480
H	2.7289660	0.8584270	-0.0002690
H	1.5275780	1.8116620	-0.8906350

C	-3.9435580	0.2372080	0.5648470
C	-2.7635050	0.9492810	0.3904890
H	1.1940270	1.0953780	-1.8719980
H	-0.8368950	-1.5232860	-0.9581970
H	-2.9530390	-2.7915470	-0.6402460
H	-4.9336520	-1.6608720	0.3327040
H	-4.8122920	0.7264870	0.9901470
H	-2.6992740	1.9932920	0.6755350
C	3.1539140	-0.1618360	-0.6892200
H	3.2924700	-0.1070690	-1.7593910
N	4.0944370	-0.7597150	-0.0030450
N	4.8684780	-1.2612910	0.6432840

H	1.5270220	1.8134490	0.8874190
H	1.9472770	-1.2200020	1.3264540
H	0.2423120	-1.6950250	1.2744630
H	0.7319640	-0.2304240	2.1557090
H	0.7330730	-0.2348240	-2.1552890
H	1.9481250	-1.2225080	-1.3234180
H	0.2432290	-1.6977170	-1.2713280

TS 1a+TBN→*Bconformer*+ N_2

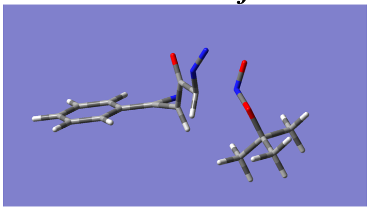

E = -987.891331, H (0K) = -987.607874,
H (298K) = -987.586417,
G (298K) = -987.659155 au.
Imaginary frequency = 1.

C	-2.1814540	-0.4673280	-0.7737690
N	-1.5043350	-0.5843650	-1.8207080
C	-0.7460410	-0.3744850	-0.4843400
C	-0.1443760	0.9620770	-0.2777280
O	-0.5673970	1.9893910	-0.7671890
C	-3.5096720	-0.4339440	-0.2179360
C	-3.6731110	-0.2223910	1.1578710
C	-4.9538140	-0.1880070	1.7007890
C	-6.0647780	-0.3644600	0.8744230
C	-5.9015190	-0.5756360	-0.4984690
C	-4.6274450	-0.6106930	-1.0502710
H	-0.2172400	-1.2305380	-0.0823100
H	-2.7997310	-0.0857510	1.7853730
H	-5.0864900	-0.0235440	2.7637540
H	-7.0620760	-0.3374970	1.2991620
H	-6.7691530	-0.7115570	-1.1336740
H	-4.4875540	-0.7731380	-2.1128920
C	1.1256210	0.9447560	0.5590940
H	1.1938750	0.2096310	1.3516660
N	1.5022050	2.2191720	0.9477450
N	2.1878090	3.0653520	0.6155580
N	2.4602860	0.5758690	-0.5755510
O	2.8227110	1.6211730	-1.1110400
O	3.5276440	0.1295200	0.3856650
C	3.8357700	-1.2919660	0.2551000
C	4.9216860	-1.5019790	1.3095580
C	2.6116950	-2.1590980	0.5593880
C	4.3743280	-1.5719300	-1.1500610
H	5.2550310	-2.5432430	1.2960760
H	5.7829340	-0.8595790	1.1091530
H	4.5390940	-1.2732980	2.3077700
H	2.8993520	-3.2140290	0.5539200
H	1.8330820	-2.0207100	-0.1925180

Molecule ***Bconformer*** (DMF)

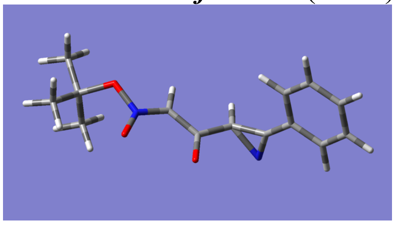

E = -878.427679, H (0K) = - -878.150390,
H (298K) = -878.130806,
G (298K) = -878.200139 au.
Imaginary frequency = 0.

C	2.4735740	-1.0485840	-0.4825630
N	2.0696470	-2.2359540	-0.4686360
C	1.0569140	-1.1213200	-0.8359460
C	0.0267390	-0.7927740	0.2000430
O	0.2308680	-0.9761660	1.3935770
C	3.6156910	-0.1970110	-0.2574040
C	3.4786930	1.1899650	-0.4034260
C	4.5763490	2.0178410	-0.1864760
C	5.8055280	1.4636780	0.1740860
C	5.9428970	0.0795230	0.3201210
C	4.8525020	-0.7543490	0.1063940
H	0.7599690	-1.0681310	-1.8789300
H	2.5169920	1.6053890	-0.6825290
H	4.4744730	3.0911740	-0.2974070
H	6.6597230	2.1099220	0.3429730
H	6.9003110	-0.3441790	0.6008590
H	4.9468050	-1.8286250	0.2177300
C	-1.2015640	-0.2368010	-0.3669580
H	-1.3248980	-0.1181360	-1.4311120
N	-2.2122780	0.1814350	0.3712240
O	-2.3630520	0.1351690	1.5867430
O	-3.2087980	0.8200260	-0.4098550
C	-4.6136080	0.3332470	-0.2021080
C	-5.2966350	0.8980690	-1.4442550
C	-4.6320320	-1.1907800	-0.1988560
C	-5.2061980	0.9346910	1.0696280
H	-6.3578650	0.6391440	-1.4179380
H	-5.2089690	1.9870100	-1.4730480
H	-4.8572400	0.4827710	-2.3538700
H	-5.6702780	-1.5297370	-0.1685670
H	-4.1221320	-1.6007080	0.6756330
H	-4.1666850	-1.5884550	-1.1039730

H	2.2012440	-1.9234480	1.5451350
H	5.2320030	-0.9289050	-1.3648670
H	4.6927850	-2.6150640	-1.2301360
H	3.6061030	-1.3917020	-1.9063070

H	-5.0649130	2.0183370	1.0786250
H	-6.2799520	0.7277480	1.0909020
H	-4.7521700	0.5134940	1.9651400

TS *Bconformer-t*-BuOH → 2a+ *t*-BuOH (DMF)

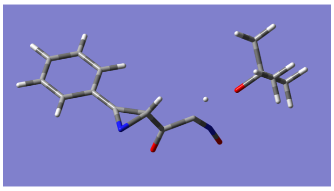

E = -878.354101, H (0K) = -878.084421,
H (298K) = -878.063790,
G (298K) = -878.137937 au.
Imaginary frequency = 1.

C	-2.3674160	-0.6133800	1.0035050
N	-2.1143930	-1.5520740	1.7931050
C	-0.9198250	-0.8190740	1.1412880
C	-0.1979050	-1.5588140	0.0824680
O	-0.6408710	-2.4788830	-0.5637780
N	1.9602400	-1.4890840	-1.0096360
O	2.4099400	-2.1249190	-1.8901040
O	3.2078670	0.2375900	0.0254720
C	4.3313870	1.0259030	0.2662770
C	4.8442170	0.7958370	1.7008650
C	5.4152940	0.5905920	-0.7475170
C	3.9922840	2.5136470	0.0522520
C	-3.3985530	0.1618910	0.3659590
C	-3.0392970	1.1698760	-0.5393190
C	-4.0323550	1.9214460	-1.1594700
C	-5.3754840	1.6680990	-0.8763780
C	-5.7333920	0.6624250	0.0273580
C	-4.7501960	-0.0942190	0.6513500
H	-0.3295560	-0.1624750	1.7712710
H	5.7395290	1.3907490	1.9097220
H	4.0697010	1.0747060	2.4223890
H	5.0851370	-0.2610490	1.8482190
H	6.3280120	1.1785130	-0.6079060
H	5.0559880	0.7369770	-1.7697580
H	5.6550980	-0.4674980	-0.6117220
H	3.2024890	2.8189940	0.7459220
H	4.8645190	3.1541350	0.2199820
H	3.6327420	2.6736200	-0.9684900
H	-1.9913800	1.3526710	-0.7482440
H	-3.7614080	2.7018790	-1.8607810
H	-6.1480240	2.2549600	-1.3606240
H	-6.7786530	0.4719860	0.2414690
H	-5.0141200	-0.8771290	1.3529270
C	1.2114260	-1.0320370	-0.1438500
H	1.7724090	-0.1763730	0.4099010

Molecule **2a** (DMF)

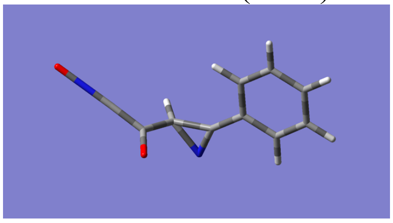

E = -644.671071, H (0K) = -644.532521,
H (298K) = -644.519472,
G (298K) = -644.573988 au.
Imaginary frequency = 0.

C	0.4382060	-0.9691660	-0.4769950
N	-0.0753980	-2.1047960	-0.5992630
C	-1.0067330	-0.8735940	-0.7184930
C	1.6551910	-0.2376220	-0.2376730
C	-1.9087670	-0.6304310	0.4307140
O	-1.7115970	-1.0193370	1.5666830
C	-3.1027270	0.1460640	0.1123450
N	-4.0603590	0.7712330	-0.0828620
O	-5.0563900	1.4201860	-0.2906210
C	1.6185120	1.1625460	-0.1864820
C	2.7926940	1.8722370	0.0451890
C	3.9956330	1.1876690	0.2256680
C	4.0316240	-0.2095910	0.1754440
C	2.8653530	-0.9274140	-0.0555080
H	-1.3843630	-0.6278260	-1.7057440
H	0.6757170	1.6787410	-0.3275850
H	2.7709090	2.9549890	0.0861230
H	4.9094100	1.7426000	0.4069120
H	4.9694890	-0.7339720	0.3175170
H	2.8791160	-2.0105800	-0.0960870

Molecule **7k** (DMF)

Molecule ***B*** (DMF)

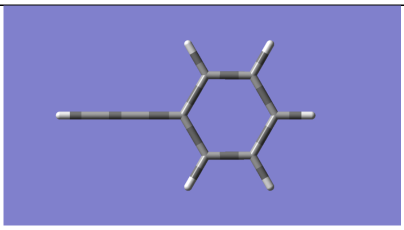

E = -308.492902, H (0K) = -308.383861,
H (298K) = -308.376495,
G (298K) = -308.414287 au.
Imaginary frequency = 0.

C	-3.2276340	0.0003340	0.0001110
C	-2.0211220	-0.0002310	-0.0001760
H	-4.2943780	-0.0009220	0.0005080
C	-0.5919500	-0.0001090	-0.0000370
C	0.1184840	-1.2136560	-0.0000440
C	0.1182870	1.2135500	-0.0000460
C	1.5101820	-1.2082170	0.0000230
H	-0.4256220	-2.1509410	-0.0001010
C	1.5099880	1.2083340	0.0000240
H	-0.4259700	2.1507480	-0.0001030
C	2.2092910	0.0001150	0.0000700
H	2.0497690	-2.1486930	0.0000210
H	2.0494190	2.1488980	0.0000220
H	3.2936260	0.0001980	0.0001050

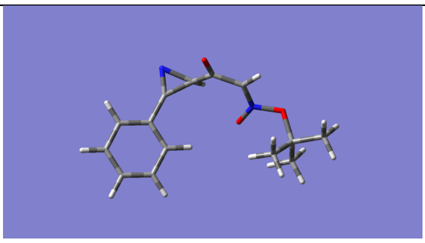

E = -878.428572, H (0K) = -878.151021,
H (298K) = -878.131611,
G (298K) = -878.200108 au.
Imaginary frequency = 0.

C	-2.2010510	1.2152040	-0.4827570
N	-2.3267610	2.4503830	-0.6738420
C	-0.9078560	1.8392860	-0.7307610
C	-0.0057630	2.1551700	0.4143070
O	-0.3806120	2.8196070	1.3745220
N	1.8228630	0.7055310	-0.3664170
O	1.1960610	0.0028160	-1.1678710
O	3.2097850	0.5029420	-0.2440780
C	3.6357990	-0.9029610	0.0858110
C	5.0673950	-0.6627110	0.5536600
C	2.7621270	-1.4488730	1.2081940
C	3.6048560	-1.7752530	-1.1656460
C	-2.8794310	-0.0216650	-0.1891930
C	-2.1224520	-1.1976820	-0.0862890
C	-2.7582870	-2.4024350	0.1983700
C	-4.1420280	-2.4330370	0.3796050
C	-4.8969230	-1.2600330	0.2765410
C	-4.2714980	-0.0523900	-0.0075040
H	-0.4492870	1.7280340	-1.7073650
H	5.5254230	-1.6212760	0.8090090
H	5.6607830	-0.1981440	-0.2377600
H	5.0867260	-0.0192830	1.4359950
H	3.1559430	-2.4198120	1.5179500
H	1.7299620	-1.5967080	0.8832320
H	2.7737190	-0.7815800	2.0731910
H	4.1424130	-1.2871350	-1.9822790
H	4.1015920	-2.7244470	-0.9453900
H	2.5854990	-1.9807060	-1.4884610
H	-1.0489640	-1.1532090	-0.2355160
H	-2.1784180	-3.3146160	0.2783830
H	-4.6362640	-3.3723600	0.6017080
H	-5.9709430	-1.2919010	0.4194250
H	-4.8455100	0.8636660	-0.0885200
C	1.3824620	1.6779910	0.4027250
H	2.0886540	2.0914740	1.1052520

TS *Bconformer* → *B* (DMF)

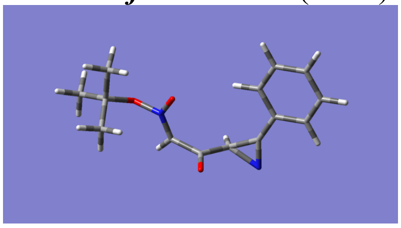

E = -878.418485, H (0K) = -878.141945,
H (298K) = -878.123090,
G (298K) = -878.190049 au.

Molecule **TBN'** (DCM)

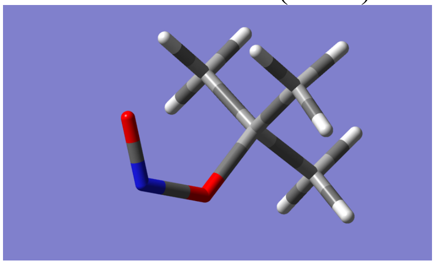

E = -363.084455, H (0K) = -362.952742,
H (298K) = -362.943592,

Imaginary frequency = 1.

C	2.3970710	-1.3389430	-0.3756280
N	2.3834820	-2.5882490	-0.2906260
C	1.0788390	-1.8737930	-0.7328980
C	-0.0149820	-1.7576320	0.2594370
O	0.1264960	-1.9402610	1.4529580
C	3.1677650	-0.1347550	-0.2006480
C	2.5227000	1.1054130	-0.3069620
C	3.2537100	2.2761700	-0.1301620
C	4.6203310	2.2089210	0.1460500
C	5.2628440	0.9711590	0.2522750
C	4.5415920	-0.2038730	0.0827750
H	0.7979900	-1.9556360	-1.7783320
H	1.4576410	1.1393000	-0.5101790
H	2.7602950	3.2380990	-0.2066920
H	5.1884040	3.1226940	0.2807290
H	6.3241830	0.9272760	0.4678530
H	5.0270700	-1.1697700	0.1634120
C	-1.3479770	-1.3676570	-0.3180380
H	-1.9760980	-2.0703150	-0.8444850
N	-1.7419160	-0.1347980	-0.2163520
O	-1.1491340	0.8230530	0.3204140
O	-2.9686600	0.1245910	-0.8815000
C	-4.0691810	0.6192760	0.0044590
C	-5.2895530	0.3760320	-0.8782860
C	-4.1244530	-0.2163160	1.2778230
C	-3.8873910	2.1077840	0.2917360
H	-6.1847850	0.7312400	-0.3620260
H	-5.1987100	0.9199210	-1.8219440
H	-5.4085650	-0.6883370	-1.0934510
H	-4.9983150	0.0853260	1.8601840
H	-3.2392970	-0.0629130	1.8997440
H	-4.2161500	-1.2799780	1.0452810
H	-3.7559470	2.6608380	-0.6416800
H	-4.7826120	2.4860170	0.7938040
H	-3.0235830	2.2877880	0.9300500

G (298K) = -362.984466 au.

Imaginary frequency = 0.

N	-1.7551850	-0.7273000	0.0000110
O	-2.0672400	0.4207360	0.0000090
O	-0.4185970	-1.0077340	-0.0000450
C	0.6554420	0.0585440	0.0000000
C	1.9138240	-0.8037060	-0.0001710
C	0.5599700	0.8910520	-1.2768360
C	0.5601500	0.8907850	1.2770290
H	2.7937840	-0.1554220	-0.0001510
H	1.9500060	-1.4383550	0.8882200
H	1.9498910	-1.4381590	-0.8887060
H	1.4605030	1.5067220	-1.3542410
H	-0.3054780	1.5520780	-1.2791690
H	0.5161310	0.2411970	-2.1543480
H	0.5164670	0.2407300	2.1544030
H	1.4606780	1.5064590	1.3544280
H	-0.3053130	1.5517880	1.2796470

TS$^{\mathbf{TBN\text{-}TBN'}}$ (DCM)

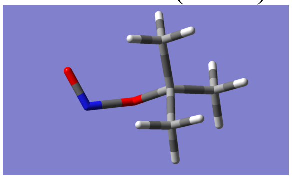

E = -363.067648, H (0K) = -362.937264,
H (298K) = -362.928453,
G (298K) = -362.968978 au.

Imaginary frequency = 1.

N	-1.7102020	-0.6261660	-0.1383090
O	-2.3147270	0.3685810	-0.0115040
O	-0.4439170	-0.4303800	-0.8130950
C	0.7005190	0.0322410	0.0303130
C	1.8569670	0.0175320	-0.9620480
C	0.4416110	1.4407090	0.5607110
C	0.9159200	-0.9731410	1.1609620
H	2.7741130	0.3282030	-0.4544150
H	2.0054520	-0.9862790	-1.3662880
H	1.6666180	0.7072490	-1.7876540

TS$^{\mathbf{TBN'\text{ - }ClNO+\mathit{t}\text{-}BuOH}}$ (DCM)

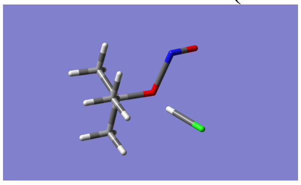

E = -823.924542, H (0K) = -823.782709,
H (298K) = -823.771287,
G (298K) = -823.819348 au.

Imaginary frequency = 1.

N	0.6239450	1.7569110	0.3725750
O	1.3523420	2.2440190	-0.2953600
O	-0.0835470	0.2912520	-0.5583450
C	-1.3363870	-0.2611650	0.0094720
C	-1.1120310	-0.6575680	1.4669500
C	-2.3744480	0.8441340	-0.1382520
C	-1.6640990	-1.4715530	-0.8658100
H	-2.0364320	-1.0795880	1.8683980
H	-0.3233310	-1.4077490	1.5508460

H	1.3396230	1.7943340	1.0756420
H	-0.3816410	1.4649420	1.2774560
H	0.2163890	2.1276630	-0.2577740
H	1.0480850	-1.9802390	0.7590940
H	1.8119440	-0.7019350	1.7255400
H	0.0698780	-0.9804250	1.8537180

H	-0.8408350	0.2079200	2.0756760
H	-3.3461280	0.4804850	0.2033700
H	-2.4651460	1.1510340	-1.1828660
H	-2.1103320	1.7166760	0.4667660
H	-0.8836290	-2.2318900	-0.7890470
H	-2.6069880	-1.9086580	-0.5262360
H	-1.7715370	-1.1751560	-1.9113380
H	0.7298180	-0.3439560	-0.4044050
Cl	2.3563740	-1.1007500	0.0021350

Molecule ***t*-BuOH** (DCM)

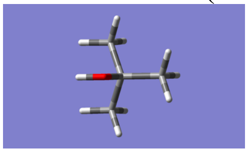

E = -233.771915, H (0K) = -233.637067,
H (298K) = -233.629497,
G (298K) = -233.665932 au.
Imaginary frequency = 0.

O	-0.0640030	-0.0006240	1.4586850
C	0.0074280	0.0000410	0.0098840
C	-0.6732060	1.2617690	-0.5304130
C	1.4989040	0.0006800	-0.3152870
C	-0.6723240	-1.2619830	-0.5310680
H	-0.6077440	1.3043840	-1.6215790
H	-1.7331440	1.2757830	-0.2551690
H	-0.1980820	2.1561870	-0.1184250
H	1.6550640	0.0018750	-1.3971520
H	1.9812670	-0.8867650	0.1034900
H	1.9805140	0.8875690	0.1055300
H	-1.7320260	-1.2777480	-0.2552850
H	-0.6068890	-1.3030570	-1.6222960
H	-0.1958940	-2.1562850	-0.1203630
H	-0.9958560	0.0000070	1.7130770

Molecule **HCl** (DCM)

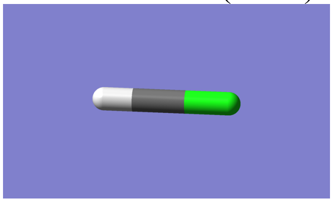

E = -460.839463, H (0K) = -460.832889,
H (298K) = -460.829584,
G (298K) = -460.850788 au.
Imaginary frequency = 0.

H	0.0000000	0.0000000	-1.2216380
Cl	0.0000000	0.0000000	0.0718610

Molecule **ClNO** (DCM)

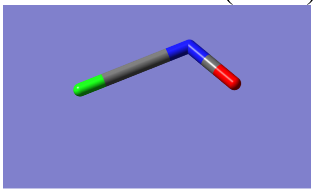

E = -590.160794, H (0K) = -590.154377,
H (298K) = -590.149967,
G (298K) = -590.179883 au.
Imaginary frequency = 0.

N	0.0000000	1.0291610	0.0000000
O	1.1005530	1.2242680	0.0000000
Cl	-0.5179070	-0.9998980	0.0000000

$\mathbf{TS^{1a+ClNO\ -\ D+N2}}$ (DCM)

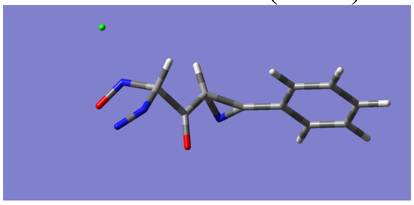

E = -1214.991554, H (0K) = -1214.832343,
H (298K) = -1214.816098,
G (298K) = -1214.878118 au.
Imaginary frequency = 1.

C	1.5909160	-0.5706130	-0.8576000
N	1.0171240	-1.4770340	-1.4999560
C	0.1384470	-0.3371870	-0.9299390
C	-0.6950620	-0.6960680	0.2281550
O	-0.4894760	-1.5877440	1.0114450
C	2.8427770	-0.0641610	-0.3617690
C	2.8589380	1.1191360	0.3893730
C	4.0683260	1.6118990	0.8683940
C	5.2539950	0.9265120	0.5987390
C	5.2372830	-0.2544990	-0.1500610
C	4.0355900	-0.7552030	-0.6332250
H	-0.2679640	0.3799390	-1.6354530

H	1.9292590	1.6393080	0.5901410
H	4.0877280	2.5261130	1.4497620
H	6.1957380	1.3120720	0.9727720
H	6.1622790	-0.7809840	-0.3543760
H	4.0083500	-1.6695550	-1.2144520
C	-1.9740690	0.2122790	0.3788680
H	-1.7585720	1.2707000	0.2827900
N	-2.5726290	-0.0873510	1.7224050
N	-3.4236410	-0.7844600	1.9833150
N	-2.9932050	-0.2291740	-0.6028770
O	-3.6992220	-1.1455880	-0.2611210
Cl	-4.1872720	1.6519190	-0.8529170

Molecule **D** (DCM)

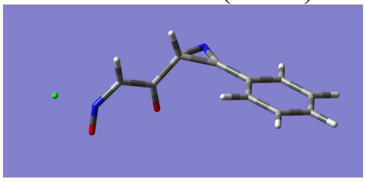

E = -1105.504474, H (0K) = -1105.352337,
H (298K) = -1105.337919,
G (298K) = -1105.395685 au.
Imaginary frequency = 0.

C	1.3498030	-1.0370250	-0.5506500
N	0.9217590	-2.2119250	-0.6272380
C	-0.0682500	-1.0607340	-0.9220800
C	-1.0903220	-0.7975020	0.1291410
O	-0.9253030	-1.0582520	1.3054180
C	2.4952460	-0.2128160	-0.2605000
C	2.3768260	1.1818500	-0.3298880
C	3.4806180	1.9812030	-0.0490590
C	4.6962120	1.3909470	0.3000800
C	4.8143470	-0.0007750	0.3703870
C	3.7184050	-0.8071340	0.0914910
H	-0.3638880	-0.9248260	-1.9573480
H	1.4255260	1.6259700	-0.5996900
H	3.3939900	3.0602040	-0.1003690
H	5.5549870	2.0152540	0.5201390
H	5.7609670	-0.4520550	0.6441470
H	3.7959030	-1.8869550	0.1448190
C	-2.3327630	-0.1725040	-0.4033840
H	-2.4690560	0.0376010	-1.4507720
N	-3.2901090	0.1366770	0.4180250
O	-3.4293970	0.0288370	1.5989840
Cl	-4.8425350	0.9702240	-0.5426400

TS$^{\mathbf{D+N2\ -\ 2a+N2+HCl}}$ (DCM)

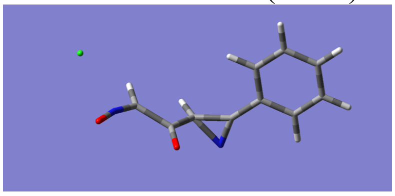

E = -1105.471110, H (0K) = -1105.322758,
H (298K) = -1105.307976,
G (298K) = -1105.366757 au.
Imaginary frequency = 1.

C	1.3023530	-0.7749790	-0.8719910
N	0.8918020	-1.7975670	-1.4668750
C	-0.1565210	-0.7429340	-1.0707190
C	-1.0189890	-1.1080250	0.0710780
O	-0.7953510	-1.8879560	0.9480860
C	2.4325770	-0.0702820	-0.3348170
C	2.2383590	1.1675010	0.2946510
C	3.3320690	1.8487310	0.8174820
C	4.6100930	1.2970730	0.7122600
C	4.8028310	0.0621910	0.0845470
C	3.7186770	-0.6272930	-0.4409500
H	-0.6055240	-0.1457930	-1.8563900
H	1.2391840	1.5807650	0.3693380
H	3.1901670	2.8052990	1.3062090
H	5.4612000	1.8297610	1.1213420
H	5.7985560	-0.3585200	0.0081540
H	3.8522890	-1.5856500	-0.9289990
C	-2.3689890	-0.2941710	0.0255950
H	-2.6231850	0.4711500	-0.7333440
N	-3.2052180	-0.5072930	0.9021170
O	-3.8064760	-0.8885700	1.8120260
Cl	-4.5093480	1.7177850	-0.7727480

TS$^{\mathbf{D+N2\ -\ D'+N2}}$ (DCM)

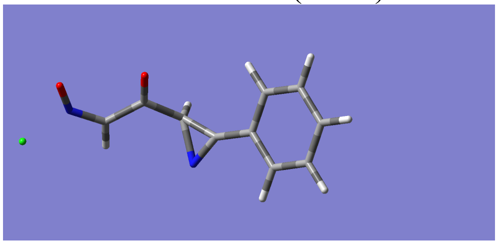

E = -1105.495966, H (0K) = -1105.344197,
H (298K) = -1105.330526,
G (298K) = -1105.385678 au.
Imaginary frequency = 1.

Molecule ***D'*** (DCM)

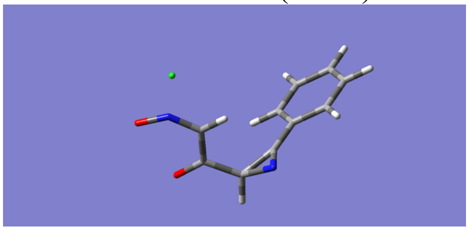

E = -1105.504611, H (0K) = -1105.352407,
H (298K) = -1105.338011,
G (298K) = -1105.395759 au.
Imaginary frequency = 0.

C	-1.1853070	-0.7208140	0.5403100
N	-0.3581810	-1.6765840	0.4848600
C	0.0677580	-0.3807850	1.1827070
C	1.0908190	0.4773700	0.4700220
O	0.8595840	1.5977240	0.0672350
C	-2.5227930	-0.3412670	0.1614390
C	-3.0012510	0.9346580	0.4913330
C	-4.2947590	1.3004070	0.1310220
C	-5.1075930	0.3979270	-0.5565500
C	-4.6310600	-0.8746540	-0.8879560
C	-3.3415560	-1.2481550	-0.5324490
H	0.1922640	-0.4206650	2.2638140
H	-2.3571590	1.6268270	1.0211130
H	-4.6680000	2.2861210	0.3832820
H	-6.1149930	0.6849090	-0.8368680
H	-5.2675870	-1.5701080	-1.4227520
H	-2.9605450	-2.2315350	-0.7835770
C	2.4064650	-0.1964440	0.3228730
H	2.5756670	-1.1998750	0.6745300
N	3.3856830	0.4355980	-0.2550980
O	3.4869230	1.5228370	-0.7378560
Cl	5.0442020	-0.6789830	-0.3223180

C	1.0318900	-0.9954640	0.8577800
N	0.3860550	-1.2240470	1.9099790
C	-0.0667670	-1.9319220	0.6074280
C	-1.3272270	-1.5141090	-0.0652220
O	-1.9100990	-2.2587850	-0.8313310
C	2.1359210	-0.2663450	0.2901390
C	2.3890760	-0.3617680	-1.0856880
C	3.4508010	0.3461890	-1.6400060
C	4.2559610	1.1436300	-0.8252160
C	4.0043880	1.2381610	0.5474800
C	2.9466500	0.5363660	1.1103260
H	0.1329150	-2.9960520	0.5262880
H	1.7550170	-0.9859640	-1.7048910
H	3.6508760	0.2771200	-2.7027960
H	5.0832350	1.6940200	-1.2591430
H	4.6346430	1.8592680	1.1732470
H	2.7411200	0.6008050	2.1727000
C	-1.8133060	-0.1431570	0.2614930
H	-1.3180350	0.4991070	0.9684530
N	-2.8877550	0.2963790	-0.3184380
O	-3.6425120	-0.1654820	-1.1203100
Cl	-3.3406650	2.1547070	0.2910230

TS$^{D'+N2 - 2a'+N2+HCl}$ (DCM)

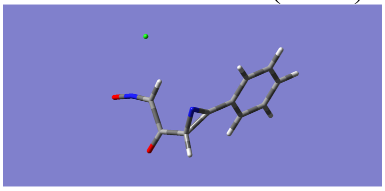

E = -1105.472324, H (0K) = -1105.324311,
H (298K) = -1105.309502,
G (298K) = -1105.368317 au.
Imaginary frequency = 1.

C	0.7434600	-0.7961920	1.0909600
N	-0.0104240	-0.6487290	2.0852370
C	-0.3198570	-1.8079050	1.1288950
C	-1.4945170	-1.7178640	0.2332540
O	-2.0543050	-2.6300460	-0.2960390
C	1.8831170	-0.3016030	0.3754390
C	2.2890250	-0.9443220	-0.8040020
C	3.3886710	-0.4592770	-1.5025370
C	4.0756560	0.6595290	-1.0273190
C	3.6693220	1.3004200	0.1478470
C	2.5741300	0.8251120	0.8550960
H	-0.1267170	-2.8224450	1.4647180
H	1.7437410	-1.8116030	-1.1581070
H	3.7099390	-0.9491090	-2.4140260
H	4.9319760	1.0367050	-1.5750120
H	4.2070240	2.1705020	0.5055370
H	2.2430240	1.3131210	1.7642660
C	-1.9808080	-0.2301890	0.0272390
H	-1.5735860	0.6495390	0.5649380
N	-2.8759760	-0.0208140	-0.7866540
O	-3.7109670	-0.0845740	-1.5833400
Cl	-2.2222130	2.8029880	0.2143180

Molecule **2a’** (DCM)

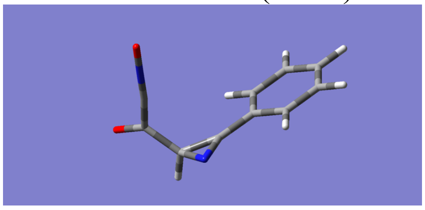

E = -644.673247, H (0K) = -644.534733,
H (298K) = -644.521651,
G (298K) = -644.576250 au.
Imaginary frequency = 0.

C	0.0558190	0.7862040	-0.8369110
N	-0.7212860	0.7761760	-1.8210660
C	-1.1606250	1.5569180	-0.5523260
C	-2.2522790	0.9870670	0.2624170
O	-2.9813430	1.6525210	0.9732890
C	1.3252030	0.3339400	-0.3301170
C	1.6934850	0.6506950	0.9849420
C	2.9165240	0.2103700	1.4808660
C	3.7670100	-0.5407330	0.6680240
C	3.4001890	-0.8555380	-0.6444940
C	2.1815300	-0.4213610	-1.1489430
H	-1.1528200	2.6409310	-0.6019960
H	1.0219540	1.2350930	1.6035410
H	3.2062600	0.4507960	2.4970040
H	4.7196120	-0.8832130	1.0564290
H	4.0661980	-1.4393260	-1.2691430
H	1.8843880	-0.6585680	-2.1639820
C	-2.4298680	-0.4641030	0.1782350
N	-2.6650110	-1.5999890	0.2169450
O	-2.8965880	-2.7824930	0.2438160

TS$^{2a' - 2a}$ (DCM)

TS$^{D'+N2 - D''+N2}$ (DCM)

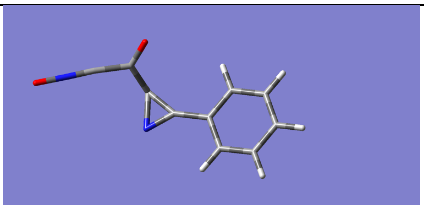

E = -644.662603, H (0K) = -644.524318,
H (298K) = -644.512024,
G (298K) = -644.564092 au.
Imaginary frequency = 1.

C	-0.2554100	-0.4647760	0.5203190
N	0.6523040	-1.3441410	0.5686480
C	1.0057770	0.0904330	0.9714560
C	1.8828220	0.8715450	0.0257950
O	1.5494510	1.8977680	-0.5269890
C	-1.6471550	-0.2681490	0.2028790
C	-2.2132630	1.0082530	0.3294080
C	-3.5583020	1.1986930	0.0272740
C	-4.3353520	0.1208740	-0.3997730
C	-3.7715810	-1.1528030	-0.5274060
C	-2.4300700	-1.3521750	-0.2284710
H	1.2145340	0.2743180	2.0243220
H	-1.5966250	1.8367160	0.6582300
H	-3.9994980	2.1839810	0.1226790
H	-5.3830590	0.2714300	-0.6352230
H	-4.3809480	-1.9851640	-0.8600770
H	-1.9813010	-2.3344300	-0.3233280
C	3.2042320	0.2976900	-0.1793020
N	4.2230990	-0.2502130	-0.2820250
O	5.2891610	-0.7957550	-0.4037650

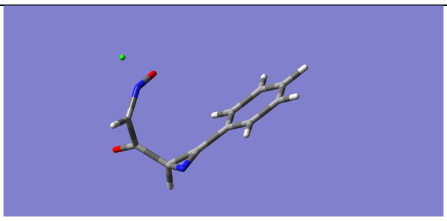

E = -1105.496179, H (0K) = -1105.344961,
H (298K) = -1105.331182,
G (298K) = -1105.387236 au.
Imaginary frequency = 1.

C	-0.5678790	-1.3156910	-0.7195590
N	0.0968660	-1.7213700	-1.7009950
C	0.5257230	-2.1963080	-0.2802620
C	1.7750600	-1.6433120	0.2709910
O	2.4618020	-2.2168310	1.0874860
C	-1.6902470	-0.5161730	-0.3042360
C	-1.9922060	-0.4077660	1.0600940
C	-3.0706840	0.3719120	1.4643760
C	-3.8413240	1.0409260	0.5120800
C	-3.5394980	0.9332720	-0.8493390
C	-2.4657820	0.1564970	-1.2635220
H	0.3258050	-3.2306490	-0.0184680
H	-1.3798360	-0.9275430	1.7874330
H	-3.3081190	0.4616870	2.5178590
H	-4.6800840	1.6502660	0.8297160
H	-4.1420150	1.4570820	-1.5822650
H	-2.2190140	0.0656600	-2.3150600
C	2.2392020	-0.2944280	-0.2723980
H	2.8888780	-0.2328240	-1.1313910
N	1.8838840	0.7739810	0.3492780
O	1.1803740	0.9695950	1.3200350
Cl	2.6633850	2.3877850	-0.4467830

Molecule ***D''*** (DCM)

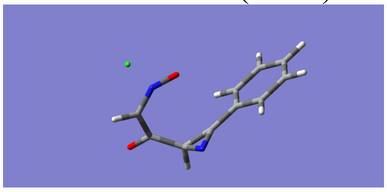

E = -1105.498866, H (0K) = -1105.346951,
H (298K) = -1105.332594,
G (298K) = -1105.389477 au.
Imaginary frequency = 0.

C	0.5533170	-1.0657110	0.7504360
N	-0.1822640	-1.2955060	1.7389060
C	-0.4979360	-2.0247290	0.3956630
C	-1.7852950	-1.7059510	-0.2555300
O	-2.4142210	-2.5487610	-0.8702140
C	1.7485480	-0.3929860	0.3060530
C	2.1571890	-0.5212070	-1.0273240
C	3.3085570	0.1301030	-1.4585640
C	4.0463110	0.9055900	-0.5626490
C	3.6376920	1.0323120	0.7687260
C	2.4895830	0.3857890	1.2082780
H	-0.2545020	-3.0821330	0.3449360
H	1.5702110	-1.1224930	-1.7120420

TS$^{\mathbf{D''+N2\ -\ 2a'+N2+HCl}}$ (DCM)

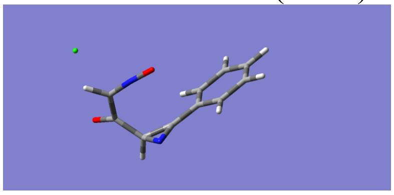

E = -1105.470550, H (0K) = -1105.322257,
H (298K) = -1105.307506,
G (298K) = -1105.365779 au.
Imaginary frequency = 1.

C	-0.8286350	1.0990720	0.8156410
N	-0.0685160	1.2881590	1.7991120
C	0.1701630	2.1411330	0.5462480
C	1.3665780	1.8577030	-0.2706040
O	1.9036560	2.6105200	-1.0242170
C	-1.9065850	0.3118220	0.2936170
C	-2.3500170	0.5285260	-1.0200940
C	-3.3763090	-0.2567450	-1.5312130
C	-3.9521460	-1.2517440	-0.7378050
C	-3.5073570	-1.4694110	0.5701320
C	-2.4842350	-0.6914360	1.0932090
H	-0.0853940	3.1952590	0.5803550
H	-1.8891910	1.3040890	-1.6209570

H	3.6291540	0.0363370	-2.4895310
H	4.9422180	1.4141860	-0.9009100
H	4.2150670	1.6367970	1.4586330
H	2.1593350	0.4784480	2.2365320
C	-2.4699850	-0.3793070	-0.0671570
H	-3.5376860	-0.3783830	0.0836810
N	-1.8643650	0.7582080	-0.1975370
O	-0.7228270	1.0644120	-0.4559130
Cl	-3.0840520	2.2629230	0.0265020

H	-3.7267740	-0.0979720	-2.5439850
H	-4.7516690	-1.8633330	-1.1404230
H	-3.9589770	-2.2463750	1.1753820
H	-2.1254730	-0.8480100	2.1038620
C	2.0480670	0.4376830	-0.1143440
H	3.1244890	0.3362950	-0.3277460
N	1.4517600	-0.6073970	0.1590510
O	0.7480240	-1.5012880	0.3721440
Cl	4.2023350	-1.7446330	-0.2697510

TS$^{D''+TBN' - 2a+ClNO+t\text{-}BuOH}$ (DCM)

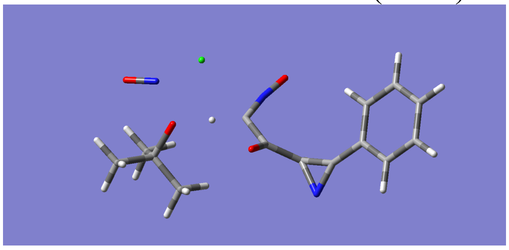

E = -1468.557412, H (0K) = -1468.277827,
H (298K) = -1468.253143,
G (298K) = -1468.334315 au.
Imaginary frequency = 1.

C	2.9357300	-1.0296980	-0.6792480
N	2.3927730	-2.0733860	-1.1076920
C	1.5360230	-0.7938540	-1.0680730
C	0.5154050	-0.7298650	-0.0044440
O	0.4299710	-1.4163970	0.9704820
C	4.1224330	-0.4176210	-0.1479610
C	4.1010510	0.9446680	0.1841290
C	5.2463300	1.5429500	0.6983580
C	6.4043340	0.7850420	0.8805900
C	6.4244690	-0.5739830	0.5507840
C	5.2871510	-1.1818810	0.0362450
H	1.2969010	-0.3148210	-2.0113930
H	3.1931030	1.5191670	0.0369600
H	5.2373650	2.5951320	0.9568720
H	7.2959440	1.2533730	1.2820710
H	7.3276780	-1.1546140	0.6972740
H	5.2877490	-2.2343150	-0.2223860
C	-0.5814530	0.3770280	-0.2063430
H	-1.6811180	0.0663970	0.1608990
N	-0.2794140	1.4776220	-0.6358670
O	0.2187450	2.4512150	-1.0366050
Cl	-2.8782580	2.7526130	-0.4877110
N	-3.5775070	0.8641470	1.4938110
O	-4.7114770	0.8482240	1.5230950
O	-2.9048190	-0.4415530	0.7170990
C	-3.7121270	-1.3892620	-0.0709270
C	-4.4924120	-0.6507620	-1.1600430
C	-2.6808430	-2.3373120	-0.6917960
C	-4.6249710	-2.1588120	0.8891100
H	-4.9796570	-1.3853120	-1.8069630
H	-5.2664180	-0.0013300	-0.7500170
H	-3.8191960	-0.0414790	-1.7654260
H	-3.2034930	-3.1276350	-1.2369820
H	-2.0636180	-2.7988080	0.0817410
H	-2.0342310	-1.8115240	-1.3988870

H	-5.3907000	-1.5211530	1.3317890
H	-5.1314050	-2.9564620	0.3388600
H	-4.0355470	-2.6114580	1.6900690

Molecule **3a** (MeCN)

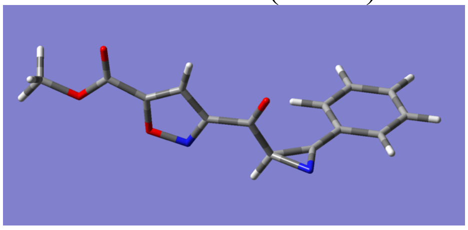

E = -950.104347, H (0K) = -949.887607,
H (298K) = -949.869201,
G (298K) = -949.937109 au.
Imaginary frequency = 0.

C	2.6970260	-1.0445730	-0.4398270
N	2.4533460	-2.2714570	-0.5015460
C	1.2811520	-1.2820300	-0.7385910
C	3.7121900	-0.0500930	-0.2023230
C	0.2926110	-1.1706760	0.3613630
O	0.5517750	-1.4334250	1.5231860
C	-1.0815180	-0.6977360	0.0095730
N	-1.4355870	-0.4513980	-1.2356470
O	-2.7485010	-0.0365840	-1.1859980
C	-2.1502200	-0.4505760	0.9141340
C	-3.1607650	-0.0400300	0.1055740
C	3.3619090	1.3069240	-0.2215550
C	4.3369490	2.2723980	0.0108980
C	5.6544970	1.8848800	0.2607760
C	6.0040180	0.5306510	0.2799320
C	5.0377820	-0.4405450	0.0503410
O	-5.2467770	0.6905250	-0.6469490
H	0.9209700	-1.1883050	-1.7561920
H	-2.1535340	-0.5622730	1.9846870
H	2.3339970	1.5910880	-0.4162100
H	4.0716780	3.3230790	-0.0019830
H	6.4129990	2.6384860	0.4408600
H	7.0291420	0.2374160	0.4746310
H	5.2956210	-1.4933360	0.0634770
C	-6.6184590	1.1147510	-0.4263030
H	-6.6363310	2.0144110	0.1899340
H	-7.0151620	1.3208610	-1.4174470
H	-7.1835860	0.3163760	0.0558840
C	-4.5443620	0.3739280	0.4364040
O	-4.9486170	0.4068020	1.5773040

Molecule **4a** (MeCN)

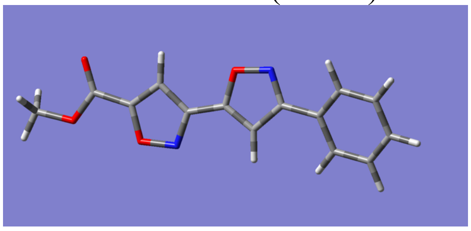

E = -950.115506, H (0K) = -949.896671,
H (298K) = -949.879122,
G (298K) = -949.944396 au.
Imaginary frequency = 0.

C	-2.3974200	-0.3240960	-0.0477790
N	-1.9403050	-1.5546970	-0.1864390
C	-1.3226430	0.6112250	0.0483930
C	-3.8438160	-0.0487420	-0.0045890
C	-0.2061160	-0.1579800	-0.0382360
O	-0.5494000	-1.4575550	-0.1780870
C	1.2151530	0.1354440	-0.0037320
N	1.6411930	1.3808600	0.0916390
O	3.0219190	1.3090400	0.1003770
C	2.2983480	-0.7855830	-0.0611370
C	3.3948640	0.0134890	0.0082700
C	-4.3161410	1.2559720	-0.2042770
C	-5.6835160	1.5221780	-0.1710460
C	-6.5934800	0.4914130	0.0630400
C	-6.1291900	-0.8100990	0.2659760
C	-4.7647100	-1.0808890	0.2341390
O	5.6172920	0.7284160	0.0730150
H	-1.3754290	1.6790090	0.1760530
H	2.2608420	-1.8585290	-0.1408690
H	-3.6196850	2.0638590	-0.3954460
H	-6.0364170	2.5349690	-0.3298930
H	-7.6572110	0.7000260	0.0900420
H	-6.8313980	-1.6145590	0.4541020
H	-4.4098440	-2.0905420	0.4025500
C	7.0488100	0.4817100	0.0668280
H	7.5069520	1.4654800	0.1322340
H	7.3261020	-0.1294760	0.9265230
H	7.3382220	-0.0168020	-0.8590160
C	4.8333260	-0.3422220	-0.0055880
O	5.2090430	-1.4908370	-0.0820850

Molecule **3c** (MeCN)

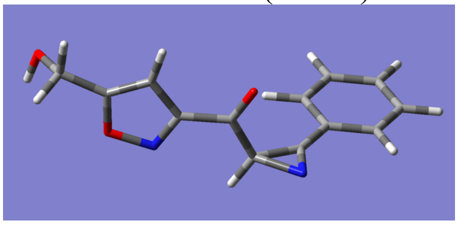

E = -836.731572, H (0K) = -836.524368,
H (298K) = -836.507767,
G (298K) = -836.570867 au.
Imaginary frequency = 0.

Molecule **4c** (MeCN)

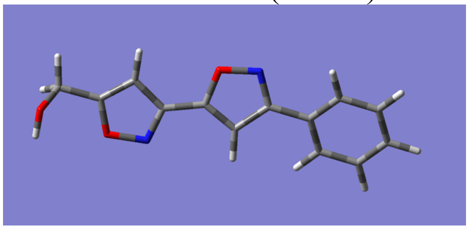

E = -836.742750, H (0K) = -836.533346,
H (298K) = -836.517690,
G (298K) = -836.577800 au.
Imaginary frequency = 0.

C	1.9572120	-1.0780250	-0.4141290
N	1.6767270	-2.2979470	-0.4612450
C	0.5337100	-1.2752220	-0.7014710
C	3.0064910	-0.1149850	-0.1937820
C	-0.4472050	-1.1249280	0.4029690
O	-0.1844070	-1.3876390	1.5649080
C	-1.8077400	-0.6182660	0.0544540
N	-2.1519130	-0.3682150	-1.1919710
O	-3.4627720	0.0750770	-1.1333260
C	-2.8702290	-0.3405170	0.9600130
C	-3.8781330	0.0905700	0.1594380
C	-5.2719940	0.5642190	0.4097490
C	2.7031600	1.2528100	-0.2339150
C	3.7116360	2.1880460	-0.0208750
C	5.0161600	1.7597930	0.2307190
C	5.3188890	0.3948100	0.2713220
C	4.3189740	-0.5462310	0.0607980
O	-5.4342360	1.9562860	0.1201380
H	0.1706140	-1.1816570	-1.7179890
H	-2.8707150	-0.4448750	2.0316040
H	-5.4933610	0.4303970	1.4683560
H	-5.9759380	-0.0433670	-0.1699820
H	1.6847650	1.5688300	-0.4294680
H	3.4822920	3.2468550	-0.0502280
H	5.8008040	2.4897390	0.3957210
H	6.3341290	0.0697730	0.4674270
H	4.5410240	-1.6068540	0.0902600
H	-5.4415730	2.0720490	-0.8386960

C	-1.6782710	-0.3398410	-0.0807140
N	-1.2300320	-1.5705310	-0.2457190
C	-0.5986720	0.5916620	-0.0138190
C	-3.1216020	-0.0612530	0.0169050
C	0.5136610	-0.1785370	-0.1449120
O	0.1611640	-1.4759780	-0.2843100
C	1.9363830	0.1121650	-0.1554960
N	2.3607700	1.3575740	-0.0744720
O	3.7482840	1.2751050	-0.1137240
C	3.0176760	-0.8114260	-0.2424580
C	4.1224040	-0.0217990	-0.2103030
C	5.5895480	-0.2992030	-0.2425810
C	-3.5978250	1.2451020	-0.1618690
C	-4.9621450	1.5151450	-0.0755390
C	-5.8654280	0.4864400	0.1913500
C	-5.3972730	-0.8167260	0.3735980
C	-4.0356940	-1.0912010	0.2887850
O	6.2255980	-0.0085250	1.0061550
H	-0.6449670	1.6582180	0.1259270
H	2.9730900	-1.8851150	-0.3124400
H	5.7325990	-1.3624580	-0.4337720
H	6.0510710	0.2648100	-1.0608160
H	-2.9066720	2.0512430	-0.3780430
H	-5.3180080	2.5292560	-0.2186660
H	-6.9267310	0.6979730	0.2598400
H	-6.0939000	-1.6196840	0.5872470
H	-3.6774380	-2.1021110	0.4418220
H	6.2788590	0.9505810	1.1075840

Molecule **3d** (MeCN)

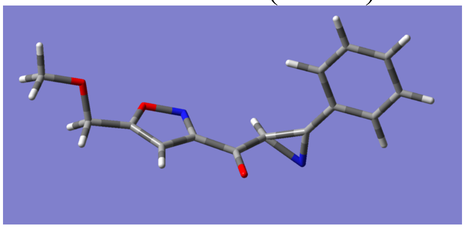

E = -876.042220, H (0K) = -875.807321,
H (298K) = -875.789303,
G (298K) = -875.856418 au.
Imaginary frequency = 0.

C	2.4619720	-1.0641470	-0.3606960
N	2.2876070	-2.3046080	-0.3565060
C	1.0667900	-1.3960520	-0.6618060
C	3.4213780	-0.0058780	-0.1698810
C	0.0535530	-1.2830600	0.4178500
O	0.3088560	-1.4911210	1.5925500
C	-1.3302260	-0.8838760	0.0264190
N	-1.6570410	-0.6687050	-1.2314680
O	-2.9951120	-0.3193520	-1.2153980
C	-2.4364290	-0.6786320	0.8975490
C	-3.4482840	-0.3269940	0.0641060
C	-4.8759890	0.0271750	0.2815490
C	3.0057090	1.3275250	-0.2864870
C	3.9274960	2.3541950	-0.1041160
C	5.2571170	2.0509380	0.1934570
C	5.6718420	0.7202280	0.3107360

Molecule **4d** (MeCN)

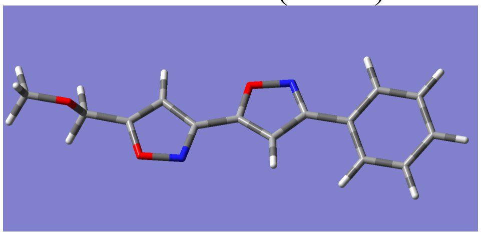

E = -876.053375, H (0K) = -875.816322,
H (298K) = -875.799225,
G (298K) = -875.863589 au.
Imaginary frequency = 0.

C	2.1387790	-0.3327840	0.1057640
N	1.6800880	-1.5501770	0.3296280
C	1.0699380	0.6106800	0.0357420
C	3.5816870	-0.0779700	-0.0465750
C	-0.0479250	-0.1384440	0.2280070
O	0.2918090	-1.4347560	0.4052530
C	-1.4659470	0.1716000	0.2658900
N	-1.8779760	1.4195350	0.1593710
O	-3.2634580	1.3576470	0.2290500
C	-2.5565750	-0.7335220	0.4051230
C	-3.6512360	0.0697680	0.3727200
C	-5.1116700	-0.1989650	0.4564660
C	4.0809630	1.2257380	0.0811770
C	5.4448930	1.4740630	-0.0593300
C	6.3245370	0.4259630	-0.3299400
C	5.8332320	-0.8748500	-0.4607650

C	4.7591230	-0.3114230	0.1306750
O	-5.0455950	1.4192170	0.0361270
H	0.7179510	-1.3774910	-1.6874040
H	-2.4632630	-0.7778150	1.9692440
H	-5.1440820	-0.2286120	1.3138840
H	-5.5095000	-0.5635710	-0.3948130
H	1.9692320	1.5458250	-0.5174090
H	3.6113200	3.3869770	-0.1931700
H	5.9741030	2.8520240	0.3346920
H	6.7059280	0.4925730	0.5423450
H	5.0679080	-1.3467330	0.2194320
C	-6.4048620	1.8245520	0.1866110
H	-6.7657270	1.6365570	1.2064340
H	-6.4412760	2.8957810	-0.0150270
H	-7.0568900	1.3004100	-0.5244250

C	4.4718570	-1.1276920	-0.3218870
O	-5.6885320	0.0084370	-0.8285980
H	1.1262700	1.6703330	-0.1464190
H	-2.5253960	-1.8049590	0.5081480
H	-5.2567050	-1.2326580	0.7933040
H	-5.5683730	0.4721340	1.1970120
H	3.4079740	2.0464150	0.3002320
H	5.8190450	2.4863640	0.0445410
H	7.3854630	0.6206520	-0.4410920
H	6.5113330	-1.6928320	-0.6770280
H	4.0948260	-2.1369520	-0.4359110
C	-7.0999640	-0.1972550	-0.8187650
H	-7.4541650	-0.0184310	-1.8347490
H	-7.3504250	-1.2250430	-0.5248050
H	-7.5985900	0.5008620	-0.1336300

Molecule **3g** (MeCN)

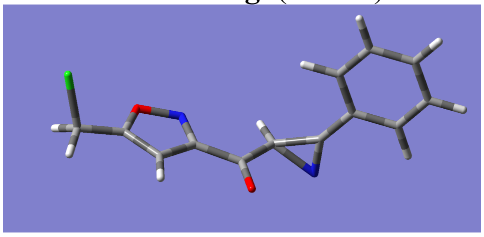

E = -1221.113947, H (0K) = -1220.920421,
H (298K) = -1220.903960,
G (298K) = -1220.967937 au.
Imaginary frequency = 0.

C	2.3688810	-1.0935510	-0.3633050
N	2.1824880	-2.3321240	-0.3549270
C	0.9689940	-1.4130330	-0.6579970
C	3.3382800	-0.0436440	-0.1780510
C	-0.0368430	-1.2861240	0.4258740
O	0.2229180	-1.4787440	1.6018120
C	-1.4241440	-0.8916620	0.0375750
N	-1.7663060	-0.7159960	-1.2221540
O	-3.1032080	-0.3663520	-1.2032740
C	-2.5187180	-0.6580760	0.9160890
C	-3.5391190	-0.3337270	0.0816220
C	-4.9544960	0.0155740	0.3072870
C	2.9330930	1.2933660	-0.2900720
C	3.8644550	2.3119030	-0.1109410
C	5.1932220	1.9969240	0.1782160
C	5.5975730	0.6625740	0.2902660
C	4.6751920	-0.3610590	0.1139210
H	0.6157420	-1.3947730	-1.6820870
H	-2.5343630	-0.7241070	1.9905800
H	-5.2487140	-0.2321070	1.3233560
H	-5.6105830	-0.4682510	-0.4130010
H	1.8971080	1.5207660	-0.5143290
H	3.5564110	3.3474820	-0.1958300
H	5.9176320	2.7917300	0.3170200
H	6.6310870	0.4258230	0.5151750
H	4.9757280	-1.3991090	0.1988780
Cl	-5.2276010	1.8254920	0.1063880

Molecule **4g** (MeCN)

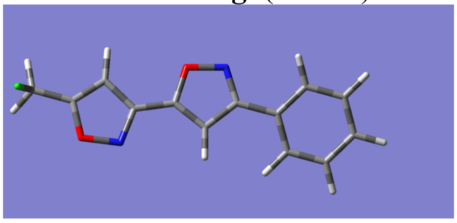

E = -1221.125083, H (0K) = -1220.929240,
H (298K) = -1220.913765,
G (298K) = -1220.974352 au.
Imaginary frequency = 0.

C	-2.0619240	-0.3255660	-0.1184400
N	-1.5974670	-1.5347970	-0.3721930
C	-0.9980300	0.6222860	-0.0317150
C	-3.5055820	-0.0816850	0.0427450
C	0.1225260	-0.1162390	-0.2460730
O	-0.2104530	-1.4101090	-0.4514620
C	1.5382000	0.2037450	-0.2893480
N	1.9494740	1.4404750	-0.0877580
O	3.3311500	1.3956770	-0.2035790
C	2.6267520	-0.6799650	-0.5376540
C	3.7171810	0.1271000	-0.4701860
C	5.1619200	-0.1244550	-0.6309910
C	-4.0136510	1.2197430	-0.0728040
C	-5.3787180	1.4577110	0.0740560
C	-6.2503580	0.4014900	0.3391240
C	-5.7500430	-0.8970080	0.4580730
C	-4.3874740	-1.1396570	0.3126050
H	-1.0599180	1.6778160	0.1715980
H	2.5988130	-1.7382760	-0.7345000
H	5.3298700	-1.0750160	-1.1289610
H	5.6557390	0.6836550	-1.1657460
H	-3.3466120	2.0463310	-0.2879340
H	-5.7600790	2.4681970	-0.0208290
H	-7.3121980	0.5881530	0.4551830
H	-6.4219770	-1.7211170	0.6702530
H	-4.0031010	-2.1471560	0.4174180
Cl	5.9979430	-0.2359920	1.0063030

Molecule **3h** (MeCN)

Molecule **4h** (MeCN)

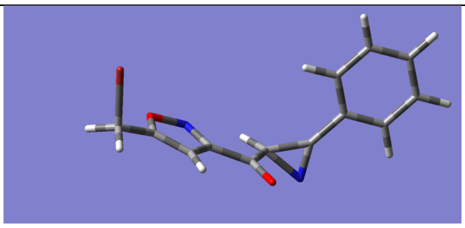

E = -3335.037256, H (0K) = -3334.844308,
H (298K) = -3334.827720,
G (298K) = -3334.892465 au.
Imaginary frequency = 0.

C	3.1027370	-1.0870130	-0.3197760
N	3.0514740	-2.3373590	-0.2645430
C	1.7510320	-1.5682890	-0.6189240
C	3.9461620	0.0711040	-0.1647530
C	0.7182970	-1.5085510	0.4453800
O	0.9723170	-1.6484960	1.6298900
C	-0.6908410	-1.2472660	0.0237860
N	-1.0164630	-1.1002680	-1.2442690
O	-2.3786840	-0.8679730	-1.2579500
C	-1.8221630	-1.1132580	0.8751010
C	-2.8476460	-0.8763590	0.0157910
C	-4.2859480	-0.6383160	0.2059970
C	3.3916100	1.3492000	-0.3182600
C	4.1990890	2.4724220	-0.1657750
C	5.5531540	2.3201510	0.1372680
C	6.1067290	1.0447010	0.2897960
C	5.3085510	-0.0824770	0.1410860
H	1.4154330	-1.6258830	-1.6475160
H	-1.8574830	-1.1818610	1.9490410
H	-4.6056640	-0.9544550	1.1945120
H	-4.8875030	-1.1091250	-0.5678890
H	2.3379930	1.4498940	-0.5528210
H	3.7753060	3.4630880	-0.2821330
H	6.1809500	3.1963560	0.2550760
H	7.1590710	0.9343680	0.5248170
H	5.7251830	-1.0764490	0.2574670
Br	-4.7092000	1.3223690	0.0907230

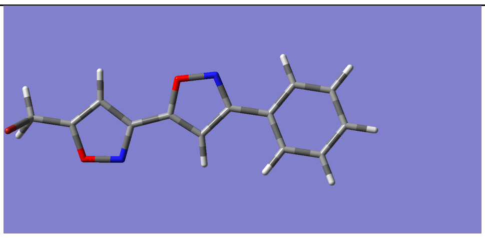

E = -3335.048376, H (0K) = -3334.853279,
H (298K) = -3334.837553,
G (298K) = -3334.900022 au.
Imaginary frequency = 0.

C	-2.7979730	0.3443900	-0.0016330
N	-2.3394300	1.5716790	-0.1634510
C	-1.7346370	-0.5640390	0.2848130
C	-4.2362740	0.0473880	-0.1151680
C	-0.6224750	0.2170750	0.2861830
O	-0.9586720	1.4988380	0.0209350
C	0.7857720	-0.0517330	0.5161450
N	1.1932280	-1.2771290	0.7843640
O	2.5667890	-1.1831400	0.9530700
C	1.8688050	0.8719090	0.4974550
C	2.9534030	0.1011820	0.7777730
C	4.3857470	0.4042270	0.9192920
C	-4.6670530	-1.2724070	-0.3096850
C	-6.0258920	-1.5597710	-0.4224180
C	-6.9688240	-0.5352390	-0.3406520
C	-6.5462970	0.7813910	-0.1441730
C	-5.1904280	1.0733330	-0.0306950
H	-1.7922800	-1.6223620	0.4740240
H	1.8421470	1.9316220	0.3074920
H	4.5458120	1.4700520	1.0519590
H	4.8471390	-0.1626050	1.7243620
H	-3.9432640	-2.0755810	-0.3830480
H	-6.3462280	-2.5842090	-0.5755760
H	-8.0260810	-0.7603020	-0.4264530
H	-7.2748880	1.5813330	-0.0735840
H	-4.8690400	2.0949510	0.1327850
Br	5.3986260	-0.1030450	-0.7392520

Molecule **3i** (MeCN)

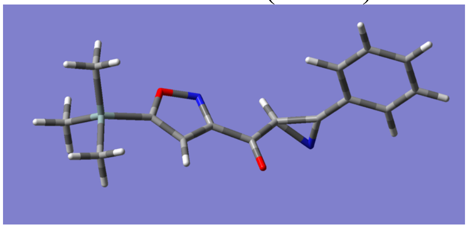

E = -1130.910921, H (0K) = -1130.636218,
H (298K) = -1130.614356,
G (298K) = -1130.689171 au.
Imaginary frequency = 0.

C	3.0428540	-1.1050380	-0.3481970
N	2.8001830	-2.3338550	-0.3225530
C	1.6398420	-1.3663780	-0.6797250
C	4.0535500	-0.0964310	-0.1518470
C	0.6062240	-1.1720800	0.3698130
O	0.8232960	-1.3644780	1.5552090
C	-0.7435580	-0.7114290	-0.0672730

Molecule **4i** (MeCN)

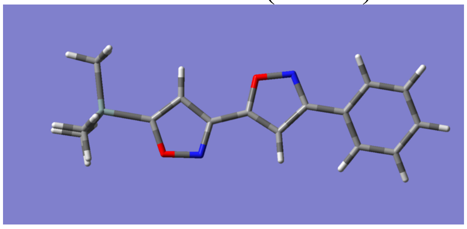

E = -1130.922069, H (0K) = -1130.645270,
H (298K) = -1130.624295,
G (298K) = -1130.696918 au.
Imaginary frequency = 0.

C	2.7379720	0.3101840	-0.0580910
N	2.2412200	1.5230830	-0.2160790
C	1.6949870	-0.6577610	0.0533190
C	4.1929950	0.0834780	-0.0094110
C	0.5514040	0.0711490	-0.0440440
O	0.8534160	1.3791280	-0.2049220
C	-0.8597690	-0.2689460	-0.0016540

N	-1.0199770	-0.4867660	-1.3344260	N	-1.2393460	-1.5252940	0.1089910
O	-2.3418200	-0.0718300	-1.3564250	O	-2.6301330	-1.4860640	0.1224050
C	-1.8674390	-0.4519570	0.7674800	C	-1.9755430	0.6152690	-0.0640100
C	-2.8510290	-0.0520650	-0.0882170	C	-3.0665380	-0.2003990	0.0181780
C	3.7128720	1.2549440	-0.3009090	C	4.7089620	-1.2077260	-0.1884580
C	4.6833820	2.2344540	-0.1118330	C	6.0842800	-1.4284090	-0.1484330
C	5.9873250	1.8665920	0.2240630	C	6.9594960	-0.3650350	0.0719140
C	6.3274230	0.5180560	0.3731510	C	6.4520280	0.9233390	0.2541540
C	5.3654860	-0.4667650	0.1871730	C	5.0793530	1.1484920	0.2156340
H	1.3189860	-1.3529130	-1.7144840	H	1.7832520	-1.7209100	0.1982810
H	-1.9210220	-0.5528330	1.8385480	H	-1.9581130	1.6886280	-0.1547410
H	2.6954770	1.5239580	-0.5613210	H	4.0400400	-2.0409880	-0.3688810
H	4.4249910	3.2808420	-0.2252690	H	6.4705330	-2.4314110	-0.2910960
H	6.7423080	2.6309920	0.3704990	H	8.0294060	-0.5383030	0.1043070
H	7.3419270	0.2398970	0.6343750	H	7.1269490	1.7531710	0.4317890
H	5.6164990	-1.5152720	0.3001710	H	4.6912780	2.1483900	0.3683070
Si	-4.6629420	0.4837000	0.1517590	C	-5.6395830	-0.5693470	1.6131270
C	-5.7336870	-0.6788350	-0.8602530	H	-6.7214220	-0.4035960	1.6579210
H	-5.6078170	-1.7162030	-0.5347910	H	-5.4601040	-1.6466730	1.6861750
H	-6.7905990	-0.4135130	-0.7488990	H	-5.1855310	-0.0871450	2.4844440
H	-5.4819610	-0.6209670	-1.9239170	C	-5.1441310	1.9846730	-0.0971470
C	-4.8166420	2.2527730	-0.4563500	H	-4.7000220	2.3791310	-1.0163180
H	-5.8468780	2.6055580	-0.3375830	H	-6.2076350	2.2465960	-0.0987650
H	-4.1609920	2.9232890	0.1079420	H	-4.6755900	2.4872420	0.7546570
H	-4.5542560	2.3293690	-1.5162910	C	-5.6675810	-0.7543160	-1.4786740
C	-5.0014370	0.3366870	1.9898350	H	-6.7505170	-0.5954050	-1.5220280
H	-6.0356830	0.6265230	2.2039080	H	-5.2314850	-0.3778510	-2.4092410
H	-4.8615020	-0.6909140	2.3395200	H	-5.4864990	-1.8326100	-1.4275700
H	-4.3425430	0.9900240	2.5700840	Si	-4.9438510	0.1239160	0.0138650

References

(1) Gaussian 09, Revision D.01, M. J. Frisch, G. W. Trucks, H. B. Schlegel, G. E. Scuseria, M. A. Robb, J. R. Cheeseman, G. Scalmani, V. Barone, B. Mennucci, G. A. Petersson, H. Nakatsuji, M. Caricato, X. Li, H. P. Hratchian, A. F. Izmaylov, J. Bloino, G. Zheng, J. L. Sonnenberg, M. Hada, M. Ehara, K. Toyota, R. Fukuda, J. Hasegawa, M. Ishida, T. Nakajima, Y. Honda, O. Kitao, H. Nakai, T. Vreven, J. A. Montgomery, Jr., J. E. Peralta, F. Ogliaro, M. Bearpark, J. J. Heyd, E. Brothers, K. N. Kudin, V. N. Staroverov, T. Keith, R. Kobayashi, J. Normand, K. Raghavachari, A. Rendell, J. C. Burant, S. S. Iyengar, J. Tomasi, M. Cossi, N. Rega, J. M. Millam, M. Klene, J. E. Knox, J. B. Cross, V. Bakken, C. Adamo, J. Jaramillo, R. Gomperts, R. E. Stratmann, O. Yazyev, A. J. Austin, R. Cammi, C. Pomelli, J. W. Ochterski, R. L. Martin, K. Morokuma, V. G. Zakrzewski, G. A. Voth, P. Salvador, J. J. Dannenberg, S. Dapprich, A. D. Daniels, O. Farkas, J. B. Foresman, J. V. Ortiz, J. Cioslowski, and D. J. Fox, Gaussian, Inc., Wallingford CT, **2013**..

(2) (a) Becke, A. D. *J. Chem. Phys*. **1993**, *98*, 5648–5652. (b) Becke, A. D. *Phys. Rev. A* **1988**, *38*, 3098–3100. (c) Lee, C.; Yang, W.; Parr, R. G. *Phys. Rev. B* **1988**, *37*, 785–789.

(3) (a) Grimme, S.; Antony, J.; Ehrlich, S.; Krieg, H. *J. Chem. Phys*. **2010**, *132*, 1054104. (b) Grimme, S.; Ehrlich, S.; Goerigk, L. *J. Comput. Chem.* **2011**, *32*, 1456–1465.

(4) Marenich, A. V.; Cramer, C. J.; Truhlar, D. G. *J. Phys. Chem. B*, **2009**, *11* , 6378–6396.

(5) Gonzalez, C.; Schlegel, H. B. *J. Chem. Phys*. **1989**, *90*, 2154-2161.

Figure S3. Energy Profile for the Nitrile Oxide Formation Reactions under HCl Catalysis[a]

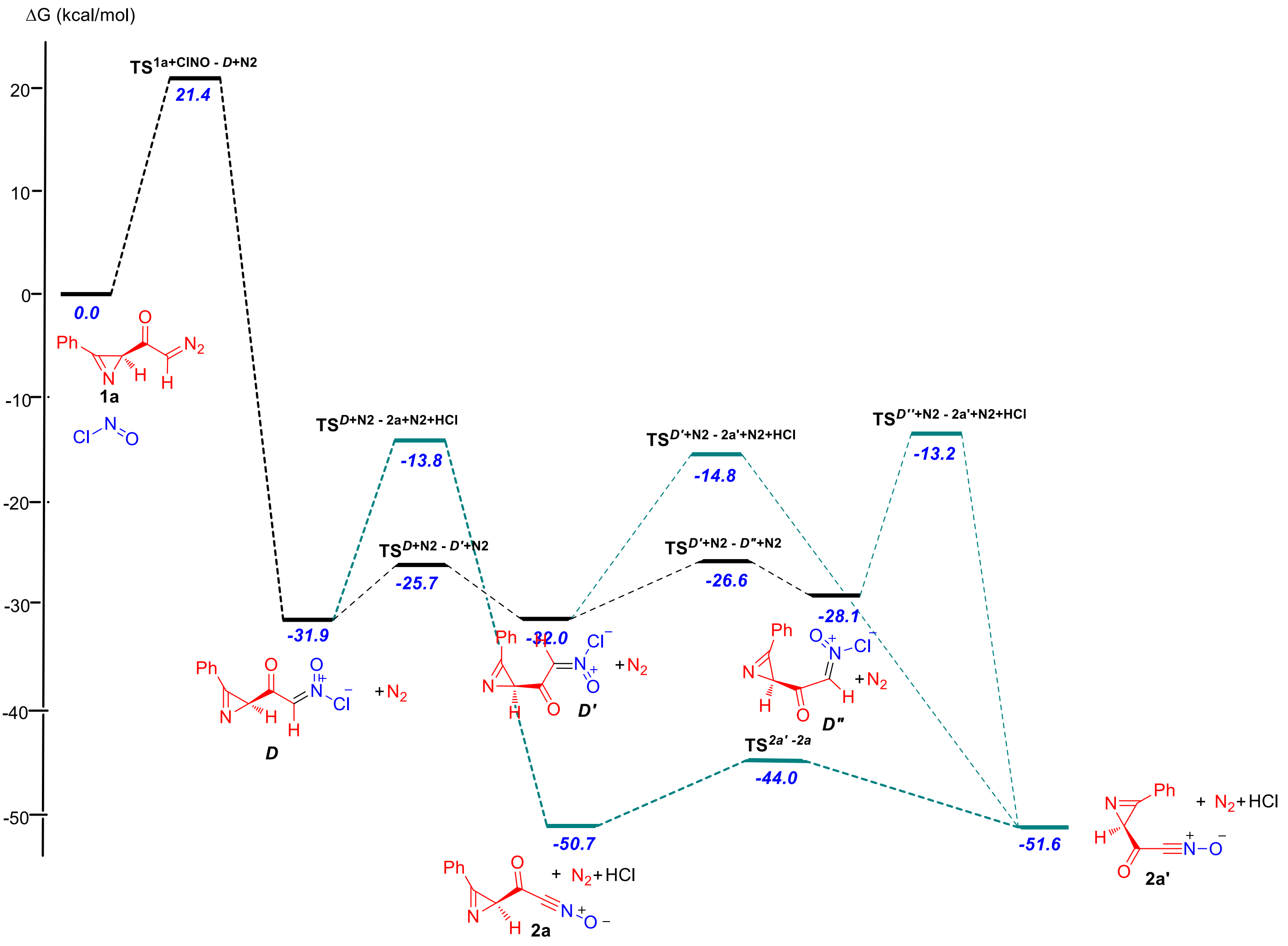

[a] DFT B3LYP-D3/6-311+G(d,p) level with SMD model for DCM, relative Gibbs free energies in kcal/mol, 298 K.
