# Supplementary material for: Azirinyl-Substituted Nitrile Oxides: Generation and Use in the Synthesis of Isoxazole Containing Heterocyclic Hybrids
Source: Molecules. 2025 Jul 2;30(13):2834. doi: 10.3390/molecules30132834 (PMC12250923; doi:10.3390/molecules30132834)

checkCIF/PLATON report

Structure factors have been supplied for datablock(s) hb_2

THIS REPORT IS FOR GUIDANCE ONLY. IF USED AS PART OF A REVIEW PROCEDURE FOR PUBLICATION, IT SHOULD NOT REPLACE THE EXPERTISE OF AN EXPERIENCED CRYSTALLOGRAPHIC REFEREE.

No syntax errors found.   CIF dictionary   Interpreting this report

Datablock: hb_2

```
Bond precision:   C-C = 0.0078 A          Wavelength=1.54184

Cell:              a=15.3451(2)      b=15.3451(2)      c=10.2061(2)
                   alpha=90          beta=90           gamma=90
Temperature:       100 K

                 Calculated                    Reported
Volume           2403.25(8)                    2403.25(8)
Space group      P -4 21 c                     P -4 21 c
Hall group       P -4 2n                       P -4 2n
Moiety formula   C13 H9 Cl N2 O2               C13 H9 Cl N2 O2
Sum formula      C13 H9 Cl N2 O2               C13 H9 Cl N2 O2
Mr               260.67                        260.67
Dx,g cm-3        1.441                         1.441
Z                8                             8
Mu (mm-1)        2.786                         2.786
F000             1072.0                        1072.0
F000'            1077.92
h,k,lmax         18,18,12                      18,18,12
Nref             2314[ 1317]                   2303
Tmin,Tmax        0.818,0.895                   0.870,1.000
Tmin'            0.757

Correction method= # Reported T Limits: Tmin=0.870 Tmax=1.000
AbsCorr = MULTI-SCAN

Data completeness= 1.75/1.00       Theta(max)= 70.643

                                                    wR2(reflections)=
R(reflections)= 0.0554( 2220)                       0.1373( 2303)

S = 1.058                  Npar= 157
```

The following ALERTS were generated. Each ALERT has the format
test-name_ALERT_alert-type_alert-level.
Click on the hyperlinks for more details of the test.

Alert level C

```
PLAT340_ALERT_3_C Low Bond Precision on  C-C Bonds ..............    0.00785 Ang.
```

Alert level G

```
PLAT171_ALERT_4_G The CIF-Embedded .res File Contains EADP Records          1 Report
PLAT395_ALERT_2_G Deviating  X-O-Y   Angle From 120 for O1       .      108.8 Degree
PLAT792_ALERT_1_G Model has Chirality at C6          (Polar  SpGr)          R Verify
PLAT912_ALERT_4_G Missing # of FCF Reflections Above STh/L=  0.600          1 Note
PLAT967_ALERT_5_G Note: Two-Theta Cutoff Value in Embedded .res ..      145.0 Degree
PLAT969_ALERT_5_G The 'Henn et al.' R-Factor-gap value ..........      3.146 Note
              Predicted wR2: Based on SigI**2  4.36 or SHELX Weight 12.97
PLAT978_ALERT_2_G Number C-C Bonds with Positive Residual Density.          2 Info
```

0 **ALERT level A** = Most likely a serious problem - resolve or explain
0 **ALERT level B** = A potentially serious problem, consider carefully
1 **ALERT level C** = Check. Ensure it is not caused by an omission or oversight
7 **ALERT level G** = General information/check it is not something unexpected

1 ALERT type 1 CIF construction/syntax error, inconsistent or missing data
2 ALERT type 2 Indicator that the structure model may be wrong or deficient
1 ALERT type 3 Indicator that the structure quality may be low
2 ALERT type 4 Improvement, methodology, query or suggestion
2 ALERT type 5 Informative message, check

It is advisable to attempt to resolve as many as possible of the alerts in all categories. Often the minor alerts point to easily fixed oversights, errors and omissions in your CIF or refinement strategy, so attention to these fine details can be worthwhile. In order to resolve some of the more serious problems it may be necessary to carry out additional measurements or structure refinements. However, the purpose of your study may justify the reported deviations and the more serious of these should normally be commented upon in the discussion or experimental section of a paper or in the "special_details" fields of the CIF. checkCIF was carefully designed to identify outliers and unusual parameters, but every test has its limitations and alerts that are not important in a particular case may appear. Conversely, the absence of alerts does not guarantee there are no aspects of the results needing attention. It is up to the individual to critically assess their own results and, if necessary, seek expert advice.

Publication of your CIF in IUCr journals

A basic structural check has been run on your CIF. These basic checks will be run on all CIFs submitted for publication in IUCr journals (*Acta Crystallographica*, *Journal of Applied Crystallography*, *Journal of Synchrotron Radiation*); however, if you intend to submit to *Acta Crystallographica Section C* or *E* or *IUCrData*, you should make sure that full publication checks are run on the final version of your CIF prior to submission.

Publication of your CIF in other journals

Please refer to the *Notes for Authors* of the relevant journal for any special instructions relating to CIF submission.

PLATON version of 11/11/2024; check.def file version of 11/11/2024

Datablock hb_2 - ellipsoid plot

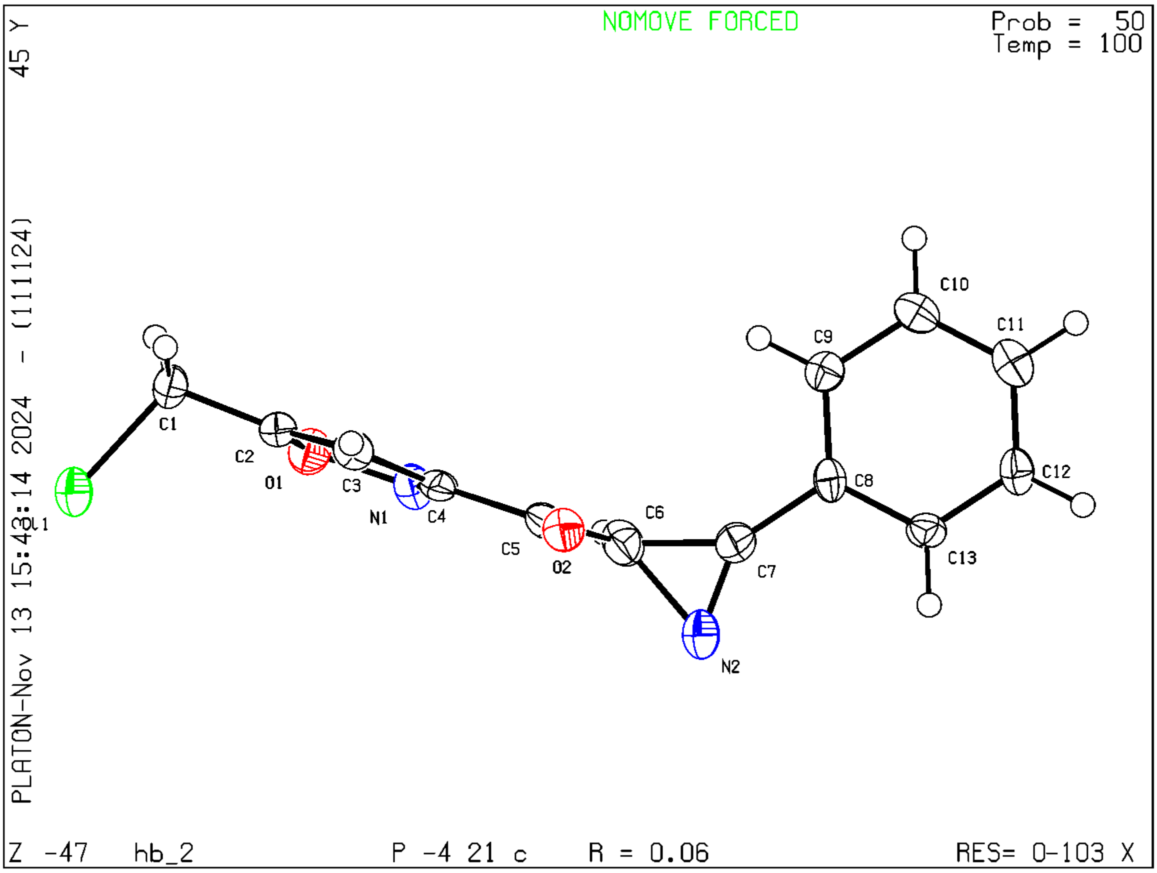

Supplement: Supplementary file 1 [file molecules-30-02834-s001.zip › checkcif 3g.pdf]
