# Supplementary material for: Azirinyl-Substituted Nitrile Oxides: Generation and Use in the Synthesis of Isoxazole Containing Heterocyclic Hybrids
Source: Molecules. 2025 Jul 2;30(13):2834. doi: 10.3390/molecules30132834 (PMC12250923; doi:10.3390/molecules30132834)

checkCIF/PLATON report

Structure factors have been supplied for datablock(s) hb_1

THIS REPORT IS FOR GUIDANCE ONLY. IF USED AS PART OF A REVIEW PROCEDURE FOR PUBLICATION, IT SHOULD NOT REPLACE THE EXPERTISE OF AN EXPERIENCED CRYSTALLOGRAPHIC REFEREE.

No syntax errors found. CIF dictionary Interpreting this report

Datablock: hb_1

Bond precision:	C-C = 0.0015 A		Wavelength=1.54184
Cell:	a=13.5255(3)	b=5.7310(1)	c=15.3411(3)
	alpha=90	beta=92.936(2)	gamma=90
Temperature:	100 K		

	Calculated	Reported
Volume	1187.60(4)	1187.60(4)
Space group	P 21/c	P 1 21/c 1
Hall group	-P 2ybc	-P 2ybc
Moiety formula	C14 H10 N2 O4	C14 H10 N2 O4
Sum formula	C14 H10 N2 O4	C14 H10 N2 O4
Mr	270.24	270.24
Dx,g cm-3	1.511	1.511
Z	4	4
Mu (mm-1)	0.952	0.952
F000	560.0	560.0
F000'	561.95	
h,k,lmax	16,7,18	16,7,18
Nref	2345	2328
Tmin,Tmax	0.859,0.909	0.493,1.000
Tmin'	0.859	

Correction method= # Reported T Limits: Tmin=0.493 Tmax=1.000
AbsCorr = MULTI-SCAN

Data completeness= 0.993 Theta(max)= 72.431

R(reflections)= 0.0349(2159) wR2(reflections)= 0.0969(2328)

S = 1.090 Npar= 182

The following ALERTS were generated. Each ALERT has the format
test-name_ALERT_alert-type_alert-level.
Click on the hyperlinks for more details of the test.

Alert level C

```
PLAT911_ALERT_3_C Missing FCF Refl Between Thmin & STh/L=    0.600          4 Report
               16  0  0,  -3  2  3,    3  1  5,    3  3 15,
```

Alert level G

```
PLAT395_ALERT_2_G Deviating  X-O-Y   Angle From 120 for O1       .      108.0 Degree
PLAT395_ALERT_2_G Deviating  X-O-Y   Angle From 120 for O2       .      108.2 Degree
PLAT912_ALERT_4_G Missing # of FCF Reflections Above STh/L=  0.600         13 Note
PLAT933_ALERT_2_G Number of HKL-OMIT Records in Embedded .res File          3 Note
               -3  2  3,    3  1  5,   12  5  2,
PLAT941_ALERT_3_G Average HKL Measurement Multiplicity ..........         3.2 Low
PLAT967_ALERT_5_G Note: Two-Theta Cutoff Value in Embedded .res ..      145.0 Degree
PLAT969_ALERT_5_G The 'Henn et al.' R-Factor-gap value ..........      3.973 Note
              Predicted wR2: Based on SigI**2  2.44 or SHELX Weight  8.89
PLAT978_ALERT_2_G Number C-C Bonds with Positive Residual Density.         13 Info
PLAT992_ALERT_5_G Repd & Actual _reflns_number_gt Values Differ by          2 Check
```

0 **ALERT level A** = Most likely a serious problem - resolve or explain
0 **ALERT level B** = A potentially serious problem, consider carefully
1 **ALERT level C** = Check. Ensure it is not caused by an omission or oversight
9 **ALERT level G** = General information/check it is not something unexpected

0 ALERT type 1 CIF construction/syntax error, inconsistent or missing data
4 ALERT type 2 Indicator that the structure model may be wrong or deficient
2 ALERT type 3 Indicator that the structure quality may be low
1 ALERT type 4 Improvement, methodology, query or suggestion
3 ALERT type 5 Informative message, check

It is advisable to attempt to resolve as many as possible of the alerts in all categories. Often the minor alerts point to easily fixed oversights, errors and omissions in your CIF or refinement strategy, so attention to these fine details can be worthwhile. In order to resolve some of the more serious problems it may be necessary to carry out additional measurements or structure refinements. However, the purpose of your study may justify the reported deviations and the more serious of these should normally be commented upon in the discussion or experimental section of a paper or in the "special_details" fields of the CIF. checkCIF was carefully designed to identify outliers and unusual parameters, but every test has its limitations and alerts that are not important in a particular case may appear. Conversely, the absence of alerts does not guarantee there are no aspects of the results needing attention. It is up to the individual to critically assess their own results and, if necessary, seek expert advice.

Publication of your CIF in IUCr journals

A basic structural check has been run on your CIF. These basic checks will be run on all CIFs submitted for publication in IUCr journals (*Acta Crystallographica*, *Journal of Applied Crystallography*, *Journal of Synchrotron Radiation*); however, if you intend to submit to *Acta Crystallographica Section C* or *E* or *IUCrData*, you should make sure that full publication checks are run on the final version of your CIF prior to submission.

Publication of your CIF in other journals

Please refer to the *Notes for Authors* of the relevant journal for any special instructions relating to CIF submission.

PLATON version of 22/08/2024; check.def file version of 21/08/2024

Datablock hb_1 - ellipsoid plot

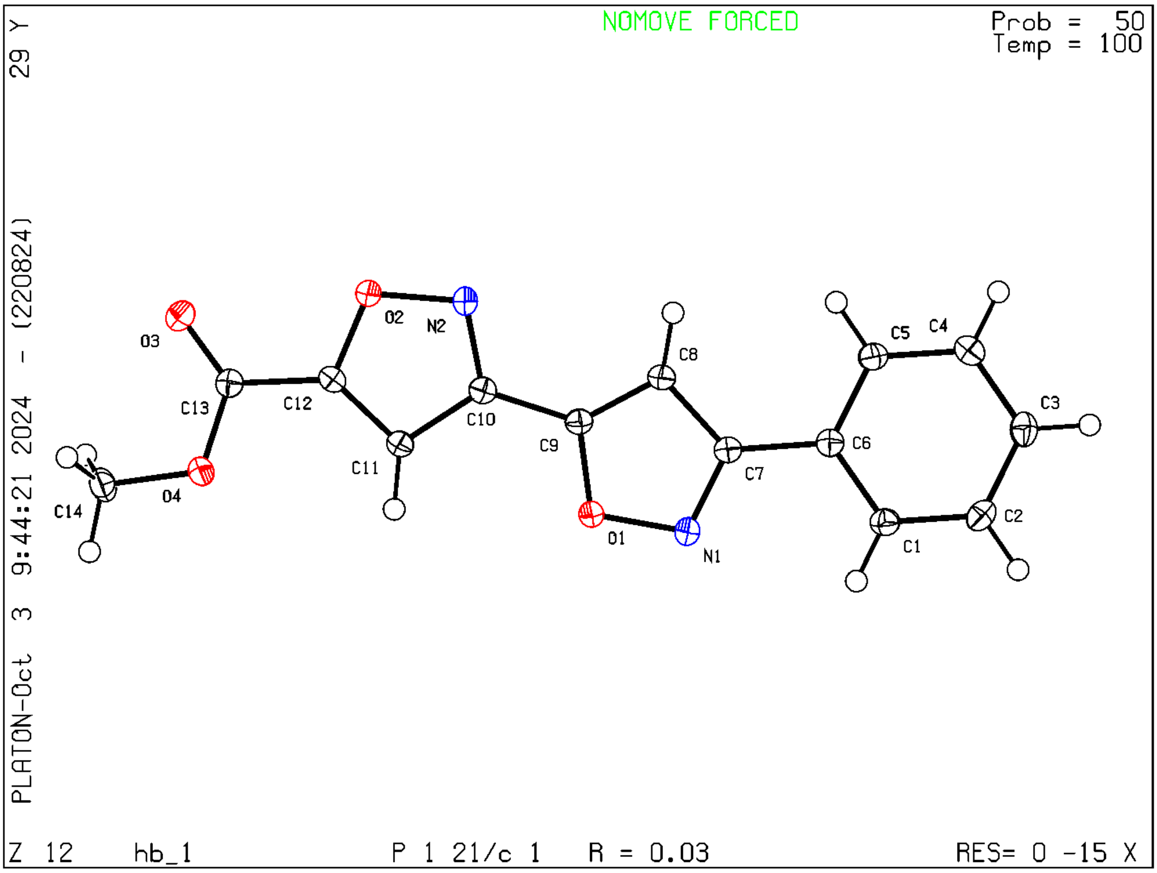

Supplement: Supplementary file 1 [file molecules-30-02834-s001.zip › checkcif 4a.pdf]
